# Supplementary material for: Global O-glycoproteome enrichment and analysis enabled by a combinatorial enzymatic workflow
Source: Cell Rep Methods. 2024 Apr 5;4(4):100744. doi: 10.1016/j.crmeth.2024.100744 (PMC11046030; doi:10.1016/j.crmeth.2024.100744)

Cell Reports Methods, Volume *4*

Supplemental information

Global O-glycoproteome enrichment and analysis enabled by a combinatorial enzymatic workflow

Taewook Kang, Rohit Budhraja, Jinyong Kim, Neha Joshi, Kishore Garapati, and Akhilesh Pandey

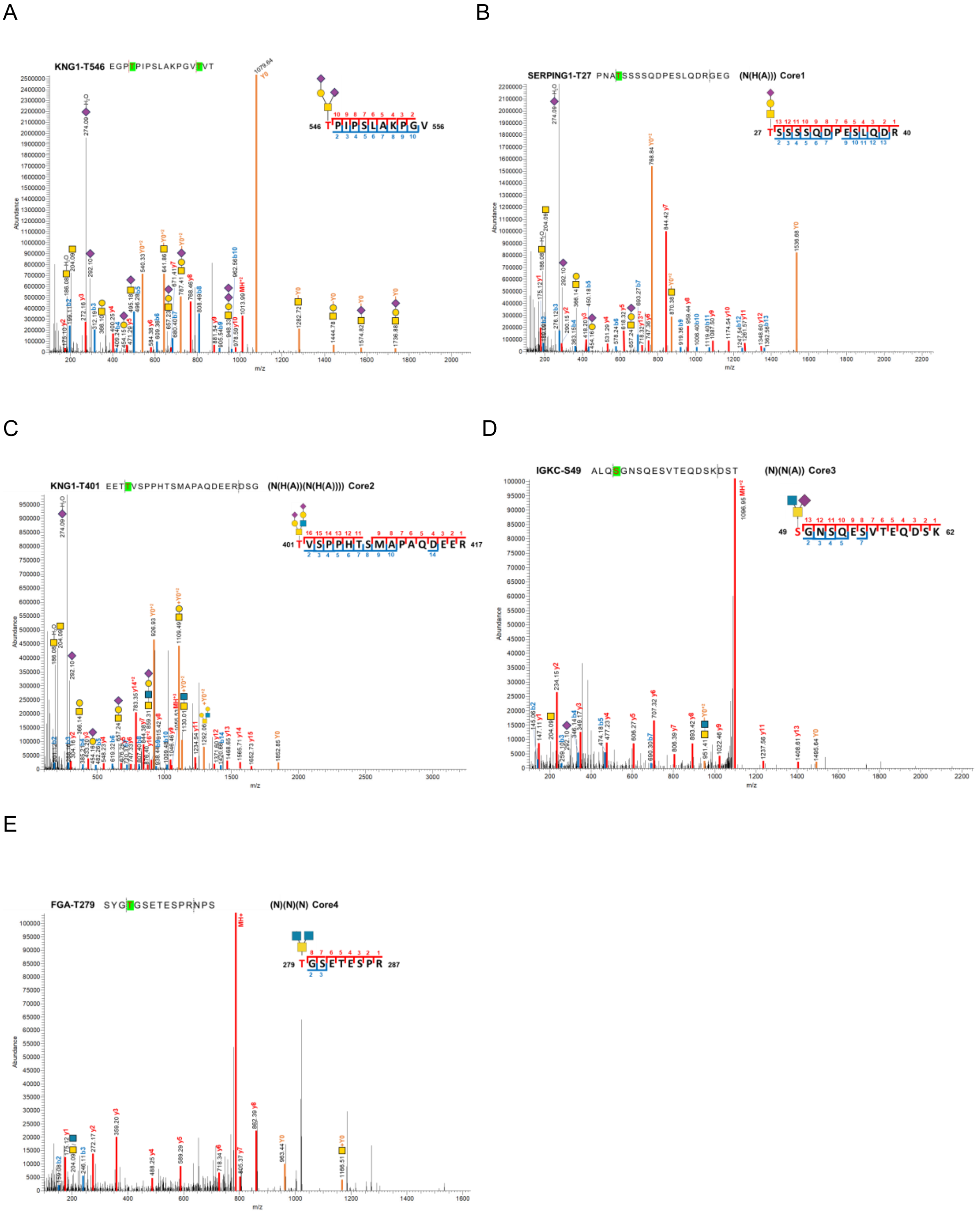

Figure S1 related to figure 4 and STAR methods. Representative MS/MS spectra of a glycopeptide showing (A) the use of sceHCD and the specificity of IMPa approach to localize the O-glycosylation site, (B) Core 1 structure, (C) Core 2 structure, (D) Core 1 structure, (E) Core 2 structure.

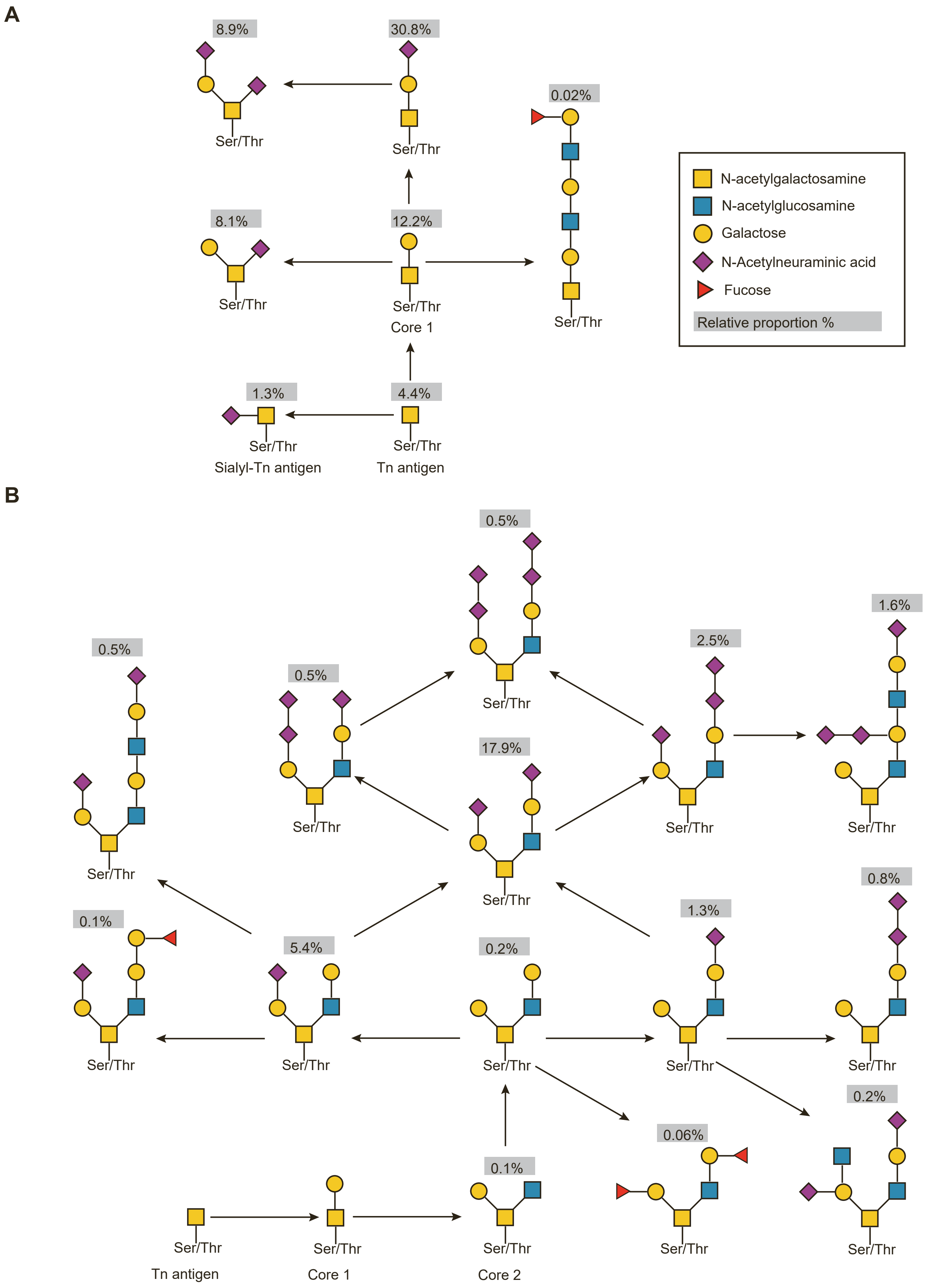

Figure S2 related to figure 4. Relative proportions of identified unique O-glycan structures. (A) Illustration of identified core 1 glycan structures. (B) Illustration of identified core 2 glycan structures

Data S1. MS/MS Spectra of identified O-glycopeptides in proteoglycan 4 (PRG4), related to Figure 3.

MS/MS spectra of identified O-glycopeptides in proteoglycan 4 (PRG4)

```
DOMAIN          26-69   Somatomedin B (SMB) 1
DOMAIN         66-108   Somatomedin B (SMB) 2
REGION        348-855   59 X 8 AA repeats of K-X-P-X-P-T-T-X
REPEAT      1148-1191   Hemopexin (PEX) 1
REPEAT      1192-1239   Hemopexin (PEX) 2
```

S/T *O*-glycosylation

X Identified sequences

KP**T**PKPPVV**D** **E**AGSGLDNGD FKVTTPDTST

TQHNKVSTSP **K**I**T**TAKPINP RP**S**LPPNSDT

S**K**ETSLTVNK E**T**TVETK**E**TT TTNKQTSTDG

KEKTTSAKET QSIEKTSAKD LAP**T**SKVLAK

P**T**PKAETTTK GPAL**TT**PKEP TPTTPKEPAS

TTPKEPTP**T**T IK**S**AP**T**TPKE PAPTTTKSAP

TTPKEPAP**TT** TKEPAP**T**TPK EPAP**TT**TKEP

APTTTKSAP**T** TPKEPAPTTP KKPAP**T**TPKE

PAP**TT**PKEP**T**	P**T**TPKEPAPT	TKEPAP**T**TPK
EPAPTAPKKP	AP**T**TPKEPAP	**T**TPKEPAPTT
TKEPSP**T**TPK	EPAPTTTKSA	P**TT**TKEPAPT
TTK**S**APTTPK	EPSP**TT**TKEP	AP**T**TPKEPAP
TTPKKPAP**T**T	PKEPAP**T**TPK	EPAP**T**TTKKP
AP**T**TPKEPAP	**T**TPKETAP**T**T	PKKLTP**T**TPE
KLAP**T**TPEKP	APTTPEELAP	**T**TPEEP**T**PTT
PEEPAP**TT**PK	AAAPNTPKEP	AP**T**TPKEPAP
**T**TPKEPAP**T**T	PKETAPTTPK	G**T**APTTLKEP
AP**T**TPKKPAP	**KE**LAPTTTKE	PTS**T**TSDKPA
P**T**TPKG**T**AP**T**	TPKEPAP**T**TP	KEPAPTTPKG
TAPTTLKEPA	P**T**TPKKPAP**K**	**E**LAPTTTKGP
TS**T**TSDKPAP	**T**TPKE**T**AP**T**T	PKEPAP**T**TPK

KPAPTTPETP PPTTSEVSTP TTTKEPT**T**IH

KSPD**ES**TPEL SAEP**T**PKAL**E** NSP**K**EPGVP**T**

TKTPAA**T**KPE M**T**TTAKDKTT **E**RDLRTTPE**T**

TTAAPKMTK**E** TATTTEKTTE SKITATTTQV

TSTTTQDTTP FKITTLKTTT LAPKVTTTKK

TITTTEIMNK PEETAKP**K**DR ATNSKATTPK

PQKP**T**KAPKK PTS**T**KKPKTM PRVRKPKTTP

TPRKMTSTMP ELNPTSRIAE AMLQTTTRPN

Q**T**PNSKLV**E**V

PRG4–T213, H (1) N (1) A (1)

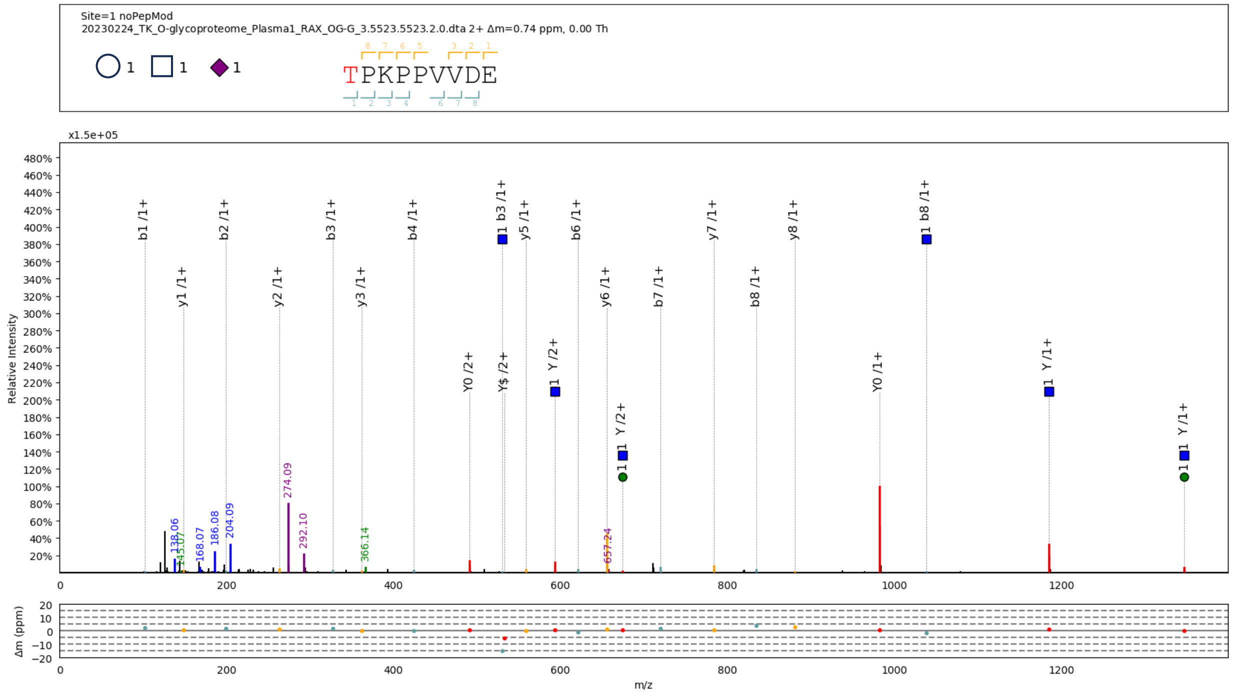

PRG4–T253, H (2) N (2) A (2)

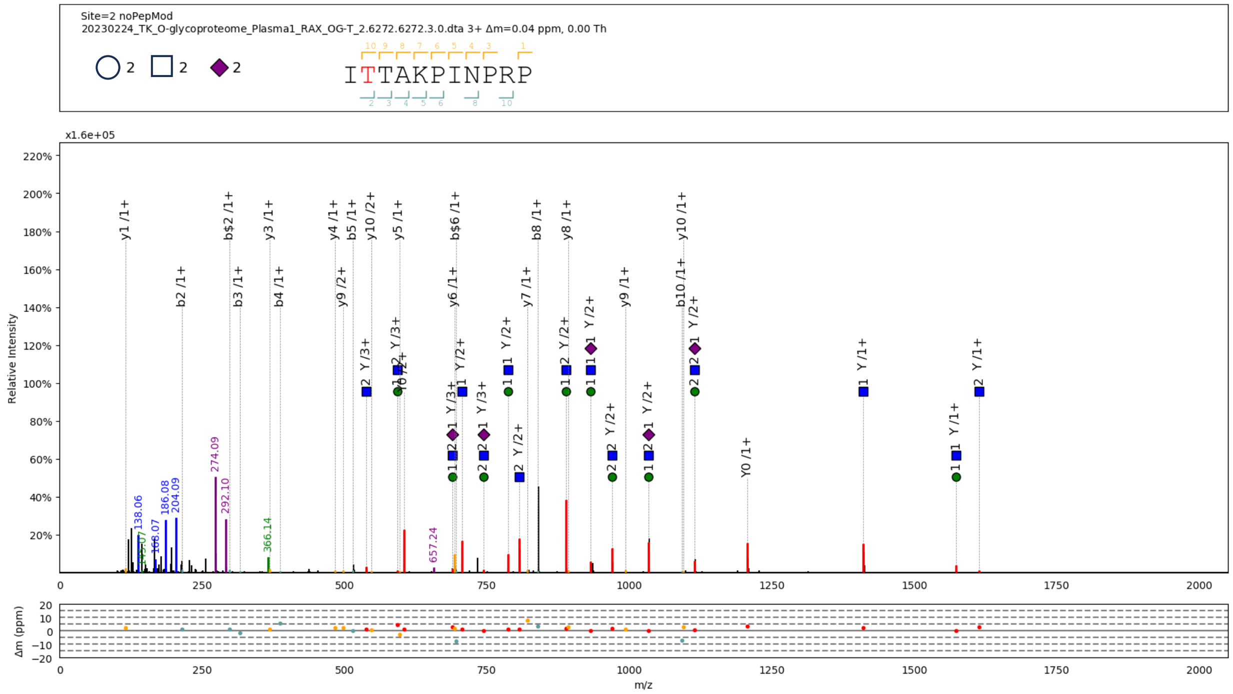

PRG4−T253, H (1) N (1) A (1)

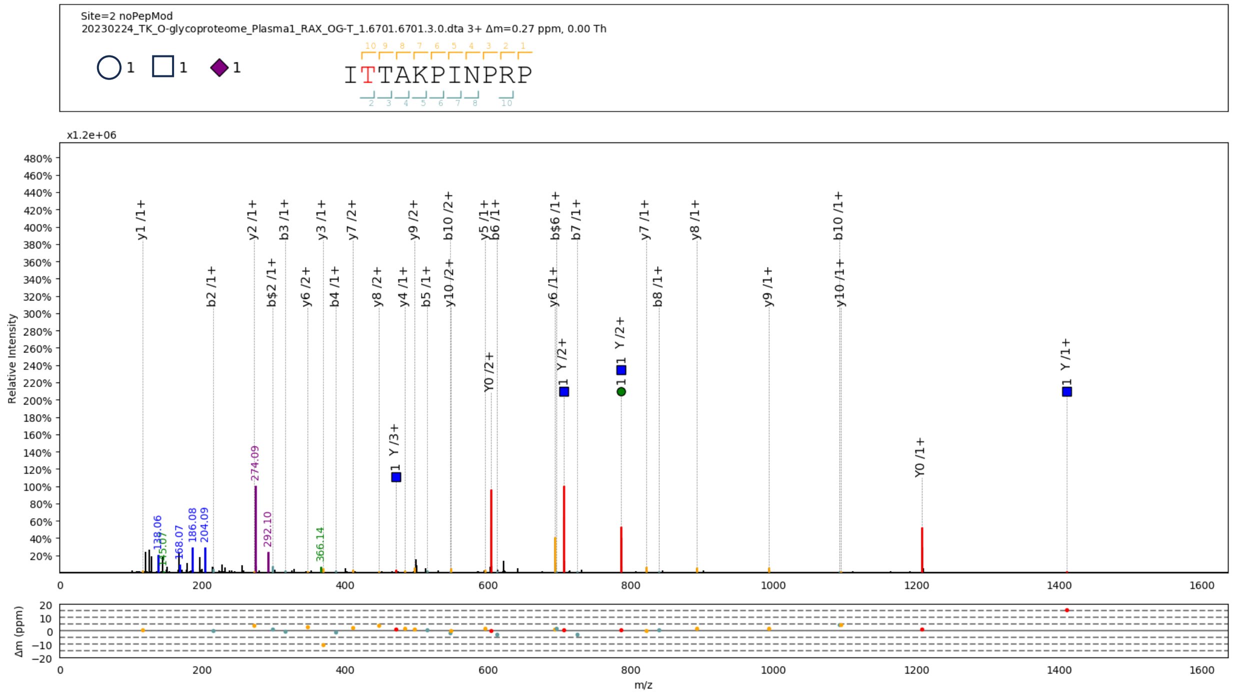

PRG4−T253, H (1) N (1)

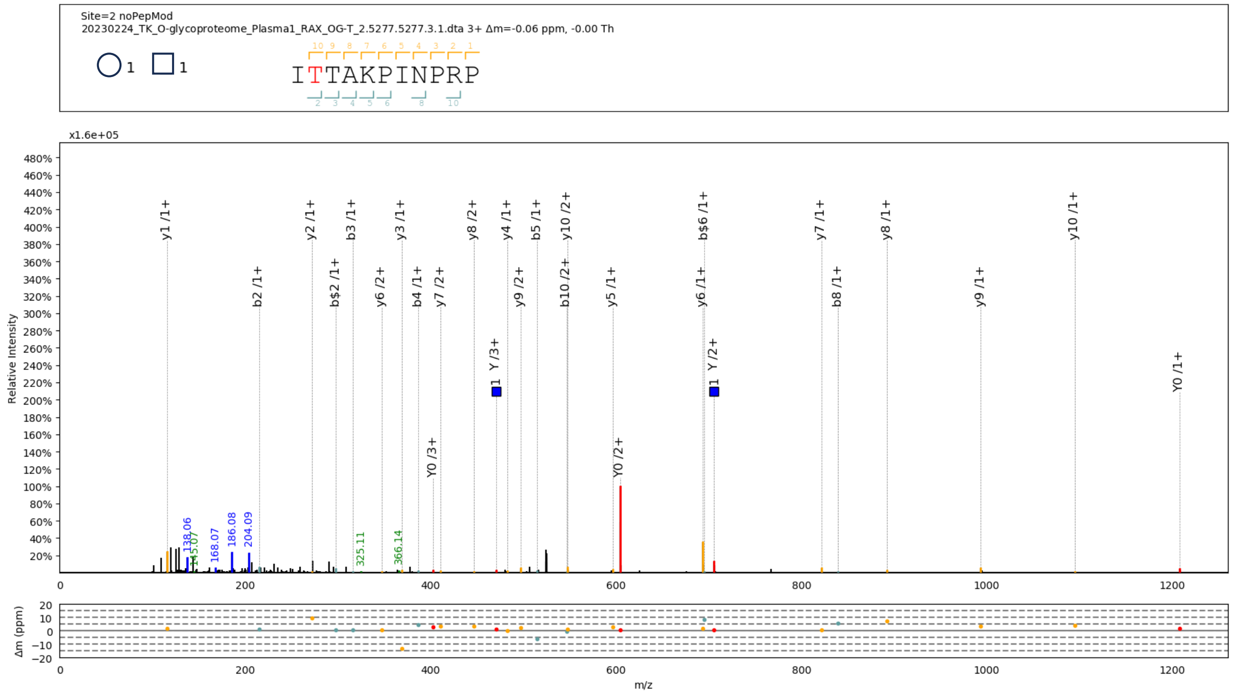

PRG4−S263, H (1) N (1) A (1)

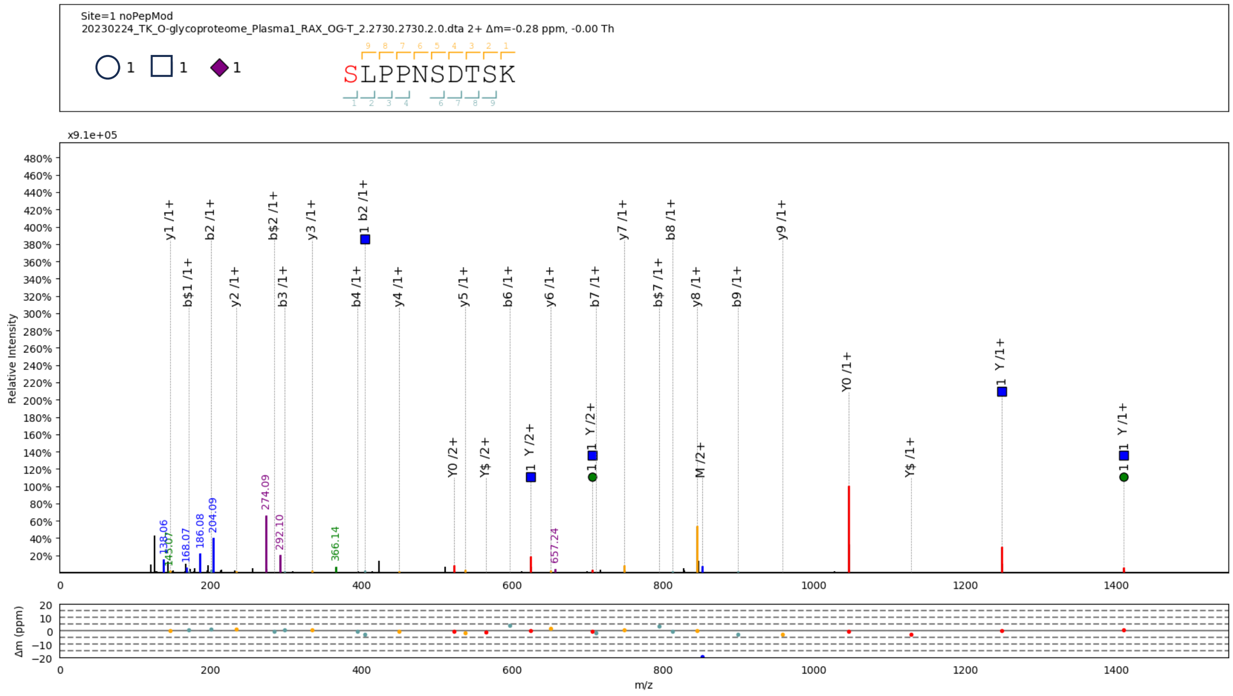

PRG4−T282, H (1) N (1) A (1)

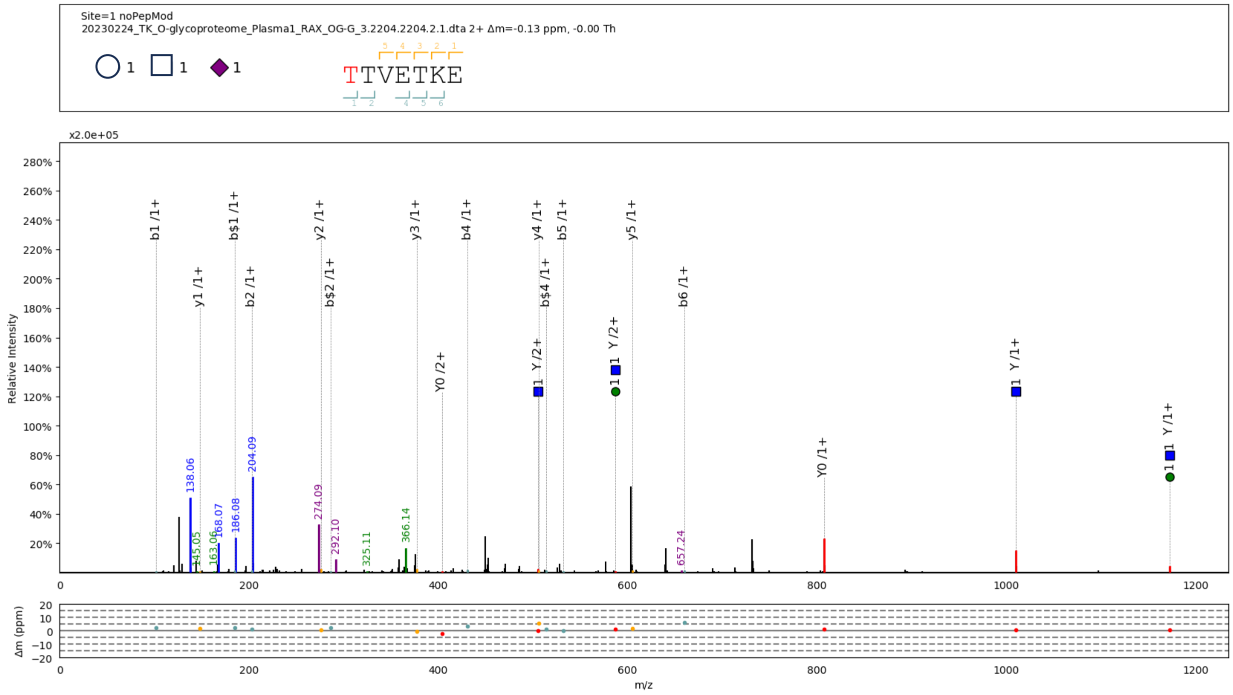

PRG4–T324, H (1) N (1) A (1)

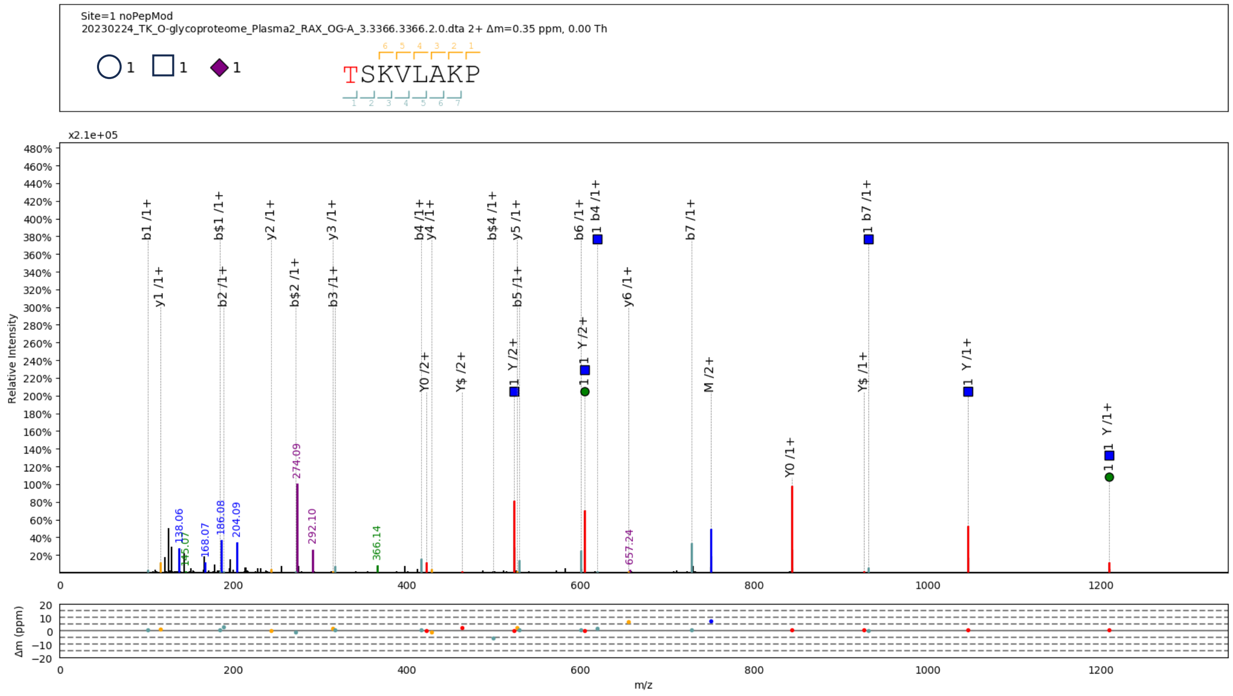

PRG4–T324, H (1) N (1)

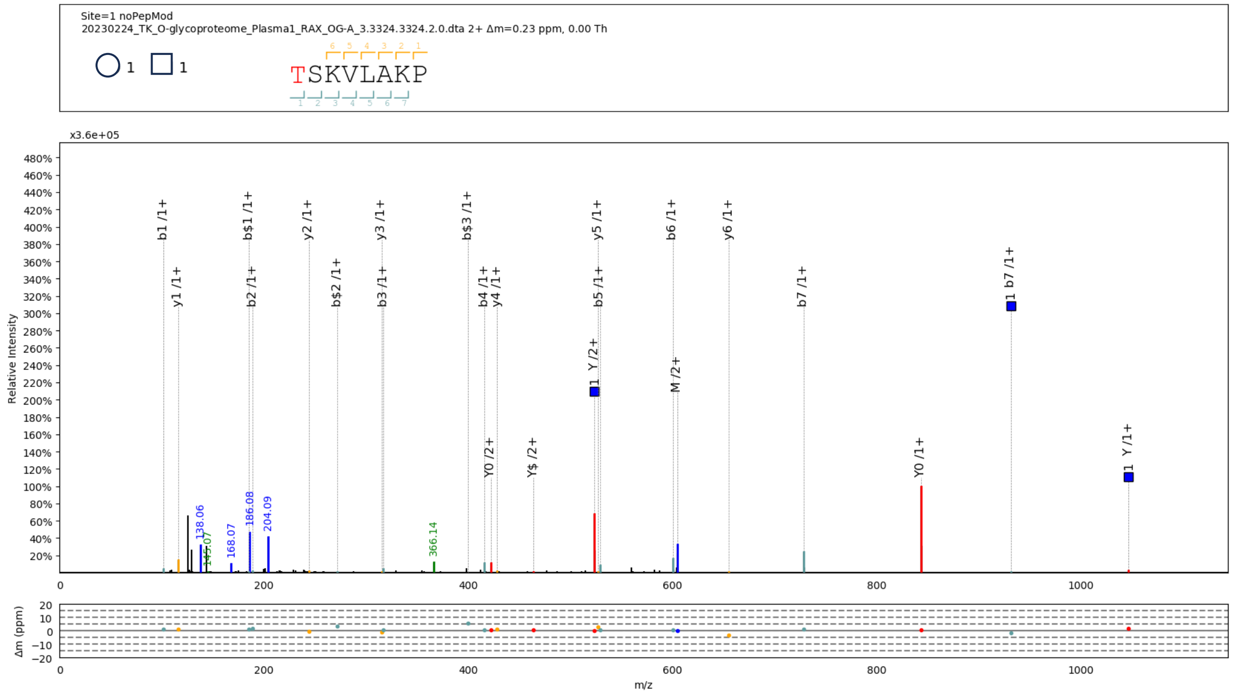

PRG4–T324, N (1)

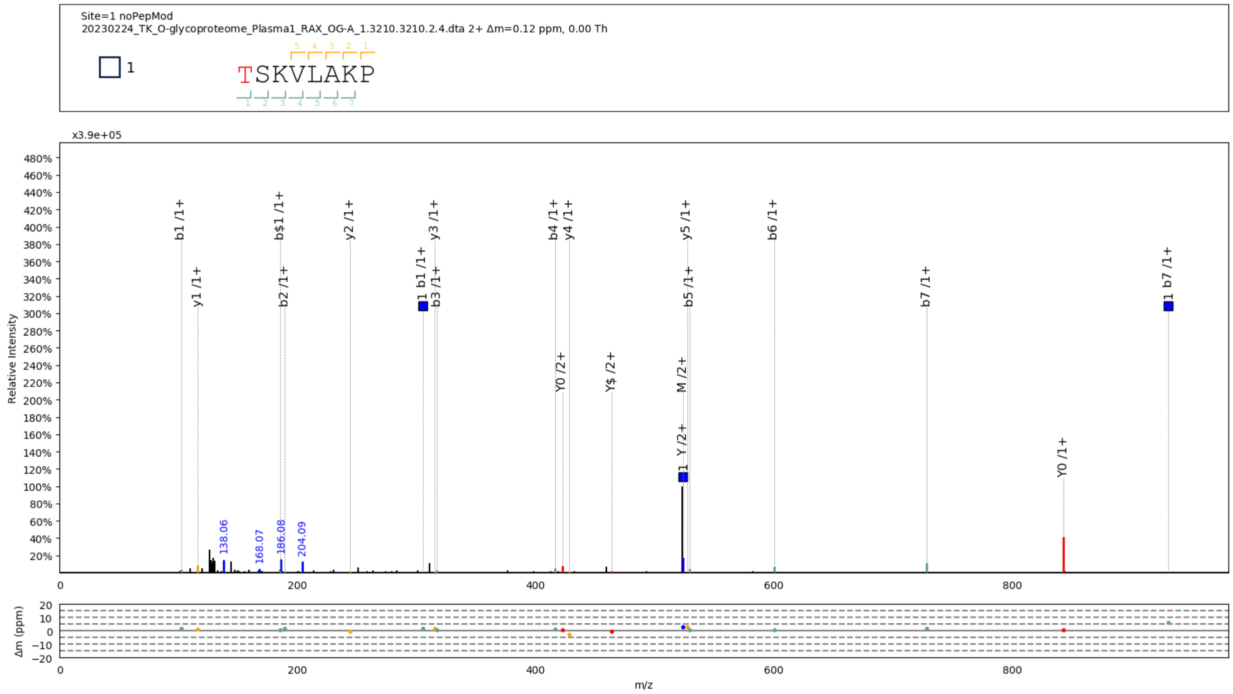

PRG4–T332, H (1) N (1) A (1)

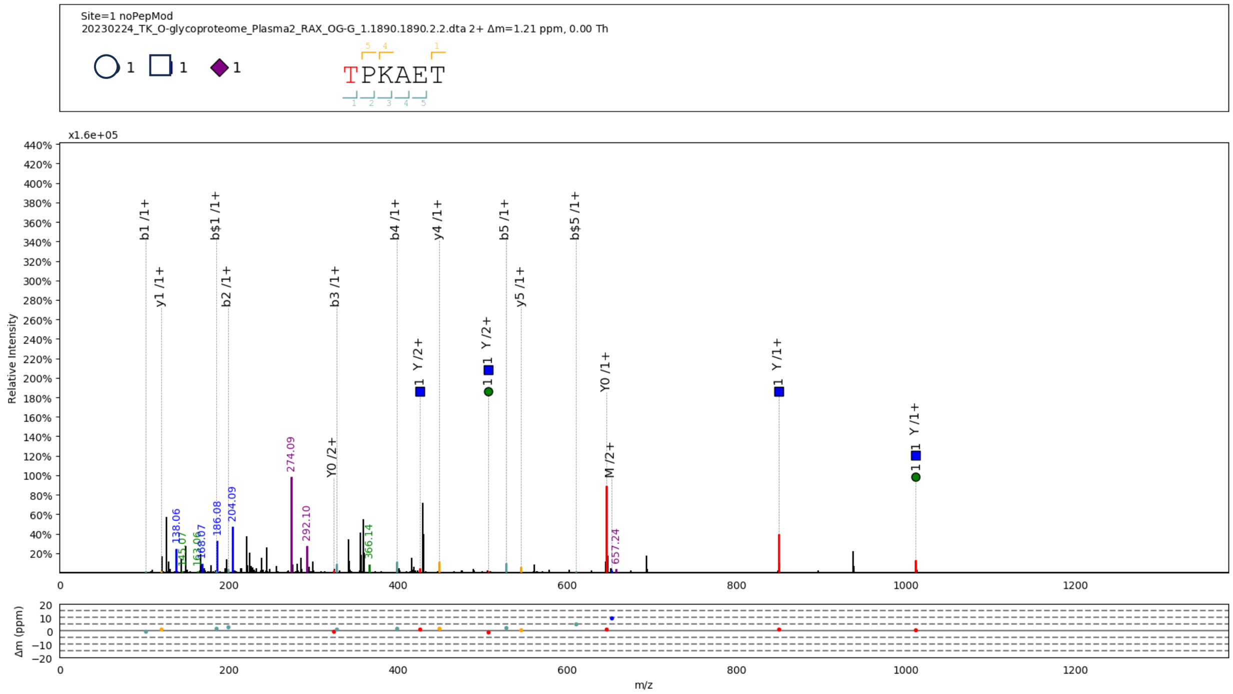

PRG4–T345-T361-T454, H (2) N (2) A (2)

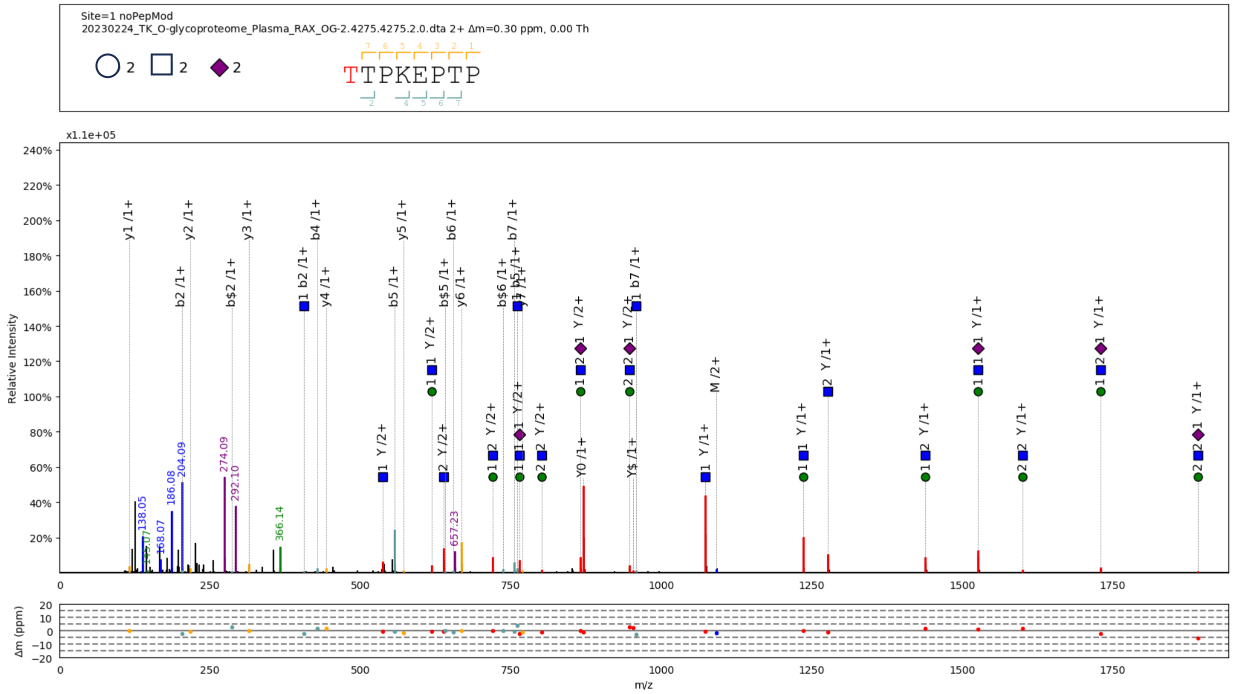

PRG4–T346-T362-T455, H (1) N (1) A (1)

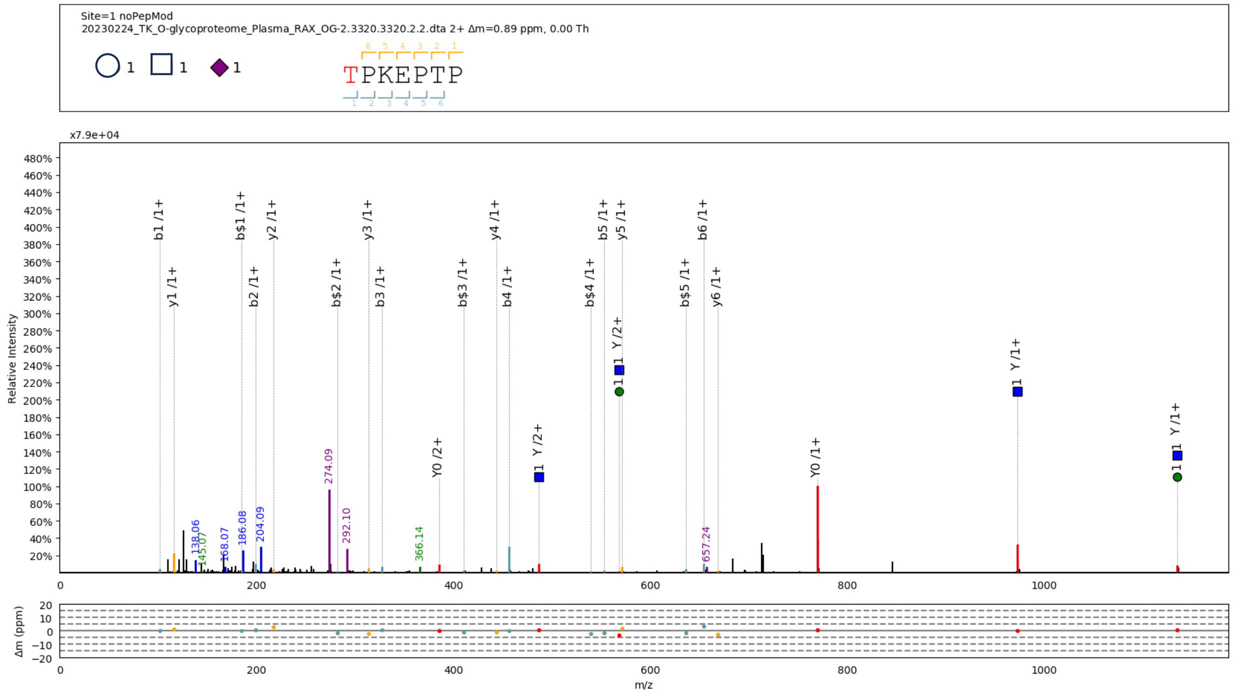

PRG4–T369, H (2) N (2) A (2)

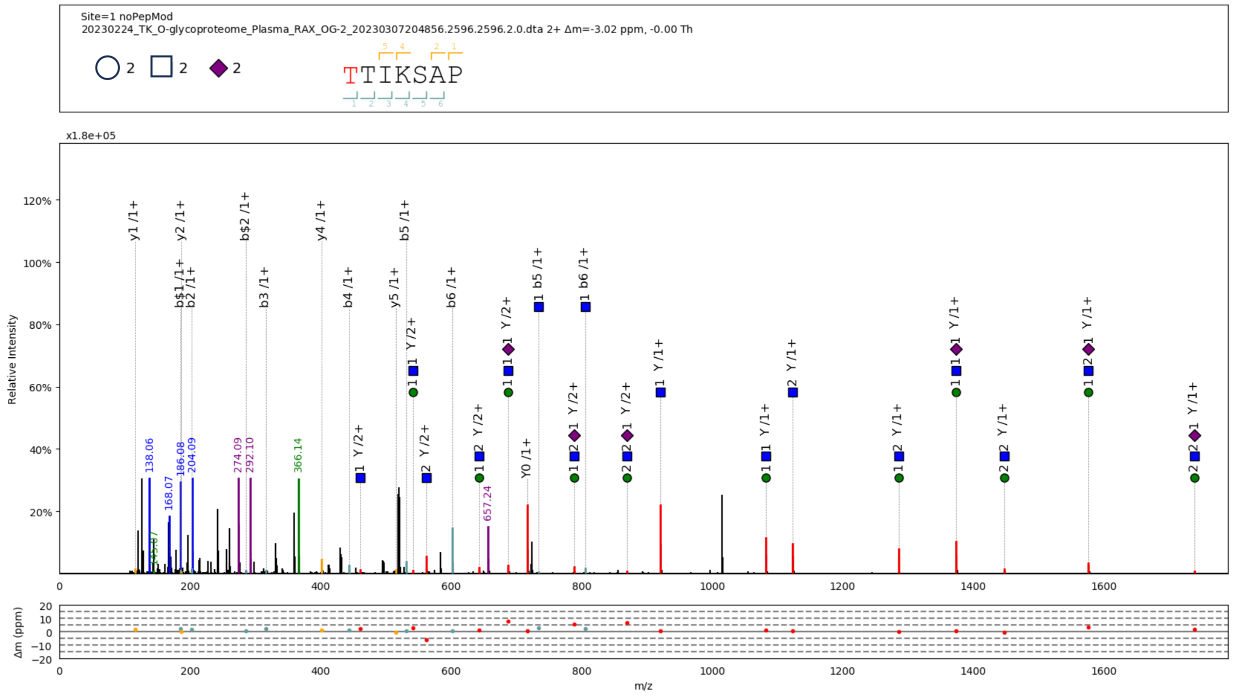

PRG4–T369, H (1) N (1) A (1)

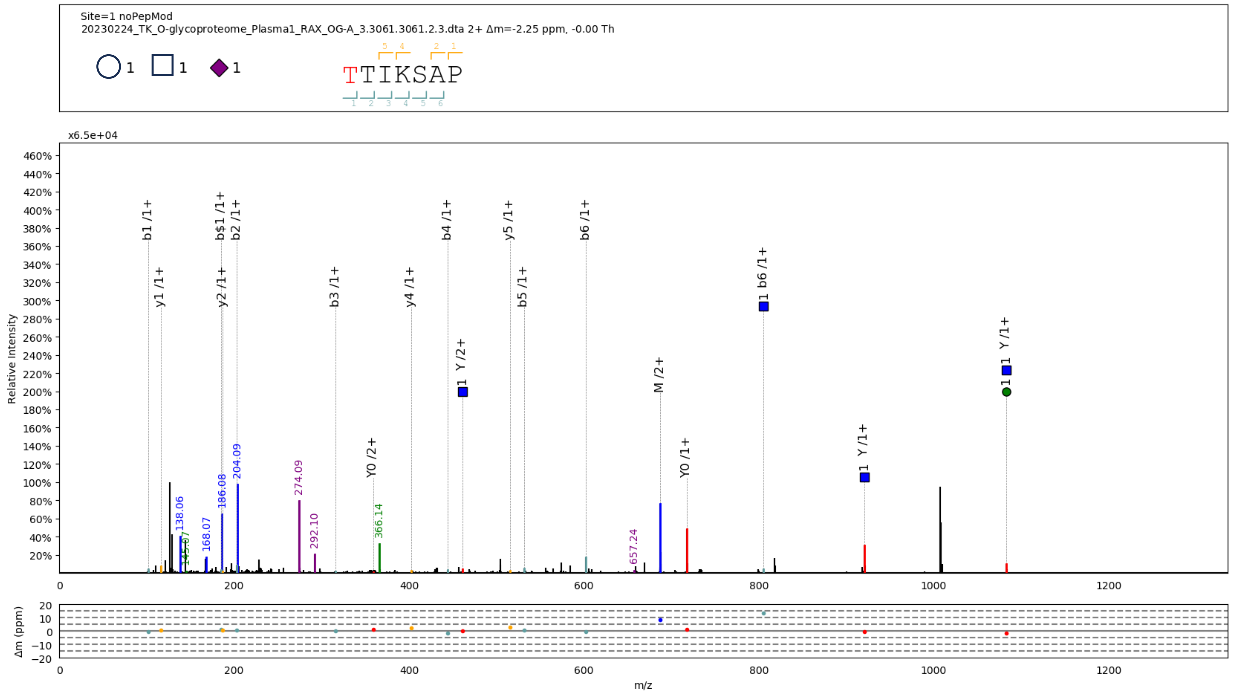

PRG4–S373, H (2) N (2) A (2)

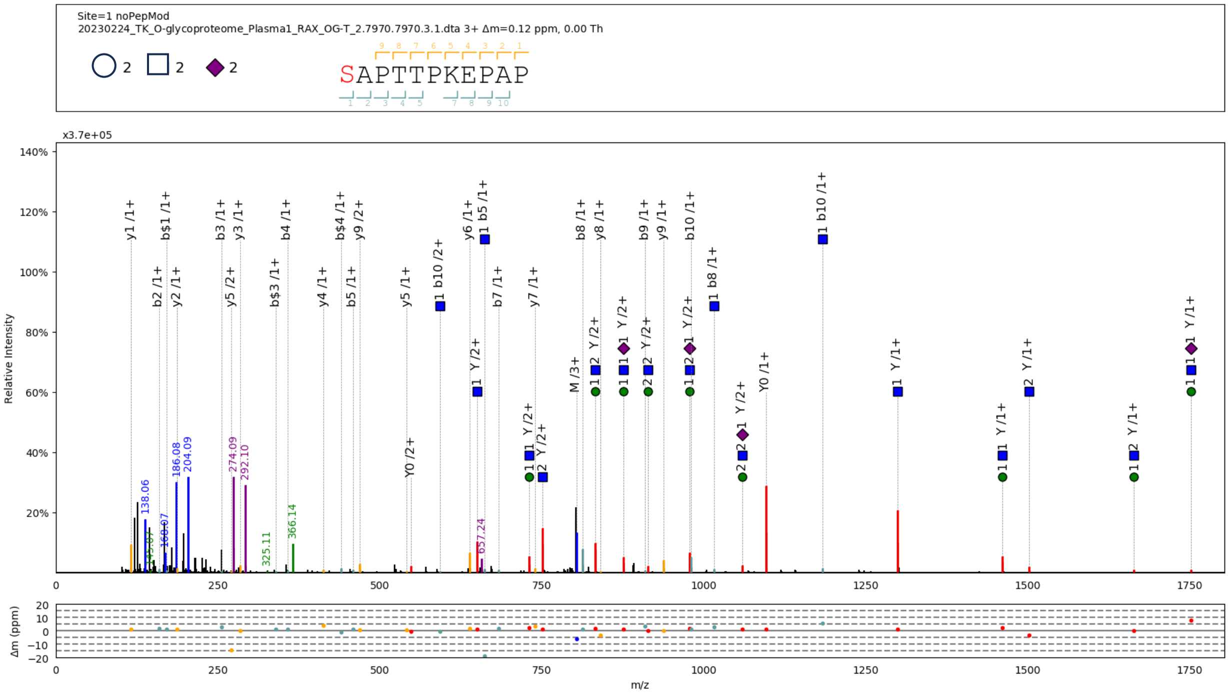

PRG4–S373, H (1) N (1) A (1)

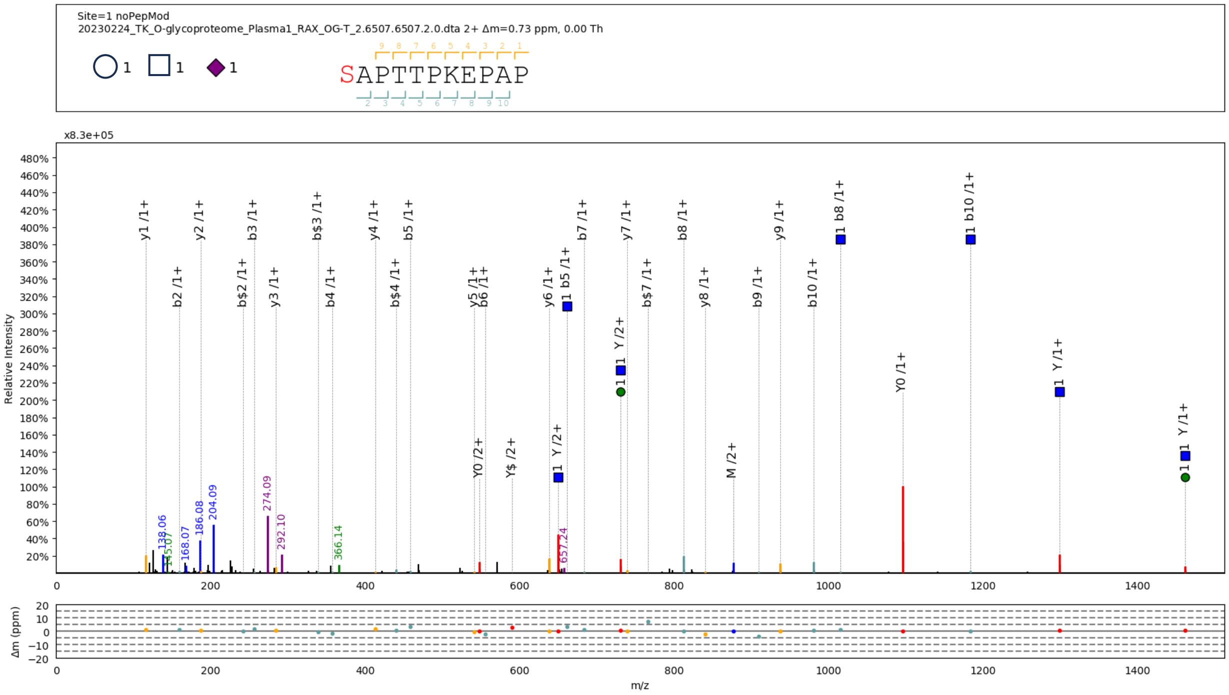

PRG4–S373, H (1) N (1)

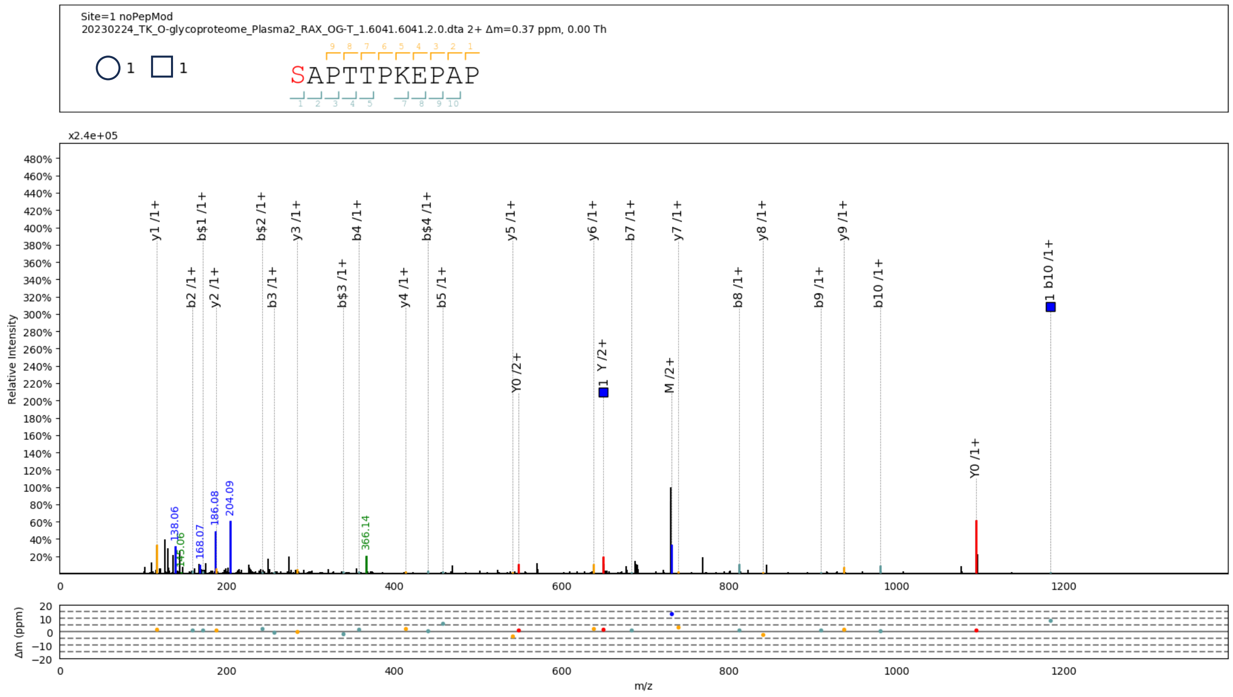

PRG4–T376-T391-T430-T446-T462-T476-T493-T501-T517-T563-T579-T587-T603-T683-T691-T760-T768-T829, H (2) N (2) A (2)

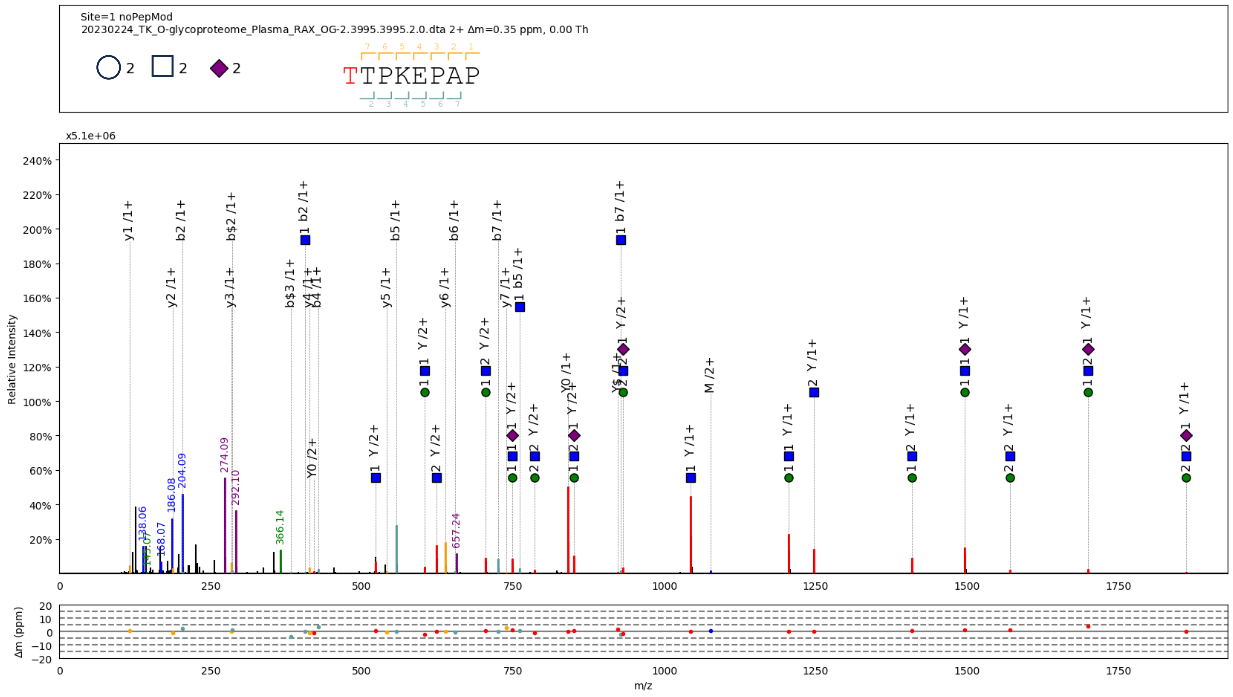

PRG4–T376-T391-T430-T446-T462-T476-T493-T501-T517-T563-T579-T587-T603-T683-T691-T760-T768-T829, H (2) N (2) A (1)

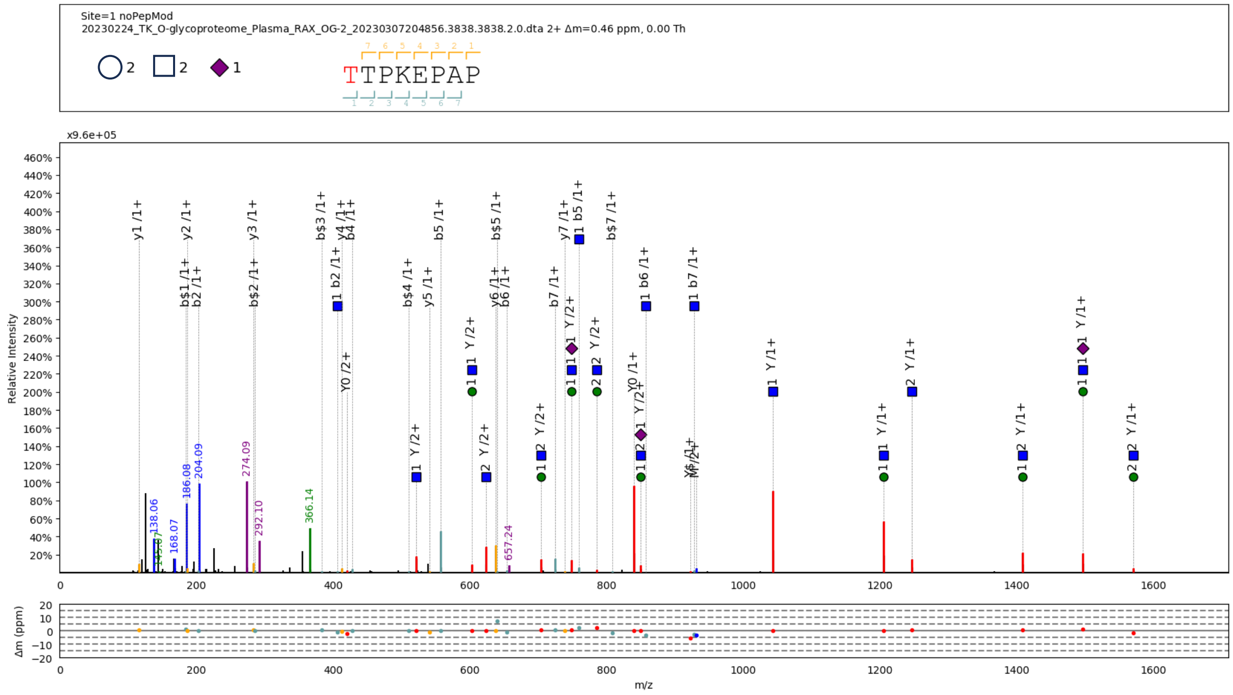

PRG4–T376-T391-T430-T446-T462-T476-T493-T501-T517-T563-T579-T587-T603-T683-T691-T760-T768-T829, H (1) N (2) A (1)

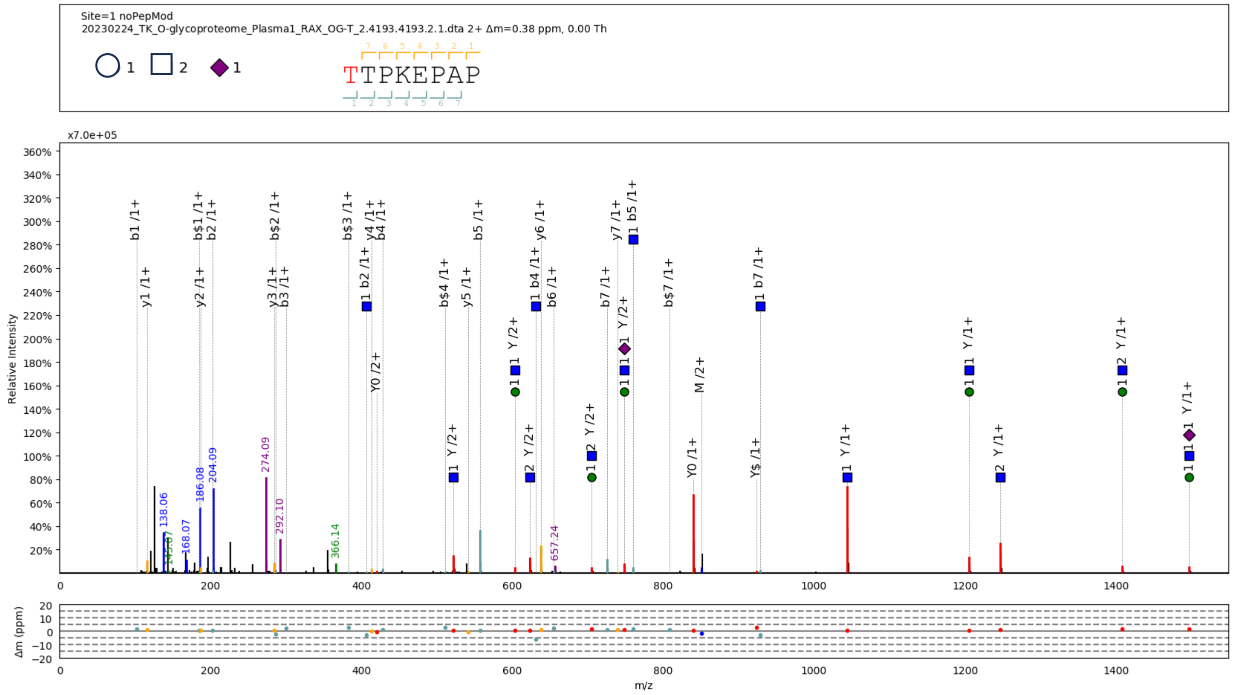

PRG4–T376-T391-T430-T446-T462-T476-T493-T501-T517-T563-T579-T587-T603-T683-T691-T760-T768-T829, H (2) N (2) A (3)

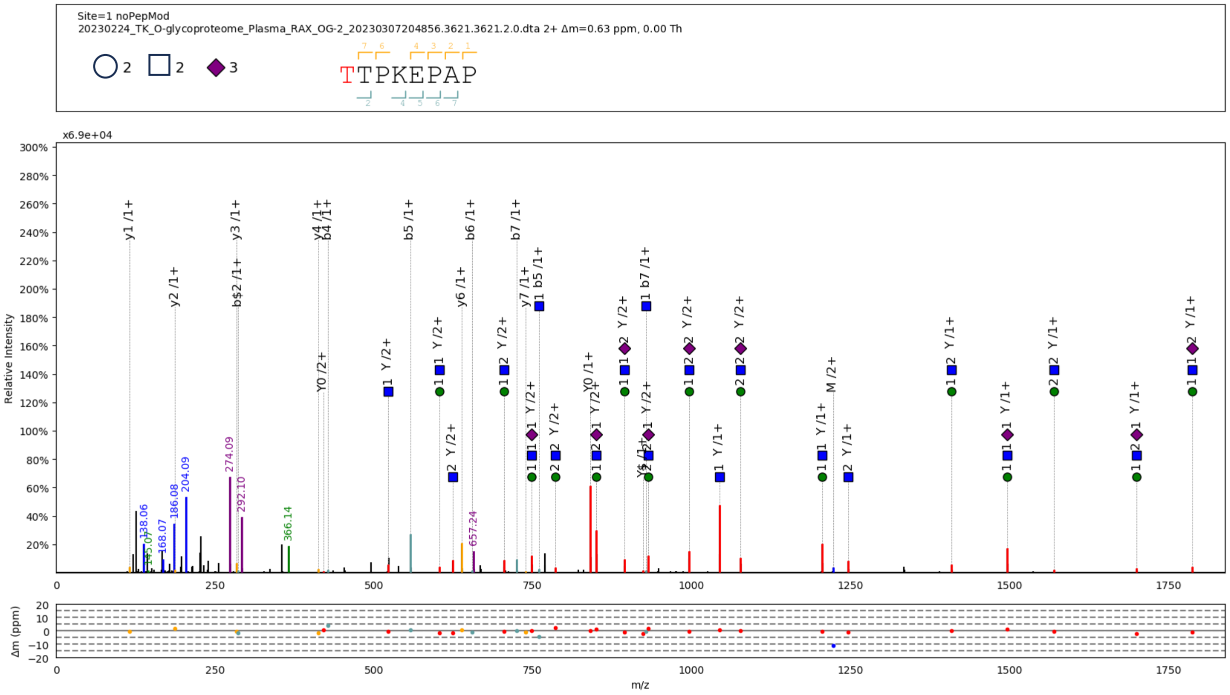

PRG4–T376-T391-T430-T446-T462-T476-T493-T501-T517-T563-T579-T587-T603-T683-T691-T760-T768-T829, H (3) N (2) A (1) F (1)

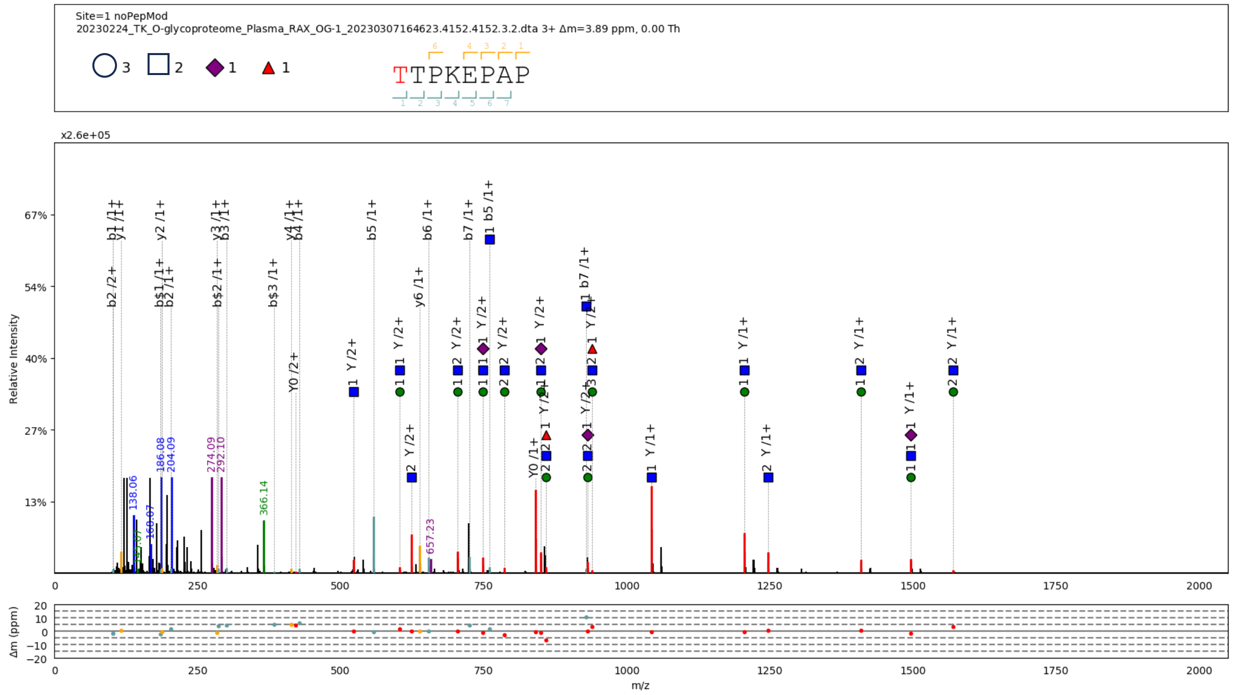

PRG4–T376-T391-T430-T446-T462-T476-T493-T501-T517-T563-T579-T587-T603-T683-T691-T760-T768-T829, H (1) N (1) A (1)

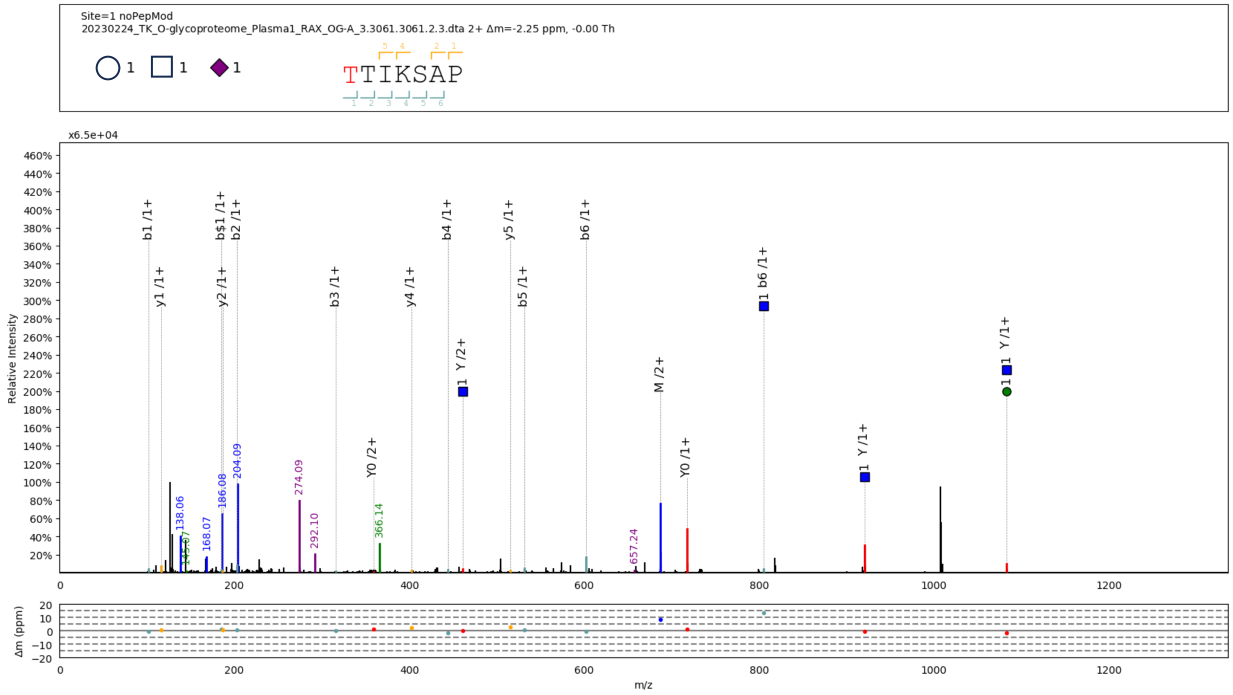

PRG4–T376-T391-T430-T446-T462-T476-T493-T501-T517-T563-T579-T587-T603-T683-T691-T760-T768-T829, H (1) N (2)

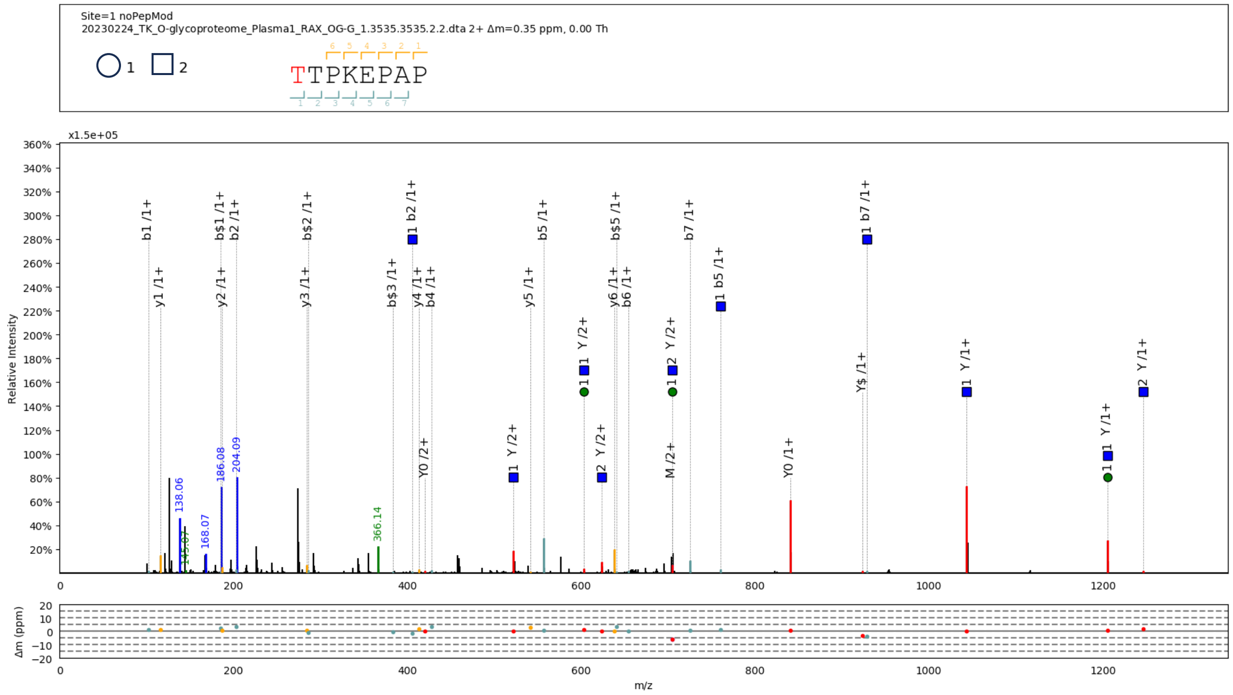

PRG4─T376-T391-T430-T446-T462-T476-T493-T501-T517-T563-T579-T587-T603-T683-T691-T760-T768-T829, H (2) N (2)

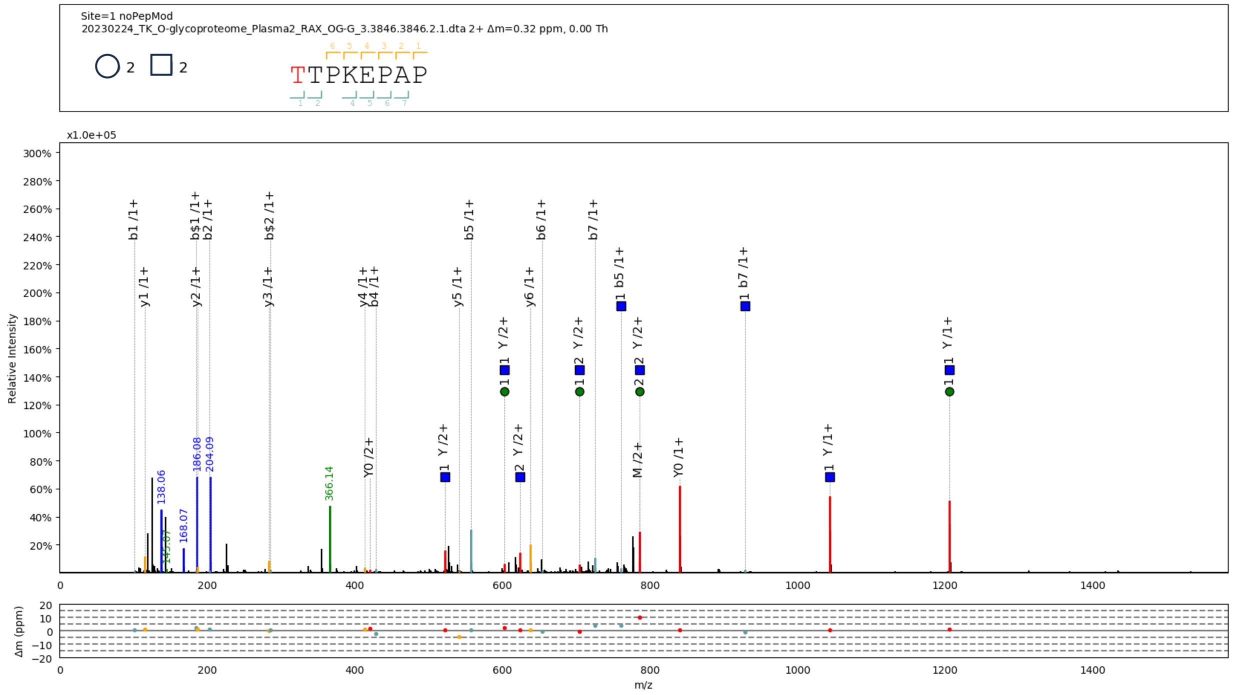

PRG4─T376-T391-T430-T446-T462-T476-T493-T501-T517-T563-T579-T587-T603-T683-T691-T760-T768-T829, H (1) N (2) A (1) F (1)

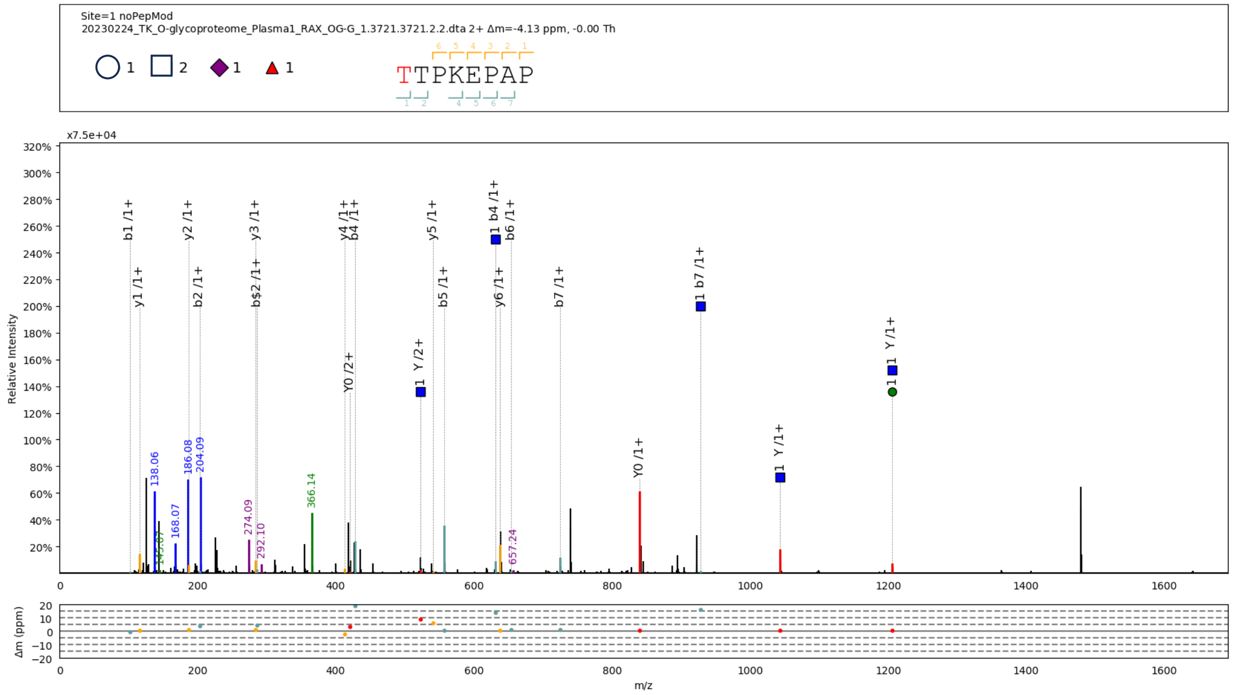

PRG4–T376-T391-T430-T446-T462-T476-T493-T501-T517-T563-T579-T587-T603-T683-T691-T760-T768-T829, H (1) N (2) A (1) F (1)

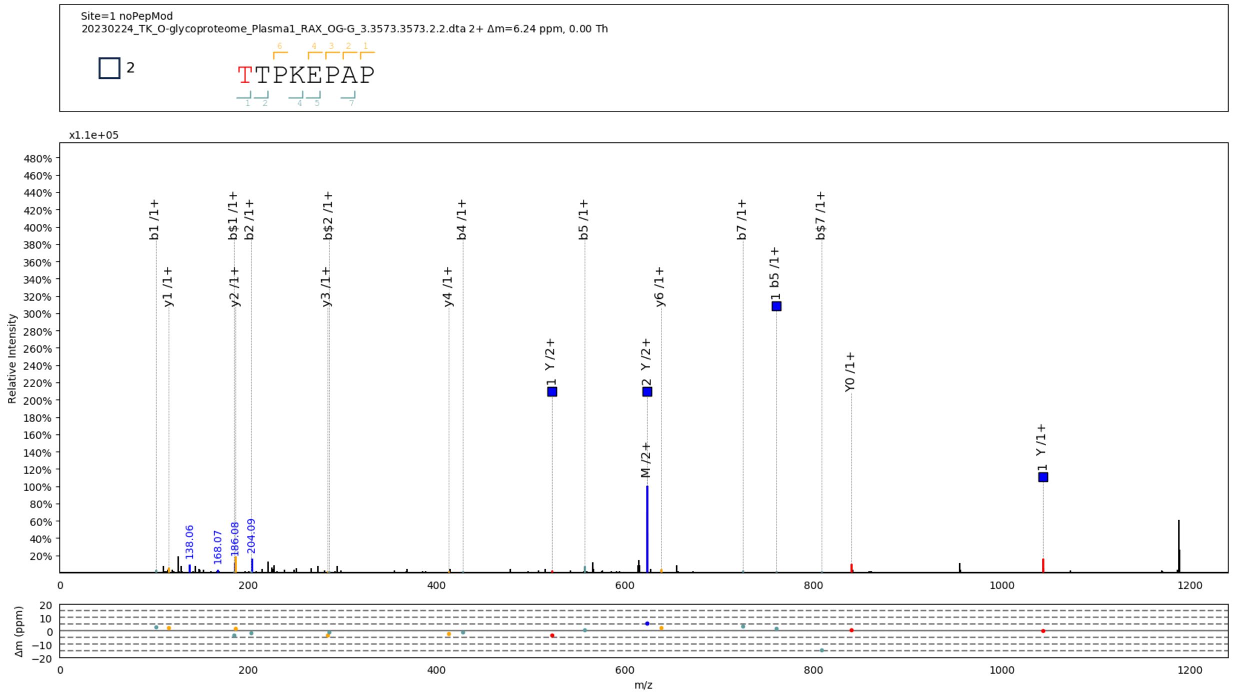

PRG4–T399-T415-T532-T555, H (3) N (3) A (3)

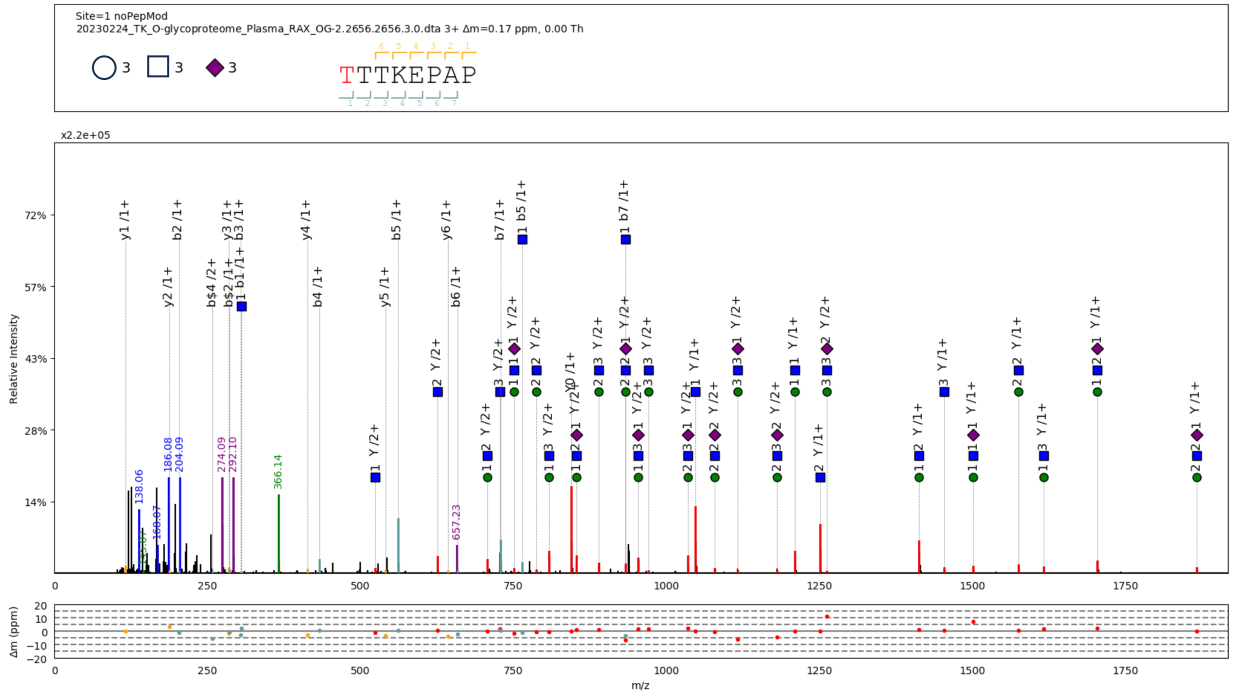

PRG4–T399-T415-T532-T555, H (2) N (2) A (2)

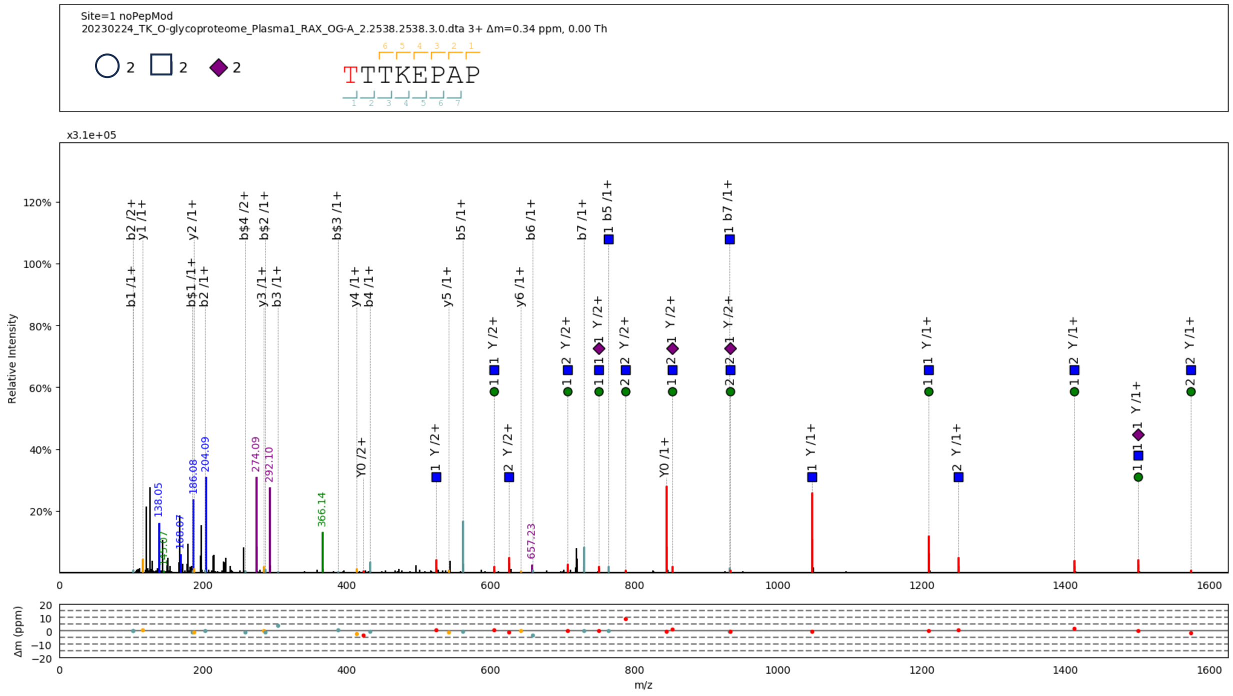

PRG4–T399-T415-T532-T555, H (3) N (3) A (2)

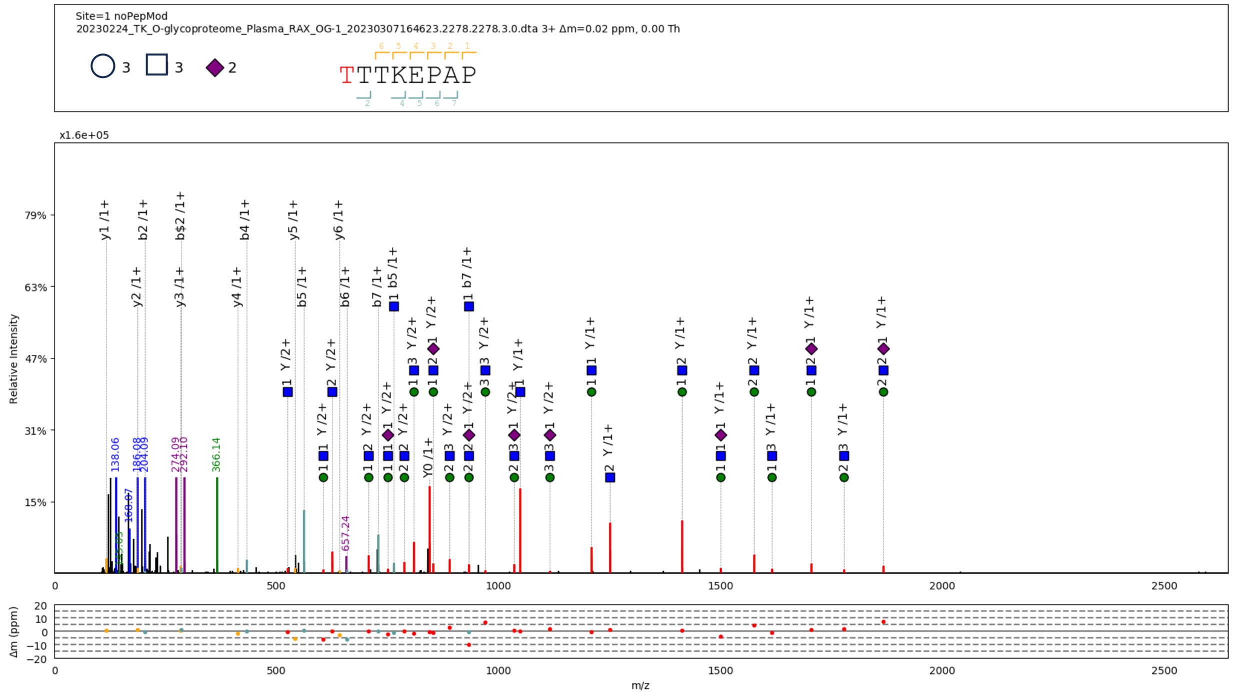

PRG4–T399-T415-T532-T555, H (2) N (3) A (2)

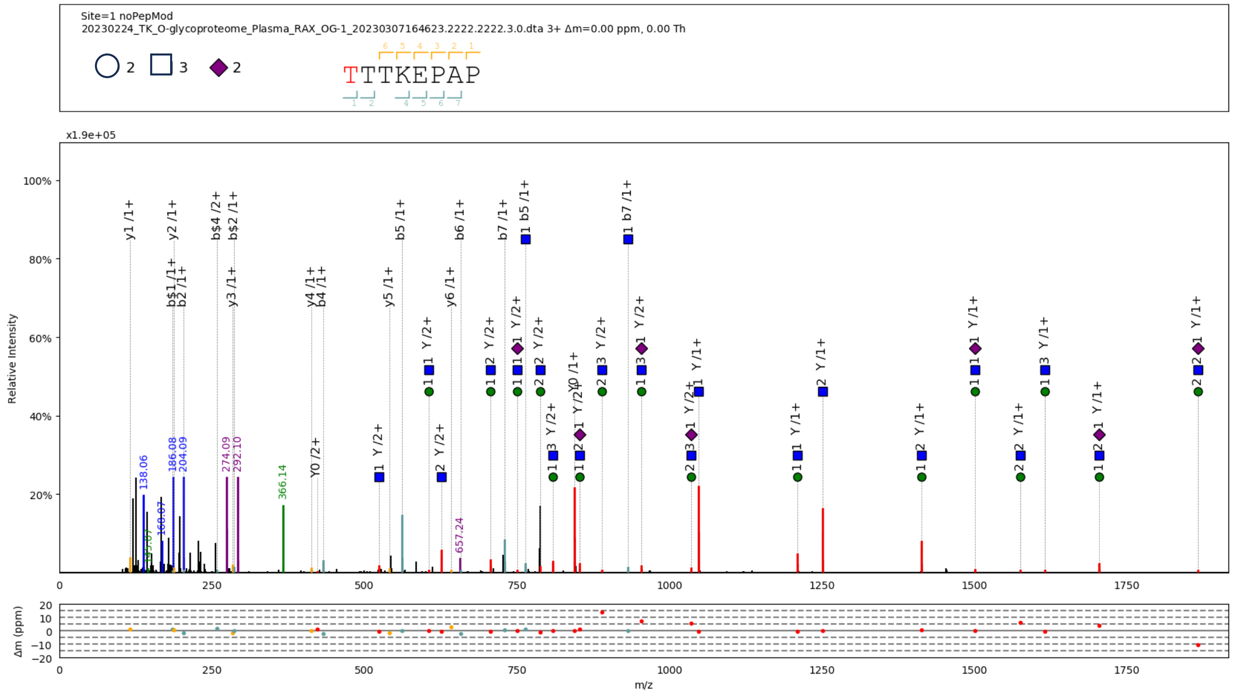

PRG4–T399-T415-T532-T555, H (2) N (2) A (1)

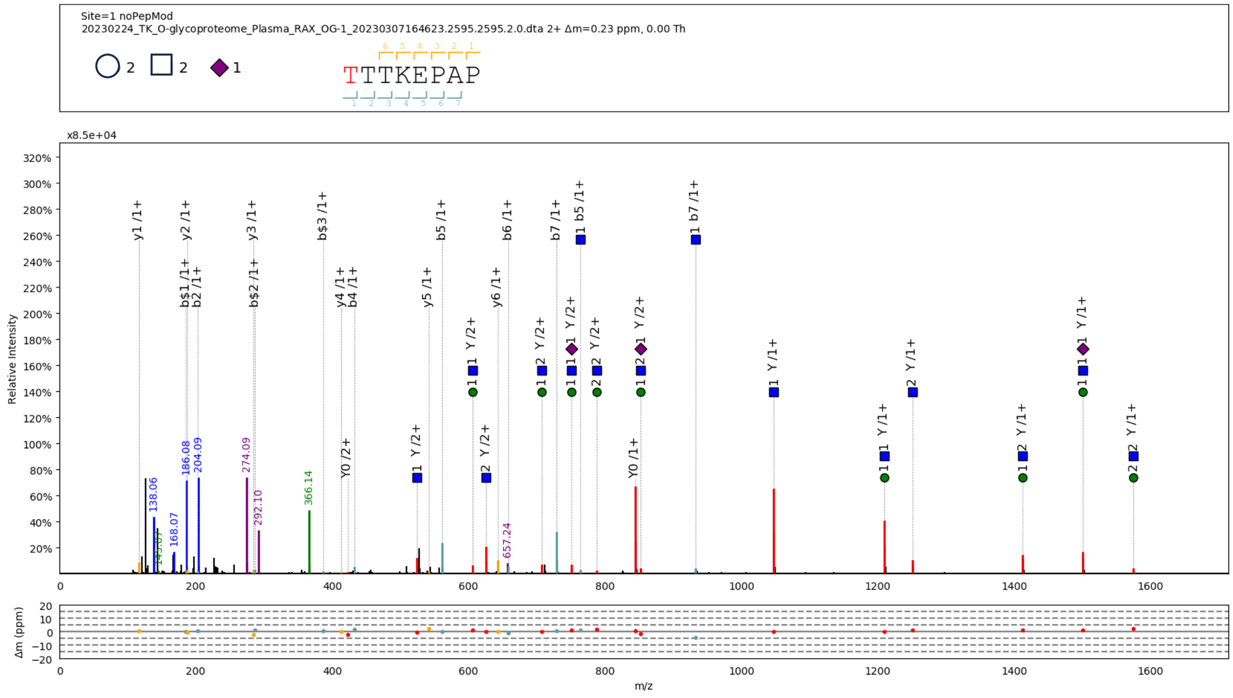

PRG4–T399-T415-T532-T555, H (2) N (2) F (2)

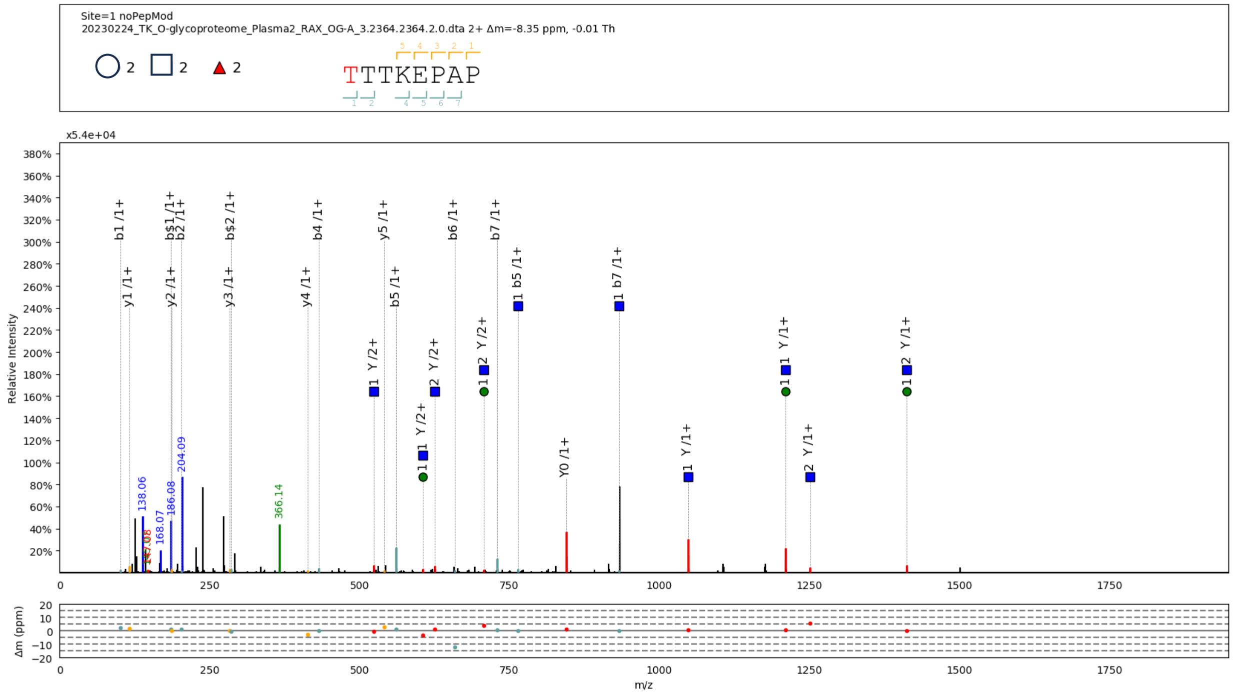

PRG4–T400-T416-T533-T556, H (2) N (2) A (2)

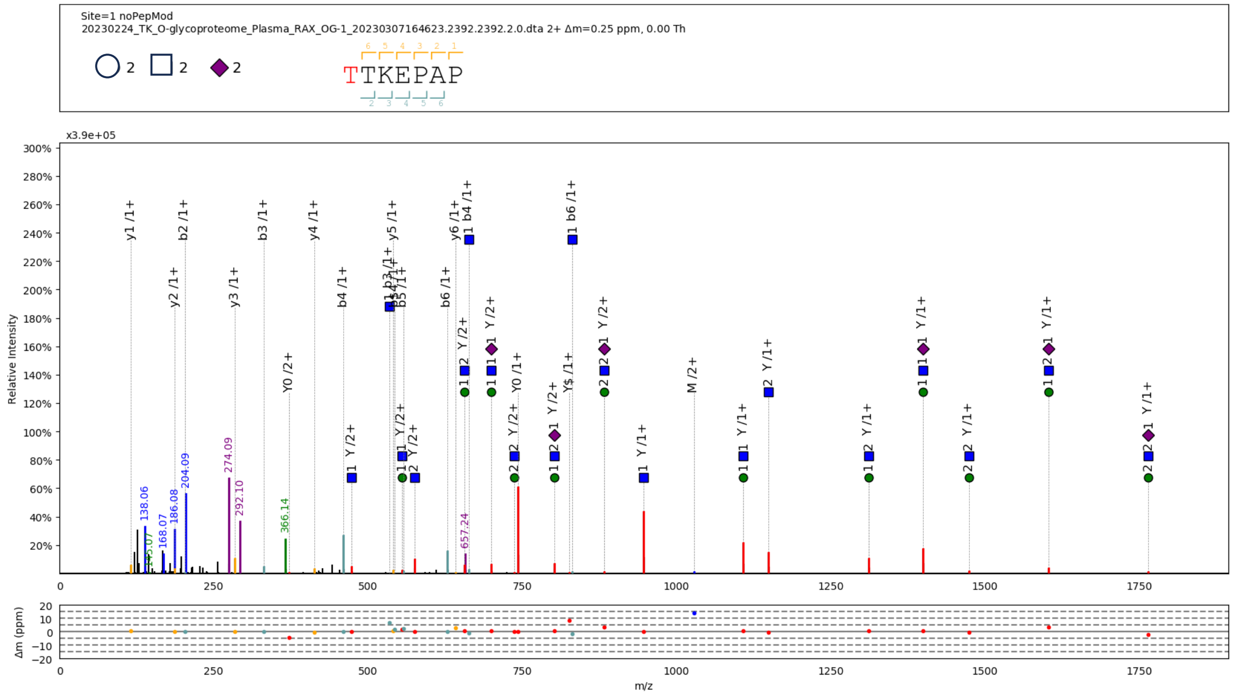

PRG4−T400-T416-T533-T556, H (2) N (2) A (1)

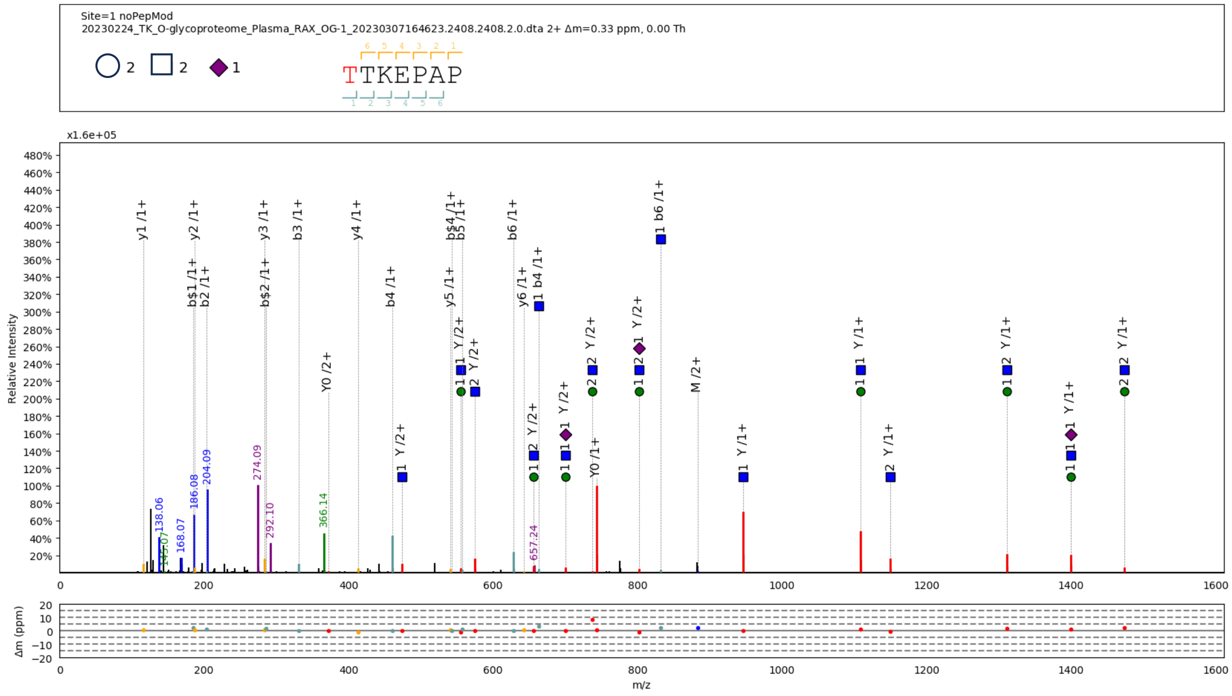

PRG4−T400-T416-T533-T556, H (1) N (2) A (1)

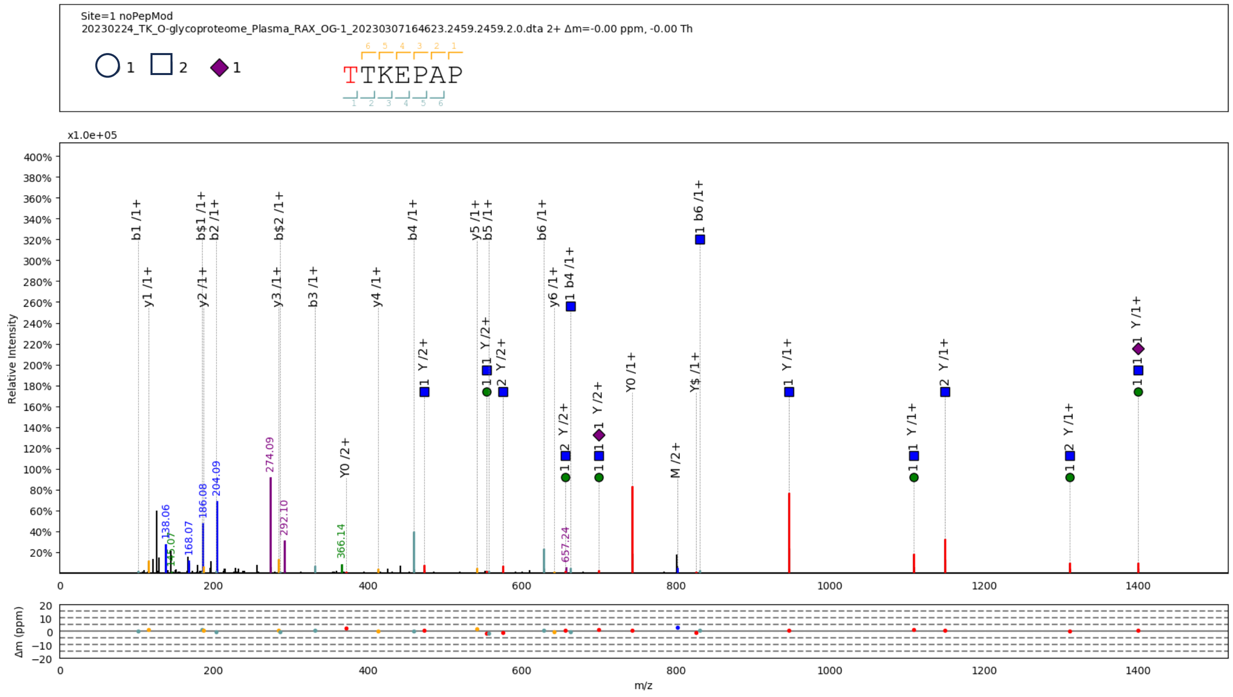

PRG4–T438-T571-T723-T792-T837, H (2) N (2) A (2)

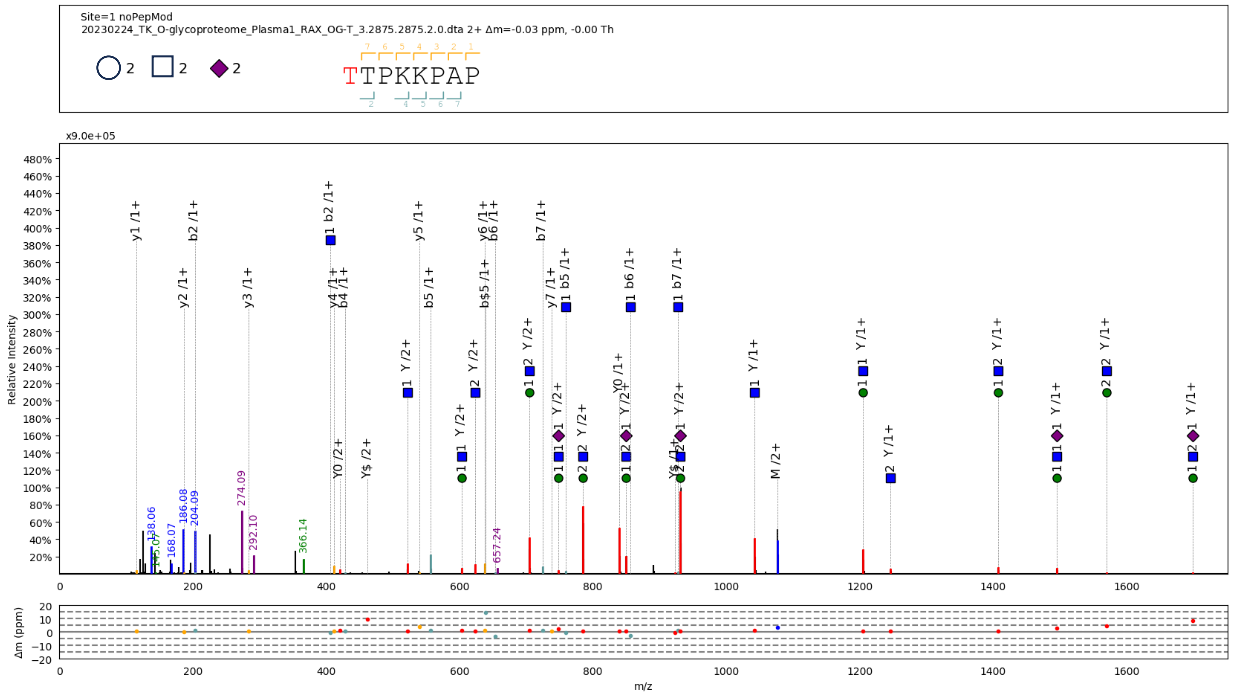

PRG4–T438-T571-T723-T792-T837, H (1) N (2) A (1)

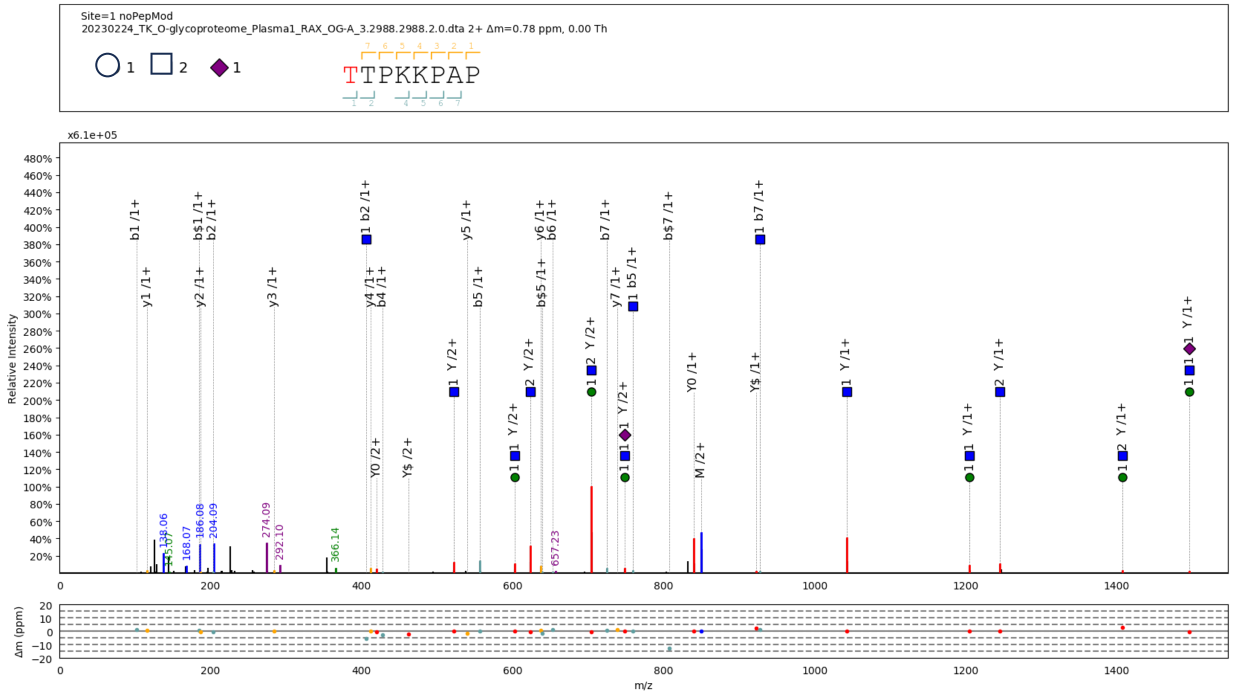

PRG4–T438-T571-T723-T792-T837, H (2) N (2) A (1)

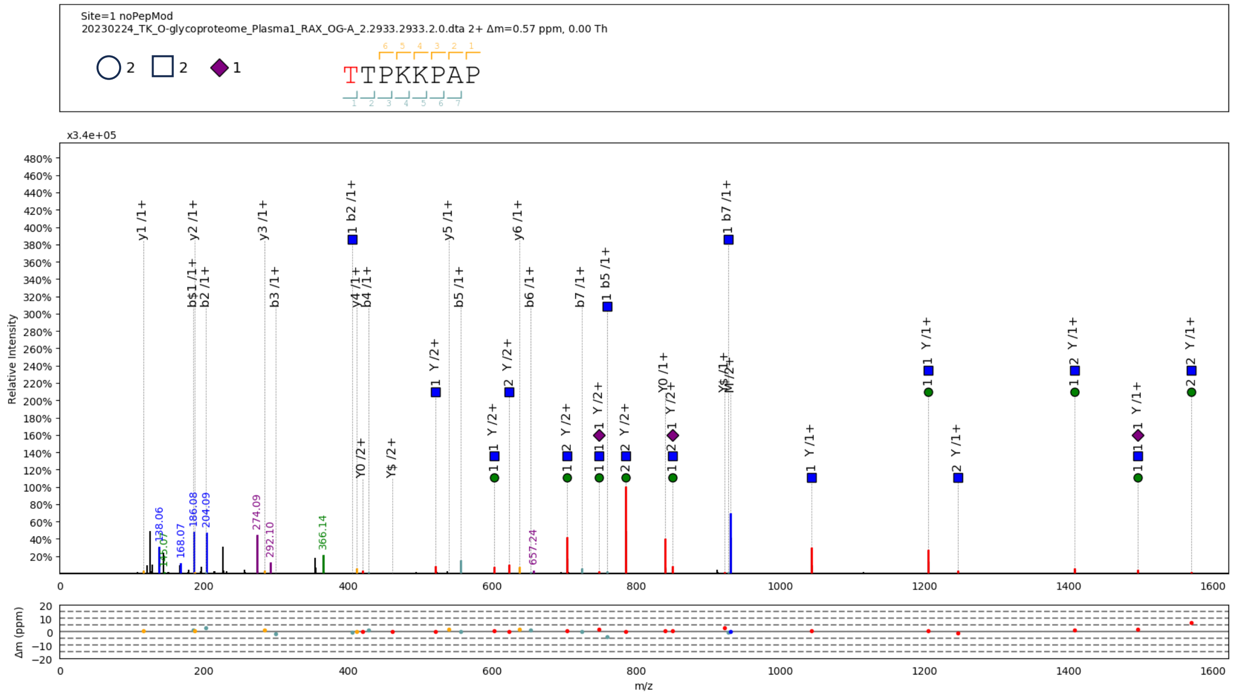

PRG4–T438-T571-T723-T792-T837, H (2) N (2)

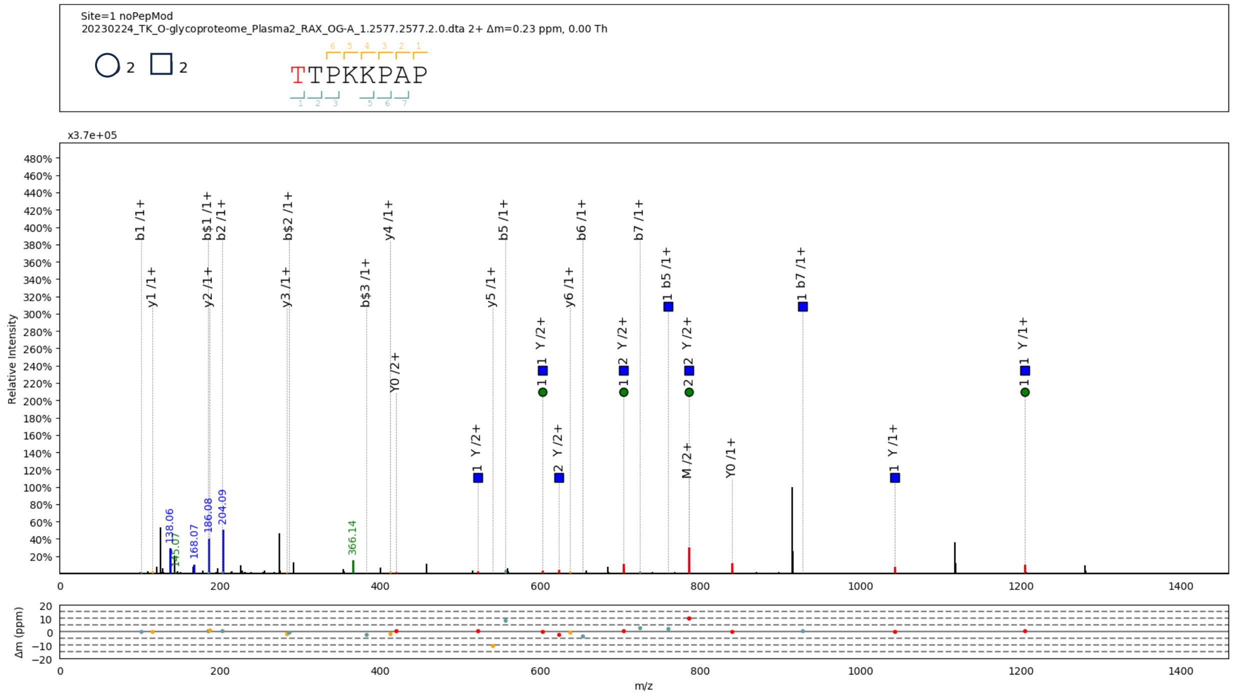

PRG4–T438-T571-T723-T792-T837, H (1) N (2)

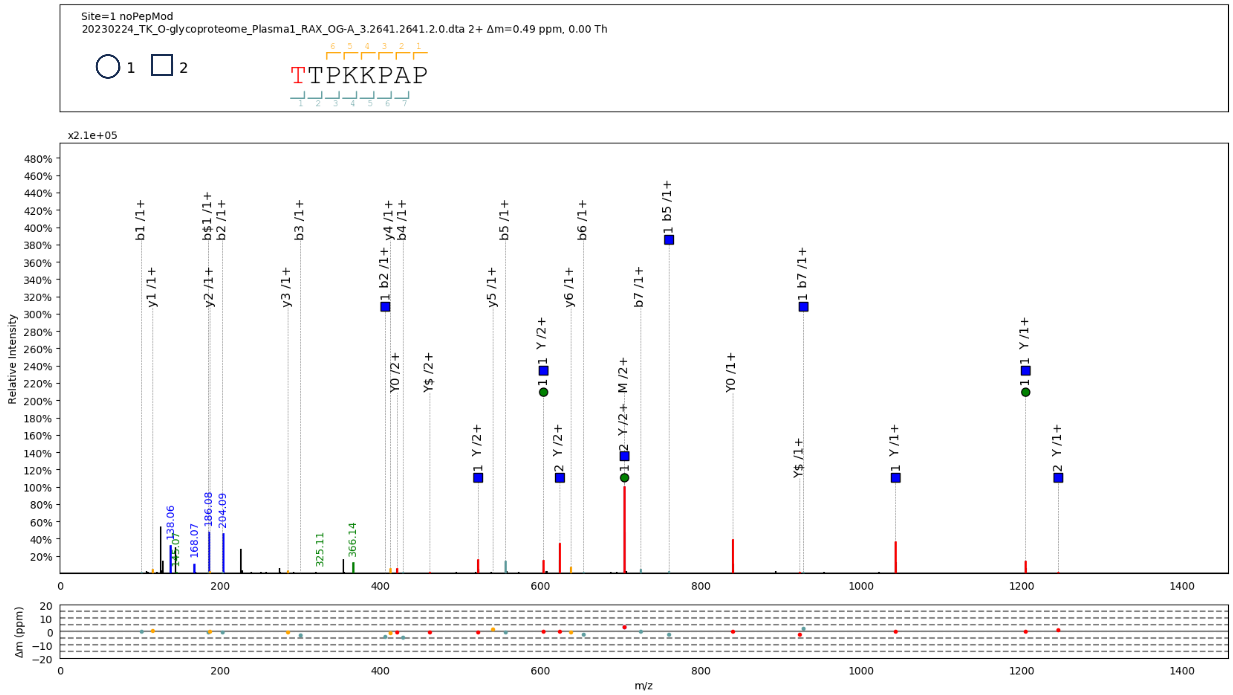

PRG4–T438-T571-T723-T792-T837, H (1) N (1)

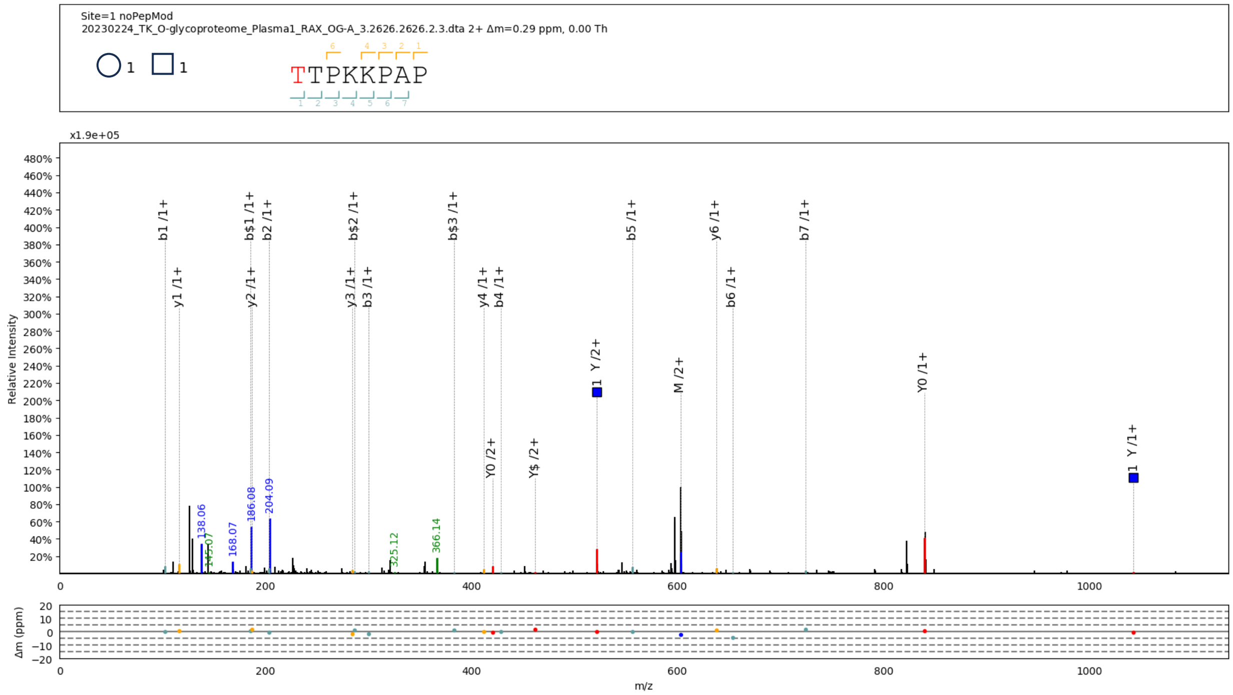

PRG4−T460, H (2) N (3) A (2)

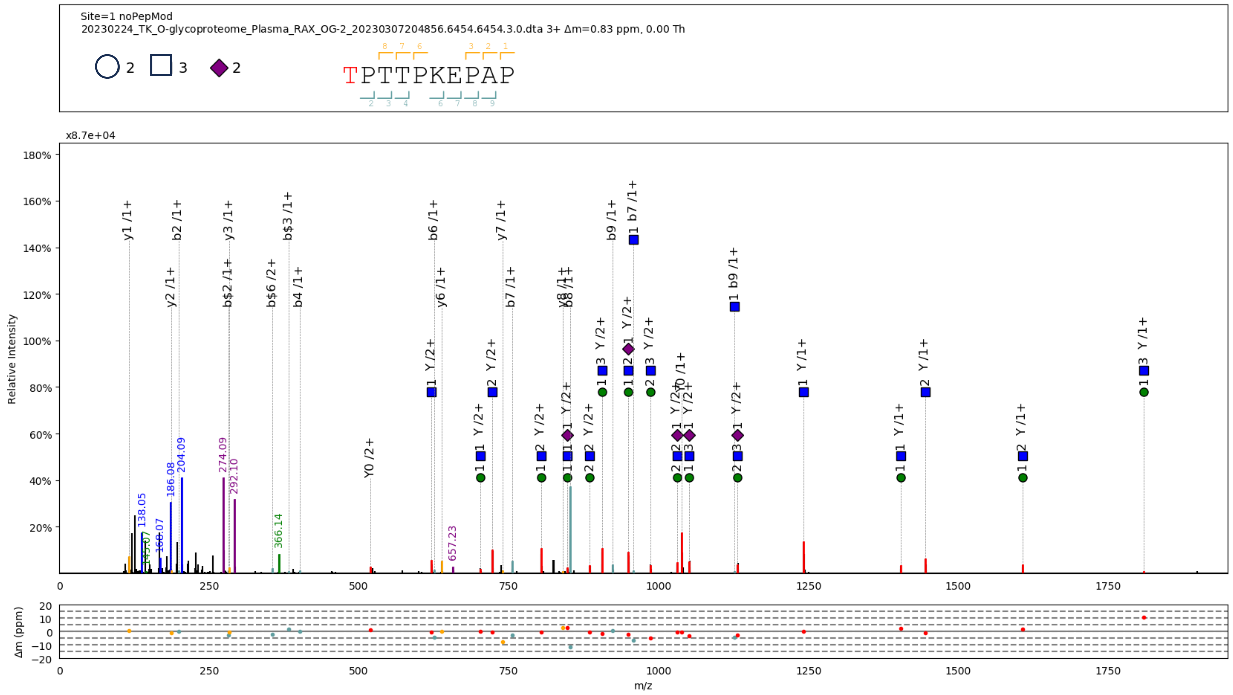

PRG4−T460, H (2) N (2) A (2)

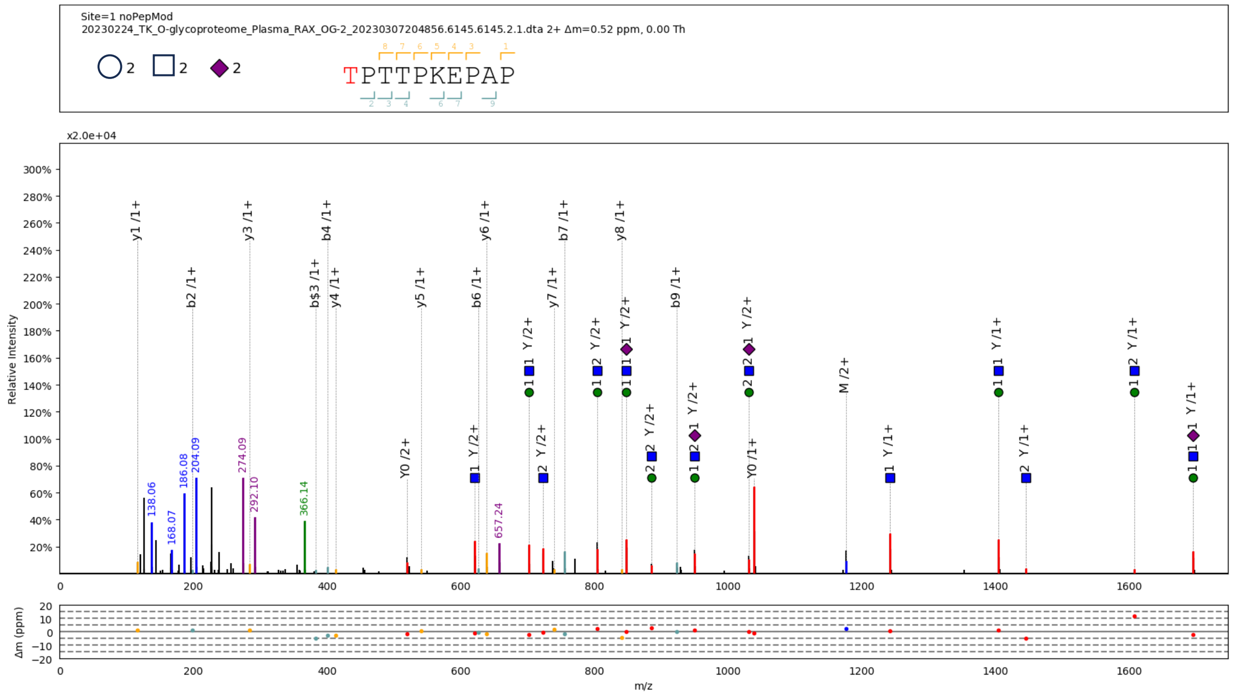

PRG4–T485, H (3) N (3) A (3)

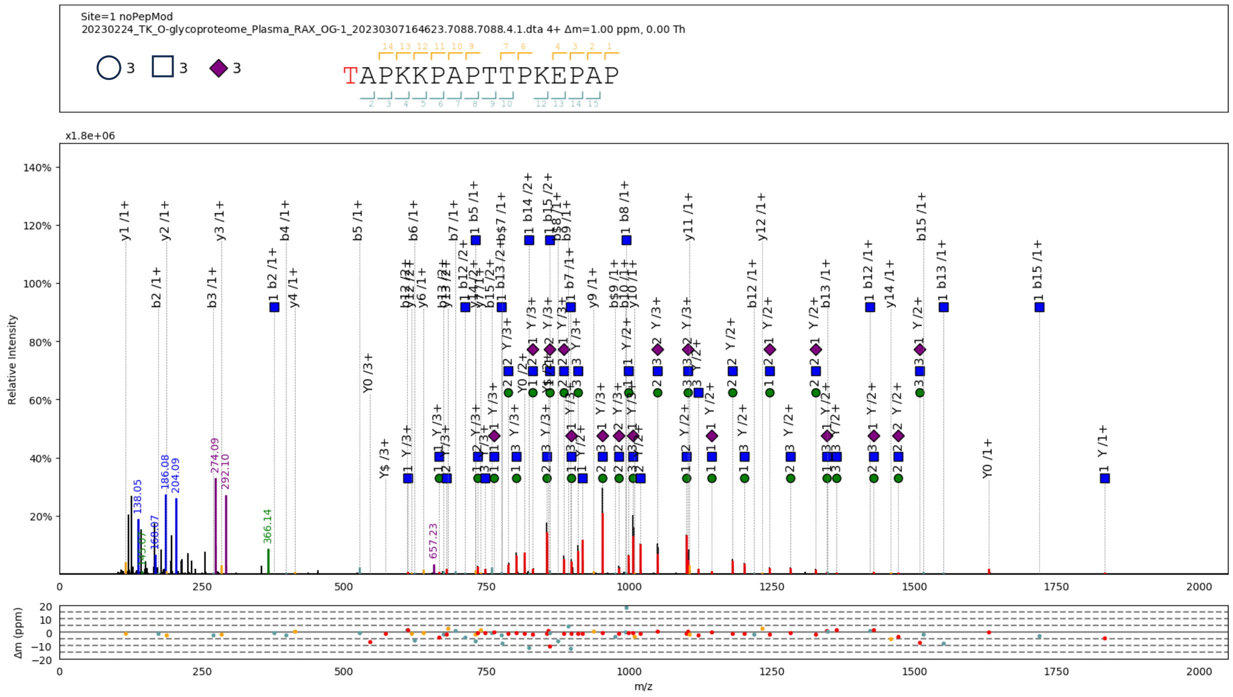

PRG4–T485, H (2) N (3) A (2)

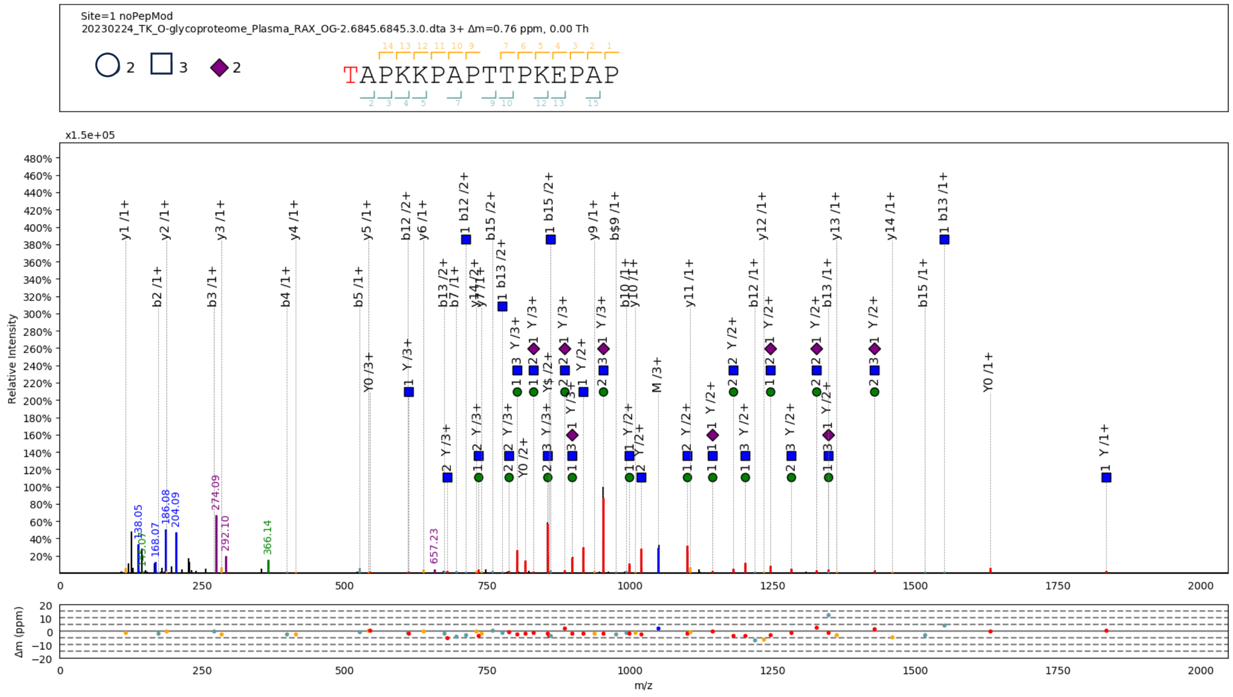

PRG4–T485, H (3) N (3) A (2)

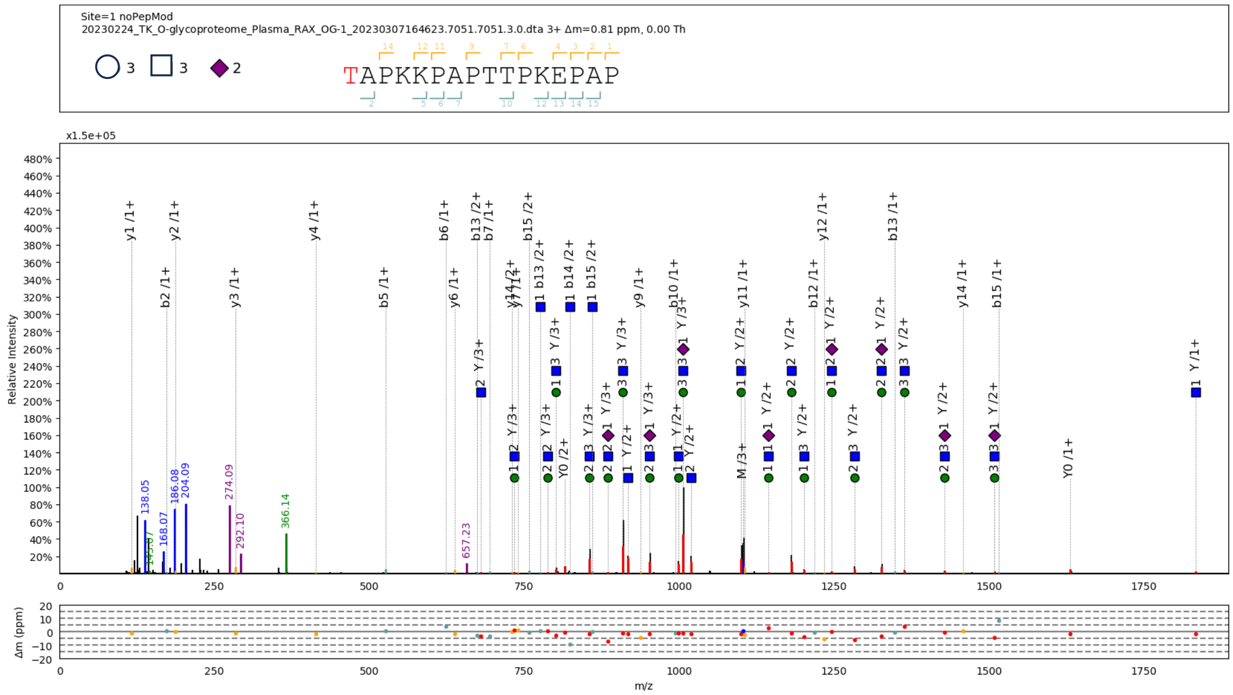

PRG4–T485, H (2) N (2) A (2)

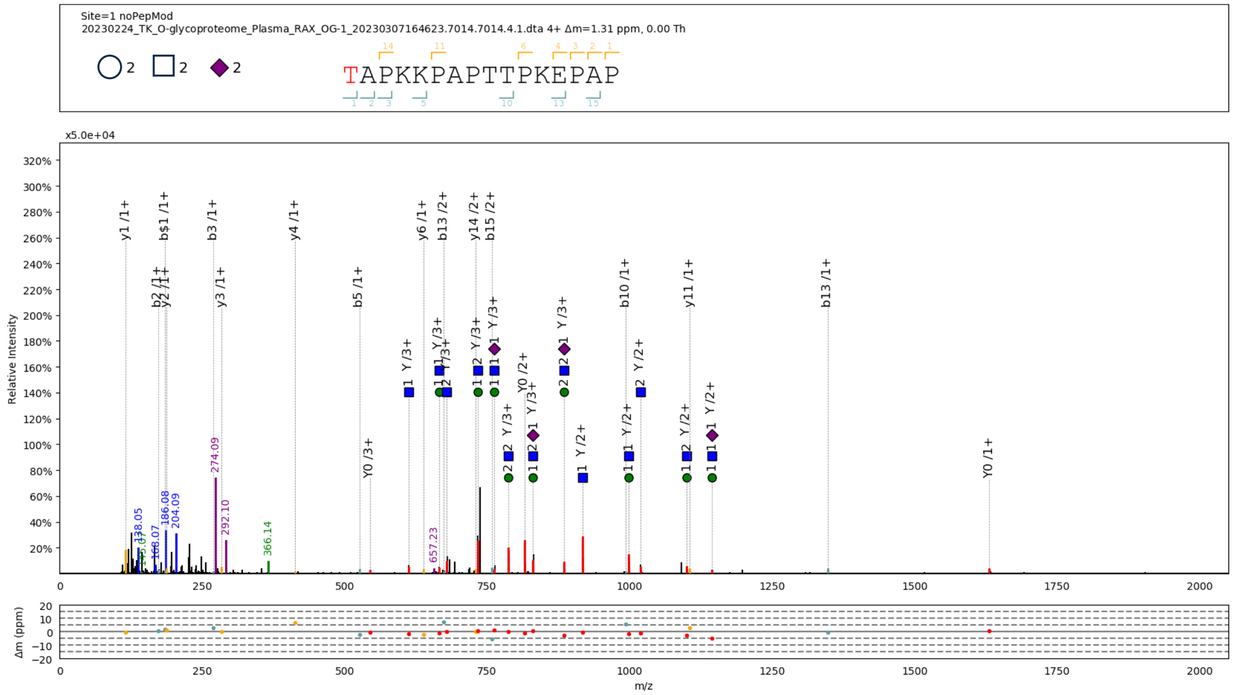

PRG4−T485, H (1) N (1) A (1)

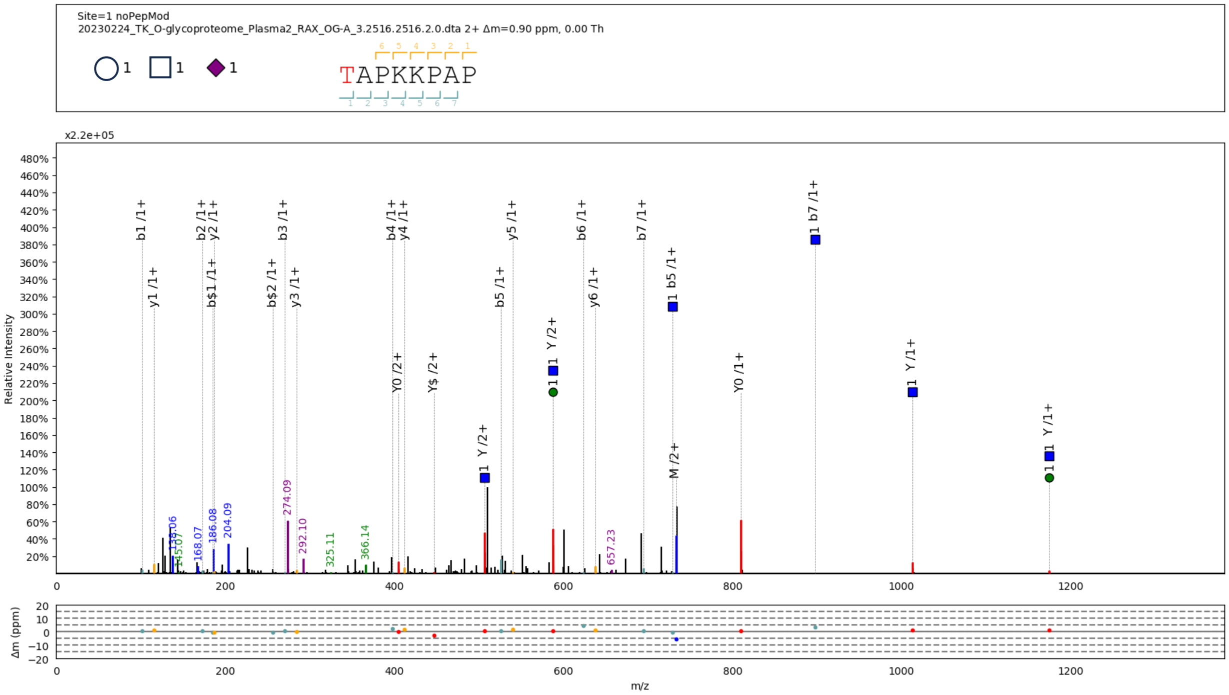

PRG4−T485, H (1) N (1)

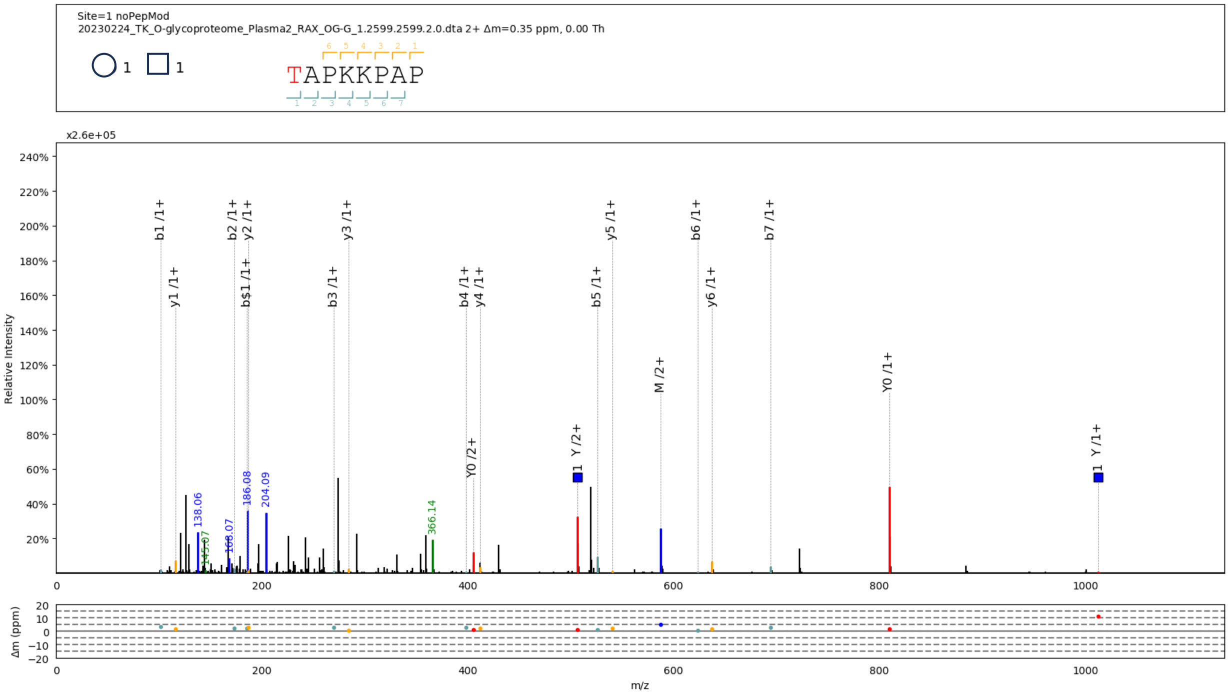

PRG4–S544, H (1) N (1) A (1)

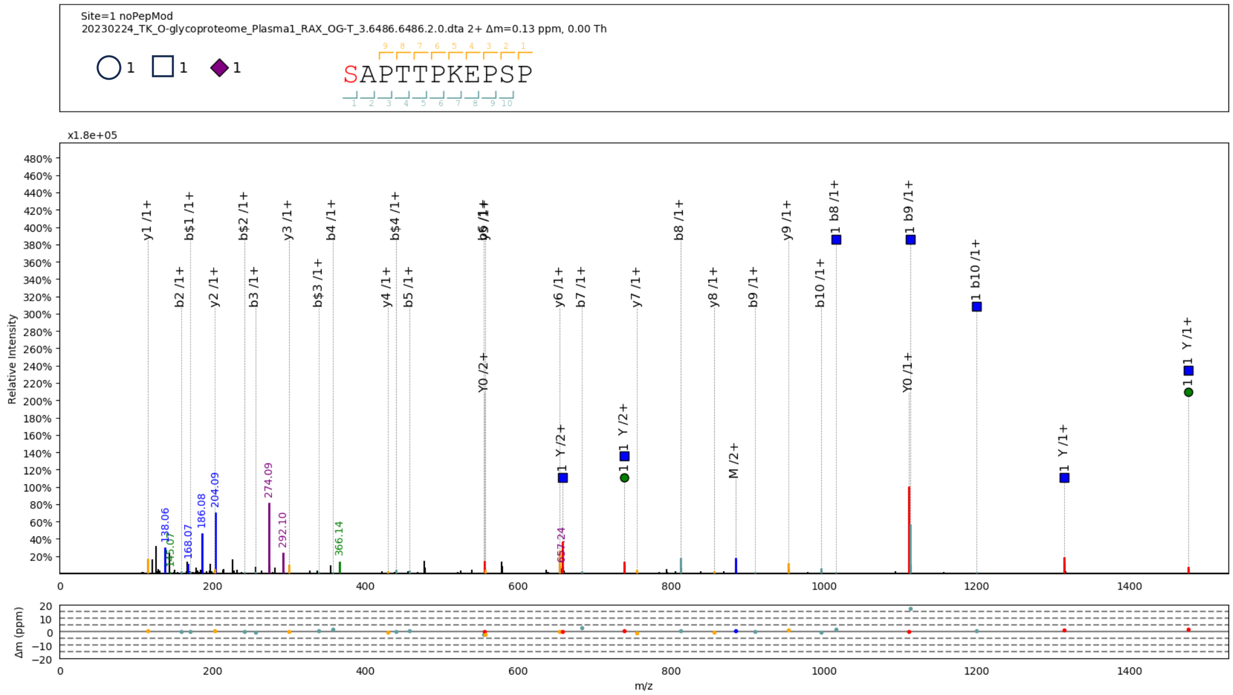

PRG4–T595, H (2) N (2) A (2)

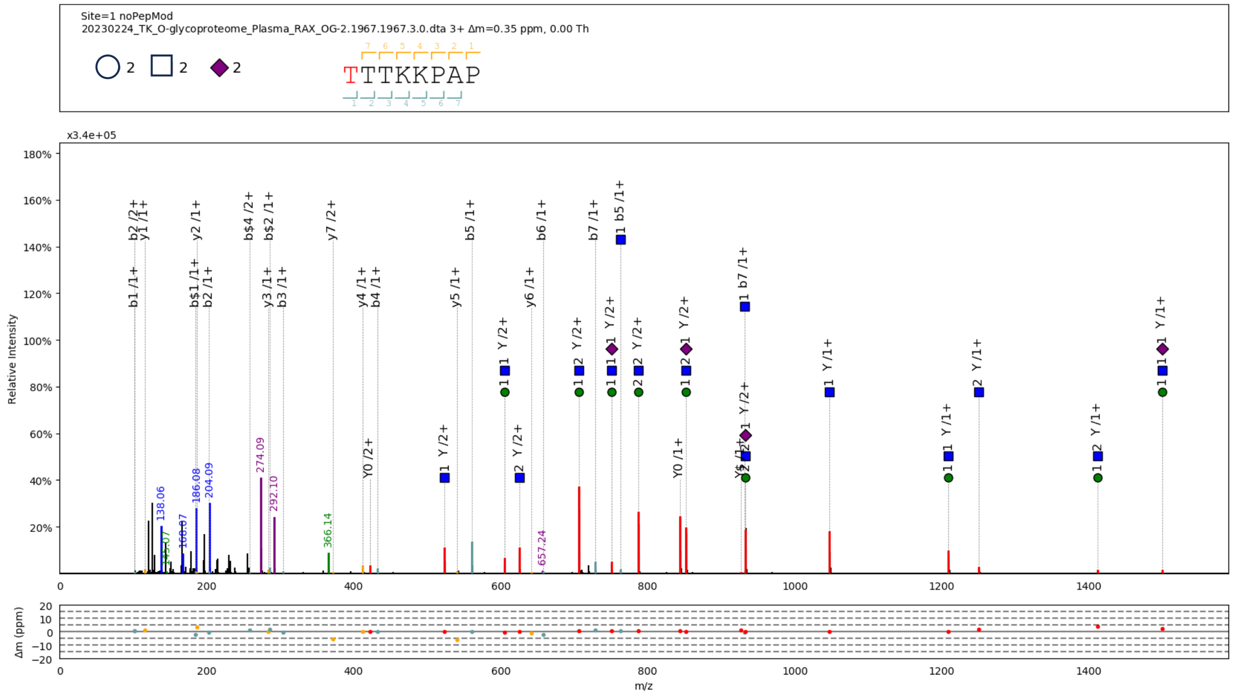

PRG4–T595, H (2) N (3) A (2)

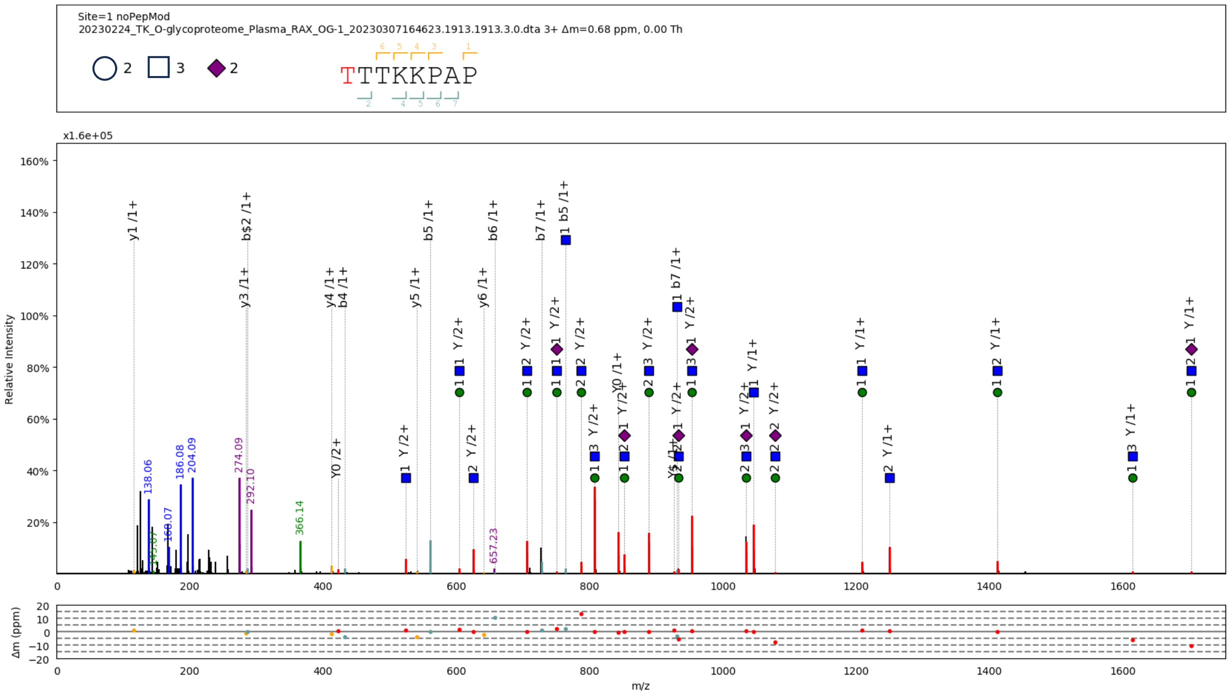

PRG4–T595, H (3) N (3) A (2)

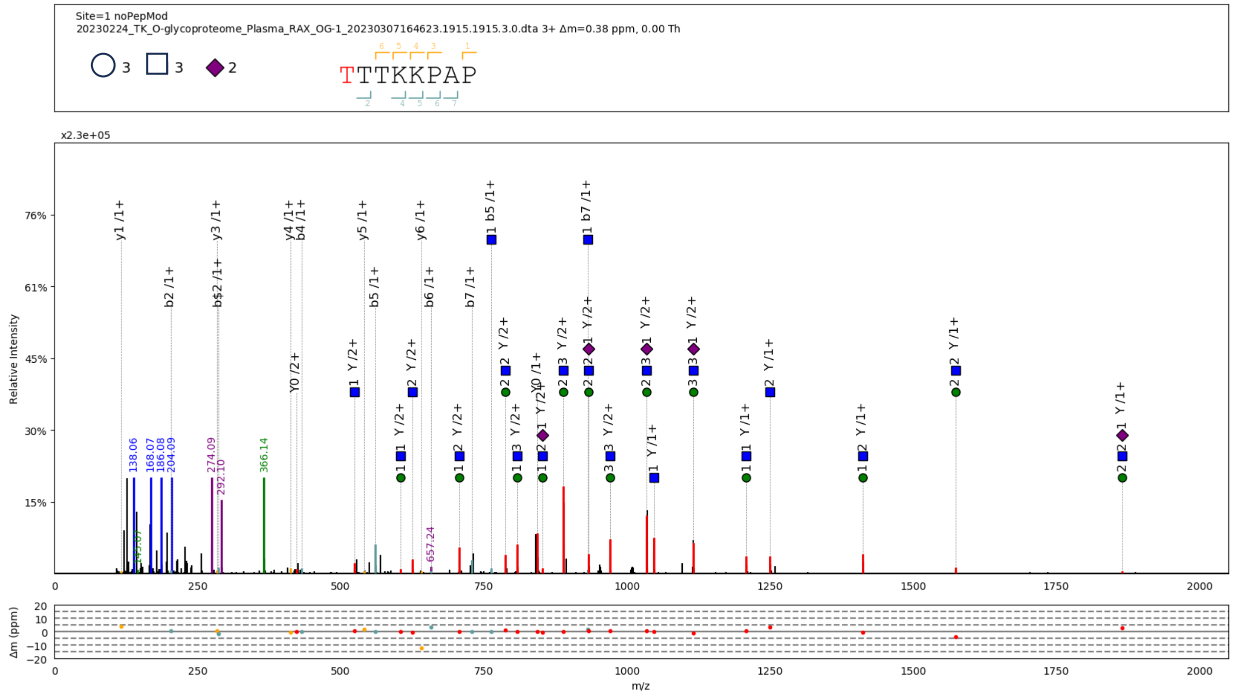

PRG4–T595, H (2) N (3) A (1)

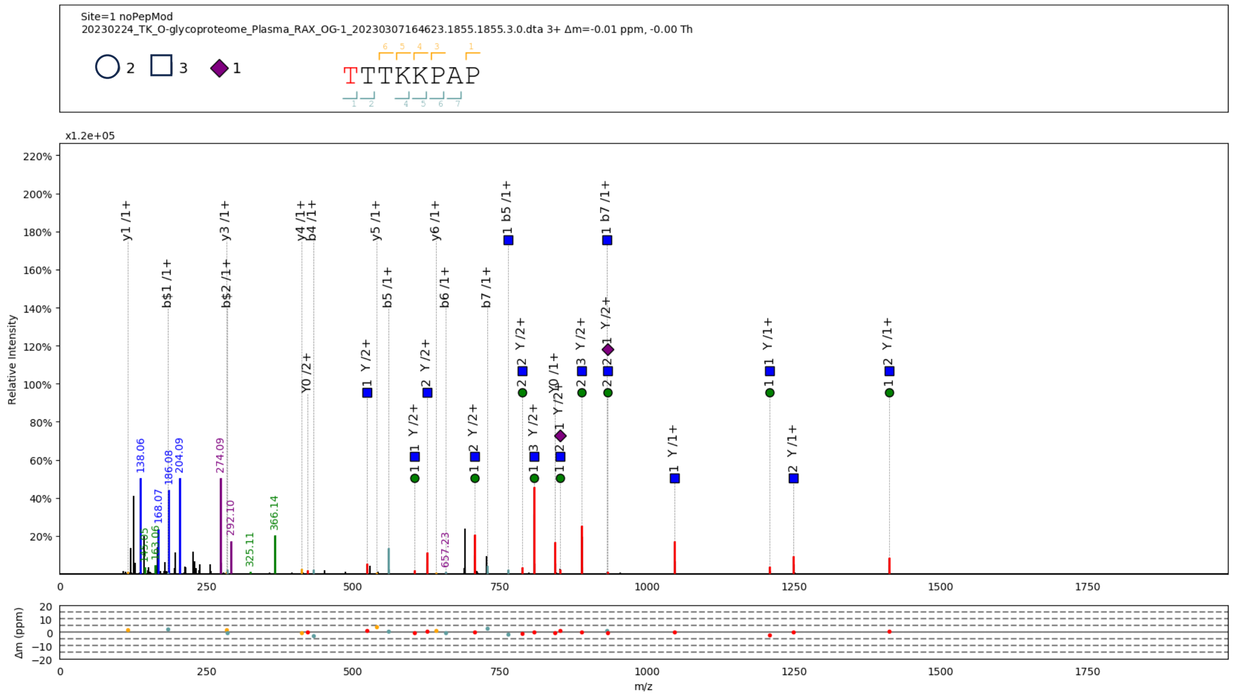

PRG4–T611-T699-T821, H (2) N (2) A (2)

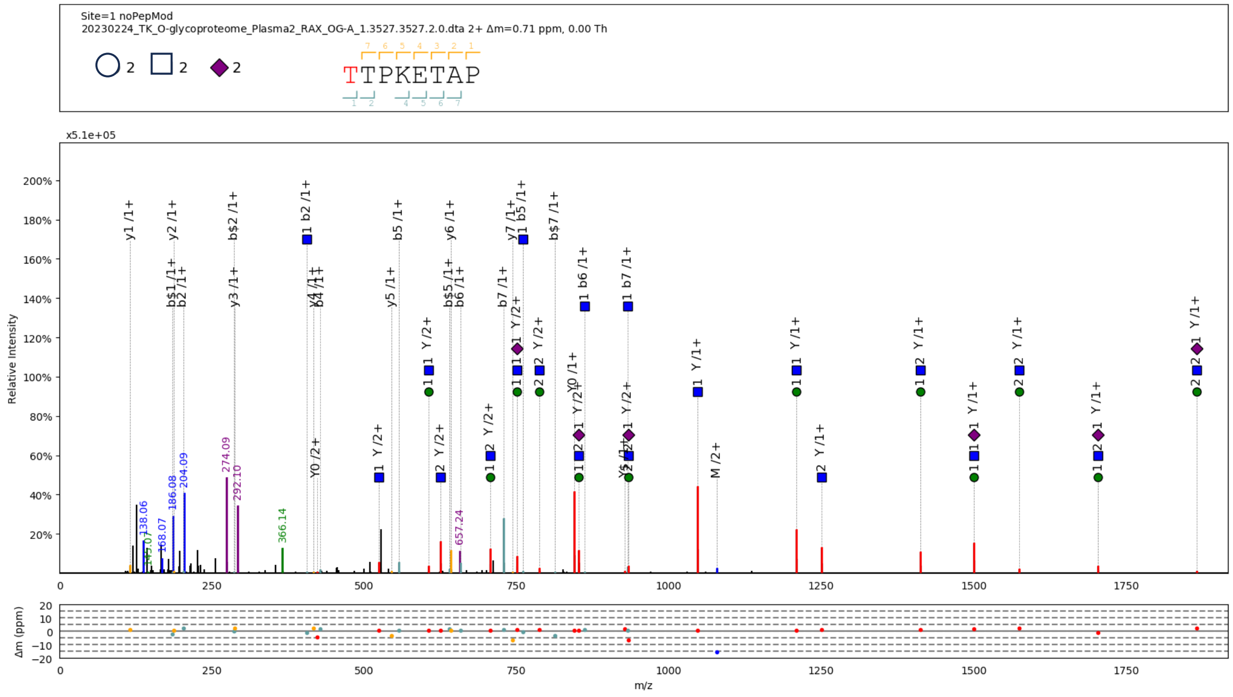

PRG4‒T611-T699-T821, H (2) N (2) A (1)

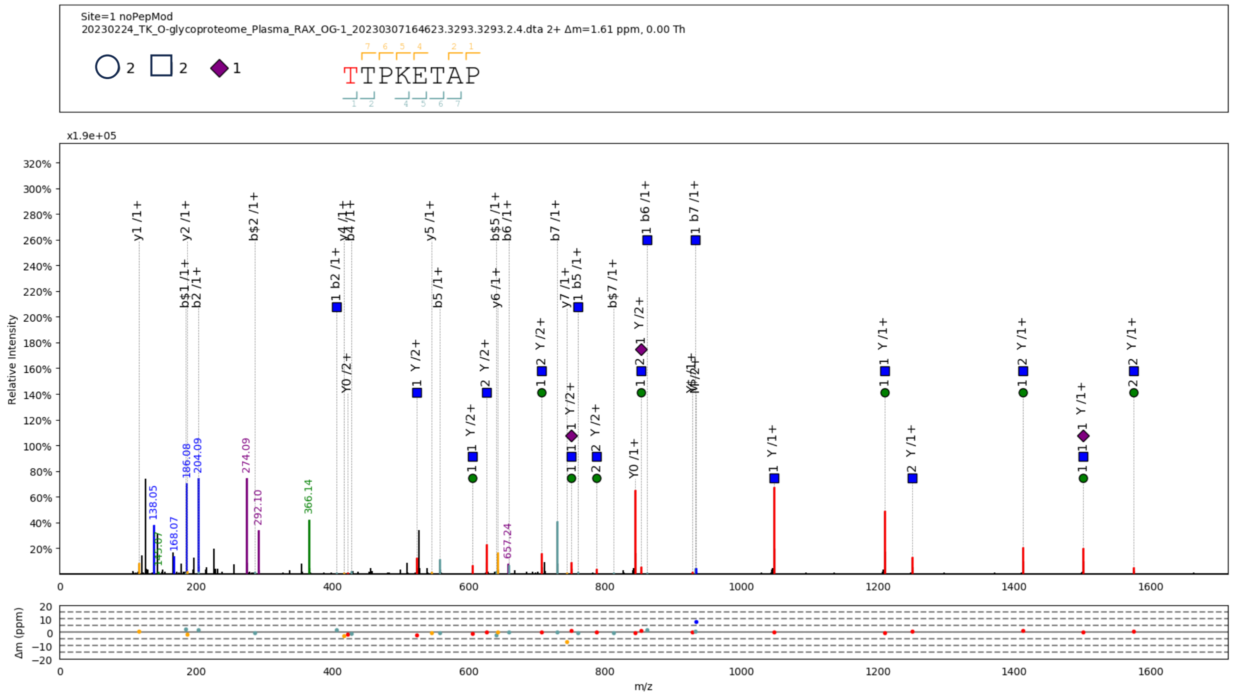

PRG4‒T611-T699-T821, H (1) N (2) A (1)

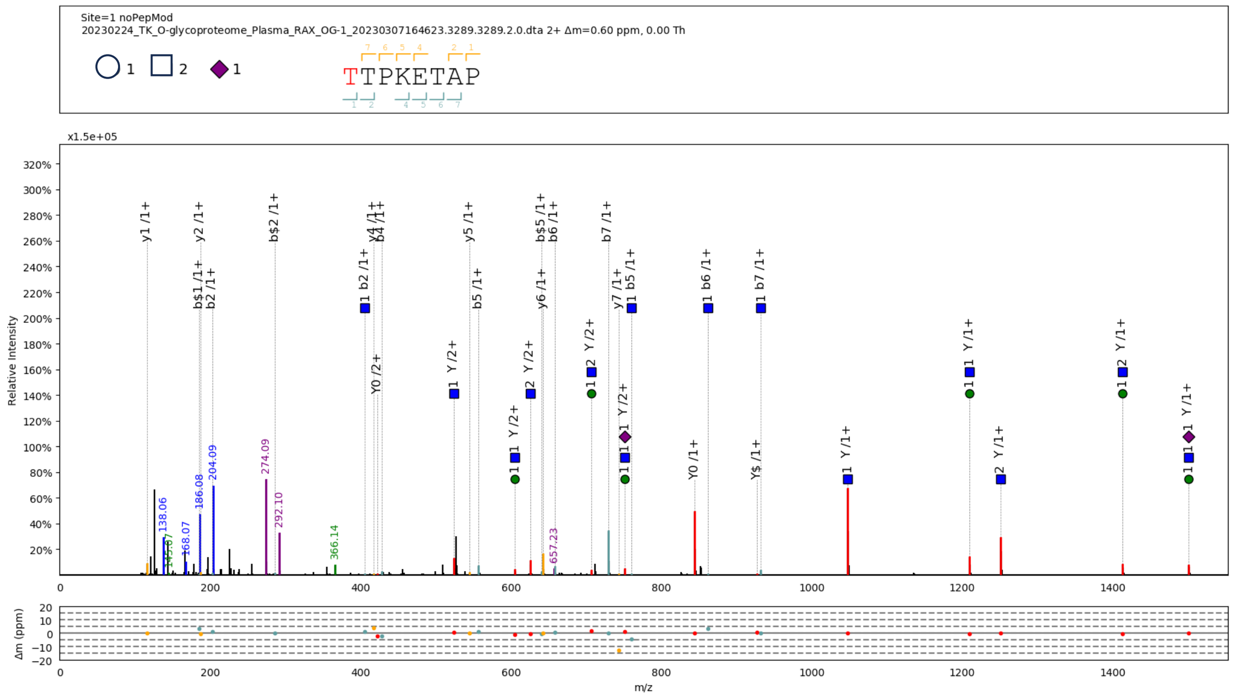

PRG4–T611-T699-T821, H (1) N (1) A (1)

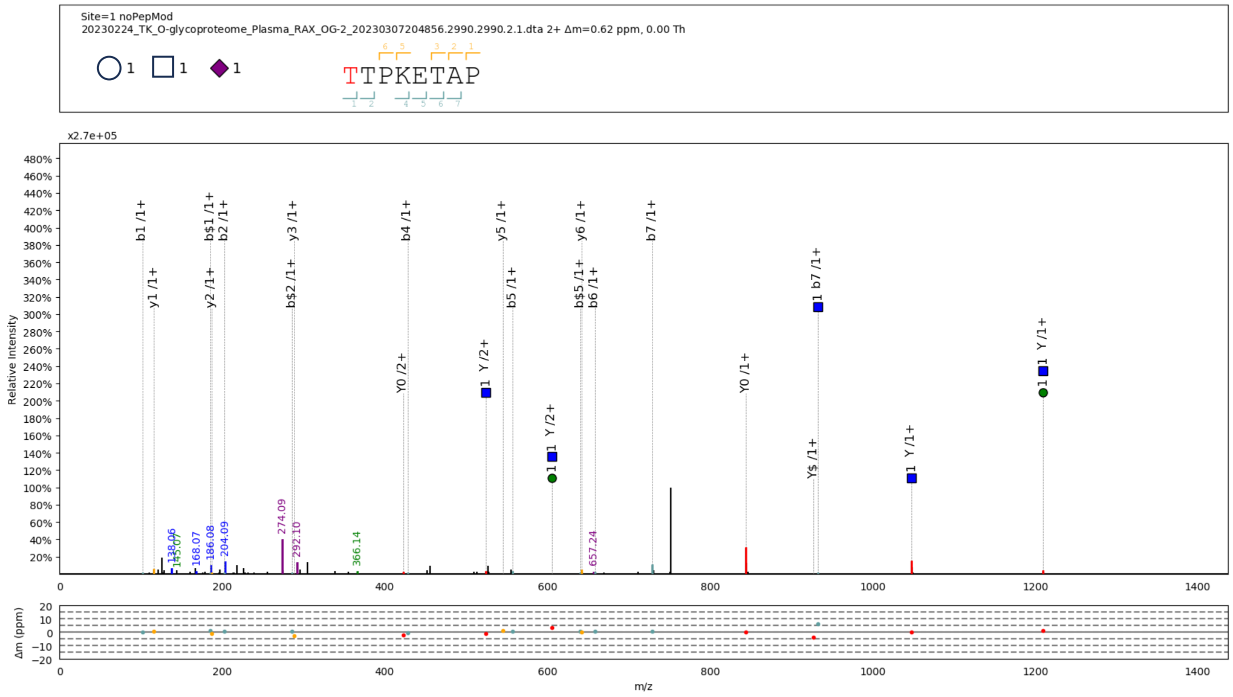

PRG4–T616-T704-T757-T826, H (2) N (2) A (2)

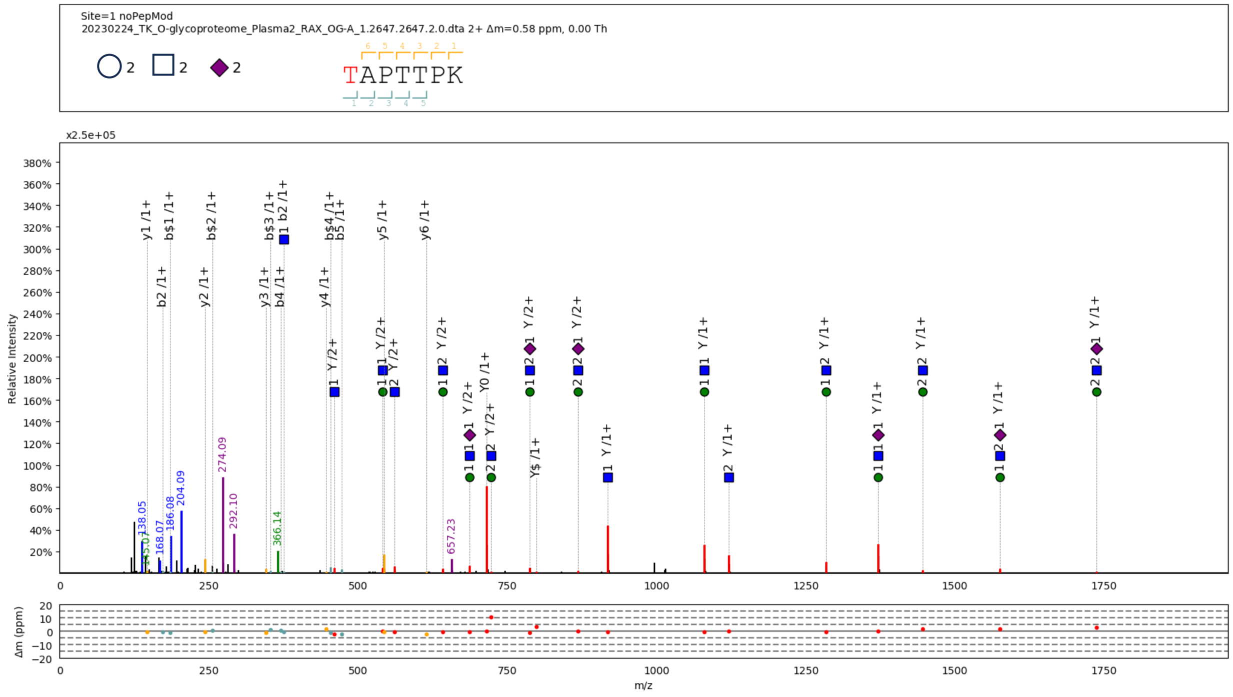

PRG4–T616-T704-T757-T826, H (2) N (2) A (1)

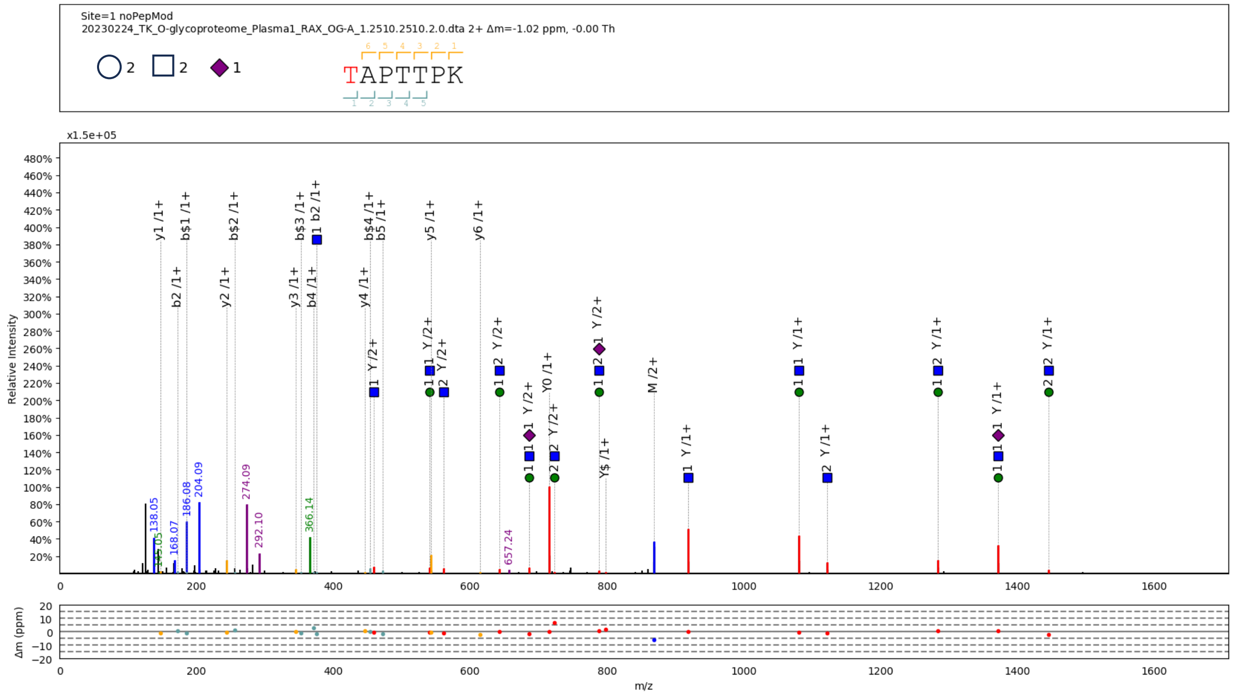

PRG4–T619, H (2) N (2) A (2)

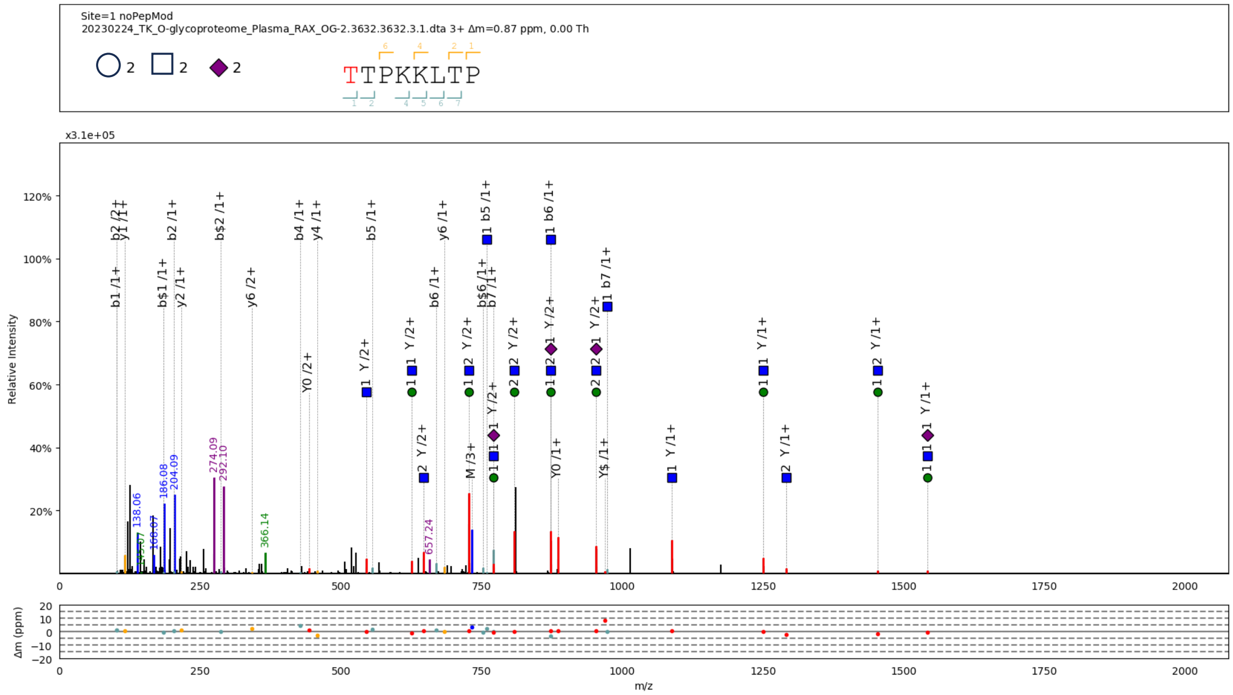

PRG4–T635, H (2) N (2) A (2)

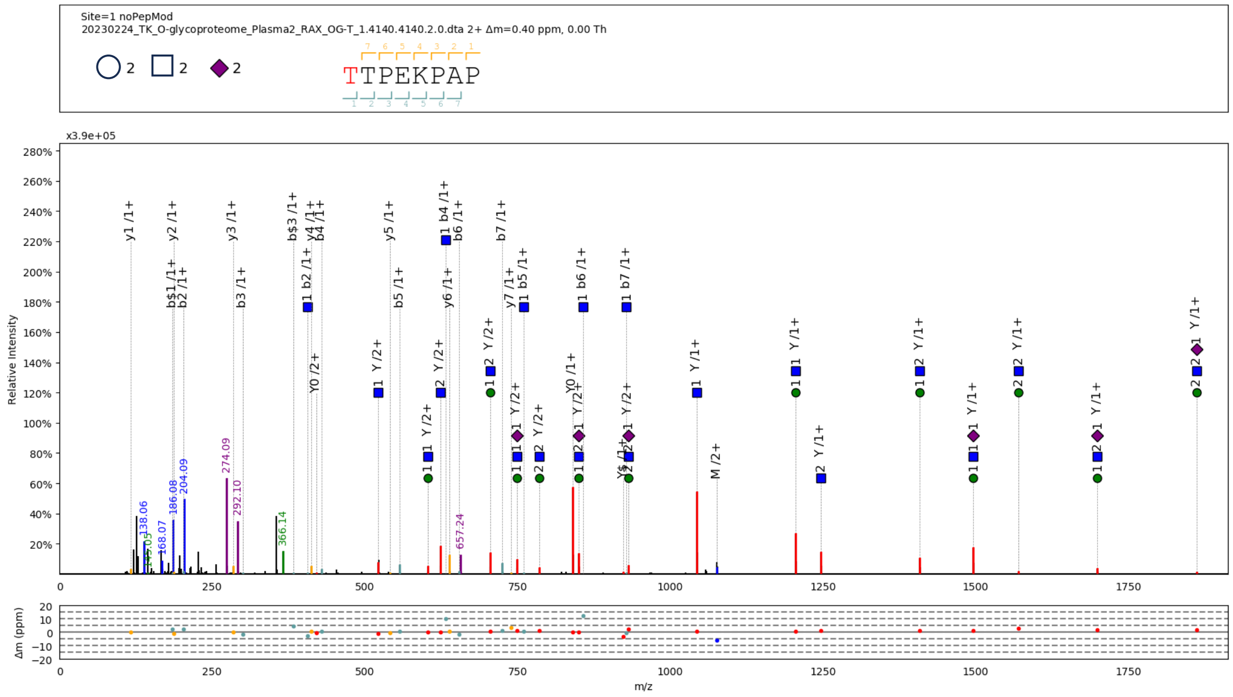

PRG4–T635, H (2) N (2) A (1)

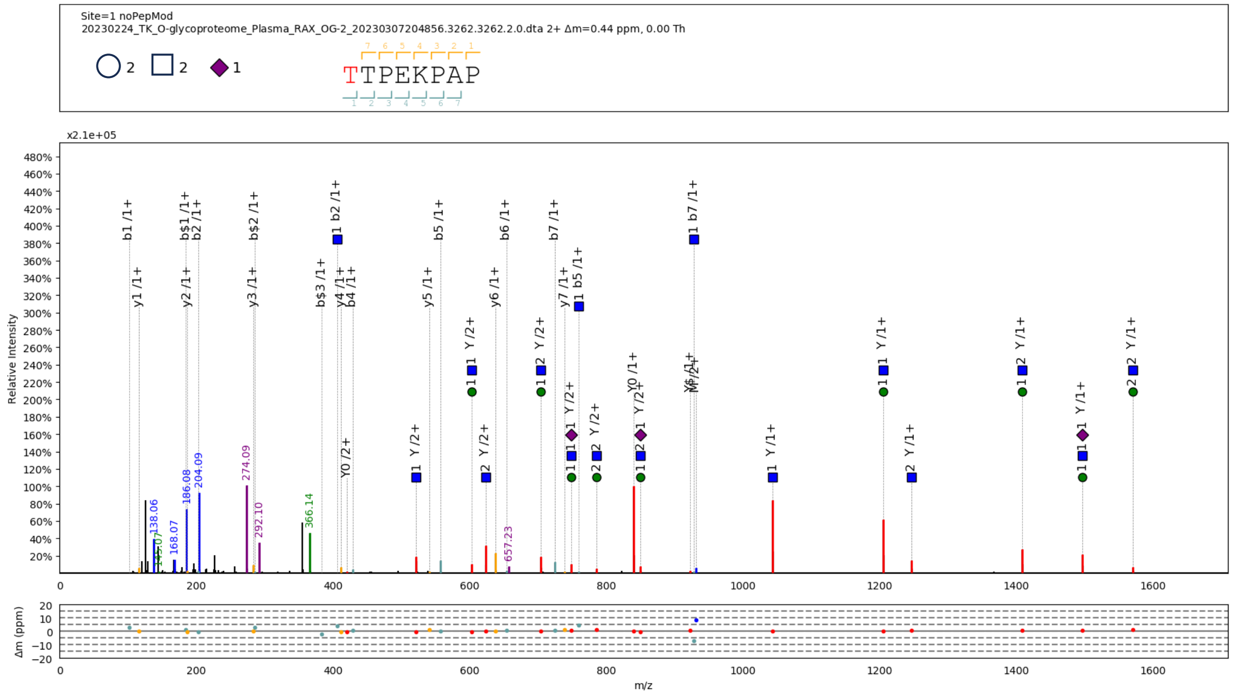

PRG4–T635, H (1) N (1) A (1)

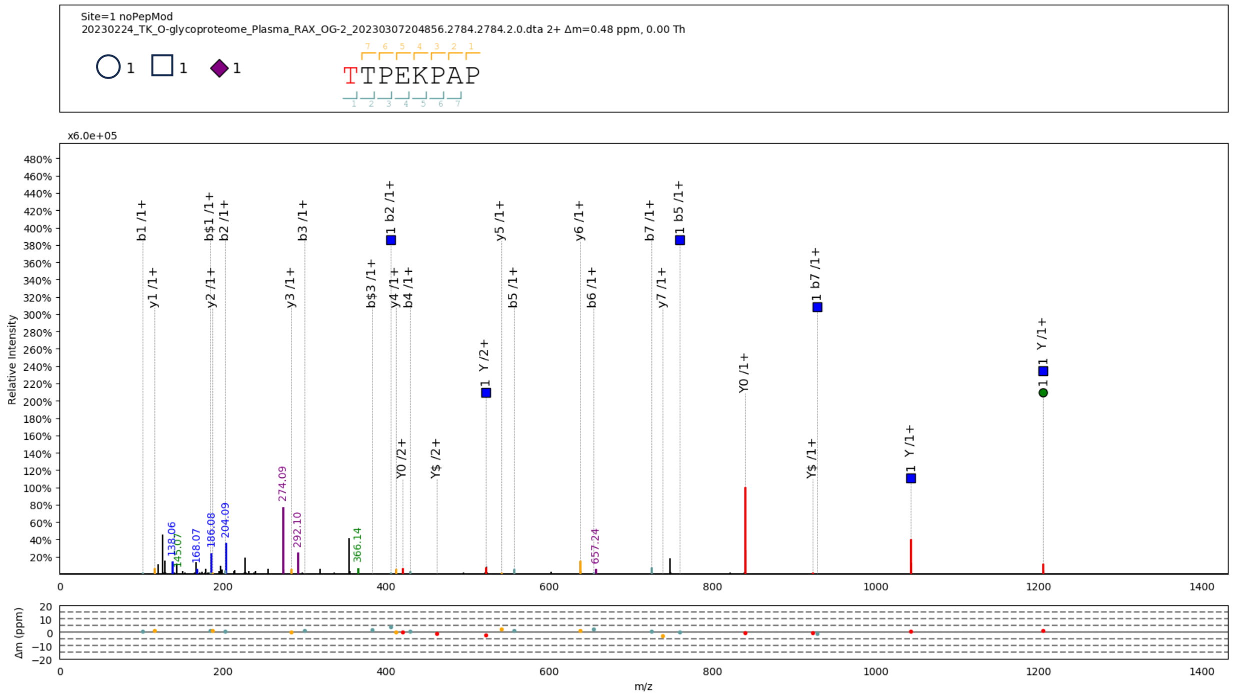

PRG4–T635, H (1) N (2) A (1)

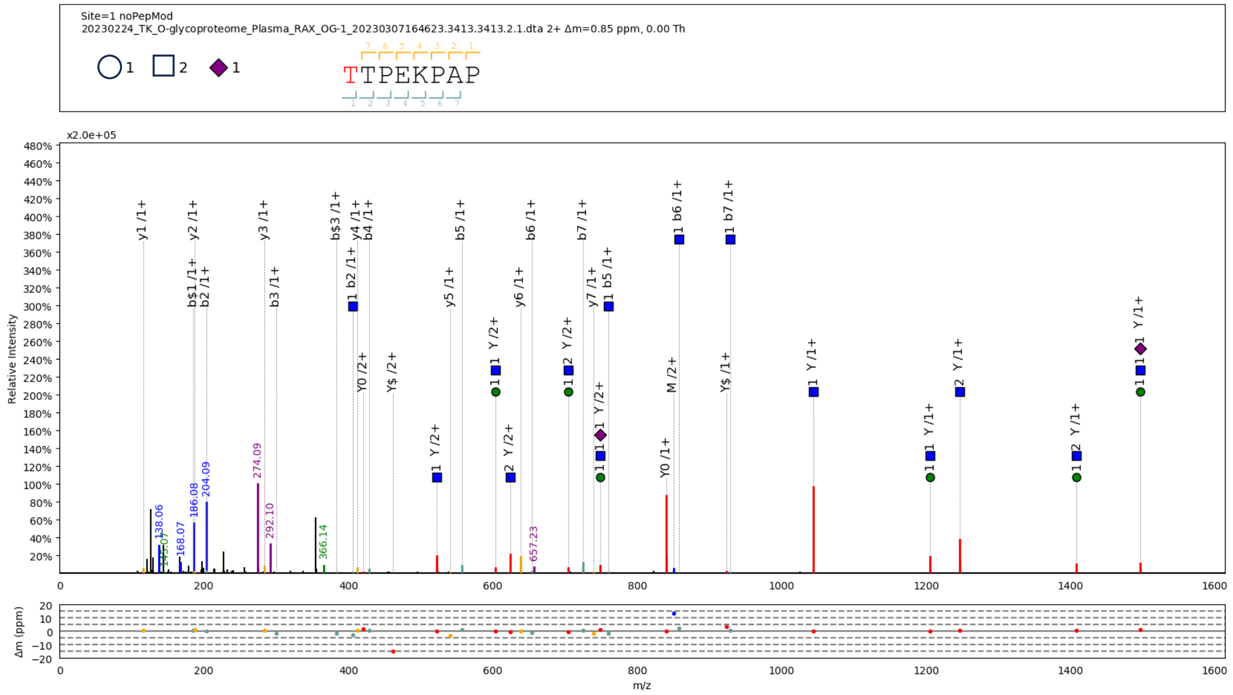

PRG4−T635, H (1) N (1) A (2)

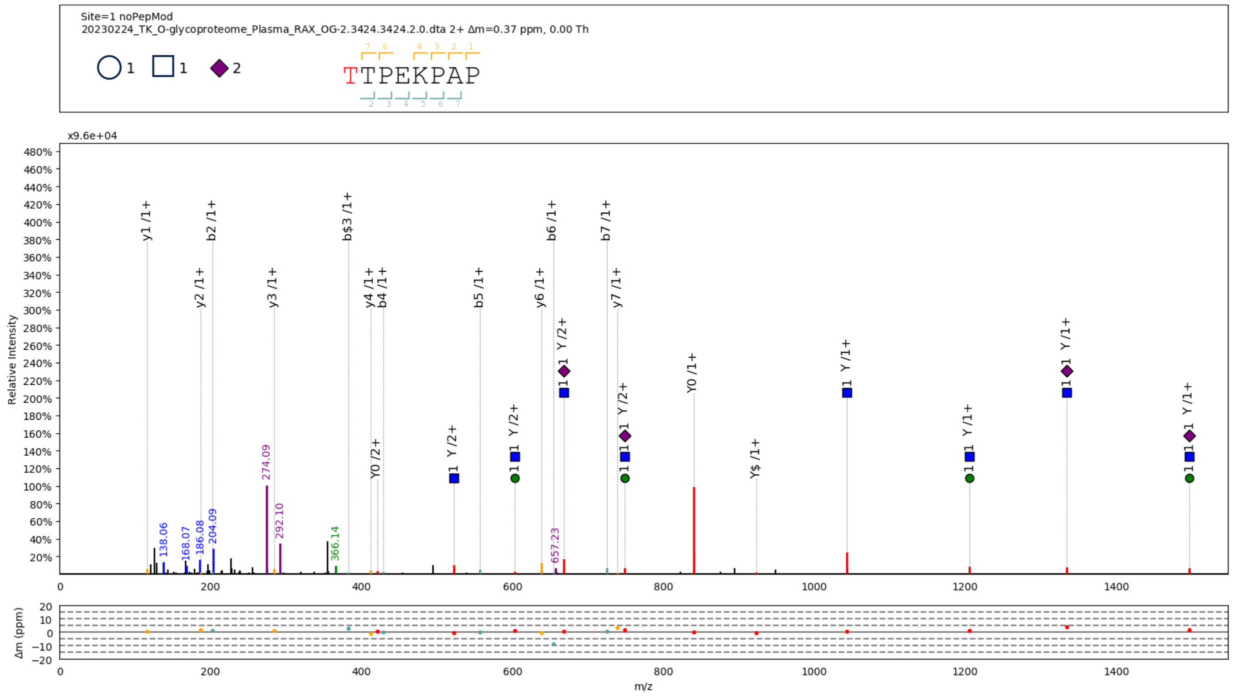

PRG4−T635, H (1) N (1)

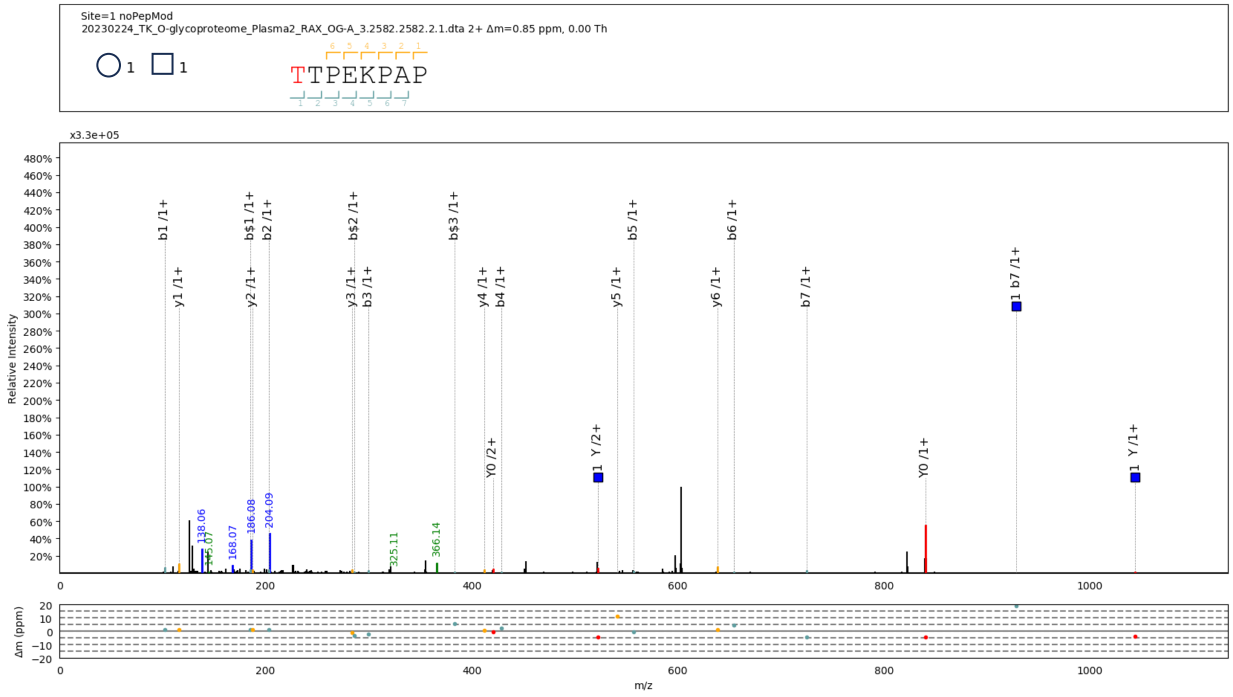

PRG4–T635, H (3) N (2) A (1) F (1)

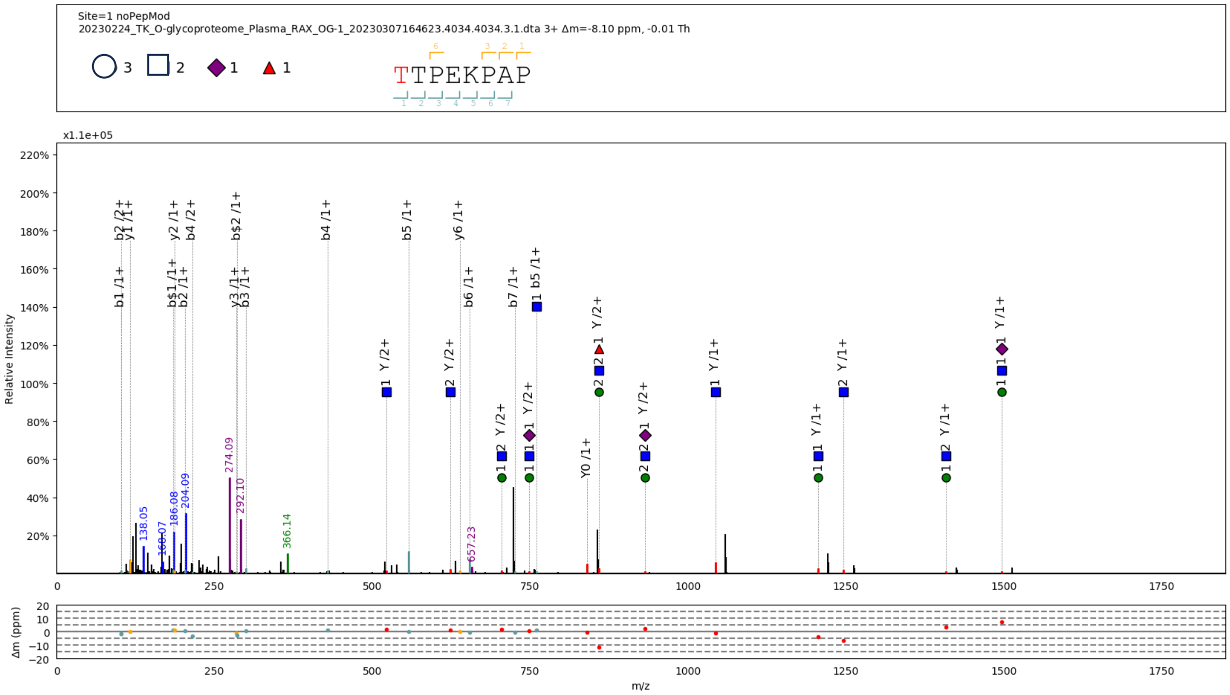

PRG4–T651, H (1) N (1) A (1)

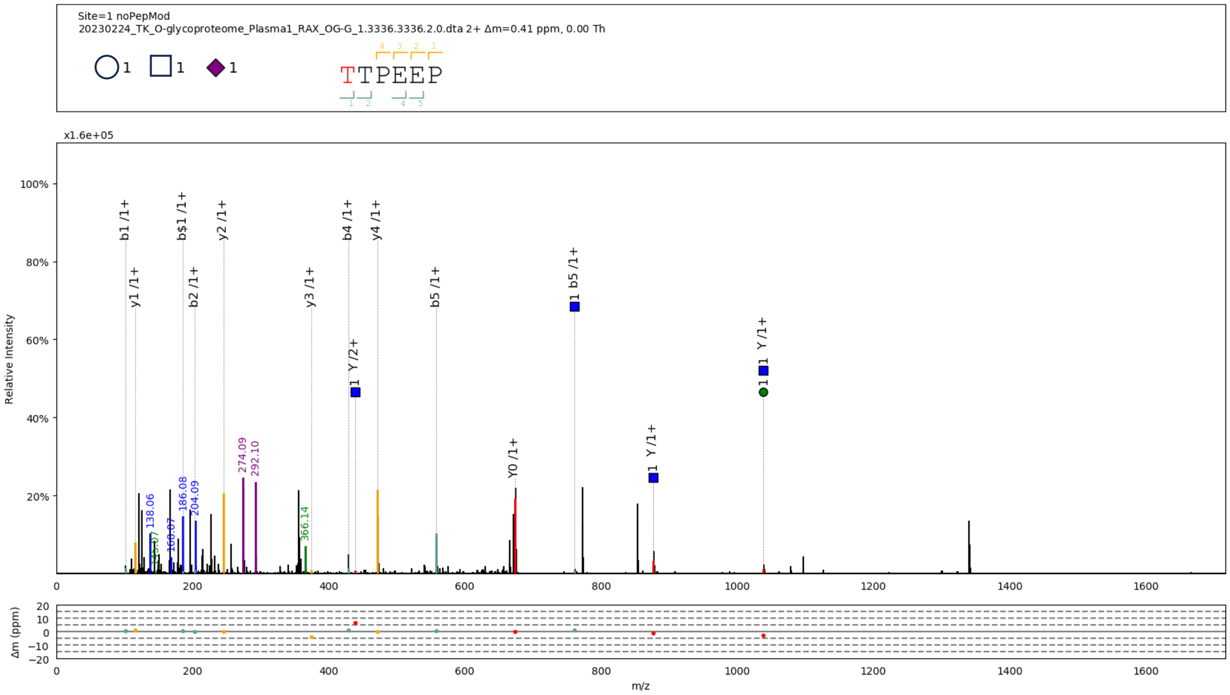

PRG4–T657, H (2) N (2) A (2)

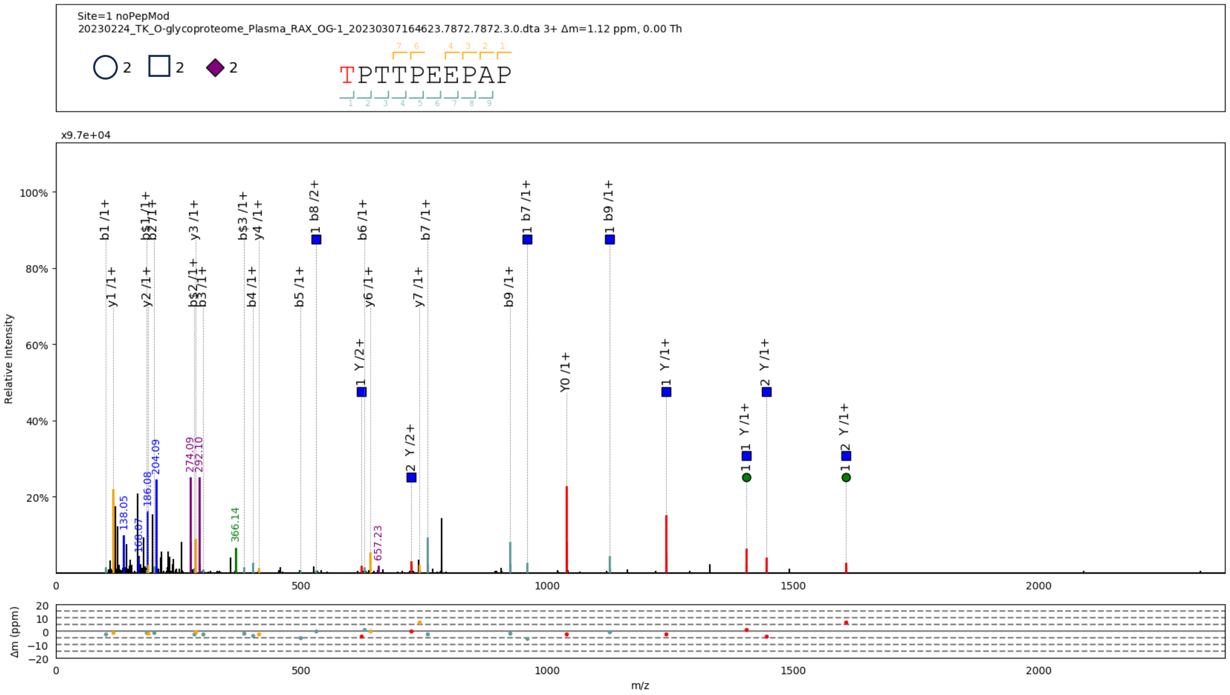

PRG4–T667, H (3) N (3) A (3)

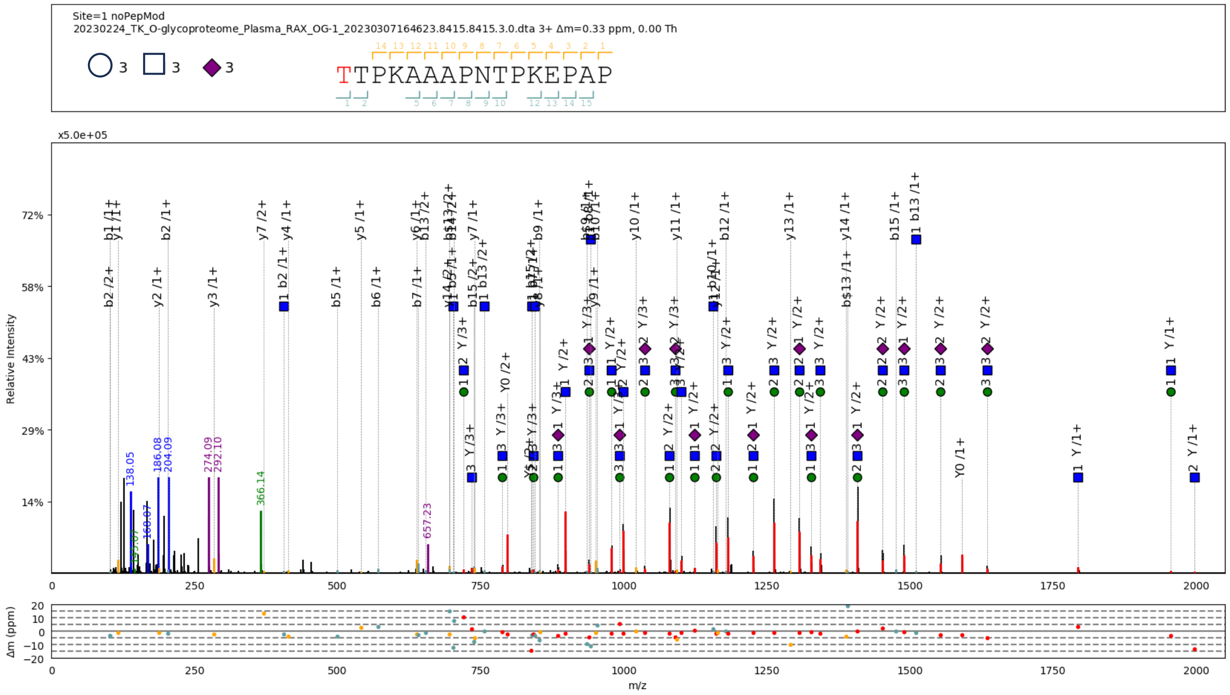

PRG4–T667, H (2) N (2) A (2)

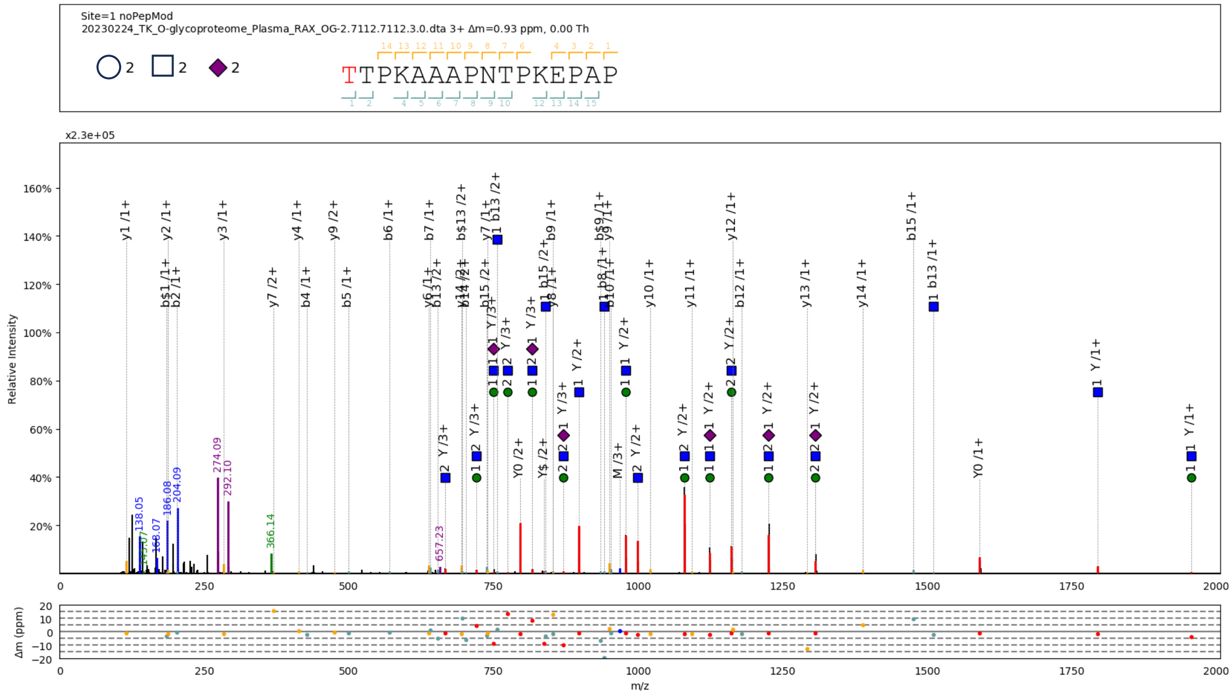

PRG4–T668, H (2) N (2) A (2)

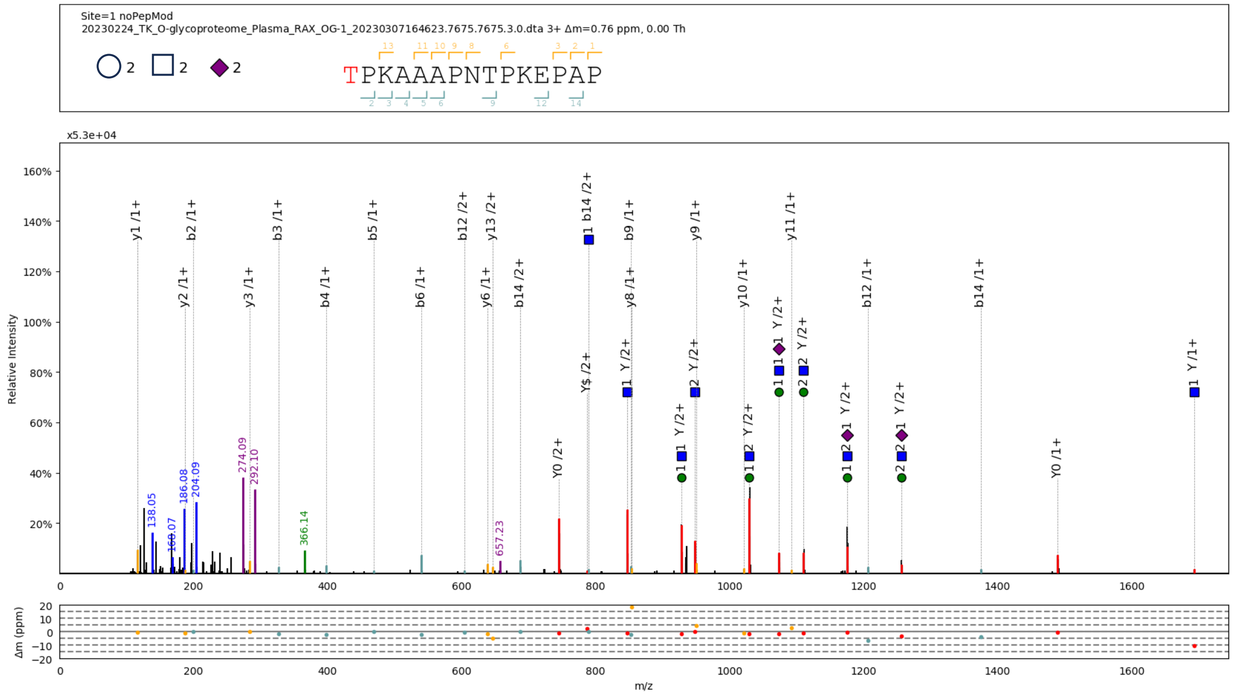

PRG4−T668, H (1) N (1) A (1)

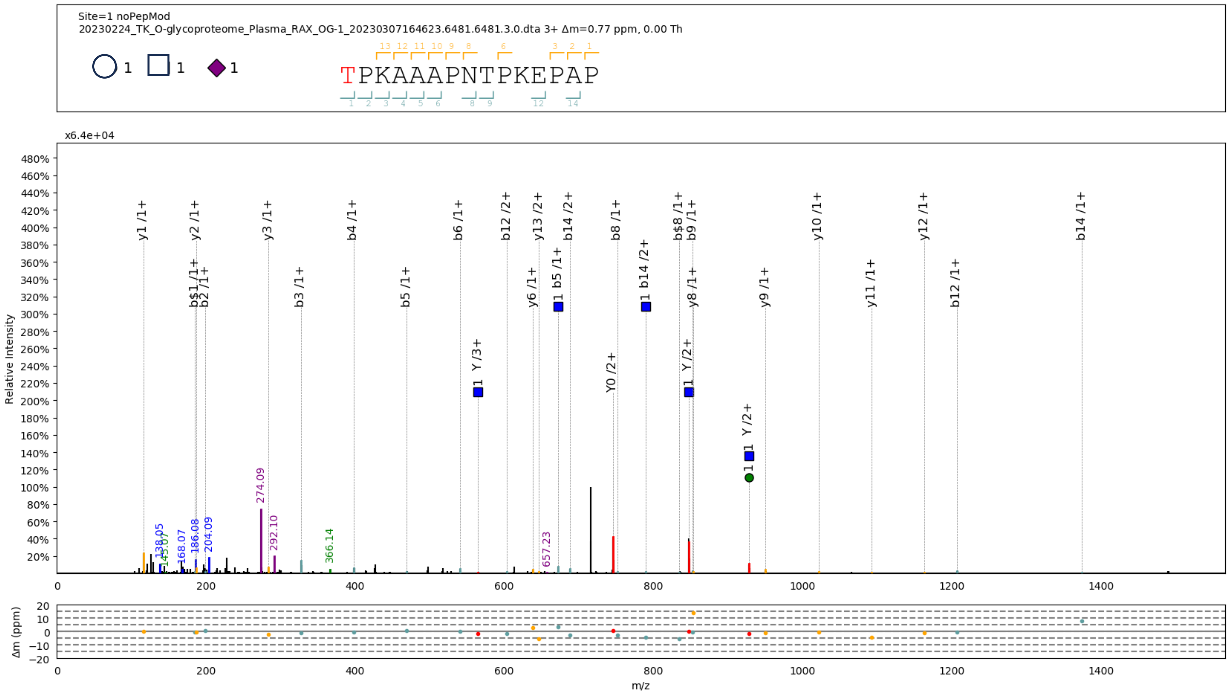

PRG4−T712, H (2) N (2) A (2)

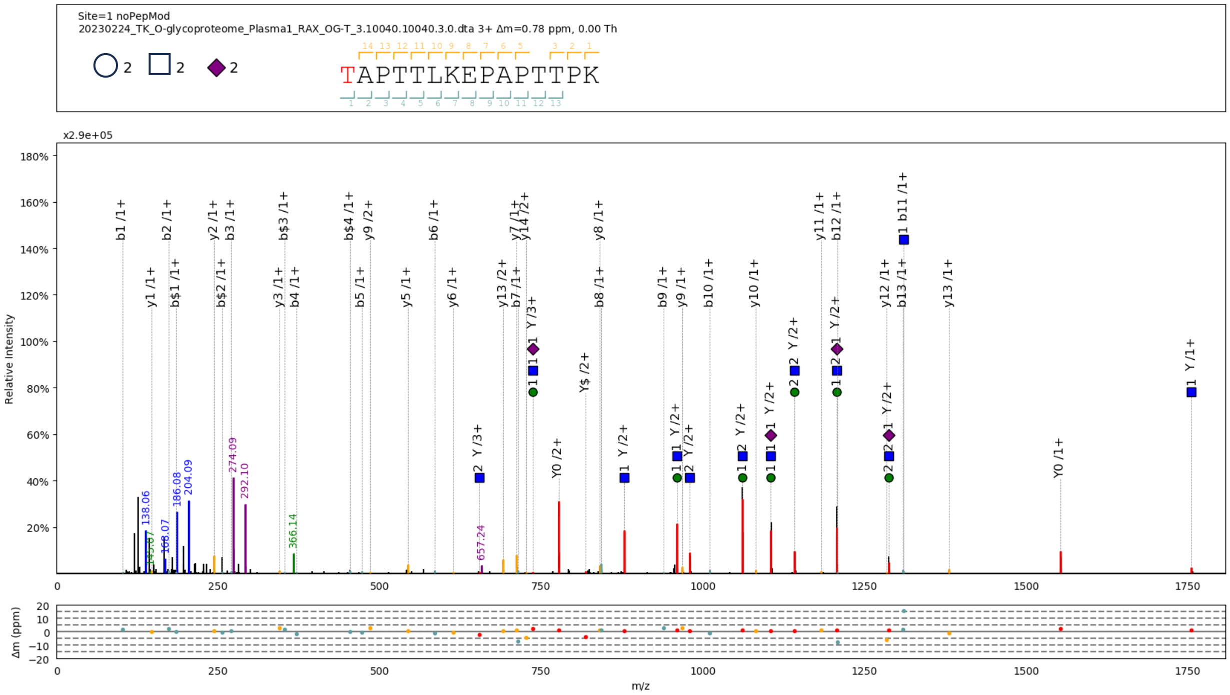

PRG4–T712, H (1) N (1) A (1)

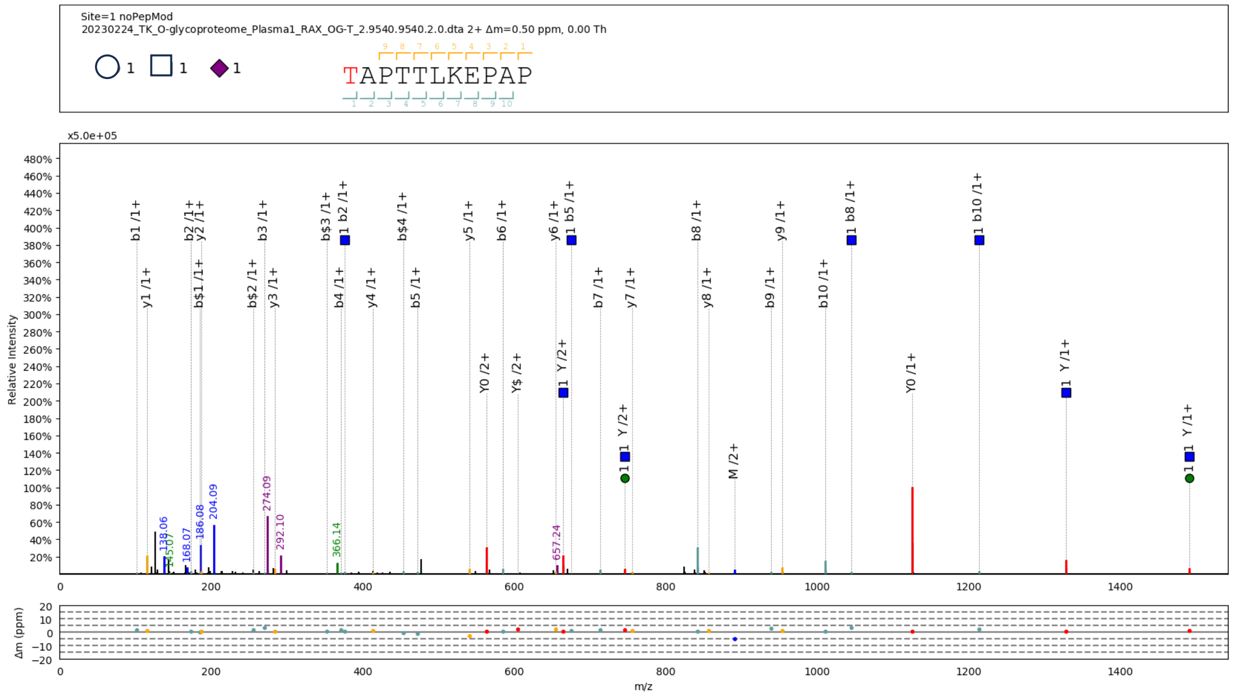

PRG4–T712, H (2) N (2) A (1)

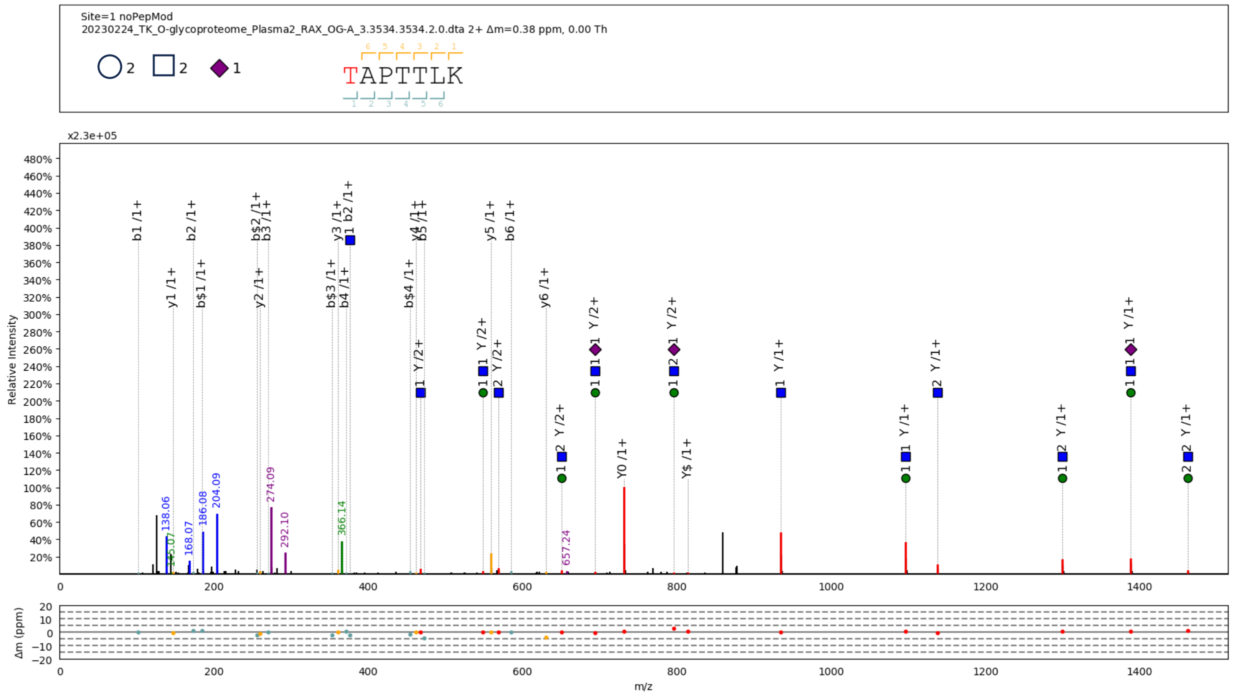

PRG4–T712, H (1) N (1)

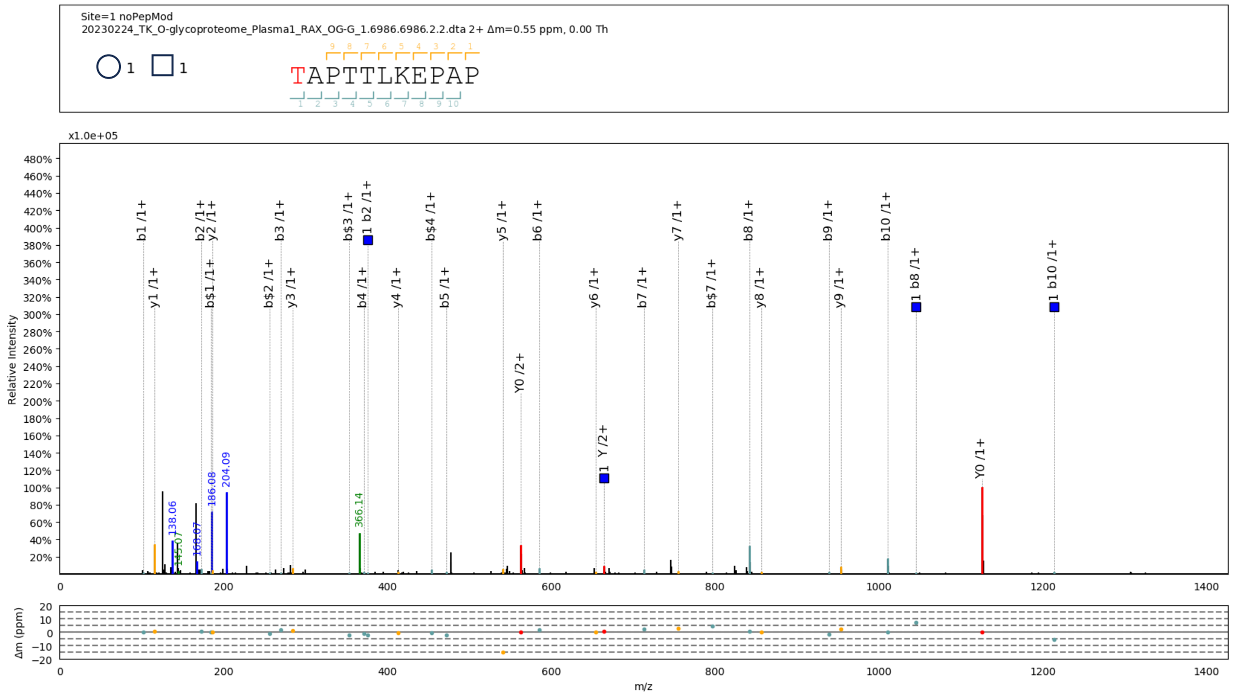

PRG4–T712, N (1)

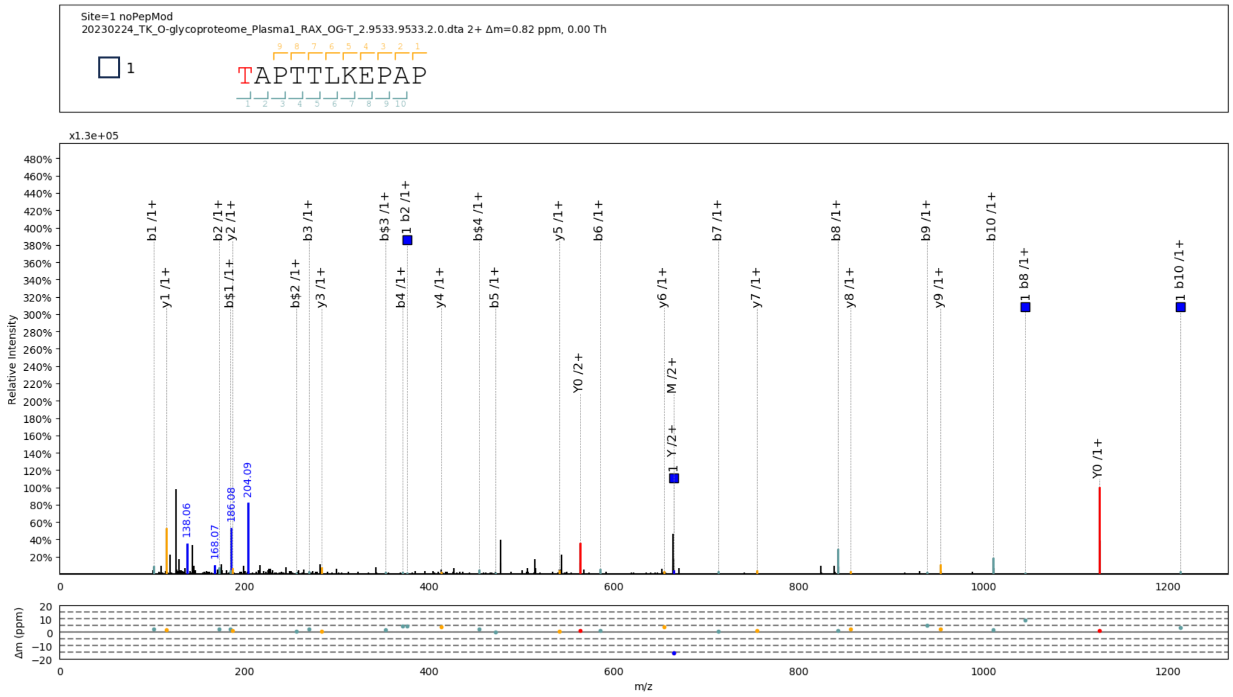

PRG4–T712, H (1) N (2) A (1)

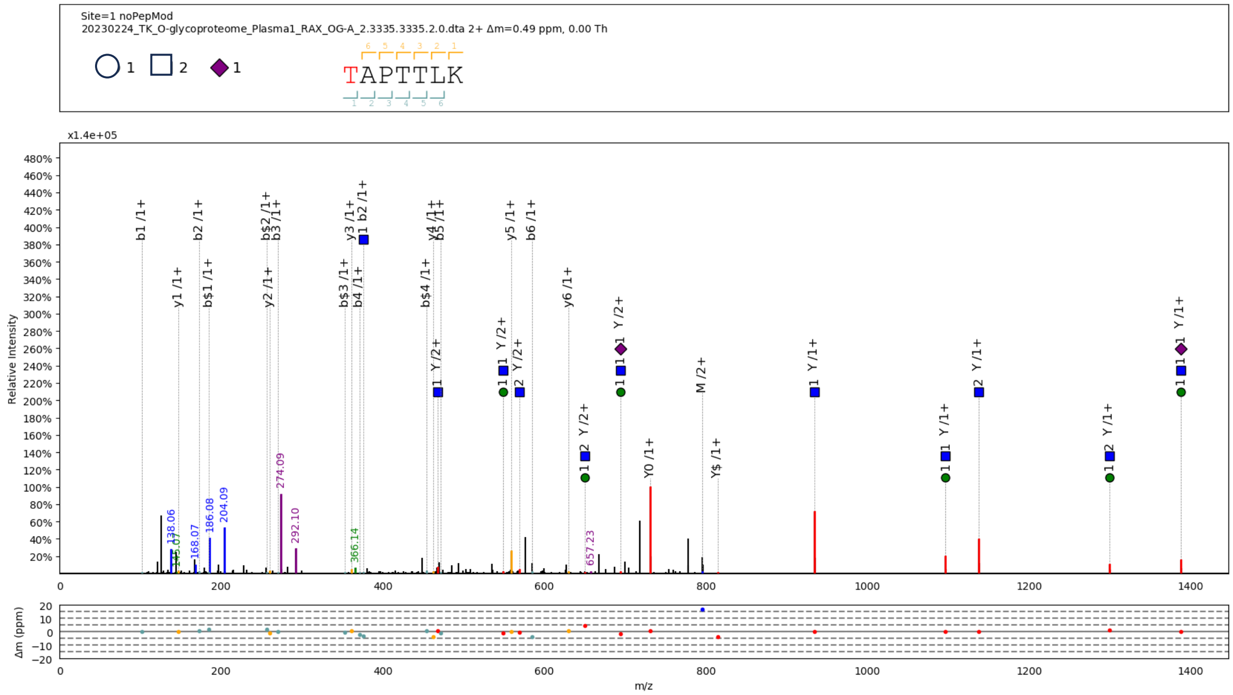

PRG4–T712, H (1) N (1) A (2)

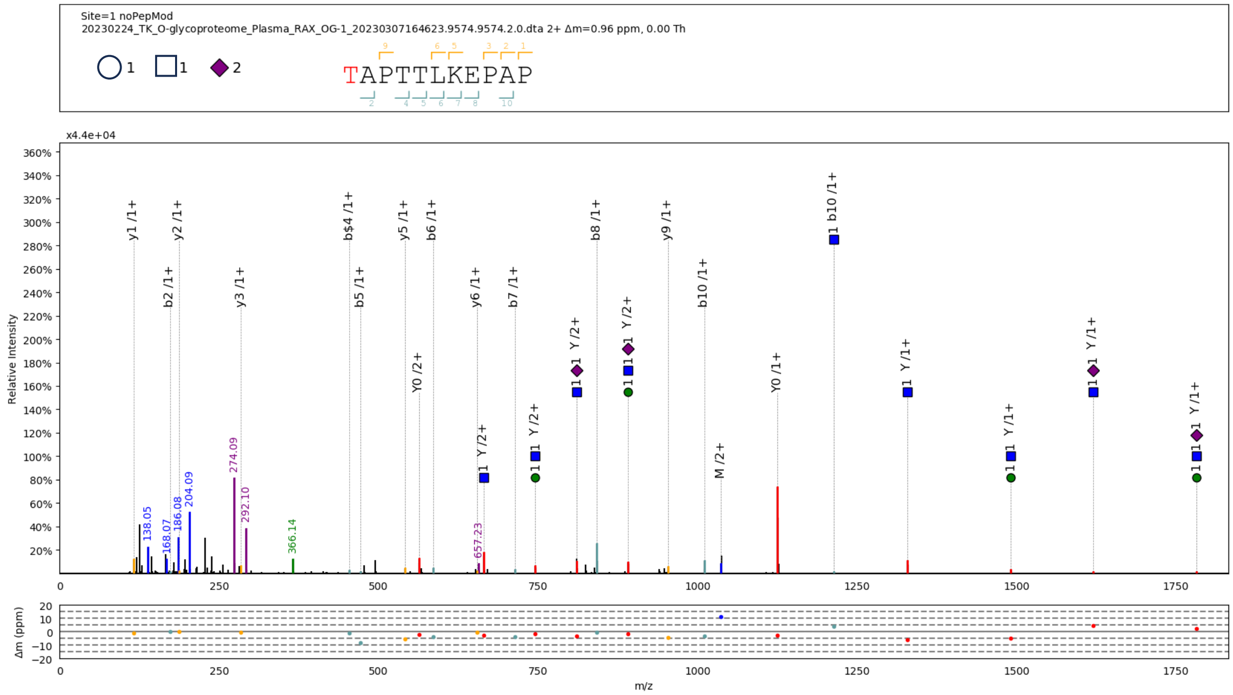

PRG4–T723-T792, H (1) N (1) A (1)

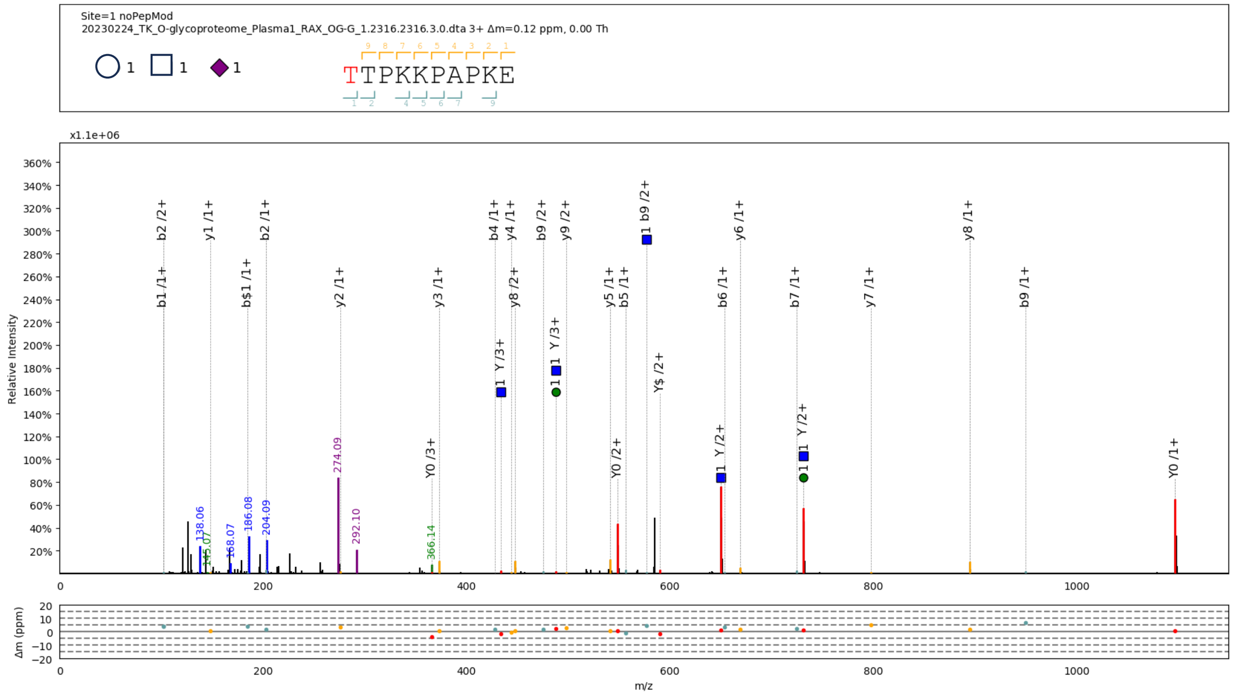

PRG4–T723-T792, H (2) N (2) A (2)

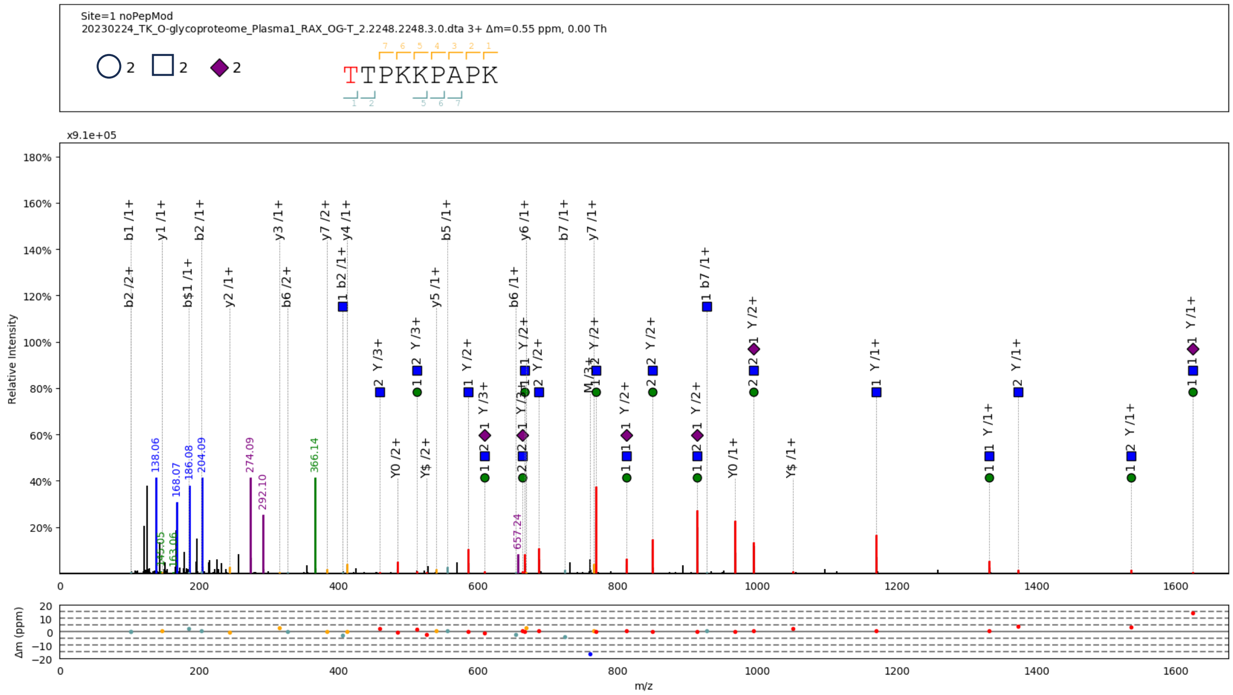

PRG4‒T723-T792, H (2) N (2) A (1)

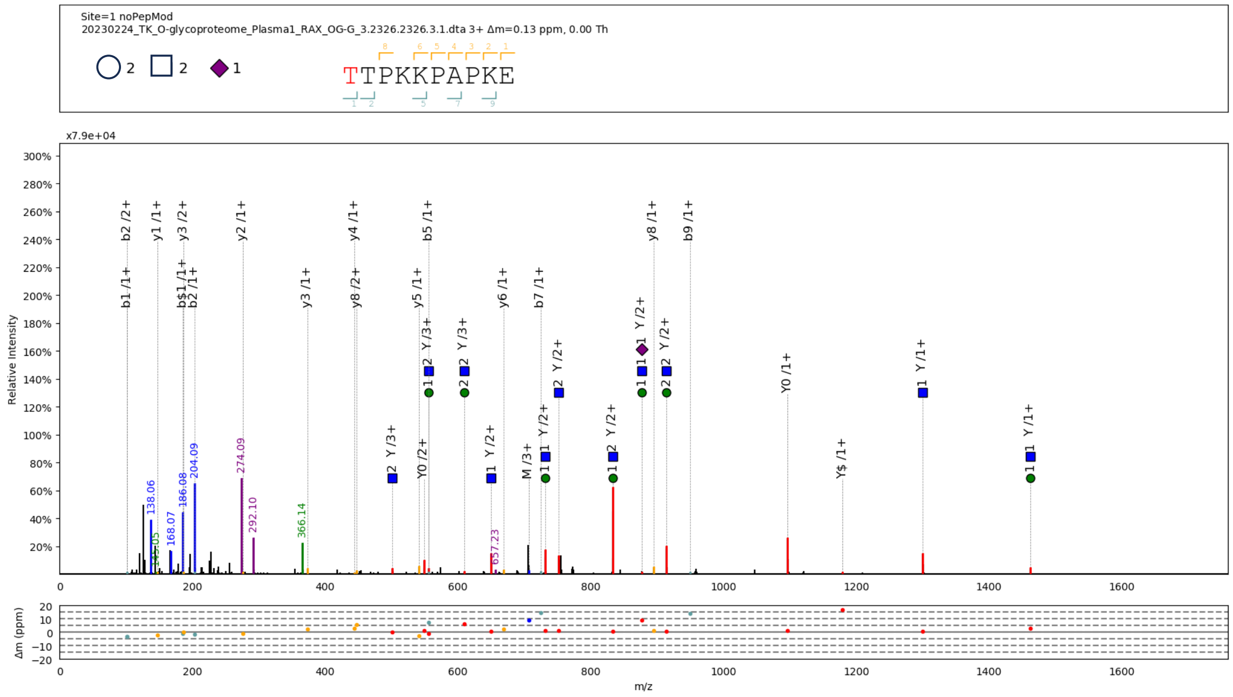

PRG4‒T723-T792, H (1) N (1)

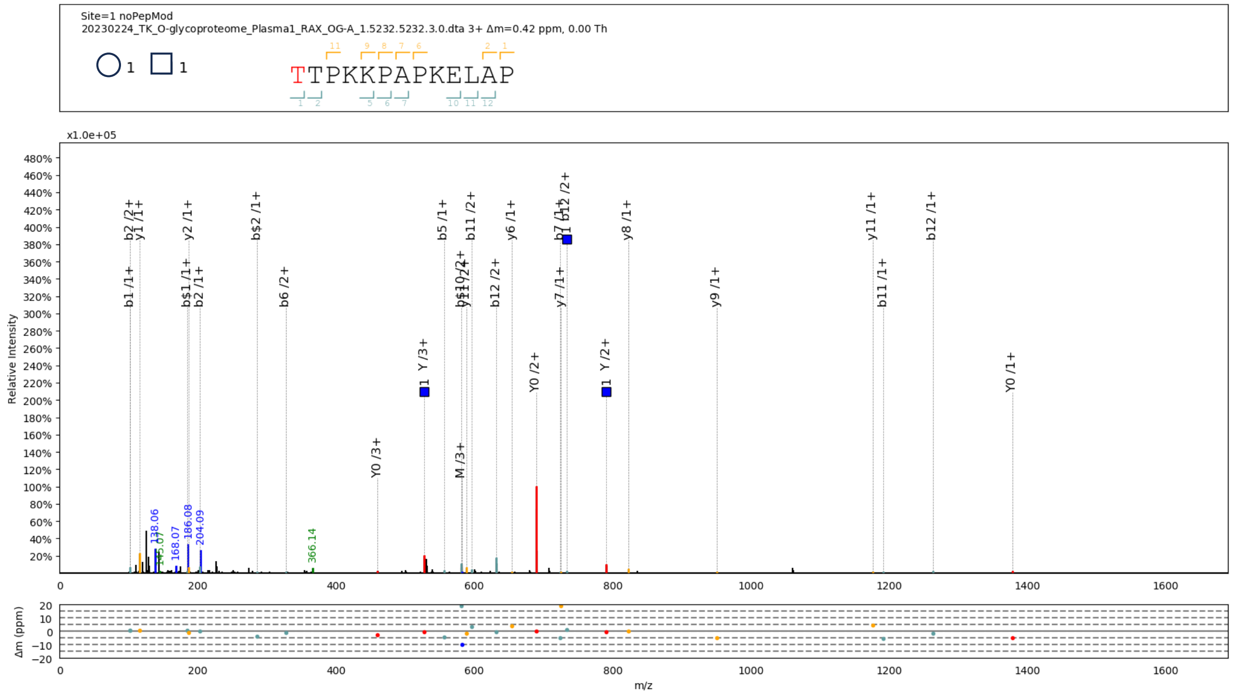

PRG4–T744-T813, H (2) N (2) A (2)

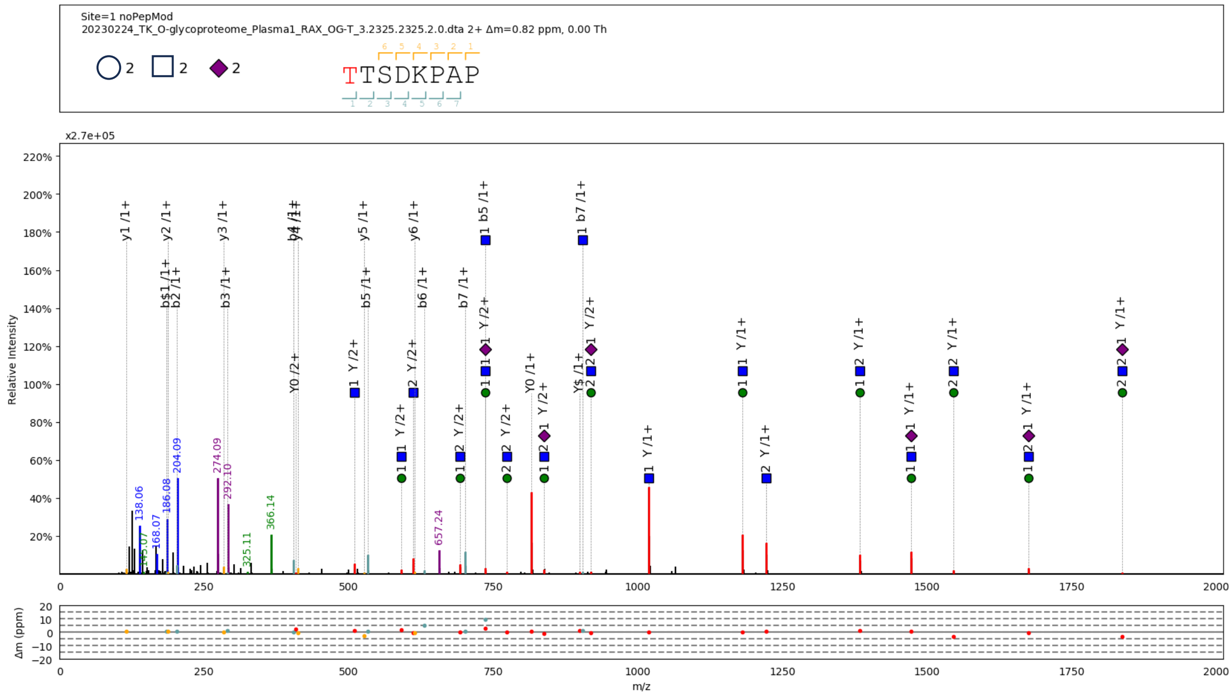

PRG4–T744-T813, H (2) N (2) A (1)

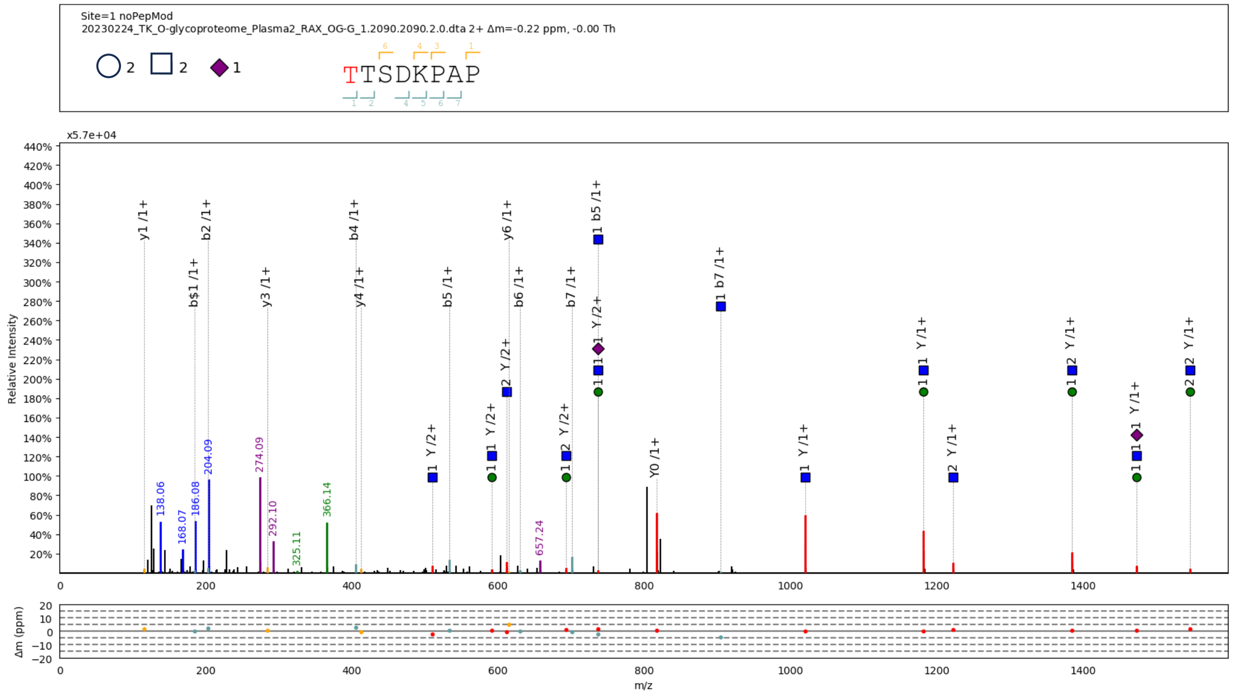

PRG4–T757-T826, H (2) N (2) A (2)

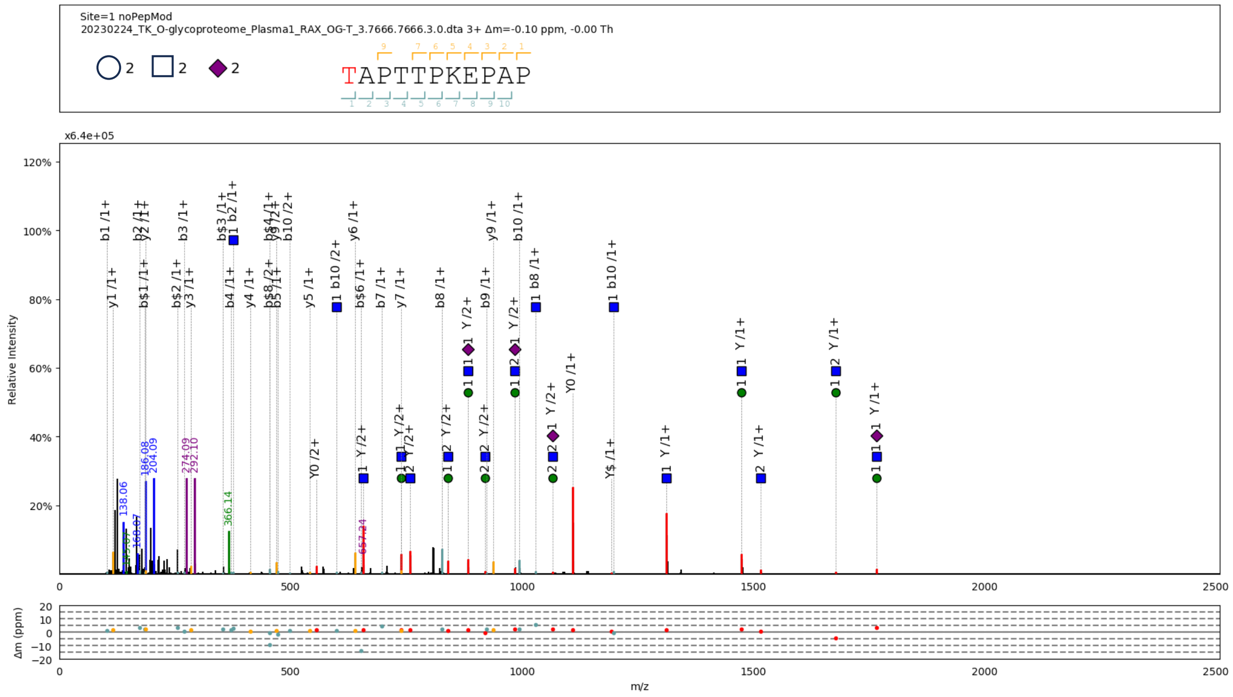

PRG4–T757-T826, H (3) N (3) A (3)

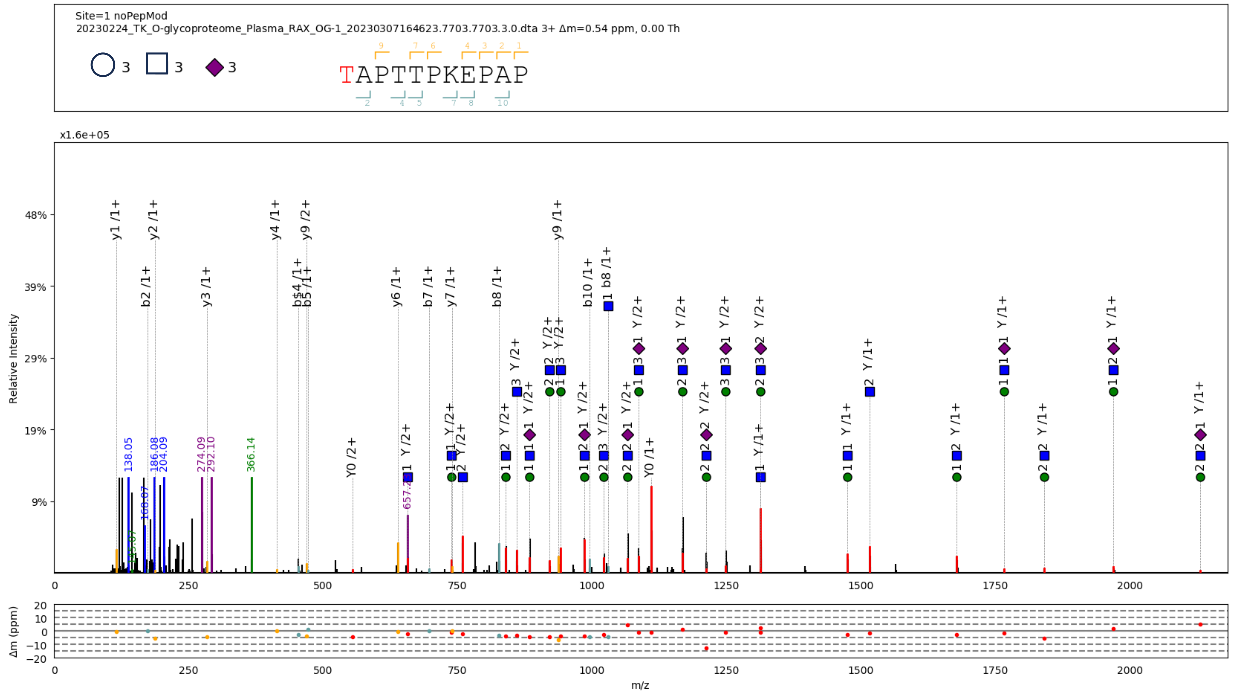

PRG4–T757-T826, H (2) N (2) A (1)

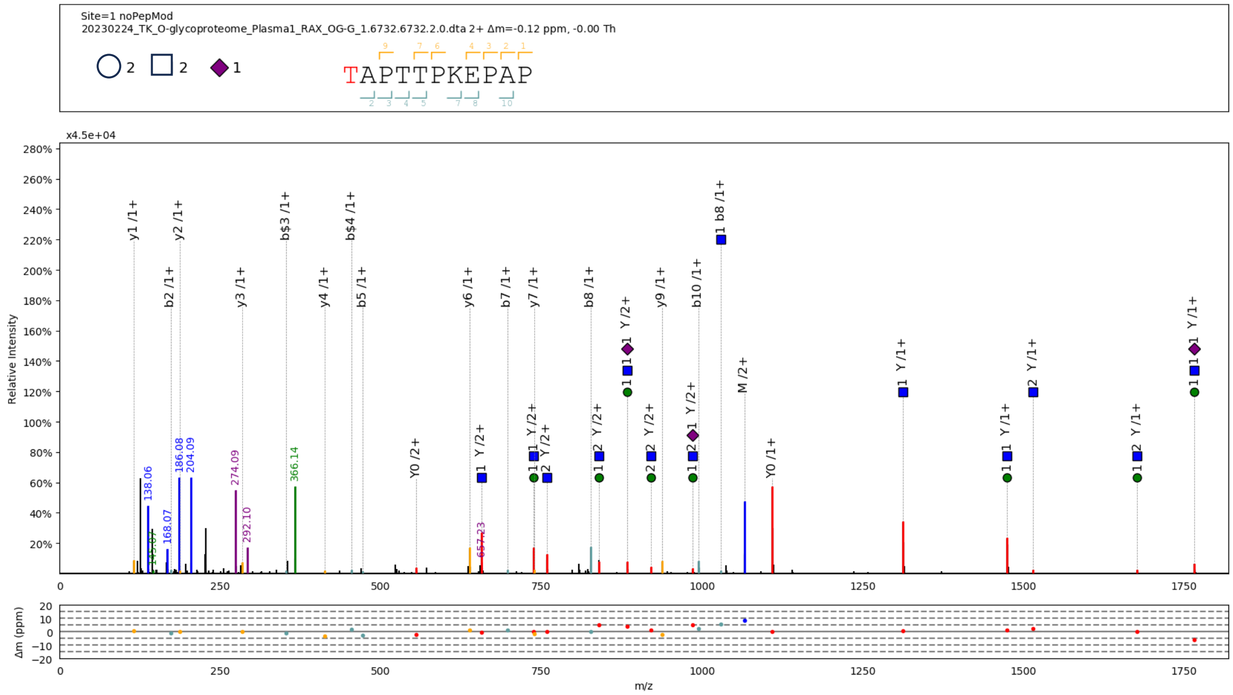

PRG4–T757-T826, H (1) N (2) A (1)

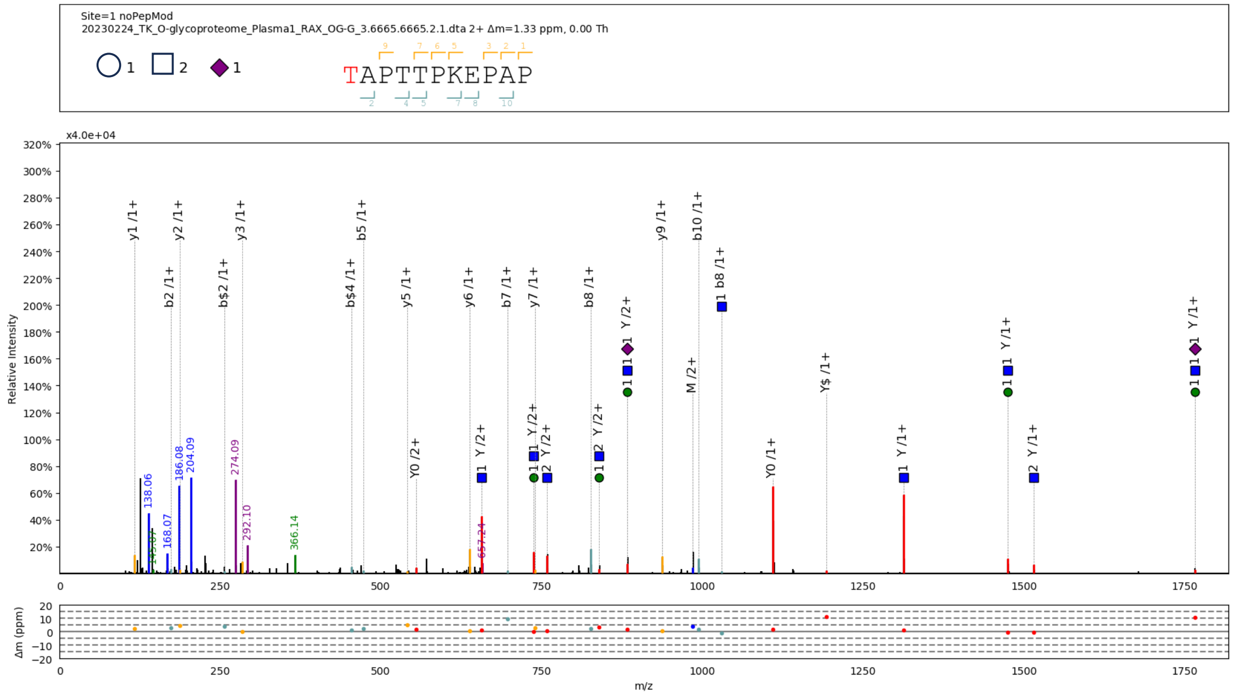

PRG4–T868, H (1) N (1)

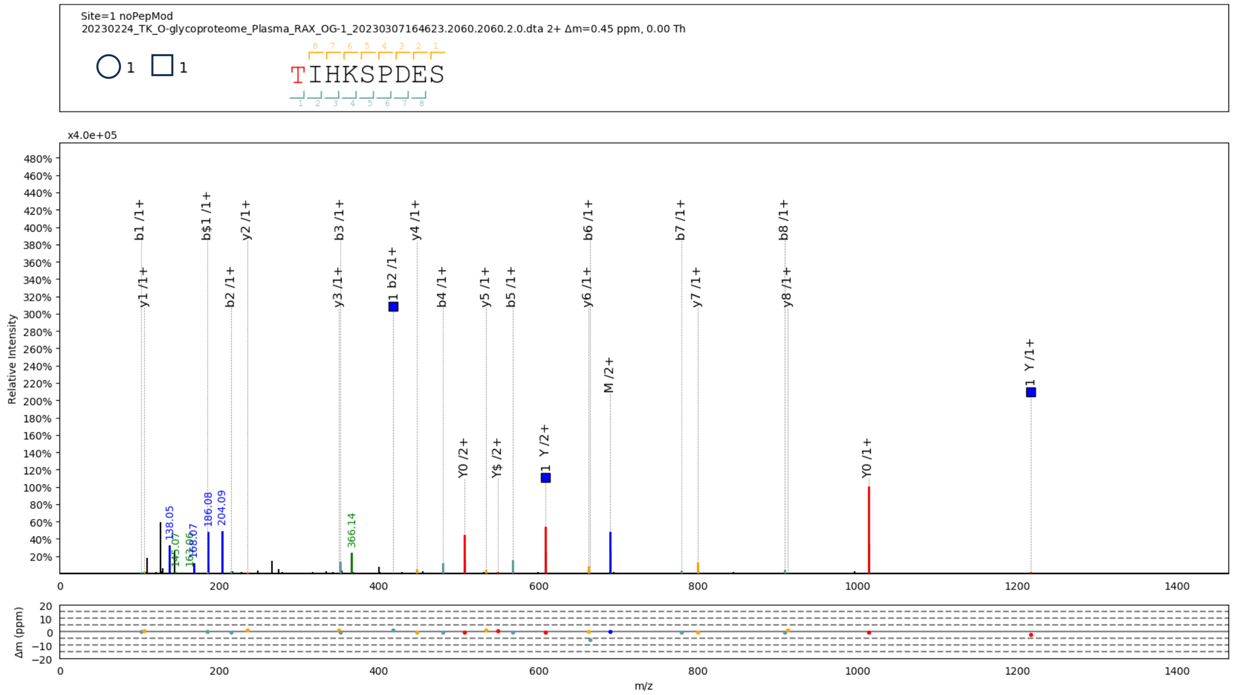

PRG4–T868, H (1) N (1) A (1)

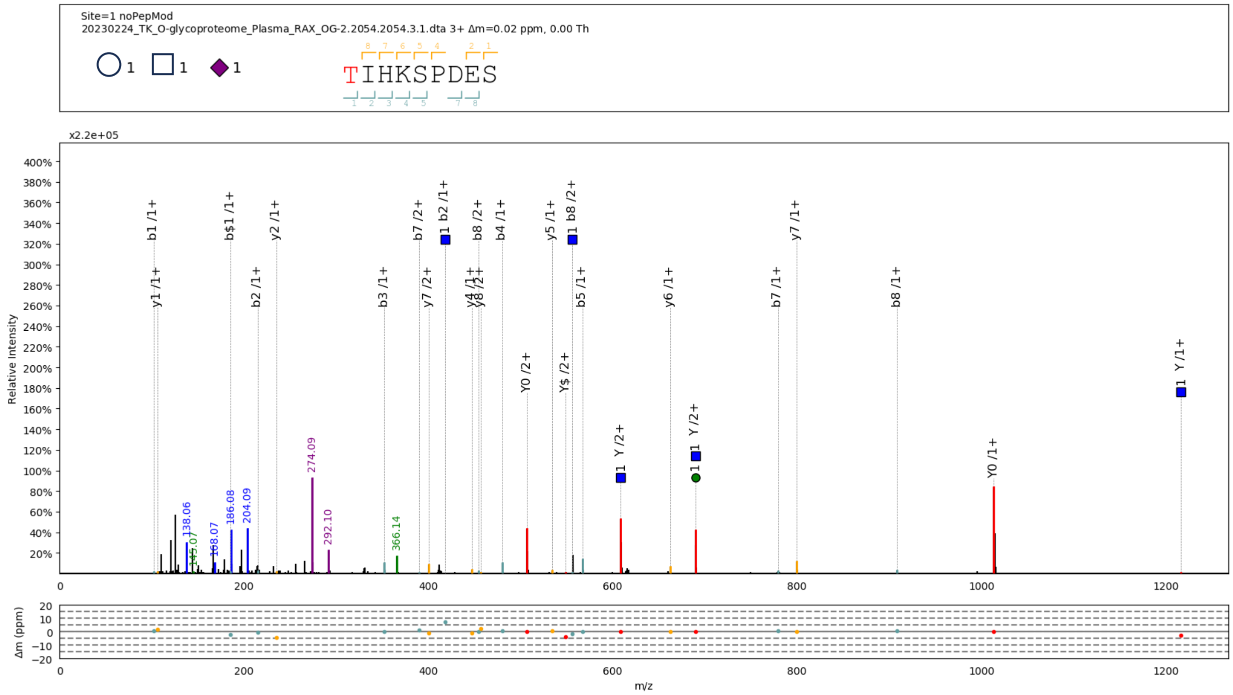

PRG4–T868, N (1)

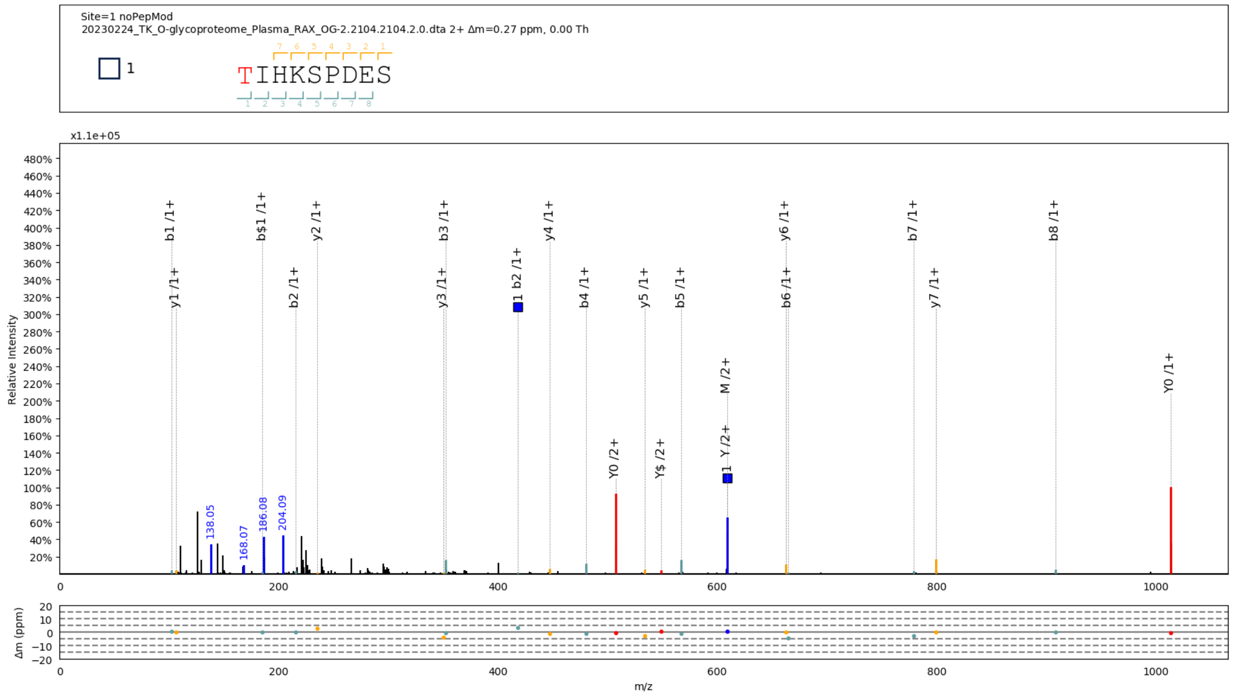

PRG4–T885, H (1) N (1) A (1)

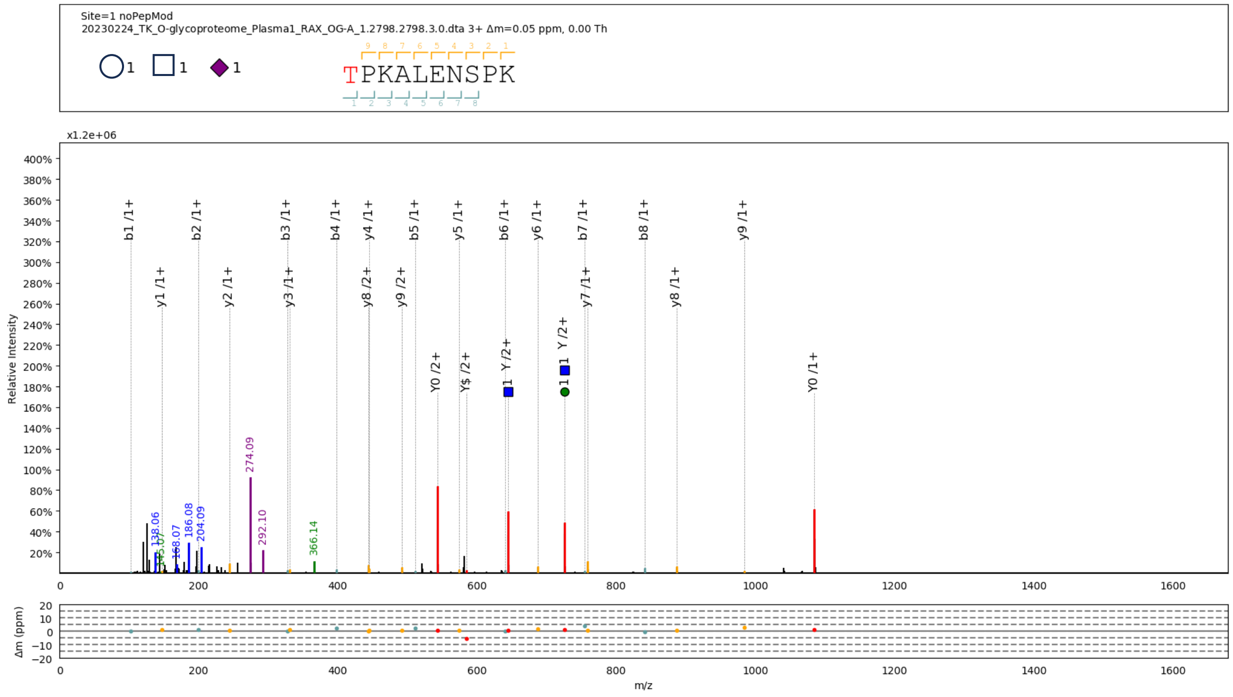

PRG4−T885, H (1) N (1) A (2)

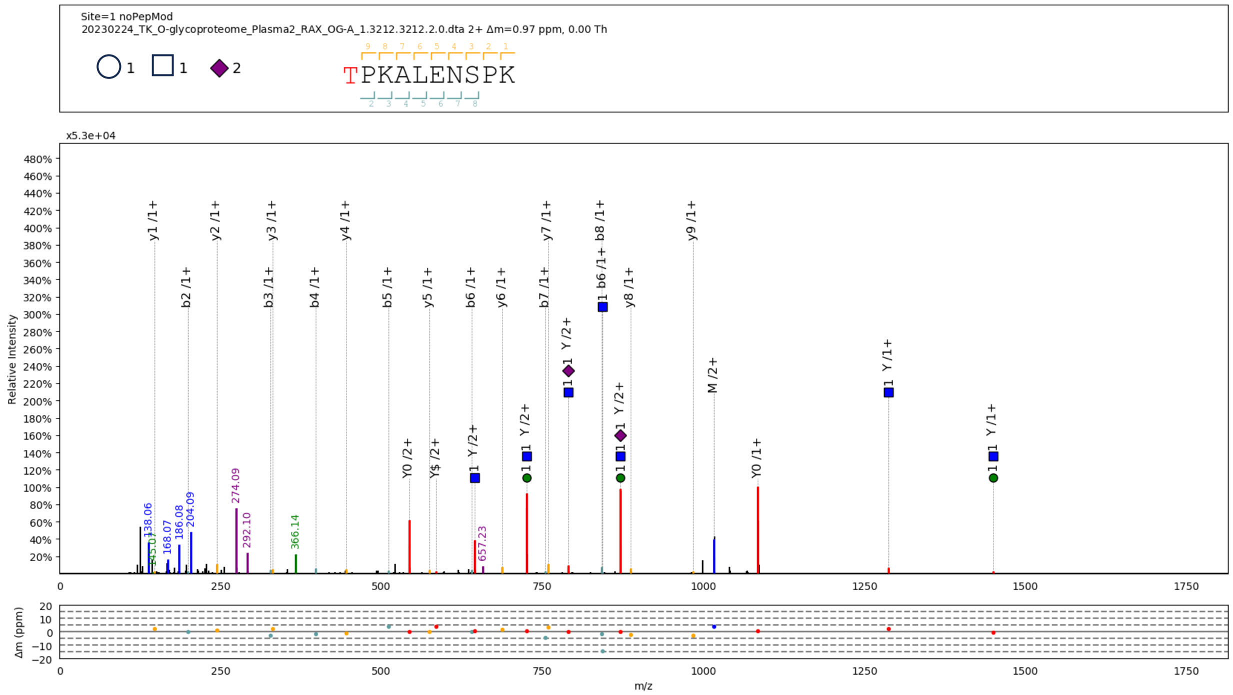

PRG4−T885, H (1) N (1)

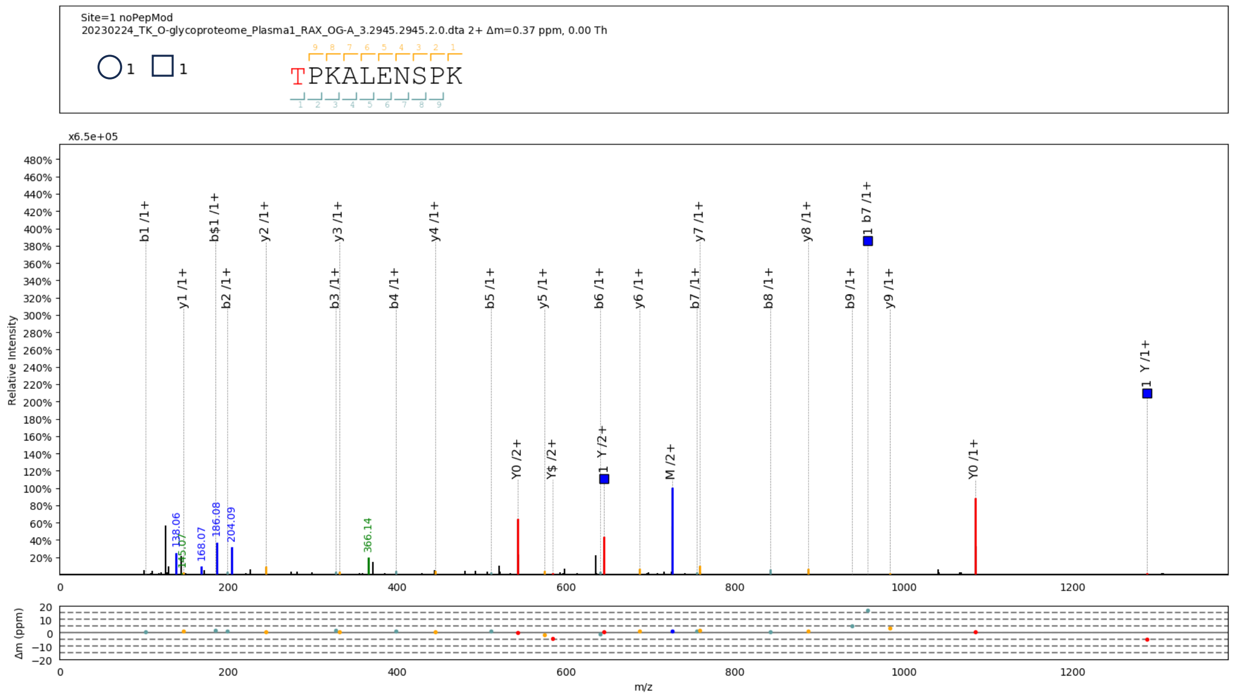

PRG4−T885, N (1)

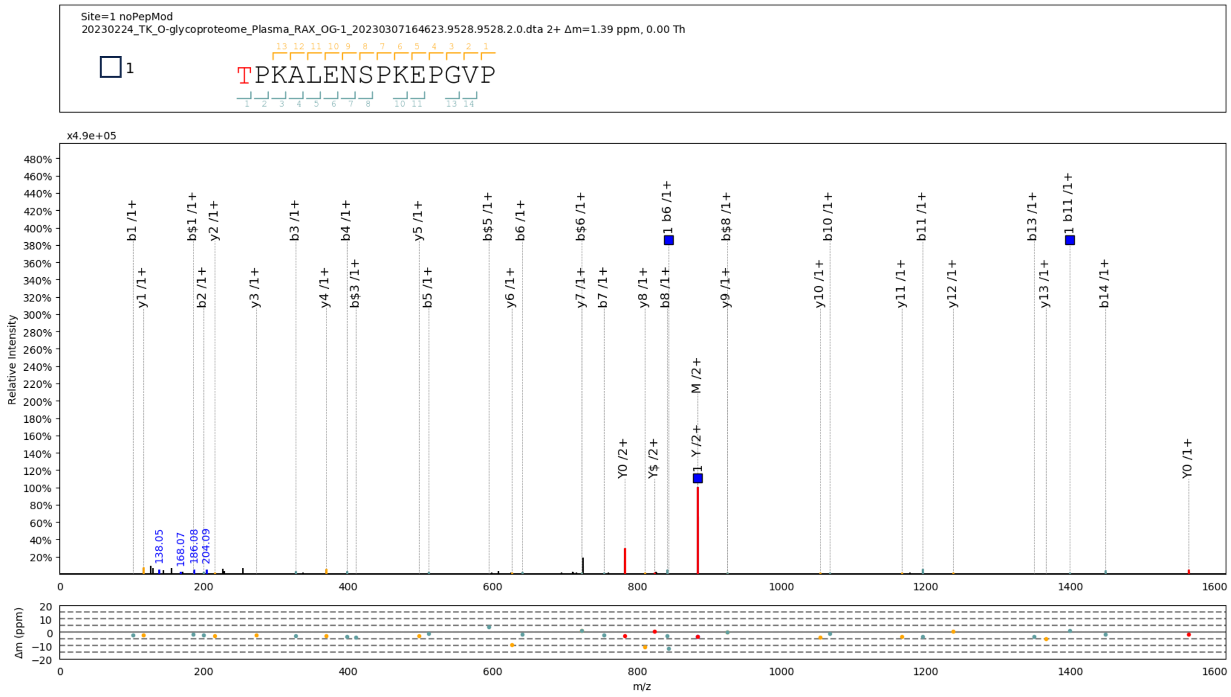

PRG4−T900, H (1) N (1) A (1)

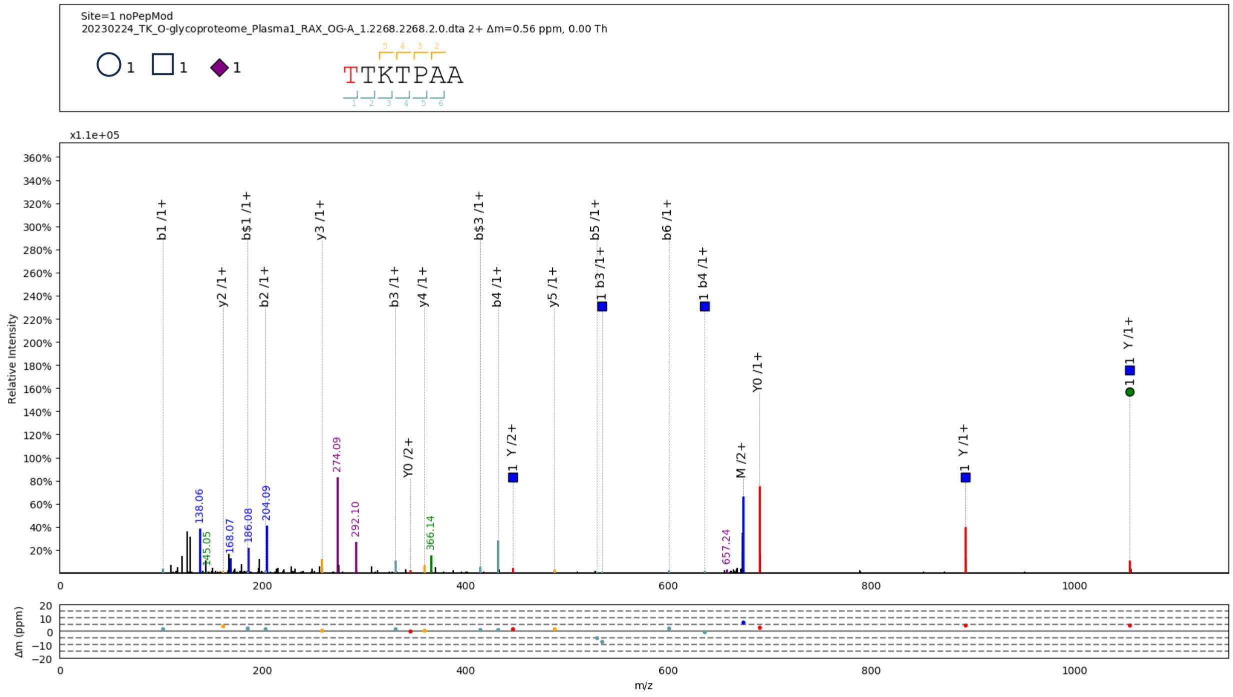

PRG4–T930, H (1) N (1) A (1)

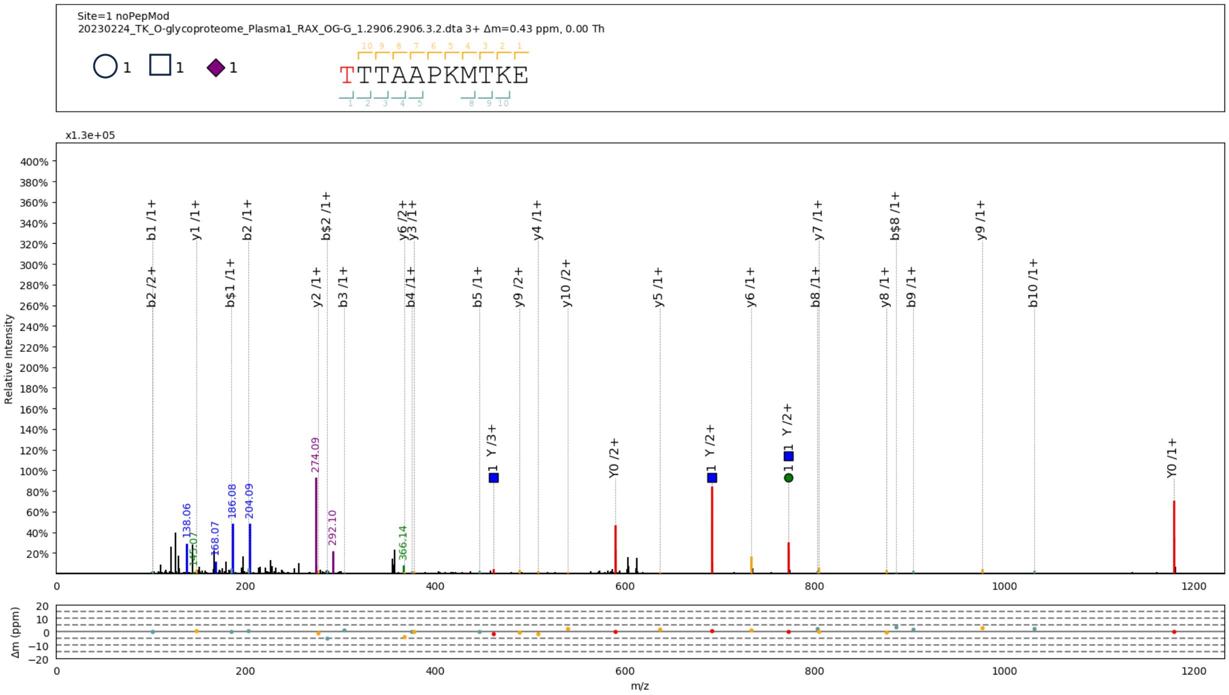

PRG4–T991, H (2) N (2) A (2)

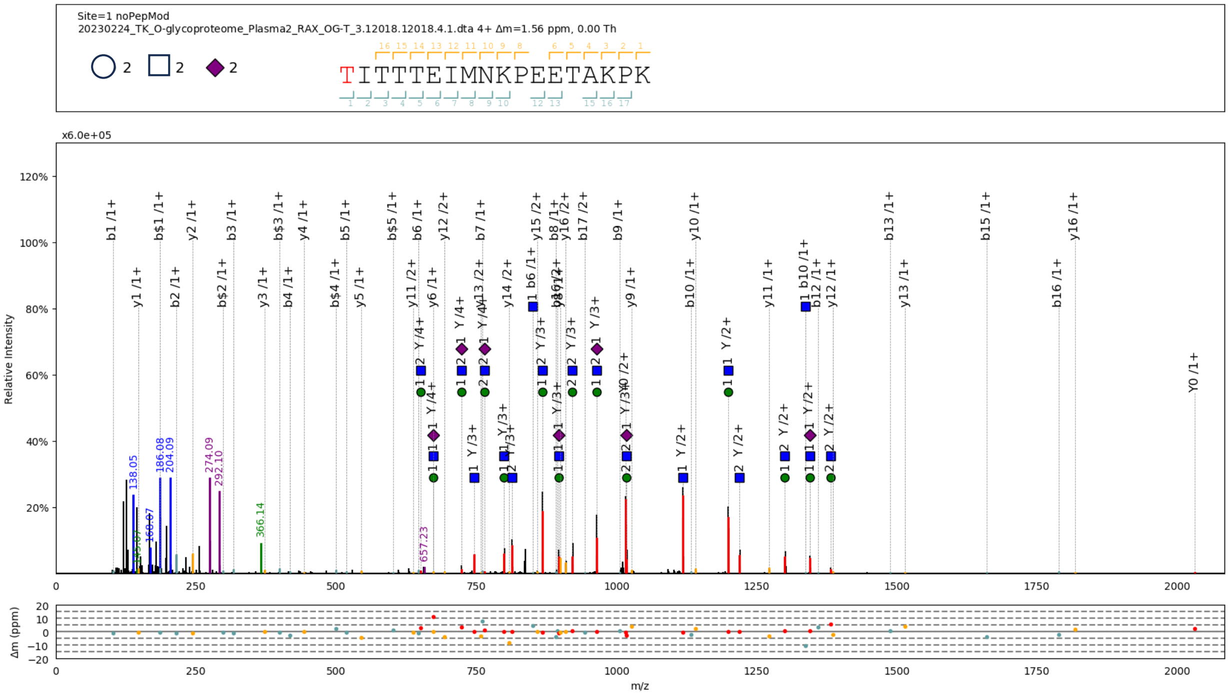

PRG4−T991, H (1) N (1) A (1)

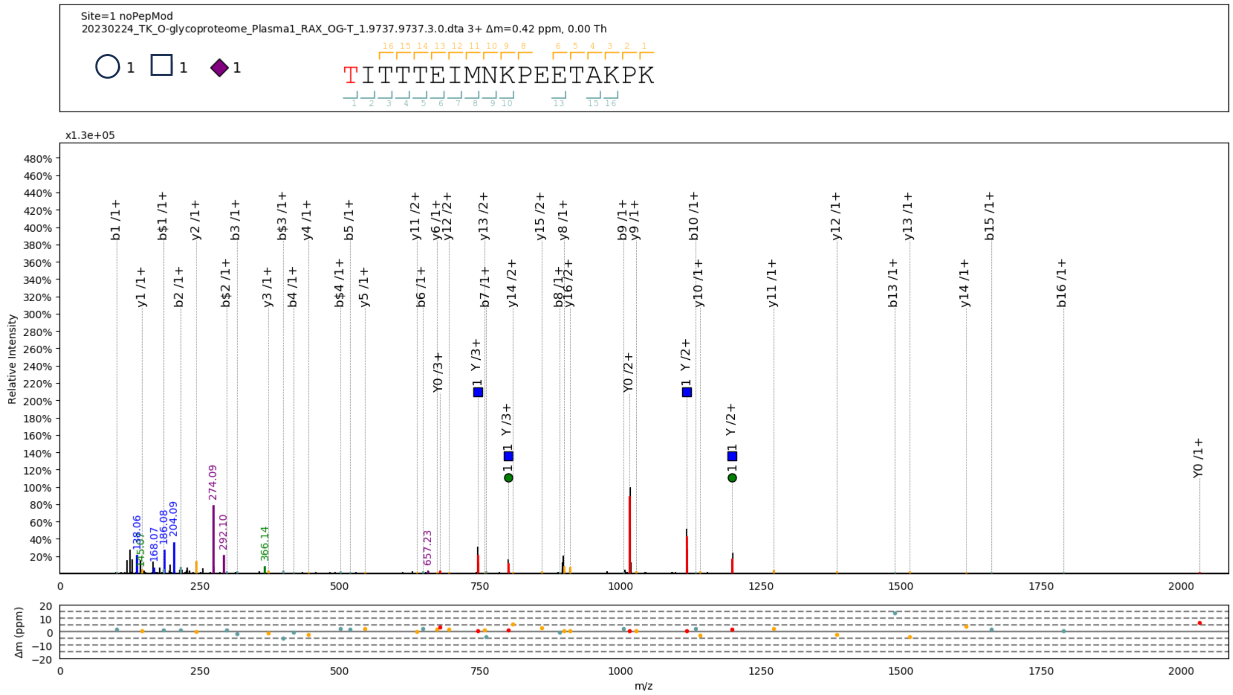

PRG4−T991, H (2) N (2) A (1)

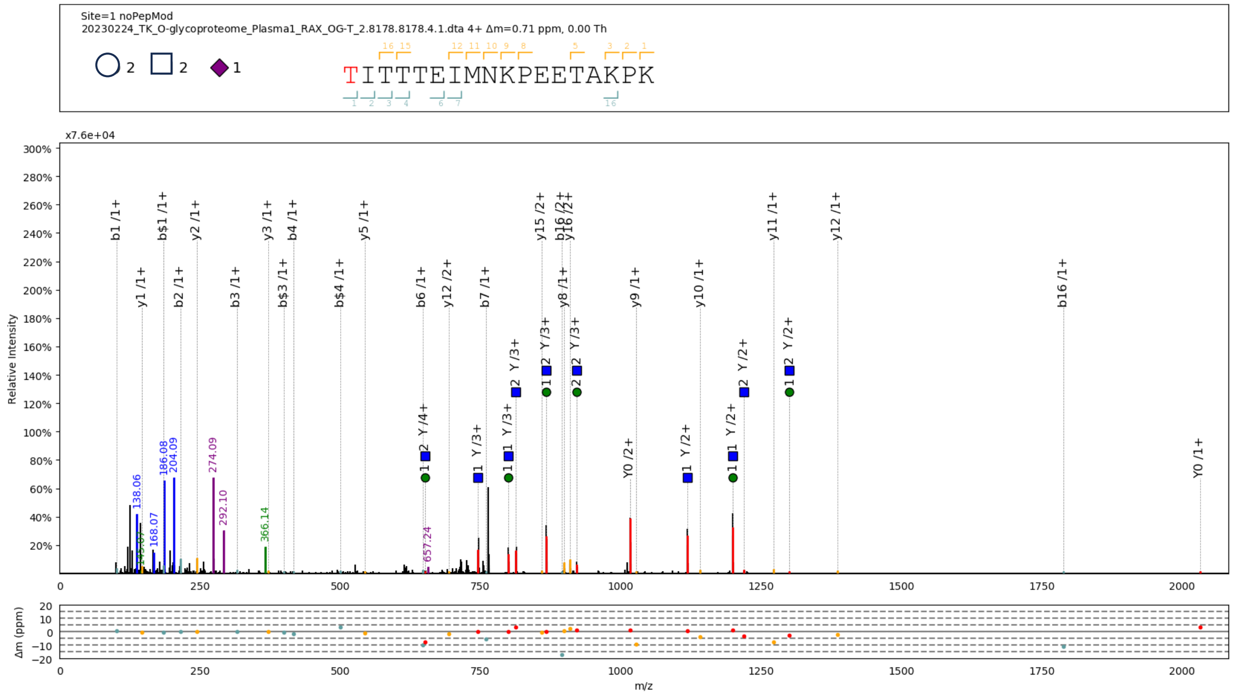

PRG4–T1025, H (1) N (1) A (1)

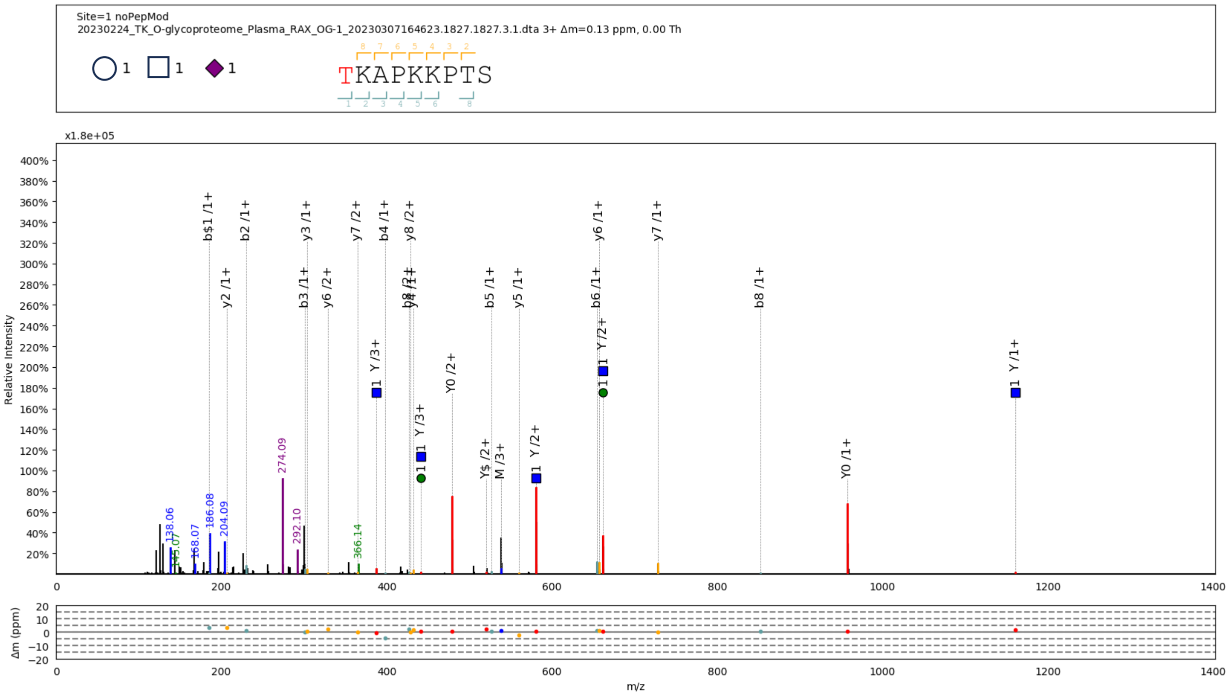

PRG4–T1082, H (1) N (1) A (1)

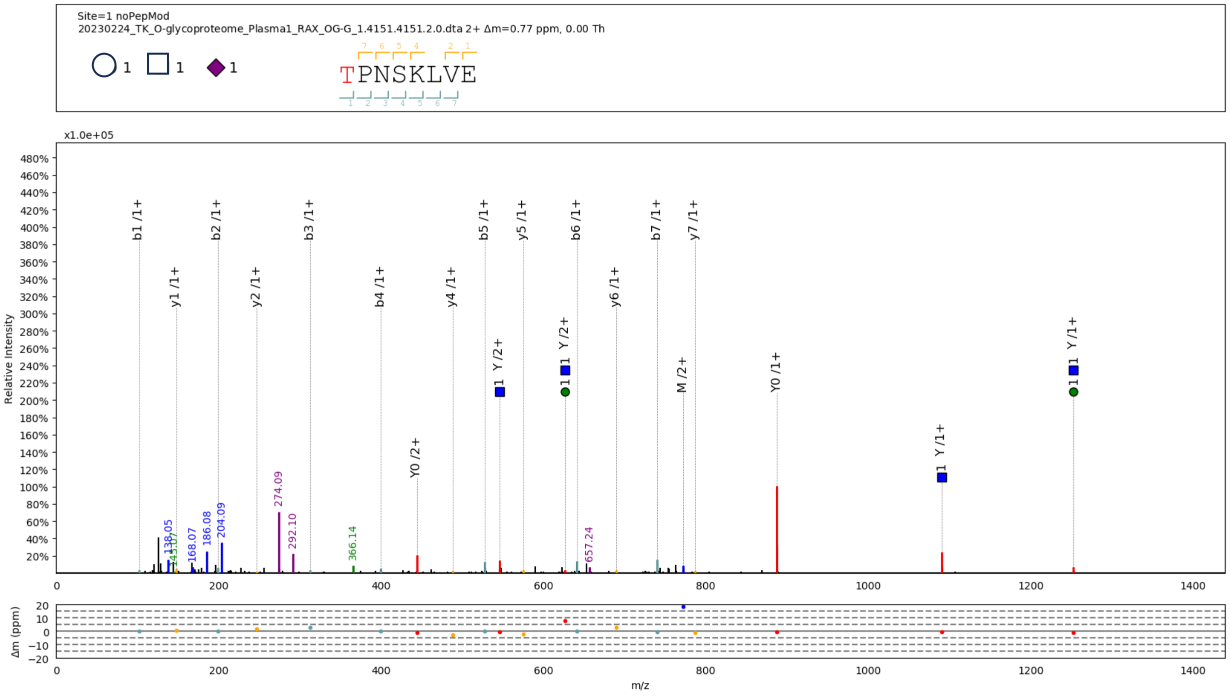

Data S2. MS/MS spectra derived from five proteins purified from human plasma (kininogen-1, fetuin-A, fibrinogen, apolipoprotein E and plasminogen), related to Figure 5.

(A) Fetuin-A (AHSG)

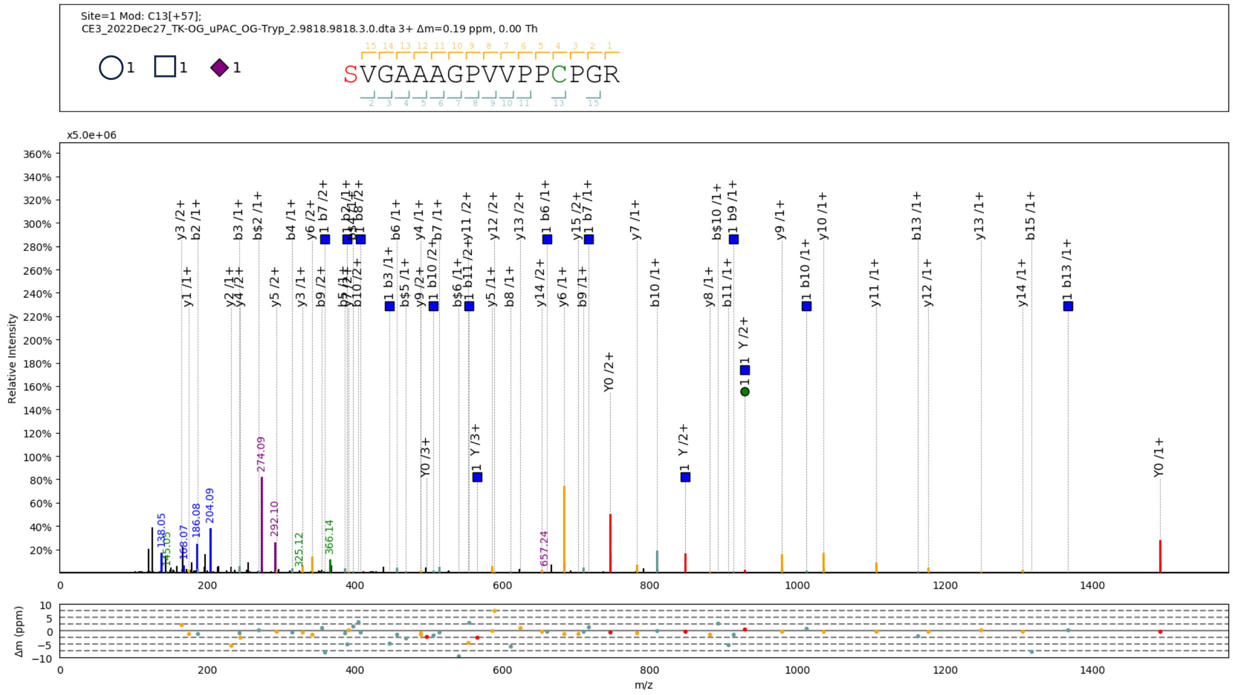

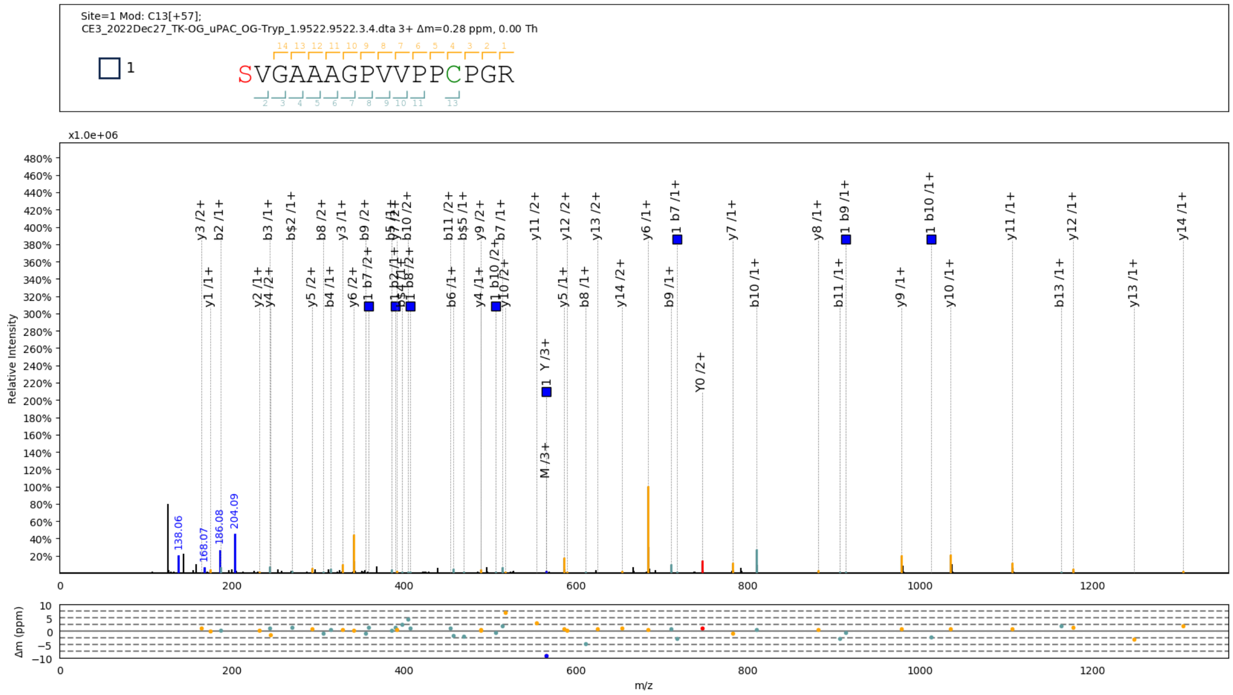

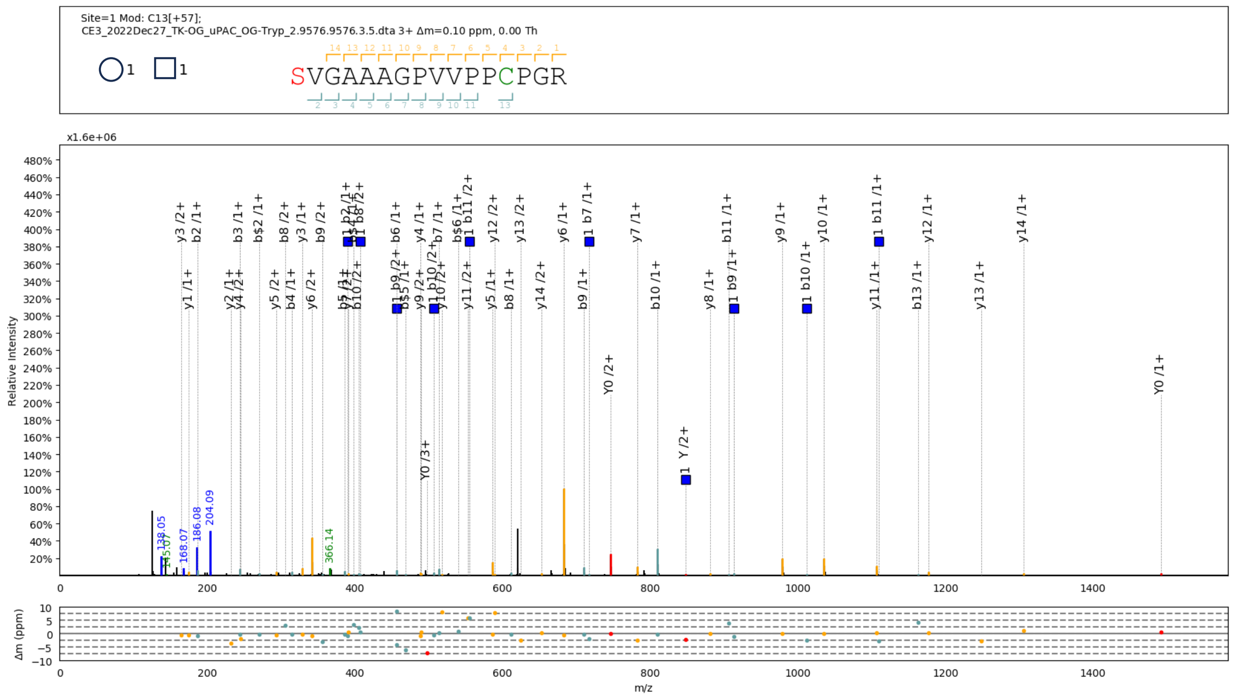
Site=1 Mod: C13[+57];
CE3_2022Dec27_TK-OG_uPAC_OG-Tryp_2.9576.9576.3.5.dta 3+ Δm=0.10 ppm, 0.00 Th
SVGAAAGPVVPPCPGR
x1.6e+06
Relative Intensity
Δm (ppm)
m/z

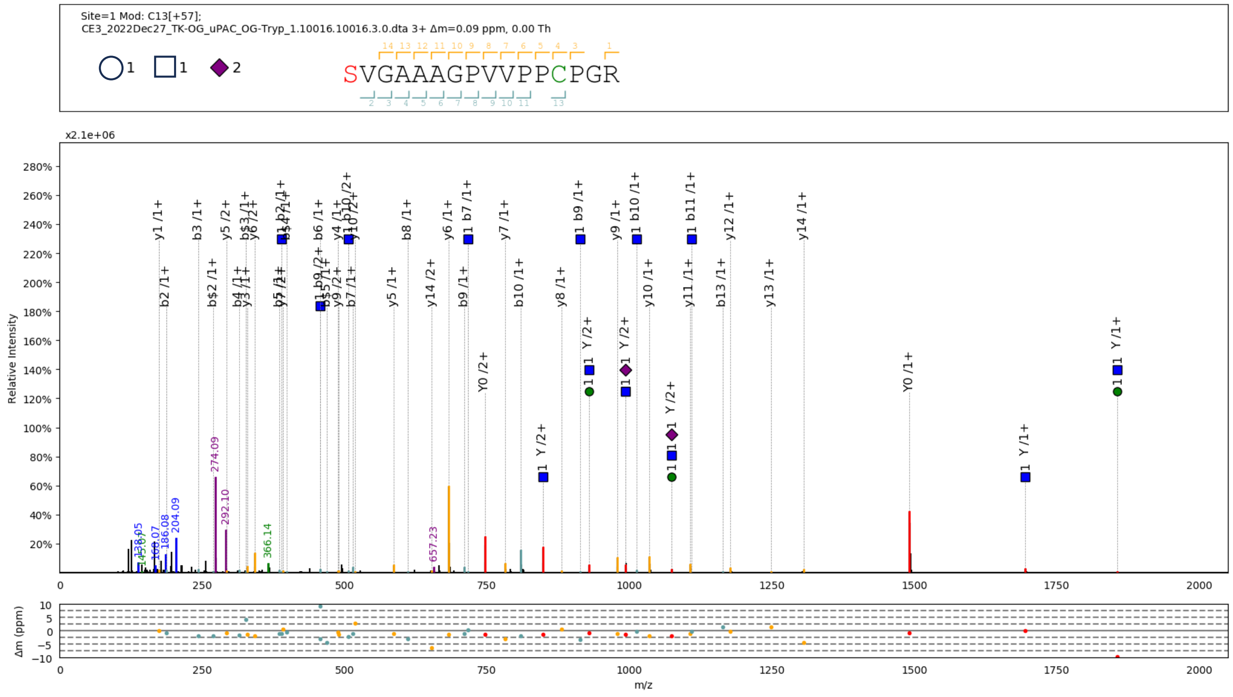
Site=1 Mod: C13[+57];
CE3_2022Dec27_TK-OG_uPAC_OG-Tryp_1.10016.10016.3.0.dta 3+ Δm=0.09 ppm, 0.00 Th
SVGAAAGPVVPPCPGR
x2.1e+06
Relative Intensity
Δm (ppm)
m/z

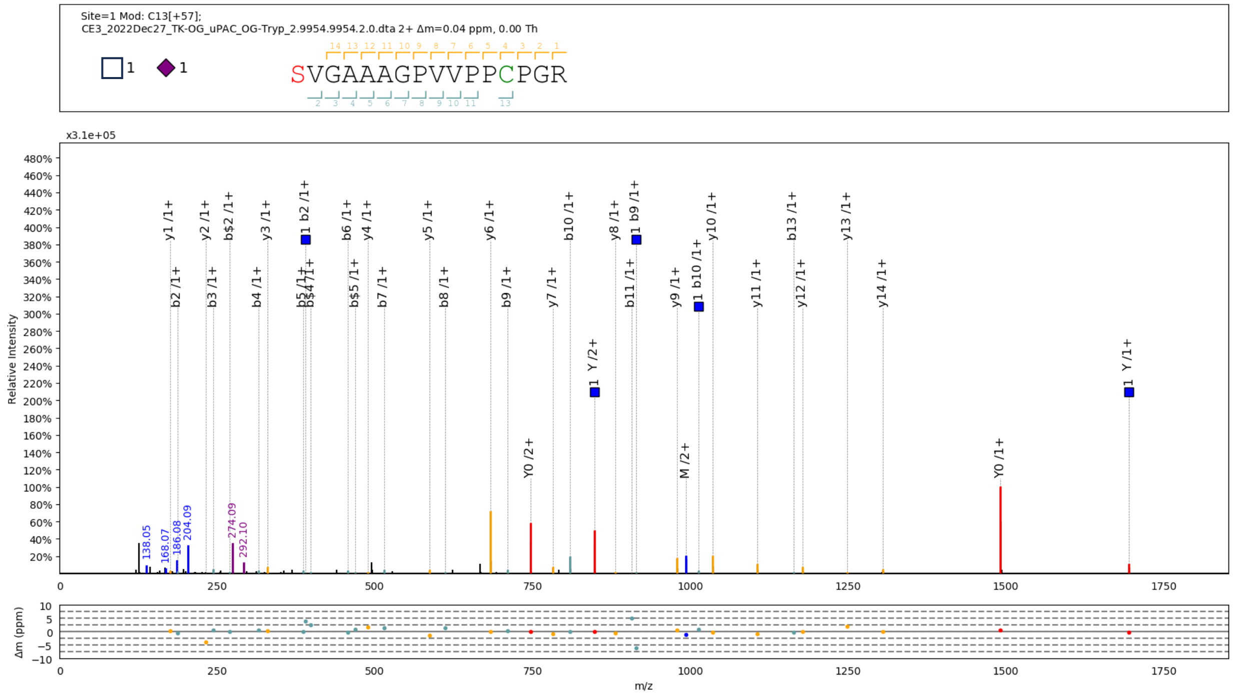
Site=1 Mod: C13[+57];
CE3_2022Dec27_TK-OG_uPAC_OG-Tryp_2.9954.9954.2.0.dta 2+ Δm=0.04 ppm, 0.00 Th
SVGAAAGPVVPPCPGR
x3.1e+05
Relative Intensity
y1 /1+
b2 /1+
y2 /1+
b3 /1+
b$2 /1+
b4 /1+
y3 /1+
1 b2 /1+
b5 /1+
b$4 /1+
b6 /1+
b$5 /1+
y4 /1+
b7 /1+
y5 /1+
b8 /1+
y6 /1+
b9 /1+
Y0 /2+
y7 /1+
b10 /1+
1 Y /2+
y8 /1+
b11 /1+
1 b9 /1+
y9 /1+
M /2+
1 b10 /1+
y10 /1+
y11 /1+
b13 /1+
y12 /1+
y13 /1+
y14 /1+
Y0 /1+
1 Y /1+
138.05
168.07
186.08
204.09
274.09
292.10
Δm (ppm)
m/z

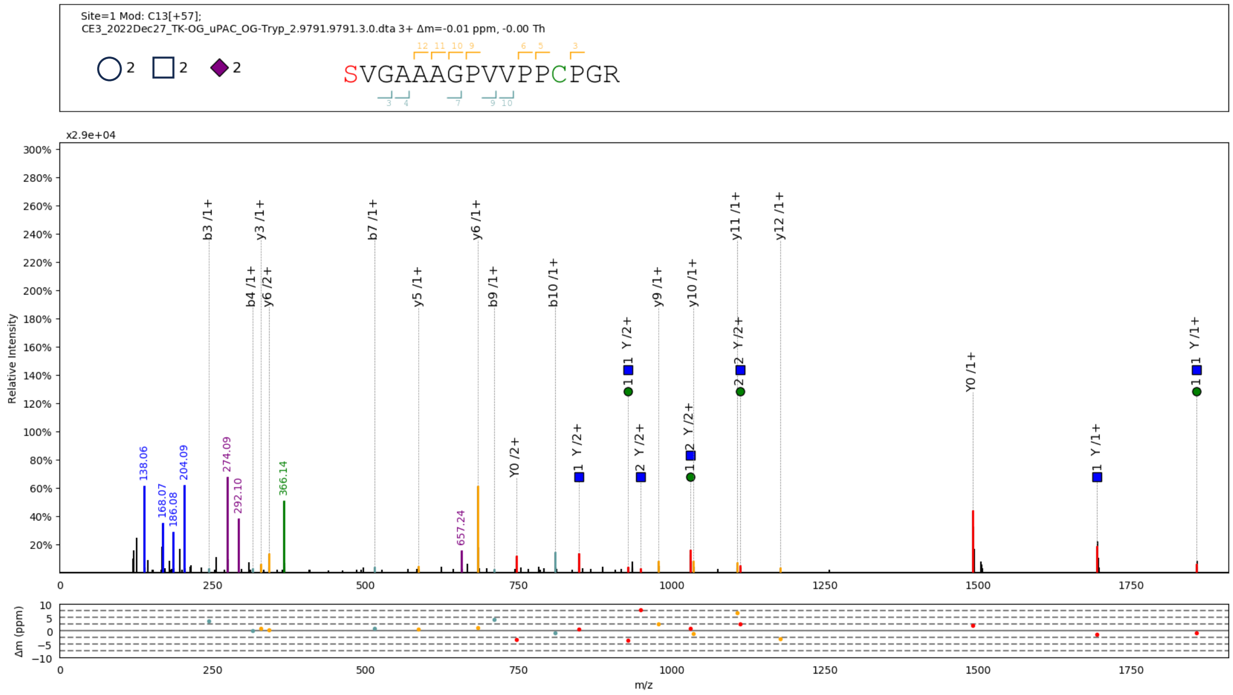
Site=1 Mod: C13[+57];
CE3_2022Dec27_TK-OG_uPAC_OG-Tryp_2.9791.9791.3.0.dta 3+ Δm=-0.01 ppm, -0.00 Th
SVGAAAGPVVPPCPGR
x2.9e+04
Relative Intensity
b3 /1+
b4 /1+
y3 /1+
y6 /2+
b7 /1+
y5 /1+
y6 /1+
b9 /1+
Y0 /2+
b10 /1+
1 Y /2+
1 1 Y /2+
2 Y /2+
y9 /1+
1 2 Y /2+
y10 /1+
y11 /1+
2 2 Y /2+
y12 /1+
Y0 /1+
1 Y /1+
1 1 Y /1+
138.06
168.07
186.08
204.09
274.09
292.10
366.14
657.24
Δm (ppm)
m/z

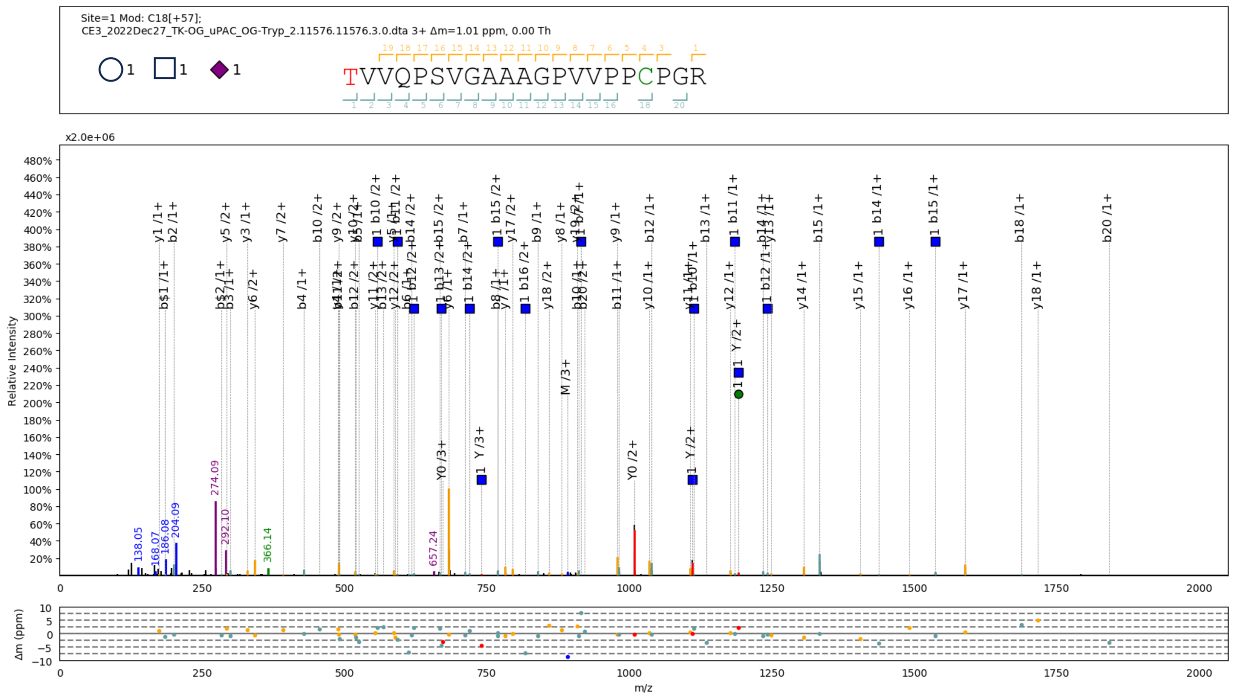
Site=1 Mod: C18[+57];
CE3_2022Dec27_TK-OG_uPAC_OG-Tryp_2.11576.11576.3.0.dta 3+ Δm=1.01 ppm, 0.00 Th
TVVQPSVGAAAGPVVPPCPGR
x2.0e+06
Relative Intensity
Δm (ppm)
m/z

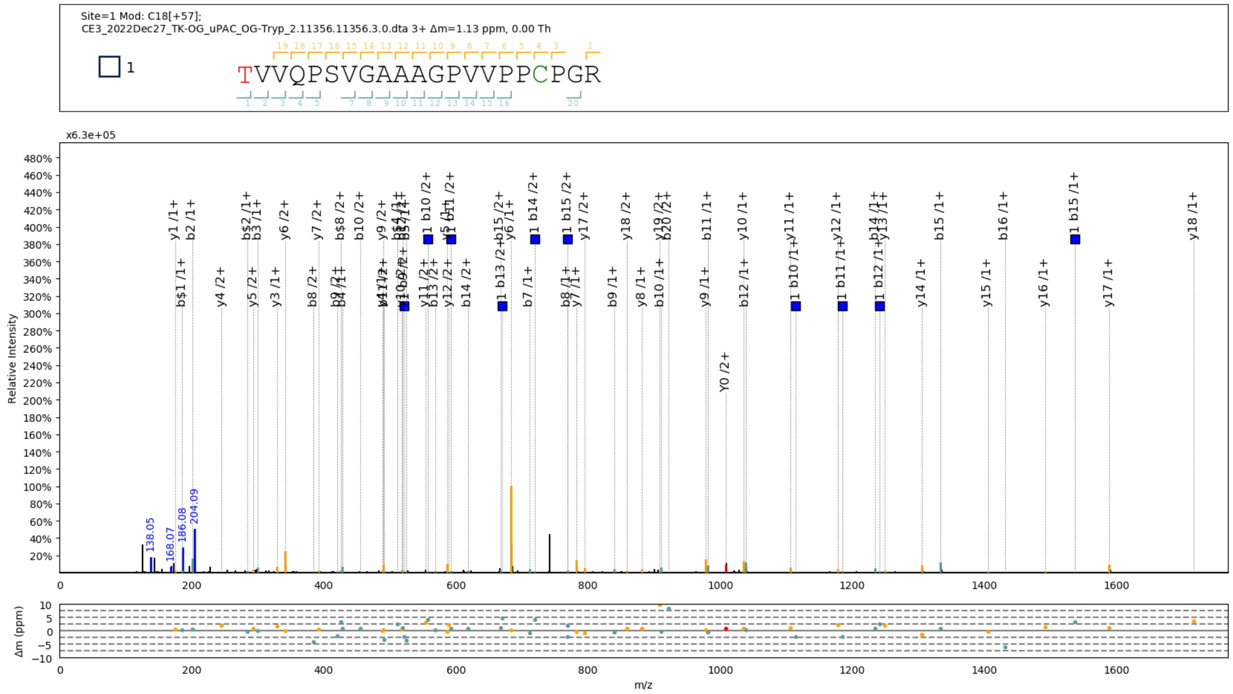
Site=1 Mod: C18[+57];
CE3_2022Dec27_TK-OG_uPAC_OG-Tryp_2.11356.11356.3.0.dta 3+ Δm=1.13 ppm, 0.00 Th
TVVQPSVGAAAGPVVPPCPGR
x6.3e+05
Relative Intensity
Δm (ppm)
m/z

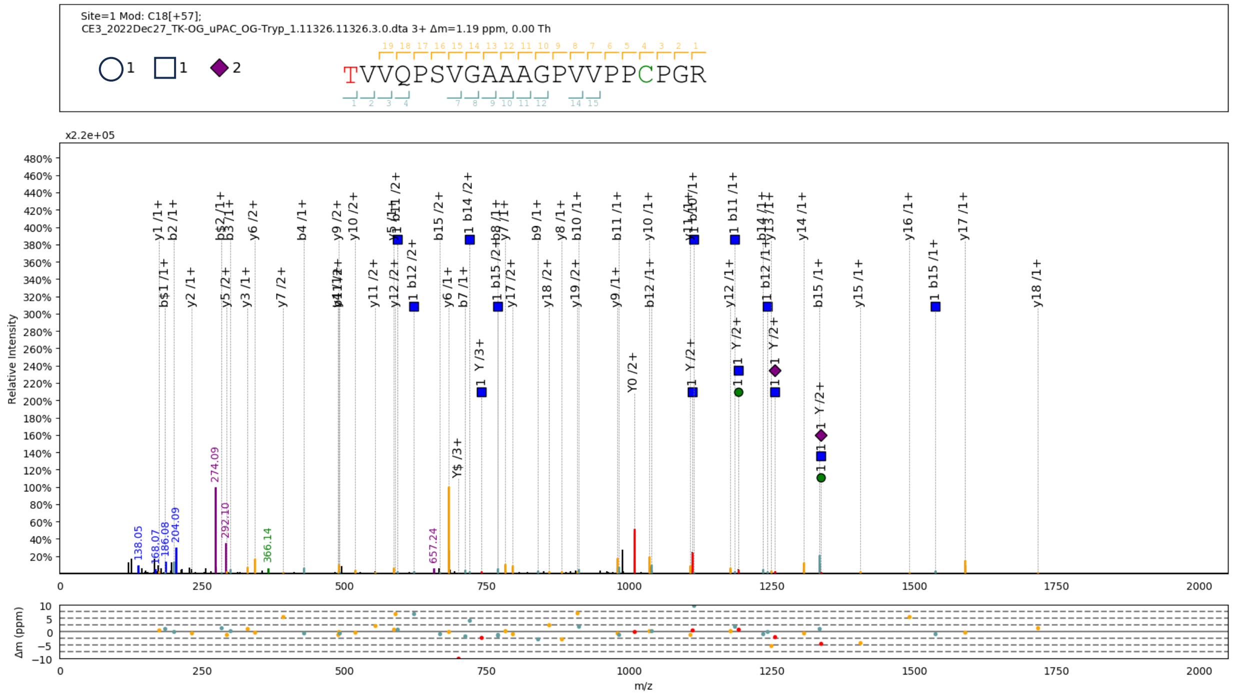
Site=1 Mod: C18[+57];
CE3_2022Dec27_TK-OG_uPAC_OG-Tryp_1.11326.11326.3.0.dta 3+ Δm=1.19 ppm, 0.00 Th
TVVQPSVGAAAGPVVPPCPGR
x2.2e+05
Relative Intensity
Δm (ppm)
m/z

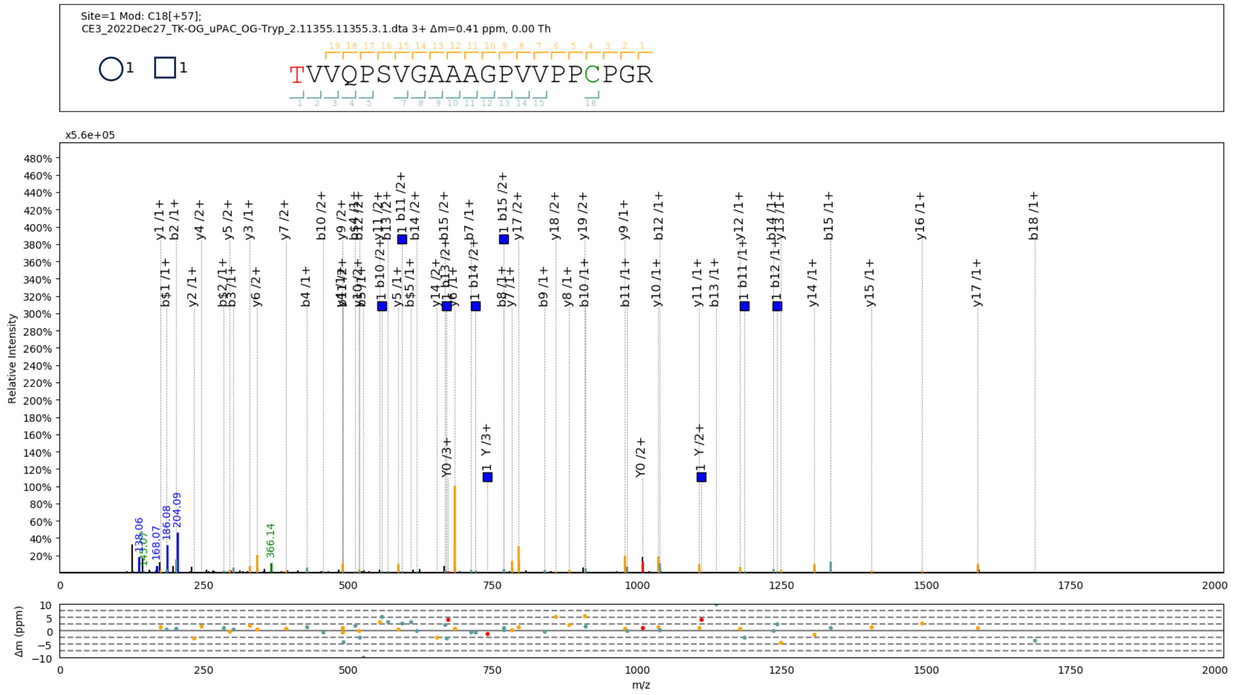
Site=1 Mod: C18[+57];
CE3_2022Dec27_TK-OG_uPAC_OG-Tryp_2.11355.11355.3.1.dta 3+ Δm=0.41 ppm, 0.00 Th
TVVQPSVGAAAGPVVPPCPGR
x5.6e+05
Relative Intensity
Δm (ppm)
m/z

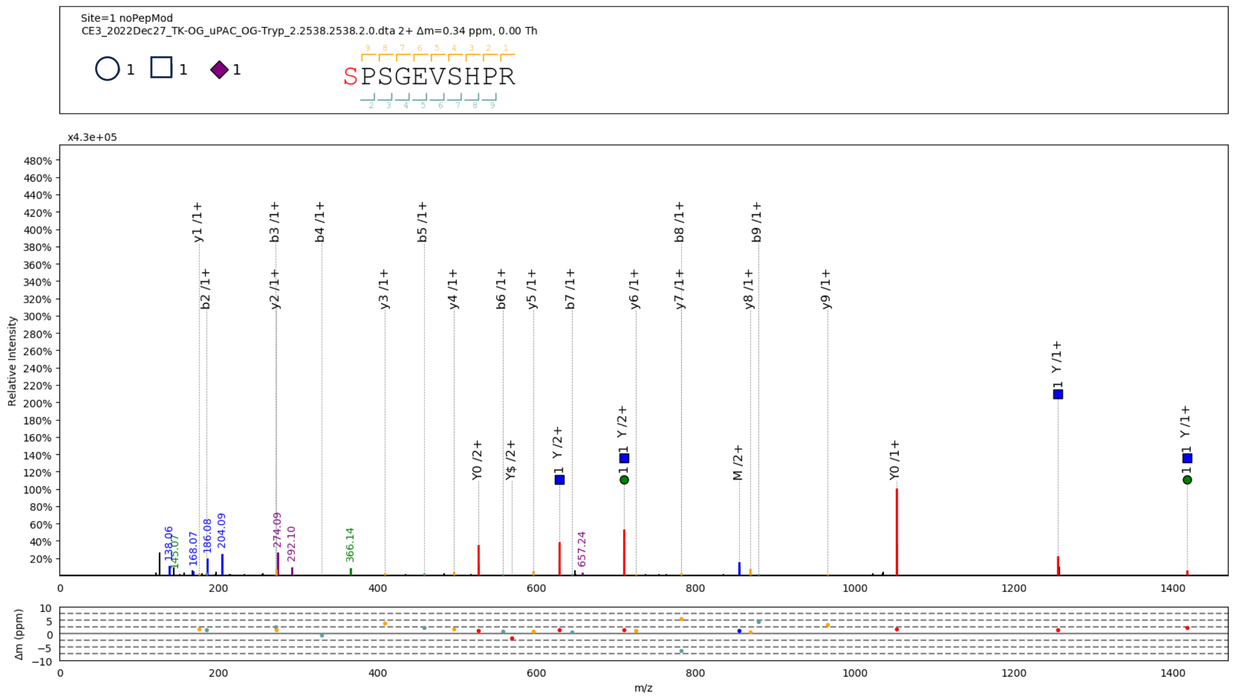

Site=1 noPepMod
CE3_2022Dec27_TK-OG_uPAC_OG-Tryp_2.2538.2538.2.0.dta 2+ Δm=0.34 ppm, 0.00 Th
1 1 1
SPSGEVSHPR
x4.3e+05
Relative Intensity
y1 /1+
b2 /1+
b3 /1+
y2 /1+
b4 /1+
y3 /1+
b5 /1+
y4 /1+
b6 /1+
y5 /1+
b7 /1+
y6 /1+
b8 /1+
y7 /1+
b9 /1+
y8 /1+
y9 /1+
Y0 /2+
Y$ /2+
1 Y /2+
1 1 Y /2+
M /2+
Y0 /1+
1 Y /1+
1 1 Y /1+
138.06
145.07
168.07
186.08
204.09
274.09
292.10
366.14
657.24
Δm (ppm)
m/z

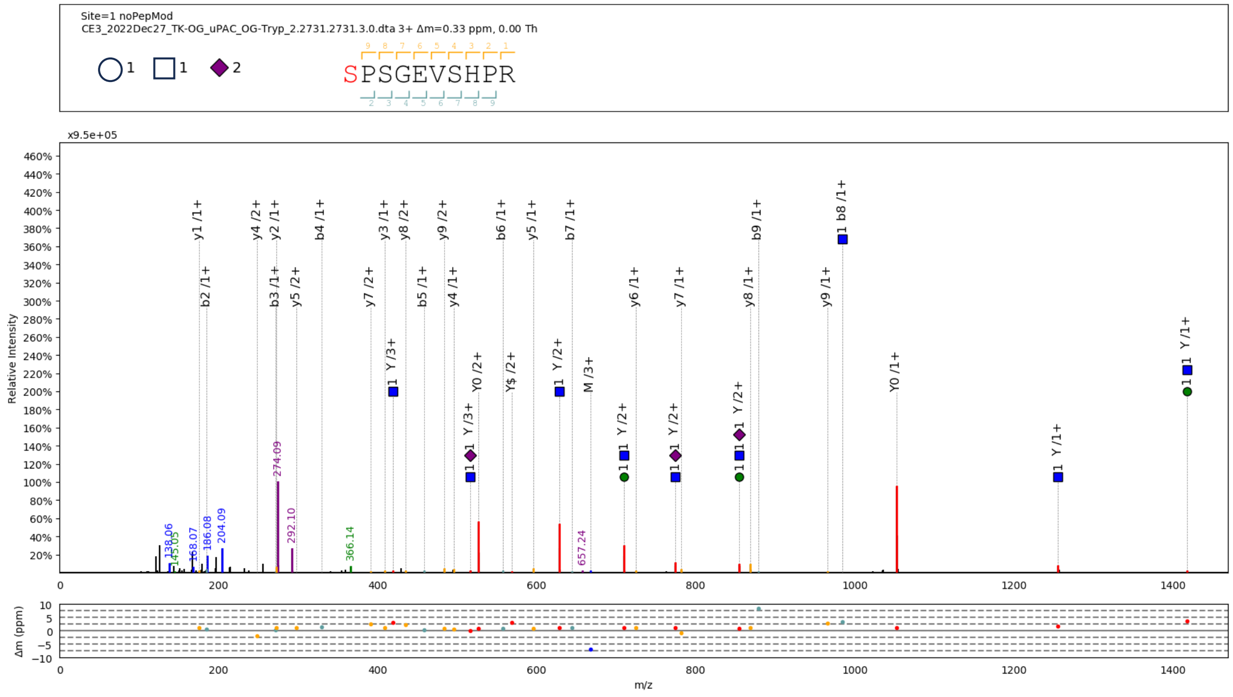

Site=1 noPepMod
CE3_2022Dec27_TK-OG_uPAC_OG-Tryp_2.2731.2731.3.0.dta 3+ Δm=0.33 ppm, 0.00 Th
1 1 2
SPSGEVSHPR
x9.5e+05
Relative Intensity
y1 /1+
b2 /1+
y4 /2+
y2 /1+
b3 /1+
y5 /2+
b4 /1+
y7 /2+
y3 /1+
y8 /2+
b5 /1+
y9 /2+
y4 /1+
b6 /1+
y5 /1+
b7 /1+
y6 /1+
y7 /1+
b9 /1+
y8 /1+
y9 /1+
1 b8 /1+
1 Y /3+
1 1 Y /3+
Y0 /2+
Y$ /2+
1 Y /2+
M /3+
1 1 Y /2+
1 1 Y /2+
1 1 Y /2+
Y0 /1+
1 Y /1+
1 1 Y /1+
138.06
145.07
168.07
186.08
204.09
274.09
292.10
366.14
657.24
Δm (ppm)
m/z

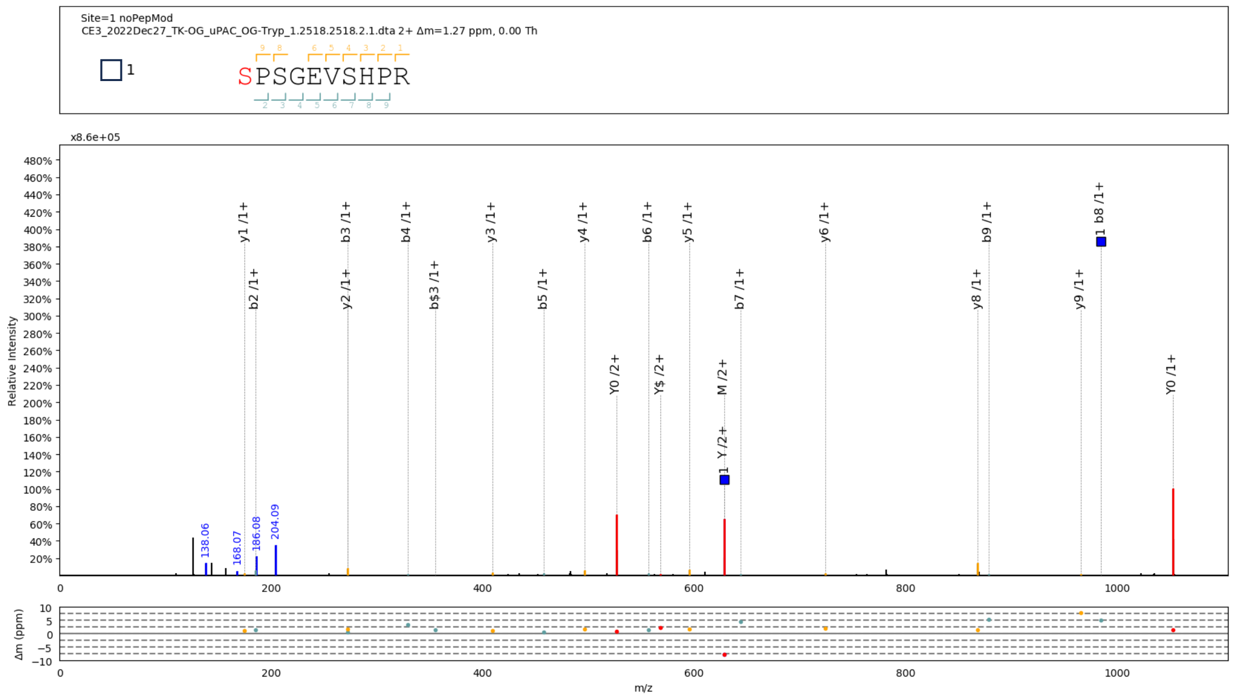
Site=1 noPepMod
CE3_2022Dec27_TK-OG_uPAC_OG-Tryp_1.2518.2518.2.1.dta 2+ Δm=1.27 ppm, 0.00 Th
SPSGEVSHPR
x8.6e+05
Relative Intensity
y1 /1+
b2 /1+
b3 /1+
y2 /1+
b4 /1+
b$3 /1+
y3 /1+
b5 /1+
y4 /1+
Y0 /2+
b6 /1+
Y$ /2+
y5 /1+
M /2+
1 Y /2+
b7 /1+
y6 /1+
y8 /1+
b9 /1+
y9 /1+
1 b8 /1+
Y0 /1+
138.06
168.07
186.08
204.09
Δm (ppm)
m/z

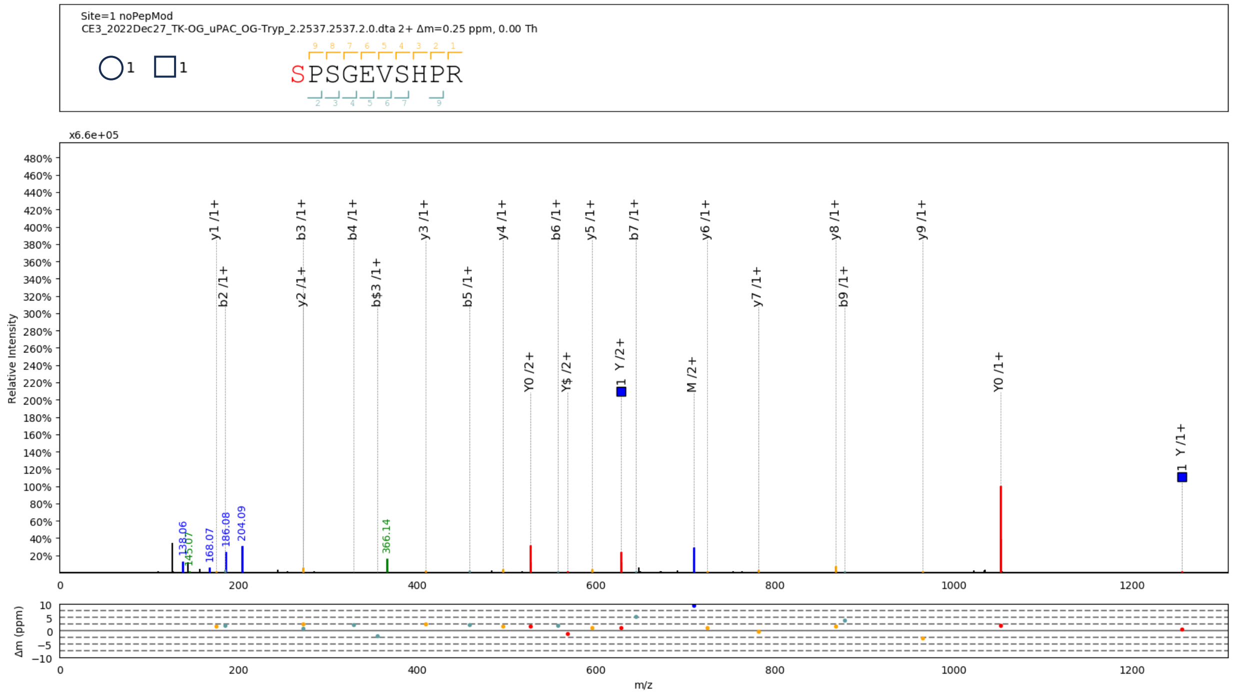
Site=1 noPepMod
CE3_2022Dec27_TK-OG_uPAC_OG-Tryp_2.2537.2537.2.0.dta 2+ Δm=0.25 ppm, 0.00 Th
SPSGEVSHPR
x6.6e+05
Relative Intensity
y1 /1+
b2 /1+
b3 /1+
y2 /1+
b4 /1+
b$3 /1+
y3 /1+
b5 /1+
y4 /1+
Y0 /2+
b6 /1+
Y$ /2+
y5 /1+
1 Y /2+
b7 /1+
M /2+
y6 /1+
y7 /1+
y8 /1+
b9 /1+
y9 /1+
Y0 /1+
1 Y /1+
138.06
168.07
186.08
204.09
366.14
Δm (ppm)
m/z

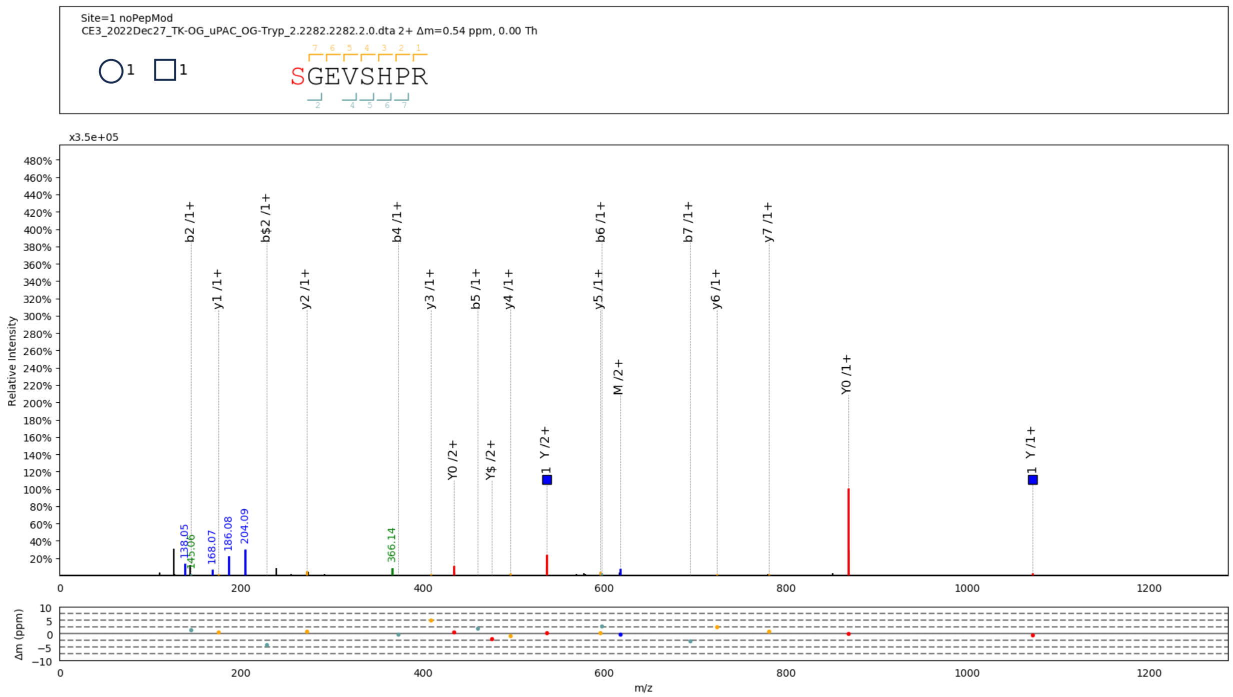
Site=1 noPepMod
CE3_2022Dec27_TK-OG_uPAC_OG-Tryp_2.2282.2282.2.0.dta 2+ Δm=0.54 ppm, 0.00 Th
1
1
SGEVSHPR
x3.5e+05
Relative Intensity
b2 /1+
y1 /1+
b$2 /1+
y2 /1+
b4 /1+
y3 /1+
b5 /1+
y4 /1+
Y0 /2+
Y$ /2+
1 Y /2+
b6 /1+
y5 /1+
M /2+
b7 /1+
y6 /1+
y7 /1+
Y0 /1+
1 Y /1+
138.05
145.06
168.07
186.08
204.09
366.14
Δm (ppm)
m/z

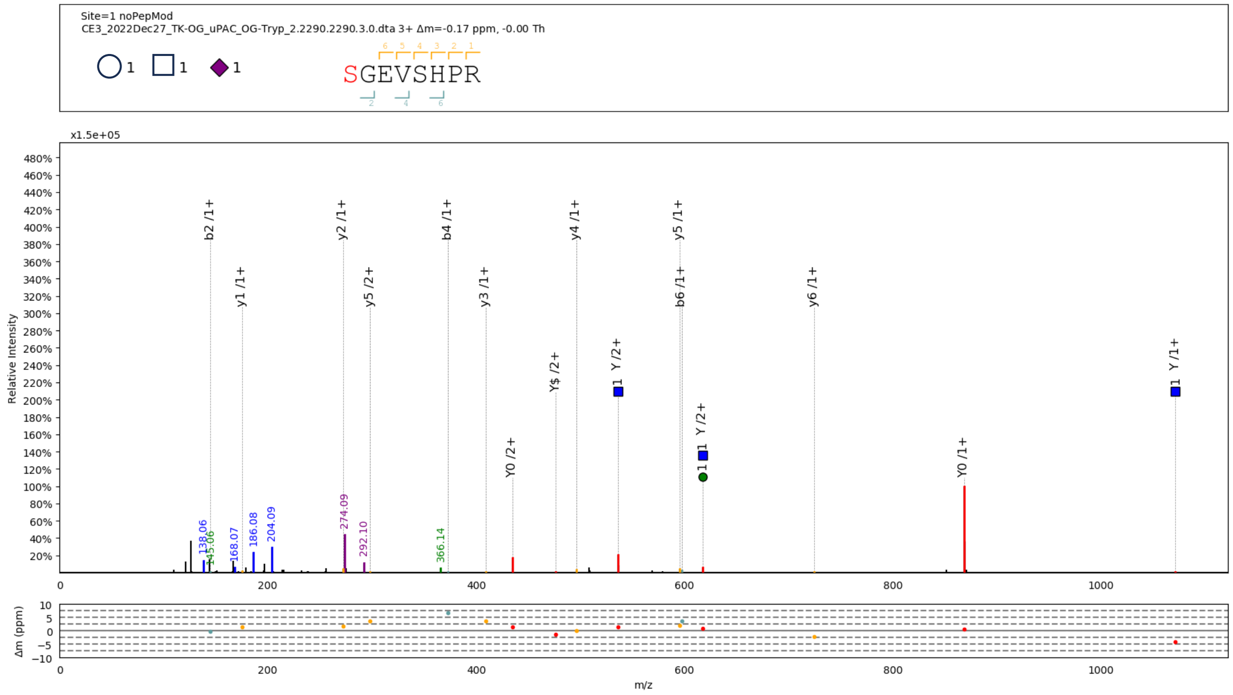
Site=1 noPepMod
CE3_2022Dec27_TK-OG_uPAC_OG-Tryp_2.2290.2290.3.0.dta 3+ Δm=-0.17 ppm, -0.00 Th
1
1
1
SGEVSHPR
x1.5e+05
Relative Intensity
b2 /1+
y1 /1+
y2 /1+
y5 /2+
b4 /1+
y3 /1+
Y$ /2+
y4 /1+
Y0 /2+
1 Y /2+
y5 /1+
b6 /1+
1 Y /2+
y6 /1+
Y0 /1+
1 Y /1+
138.06
145.06
168.07
186.08
204.09
274.09
292.10
366.14
Δm (ppm)
m/z

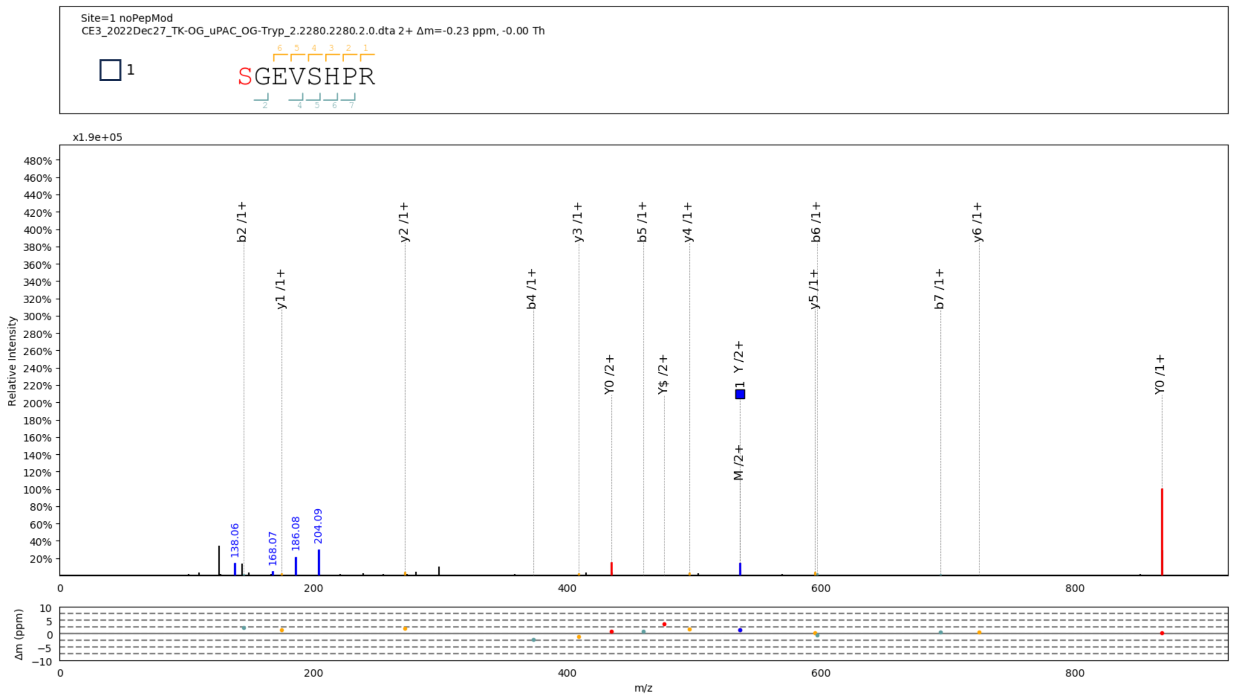

Site=1 noPepMod
CE3_2022Dec27_TK-OG_uPAC_OG-Tryp_2.2280.2280.2.0.dta 2+ Δm=-0.23 ppm, -0.00 Th
1
SGEVSHPR
x1.9e+05
Relative Intensity
b2 /1+
y1 /1+
y2 /1+
b4 /1+
y3 /1+
Y0 /2+
b5 /1+
Y$ /2+
y4 /1+
1 Y /2+
M /2+
b6 /1+
y5 /1+
b7 /1+
y6 /1+
Y0 /1+
138.06
168.07
186.08
204.09
Δm (ppm)
m/z

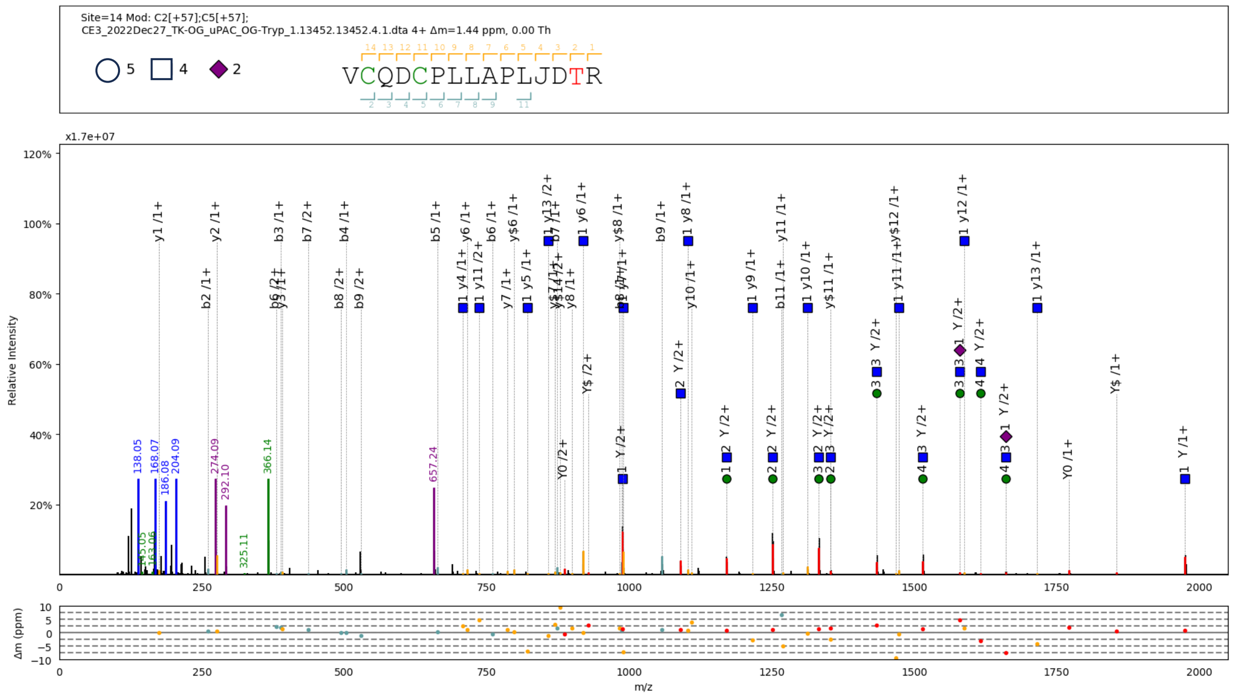

Site=14 Mod: C2[+57];C5[+57];
CE3_2022Dec27_TK-OG_uPAC_OG-Tryp_1.13452.13452.4.1.dta 4+ Δm=1.44 ppm, 0.00 Th
5
4
2
VCQDCPLLAPLJDTR
x1.7e+07
Relative Intensity
y1 /1+
b2 /1+
y2 /1+
b6 /2+
y3 /1+
b3 /1+
b7 /2+
b8 /2+
b4 /1+
b9 /2+
b5 /1+
y4 /1+
y6 /1+
y11 /2+
b6 /1+
y7 /1+
y$6 /1+
y5 /1+
y13 /2+
b7 /1+
y8 /1+
y6 /1+
Y0 /2+
Y$ /2+
y$8 /1+
b9 /1+
y8 /1+
y10 /1+
y9 /1+
b11 /1+
y11 /1+
y10 /1+
y$11 /1+
y11 /1+
y$12 /1+
y12 /1+
y13 /1+
Y0 /1+
Y$ /1+
Y /1+
Y /2+
138.05
168.07
186.08
204.09
274.09
292.10
366.14
325.11
657.24
Δm (ppm)
m/z

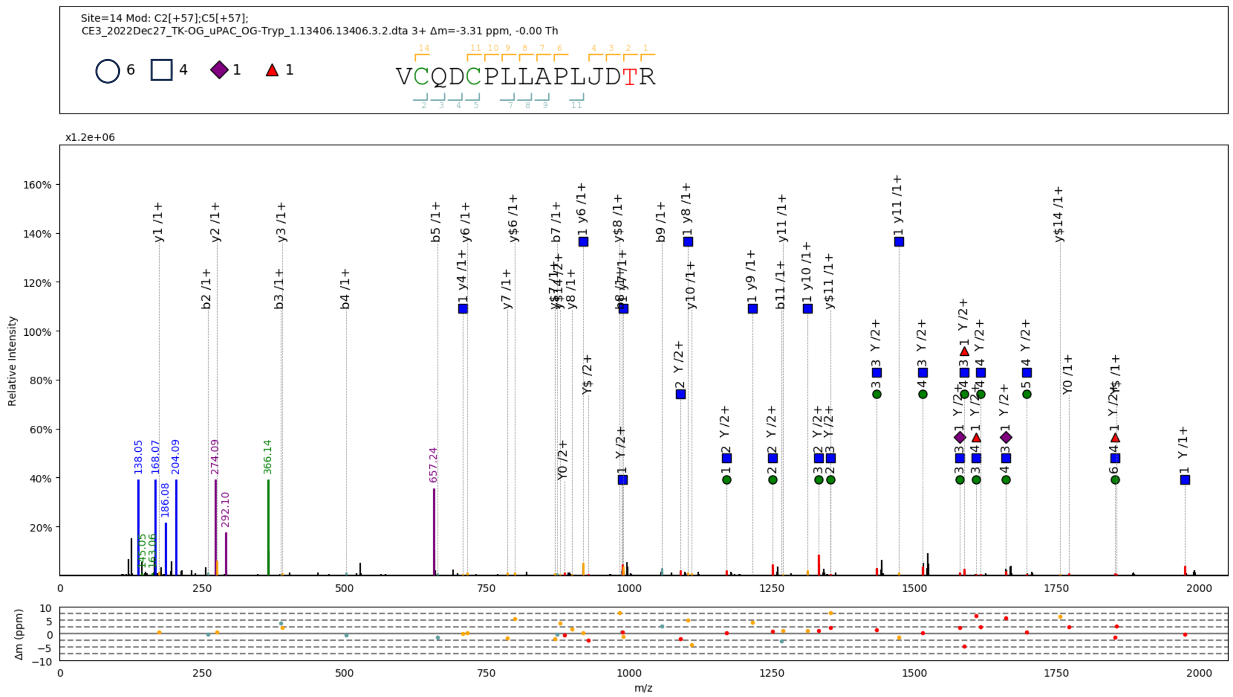
Site=14 Mod: C2[+57];C5[+57];
CE3_2022Dec27_TK-OG_uPAC_OG-Tryp_1.13406.13406.3.2.dta 3+ Δm=-3.31 ppm, -0.00 Th
6
4
1
1
VCQDCPLLAPLJDTR
x1.2e+06
Relative Intensity
m/z
Δm (ppm)

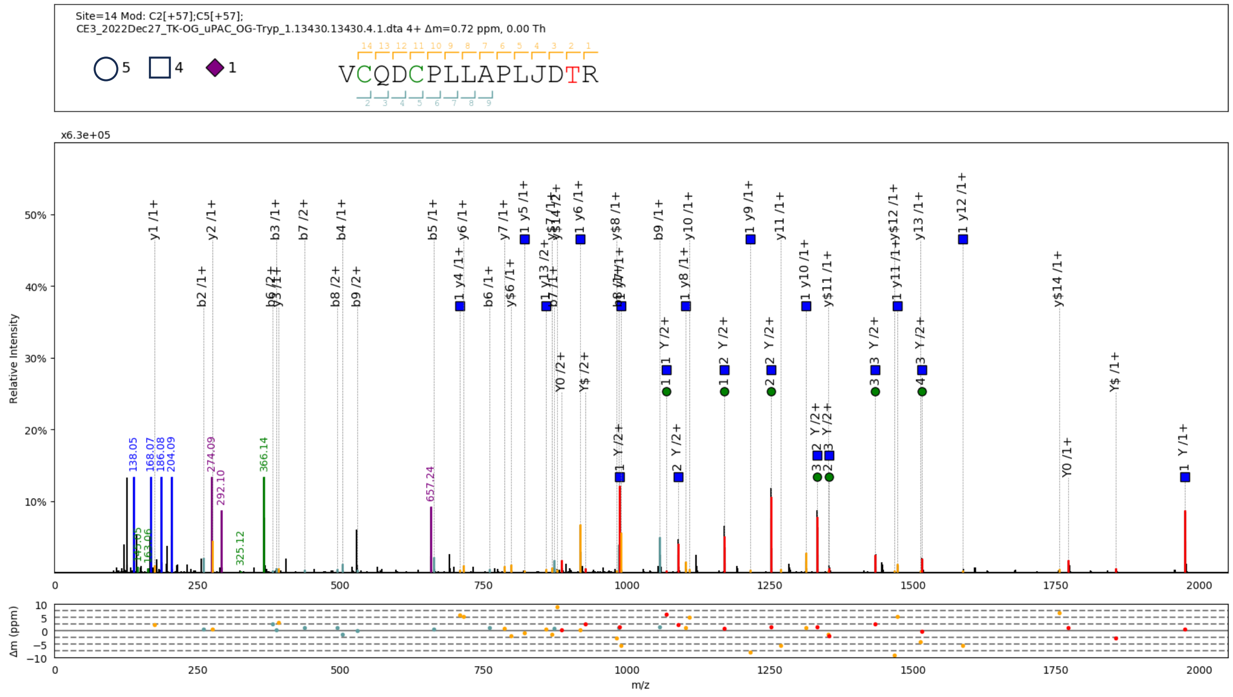
Site=14 Mod: C2[+57];C5[+57];
CE3_2022Dec27_TK-OG_uPAC_OG-Tryp_1.13430.13430.4.1.dta 4+ Δm=0.72 ppm, 0.00 Th
5
4
1
VCQDCPLLAPLJDTR
x6.3e+05
Relative Intensity
m/z
Δm (ppm)

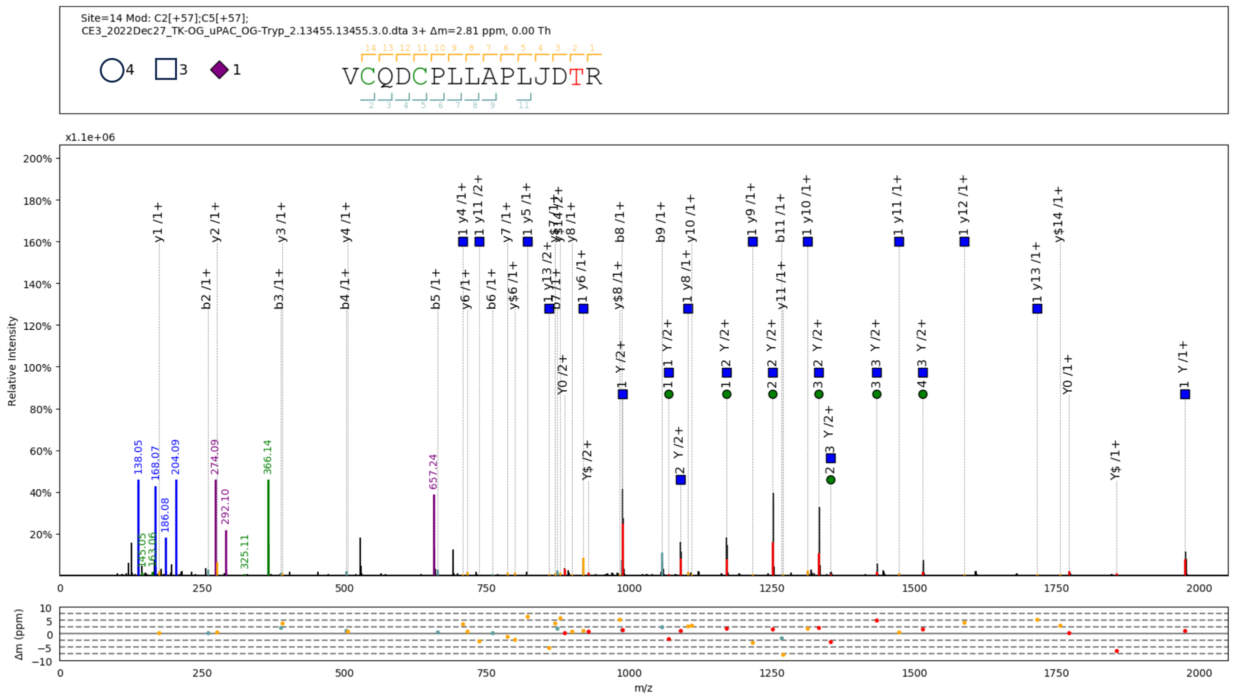
Site=14 Mod: C2[+57];C5[+57];
CE3_2022Dec27_TK-OG_uPAC_OG-Tryp_2.13455.13455.3.0.dta 3+ Δm=2.81 ppm, 0.00 Th
VCQDCPLLAPLJDTR
x1.1e+06
Relative Intensity
Δm (ppm)
m/z

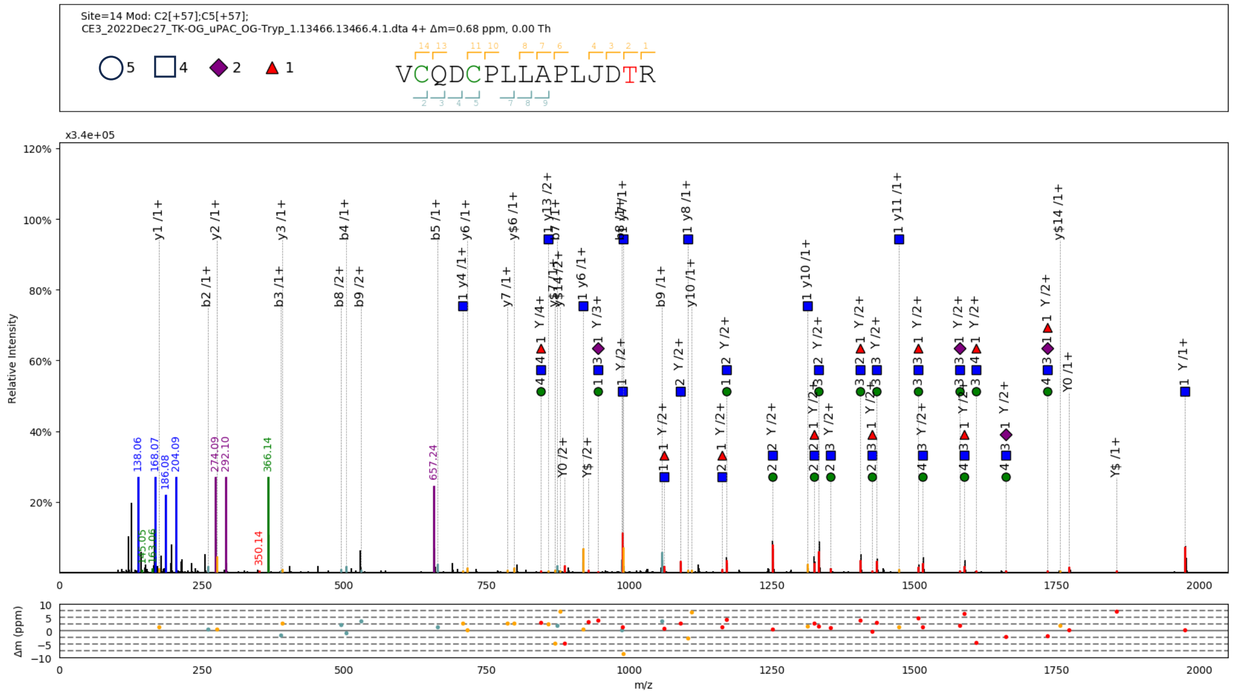
Site=14 Mod: C2[+57];C5[+57];
CE3_2022Dec27_TK-OG_uPAC_OG-Tryp_1.13466.13466.4.1.dta 4+ Δm=0.68 ppm, 0.00 Th
VCQDCPLLAPLJDTR
x3.4e+05
Relative Intensity
Δm (ppm)
m/z

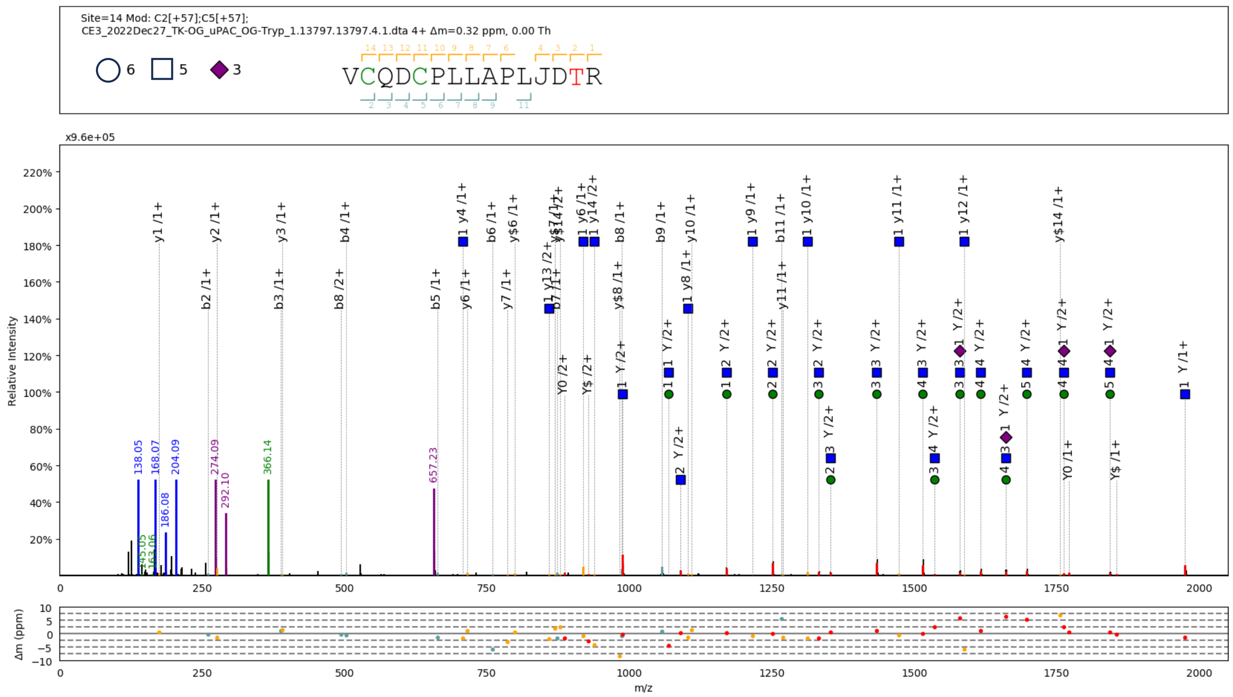
Site=14 Mod: C2[+57];C5[+57];
CE3_2022Dec27_TK-OG_uPAC_OG-Tryp_1.13797.13797.4.1.dta 4+ Δm=0.32 ppm, 0.00 Th
6
5
3
VCQDCPLLAPLJDTR
x9.6e+05
Relative Intensity
Δm (ppm)
m/z

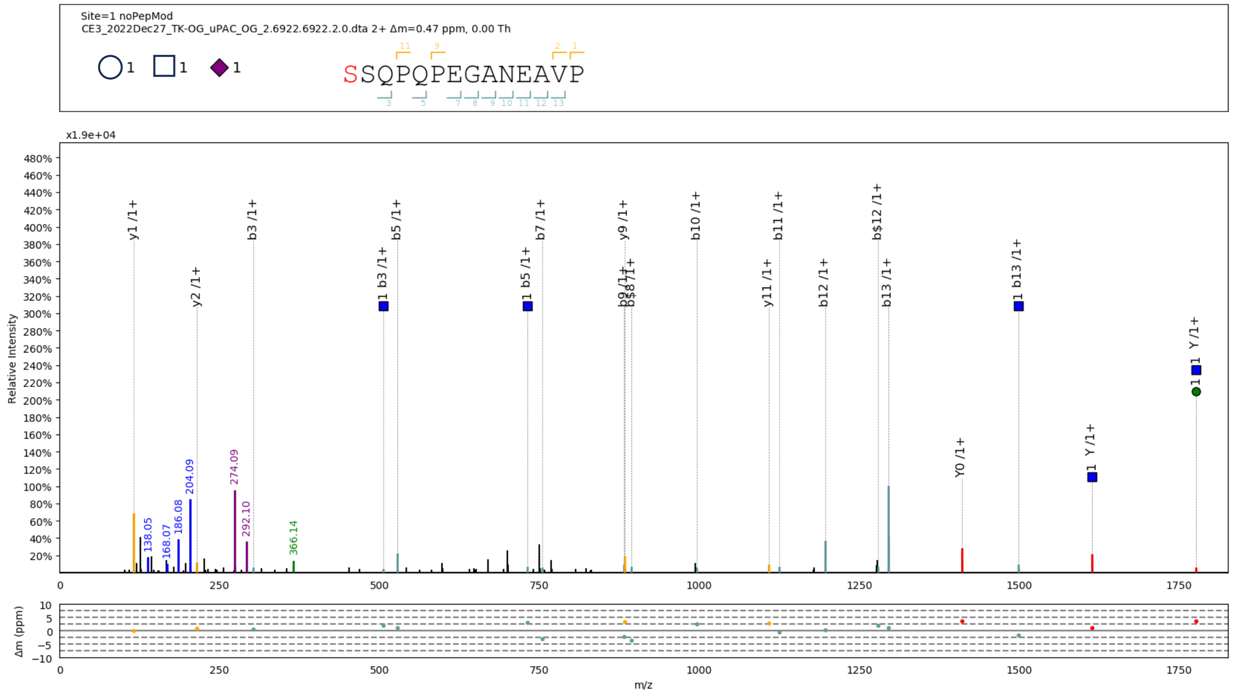
Site=1 noPepMod
CE3_2022Dec27_TK-OG_uPAC_OG_2.6922.6922.2.0.dta 2+ Δm=0.47 ppm, 0.00 Th
1
1
1
SSQPQPEGANEAVP
x1.9e+04
Relative Intensity
Δm (ppm)
m/z

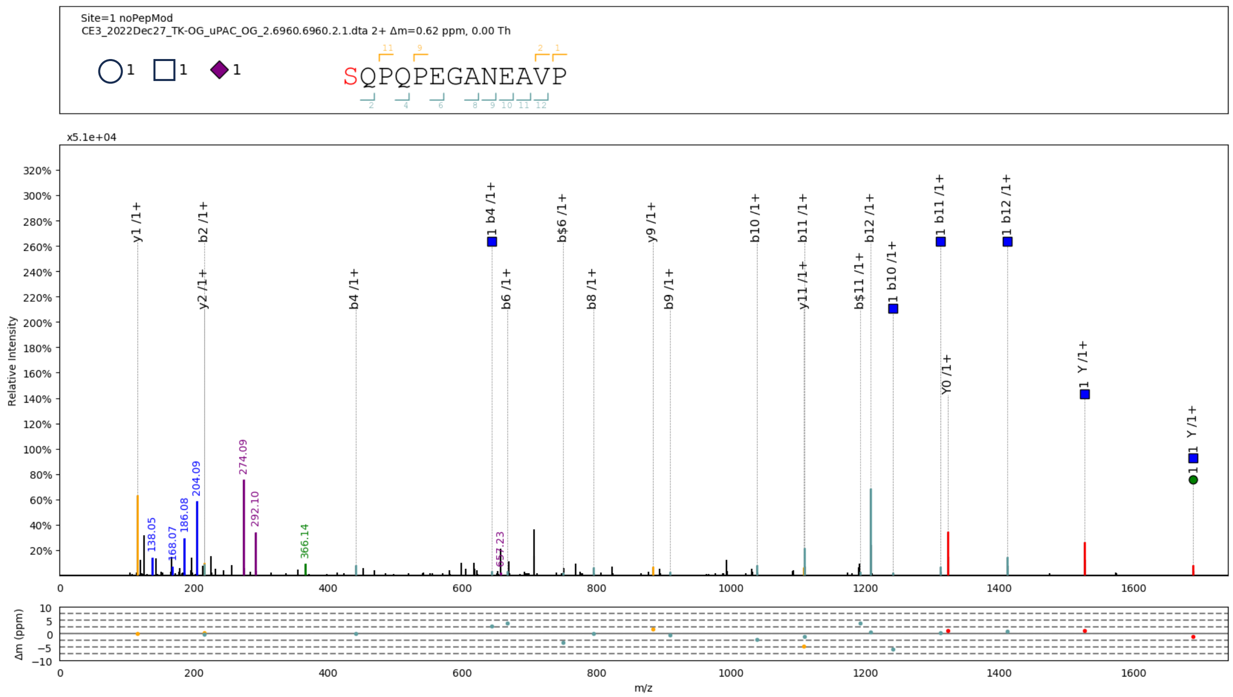
Site=1 noPepMod
CE3_2022Dec27_TK-OG_uPAC_OG_2.6960.6960.2.1.dta 2+ Δm=0.62 ppm, 0.00 Th
SQPQPEGANEAVP
x5.1e+04
Relative Intensity
y1 /1+
b2 /1+
y2 /1+
b4 /1+
b4 /1+
b6 /1+
b$6 /1+
b8 /1+
y9 /1+
b9 /1+
b10 /1+
b11 /1+
y11 /1+
b$11 /1+
b12 /1+
b10 /1+
b11 /1+
b12 /1+
Y0 /1+
Y /1+
Y /1+
138.05
168.07
186.08
204.09
274.09
292.10
366.14
657.23
Δm (ppm)
m/z

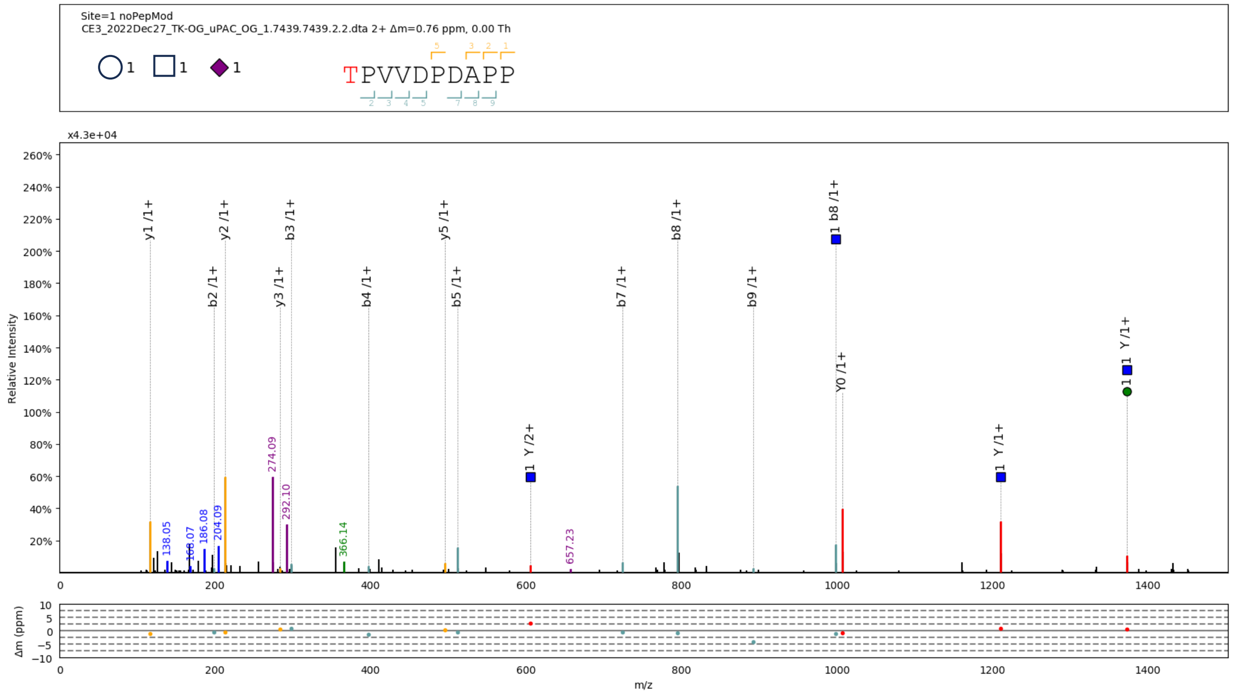
Site=1 noPepMod
CE3_2022Dec27_TK-OG_uPAC_OG_1.7439.7439.2.2.dta 2+ Δm=0.76 ppm, 0.00 Th
TPVVDPDAPP
x4.3e+04
Relative Intensity
y1 /1+
b2 /1+
y2 /1+
y3 /1+
b3 /1+
b4 /1+
y5 /1+
b5 /1+
Y /2+
b7 /1+
b8 /1+
b9 /1+
b8 /1+
Y0 /1+
Y /1+
Y /1+
138.05
168.07
186.08
204.09
274.09
292.10
366.14
657.23
Δm (ppm)
m/z

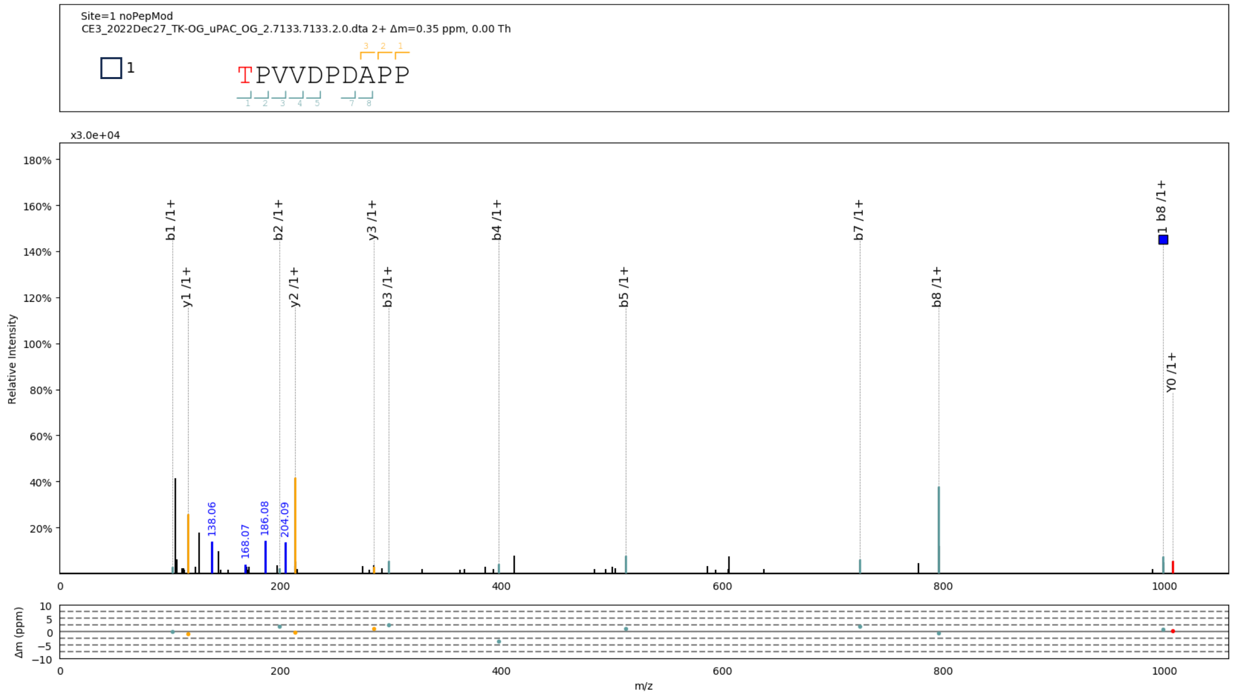
Site=1 noPepMod
CE3_2022Dec27_TK-OG_uPAC_OG_2.7133.7133.2.0.dta 2+ Δm=0.35 ppm, 0.00 Th
1
TPVVDPDAPP
x3.0e+04
Relative Intensity
b1 /1+
y1 /1+
b2 /1+
y2 /1+
y3 /1+
b3 /1+
b4 /1+
b5 /1+
b7 /1+
b8 /1+
1 b8 /1+
Y0 /1+
138.06
168.07
186.08
204.09
Δm (ppm)
m/z

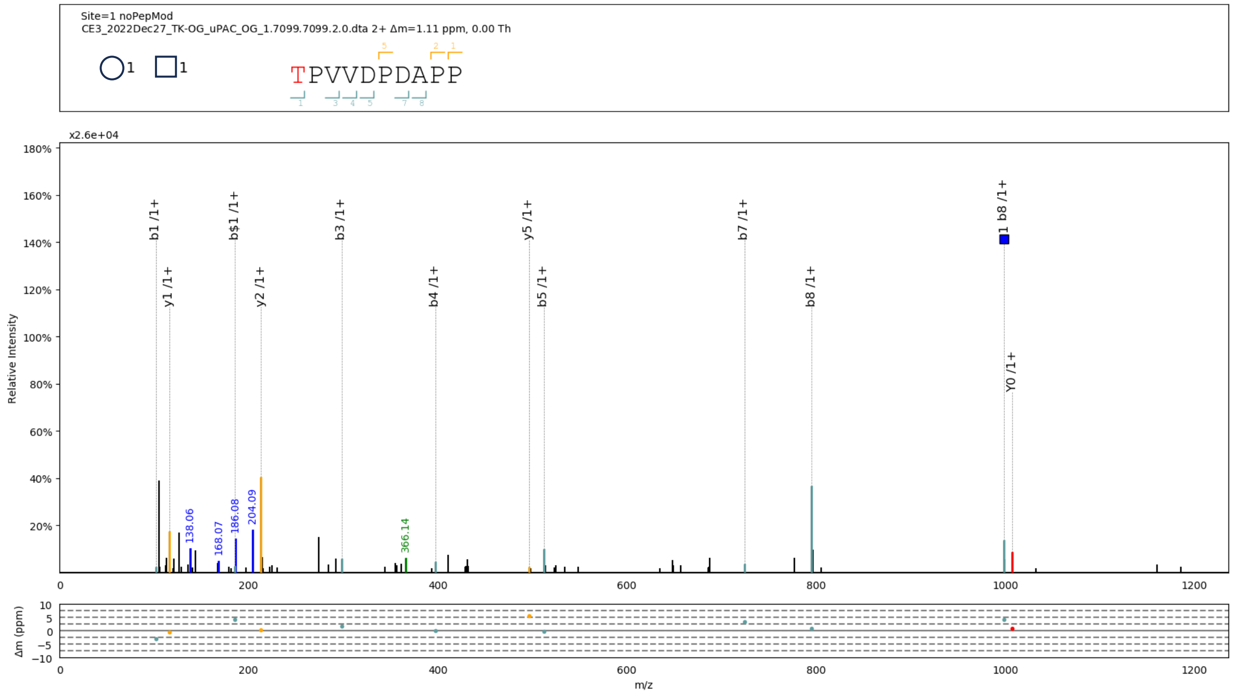
Site=1 noPepMod
CE3_2022Dec27_TK-OG_uPAC_OG_1.7099.7099.2.0.dta 2+ Δm=1.11 ppm, 0.00 Th
1
1
TPVVDPDAPP
x2.6e+04
Relative Intensity
b1 /1+
y1 /1+
b$1 /1+
y2 /1+
b3 /1+
b4 /1+
y5 /1+
b5 /1+
b7 /1+
b8 /1+
1 b8 /1+
Y0 /1+
138.06
168.07
186.08
204.09
366.14
Δm (ppm)
m/z

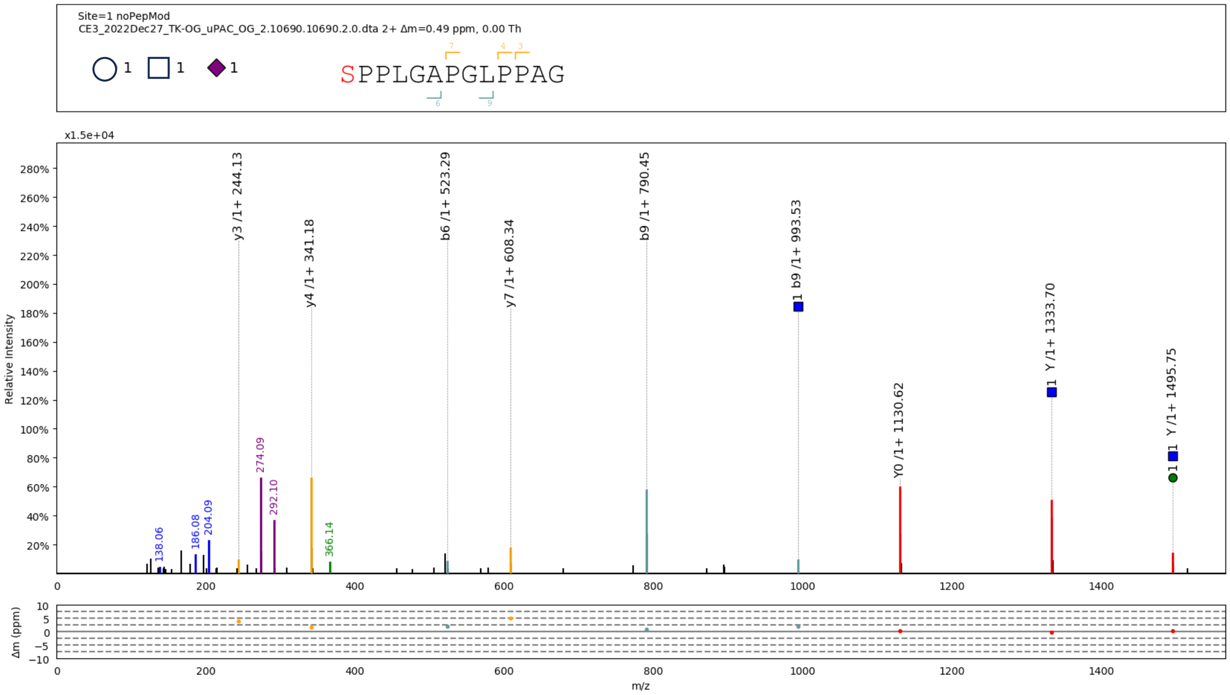
Site=1 noPepMod
CE3_2022Dec27_TK-OG_uPAC_OG_2.10690.10690.2.0.dta 2+ Δm=0.49 ppm, 0.00 Th
SPPLGAPGLPPAG
x1.5e+04
Relative Intensity
y3 /1+ 244.13
y4 /1+ 341.18
b6 /1+ 523.29
y7 /1+ 608.34
b9 /1+ 790.45
1 b9 /1+ 993.53
Y0 /1+ 1130.62
1 Y /1+ 1333.70
1 1 Y /1+ 1495.75
138.06
186.08
204.09
274.09
292.10
366.14
m/z
Δm (ppm)

(B) Kininogen-1 (KNG1)

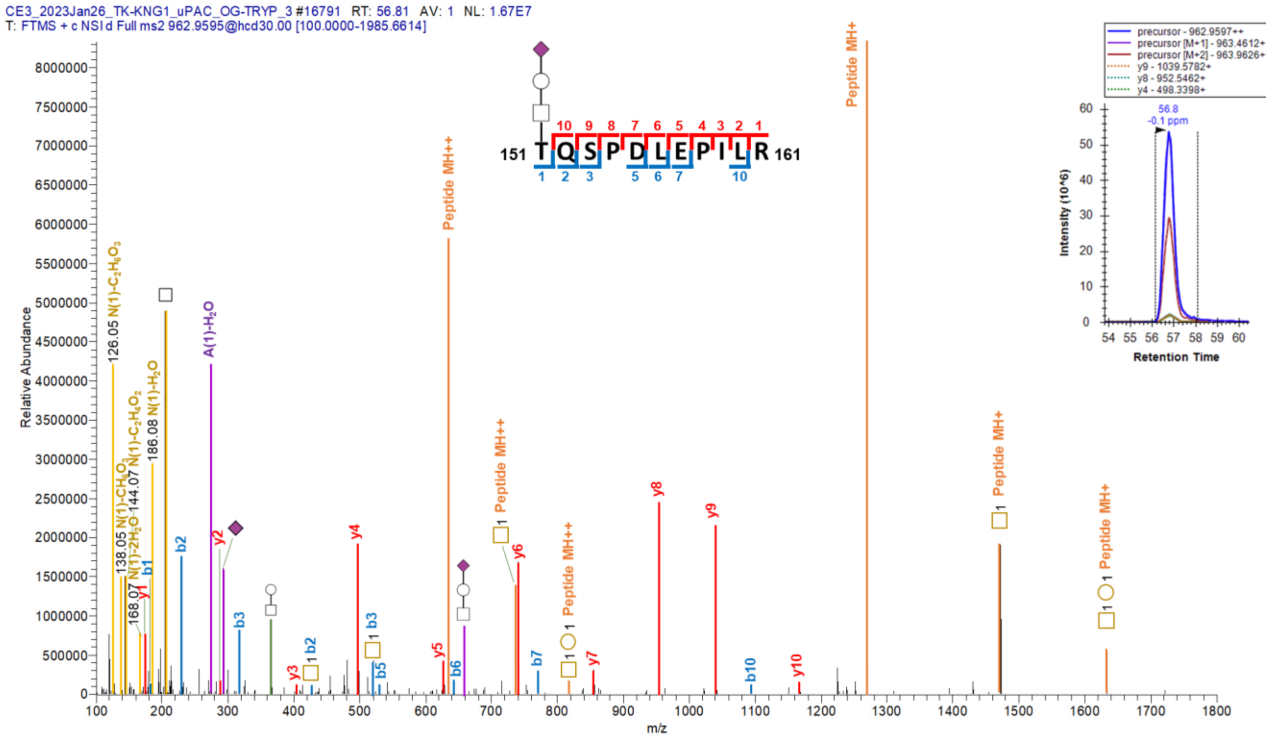

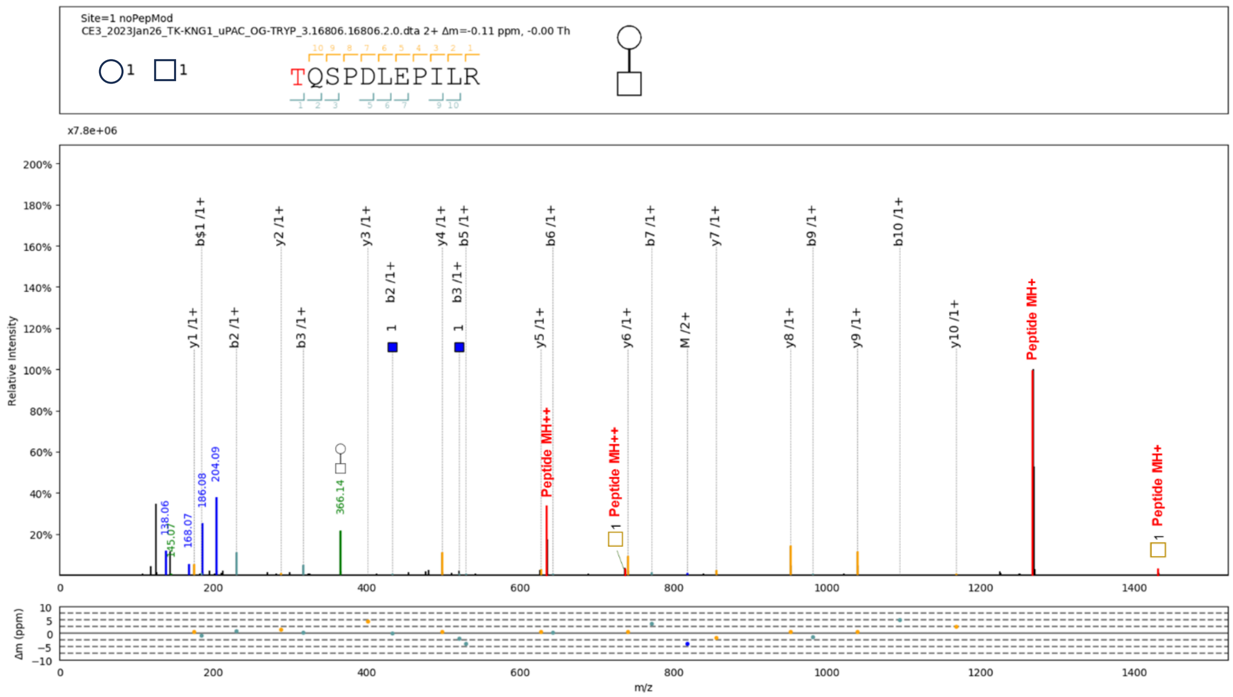

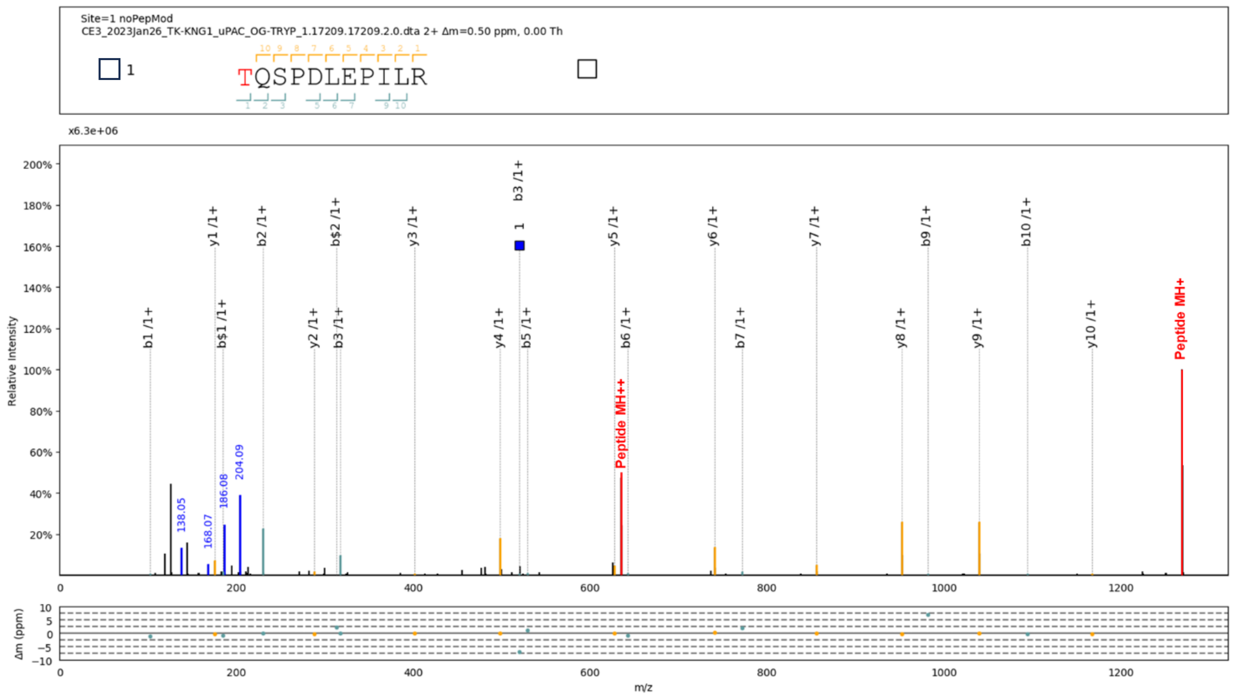
Site=1 noPepMod
CE3_2023Jan26_TK-KNG1_uPAC_OG-TRYP_1.17209.17209.2.0.dta 2+ Δm=0.50 ppm, 0.00 Th
1
TQSPDLEPILR
x6.3e+06
Relative Intensity
b1 /1+
b$1 /1+
y1 /1+
b2 /1+
y2 /1+
b$2 /1+
b3 /1+
y3 /1+
y4 /1+
b3 /1+
b5 /1+
y5 /1+
b6 /1+
y6 /1+
b7 /1+
y7 /1+
y8 /1+
b9 /1+
y9 /1+
b10 /1+
y10 /1+
Peptide MH++
Peptide MH+
138.05
168.07
186.08
204.09
Δm (ppm)
m/z

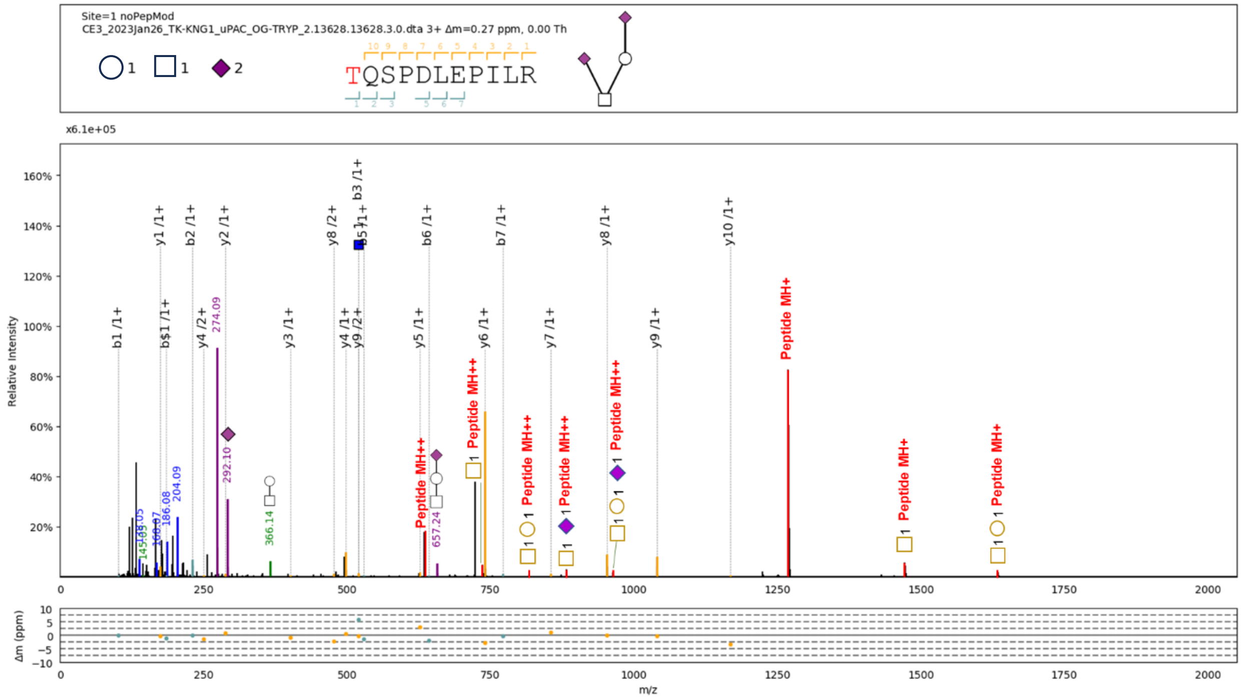
Site=1 noPepMod
CE3_2023Jan26_TK-KNG1_uPAC_OG-TRYP_2.13628.13628.3.0.dta 3+ Δm=0.27 ppm, 0.00 Th
1
1
2
TQSPDLEPILR
x6.1e+05
Relative Intensity
b1 /1+
b$1 /1+
y1 /1+
b2 /1+
y4 /2+
y2 /1+
y3 /1+
y8 /2+
y4 /1+
y9 /2+
b3 /1+
b5 /1+
y5 /1+
b6 /1+
y6 /1+
b7 /1+
y7 /1+
y8 /1+
y9 /1+
y10 /1+
Peptide MH++
Peptide MH+
145.05
168.07
186.08
204.09
274.09
292.10
366.14
657.24
Δm (ppm)
m/z

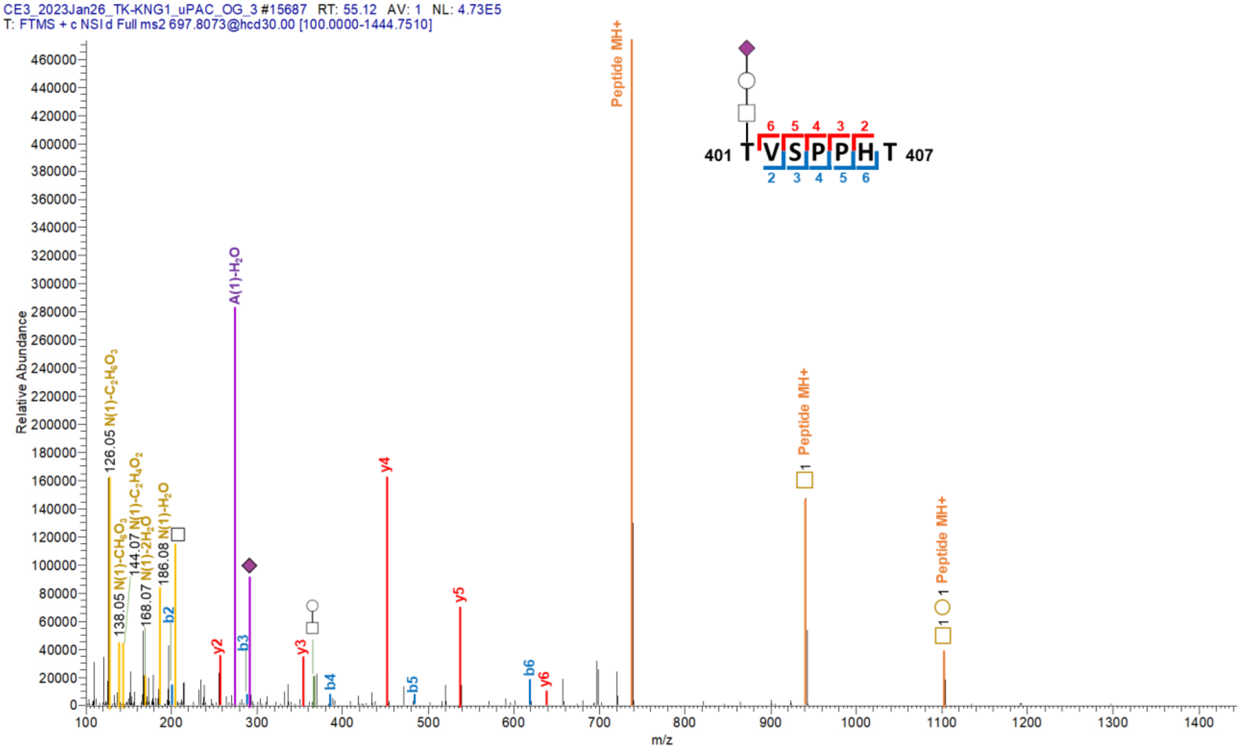
CE3_2023Jan26_TK-KNG1_uPAC_OG_3 #15687 RT: 55.12 AV: 1 NL: 4.73E5
T: FTMS + c NSI d Full ms2 697.8073@hcd30.00 [100.0000-1444.7510]
Relative Abundance
m/z
Peptide MH+
401 T V S P P H T 407
A(1)-H2O
126.05 N(1)-C2H6O3
138.05 N(1)-CH6O3
144.07 N(1)-C2H4O2
168.07 N(1)-2H2O
186.08 N(1)-H2O
y2 y3 y4 y5 y6
b2 b3 b4 b5 b6
1 Peptide MH+
1 1 Peptide MH+

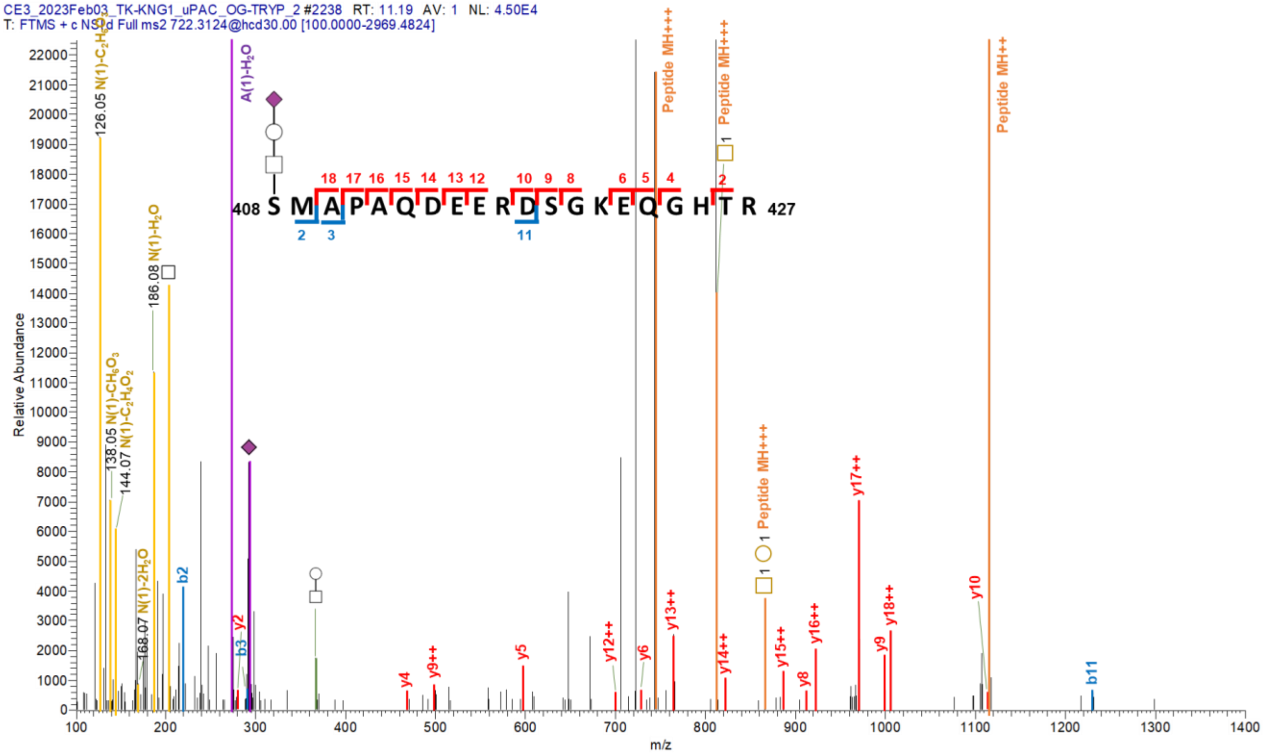
CE3_2023Feb03_TK-KNG1_uPAC_OG-TRYP_2 #2238 RT: 11.19 AV: 1 NL: 4.50E4
T: FTMS + c NSI d Full ms2 722.3124@hcd30.00 [100.0000-2969.4824]
Relative Abundance
m/z
408 S M A P A Q D E E R D S G K E Q G H T R 427
A(1)-H2O
126.05 N(1)-C2H6O3
138.05 N(1)-CH6O3
144.07 N(1)-C2H4O2
168.07 N(1)-2H2O
186.08 N(1)-H2O
Peptide MH+++
Peptide MH++
1 1 Peptide MH+++
y2 y4 y5 y6 y8 y9 y10 y9++ y12++ y13++ y14++ y15++ y16++ y17++ y18++
b2 b3 b11

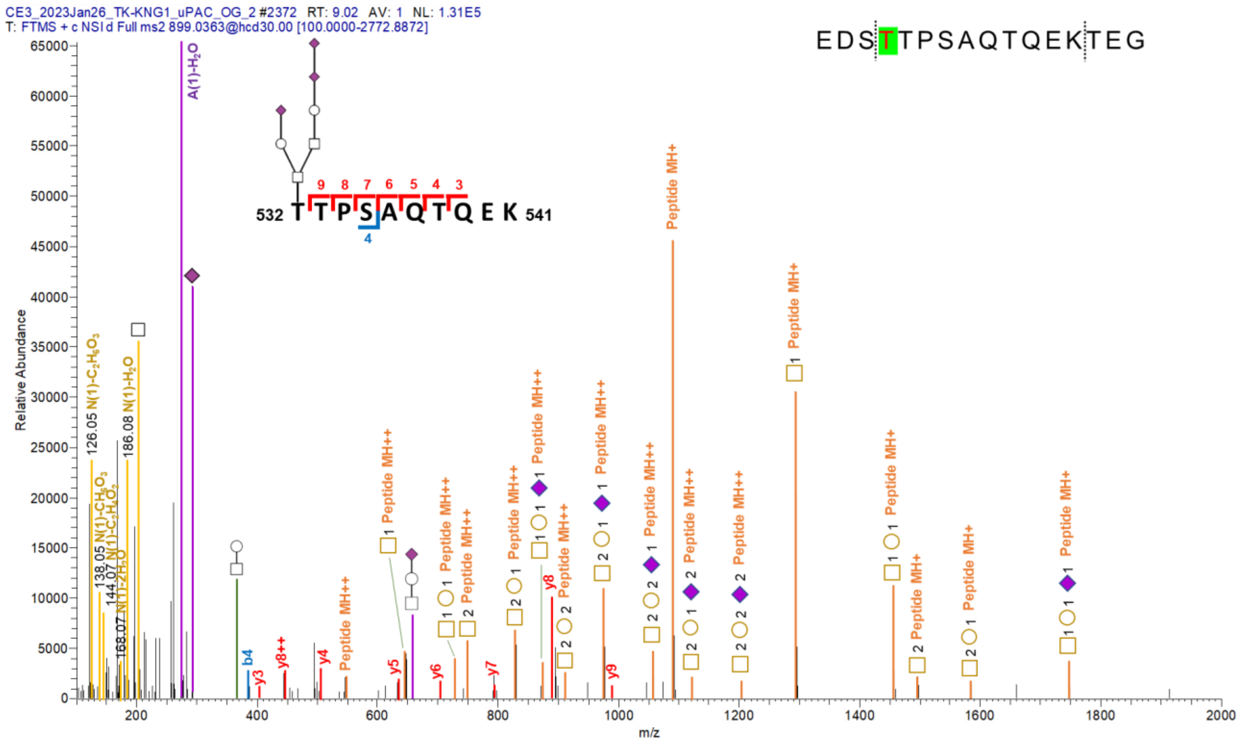
CE3_2023Jan26_TK-KNG1_uPAC_OG_2 #2372 RT: 9.02 AV: 1 NL: 1.31E5
T: FTMS + c NSI d Full ms2 899.0363@hcd30.00 [100.0000-2772.8872]
EDSTTPSAQTQEKTEG
532 T T P S A Q T Q E K 541
Relative Abundance
m/z
A(1)-H₂O
126.05 N(1)-C₂H₆O₃
138.05 N(1)-CH₆O₃
144.07 N(1)-C₂H₄O₂
168.07 N(1)-2H₂O
186.08 N(1)-H₂O
Peptide MH+
Peptide MH++

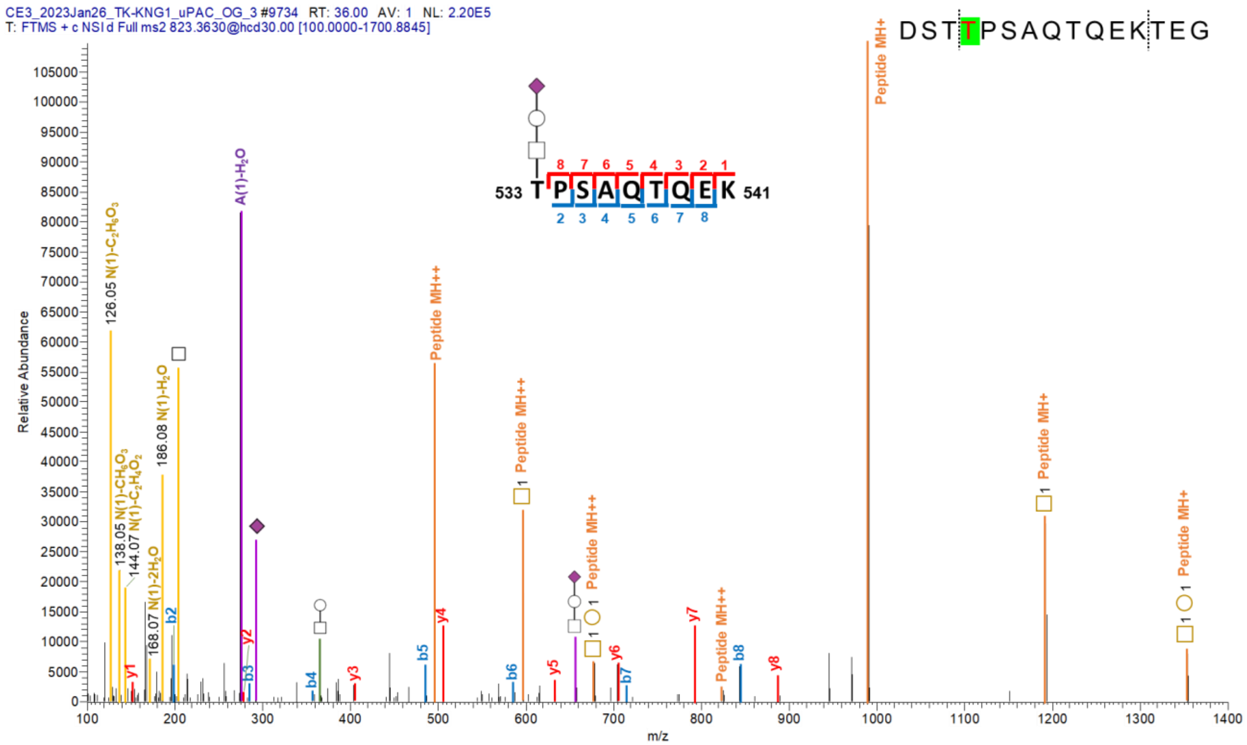
CE3_2023Jan26_TK-KNG1_uPAC_OG_3 #9734 RT: 36.00 AV: 1 NL: 2.20E5
T: FTMS + c NSI d Full ms2 823.3630@hcd30.00 [100.0000-1700.8845]
DSTTPSAQTQEKTEG
533 T P S A Q T Q E K 541
Relative Abundance
m/z
A(1)-H₂O
126.05 N(1)-C₂H₆O₃
138.05 N(1)-CH₆O₃
144.07 N(1)-C₂H₄O₂
168.07 N(1)-2H₂O
186.08 N(1)-H₂O
Peptide MH+
Peptide MH++

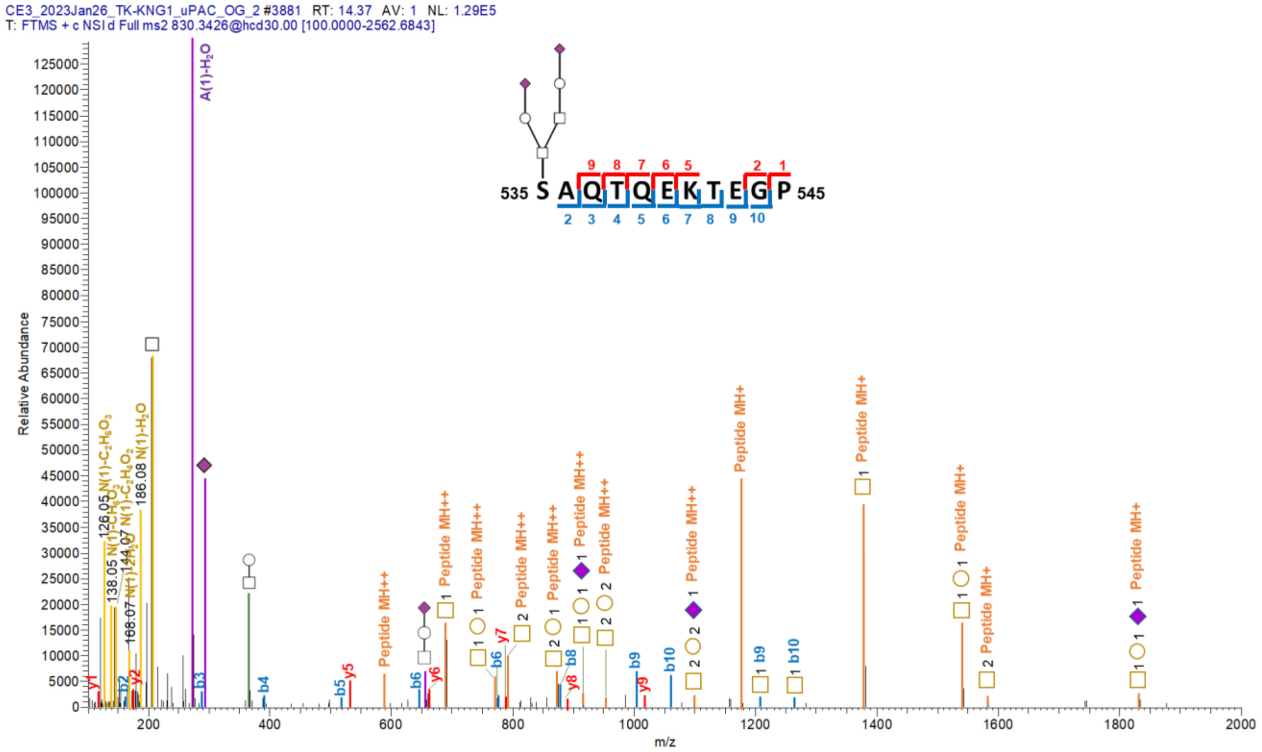
CE3_2023Jan26_TK-KNG1_uPAC_OG_2 #3881 RT: 14.37 AV: 1 NL: 1.29E5
T: FTMS + c NSI d Full ms2 830.3426@hcd30.00 [100.0000-2562.6843]
535 S A Q T Q E K T E G P 545
Relative Abundance
m/z
A(1)-H2O
Peptide MH+
Peptide MH++

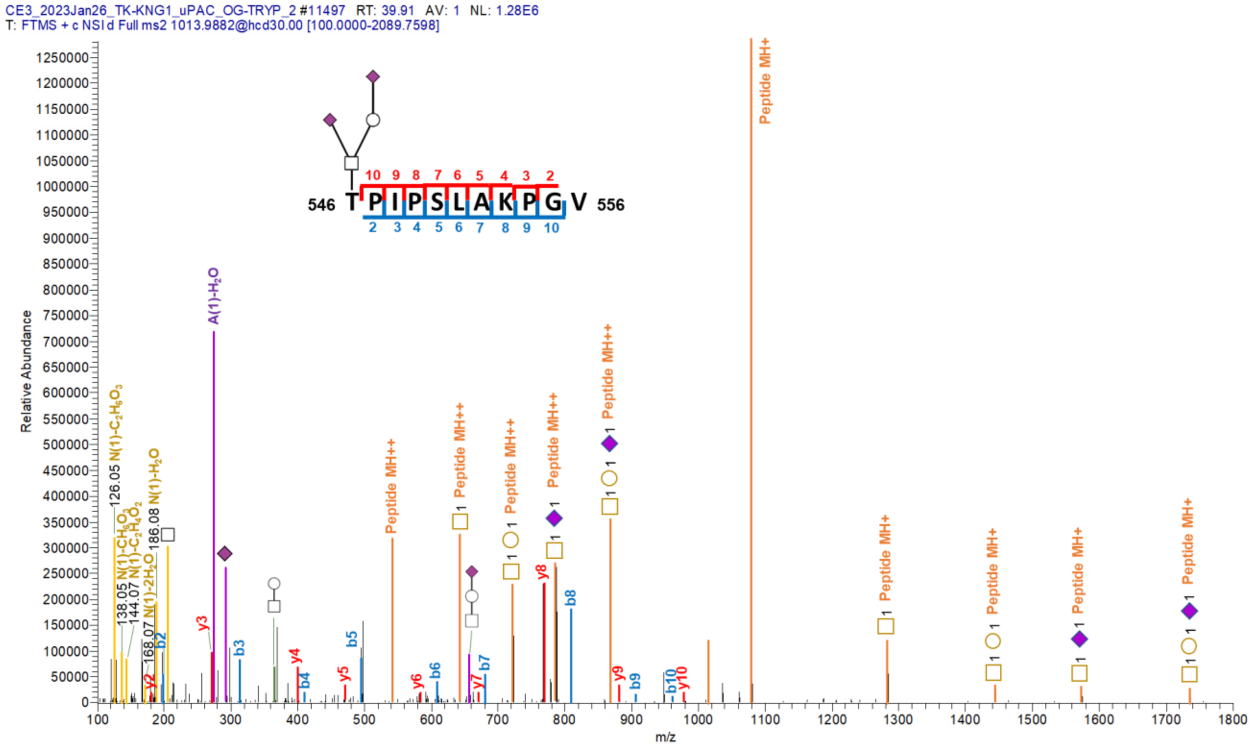
CE3_2023Jan26_TK-KNG1_uPAC_OG-TRYP_2 #11497 RT: 39.91 AV: 1 NL: 1.28E6
T: FTMS + c NSI d Full ms2 1013.9882@hcd30.00 [100.0000-2089.7598]
546 T P I P S L A K P G V 556
Relative Abundance
m/z
A(1)-H2O
Peptide MH+
Peptide MH++

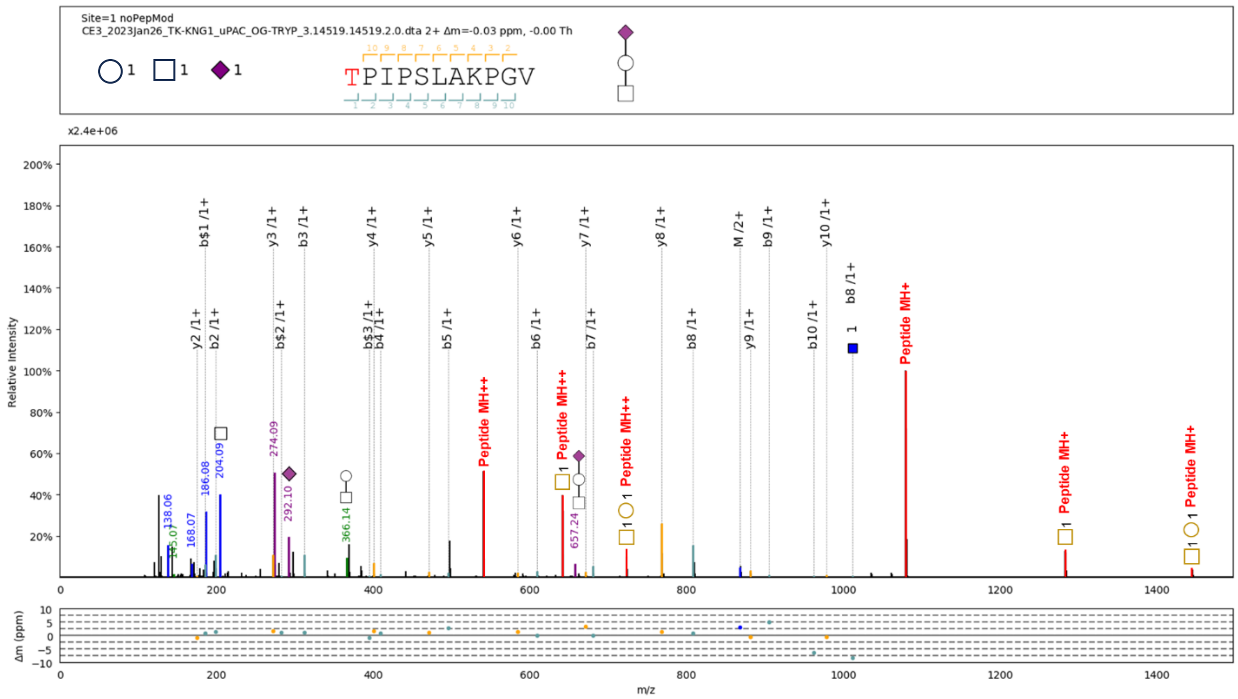

Site=1 noPepMod
CE3_2023Jan26_TK-KNG1_uPAC_OG-TRYP_3.14519.14519.2.0.dta 2+ Δm=-0.03 ppm, -0.00 Th
TPIPSLAKPGV
x2.4e+06
Relative Intensity
b$1 /1+
y3 /1+
b3 /1+
y4 /1+
y5 /1+
y6 /1+
y7 /1+
y8 /1+
M /2+
b9 /1+
y10 /1+
y2 /1+
b2 /1+
b$2 /1+
b$3 /1+
b4 /1+
b5 /1+
b6 /1+
b7 /1+
b8 /1+
y9 /1+
b10 /1+
Peptide MH++
Peptide MH+
138.06
145.07
168.07
186.08
204.09
274.09
292.10
366.14
657.24
Δm (ppm)
m/z

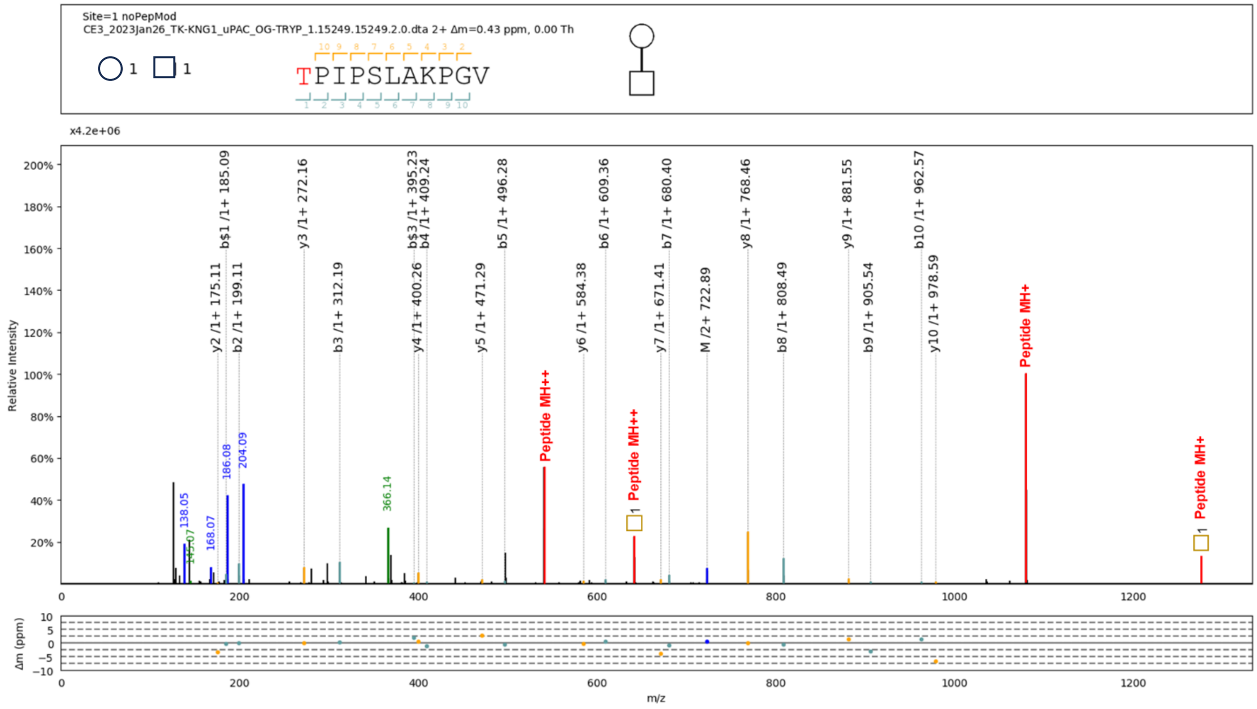

Site=1 noPepMod
CE3_2023Jan26_TK-KNG1_uPAC_OG-TRYP_1.15249.15249.2.0.dta 2+ Δm=0.43 ppm, 0.00 Th
TPIPSLAKPGV
x4.2e+06
Relative Intensity
b$1 /1+ 185.09
y3 /1+ 272.16
b$3 /1+ 395.23
b4 /1+ 409.24
b5 /1+ 496.28
b6 /1+ 609.36
b7 /1+ 680.40
y8 /1+ 768.46
y9 /1+ 881.55
b10 /1+ 962.57
y2 /1+ 175.11
b2 /1+ 199.11
b3 /1+ 312.19
y4 /1+ 400.26
y5 /1+ 471.29
y6 /1+ 584.38
y7 /1+ 671.41
M /2+ 722.89
b8 /1+ 808.49
b9 /1+ 905.54
y10 /1+ 978.59
Peptide MH++
Peptide MH+
138.05
145.07
168.07
186.08
204.09
366.14
Δm (ppm)
m/z

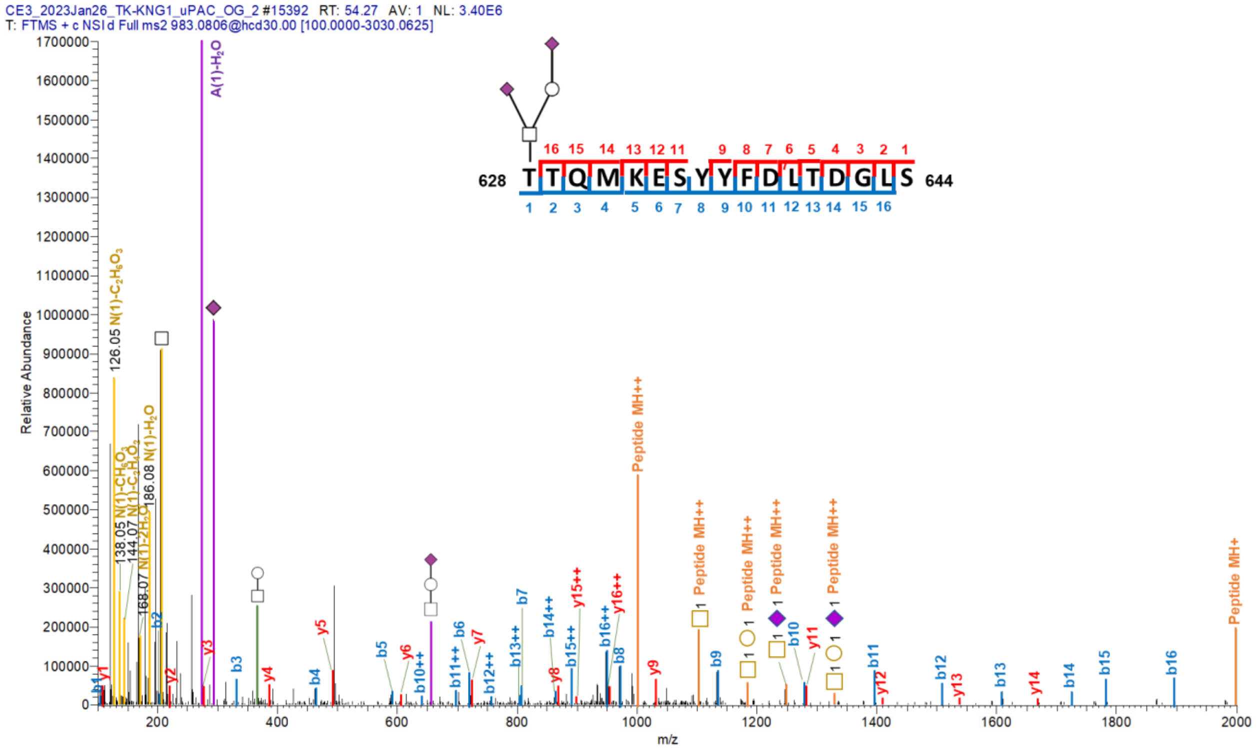

CE3_2023Jan26_TK-KNG1_uPAC_OG_2 #15392 RT: 54.27 AV: 1 NL: 3.40E6
T: FTMS + c NSI d Full ms2 983.0806@hcd30.00 [100.0000-3030.0625]
628 TTQMKESYYFDLTDGLS 644
Relative Abundance
m/z

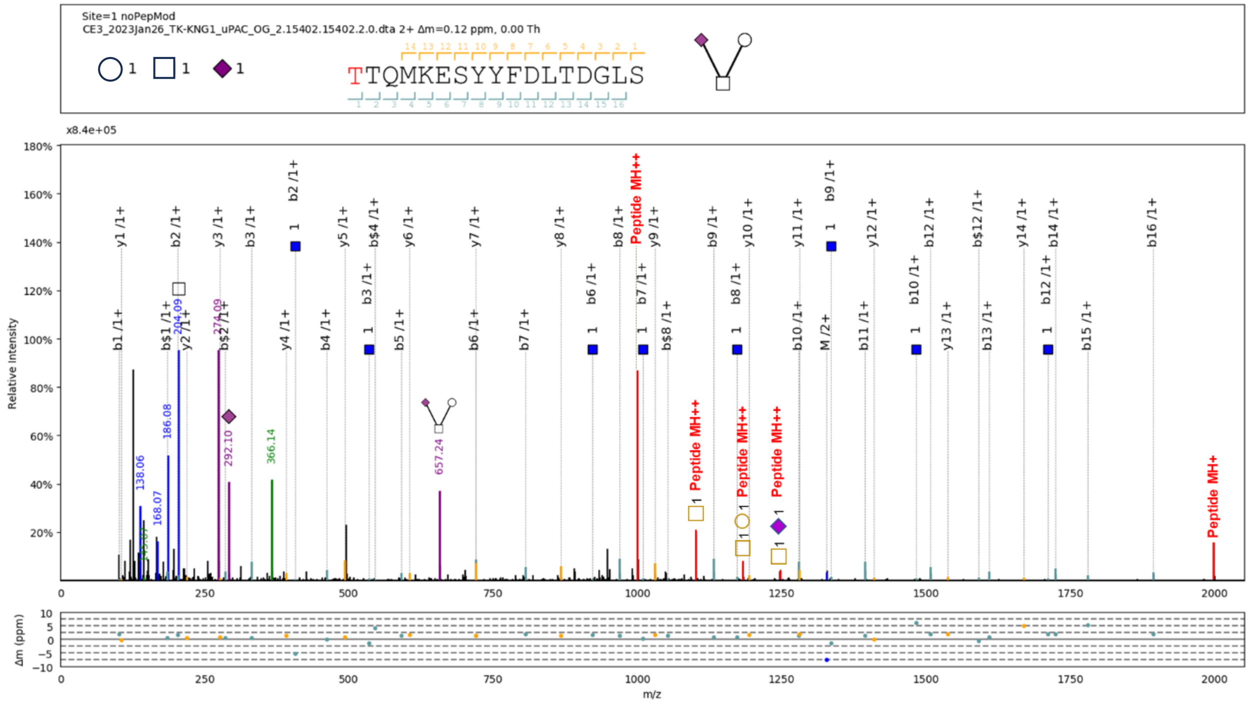

Site=1 noPepMod
CE3_2023Jan26_TK-KNG1_uPAC_OG_2.15402.15402.2.0.dta 2+ Δm=0.12 ppm, 0.00 Th
TTQMKESYYFDLTDGLS
Relative Intensity
Δm (ppm)
m/z

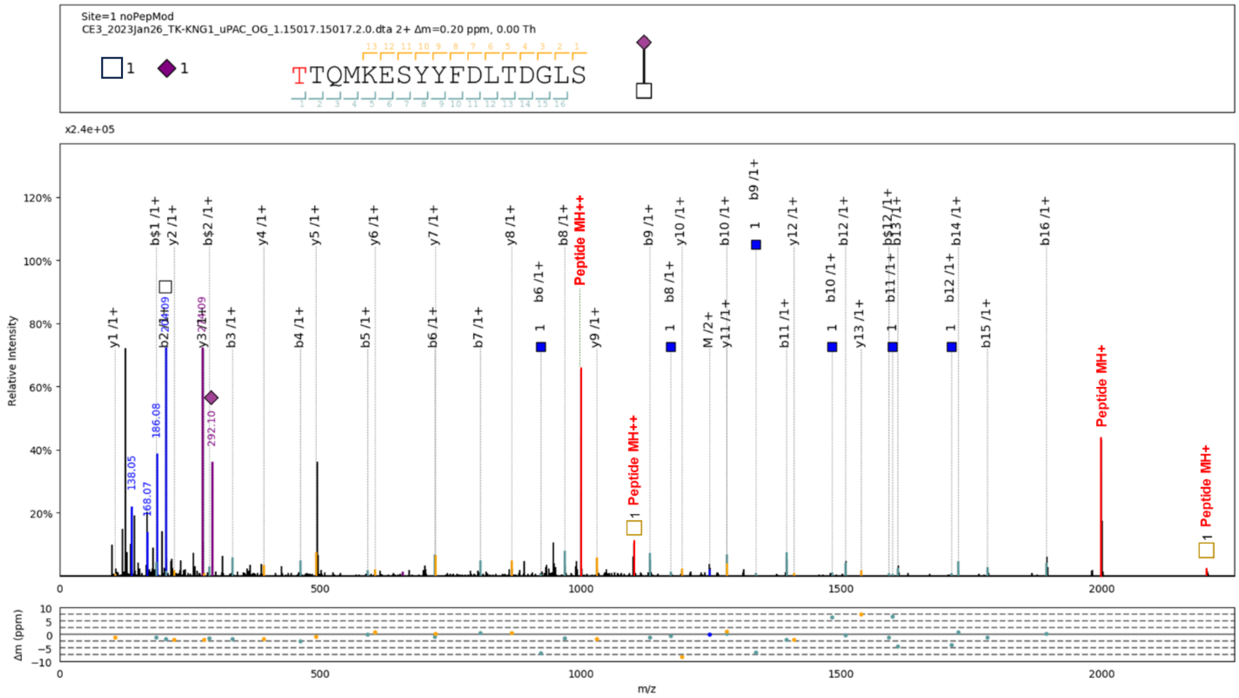

(C) Apolipoprotein E (APOE)

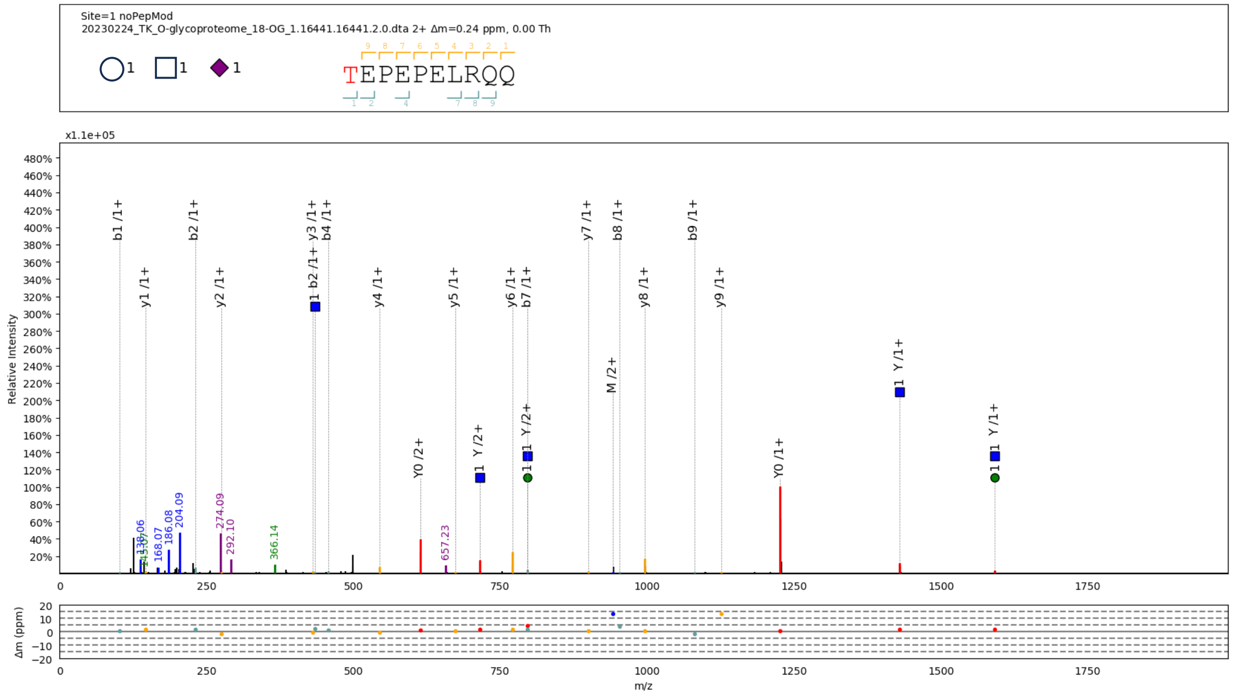

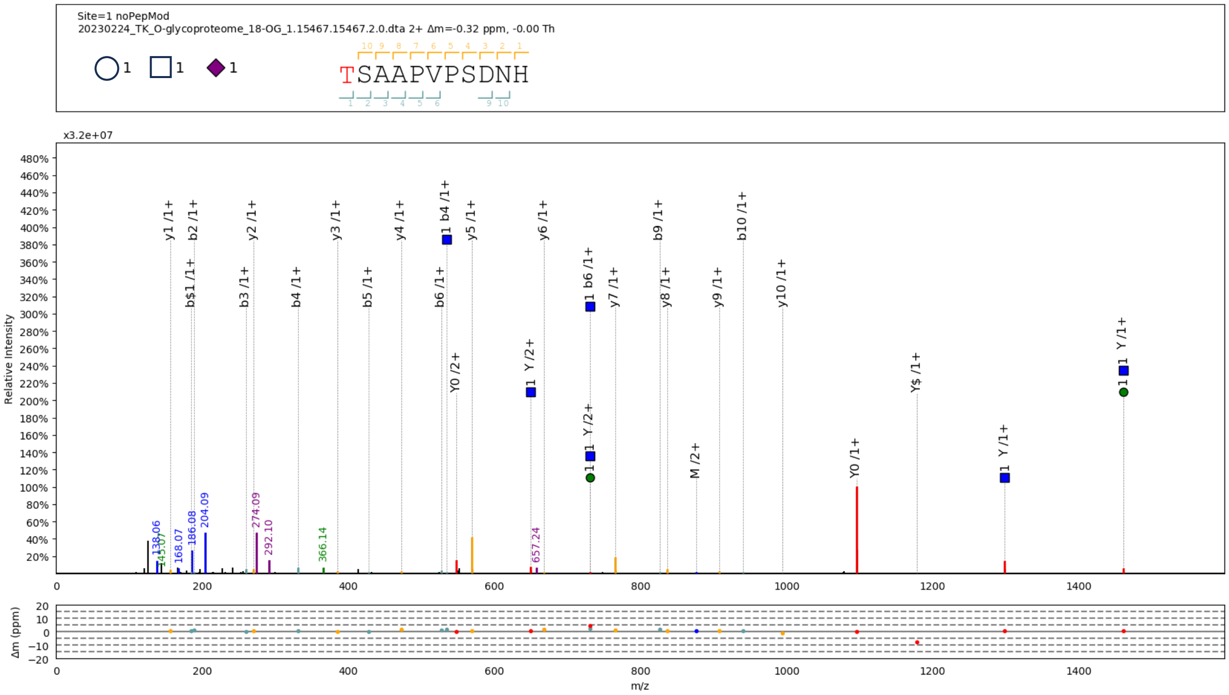
Site=1 noPepMod
20230224_TK_O-glycoproteome_18-OG_1.15467.15467.2.0.dta 2+ Δm=-0.32 ppm, -0.00 Th
TSAAPVPSDNH
x3.2e+07
Relative Intensity
Δm (ppm)
m/z

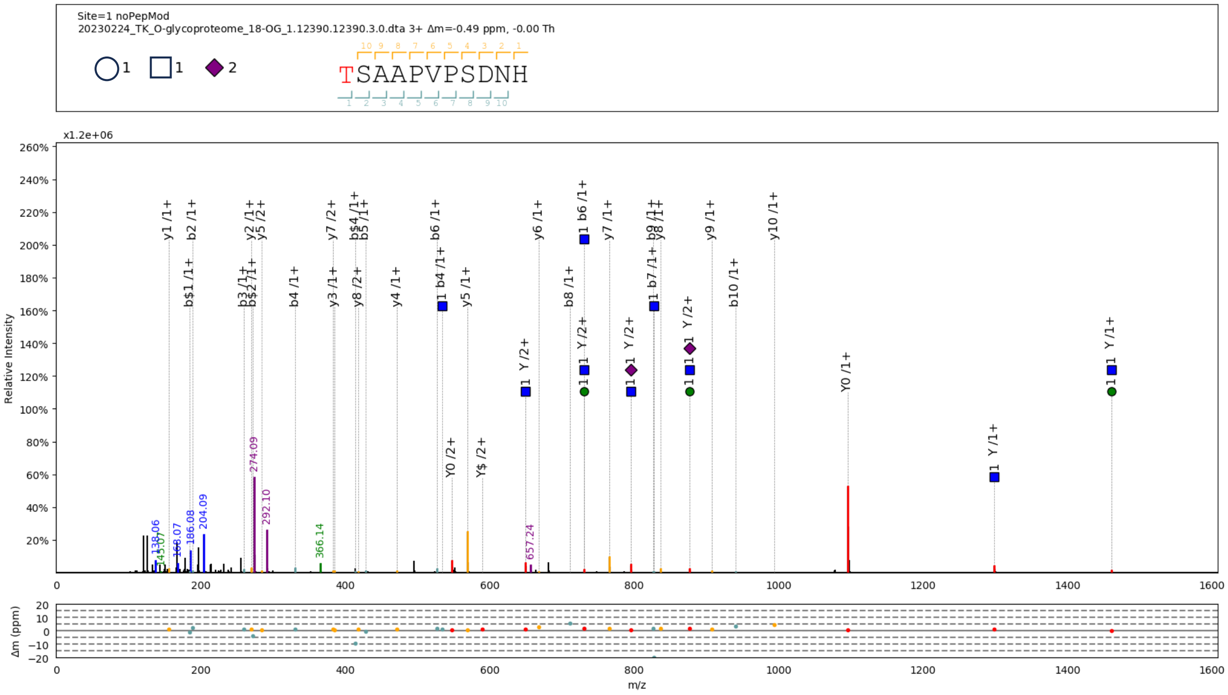
Site=1 noPepMod
20230224_TK_O-glycoproteome_18-OG_1.12390.12390.3.0.dta 3+ Δm=-0.49 ppm, -0.00 Th
TSAAPVPSDNH
x1.2e+06
Relative Intensity
Δm (ppm)
m/z

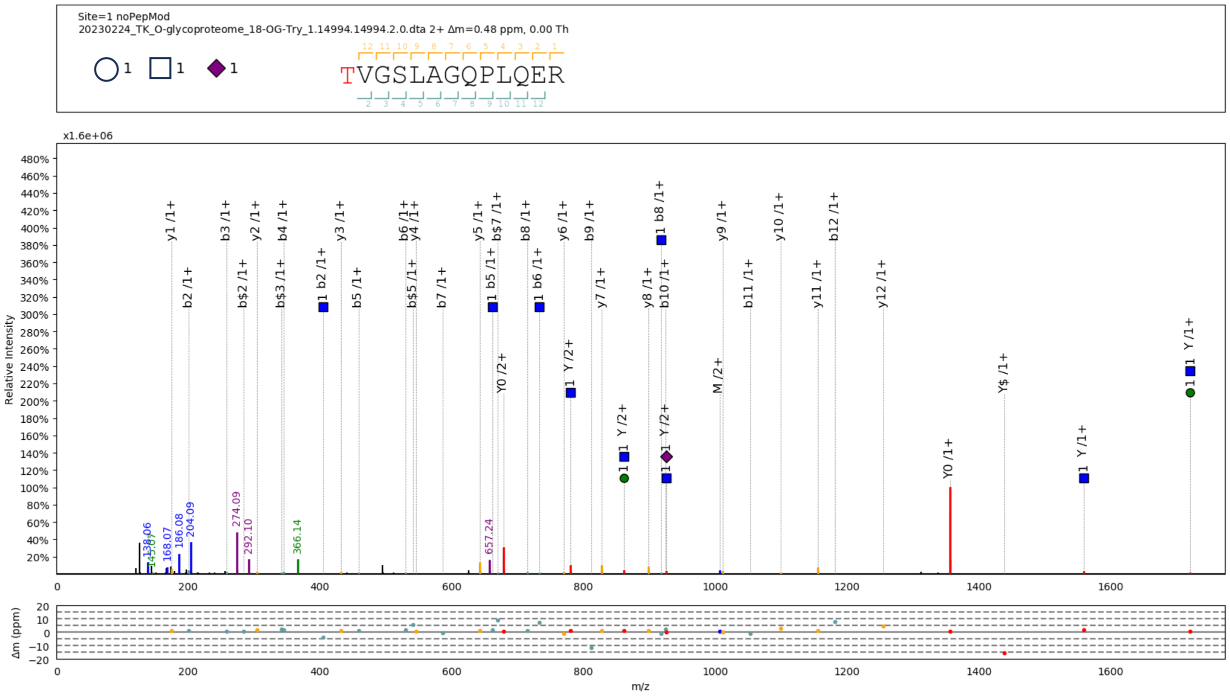

Site=1 noPepMod
20230224_TK_O-glycoproteome_18-OG-Try_1.14994.14994.2.0.dta 2+ Δm=0.48 ppm, 0.00 Th
TVGSLAGQPLQER
x1.6e+06
Relative Intensity
Δm (ppm)
m/z

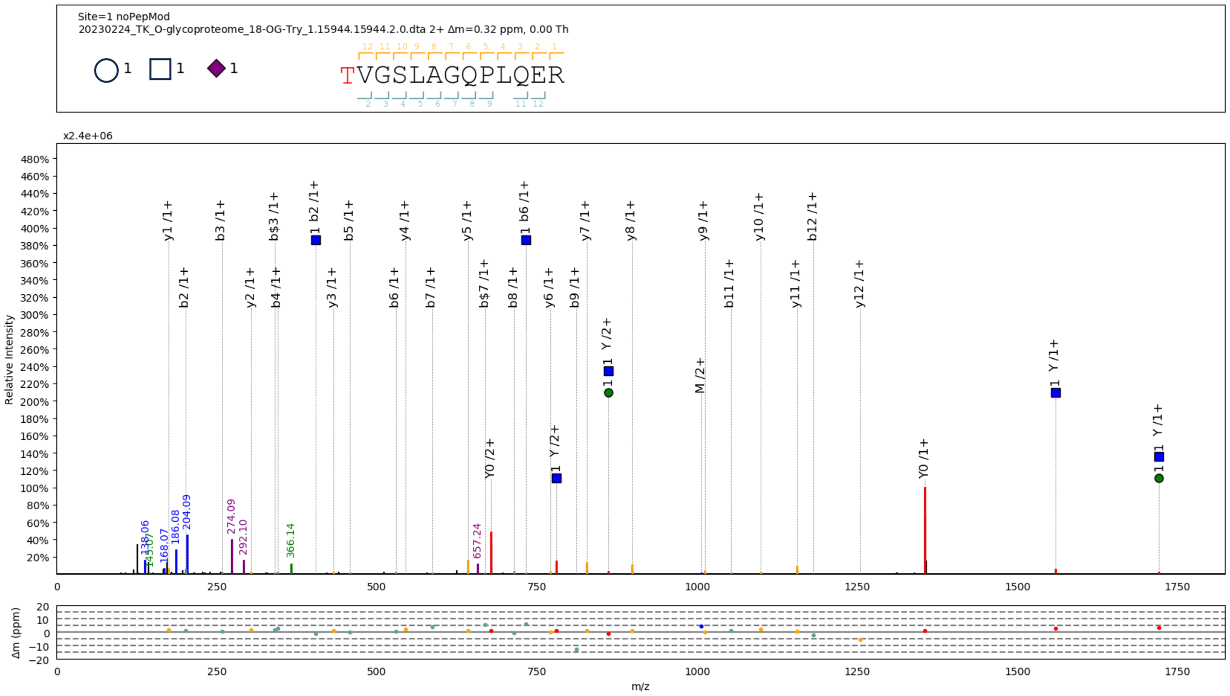

Site=1 noPepMod
20230224_TK_O-glycoproteome_18-OG-Try_1.15944.15944.2.0.dta 2+ Δm=0.32 ppm, 0.00 Th
TVGSLAGQPLQER
x2.4e+06
Relative Intensity
Δm (ppm)
m/z

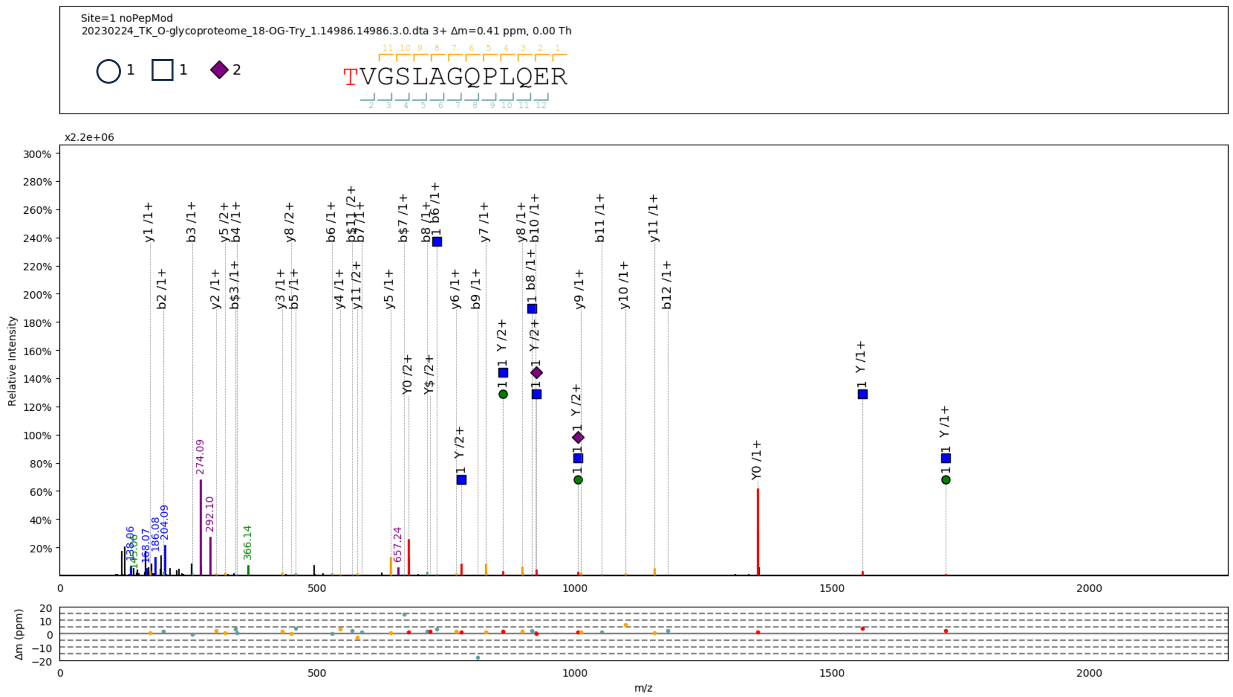
Site=1 noPepMod
20230224_TK_O-glycoproteome_18-OG-Try_1.14986.14986.3.0.dta 3+ Δm=0.41 ppm, 0.00 Th
TVGSLAGQPLQER
x2.2e+06
Relative Intensity
Δm (ppm)
m/z

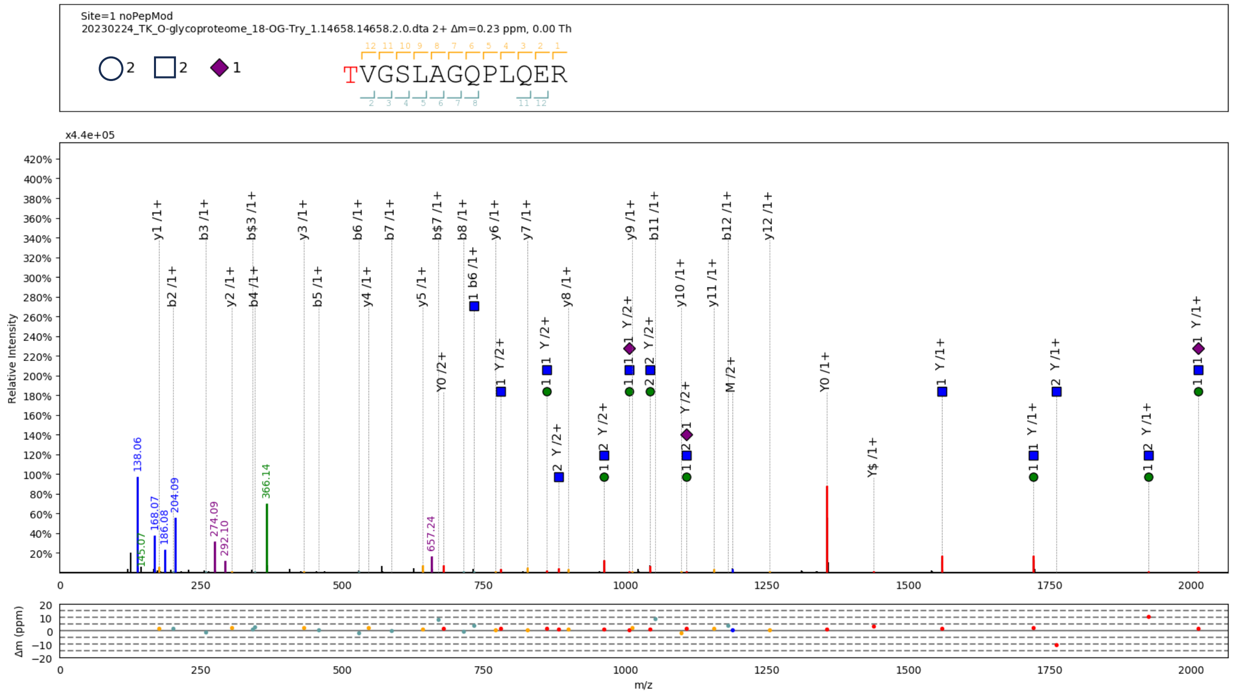
Site=1 noPepMod
20230224_TK_O-glycoproteome_18-OG-Try_1.14658.14658.2.0.dta 2+ Δm=0.23 ppm, 0.00 Th
TVGSLAGQPLQER
x4.4e+05
Relative Intensity
Δm (ppm)
m/z

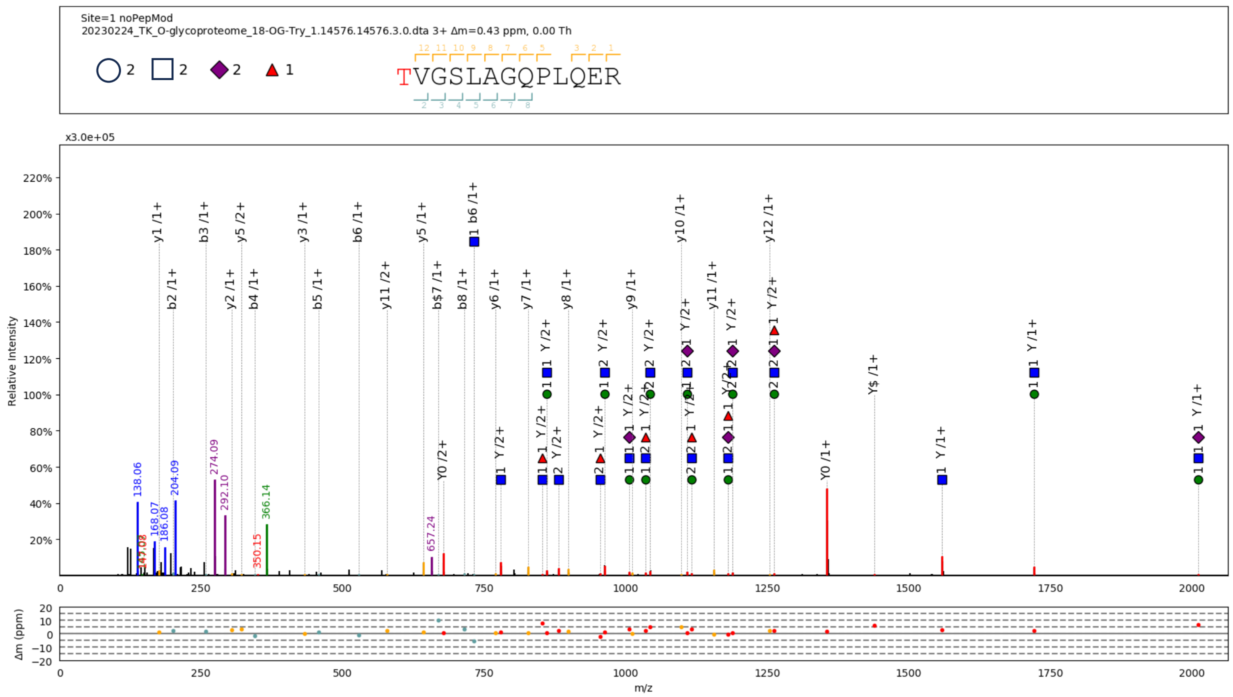

(D) Fibrinogen alpha chain (FGA)

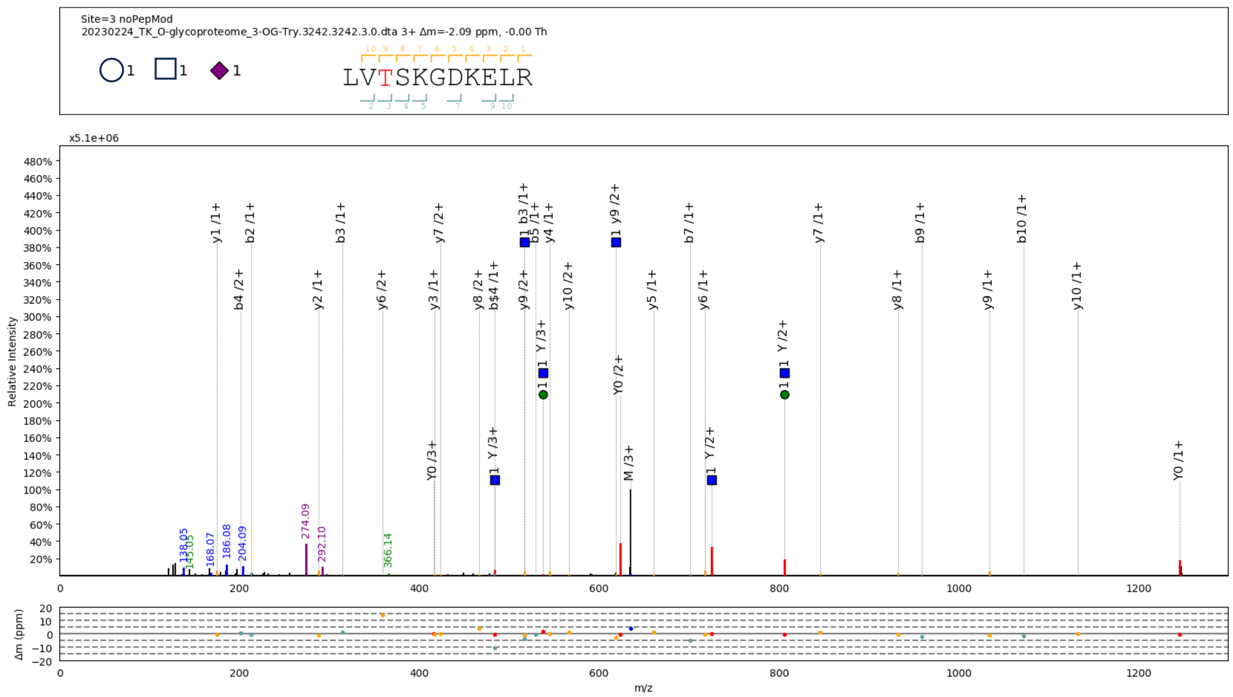

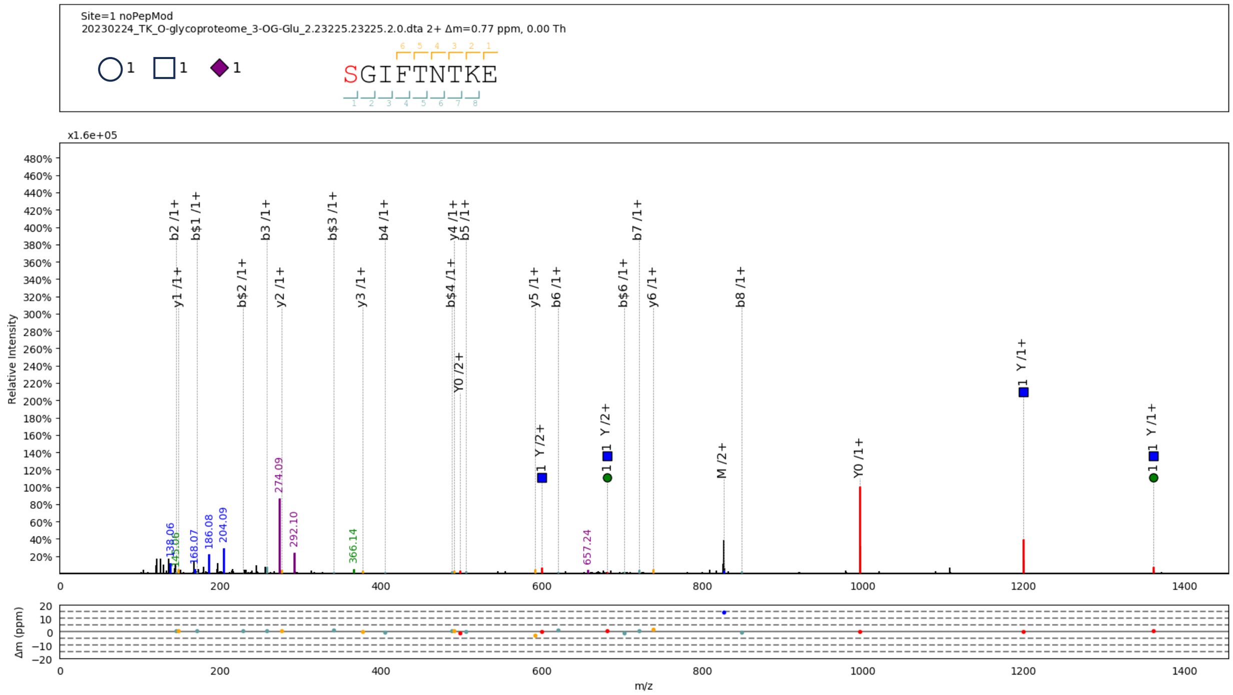
Site=1 noPepMod
20230224_TK_O-glycoproteome_3-OG-Glu_2.23225.23225.2.0.dta 2+ Δm=0.77 ppm, 0.00 Th
SGIFTNTKE
x1.6e+05
Relative Intensity
Δm (ppm)
m/z

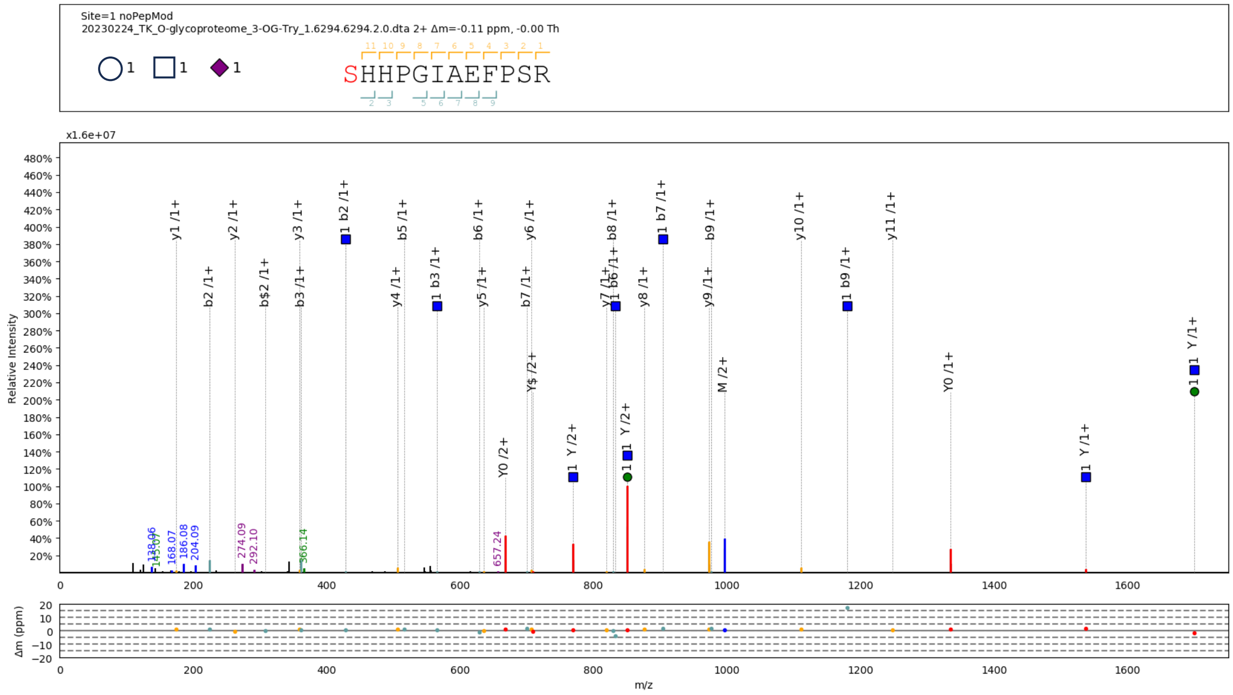
Site=1 noPepMod
20230224_TK_O-glycoproteome_3-OG-Try_1.6294.6294.2.0.dta 2+ Δm=-0.11 ppm, -0.00 Th
SHHPGIAEFPSR
x1.6e+07
Relative Intensity
Δm (ppm)
m/z

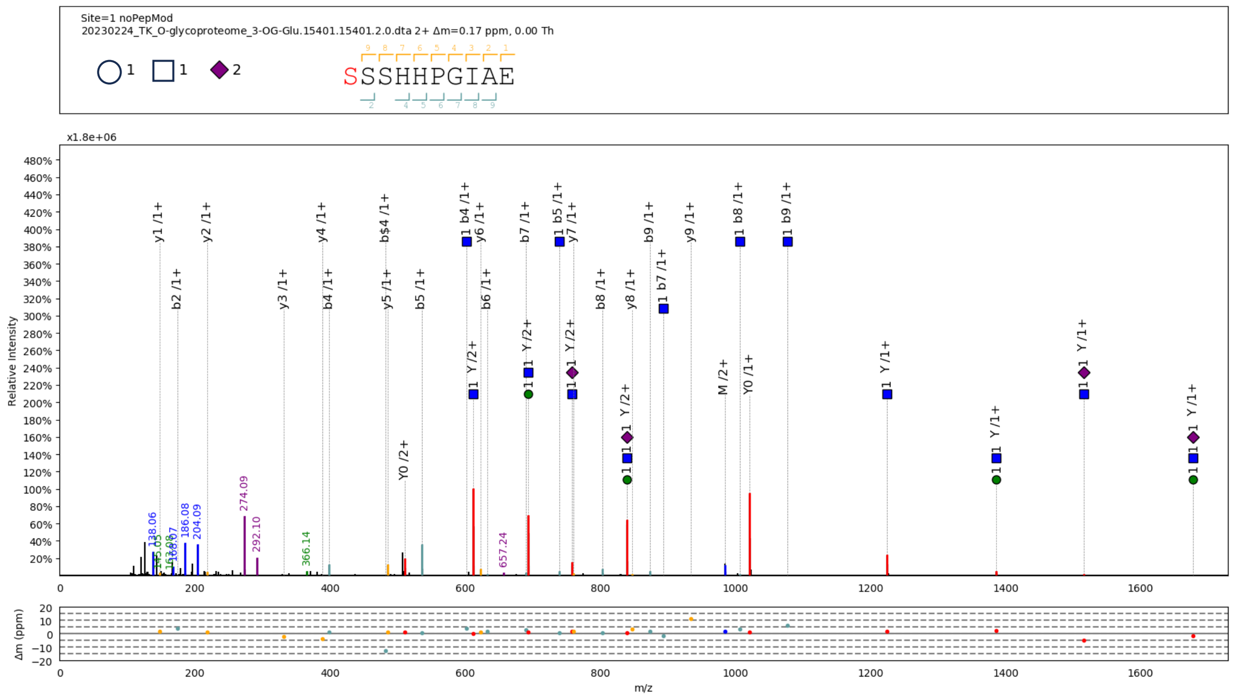
Site=1 noPepMod
20230224_TK_O-glycoproteome_3-OG-Glu.15401.15401.2.0.dta 2+ Δm=0.17 ppm, 0.00 Th
SSSHHPGIAE
x1.8e+06
Relative Intensity
Δm (ppm)
m/z

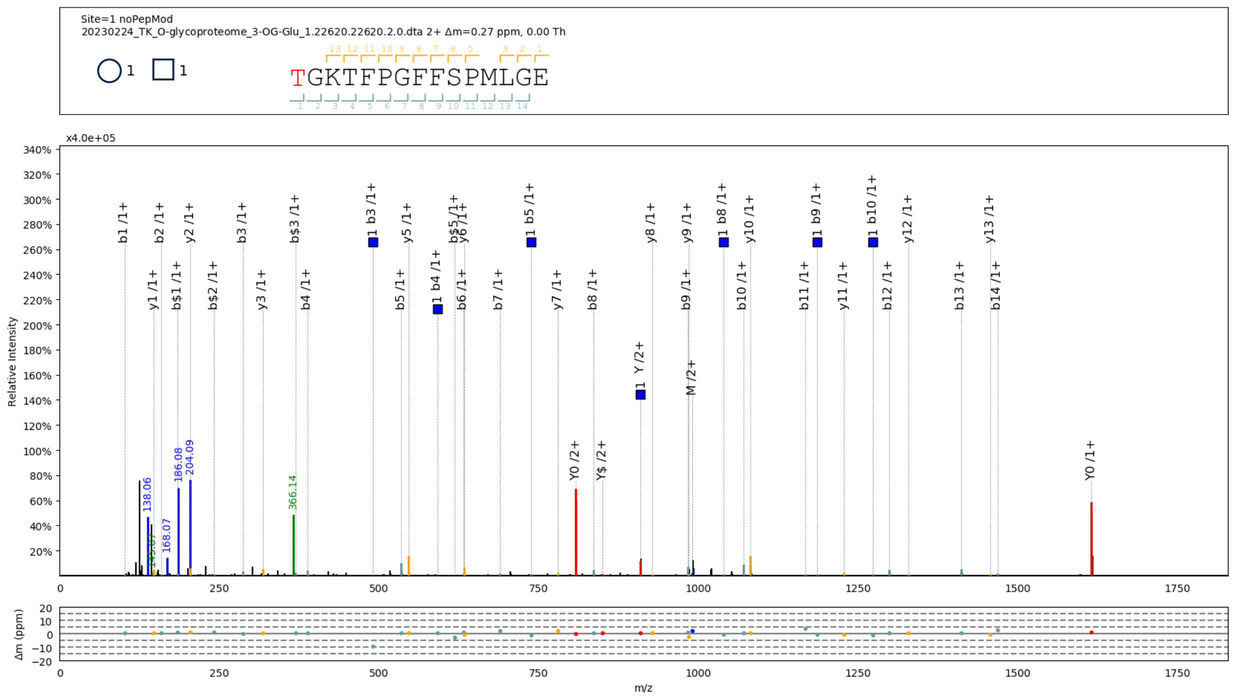
Site=1 noPepMod
20230224_TK_O-glycoproteome_3-OG-Glu_1.22620.22620.2.0.dta 2+ Δm=0.27 ppm, 0.00 Th
TGKTFPGFFSPMLGE
x4.0e+05
Relative Intensity
Δm (ppm)
m/z

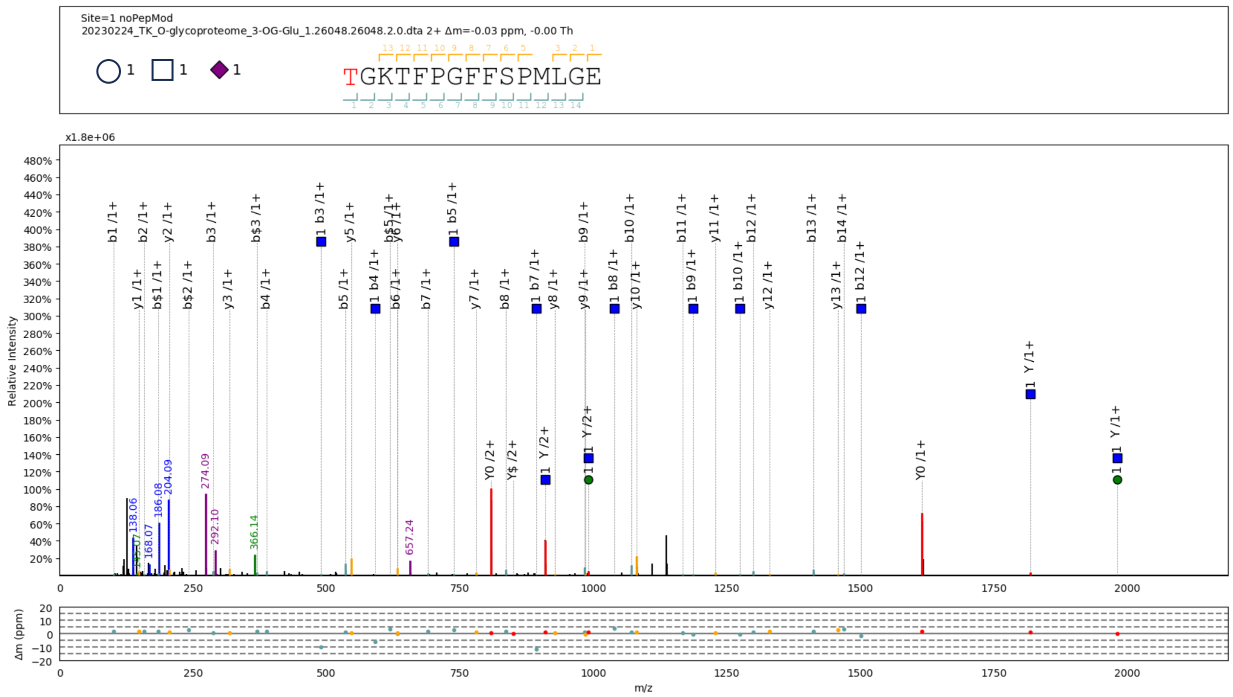
Site=1 noPepMod
20230224_TK_O-glycoproteome_3-OG-Glu_1.26048.26048.2.0.dta 2+ Δm=-0.03 ppm, -0.00 Th
TGKTFPGFFSPMLGE
x1.8e+06
Relative Intensity
Δm (ppm)
m/z

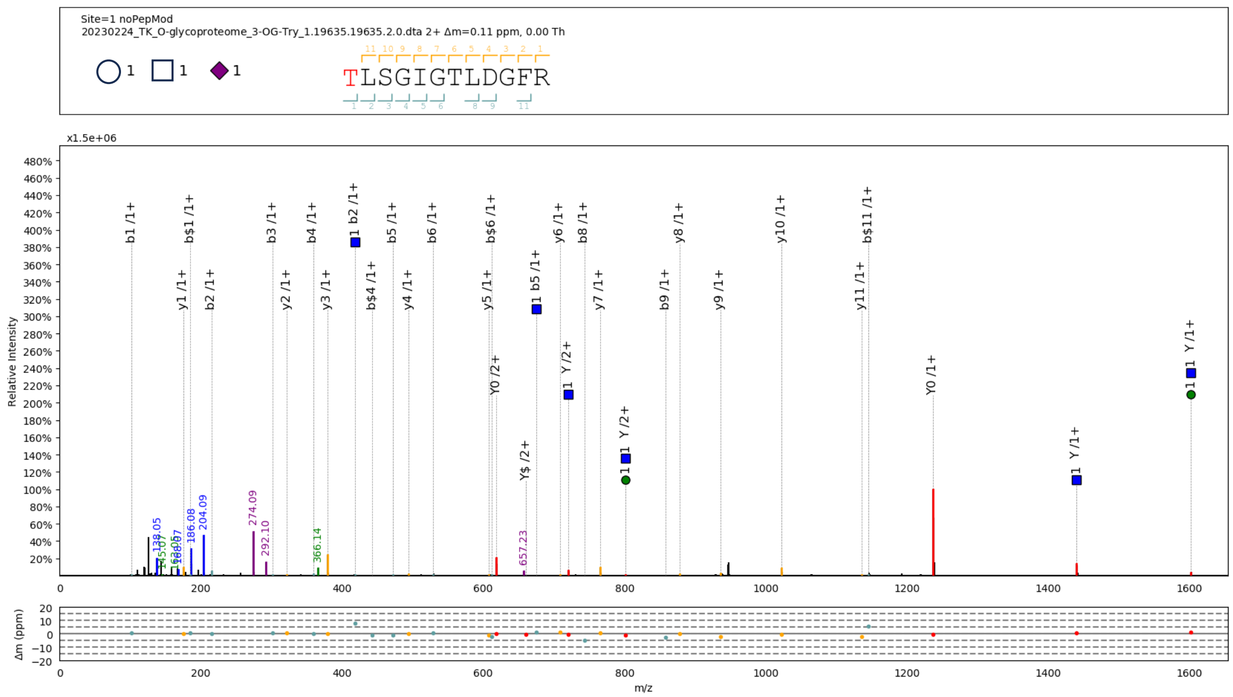
Site=1 noPepMod
20230224_TK_O-glycoproteome_3-OG-Try_1.19635.19635.2.0.dta 2+ Δm=0.11 ppm, 0.00 Th
TLSGIGTLDGFR
x1.5e+06
Relative Intensity
Δm (ppm)
m/z

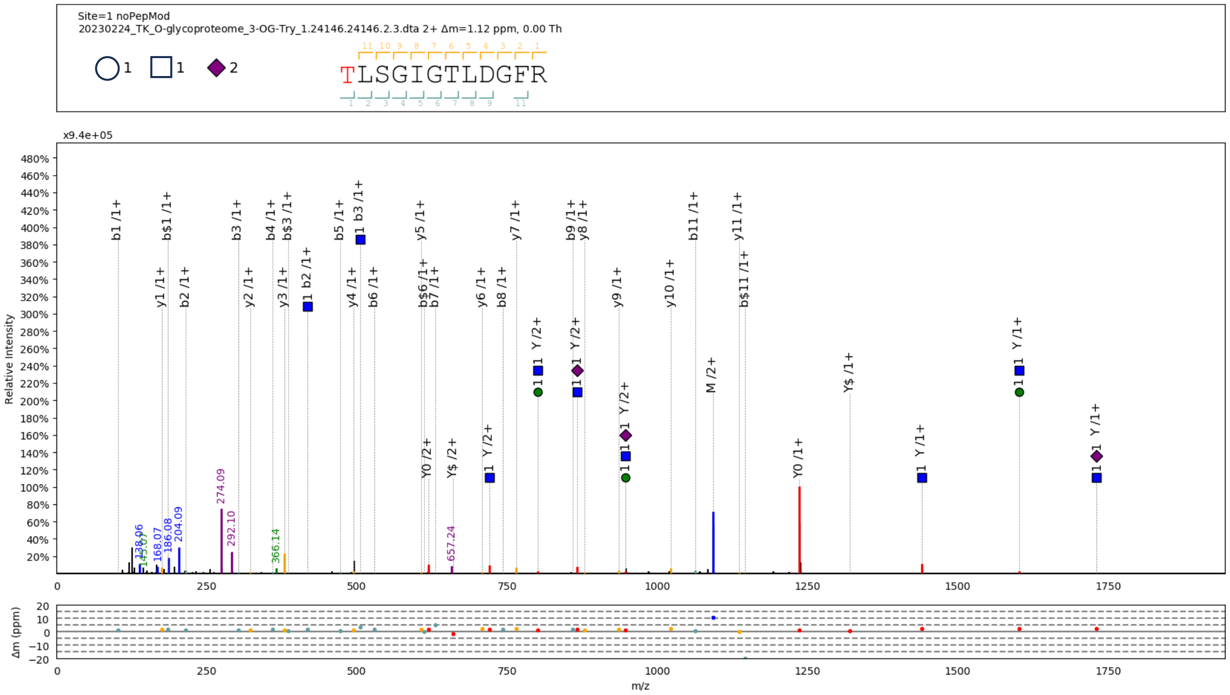
Site=1 noPepMod
20230224_TK_O-glycoproteome_3-OG-Try_1.24146.24146.2.3.dta 2+ Δm=1.12 ppm, 0.00 Th
1 1 2
TLSGIGTLDGFR
x9.4e+05
Relative Intensity
Δm (ppm)
m/z

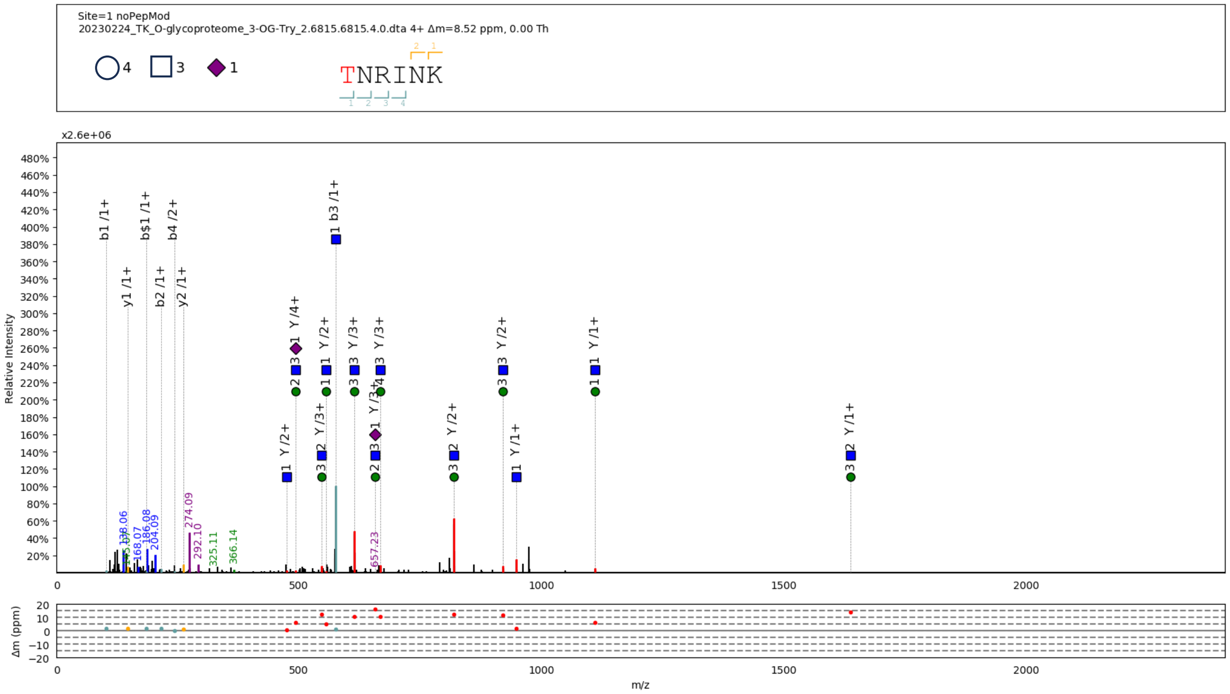
Site=1 noPepMod
20230224_TK_O-glycoproteome_3-OG-Try_2.6815.6815.4.0.dta 4+ Δm=8.52 ppm, 0.00 Th
4 3 1
TNRINK
x2.6e+06
Relative Intensity
Δm (ppm)
m/z

(E) Fibrinogen beta chain (FGB)

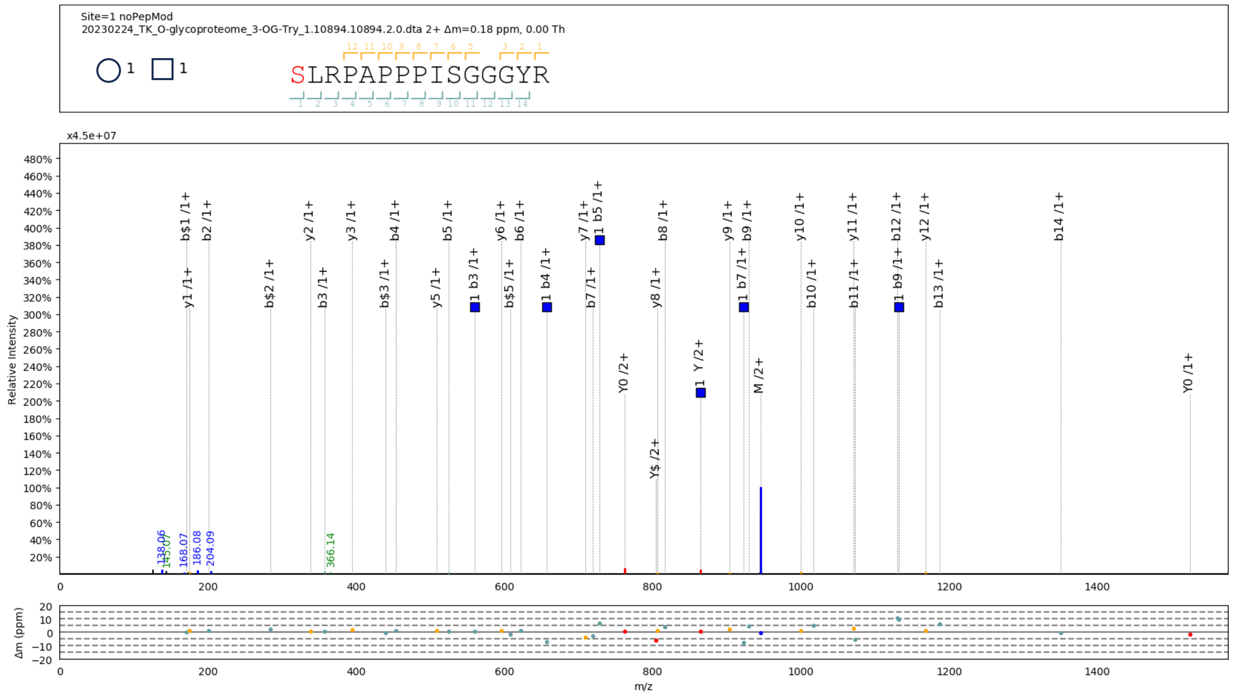

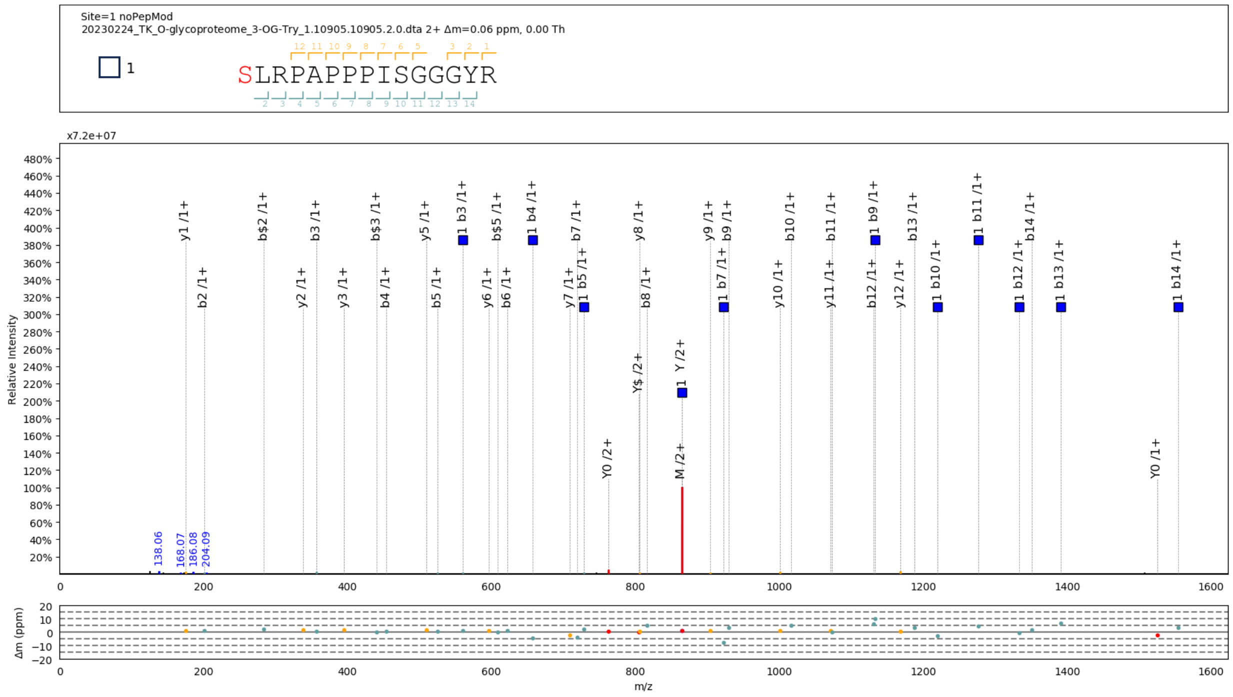

Supplement: Document S1. Figures S1 and S2 and Data S1 and S2 [file mmc1.pdf]
