# Supplementary material for: Global O-glycoproteome enrichment and analysis enabled by a combinatorial enzymatic workflow
Source: Cell Rep Methods. 2024 Apr 5;4(4):100744. doi: 10.1016/j.crmeth.2024.100744 (PMC11046030; doi:10.1016/j.crmeth.2024.100744)

Cell Reports
Methods

Article

Global O-glycoproteome enrichment and analysis enabled by a combinatorial enzymatic workflow

Graphical abstract

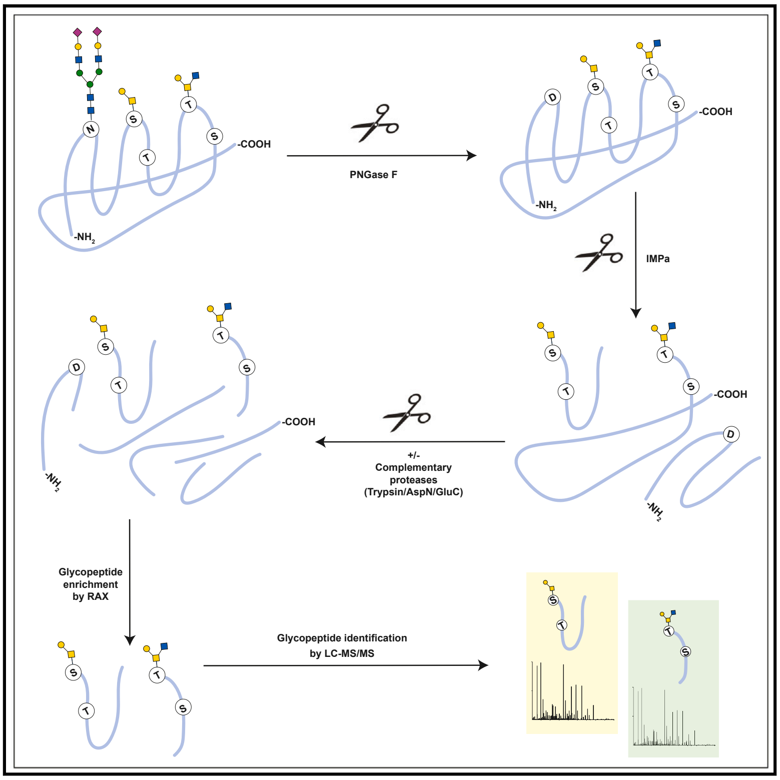

Highlights

- Four-step workflow enables identification of O-glycosylation sites and glycan composition
- A combinatorial enzymatic approach allows in-depth O-glycoproteome profiling
- Cleavage specificity and stepped collision energy aid site localization
- Identification of previously unreported sites expands the known O-glycoproteome

Authors

Taewook Kang, Rohit Budhraja, Jinyong Kim, Neha Joshi, Kishore Garapati, Akhilesh Pandey

Correspondence

pandey.akhilesh@mayo.edu

In brief

Kang et al. present a simple and efficient mass spectrometry-based method for in-depth O-glycoproteomics analysis of complex biological samples. This study used a multi-step combinatorial enzymatic approach, including an O-glycoprotease (IMPa), to profile the mucin-type O-glycoproteome in human plasma and plasma-purified glycoproteins.

Kang et al., 2024, Cell Reports Methods *4*, 100744
April 22, 2024 © 2024 The Author(s). Published by Elsevier Inc.
https://doi.org/10.1016/j.crmeth.2024.100744

CellPress

Cell Reports Methods

CellPress
OPEN ACCESS

Article

Global O-glycoproteome enrichment and analysis enabled by a combinatorial enzymatic workflow

Taewook Kang,[1,4] Rohit Budhraja,[1,4] Jinyong Kim,[1] Neha Joshi,[1,2] Kishore Garapati,[1,2] and Akhilesh Pandey[1,2,3,5,*]
[1]Department of Laboratory Medicine and Pathology, Mayo Clinic, Rochester, MN 55905, USA
[2]Manipal Academy of Higher Education, Manipal, Karnataka 576104, India
[3]Center for Individualized Medicine, Mayo Clinic, Rochester, MN 55905, USA
[4]These authors contributed equally
[5]Lead contact
*Correspondence: pandey.akhilesh@mayo.edu
https://doi.org/10.1016/j.crmeth.2024.100744

MOTIVATION O-linked glycosylation is critical to the function of most cell surface and secreted proteins. However, only a small fraction of O-glycosites have been cataloged across the human proteome, which impedes our understanding of such alterations in various pathologies including cancer and disorders of glycosylation. Through complementary use of proteases with the recently described O-glycoprotease IMPa, we have developed a combinatorial approach to identify many previously unknown O-glycosites. This method is broadly applicable for comprehensive characterization of O-glycoproteomes in biomedical research.

SUMMARY

A comprehensive analysis of site-specific protein O-glycosylation is hindered by the absence of a consensus O-glycosylation motif, the diversity of O-glycan structures, and the lack of a universal enzyme that cleaves attached O-glycans. Here, we report the development of a robust O-glycoproteomic workflow for analyzing complex biological samples by combining four different strategies: removal of N-glycans, complementary digestion using O-glycoprotease (IMPa) with/without another protease, glycopeptide enrichment, and mass spectrometry with fragmentation of glycopeptides using stepped collision energy. Using this workflow, we cataloged 474 O-glycopeptides on 189 O-glycosites derived from 79 O-glycoproteins from human plasma. These data revealed O-glycosylation of several abundant proteins that have not been previously reported. Because many of the proteins that contained unannotated O-glycosylation sites have been extensively studied, we wished to confirm glycosylation at these sites in a targeted fashion. Thus, we analyzed selected purified proteins (kininogen-1, fetuin-A, fibrinogen, apolipoprotein E, and plasminogen) in independent experiments and validated the previously unknown O-glycosites.

INTRODUCTION

Proteins can be post-translationally modified by the enzymatic addition of sugar chains to specific amino acid residues, most notably to the amide nitrogen of asparagine (N-linked glycosylation) and the hydroxyl oxygen of serine/threonine (O-linked glycosylation). O-glycosylation predominantly occurs in the extracellular regions of cell surface and secreted proteins via the O-linked N-acetylgalactosamine (O-GalNAc) glycan biosynthesis pathway.[1] Up to 83% of the proteins in Golgi-apparatus-related secretory pathways may be O-glycosylated with at least one O-GalNAc moiety.[2] O-glycosylation is classified as core 1 to core 8 based on the transfer of subsequent sugars to the initial O-GalNAc on the Ser/Thr residue.[3] Thus, glycosyltransferases extend each core structure in the Golgi stacks, and the mature glycan termini are capped with sialic acid and/or fucose in the *trans*-Golgi network.[4] Conversely, the N-glycosylation biosynthetic pathway is initiated in the endoplasmic reticulum by the synthesis of dolichol-linked donor substrates and their transfer to Asn residues in the Asn-X-Ser/Thr/Cys consensus sequence of proteins (X represents any amino acid except Pro).[5] Recognition of the consensus N-glycosylation motif enables the mapping of probable glycosylation site occupancy of intact glycopeptides or PNGase F digestion-induced previously glycosylated peptides (deamidation of Asn) with high reliability. However, no consensus O-glycosylation sequence has yet been identified. The absence of a consensus motif impedes probable O-glycosylation site characterization because of different degrees of occupancy within a protein, especially in highly O-glycosylated proteins containing mucin domains.[6] Despite extensive research on establishing the macroheterogeneity (site occupancy) of O-glycosylation, only a few approaches[7]

This is an open access article under the CC BY license (http://creativecommons.org/licenses/by/4.0/).

have been developed to effectively characterize intact O-glycopeptides. Thus, there is a need for improved strategies for global characterization of O-glycopeptides.

Recent advances in mass spectrometry (MS) have enabled large-scale characterization of glycopeptides and their glycan compositions. Tandem MS (MS/MS) is a key technique for breaking down intact glycopeptides into peptide, glycan, or glycan-containing peptide fragments. This enables the unambiguous localization of glycosites by matching specific glycosite-containing fragments. Two MS/MS dissociation methods, namely electron transfer dissociation (ETD) and high-energy collisional dissociation (HCD), are widely used for glycopeptide identification.[8,9] However, ETD-based MS/MS is less suitable for low-charge-density precursor ions such as O-glycopeptides.[10] HCD produces better peptide backbone fragmentation owing to higher collision energy; however, lower collisional energy is preferred for obtaining better information on glycan fragments, along with the production of glycan-specific oxonium ions from O-glycopeptides.[10,11] Therefore, a single MS/MS dissociation mode is unfavorable for the characterization of intact O-glycopeptides. Interestingly, stepped collision energy HCD (sceHCD) provides a single spectrum with information pertaining to the glycan compositions and peptide sequences of intact glycopeptides.[12] Additionally, recent advances in software tools enable the precise mapping of intact glycopeptides from the sceHCD-based MS/MS spectrum via false discovery rate (FDR) assessment at all three levels of matching, namely, glycans, peptides, and glycopeptides.[12,13] Moreover, alterations of O-glycan composition at the same site can impart unique functions to glycoproteins, thereby affecting the physiological and pathological processes associated with diseases. Differential expression of enzymes, such as glycosyltransferases and glycosidases, is most likely associated with aberrant O-glycosylation in diseases; however, this aspect of the disease microenvironment is not fully understood. In human carcinomas, the truncated GalNAc (Tn) and sialyl Tn antigen levels are elevated and have been highlighted as promising immunological targets for cancer therapy, including glycan-binding chimeric antigen receptor T cell therapy for tumors of the breast, colon, lung, pancreas, and skin.[14–16] Nonetheless, alterations of O-glycosylation levels in relation to pathophysiological molecular events are not known owing to a high degree of complexity due to macro- and microheterogeneities (diverse glycan structures on a single site).[17] This makes detection using liquid chromatography MS/MS (LC-MS/MS) difficult. Thus, additional strategies are required for more accurate O-glycopeptide characterization to enable the identification of unique sites and their glycan structures with high confidence.

For the development of a more complete approach, O-glycosylation-specific glycoproteases have emerged as “game changers” in O-glycoproteomics. They recognize and cleave O-glycosylated Ser/Thr residues, making it possible to pinpoint exact glycosites within O-glycoproteins. For instance, the O-endoprotease OgpA cleaves the peptide bond N terminus to core 1 O-glycans (consensus motif: X-S/T*-X′, where the asterisk indicates O-glycosylated Ser/Thr) but is unable to cleave other core structures including the Tn antigen or O-glycans containing sialic acid.[18] Further, the secreted protease of C1 esterase inhibitor (StcE) can selectively cleave the N terminus of O-glycosylated Ser/Thr (motif: S/T*-X-S/T) in densely glycosylated proteins containing mucin-like domains but shows restricted activity on non-mucin proteins.[6] In addition, zinc metalloendopeptidase (CpaA) cleaves only at the N-terminus of the O-glycosylated Ser/Thr residue that is preceded by Pro (motif: P-S/T*-X) in glycoproteins.[19] Recently, the immunomodulating metalloprotease IMPa was introduced and shown to cleave at the N terminus of O-glycosylated Ser/Thr (motif: X-S/T*-X′, X $\neq$ I/R/D) across all major types of O-glycan core structures regardless of the presence of sialic acids.[20] Thus, individual O-glycoproteases that can cleave a specific cleavage motif within O-glycoproteins add complementary tools for O-glycopeptide identification, and their cleavage properties allow interpretation of complex MS/MS spectra and identification of their exact glycosylation site. However, in any sample, non-glycopeptides and glycopeptides are mixed together; thus, effective enrichment of O-glycopeptides remains an essential step prior to LC-MS/MS analysis.

Selective enrichment of O-glycopeptides of interest from peptide mixtures is an important technique in O-glycoproteomic analysis. Several alternative chemical/biological affinity-based approaches are broadly used to achieve this, but they are far from perfect.[21] For example, lectin weak-affinity chromatography with *Vicia villosa* agglutinin is a selective lectin enrichment method shown to be amenable for simplified of O-GalNAc-glycopeptides,[22] zwitterionic hydrophilic interaction LC exploits the hydrophilicity of glycans,[23] electrostatic repulsion LC retains hydrophilic peptides with negative charges based on electrostatic interactions,[24] and the HyperSep Retain AX cartridge (RAX) based on strong anion exchange greatly enriches O-glycopeptides, with 75% of the enriched species containing oxonium ions.[25]

In the present study, we sought to develop a robust and reliable O-glycoproteomic profile workflow by implementing an MS-based O-glycoproteomic approach that reveals unreported global O-glycosylation events on different proteins. This workflow included O-glycosylation-specific protein digestion of plasma proteins with an additional enzyme for complementary cleavage specificity; enrichment of O-glycopeptides from complex peptide mixtures; identification of both the glycan structures and peptide sequences of intact O-glycopeptides; deciphering of annotations of matched peptides, glycans, or glycan-containing peptide fragment ions; and, finally, clarification of the macro- and microheterogeneities of O-glycopeptides using bioinformatics.

Additionally, we performed an in-depth profiling of both known and unknown O-glycosites in five well-known purified human plasma glycoproteins, namely kininogen-1, fetuin-A, fibrinogen, apolipoprotein E, and plasminogen, using our O-glycoproteomic approach. Overall, we believe that investigating the O-glycoproteome in a comprehensive fashion will provide a better understanding of the molecular features that mediate numerous physiological and pathological phenomena through a feasible workflow that opens new opportunities for identifying potential diagnostic, prognostic, and therapeutic biomarkers.

RESULTS

Experimental design

In this study, we aimed to develop a robust and reliable O-glycoproteomic profile workflow and achieve the long-standing goal

of glycobiology by implementing an attractive strategy using an MS-based O-glycoproteomics approach (Figure 1), revealing global O-glycosylation events on different proteins. O-glycosylation-specific protein digestion was performed in plasma and plasma-derived purified proteins using IMPa in combination with an additional enzyme with complementary cleavage specificity (trypsin, Asp-N, or Glu-C). To enrich O-glycopeptides in complex peptide mixtures, we applied a RAX-based enrichment method, and most O-glycopeptides were selectively enriched only in the elution fraction. We employed sceHCD-based MS/MS technology with Orbitrap MS to identify both the glycan structures and peptide sequences of intact O-glycopeptides. O-glycopeptides were deciphered with annotations of matched peptides, glycans, or glycan-containing peptide fragment ions based on FDR evaluation at all fragment levels using pGlyco3.[13]

Characterization of the plasma O-glycoproteome

Using our approach, from human plasma samples, we cataloged 474 O-glycopeptides on 253 peptide backbones derived from 79 proteins. These glycopeptides encompassed 37 glycan compositions on 189 unique O-glycosites (Figure 2A), which are listed in Table S1. IMPa, in conjunction with sceHCD, facilitates the localization of O-glycosites through selective cleavage at the N terminus of the O-glycosylation site (Figure S1A). IMPa accurately recognized and cleaved 95.2% of all cleavage events for the N terminus of the O-glycosylated Ser/Thr residue. Only 4.8% of all peptide-spectrum matches (PSMs) were not cleaved at the O-glycosites preceded by Ile, Arg, or Asp (X-I/R/D-S/T*-X) (Figure 2B). Remarkably, >99% of all O-glycoPSMs were effectively isolated from complex crude samples using RAX solid-phase extraction cartridges, whereas only 0.5% of O-glycoPSMs were identified in flow-through fractions, which majorly consisted of non-glycoPSMs (Figure 2C). RAX flow through comprised predominantly non-glycopeptides contributing to ion suppression of glycopeptides, which limited our capacity to identify the minority of glycosylated peptides. Non-glycoPSMs still existed in the RAX elution fractions (data not shown), but the yields of non-specifically bound non-glycopeptides from RAX SPE columns were markedly lower in the IMPa-only group (without complementary enzymes), as the generated peptides were larger than when IMPa was combined with other protease (Table S2). Collectively, our approach is highly advantageous for identifying O-glycopeptides from complex peptide mixtures in the presence of non-glycopeptides.

To enhance the number of O-glycopeptides identified, we performed combinatorial protein digestion of the base IMPa with complementary enzymes (trypsin, Asp-N, or Glu-C). As shown in Figure 2D, the number of O-glycosites and site-specific O-glycans identified was not strikingly different when IMPa was followed by trypsin, Asp-N, or Glu-C, and there were only 16 O-glycosites that overlapped across all enzyme combinations. We observed several unique O-glycosites while using different combinations of enzymes (Figure 2E). Thus, combinatorial protein digestion enabled us to explore a deep O-glycoproteome profiles in human plasma samples.

Mapping of densely O-glycosylated protein in plasma

In our global study, O-glycopeptides were generated from proteoglycan 4 (PRG4; also known as lubricin), which is a mucinous glycoprotein secreted in articular joints that was most abundantly detected, especially in the IMPa-only group. We cataloged 69 unique O-glycosites (66 T* and 3 S*) and 106 site-specific glycan compositions in PRG4. These identified O-glycosites were mostly localized to the region containing eight tandemly repeated amino acids (K-X-P-X-P-T-T-X) between the somatomedin B and hemopexin domains (Figure 3A). Interestingly, we observed 35 unique O-glycosites that were not reported in the UniProt and GlyGen databases and defined their reliable glycan compositions (Figure 3B). For instance, O-glycosylation with the sialyl T antigen of PRG4 at T213 was detected using a combination of IMPa and Glu-C/Asp-N (**T***PKPPVV**DE**) (Figure 3B). Otherwise, a typical digestion workflow with trypsin could produce a short peptide sequence (3 amino acids) or a long peptide backbone (41 amino acids) with IMPa-only digestion. In addition, a glycopeptide (**K**IT*TAKPINP**R**) containing O-glycosylation at T253 preceded by isoleucine was detected in concert with trypsin and IMPa digestion despite disadvantageous missed cleavage with IMPa. In addition, O-glycosylation at S263 (**S***LPPNSDTS**K**) was observed following combinatorial enzyme digestion. The quality of the annotated spectral data for all glycan structures of the O-glycosites was manually checked (Data S1).

Landscape of O-glycan core structures in plasma

Based on these findings, we further deciphered the distribution of O-glycan core structures. We found 37 unique O-glycan compositions on 189 unique O-glycosites. The details about these glycan compositions and plausible O-glycan structures (obtained from pGlyco) are provided in Table S1. In this study, 91.7% of the O-glycan forms were distinctively identified as core 1 and core 2 structures, while core 3 and core 4 together made up 2.6% (Figure 4A). Representative MS/MS spectra for glycopeptides containing different groups are shown in Figures S1B–S1E). We observed that the sialyl T antigen (30.8%) was the most abundant glycan. The next highest detected O-glycan form was the di-sialyl core 2 form (17.9%) (Figure S2).

Sequence homology of O-glycoproteins in plasma

We investigated the sequence homology of evolutionarily conserved O-glycoprotein domains in 79 O-glycoproteins (Figure 4B). We used the FASTA O-glycoprotein sequences retrieved from the UniProt database and aligned them using ClustalX2.1 with default parameters for multiple alignments and bootstrapping of the neighbor-joining tree.[26] O-glycoprotein sequences were visualized based on phylogenetic distances using the Interactive Tree of Life (https://itol.embl.de) tool. The related protein sequences were classified into six groups. Of these, a large proportion of clustered O-glycoproteins were included in group 6, and they generally contained mucin-like domains (Figure 4B).

O-glycosites and their glycan structures in human plasma-derived purified proteins

Using our workflow of performing combinatorial enzymatic O-glycoproteomics followed by RAX enrichment, we cataloged several previously unknown O-glycosites on plasma proteins. Many O-glycosites identified in our data have not been reported

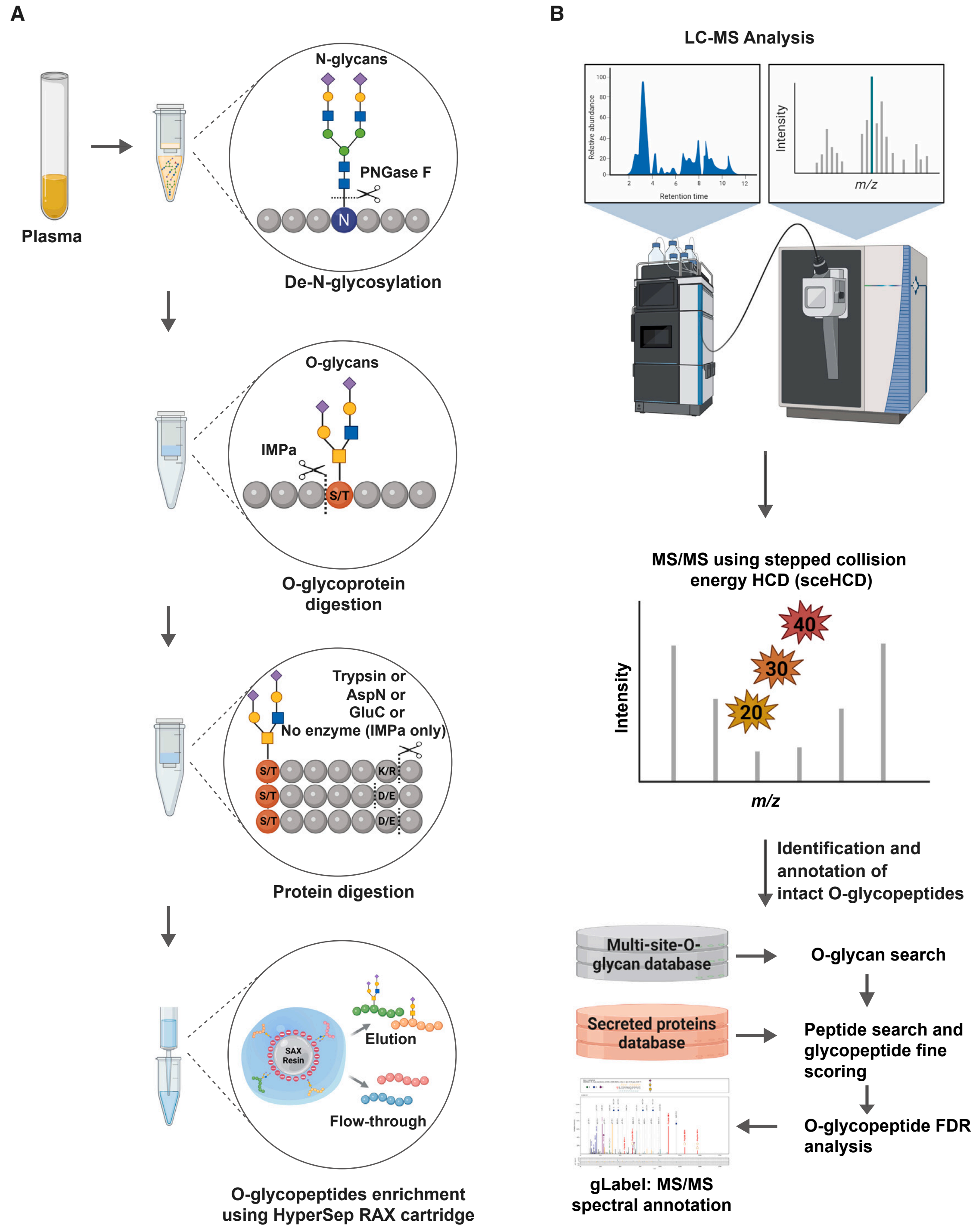

Figure 1. A schematic workflow depicting combinatorial enzymatic O-glycoproteomics

(A) Four-step experimental strategy for the enrichment of O-glycopeptides from plasma and purified proteins. Samples were first de-N-glycosylated using PNGase F (step 1), cleaved at the N terminus of O-glycosylation sites using IMPa (step 2), and separately digested with different proteolytic enzymes (step 3), and then the resulting peptides were enriched for O-glycopeptides by using RAX cartridges (step 4).

(B) Strategy illustrating identification of O-glycopeptides. Mass spectrometry analysis was done on an Orbitrap Exploris 480 mass spectrometer for enriched O-glycopeptides using stepped collision energy HCD. Identification and spectral annotation of O-glycopeptides was performed at FDR <1%.

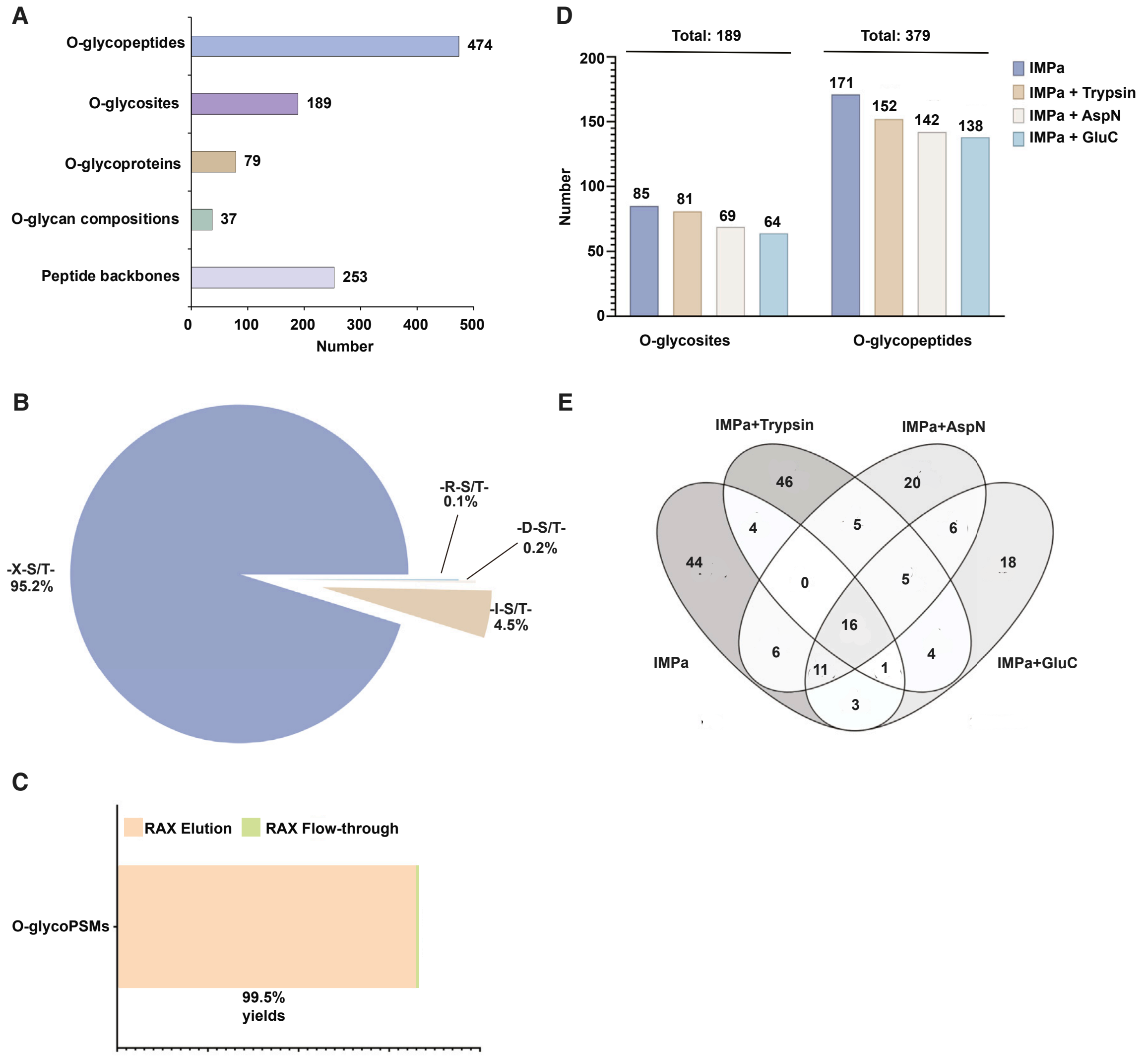

Figure 2. Global O-glycoproteomics study in human plasma sample

(A) A summary of the identified O-glycopeptides, O-glycosites, O-glycoproteins, O-glycan compositions, and peptide backbones in human plasma sample.
(B) O-glycoPSM yield in plasma sample with different cleavage sites. 95.2% of O-glycoPSMs were obtained with IMPa-specific cleavage sites: -X-S/T, where X is any amino acid except Ile, Arg, or Asp. The remaining 4.8% of O-glycoPSMs contained Ile, Arg, or Asp (I/R/D-S/T-) at the N terminus of O-glycosites.
(C) A bar diagram showing the high selectivity for O-glycoPSMs observed in RAX elution.
(D) Histograms showing the O-glycosites and site-specific glycan structures from different combinatorial digestion groups, respectively.
(E) Overlap of O-glycosites identified from four different combinatorial digestion groups as indicated.
See also Tables S1 and S2.

in the UniProt and GlyGen databases.[27] From our dataset, we selected a subset of five well-characterized O-glycoproteins (fetuin-A, AHSG; apolipoprotein E, APOE; fibrinogen A/B, FGA/FGB; kininogen-1, KNG1; and plasminogen, PLG) to validate our findings using purified protein preparations referred to as "targeted experiments." We confirmed previously unreported O-glycosites in these proteins, which was consistent with our global O-glycoproteomics analysis (Figure 5). We identified 11 O-glycosites for the fetuin-A protein from both global and targeted experiments, of which four O-glycosites have been reported in the Uniprot/GlyGen databases and seven were previously unreported (T158, T252, S256, S257, S328, S330, and T341) (Figure 5A). O-glycosylation at S328 was observed in both experiments. For fibrinogen B, there is no report of O-glycosylation in UniProt/GlyGen databases. However, we confirmed a previously unknown O-glycosylation at S58 from both our global and targeted experiments (Figure 5B). For fibrinogen A, only two O-glycosites have been reported in the UniProt/GlyGen databases, although we were able to identify 13 previously unknown O-glycosites (T82, T275, T279, T357, S417, T435, T484, T499, T522, T525, S551, S560, and S562) using both global and targeted experiments (Figure 5C). Two

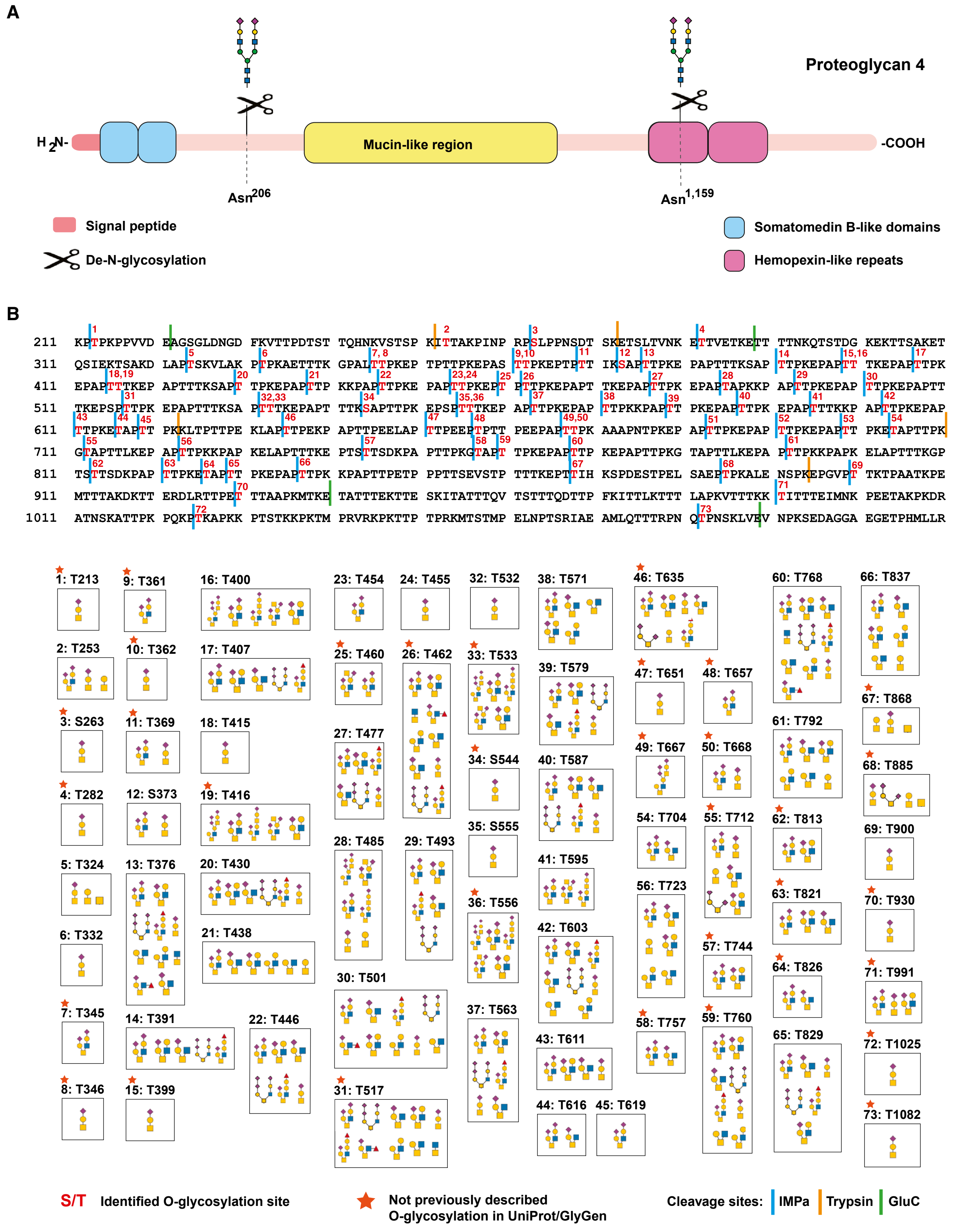

(legend on next page)

CellPress
OPEN ACCESS

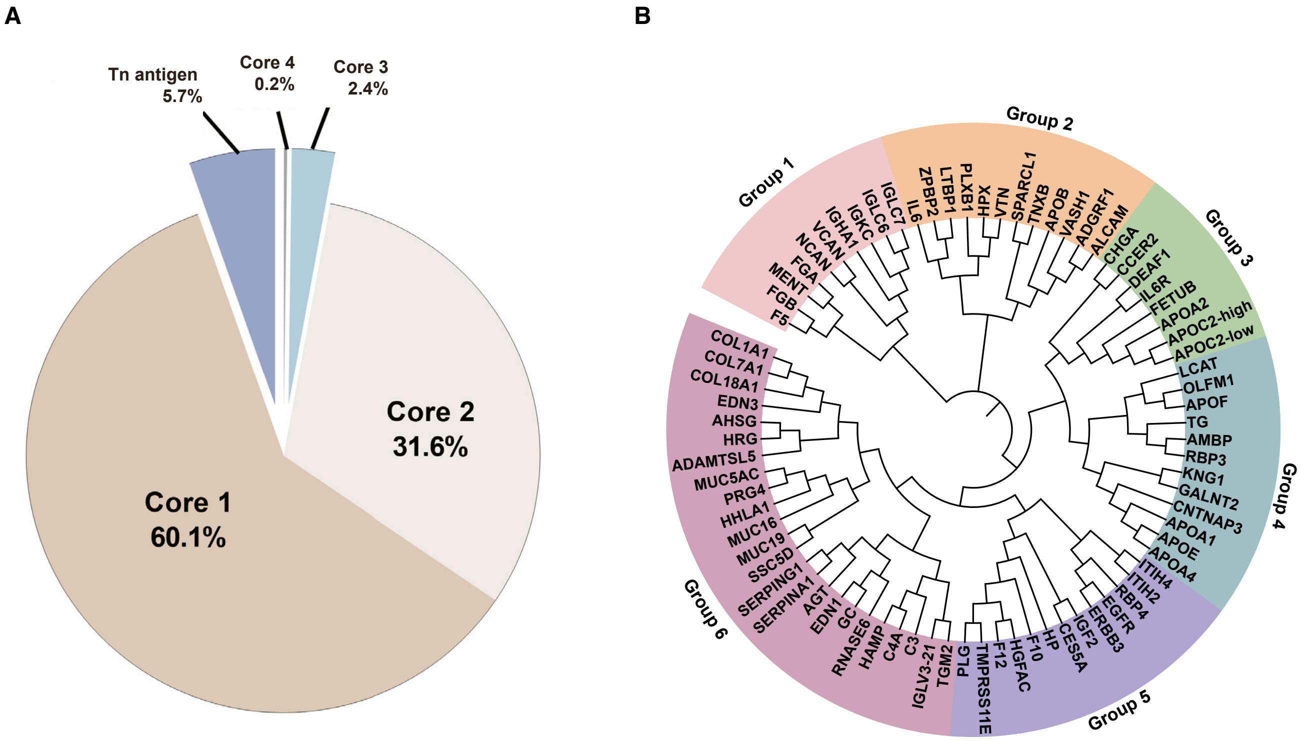

Figure 4. Diversity of O-glycan cores and O-glycoproteome in plasma

(A) Relative proportions of 474 unique O-glycopeptides in human plasma. Venn diagram representing the relative distribution of O-glycopeptides with major O-glycan core structures.

(B) Sequence homology analysis of cataloged 79 O-glycoproteins. The rims indicate groups 1–6 by color bars according to the homology of the protein sequences.

O-glycosylation sites at T525 and S562 were observed in both global and targeted experiments. For kininogen, eight O-glycosites have been reported in the UniProt/GlyGen databases. However, our data showed eight previously unreported O-glycosites (T151, T273, S390, T400, S408, T521, T532, and T538) among the 14 identified sites using both our global and targeted experiments (Figure 5D). In total, six O-glycosites have been reported in UniProt/GlyGen databases for APOE protein, and we identified five known O-glycosites (T26, T36, T212, T307, and S308) using both global and targeted experiments (Figure 5E). Two previously unknown O-glycosylations at T151 and S408 were confirmed by both O-glycoproteomics results. Finally, for plasminogen, we observed five O-glycosites by using both global and targeted methods, of which four sites (S355, TT457, S460, and T710) have not been reported previously (Figure 5F). All spectra pertaining to the five purified proteins from human plasma are included in Data S2.

DISCUSSION

To capture and identify unique O-glycosites and their specific O-glycan structures, we designed a four-step workflow (Figure 1A). First, prior to protein digestion, N-glycosites were deglycosylated on membrane filters using PNGase F with a 10 kDa molecular weight cutoff (MWCO) (first image in Figure 1A). Both types of diverse N- and O-glycopeptide mixtures may cause computational and analytical complexities in defining glycan, peptide, or glycan-containing peptide ions in glycopeptide spectra involving a biased interpretation of the glycosites and its glycan compositions, such as micro- and macroheterogeneity. Additionally, low-abundance O-glycopeptides are inherently hampered by the physicochemical characteristics and heterogeneity of N-glycopeptides in SAX-based enrichment procedures and high-throughput MS analyses. Thus, N-glycans were first eliminated from glycoproteins (although they could be

Figure 3. Identification of O-glycosites and their glycan compositions on proteoglycan 4 (PRG4) protein in plasma sample

(A) A schematic representation of the domain structure of PRG4 protein with somatomedin B domain marked (SMB; blue) along with mucin-like region (yellow) and hemopexin-like repeats (pink). The known N-glycosylation sites on two different asparagine residues are also represented.

(B) Effective complementary enzyme digestion for identifying densely O-glycosylated PRG4 protein. Different O-glycan compositions are represented on identified O-glycosites on the full sequence of the protein. Structures of glycans are shown with symbols drawn according to the Symbol Nomenclature for Glycans (SNFG).

See also Data S1.

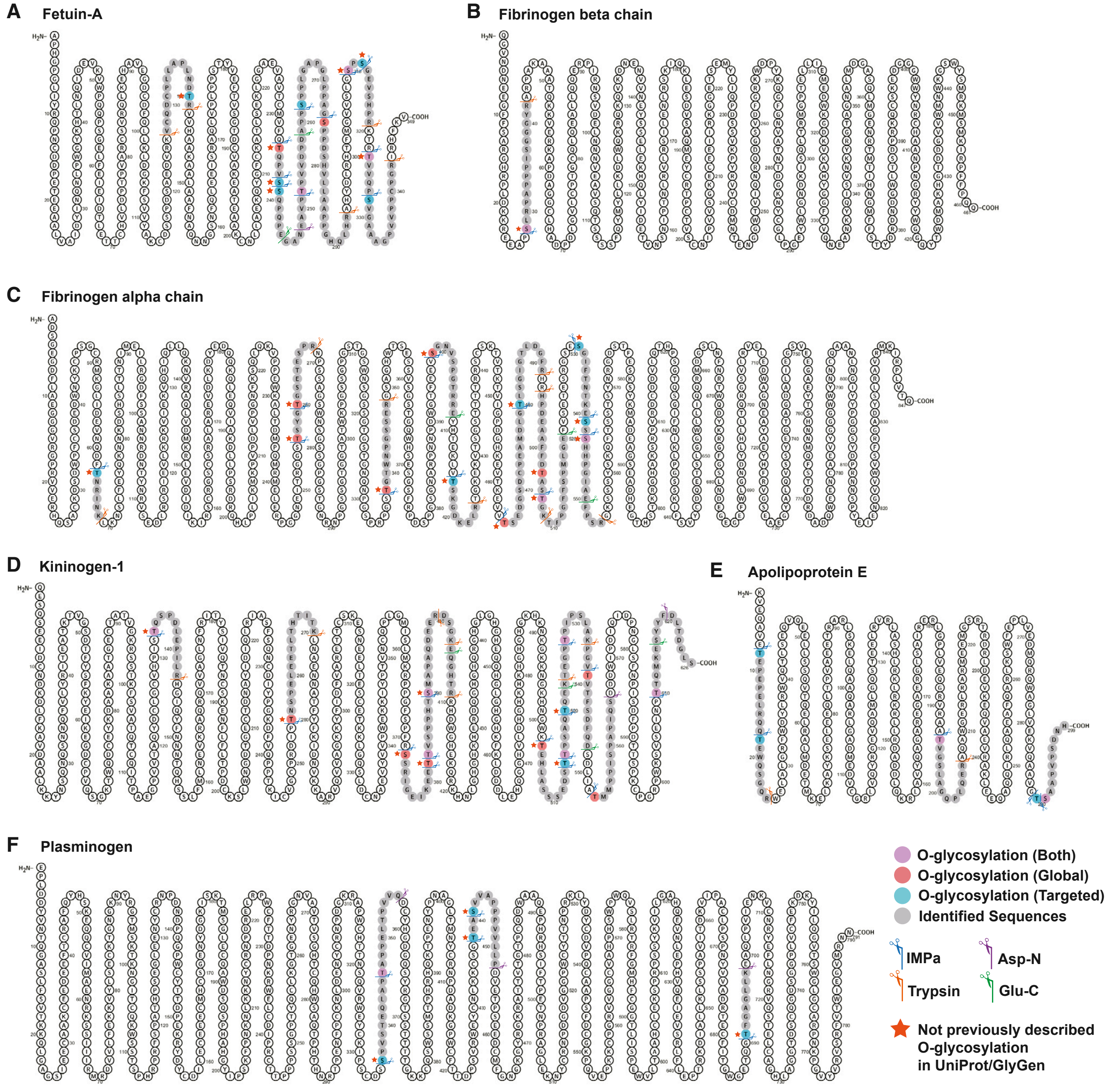

Figure 5. Mapping of human plasma-derived purified proteins

(A) Fetuin A (AHSG), (B) fibrinogen beta chain (FGB), (C) fibrinogen alpha chain (FGA), (D) kininogen-1 (KNG1), (E) apolipoprotein E (APOE), and (F) plasminogen (PLG). N termini are marked with the color-coded scissors for O-glycosites identified by either global plasma profiling or plasma-derived, or both, approaches. These sites contain both previously reported and previously not described O-glycosites.

See also Data S2.

used for potential exploration of the N-glycome). Formerly N-glycosylated proteins (deamidation of Asn) remained on the filter because of the retention capabilities of membrane filtration with a 10 kDa MWCO. Second, O-glycopeptides were generated from the resulting protein mix using only IMPa (Figure 1A). Peptides generated from digestion with IMPa were separately collected from the membrane filters without secondary breakdown of O-glycopeptides in three replicates. Third, the digested O-glycopeptides and non-glycopeptides were cleaved using a different proteolytic enzyme with complementary cleavage specificity (trypsin, Asp-N, or Glu-C) in six replicates (third image in Figure 1A). Trypsin, Asp-N, and Glu-C cleave the C terminus of

K/R, the N terminus of D/E, and the C terminus of D/E, respectively. Fourth, an anion-exchange chromatographic RAX column was used for enrichment of intact O-glycopeptides that contributed to the discrimination between O-glycopeptides and non-glycopeptides (fourth image in Figure 1A). Next, we analyzed the enriched intact O-glycopeptides using high-resolution nanoLC-based Orbitrap MS in sceHCD-MS/MS acquisition mode using 20%, 30%, and 40% normalized collision energies. sceHCD-MS/MS was utilized for identifying the production of oxonium ions, peptide fragments, and glycan-containing peptide fragments, which corroborate the introduction of intact O-glycopeptide characterization. In our protocol, we employed the pGlyco3 search engine that was optimized for glycopeptide identification in sceHCD-MS/MS spectra and searched against the human secreted protein sequence database (from UniProtKB-Swiss-Prot) and multi-site-O-glycan database (from pGlyco3); eventually, FDR analysis was used for evaluation at the individual search level (FDR $<$ 1%) (bottom in Figure 1B). Importantly, the method for identifying intact O-glycopeptides comprises four criteria: (1) correlating peptide sequences using the peptide fragments, (2) determining glycan masses by calculating the mass difference between the peptide backbone and intact O-glycopeptide precursor, (3) localizing the glycosites by matching-specific glycosite-containing fragments; and (4) characterizing glycan structures using oxonium ions and glycan-containing peptide fragment ions. These were adopted by pGlyco3 and manually confirmed using the gLabel software tool.[12]

In this study, we introduce and demonstrate the potential utility of our workflow, which provides a robust approach to identify unknown O-glycosylation and complex glycan forms. The translation of O-glycoproteomics to clinical settings remains challenging; thus, identifying potential molecular biomarkers is crucial and will require biomedical studies to adopt O-glycoproteomics approaches on a larger scale in the future. This can be achieved by continued efforts to optimize methods for global or targeted quantitative O-glycoproteomics.

In summary, we performed large-scale and targeted bioinformatics analyses of O-glycoproteins using our complementary enzymatic and enrichment strategy, thereby identifying 189 unique O-glycosites carrying 474 O-glycopeptides on 253 unique peptide backbones of 79 O-glycoproteins. Further, we confirmed our findings in global plasma O-glycoproteomics through the analysis of five proteins purified from human plasma that showed better results than those of complex crude samples, thereby confirming previously unknown glycosites. O-glycoprotease IMPa demonstrates the feasibility of characterizing diverse O-glycosylated peptide substrates including mucinous proteins, and the SAX-based RAX column also contributes to improved yields of O-glycopeptides. Although the exact function of most of the identified O-glycosylation events has not been investigated, our high-throughput characterization of O-glycopeptides has provided in-depth profiling of O-glycosites and their O-glycan structures. Importantly, alteration of glycan compositions is essential for a better understanding of molecular mechanisms that contribute to the pathogenesis of many different diseases such as severe acute respiratory syndrome coronavirus 2, cancers, nephropathy, autoimmune disease, osteoarthritis, and Alzheimer's disease. We anticipate that this strategy will be widely adopted to discover potential diagnostic, prognostic, or therapeutic biomarkers at glycan, glycopeptide, and/or glycosite levels in the future.

Limitations of the study

IMPa does not cleave the C terminus to the amino acids isoleucine, arginine, and aspartate. Thus, this method would not confidently identify glycosylation sites that are affected by these factors. Other approaches need to be developed for relative quantitation of glycosylation profiles on specific sites. Further, additional studies are needed to characterize other purified proteins from which we identified glycosylation sites in our global plasma O-glycoproteomics experiment.

STAR★METHODS

Detailed methods are provided in the online version of this paper and include the following:

- KEY RESOURCES TABLE
- RESOURCE AVAILABILITY
  - Lead contact
  - Materials availability
  - Data and code availability
- EXPERIMENTAL MODEL AND STUDY PARTICIPANT DETAILS
  - Human plasma samples
- METHOD DETAILS
  - De-N-glycosylation
  - Sequential and Combinational Protein digestion
  - Enrichment of O-glycopeptides
  - Reversed-phase nanoLC-MS/MS
  - MS Data processing
- QUANTIFICATION AND STATISTICAL ANALYSIS

SUPPLEMENTAL INFORMATION

Supplemental information can be found online at https://doi.org/10.1016/j.crmeth.2024.100744.

ACKNOWLEDGMENTS

This work was supported in part by grants from NCI to A.P. (U01CA271410 and P30CA15083).

AUTHOR CONTRIBUTIONS

Conceptualization, T.K., R.B., and A.P.; methodology, T.K., R.B., J.K., N.J., and A.P.; software, T.K. and R.B.; formal analysis, T.K., R.B., J.K., N.J., and K.G.; investigation, T.K., R.B., and A.P.; data curation, T.K., R.B., J.K., N.J., and K.G.; writing – original draft, T.K., R.B., J.K., N.J., and K.G.; writing – review & editing, T.K., R.B., and A.P.; visualization, J.K. and N.J.; resources, A.P.; supervision, A.P.; funding acquisition, A.P.

DECLARATION OF INTERESTS

The authors declare no competing interests

Received: September 22, 2023
Revised: January 25, 2024

Accepted: March 18, 2024
Published: April 5, 2024

REFERENCES

1. Bagdonaite, I., Pallesen, E.M.H., Nielsen, M.I., Bennett, E.P., and Wandall, H.H. (2021). Correction to: Mucin-Type O-GalNAc Glycosylation in Health and Disease. Adv. Exp. Med. Biol. *1325*, C1–C2.
2. Steentoft, C., Vakhrushev, S.Y., Joshi, H.J., Kong, Y., Vester-Christensen, M.B., Schjoldager, K.T.B.G., Lavrsen, K., Dabelsteen, S., Pedersen, N.B., Marcos-Silva, L., et al. (2013). Precision mapping of the human O-GalNAc glycoproteome through SimpleCell technology. EMBO J. *32*, 1478–1488.
3. Bennett, E.P., Mandel, U., Clausen, H., Gerken, T.A., Fritz, T.A., and Tabak, L.A. (2012). Control of mucin-type O-glycosylation: a classification of the polypeptide GalNAc-transferase gene family. Glycobiology *22*, 736–756.
4. Wandall, H.H., Nielsen, M.A.I., King-Smith, S., de Haan, N., and Bagdonaite, I. (2021). Global functions of O-glycosylation: promises and challenges in O-glycobiology. FEBS J. *288*, 7183–7212.
5. Helenius, J., Ng, D.T.W., Marolda, C.L., Walter, P., Valvano, M.A., and Aebi, M. (2002). Translocation of lipid-linked oligosaccharides across the ER membrane requires Rft1 protein. Nature *415*, 447–450.
6. Malaker, S.A., Pedram, K., Ferracane, M.J., Bensing, B.A., Krishnan, V., Pett, C., Yu, J., Woods, E.C., Kramer, J.R., Westerlind, U., et al. (2019). The mucin-selective protease StcE enables molecular and functional analysis of human cancer-associated mucins. Proc. Natl. Acad. Sci. USA *116*, 7278–7287.
7. Bagdonaite, I., Malaker, S.A., Polasky, D.A., Riley, N.M., Schjoldager, K., Vakhrushev, S.Y., Halim, A., Aoki-Kinoshita, K.F., Nesvizhskii, A.I., Bertozzi, C.R., et al. (2022). Glycoproteomics. Nat Rev Method Prime *2*, 48.
8. Thaysen-Andersen, M., Wilkinson, B.L., Payne, R.J., and Packer, N.H. (2011). Site-specific characterisation of densely O-glycosylated mucin-type peptides using electron transfer dissociation ESI-MS/MS. Electrophoresis *32*, 3536–3545.
9. Kawahara, R., Chernykh, A., Alagesan, K., Bern, M., Cao, W., Chalkley, R.J., Cheng, K., Choo, M.S., Edwards, N., Goldman, R., et al. (2021). Community evaluation of glycoproteomics informatics solutions reveals high-performance search strategies for serum glycopeptide analysis. Nat. Methods *18*, 1304–1316.
10. Riley, N.M., Malaker, S.A., Driessen, M.D., and Bertozzi, C.R. (2020). Optimal Dissociation Methods Differ for N- and O-Glycopeptides. J. Proteome Res. *19*, 3286–3301.
11. Cao, L., Tolić, N., Qu, Y., Meng, D., Zhao, R., Zhang, Q., Moore, R.J., Zink, E.M., Lipton, M.S., Paša-Tolić, L., and Wu, S. (2014). Characterization of intact N- and O-linked glycopeptides using higher energy collisional dissociation. Anal. Biochem. *452*, 96–102.
12. Liu, M.Q., Zeng, W.F., Fang, P., Cao, W.Q., Liu, C., Yan, G.Q., Zhang, Y., Peng, C., Wu, J.Q., Zhang, X.J., et al. (2017). pGlyco 2.0 enables precision N-glycoproteomics with comprehensive quality control and one-step mass spectrometry for intact glycopeptide identification. Nat. Commun. *8*, 438.
13. Zeng, W.F., Cao, W.Q., Liu, M.Q., He, S.M., and Yang, P.Y. (2021). Precise, fast and comprehensive analysis of intact glycopeptides and modified glycans with pGlyco3. Nat. Methods *18*, 1515–1523.
14. Romer, T.B., Aasted, M.K.M., Dabelsteen, S., Groen, A., Schnabel, J., Tan, E., Pedersen, J.W., Haue, A.D., and Wandall, H.H. (2021). Mapping of truncated O-glycans in cancers of epithelial and non-epithelial origin. Br. J. Cancer *125*, 1239–1250.
15. Raglow, Z., McKenna, M.K., Bonifant, C.L., Wang, W., Pasca di Magliano, M., Stadlmann, J., Penninger, J.M., Cummings, R.D., Brenner, M.K., and Markovitz, D.M. (2022). Targeting glycans for CAR therapy: The advent of sweet CARs. Mol. Ther. *30*, 2881–2890.
16. Fernandes, Â., Azevedo, C.M., Silva, M.C., Faria, G., Dantas, C.S., Vicente, M.M., and Pinho, S.S. (2023). Glycans as shapers of tumour microenvironment: A sweet driver of T-cell-mediated anti-tumour immune response. Immunology *168*, 217–232.
17. Mariño, K., Bones, J., Kattla, J.J., and Rudd, P.M. (2010). A systematic approach to protein glycosylation analysis: a path through the maze. Nat. Chem. Biol. *6*, 713–723.
18. Trastoy, B., Naegeli, A., Anso, I., Sjögren, J., and Guerin, M.E. (2020). Structural basis of mammalian mucin processing by the human gut O-glycopeptidase OgpA from Akkermansia muciniphila. Nat. Commun. *11*, 4844.
19. Haurat, M.F., Scott, N.E., Di Venanzio, G., Lopez, J., Pluvinage, B., Boraston, A.B., Ferracane, M.J., and Feldman, M.F. (2020). The Glycoprotease CpaA Secreted by Medically Relevant Acinetobacter Species Targets Multiple O-Linked Host Glycoproteins. mBio *11*, e02033-20.
20. Vainauskas, S., Guntz, H., McLeod, E., McClung, C., Ruse, C., Shi, X., and Taron, C.H. (2022). A Broad-Specificity O-Glycoprotease That Enables Improved Analysis of Glycoproteins and Glycopeptides Containing Intact Complex O-Glycans. Anal. Chem. *94*, 1060–1069.
21. Riley, N.M., Bertozzi, C.R., and Pitteri, S.J. (2021). A Pragmatic Guide to Enrichment Strategies for Mass Spectrometry-Based Glycoproteomics. Mol. Cell. Proteomics *20*, 100029.
22. Schjoldager, K.T.B.G., Vakhrushev, S.Y., Kong, Y., Steentoft, C., Nudelman, A.S., Pedersen, N.B., Wandall, H.H., Mandel, U., Bennett, E.P., Levery, S.B., and Clausen, H. (2012). Probing isoform-specific functions of polypeptide GalNAc-transferases using zinc finger nuclease glycoengineered SimpleCells. Proc. Natl. Acad. Sci. USA *109*, 9893–9898.
23. Scott, N.E., Parker, B.L., Connolly, A.M., Paulech, J., Edwards, A.V.G., Crossett, B., Falconer, L., Kolarich, D., Djordjevic, S.P., Højrup, P., et al. (2011). Simultaneous glycan-peptide characterization using hydrophilic interaction chromatography and parallel fragmentation by CID, higher energy collisional dissociation, and electron transfer dissociation MS applied to the N-linked glycoproteome of Campylobacter jejuni. Mol. Cell. Proteomics *10*, M000031–MCP201.
24. Darula, Z., Sherman, J., and Medzihradszky, K.F. (2012). How to dig deeper? Improved enrichment methods for mucin core-1 type glycopeptides. Mol. Cell. Proteomics *11*, 0111016774.
25. Yang, W., Shah, P., Hu, Y., Toghi Eshghi, S., Sun, S., Liu, Y., and Zhang, H. (2017). Comparison of Enrichment Methods for Intact N- and O-Linked Glycopeptides Using Strong Anion Exchange and Hydrophilic Interaction Liquid Chromatography. Anal. Chem. *89*, 11193–11197.
26. Kang, T., Boland, B.B., Jensen, P., Alarcon, C., Nawrocki, A., Grimsby, J.S., Rhodes, C.J., and Larsen, M.R. (2020). Characterization of Signaling Pathways Associated with Pancreatic beta-cell Adaptive Flexibility in Compensation of Obesity-linked Diabetes in db/db Mice. Mol. Cell. Proteomics *19*, 971–993.
27. York, W.S., Mazumder, R., Ranzinger, R., Edwards, N., Kahsay, R., Aoki-Kinoshita, K.F., Campbell, M.P., Cummings, R.D., Feizi, T., Martin, M., et al. (2020). GlyGen: Computational and Informatics Resources for Glycoscience. Glycobiology *30*, 72–73.

CellPress
OPEN ACCESS

STAR★METHODS

KEY RESOURCES TABLE

REAGENT or RESOURCE	SOURCE	IDENTIFIER
Biological samples		
Normal human plasma	Mayo Clinic	IRB19-004317
Chemicals, peptides, and recombinant proteins		
Acetonitrile HPLC grade	Merck	75-05-8
Dithiothreitol	Sigma	D9163
Iodoacetamide	Sigma	I1149
HEPES	Sigma	H3375
Trifluoroacetic acid	Sigma	302031
Formic acid	Fisher chemical	A117
Native Human AHSG protein	abcam	ab93727
Native Human APOE Protein	Abnova	P4281
Native Human Fibrinogen Protein (Plasminogen-Depleted)	Sigma	341578
Native Human KNG1 Protein	abcam	ab90353
Native Human PLG Protein	abcam	ab77879
Endoproteinase GluC	New England Biolabs	P8100S
Endoproteinase AspN	New England Biolabs	P8104S
PNGase F	New England Biolabs	P0704S
Trypsin/Lys-C Mix, Mass Spec Grade	Promega	V5071
O-Glycoprotease (IMPa)	New England Biolabs	P0761S
Software and algorithms		
Xcalibur	Thermo Scientific	RRID:SCR_014593
Adobe Illustrator	Adobe	RRID:SCR_010279
Biorender	Biorender.com	RRID:SCR_018361
GraphPad Prism	GraphPad software	RRID:SCR_000306
pGlyco3	http://pfind.org/software/pGlyco/index.html	N/A
Deposited data		
ProteomeXchange Consortium via the PRIDE partner repository	This paper	PXD042701
Other		
HyperSep™ Retain AX Cartridges	Thermo Scientific	60107–401
Amicon Ultra-0.5 Centrifugal Filter Unit	Millipore	UFC501096
Orbitrap Exploris 480	Thermo Scientific	BRE725533
Vanquish HPLC and UHPLC system	Thermo Scientific	VN-S10-A-01
50 cm µPAC micro pillar array column	Thermo Scientific	COL-NANO050G1B

RESOURCE AVAILABILITY

Lead contact

Further information and requests for resources and reagents should be directed to and will be fulfilled by the lead contact, Akhilesh Pandey (Pandey.Akhilesh@Mayo.edu).

Materials availability

This study did not generate any new materials or reagents.

Data and code availability

- The mass spectrometry proteomics data have been deposited with the ProteomeXchange Consortium via the PRIDE partner repository with the dataset identifier PXD042701.
- This study does not report original code.
- Any additional information required to reanalyze the data reported in this paper is available from the lead contact upon request.

EXPERIMENTAL MODEL AND STUDY PARTICIPANT DETAILS

Human plasma samples

Pooled plasma, obtained by pooling samples from fifty individuals, was used for experiments described in this study. Specific information on age and sex is not included as it was not available. This study was approved by the Institutional Review Board at Mayo Clinic (approval number IRB19-004317).

METHOD DETAILS

De-N-glycosylation

First, we performed de-N-glycosylation of human plasma using PNGase F enzyme according to manufacturer's instruction. Briefly, 2.5 μL of plasma samples and commercially available human plasma-derived purified proteins were denatured with 3 μL of 10X Denaturing buffer (New England BioLabs) and 24.5 μL of distilled water (DW) was added to make a 30 μL total reaction volume. Samples were incubated at 99°C for 10 min in a heating block, and then cooled down at room temperature (RT) for 10 min. Supernatants were transferred to Amicon Ultra-10 kDa MWCO (Millipore). 6 μL of 10X GlycoBuffer 2 (New England BioLabs) and 24 μL of DW were added to MWCO to make a total reaction volume of 60 μL. 2 μL of PNGase F was added and incubated at 37°C for 4 h. After deglycosylation, released N-glycan mixture was collected by centrifugation at 14,000×g for 15 min. To fully remove denaturation buffer, 400 μL of 100 mM HEPES buffer (pH 7.8) was added and washed by centrifugation at 14,000×g for 15 min at least three times.

Sequential and Combinational Protein digestion

After de-N-glycosylation, IMPa was added to the samples (1:20, w/w) and incubated overnight at 37°C. The sample was then split into different tubes and digested with trypsin, Asp-N, or Glu-C separately (1:50, w/w) at 37°C. After this proteolysis, the peptides were extracted by centrifuging the filters at 14,000 g for 15 min and lyophilized prior to enrichment of O-glycopeptides.

Enrichment of O-glycopeptides

The dried peptide mixture was dissolved in 400 μL of loading buffer (95% acetonitrile (ACN), 1% trifluoroacetic acid (TFA)) for glycopeptide enrichment using RAX cartridge. The RAX cartridge was equilibrated in 1 mL of 100% ACN for three times, 100 mM triethylammonium acetate for three times, DW for three times, and subsequently loading buffer for three times. The peptide mixture was loaded and collected the flow-through (unbound fraction) in a new tube. The cartridges were washed with loading buffer for three times. Finally, bound O-glycopeptides were eluted in 400 μL of elution buffer (50% ACN, 0.1% TFA). The eluate was dried in speed-vac and stored at −80°C before until LC-MS/MS analysis.

Reversed-phase nanoLC-MS/MS

All samples were redissolved in solvent A (0.2% formic acid, 0.05% TFA) and analyzed using Orbitrap Exploris 480 mass spectrometer (MS) (Thermo Fisher Scientific) coupled with Vanquish UHPLC System (Thermo Fisher Scientific). The samples were first loaded onto a 1.6 cm trap column (100 μm inner diameter) and then separated on a 50 cm μPAC micro pillar array column (PharmaFluidics, Belgium). The peptides were eluted over 60 min gradient from 7 to 38% solvent B (80% ACN, 10% isopropanol, 0.1% formic acid) at a flow rate of 300 nL/min and introduced into the MS instrument via nano electrospray. A full MS scan in the range of 400–1400 m/z was performed in the Orbitrap with a resolution of 120,000, an AGC target value of 1×10^4, and a maximum injection time of 50 ms. For each full scan, "Top speed" mode was selected for sceHCD. The settings for the sceHCD were as follows: AGC target value of 3×10^4, maximum injection time of 190 ms, isolation window of 1.2 Da, and normalized collision energy of 20, 30, and 40%.

MS Data processing

The raw MS data were processed for intact O-glycopeptide identification using pGlyco3 with a precursor mass tolerance of 10 ppm, a fragment mass tolerance of 20 ppm, and an FDR of 1% for glycopeptides. All peak lists were searched against the secretome UniProtKB/Swiss-Prot database (03/2023, 3,863 entries) of human sequences with decoy and the Multi-site O-glycan database using the parameters as follows: enzyme specificities IMPa, trypsin, Asp-N, or Glu-C; maximum missed cleavages, 2; fixed modification, carbamidomethylation (C); variable modifications, oxidation (M), phosphorylation (S,T,Y), and deamidation (N). The MS/MS spectra were manually examined for glycan compositions, peptide, and glycan-containing peptide fragments, providing the probable glycosite localization and glycan structures.

QUANTIFICATION AND STATISTICAL ANALYSIS

Data analysis was performed using pGlyco3 as described in the section above. Since the study was performed as a qualitative study using a pooled sample, there was no quantitation or statistical analysis performed in this study.

Cell Reports Methods, Volume *4*

Supplemental information

Global O-glycoproteome enrichment and analysis enabled by a combinatorial enzymatic workflow

Taewook Kang, Rohit Budhraja, Jinyong Kim, Neha Joshi, Kishore Garapati, and Akhilesh Pandey

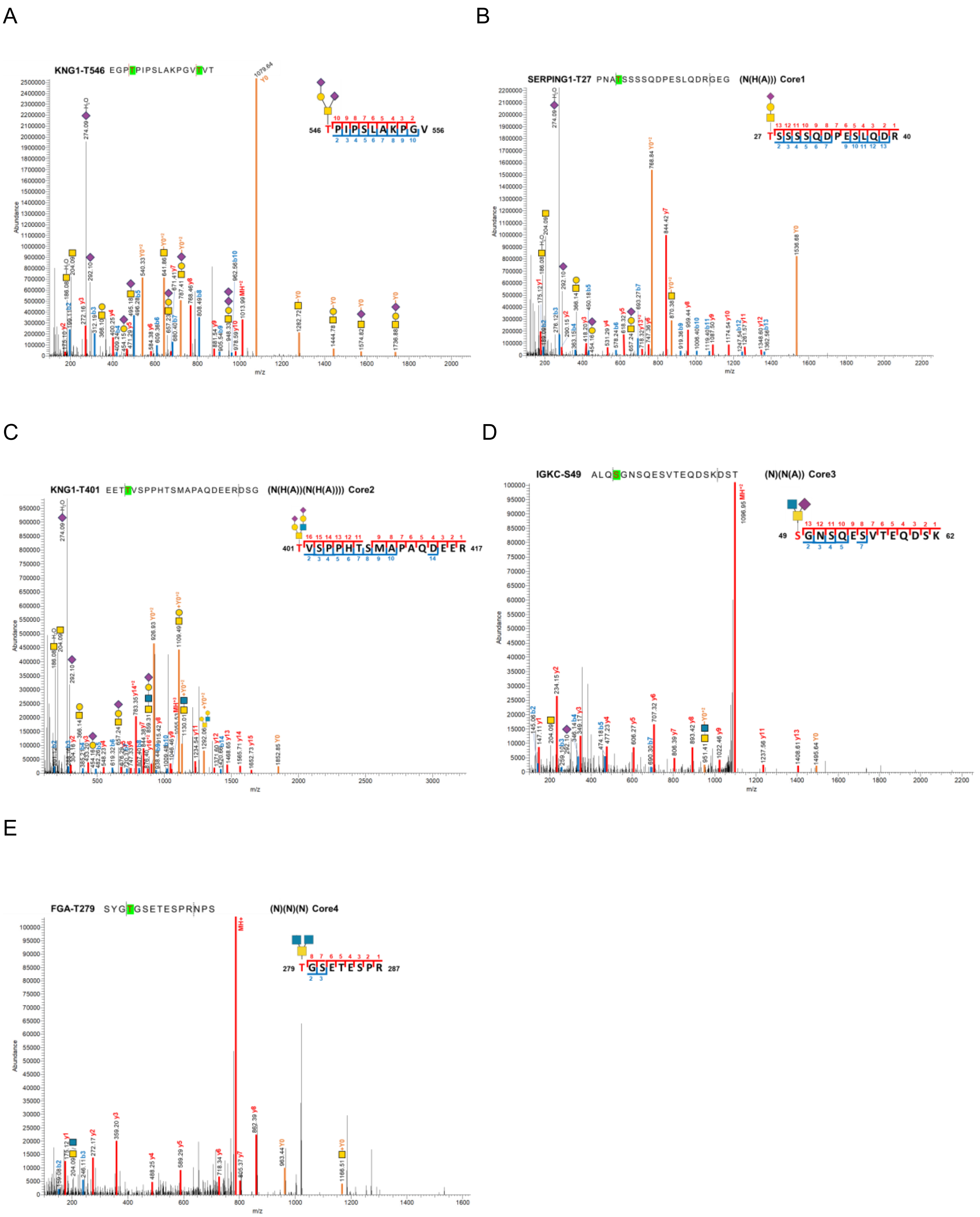

Figure S1 related to figure 4 and STAR methods. Representative MS/MS spectra of a glycopeptide showing (A) the use of sceHCD and the specificity of IMPa approach to localize the O-glycosylation site, (B) Core 1 structure, (C) Core 2 structure, (D) Core 1 structure, (E) Core 2 structure.

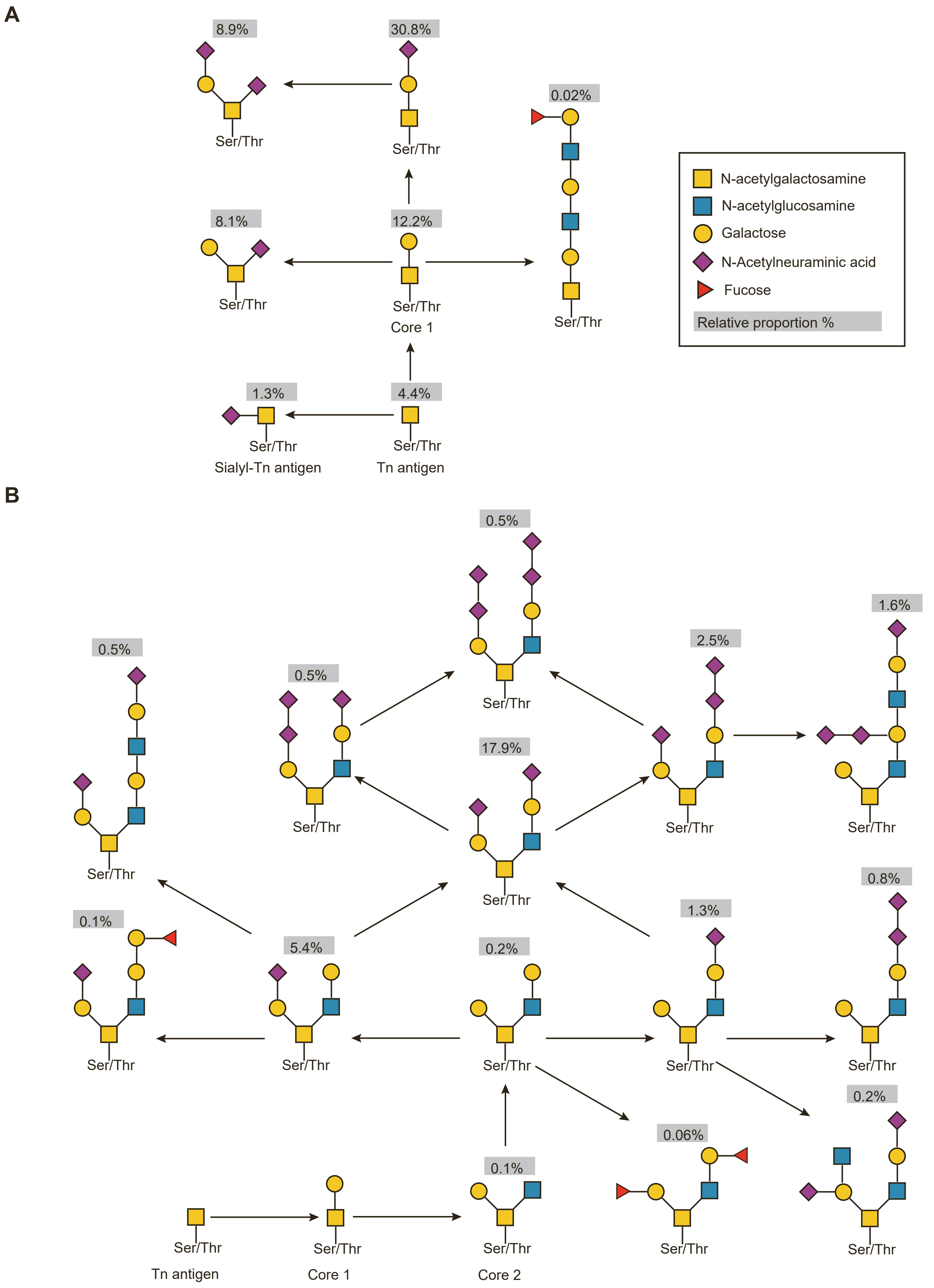

Figure S2 related to figure 4. Relative proportions of identified unique O-glycan structures. (A) Illustration of identified core 1 glycan structures. (B) Illustration of identified core 2 glycan structures

Data S1. MS/MS Spectra of identified O-glycopeptides in proteoglycan 4 (PRG4), related to Figure 3.

MS/MS spectra of identified O-glycopeptides in proteoglycan 4 (PRG4)

```
DOMAIN          26-69   Somatomedin B (SMB) 1
DOMAIN         66-108   Somatomedin B (SMB) 2
REGION        348-855   59 X 8 AA repeats of K-X-P-X-P-T-T-X
REPEAT      1148-1191   Hemopexin (PEX) 1
REPEAT      1192-1239   Hemopexin (PEX) 2
```

S/T *O*-glycosylation

X Identified sequences

KP**T**PKPPVV**D** **E**AGSGLDNGD FKVTTPDTST

TQHNKVSTSP **K**I**T**TAKPINP RP**S**LPPNSDT

S**K**ETSLTVNK E**T**TVETK**E**TT TTNKQTSTDG

KEKTTSAKET QSIEKTSAKD LAP**T**SKVLAK

P**T**PKAETTTK GPAL**TT**PKEP TPTTPKEPAS

TTPKEPTP**T**T IK**S**AP**T**TPKE PAPTTTKSAP

TTPKEPAP**TT** TKEPAP**T**TPK EPAP**TT**TKEP

APTTTKSAP**T** TPKEPAPTTP KKPAP**T**TPKE

PAP**TT**PKEP**T**	P**T**TPKEPAPT	TKEPAP**T**TPK
EPAPTAPKKP	AP**T**TPKEPAP	**T**TPKEPAPTT
TKEPSP**T**TPK	EPAPTTTKSA	P**TT**TKEPAPT
TTK**S**APTTPK	EPSP**TT**TKEP	AP**T**TPKEPAP
TTPKKPAP**T**T	PKEPAP**T**TPK	EPAP**T**TTKKP
AP**T**TPKEPAP	**T**TPKETAP**T**T	PKKLTP**T**TPE
KLAP**T**TPEKP	APTTPEELAP	**T**TPEEP**T**PTT
PEEPAP**TT**PK	AAAPNTPKEP	AP**T**TPKEPAP
**T**TPKEPAP**T**T	PKETAPTTPK	G**T**APTTLKEP
AP**T**TPKKPAP	**KE**LAPTTTKE	PTS**T**TSDKPA
P**T**TPKG**T**AP**T**	TPKEPAP**T**TP	KEPAPTTPKG
TAPTTLKEPA	P**T**TPKKPAP**K**	**E**LAPTTTKGP
TS**T**TSDKPAP	**T**TPKE**T**AP**T**T	PKEPAP**T**TPK

KPAPTTPETP PPTTSEVSTP TTTKEPT**T**IH

KSPD**ES**TPEL SAEP**T**PKAL**E** NSP**K**EPGVP**T**

TKTPAA**T**KPE M**T**TTAKDKTT **E**RDLRTTPE**T**

TTAAPKMTK**E** TATTTEKTTE SKITATTTQV

TSTTTQDTTP FKITTLKTTT LAPKVTTTKK

TITTTEIMNK PEETAKP**K**DR ATNSKATTPK

PQKP**T**KAPKK PTS**T**KKPKTM PRVRKPKTTP

TPRKMTSTMP ELNPTSRIAE AMLQTTTRPN

Q**T**PNSKLV**E**V

PRG4−T213, H (1) N (1) A (1)

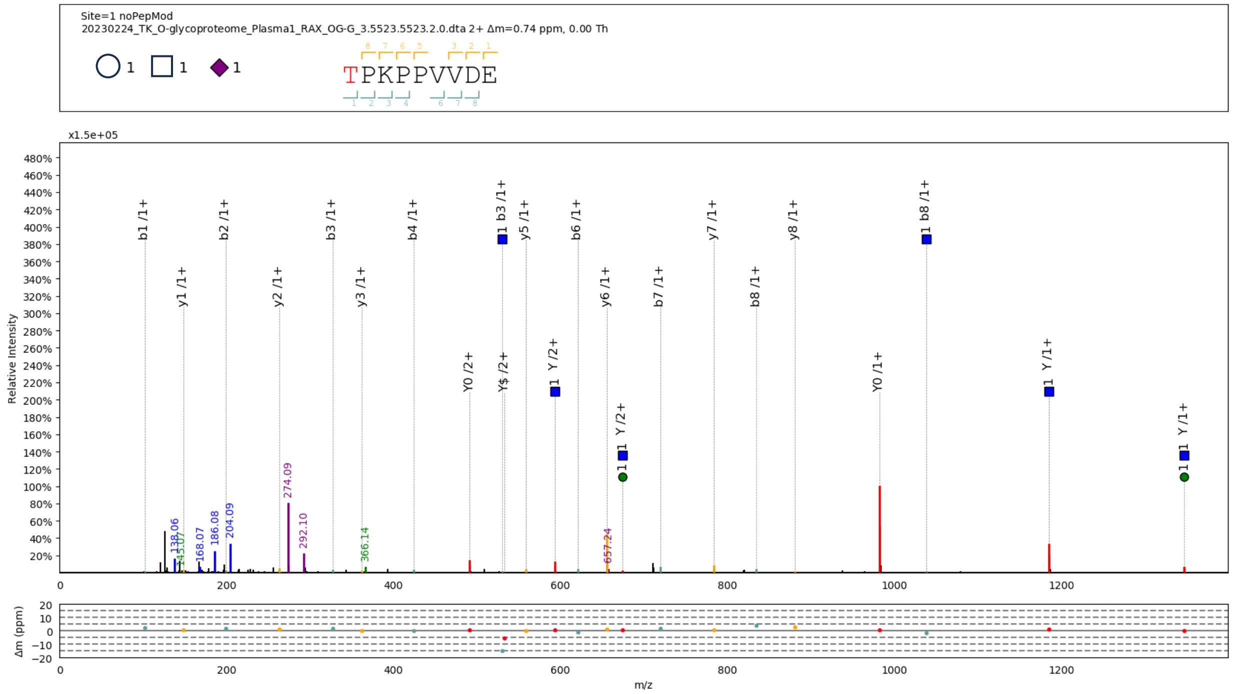

PRG4−T253, H (2) N (2) A (2)

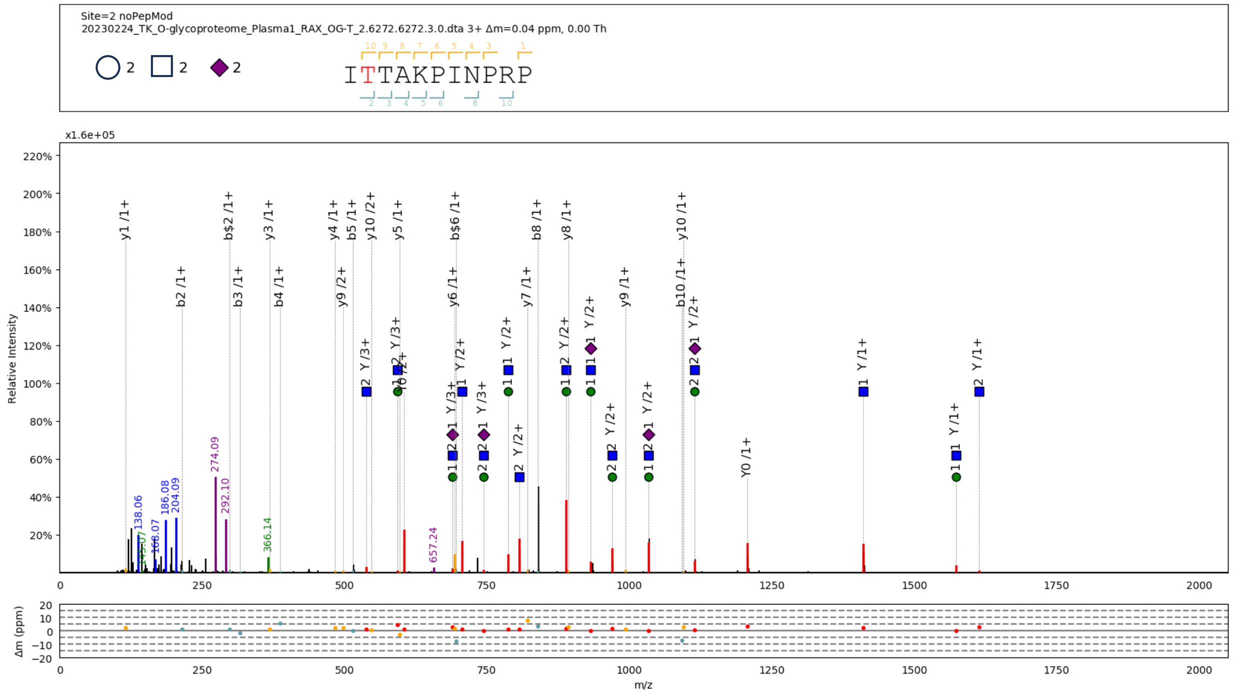

PRG4–T253, H (1) N (1) A (1)

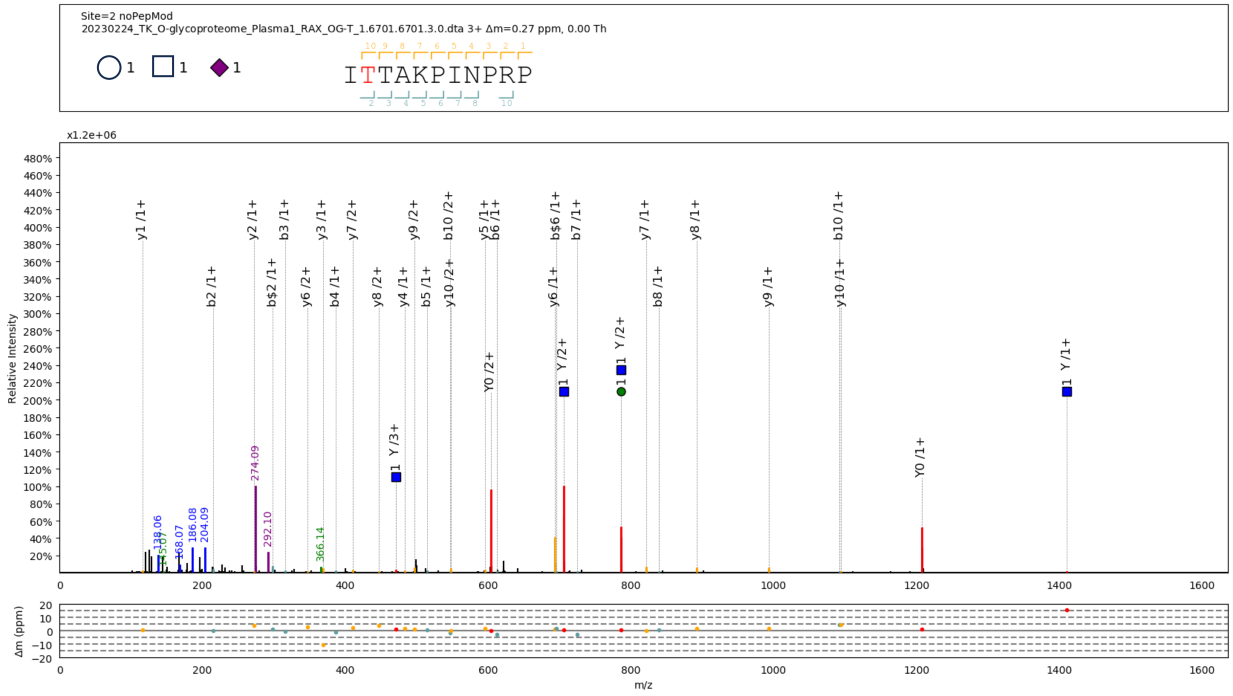

PRG4–T253, H (1) N (1)

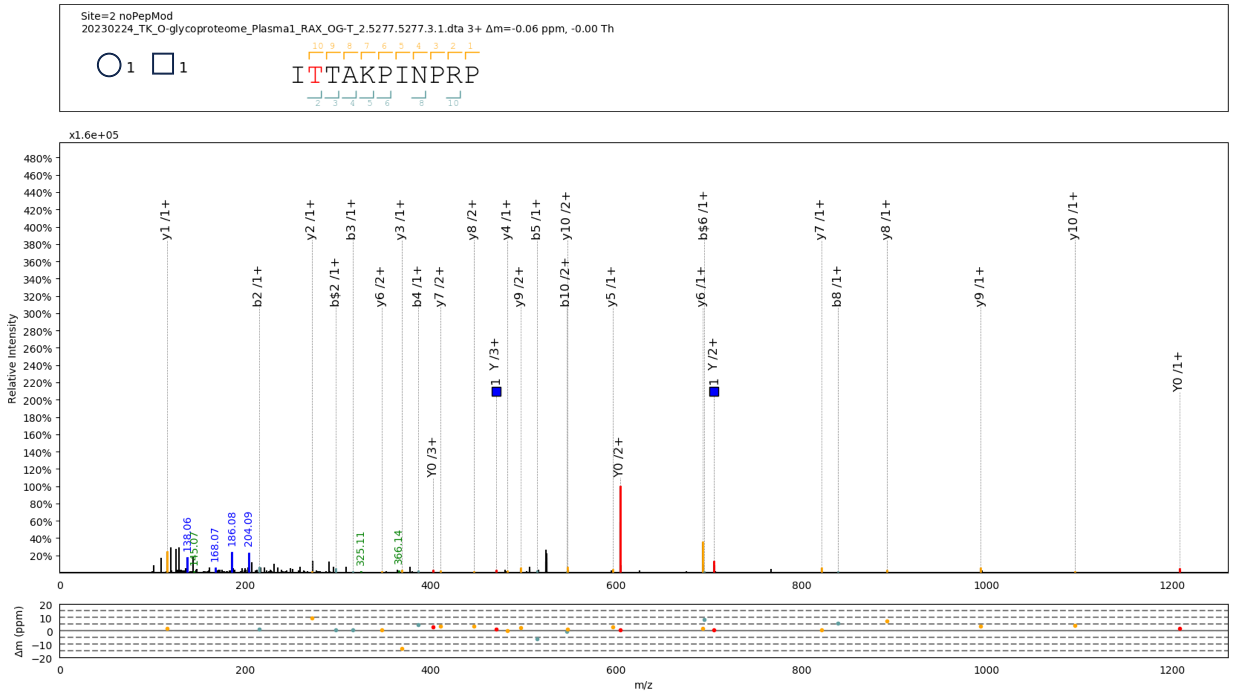

PRG4–S263, H (1) N (1) A (1)

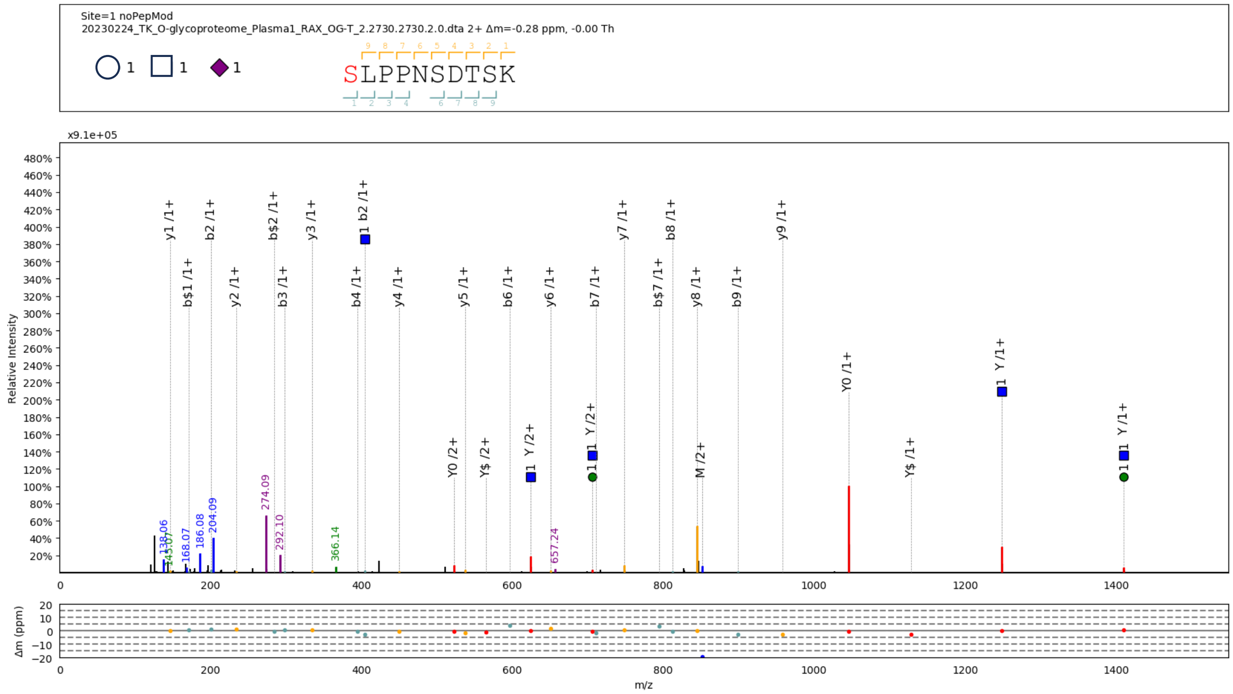

PRG4–T282, H (1) N (1) A (1)

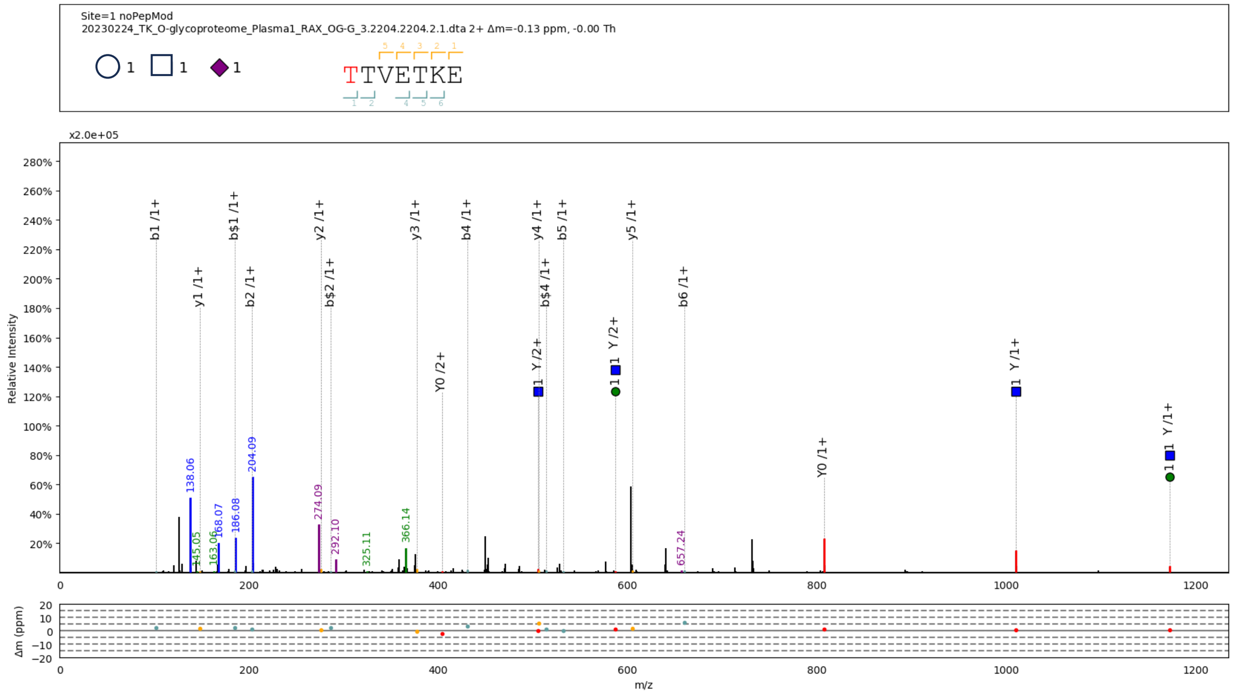

PRG4–T324, H (1) N (1) A (1)

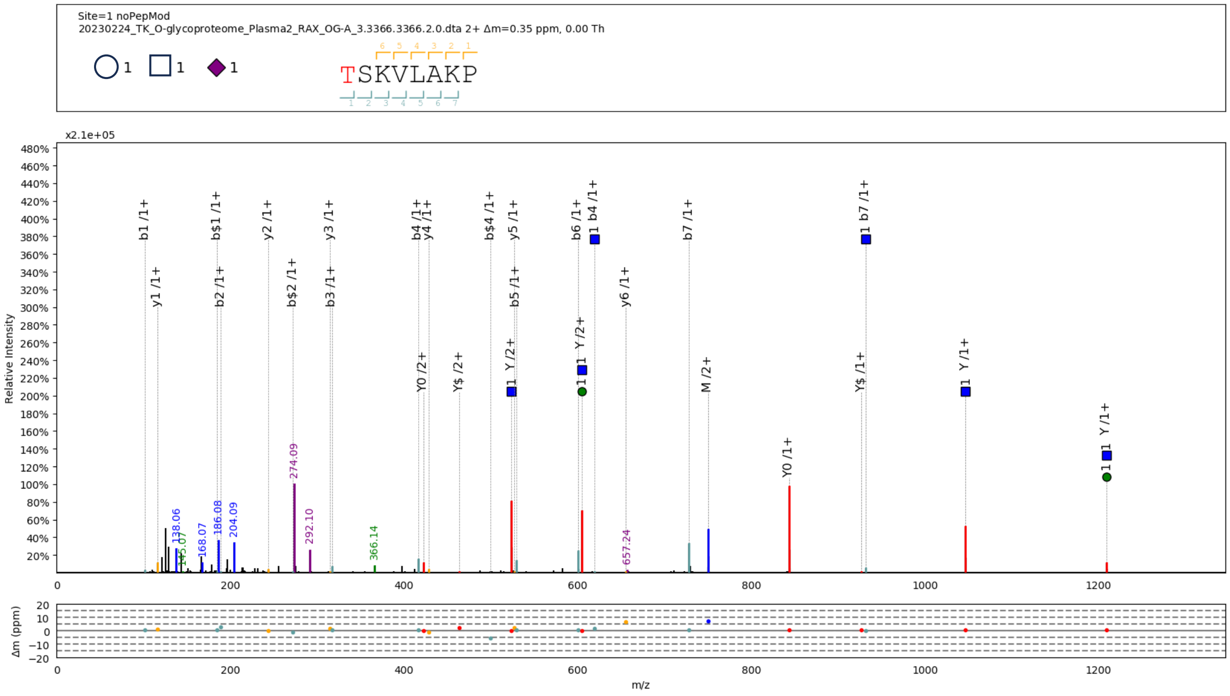

PRG4–T324, H (1) N (1)

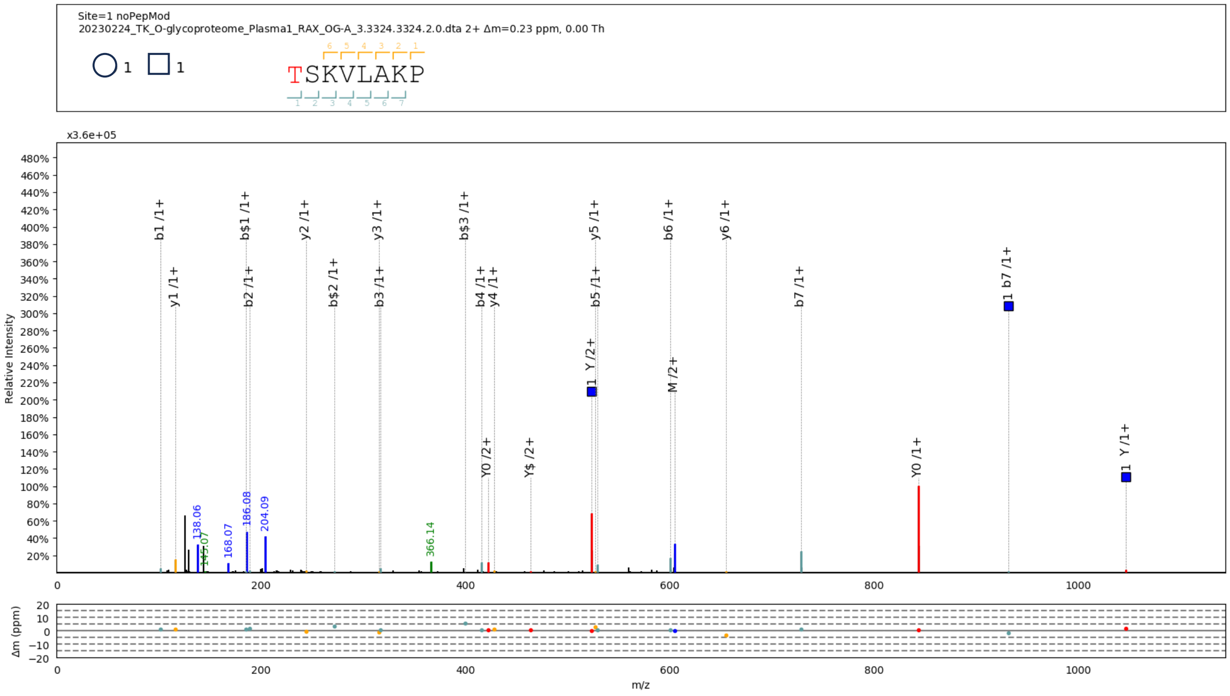

PRG4−T324, N (1)

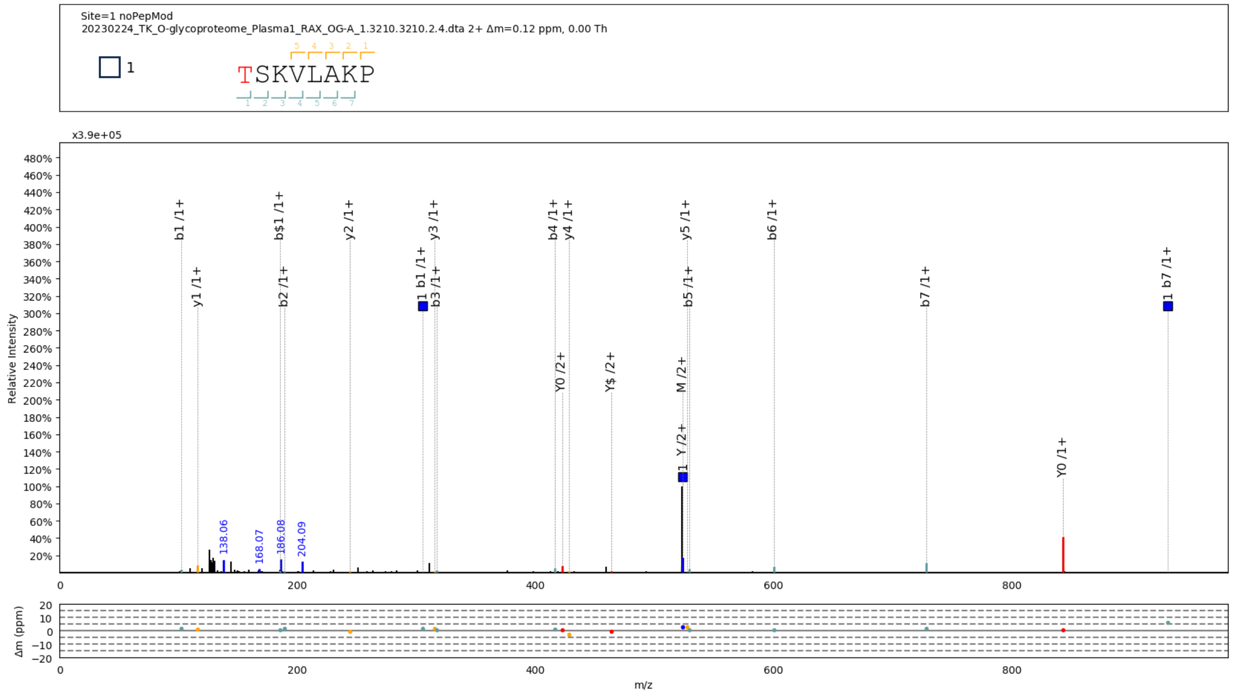

PRG4−T332, H (1) N (1) A (1)

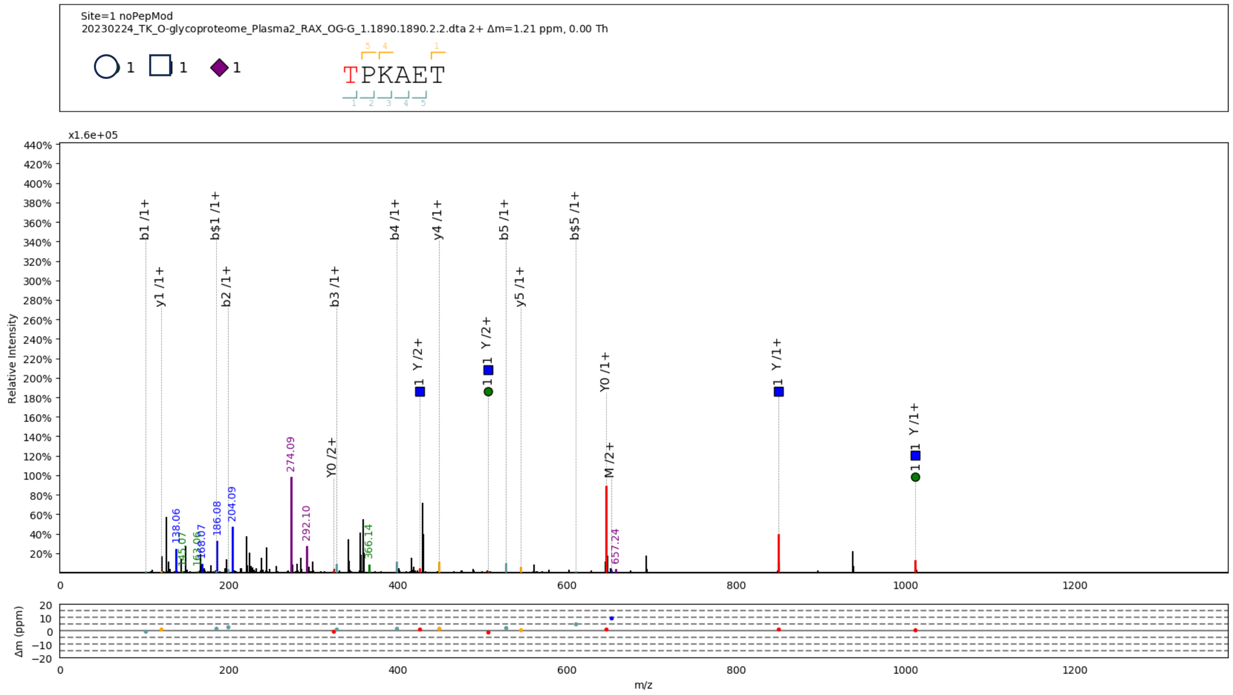

PRG4–T345-T361-T454, H (2) N (2) A (2)

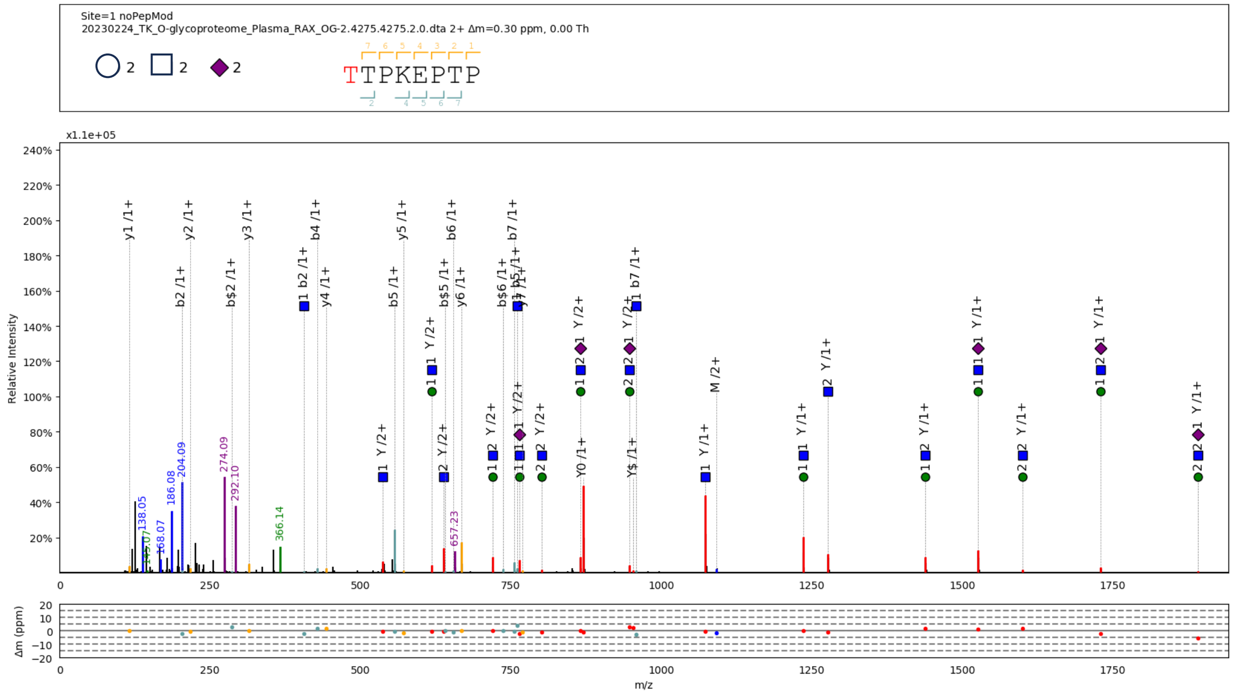

PRG4–T346-T362-T455, H (1) N (1) A (1)

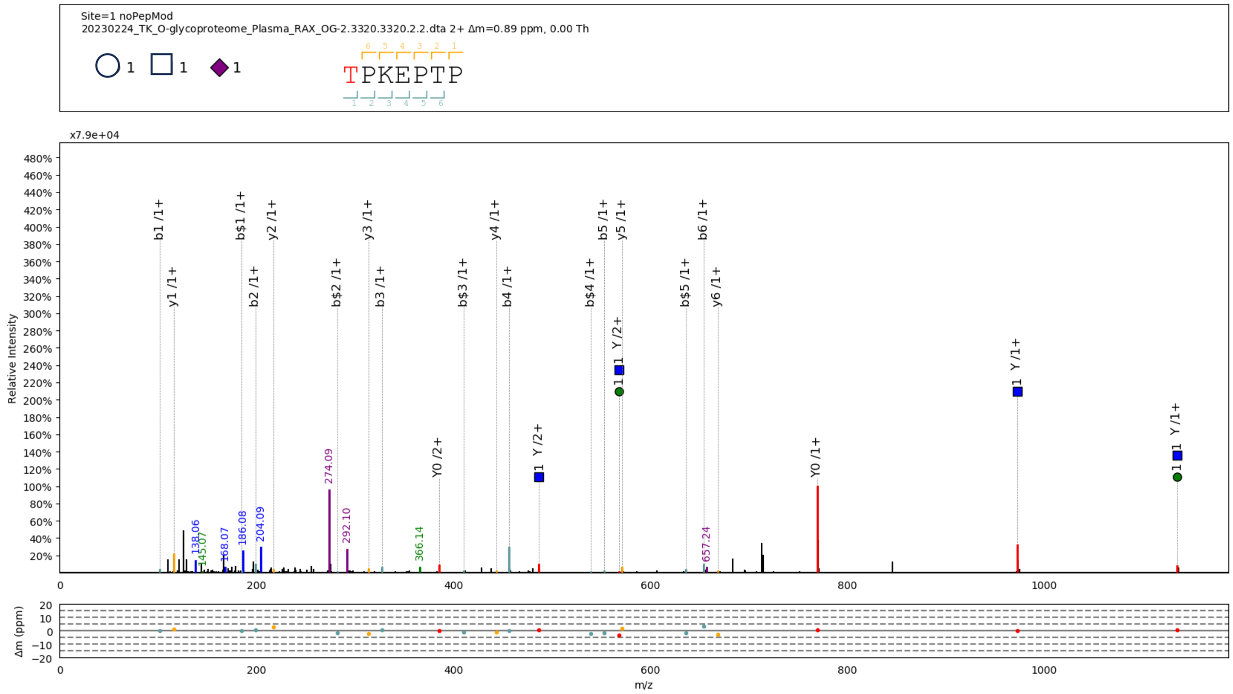

PRG4–T369, H (2) N (2) A (2)

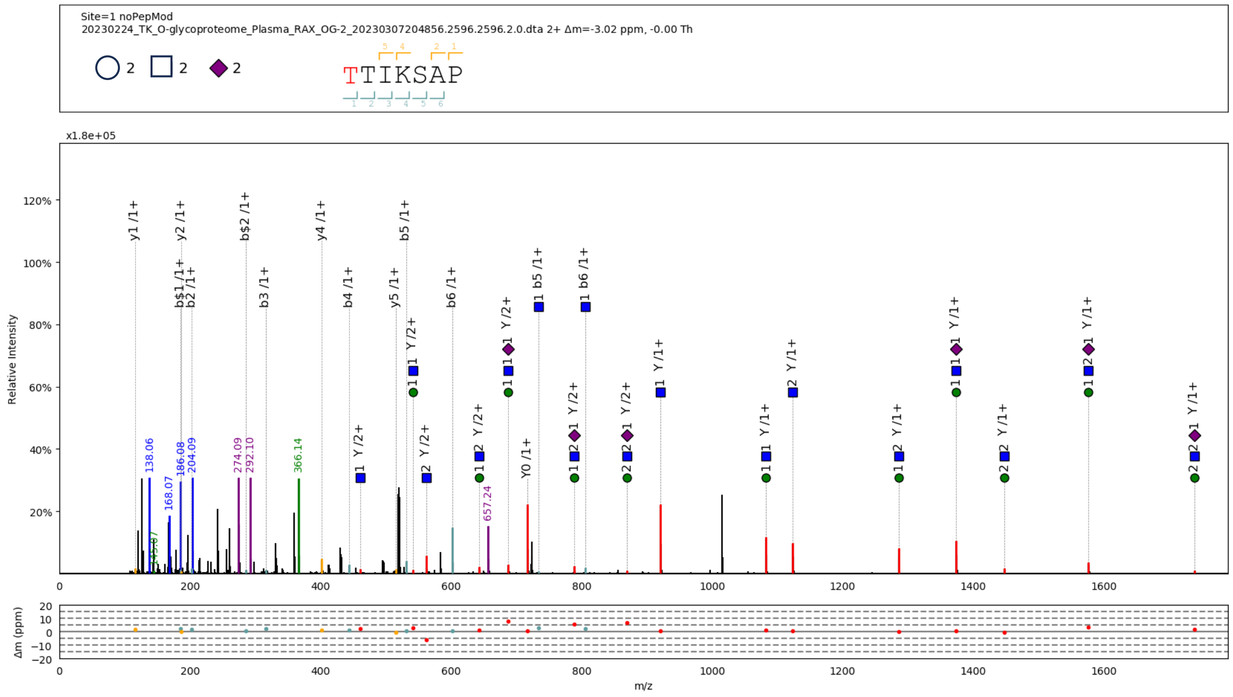

PRG4–T369, H (1) N (1) A (1)

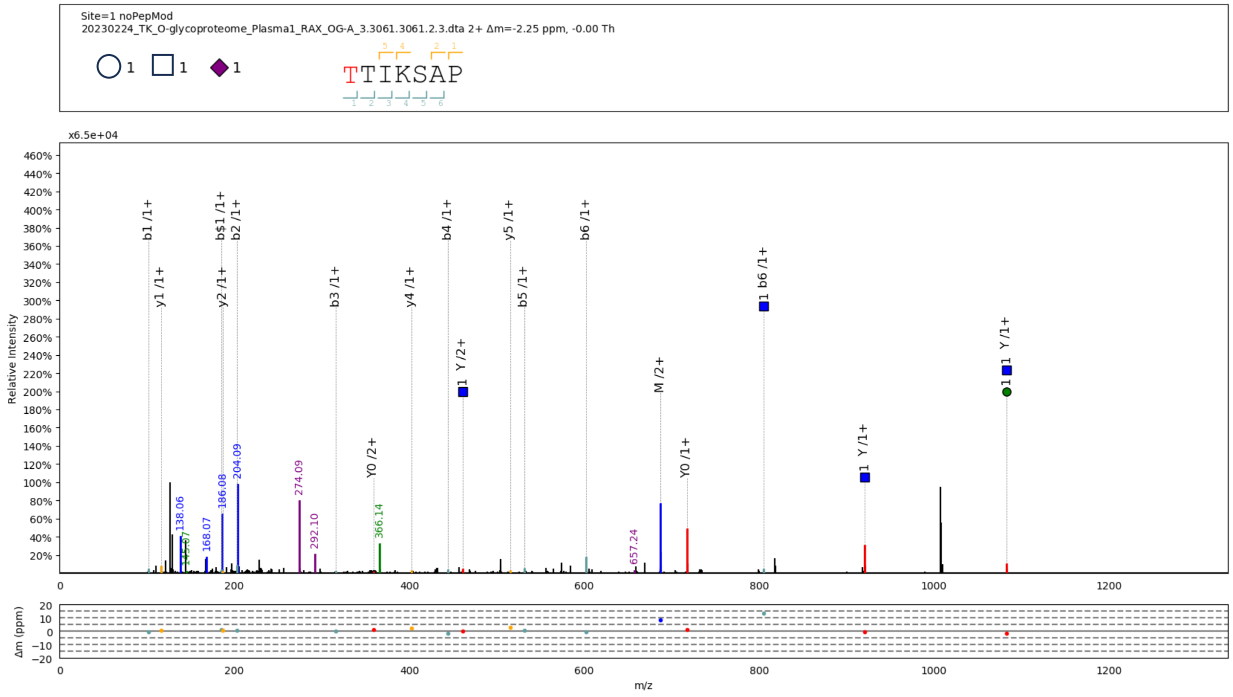

PRG4–S373, H (2) N (2) A (2)

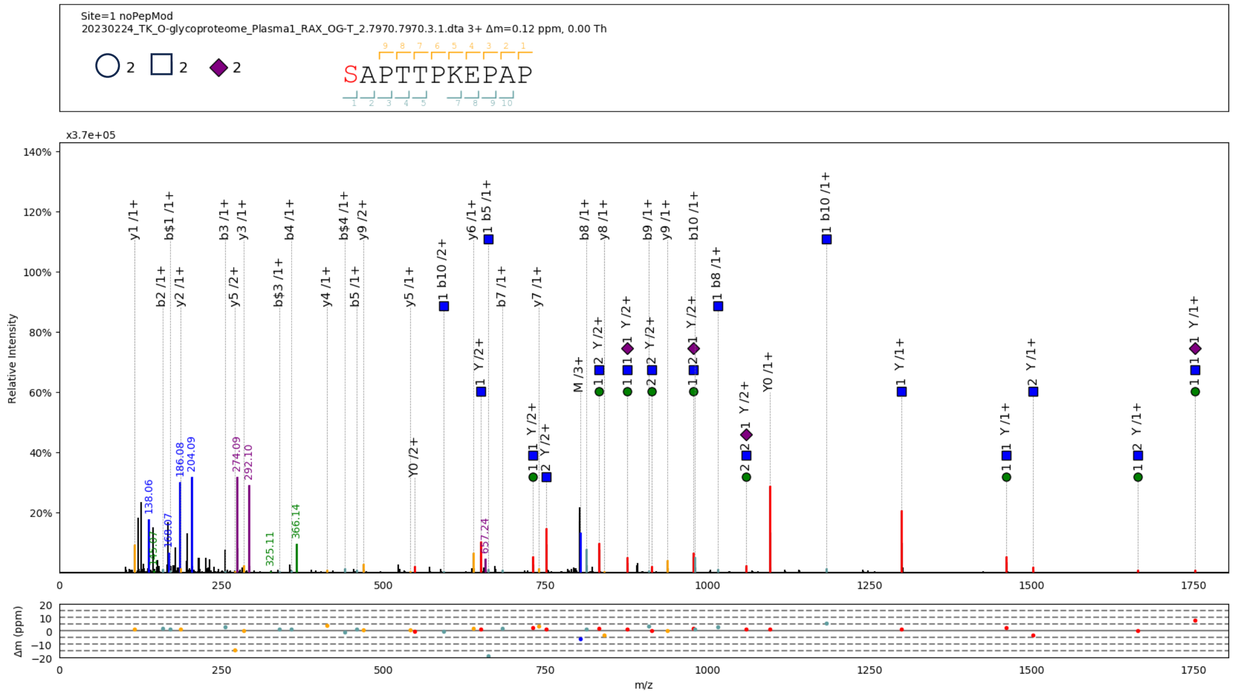

PRG4–S373, H (1) N (1) A (1)

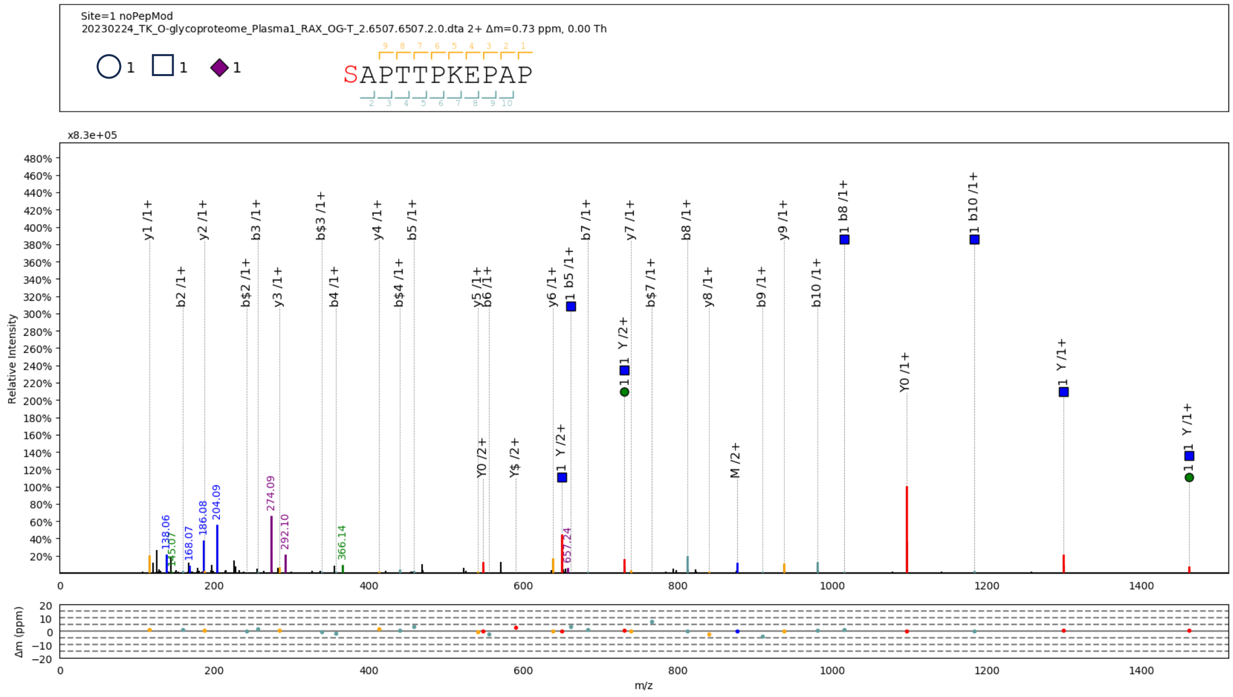

PRG4–S373, H (1) N (1)

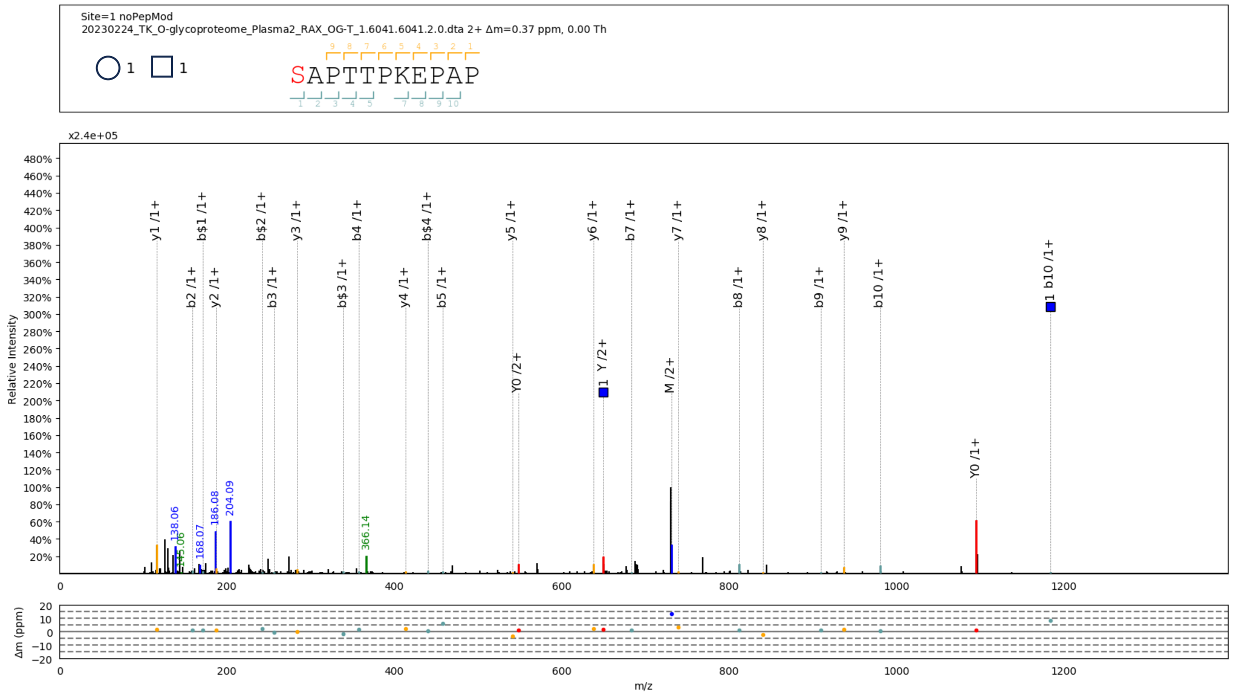

PRG4–T376-T391-T430-T446-T462-T476-T493-T501-T517-T563-T579-T587-T603-T683-T691-T760-T768-T829, H (2) N (2) A (2)

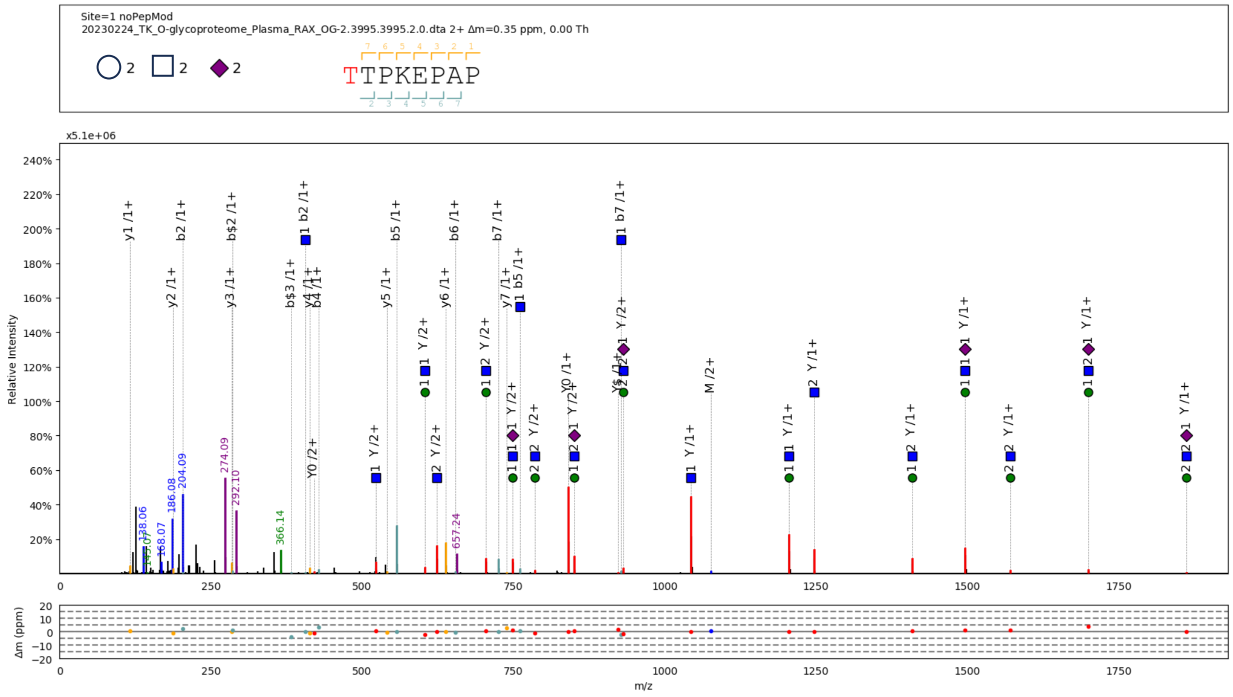

PRG4–T376-T391-T430-T446-T462-T476-T493-T501-T517-T563-T579-T587-T603-T683-T691-T760-T768-T829, H (2) N (2) A (1)

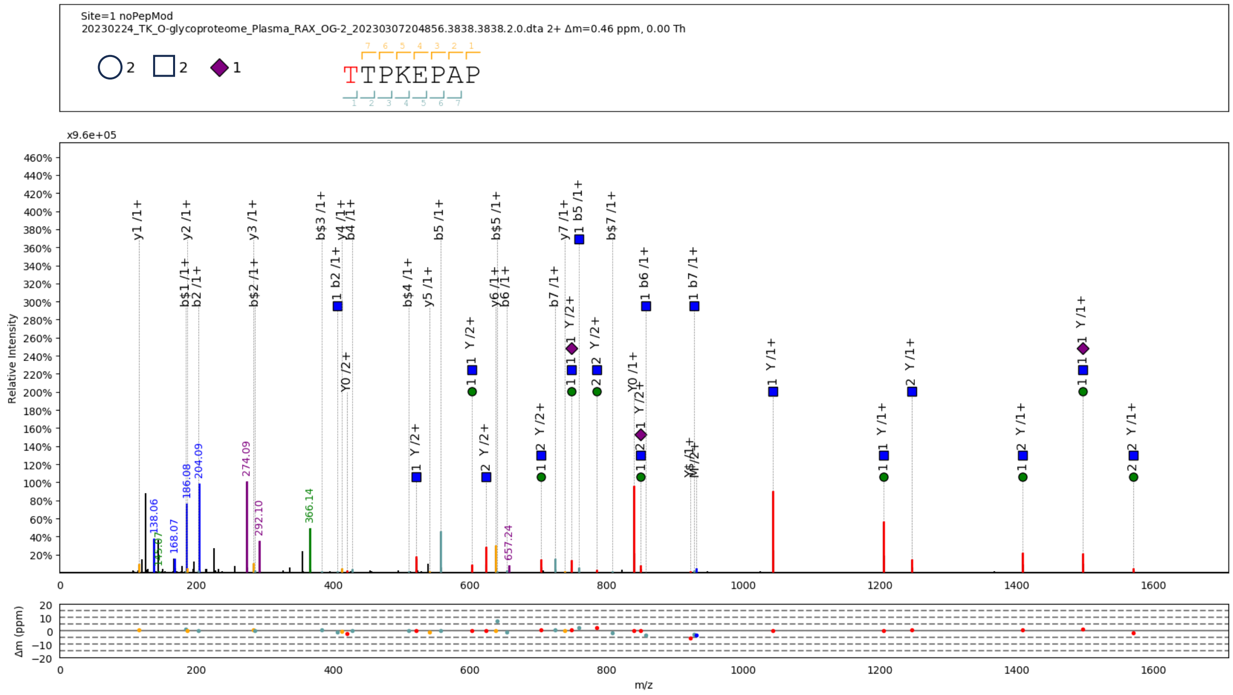

PRG4–T376-T391-T430-T446-T462-T476-T493-T501-T517-T563-T579-T587-T603-T683-T691-T760-T768-T829, H (1) N (2) A (1)

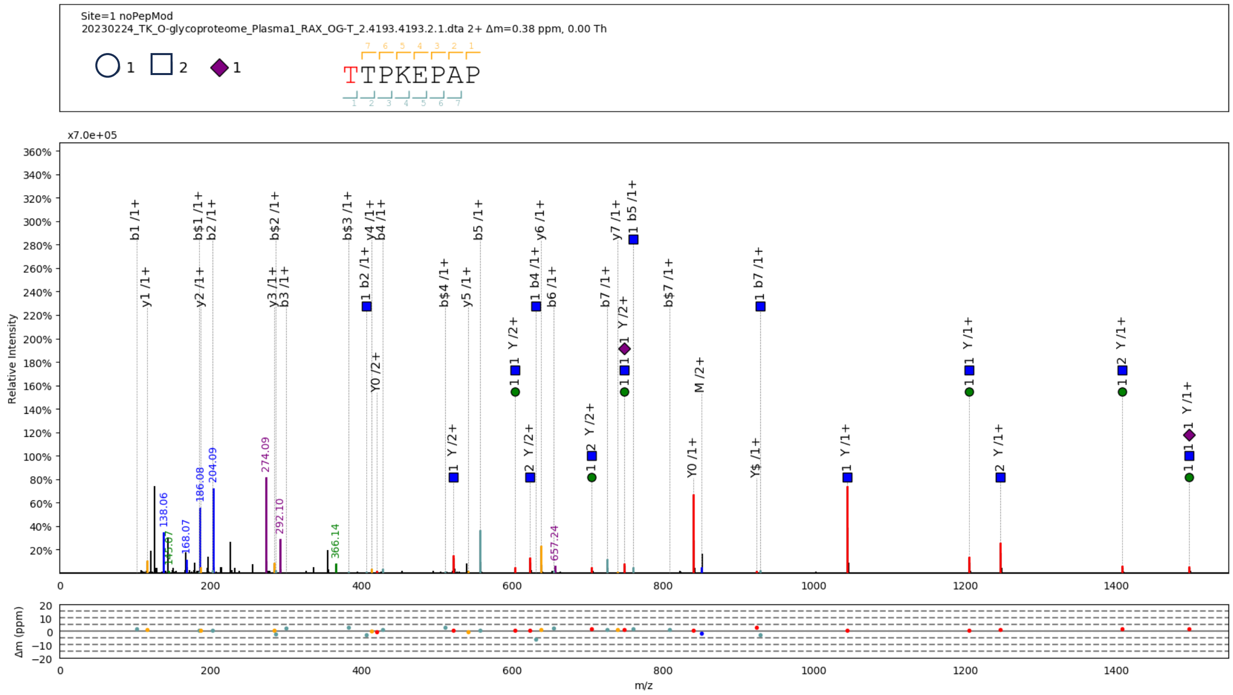

PRG4–T376-T391-T430-T446-T462-T476-T493-T501-T517-T563-T579-T587-T603-T683-T691-T760-T768-T829, H (2) N (2) A (3)

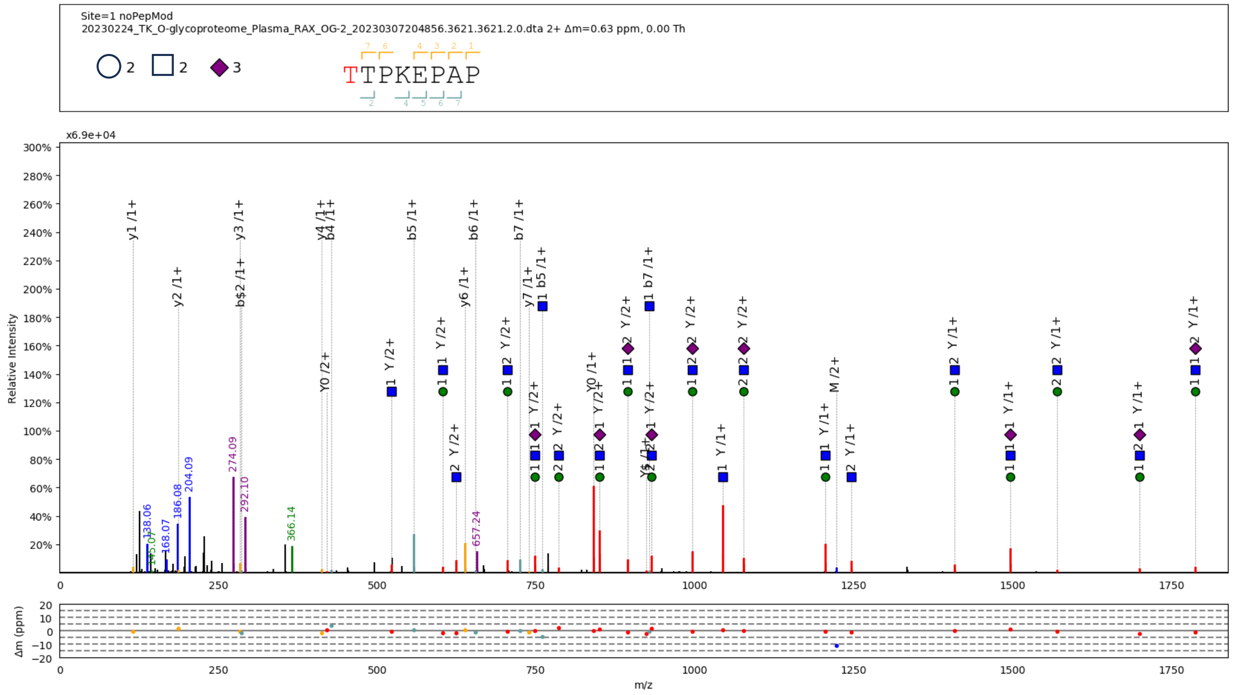

PRG4–T376-T391-T430-T446-T462-T476-T493-T501-T517-T563-T579-T587-T603-T683-T691-T760-T768-T829, H (3) N (2) A (1) F (1)

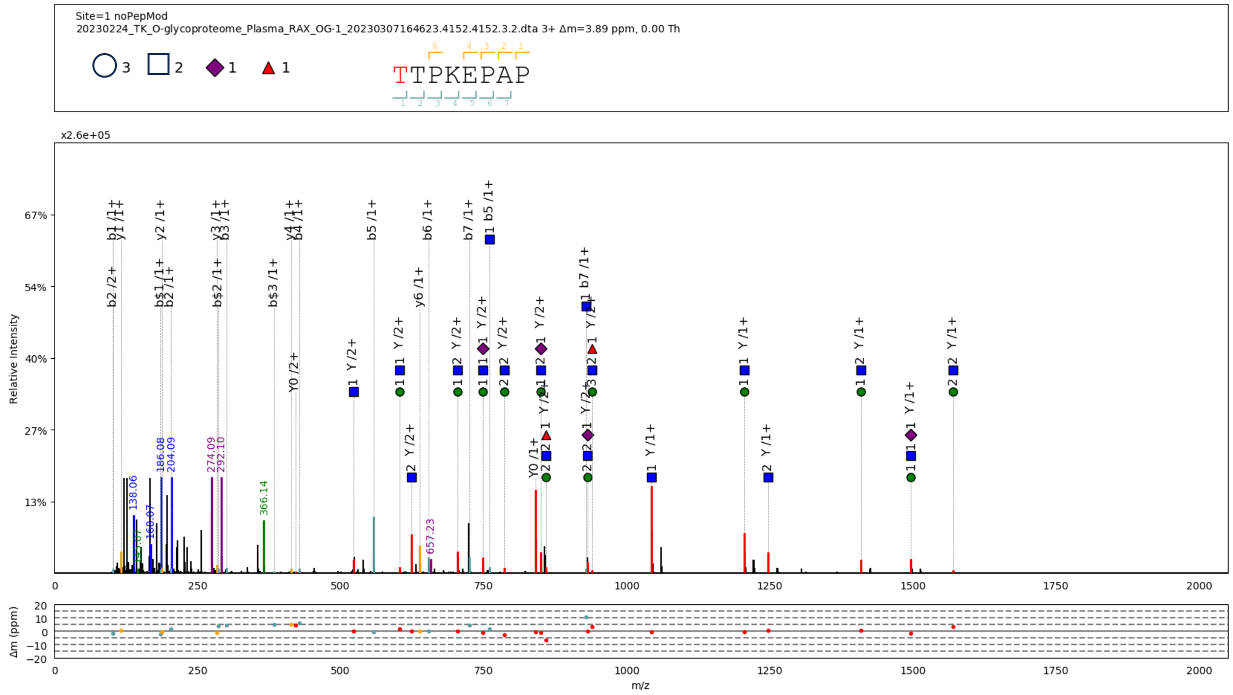

PRG4–T376-T391-T430-T446-T462-T476-T493-T501-T517-T563-T579-T587-T603-T683-T691-T760-T768-T829, H (1) N (1) A (1)

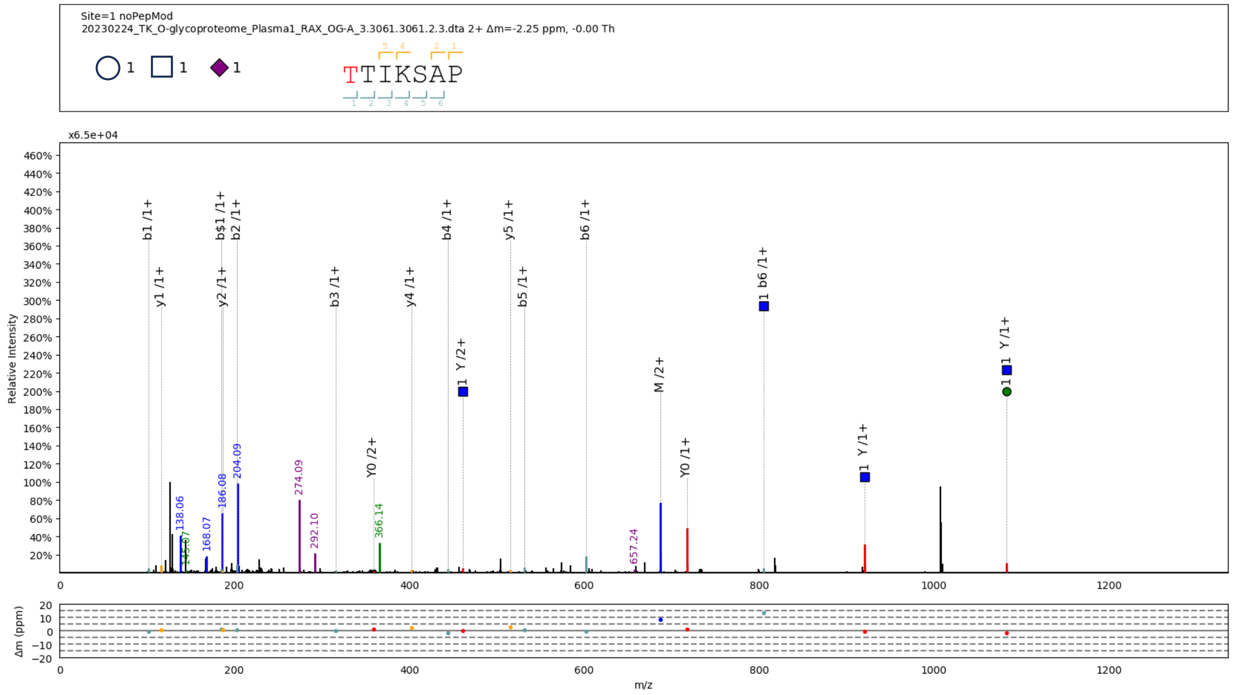

PRG4–T376-T391-T430-T446-T462-T476-T493-T501-T517-T563-T579-T587-T603-T683-T691-T760-T768-T829, H (1) N (2)

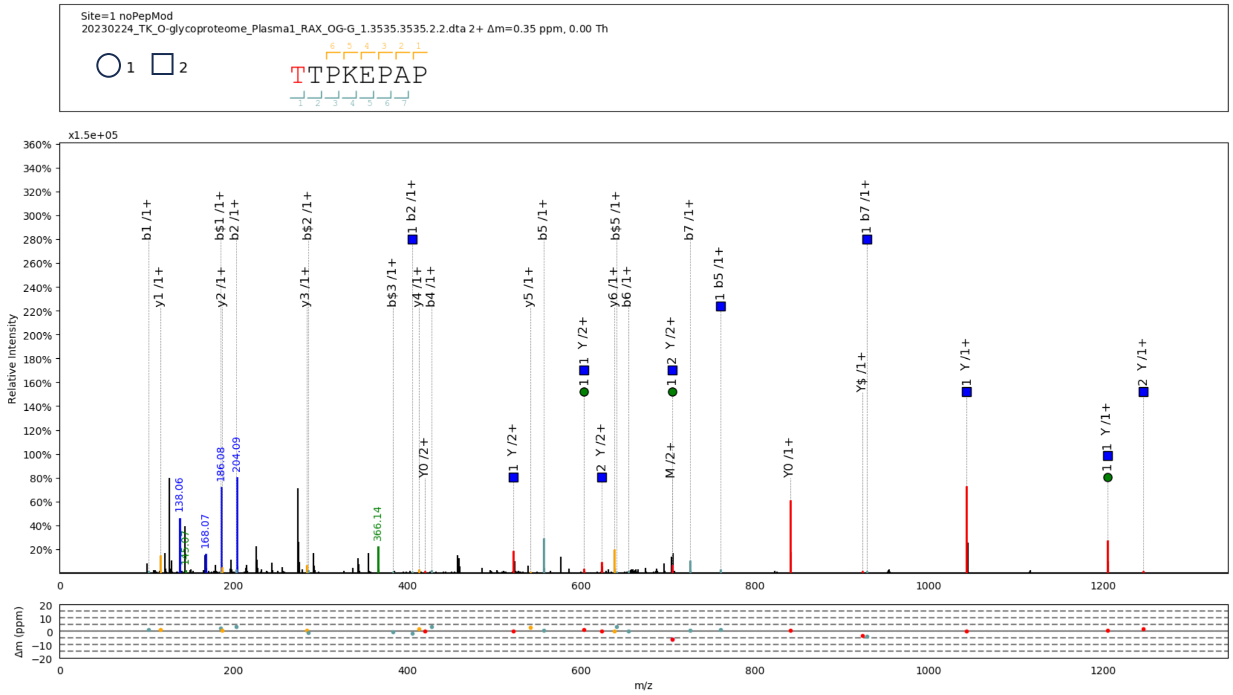

PRG4‒T376-T391-T430-T446-T462-T476-T493-T501-T517-T563-T579-T587-T603-T683-T691-T760-T768-T829, H (2) N (2)

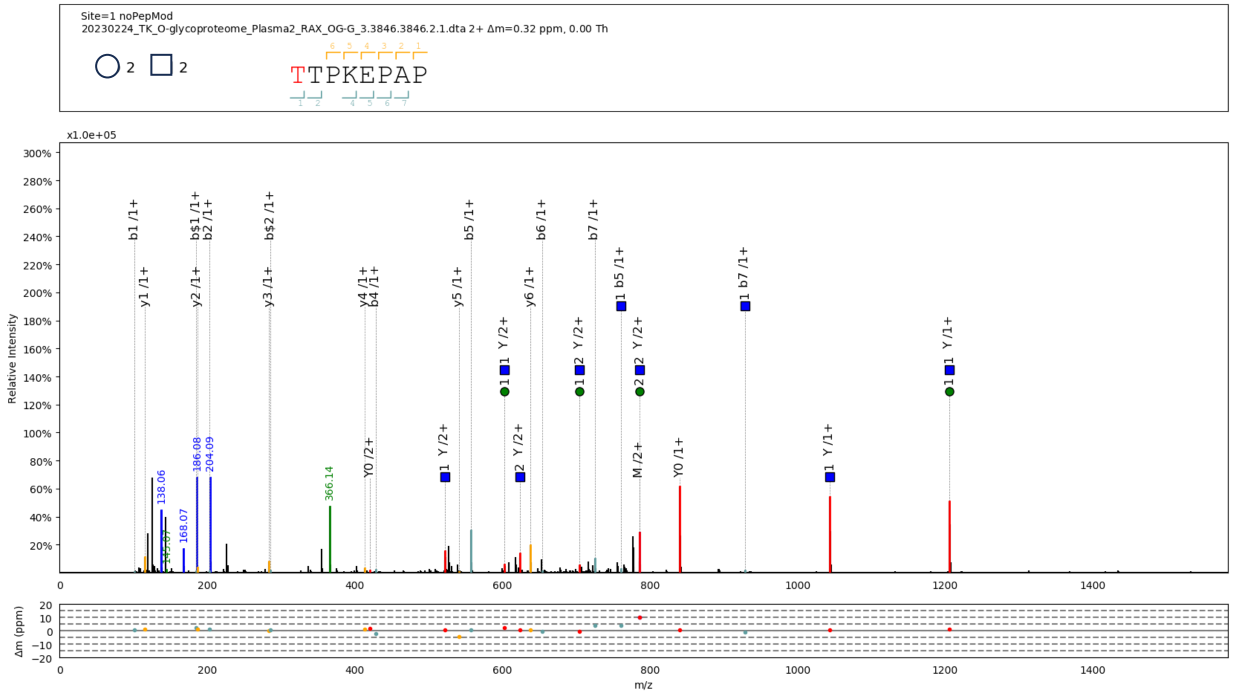

PRG4‒T376-T391-T430-T446-T462-T476-T493-T501-T517-T563-T579-T587-T603-T683-T691-T760-T768-T829, H (1) N (2) A (1) F (1)

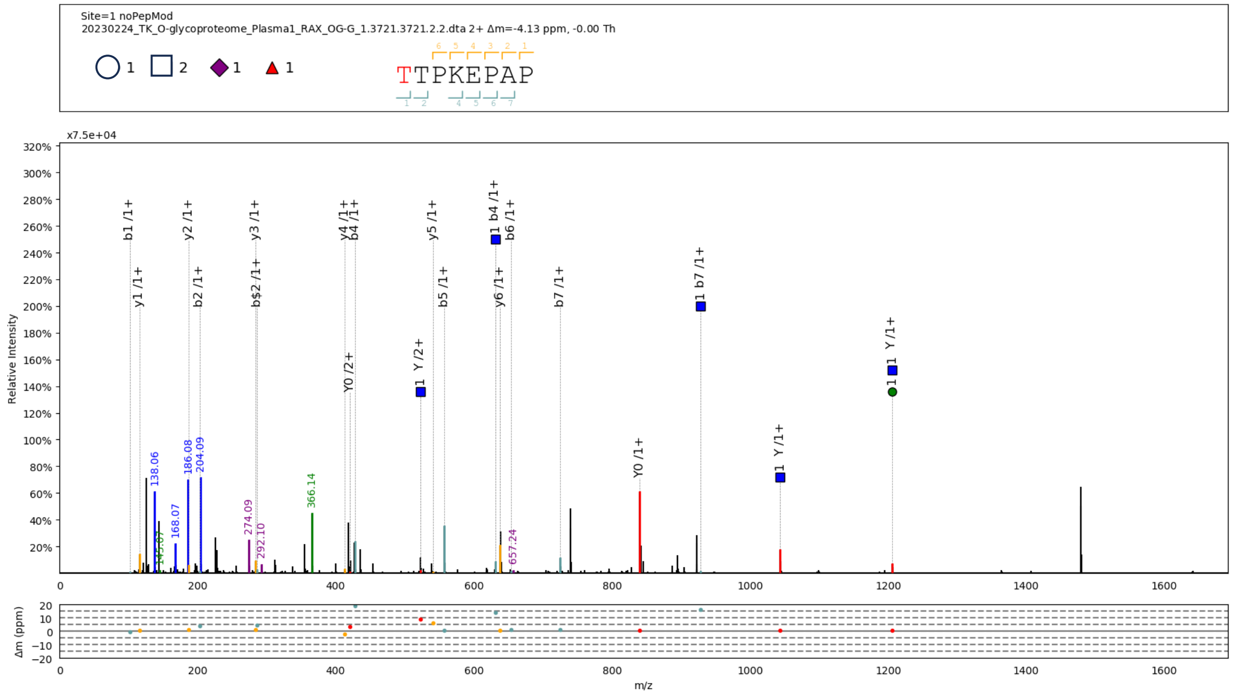

PRG4–T376-T391-T430-T446-T462-T476-T493-T501-T517-T563-T579-T587-T603-T683-T691-T760-T768-T829, H (1) N (2) A (1) F (1)

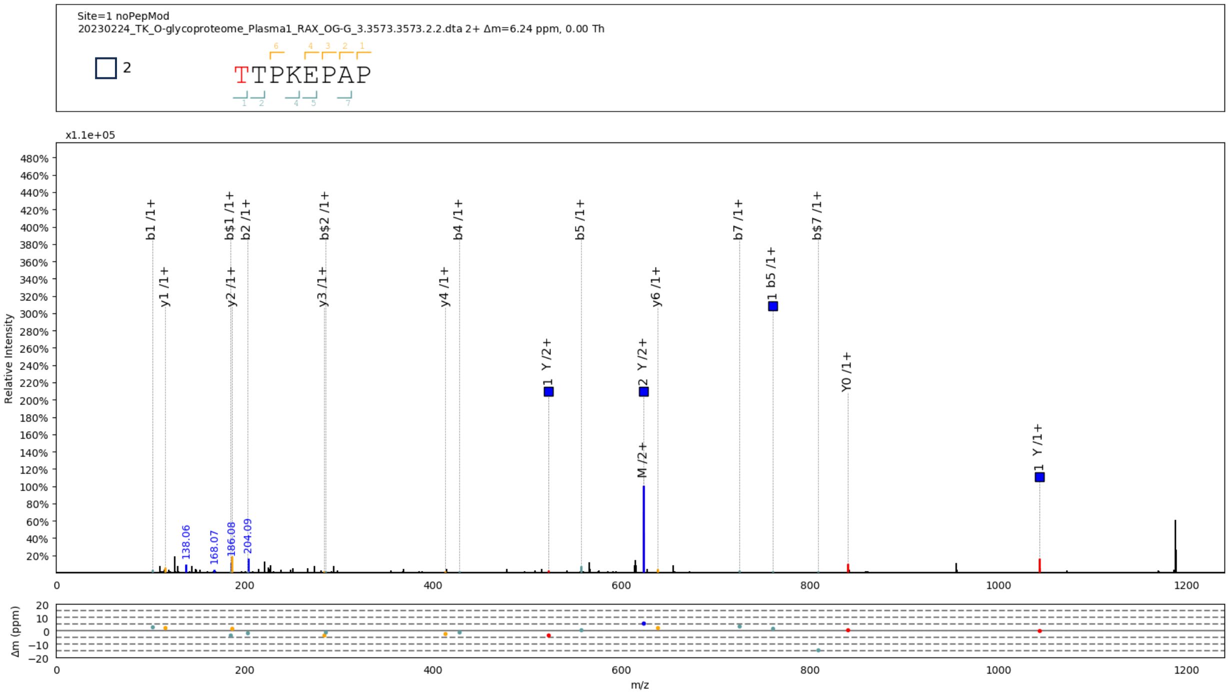

PRG4–T399-T415-T532-T555, H (3) N (3) A (3)

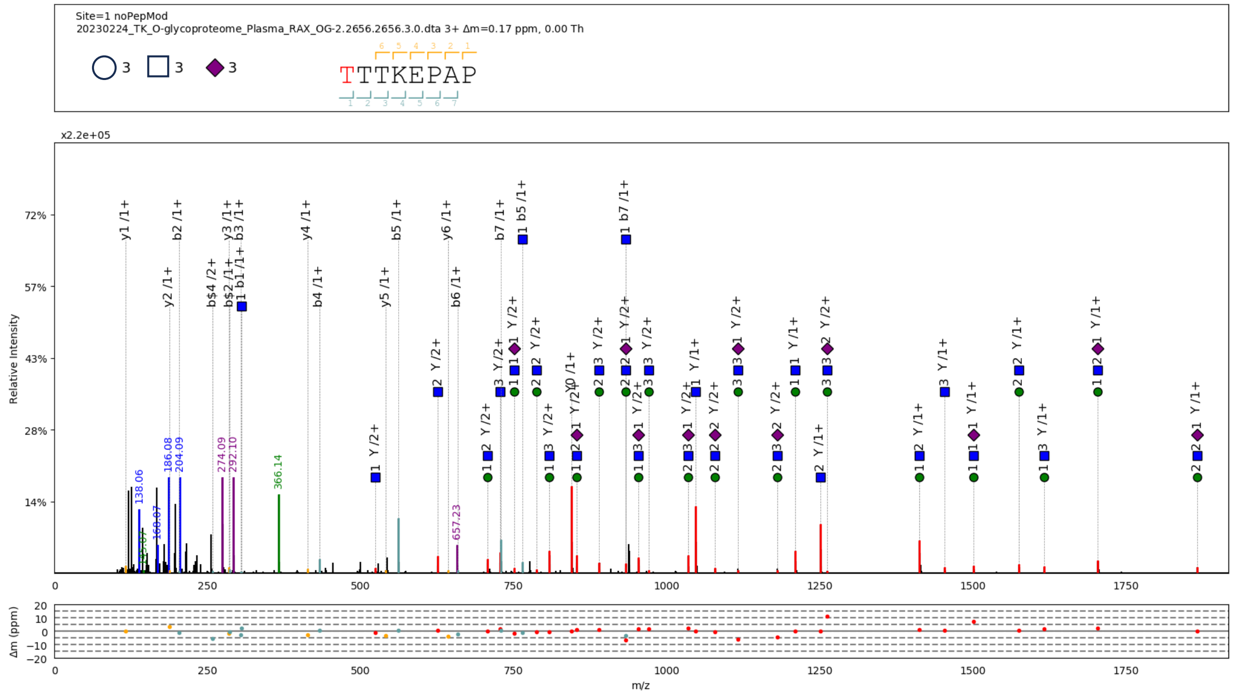

PRG4–T399-T415-T532-T555, H (2) N (2) A (2)

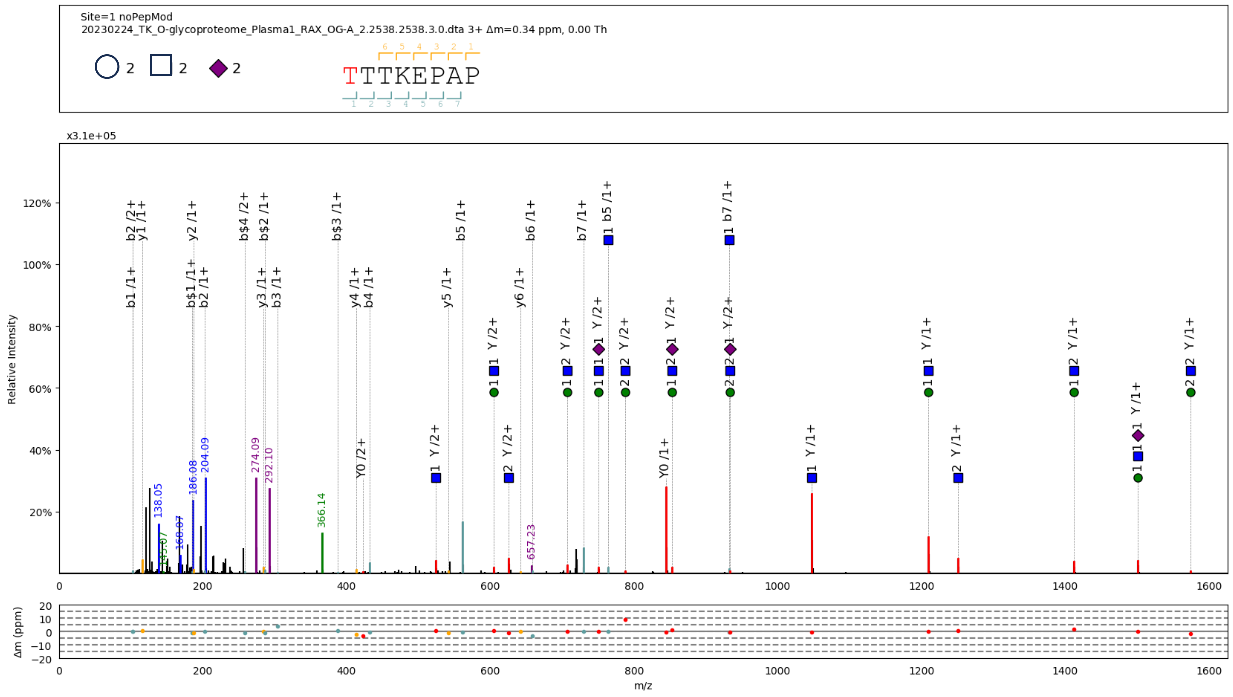

PRG4–T399-T415-T532-T555, H (3) N (3) A (2)

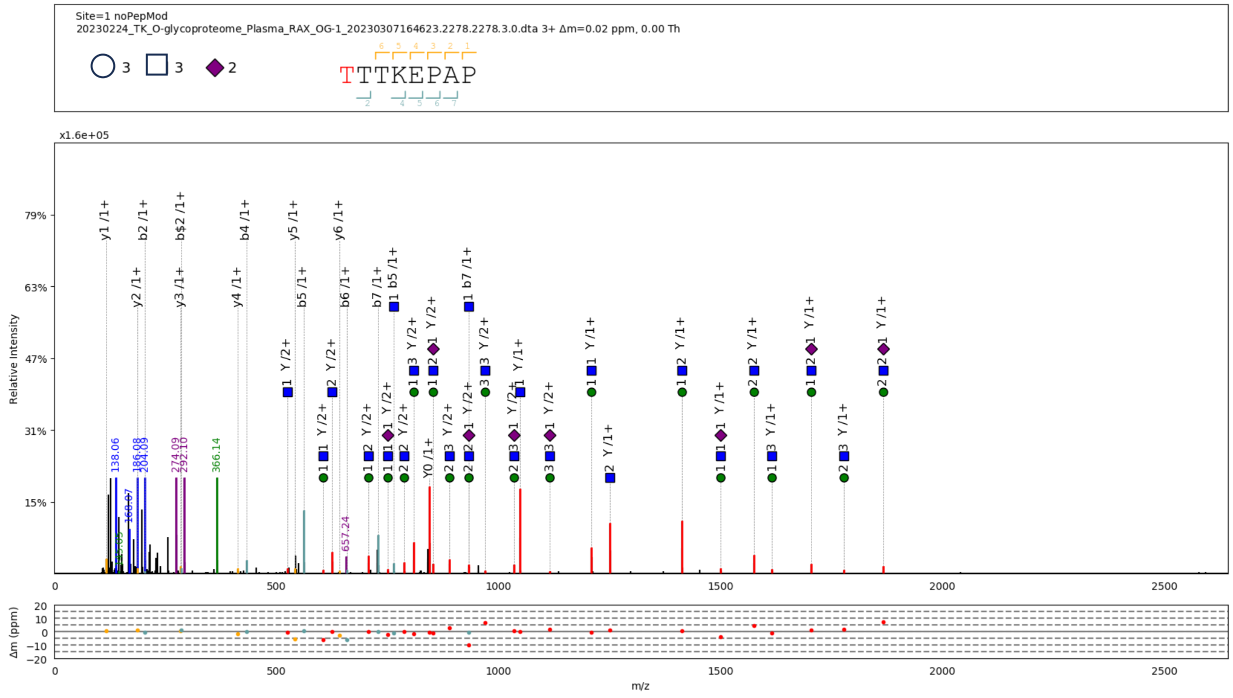

PRG4–T399-T415-T532-T555, H (2) N (3) A (2)

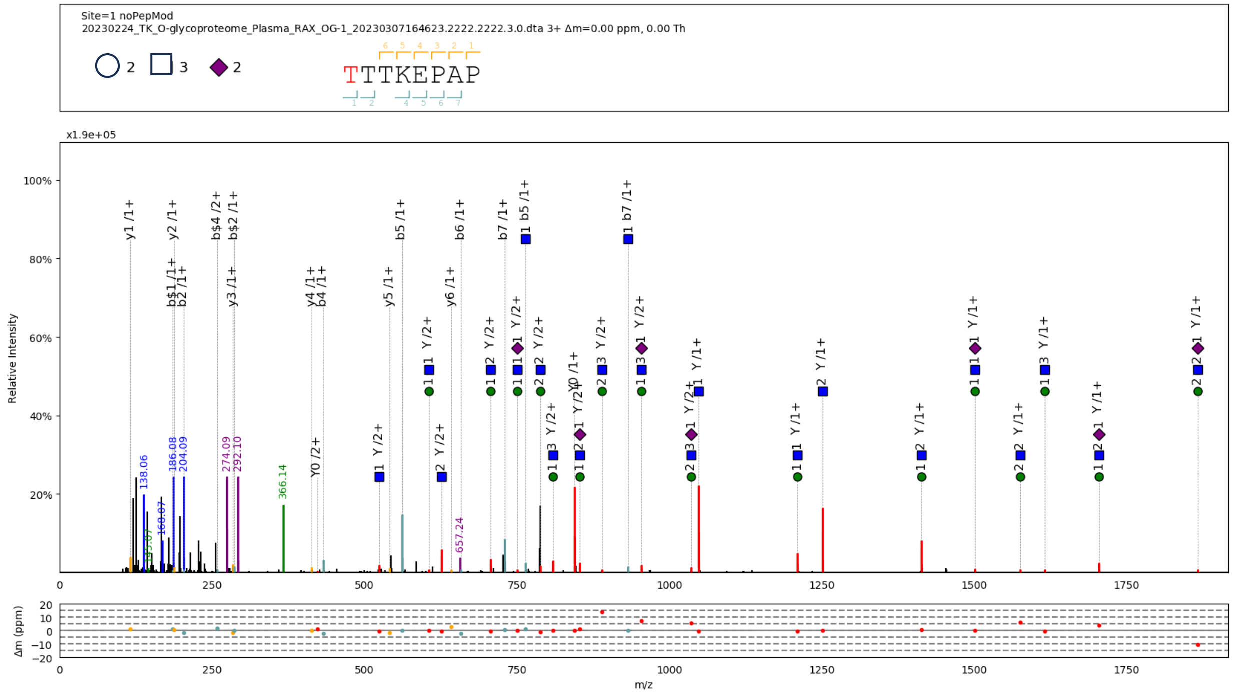

PRG4–T399-T415-T532-T555, H (2) N (2) A (1)

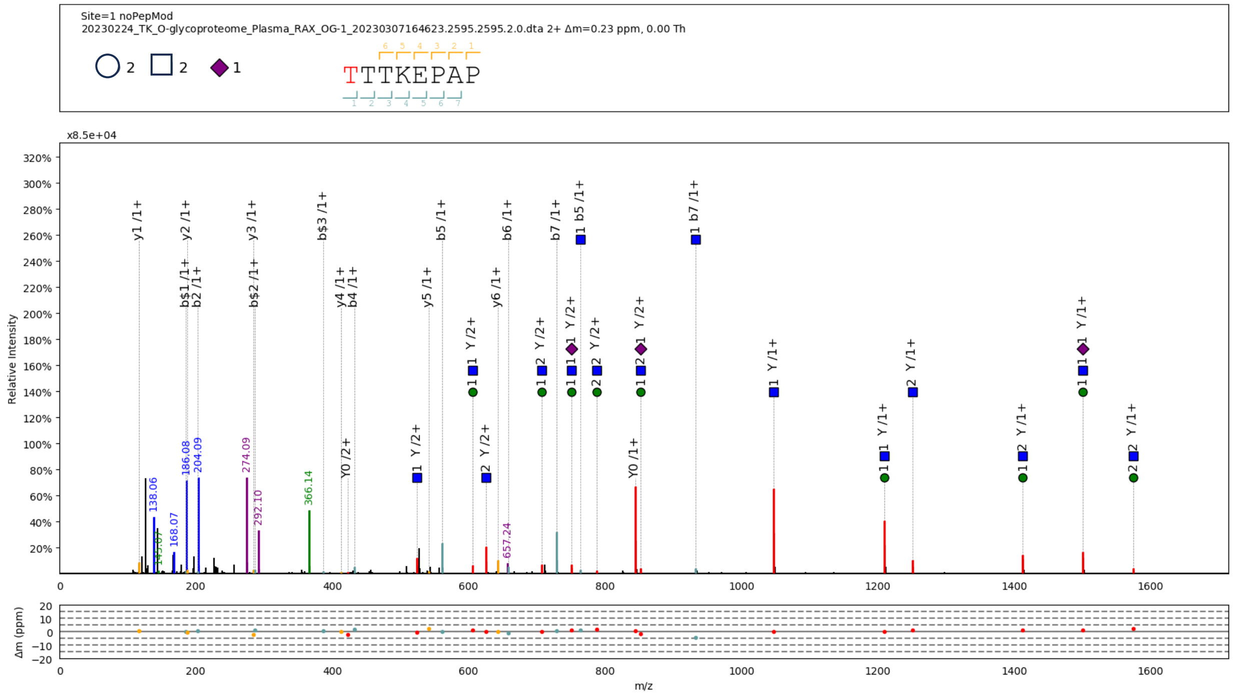

PRG4–T399-T415-T532-T555, H (2) N (2) F (2)

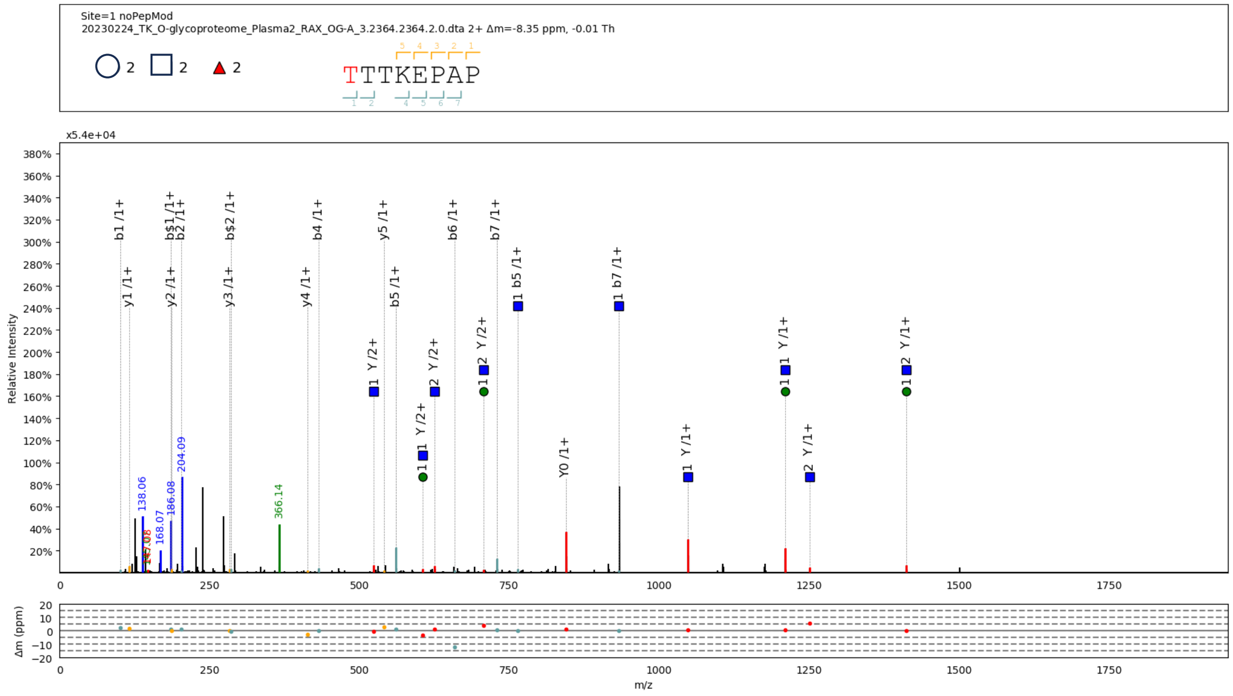

PRG4–T400-T416-T533-T556, H (2) N (2) A (2)

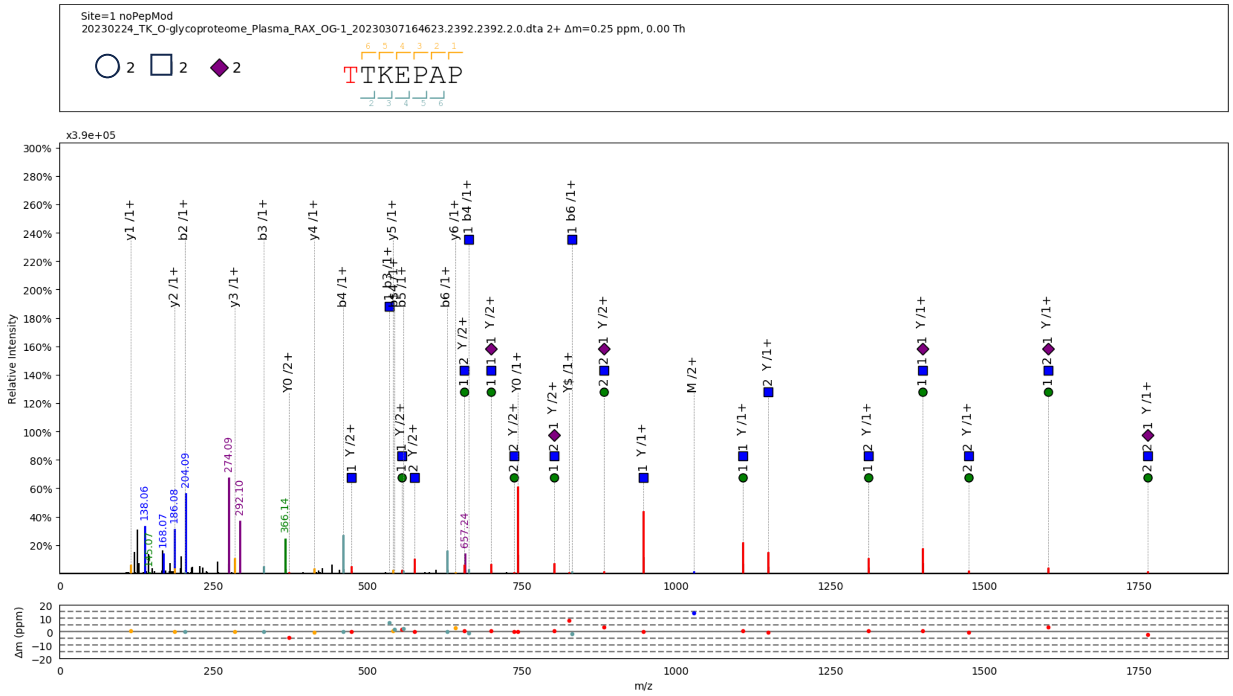

PRG4–T400-T416-T533-T556, H (2) N (2) A (1)

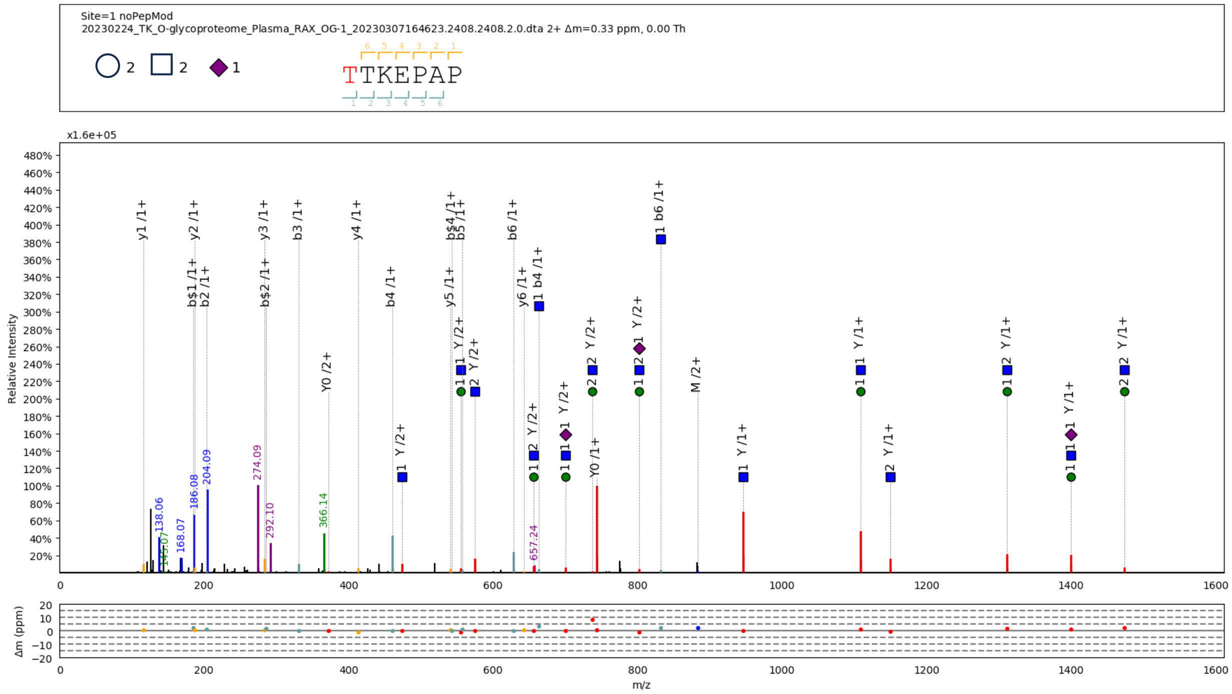

PRG4–T400-T416-T533-T556, H (1) N (2) A (1)

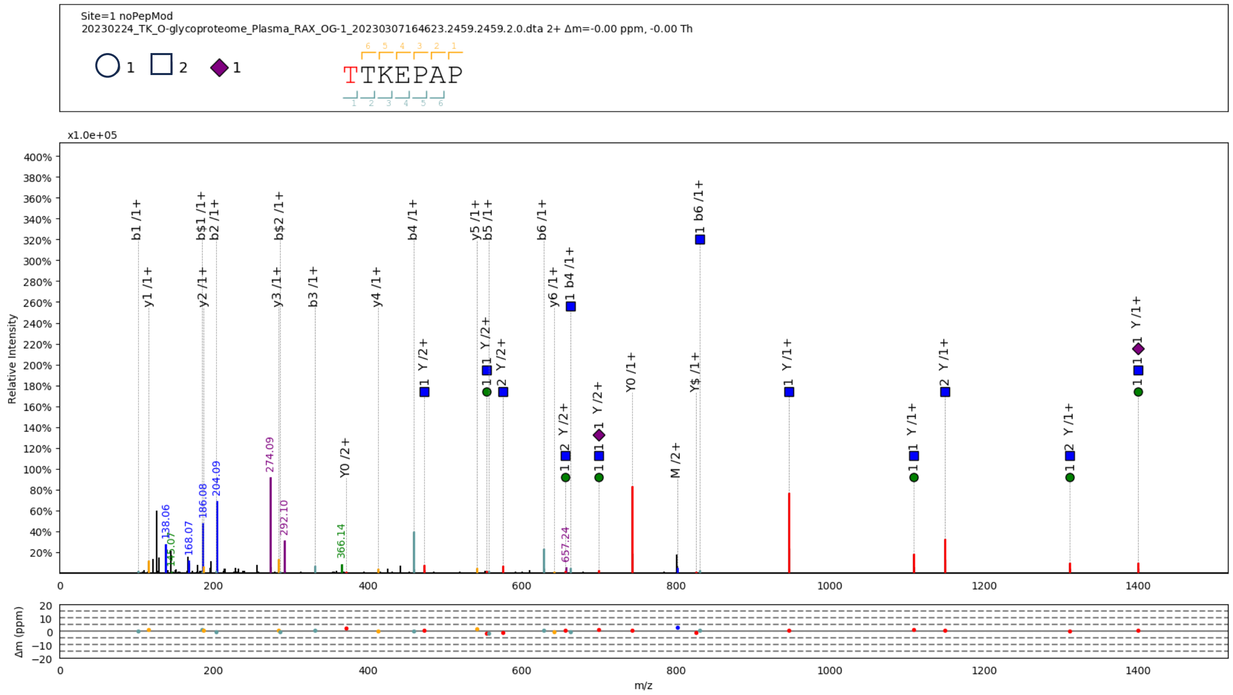

PRG4–T438-T571-T723-T792-T837, H (2) N (2) A (2)

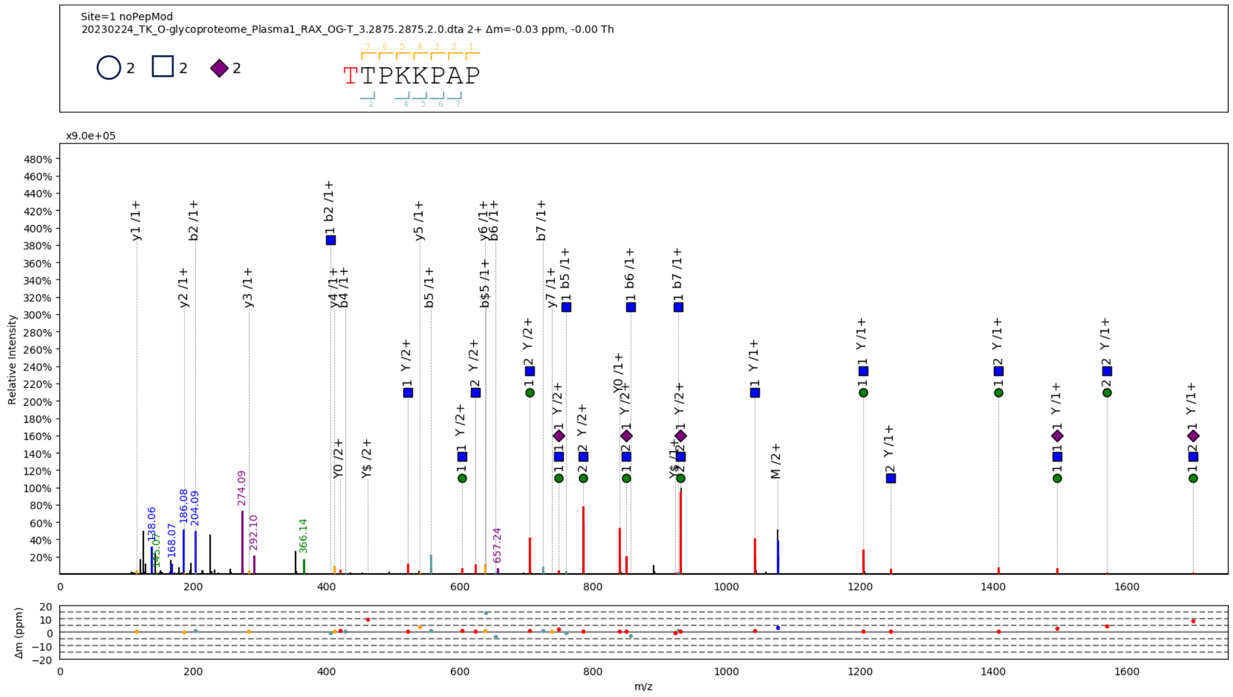

PRG4–T438-T571-T723-T792-T837, H (1) N (2) A (1)

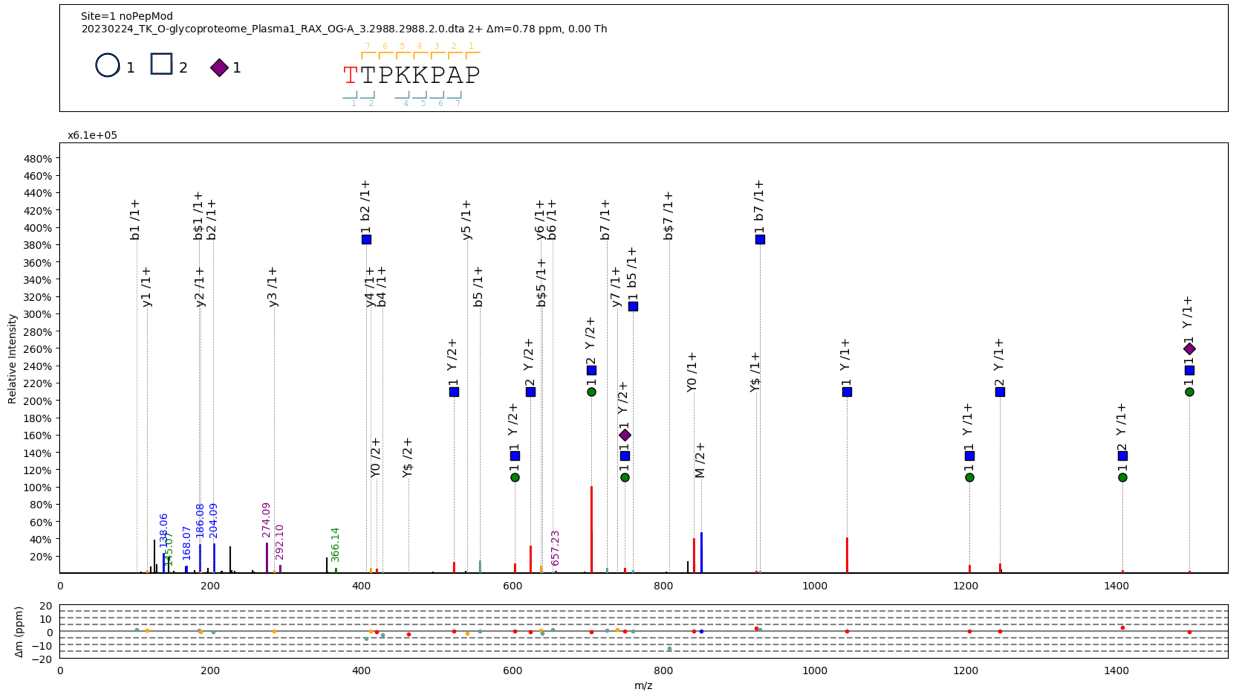

PRG4–T438-T571-T723-T792-T837, H (2) N (2) A (1)

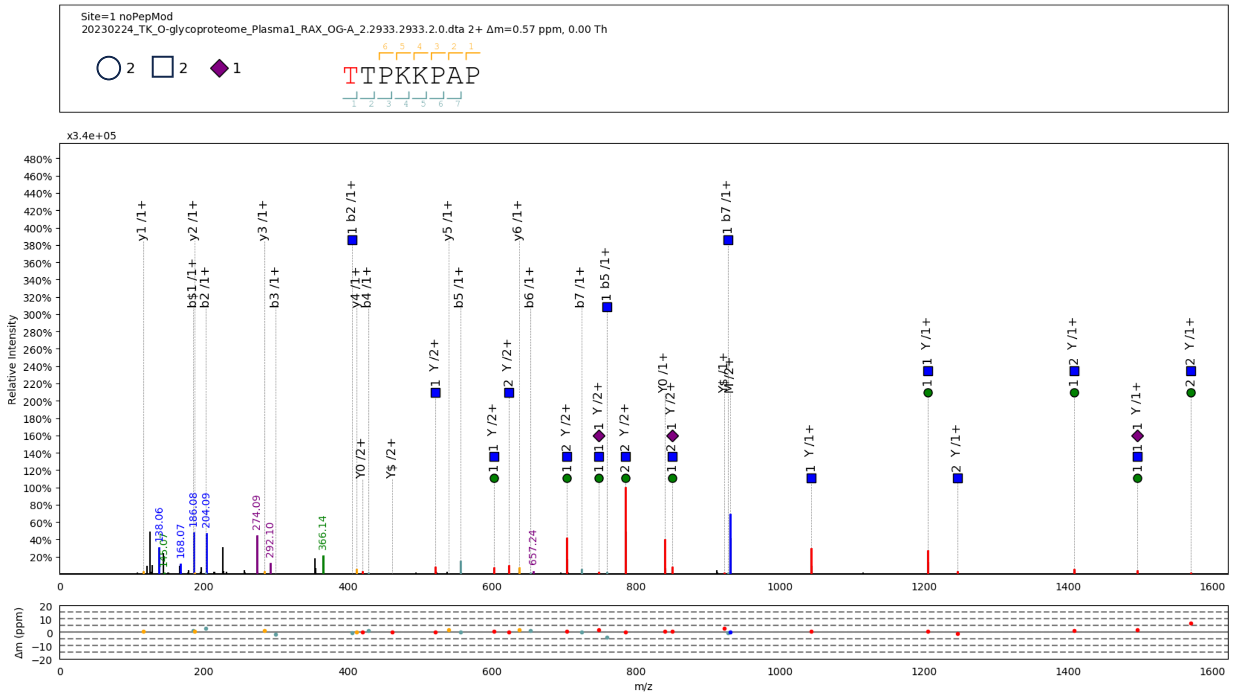

PRG4–T438-T571-T723-T792-T837, H (2) N (2)

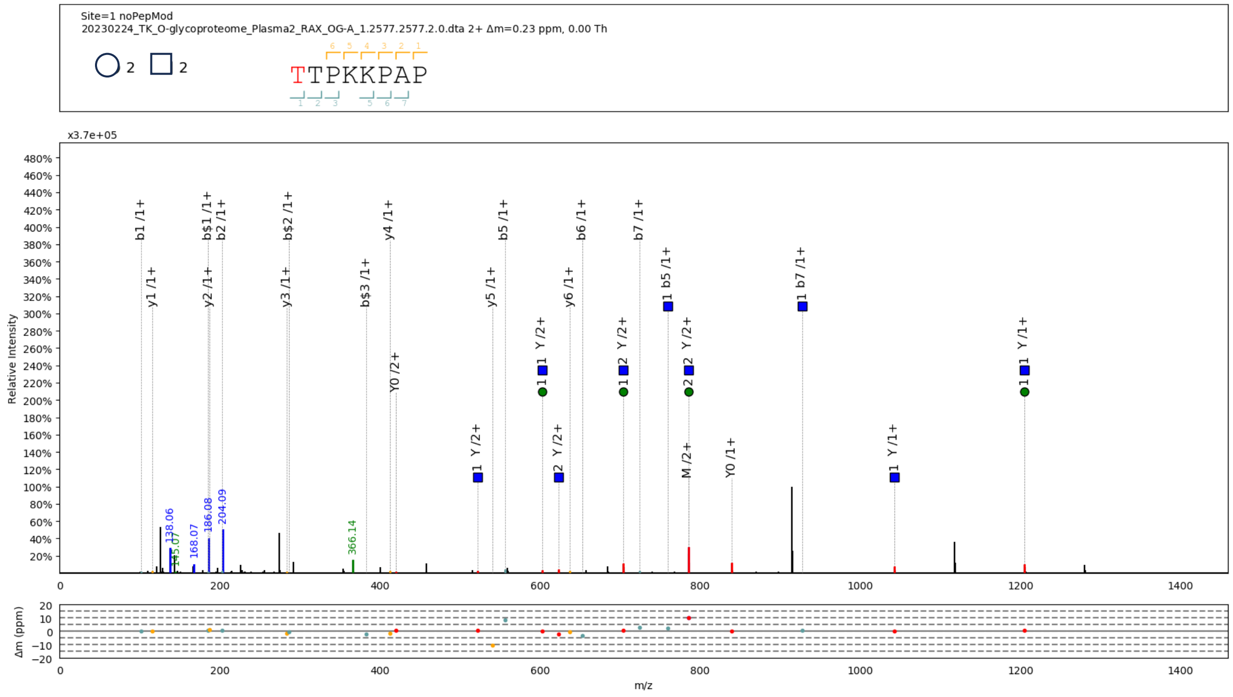

PRG4–T438-T571-T723-T792-T837, H (1) N (2)

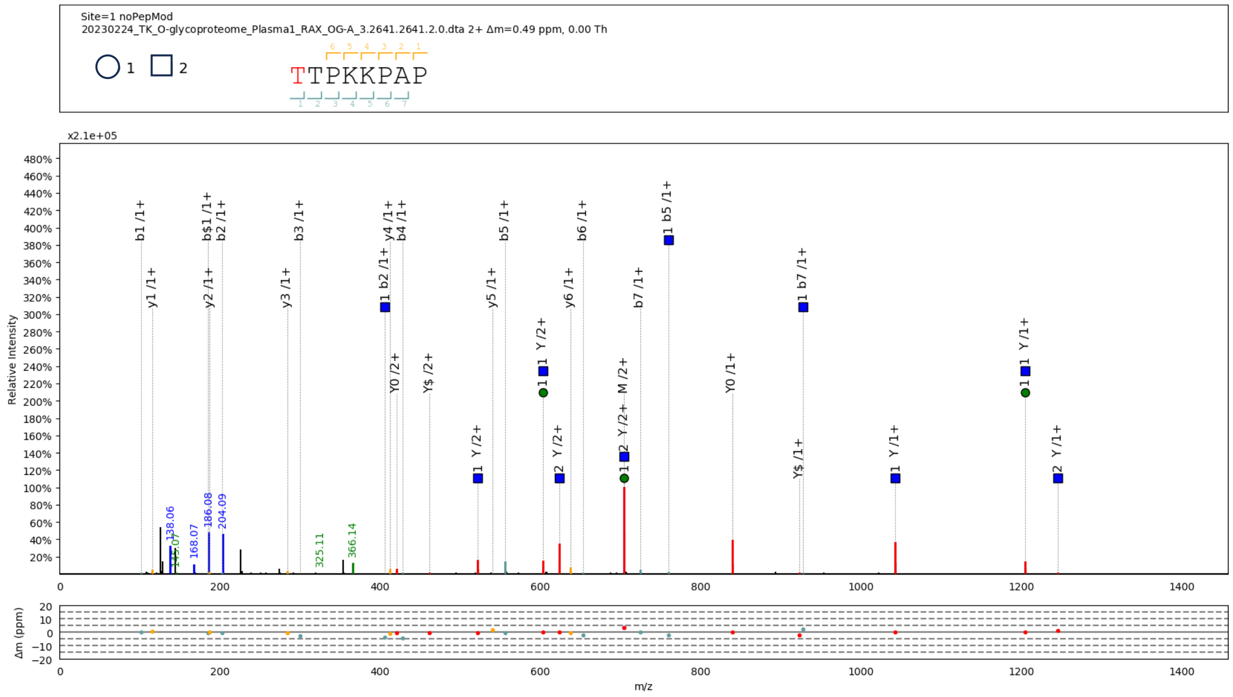

PRG4–T438-T571-T723-T792-T837, H (1) N (1)

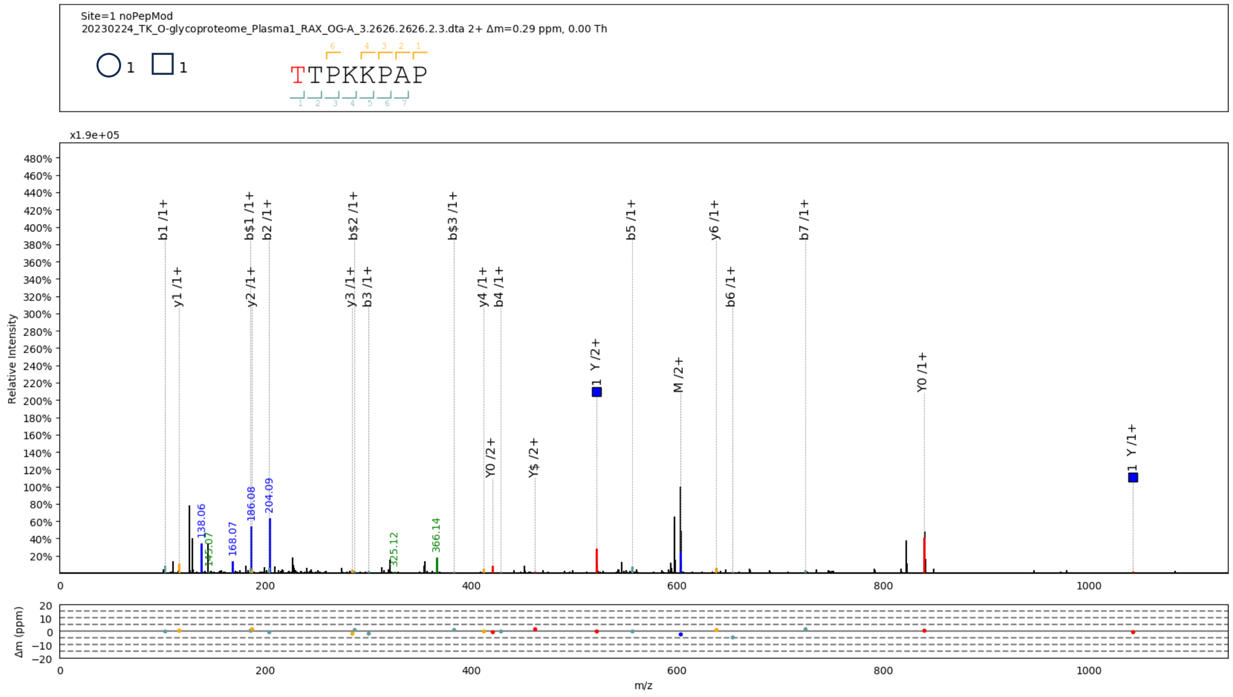

PRG4−T460, H (2) N (3) A (2)

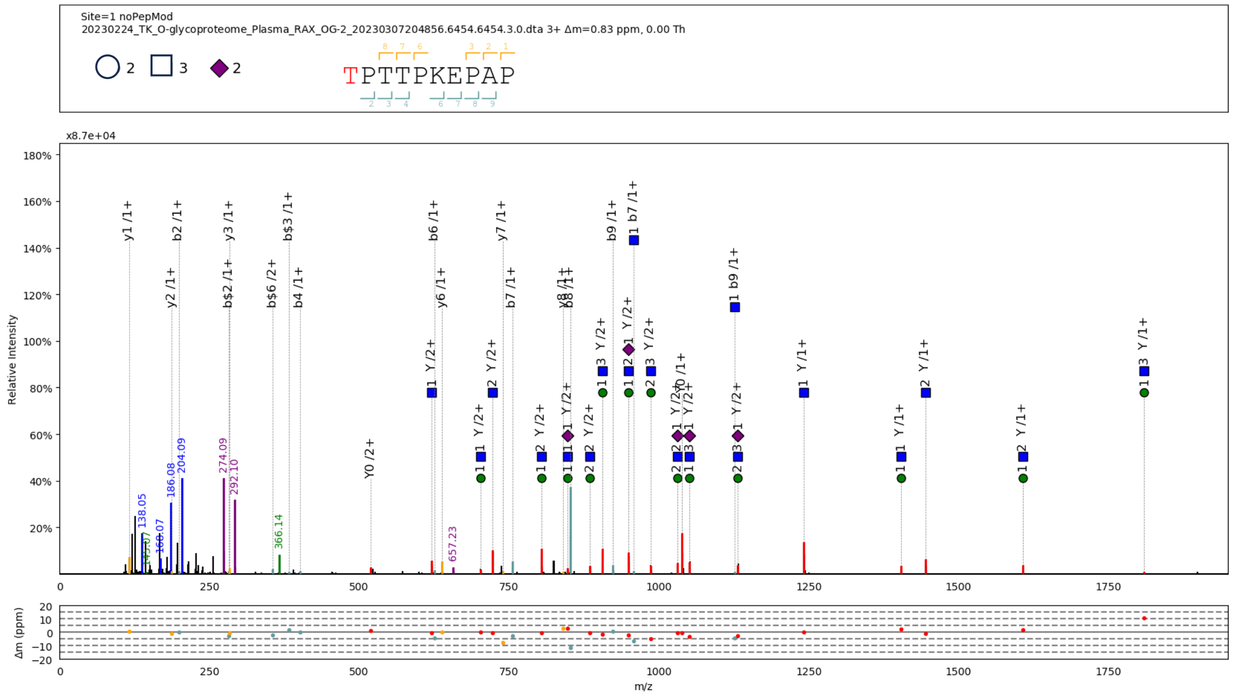

PRG4−T460, H (2) N (2) A (2)

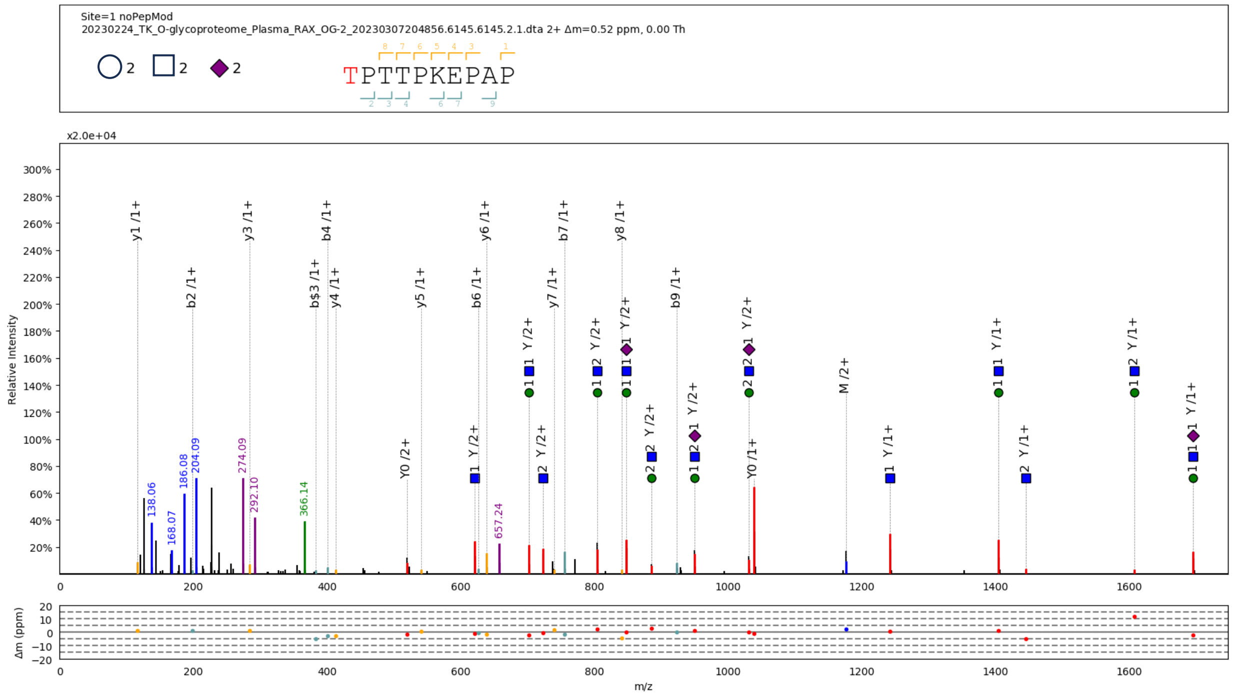

PRG4–T485, H (3) N (3) A (3)

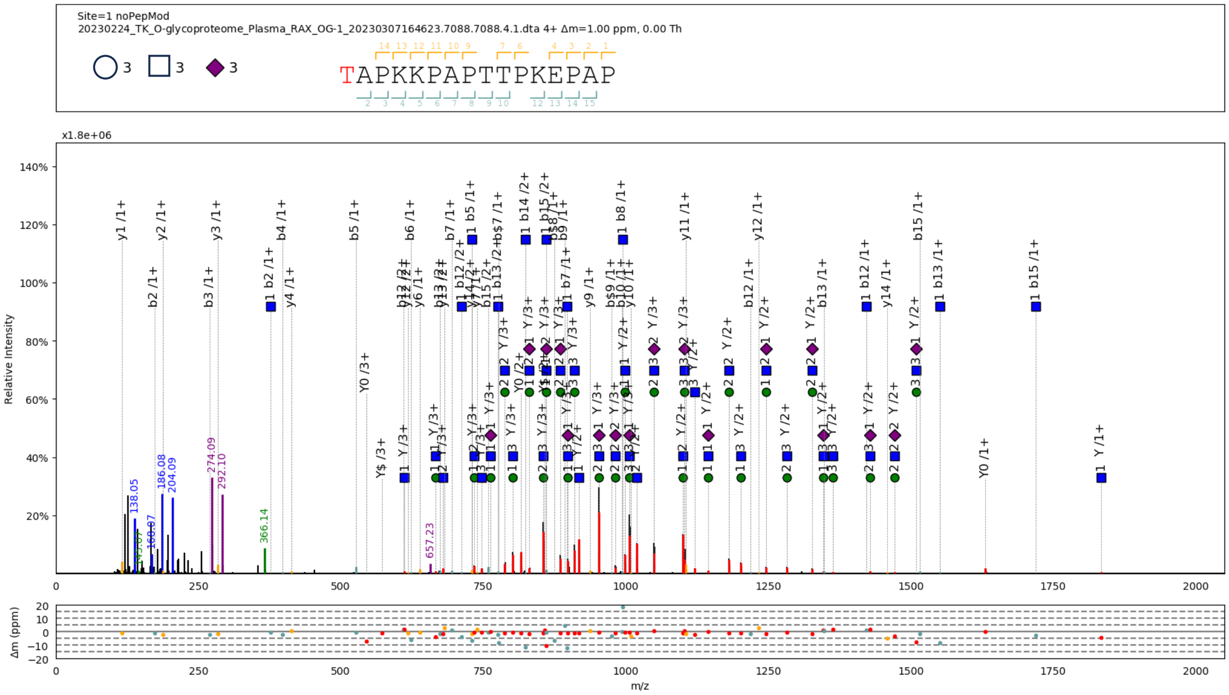

PRG4–T485, H (2) N (3) A (2)

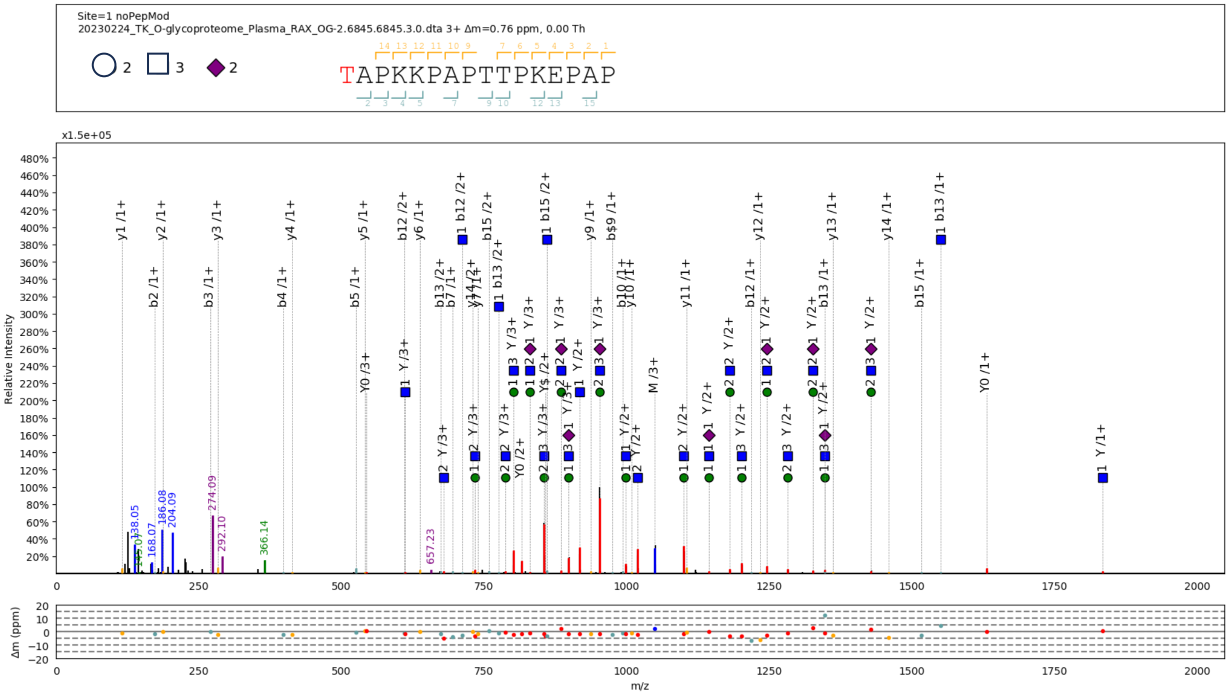

PRG4–T485, H (3) N (3) A (2)

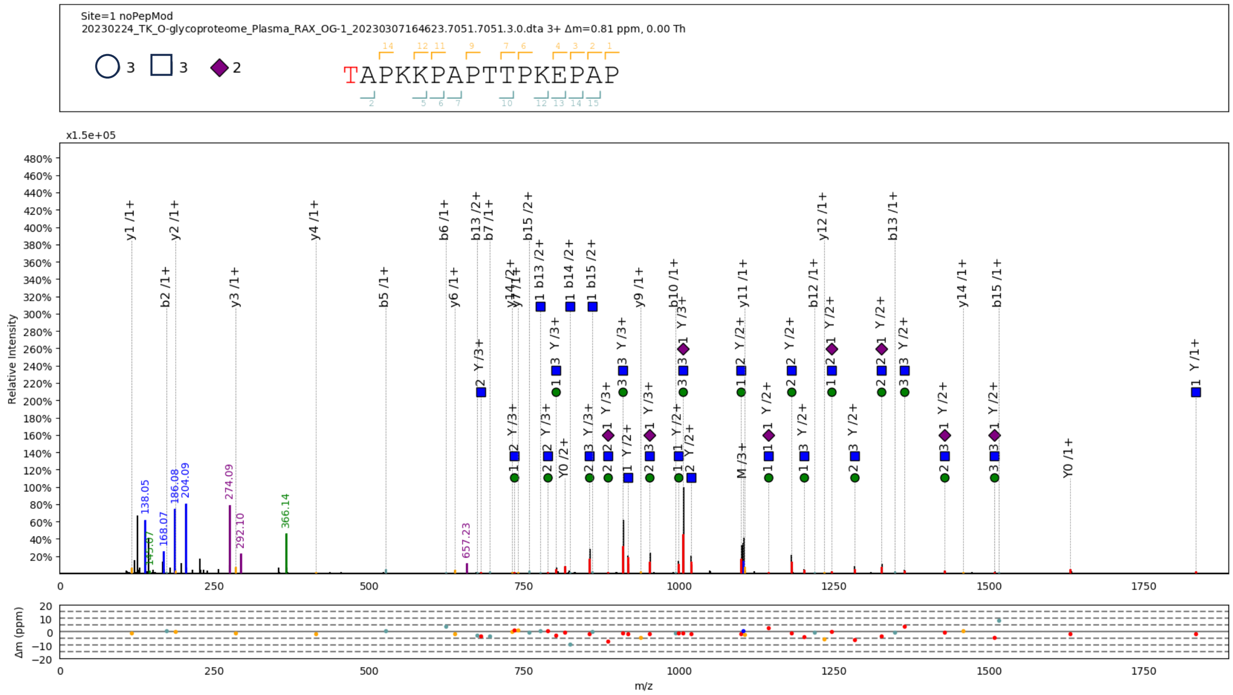

PRG4–T485, H (2) N (2) A (2)

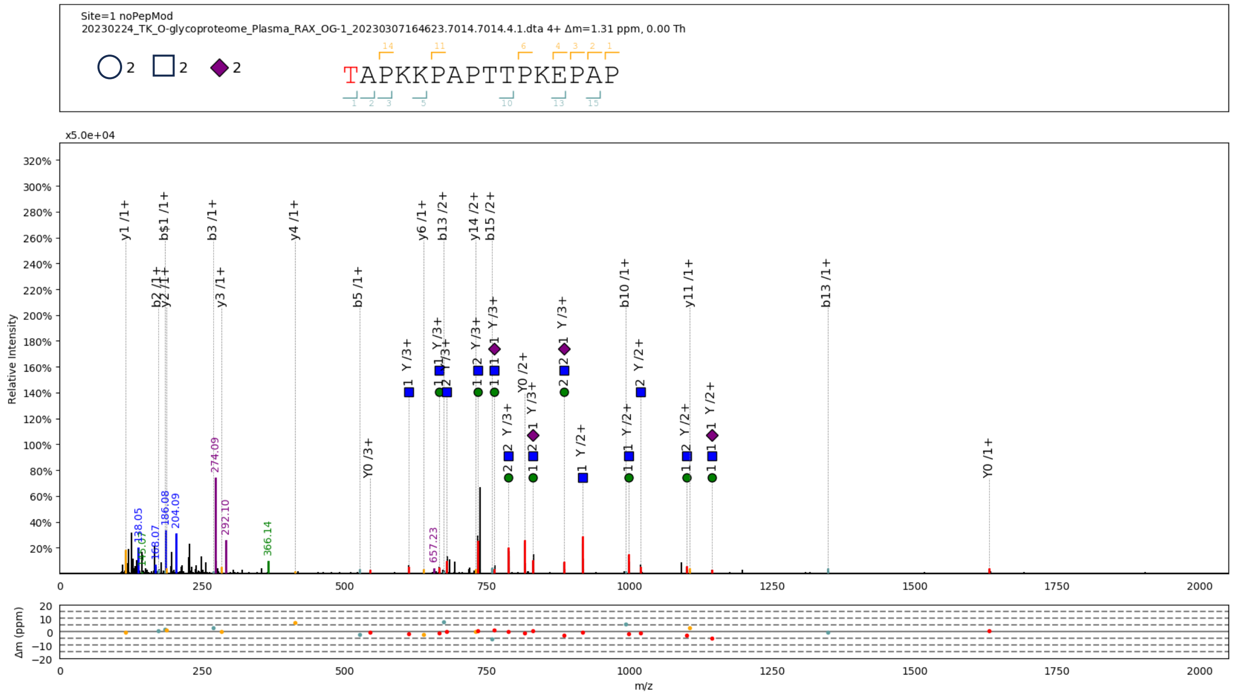

PRG4−T485, H (1) N (1) A (1)

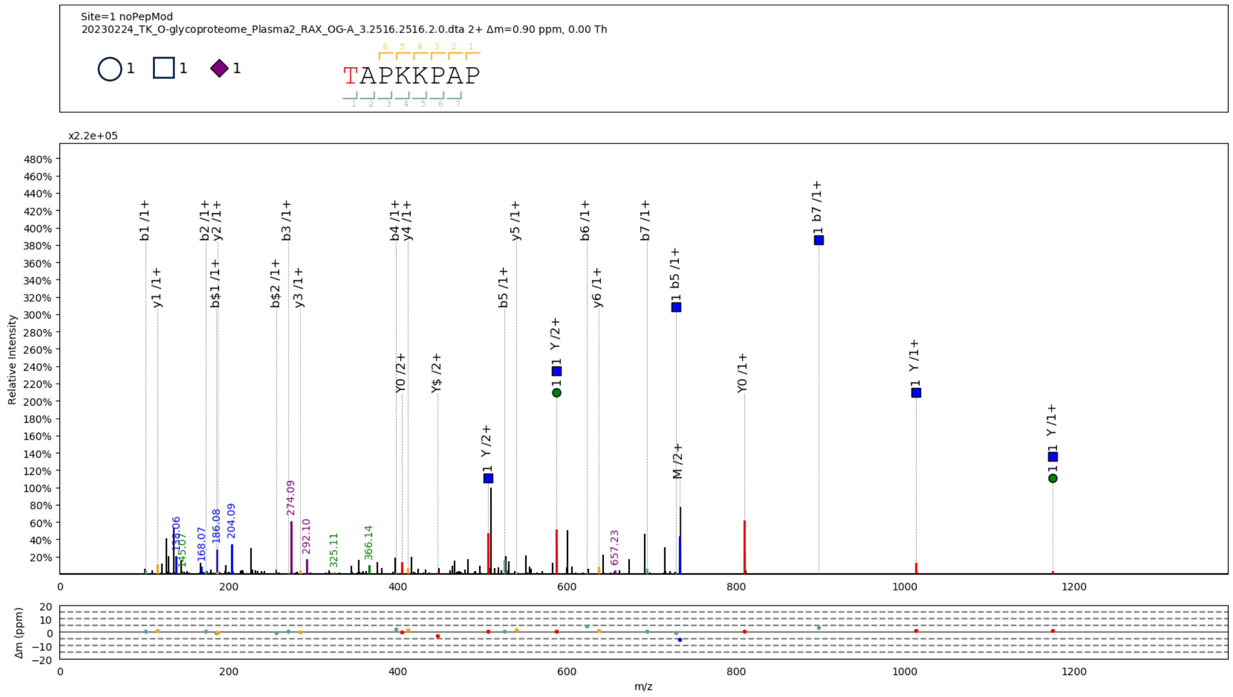

PRG4−T485, H (1) N (1)

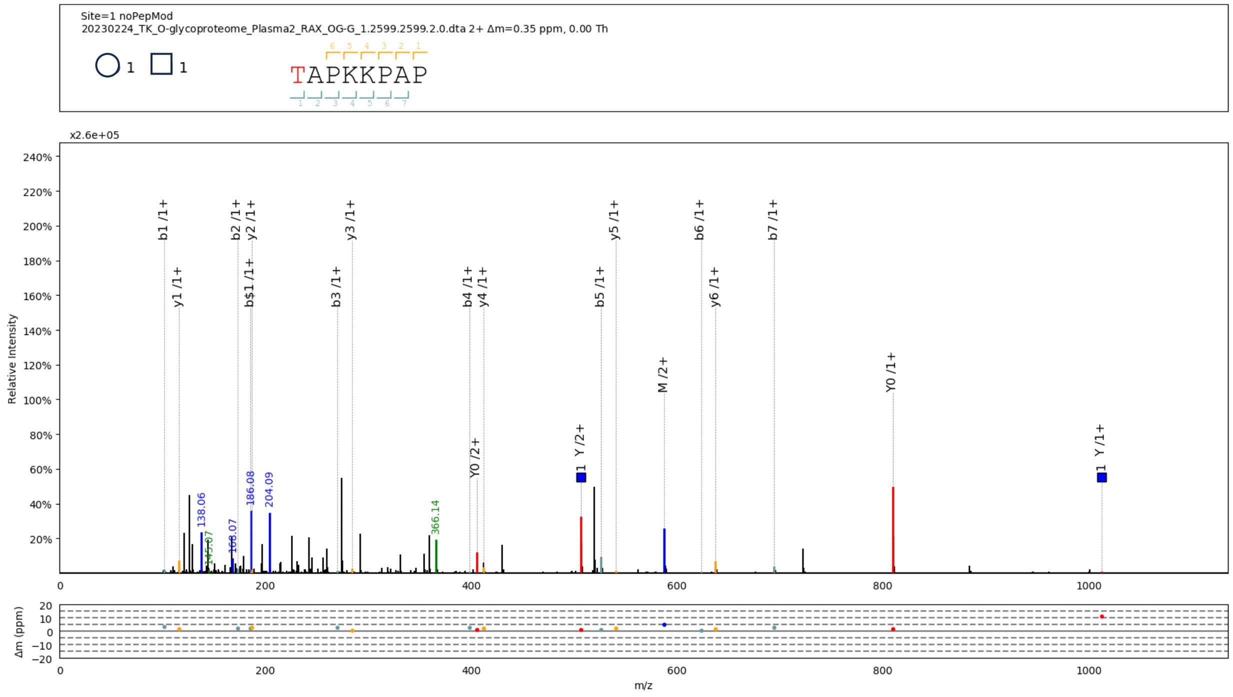

PRG4–S544, H (1) N (1) A (1)

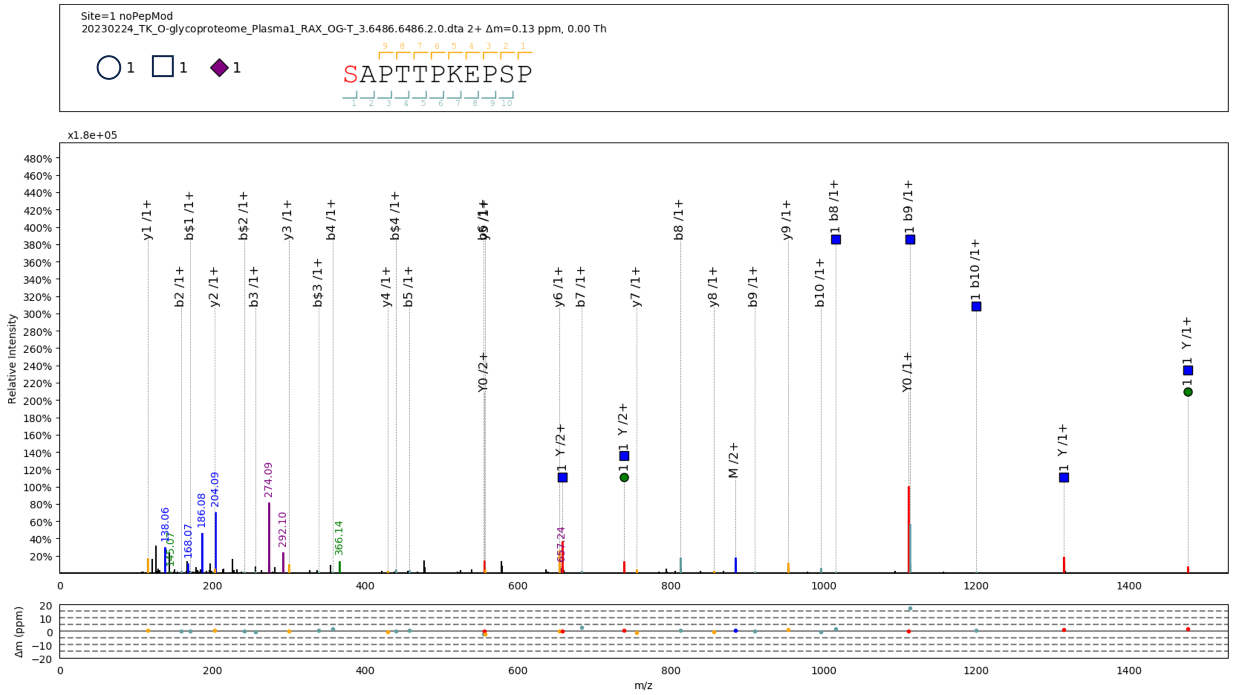

PRG4–T595, H (2) N (2) A (2)

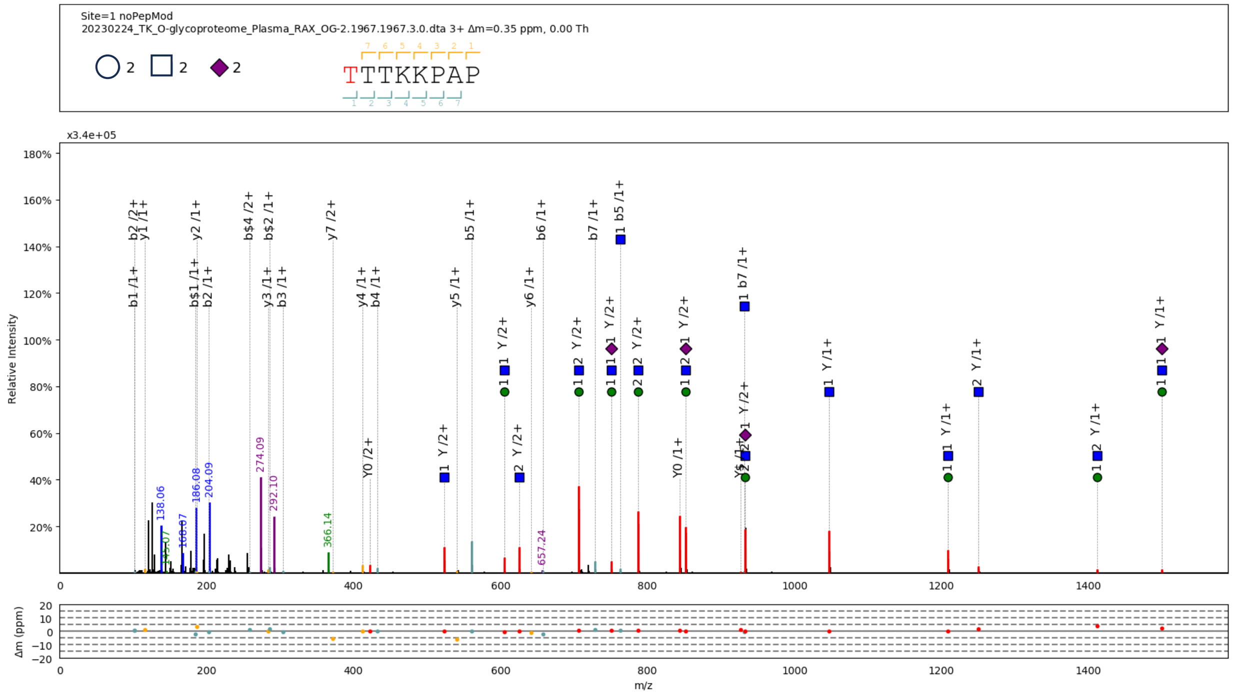

PRG4–T595, H (2) N (3) A (2)

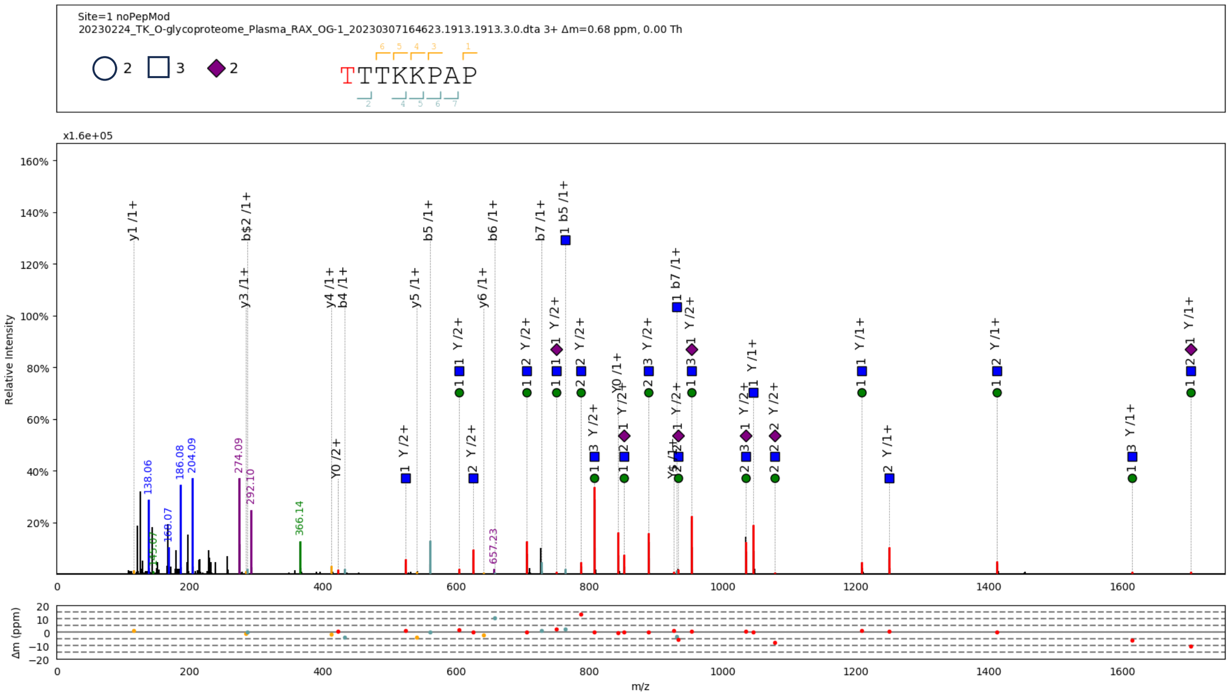

PRG4–T595, H (3) N (3) A (2)

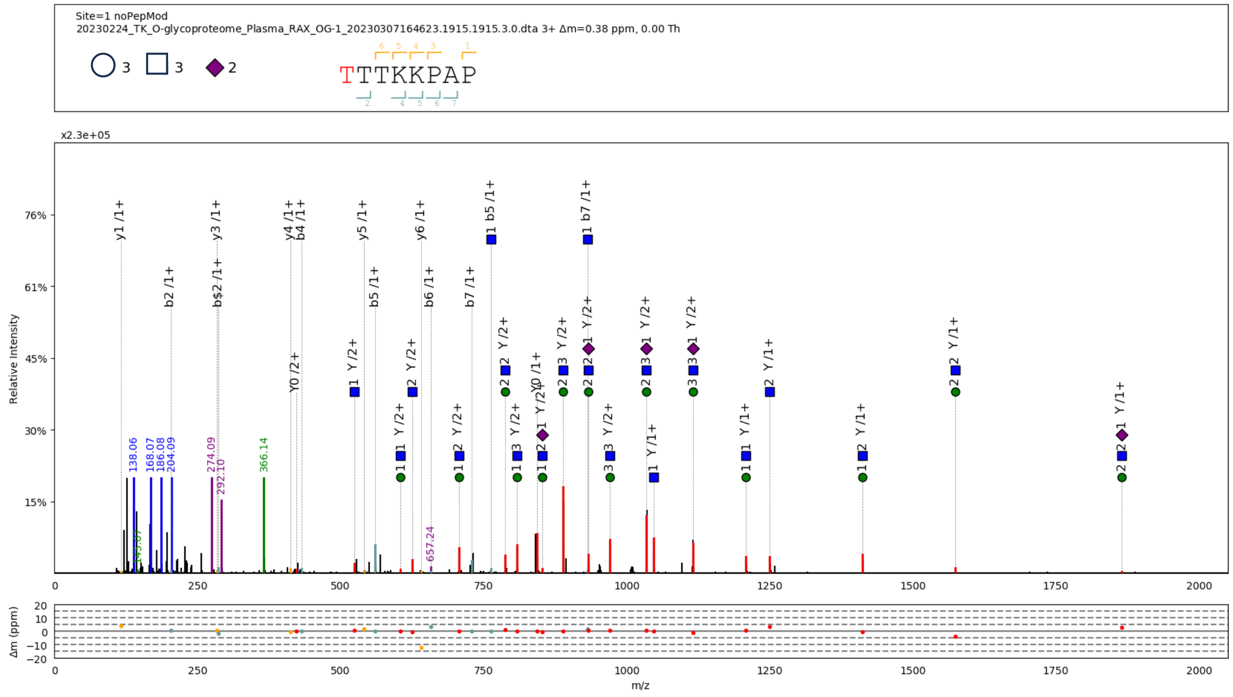

PRG4−T595, H (2) N (3) A (1)

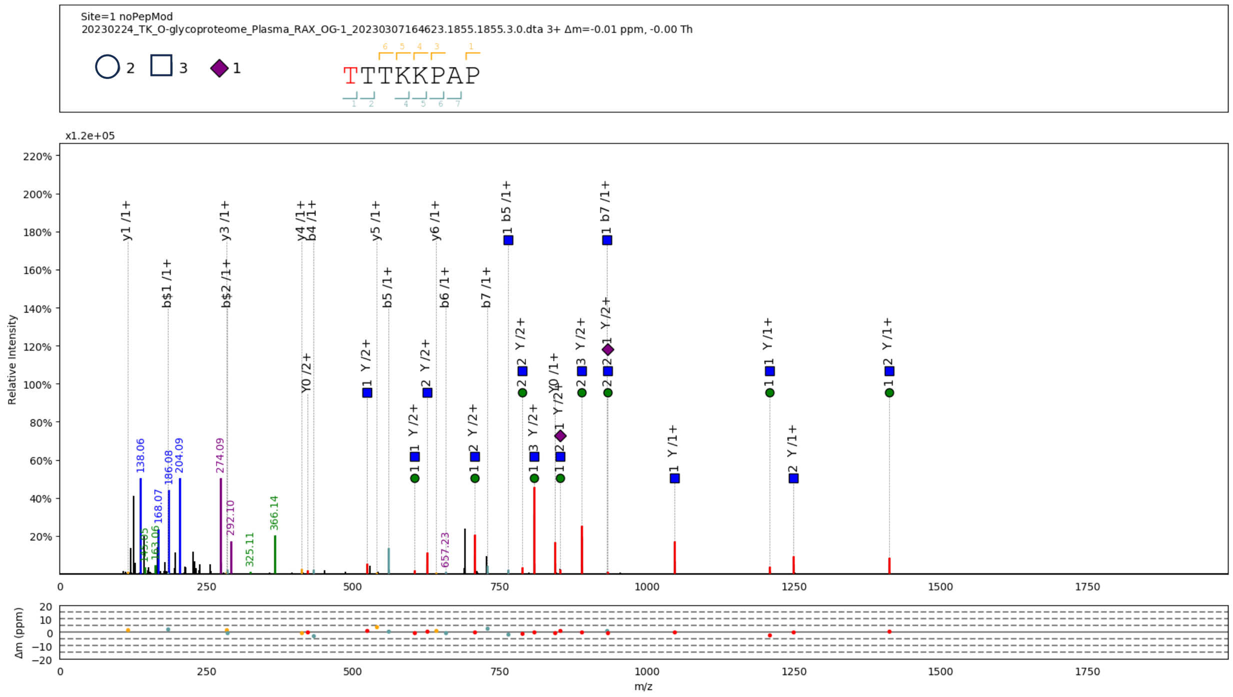

PRG4−T611-T699-T821, H (2) N (2) A (2)

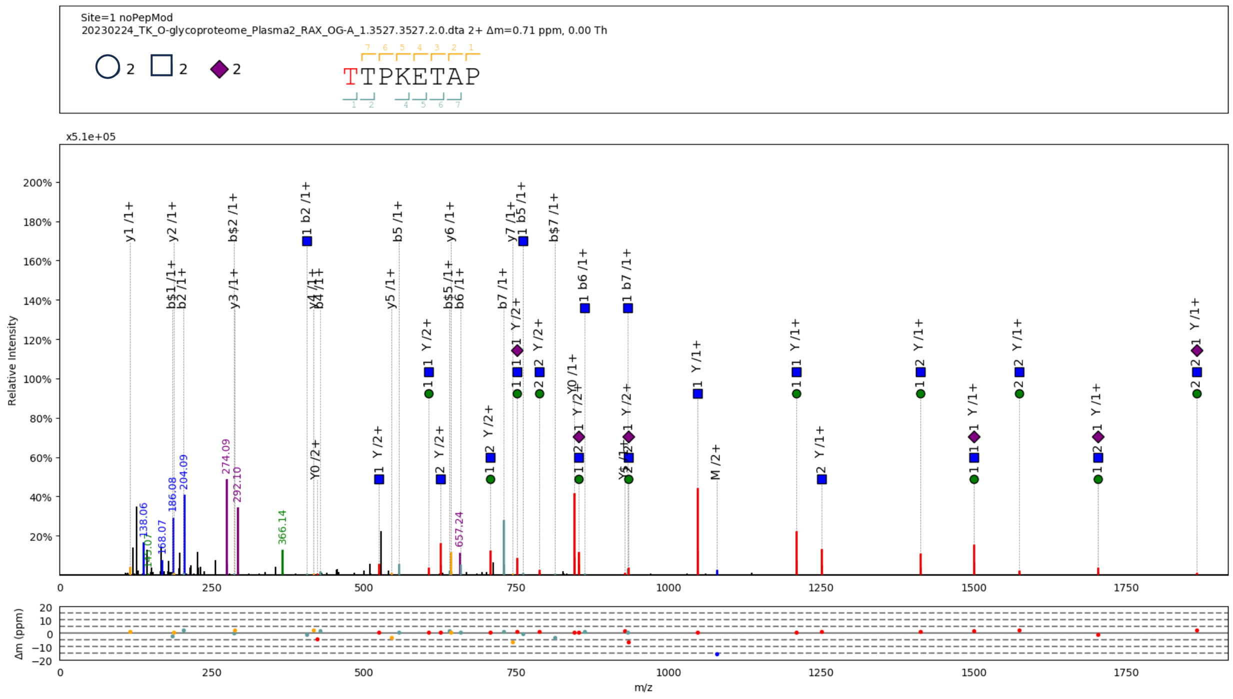

PRG4–T611-T699-T821, H (2) N (2) A (1)

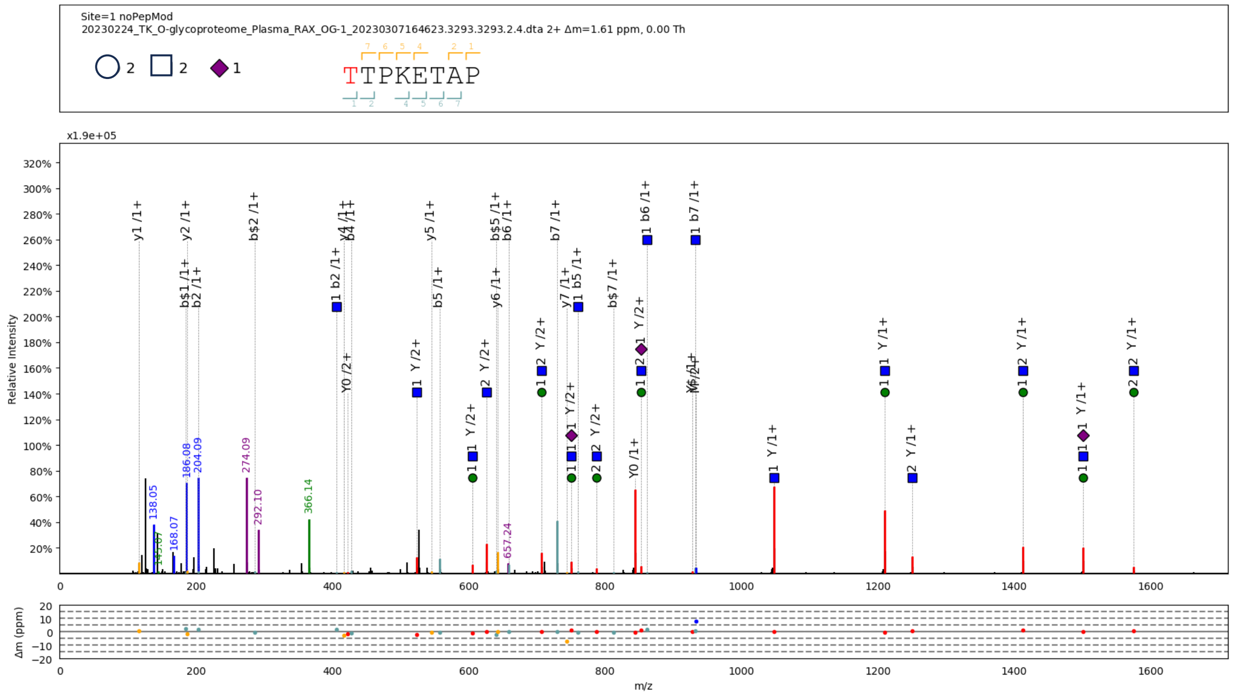

PRG4–T611-T699-T821, H (1) N (2) A (1)

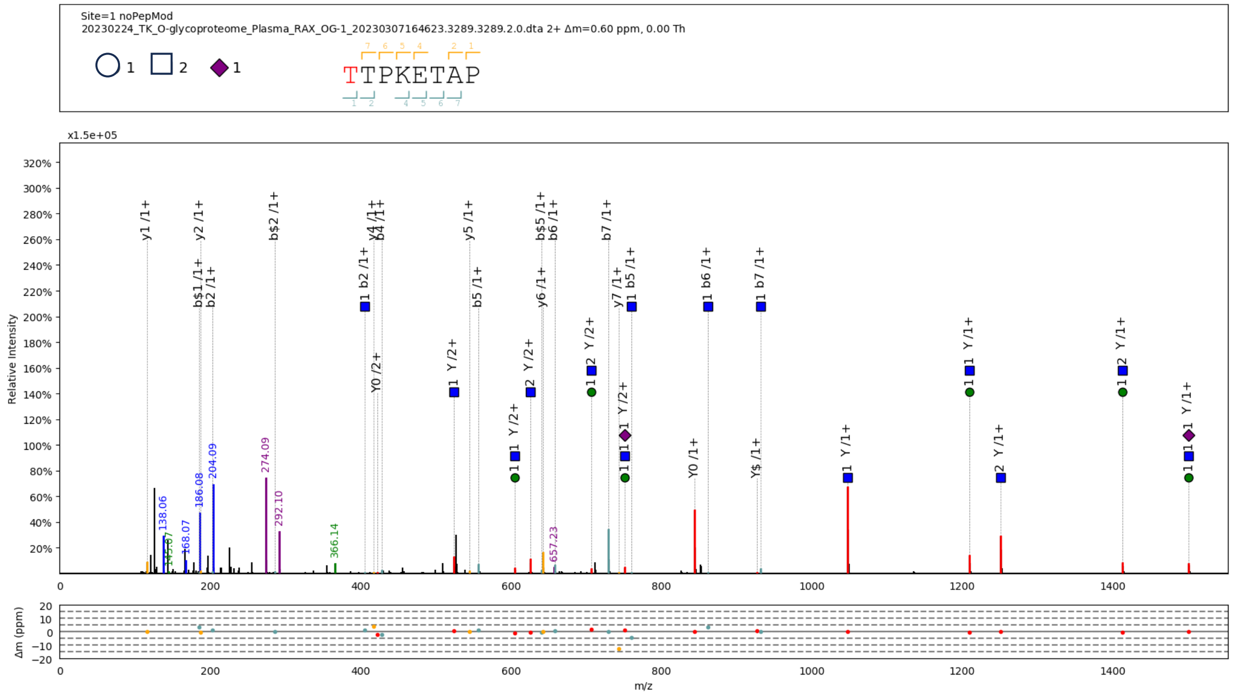

PRG4–T611-T699-T821, H (1) N (1) A (1)

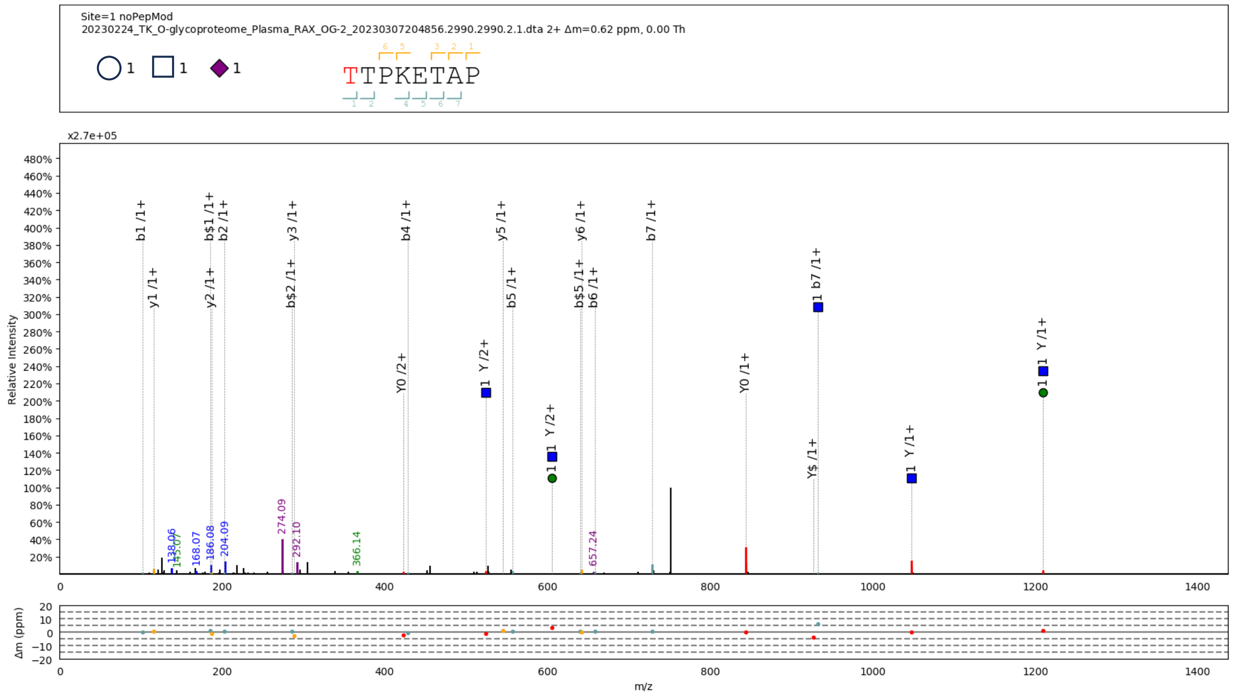

PRG4–T616-T704-T757-T826, H (2) N (2) A (2)

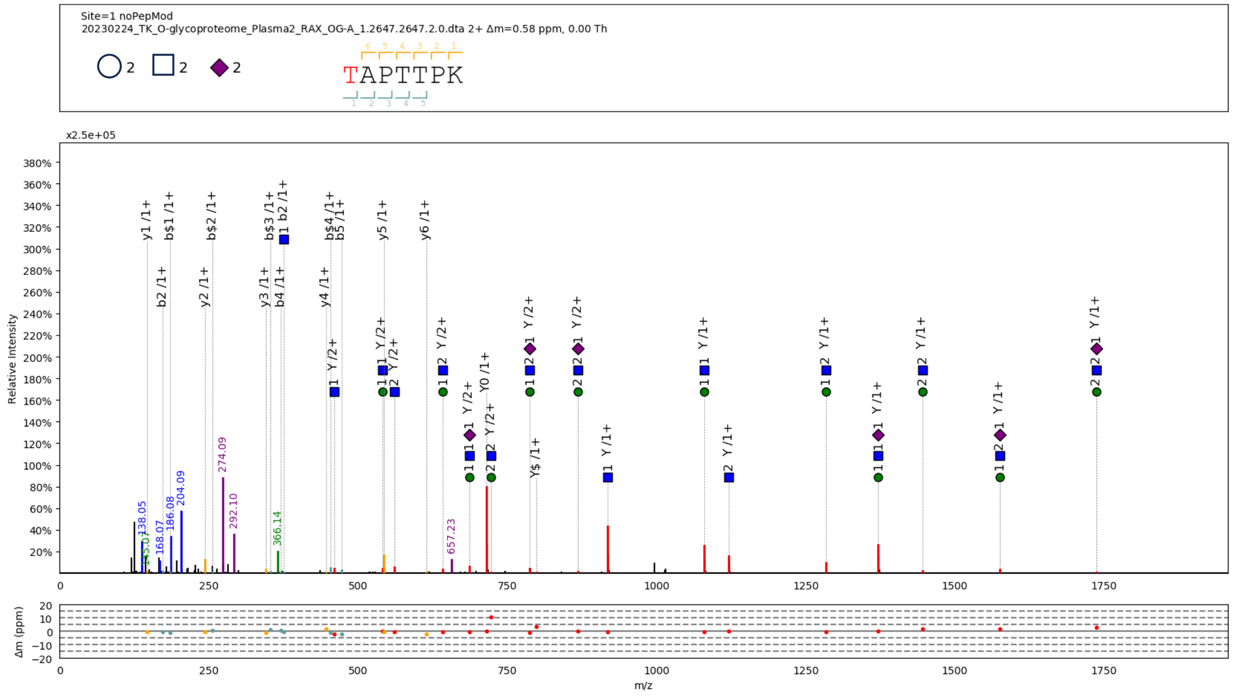

PRG4–T616-T704-T757-T826, H (2) N (2) A (1)

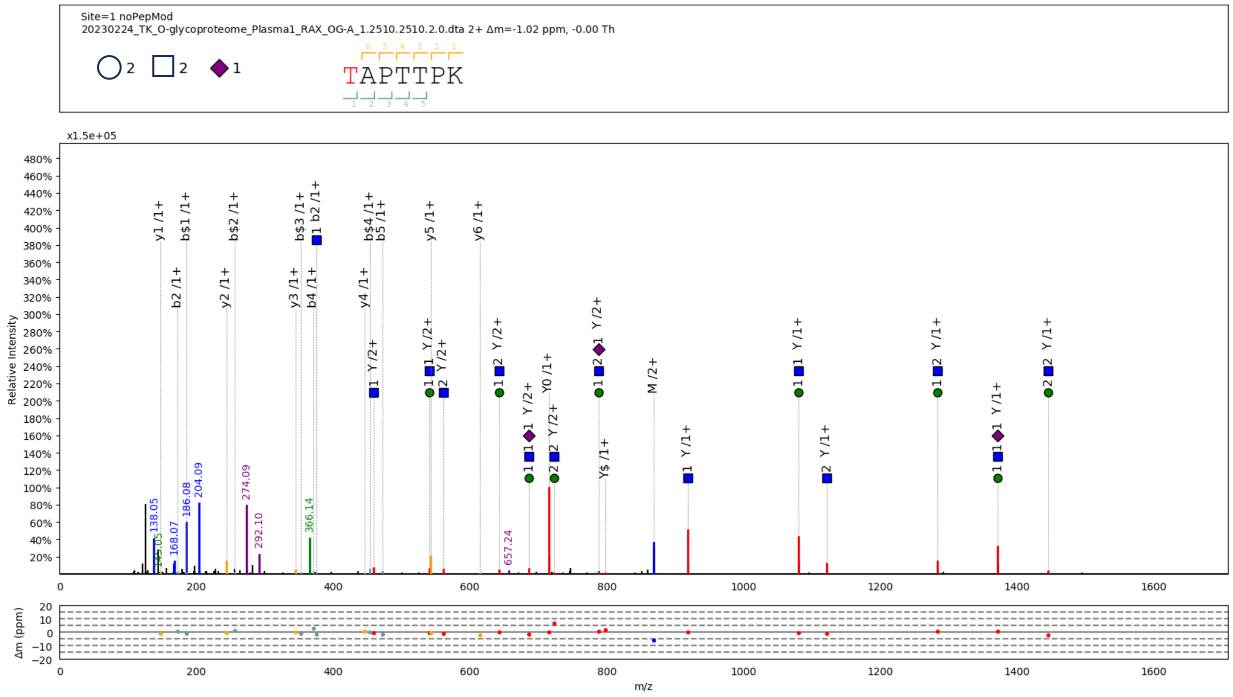

PRG4–T619, H (2) N (2) A (2)

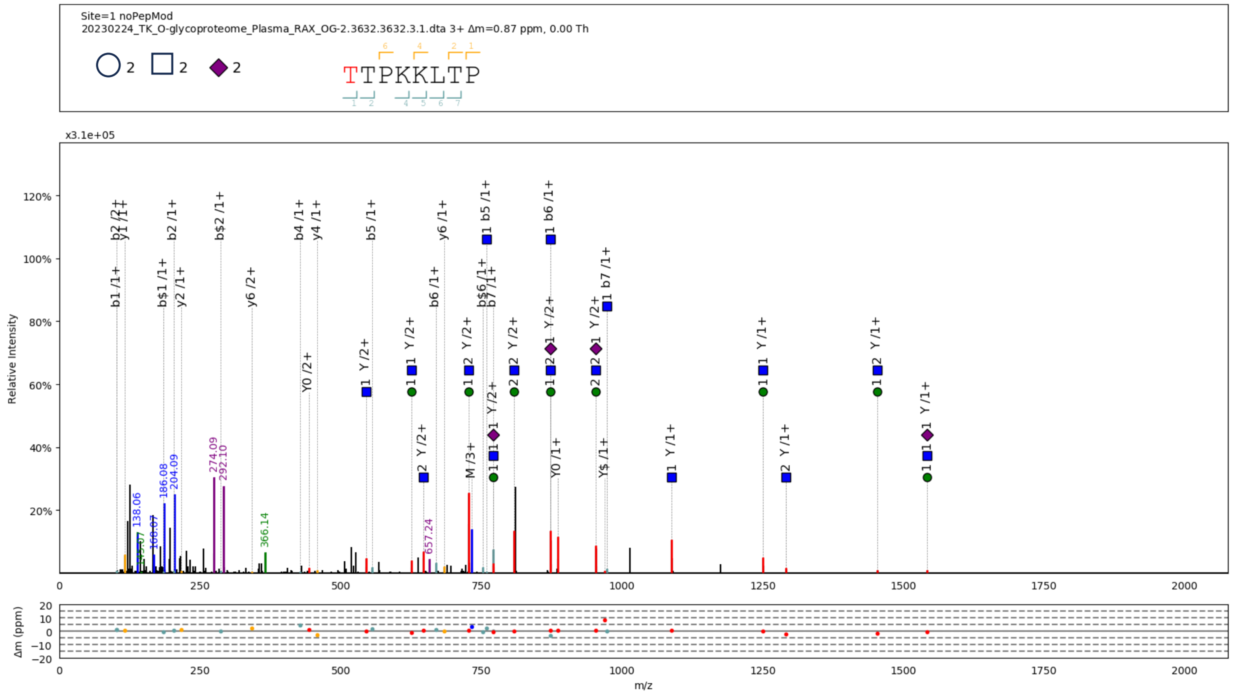

PRG4–T635, H (2) N (2) A (2)

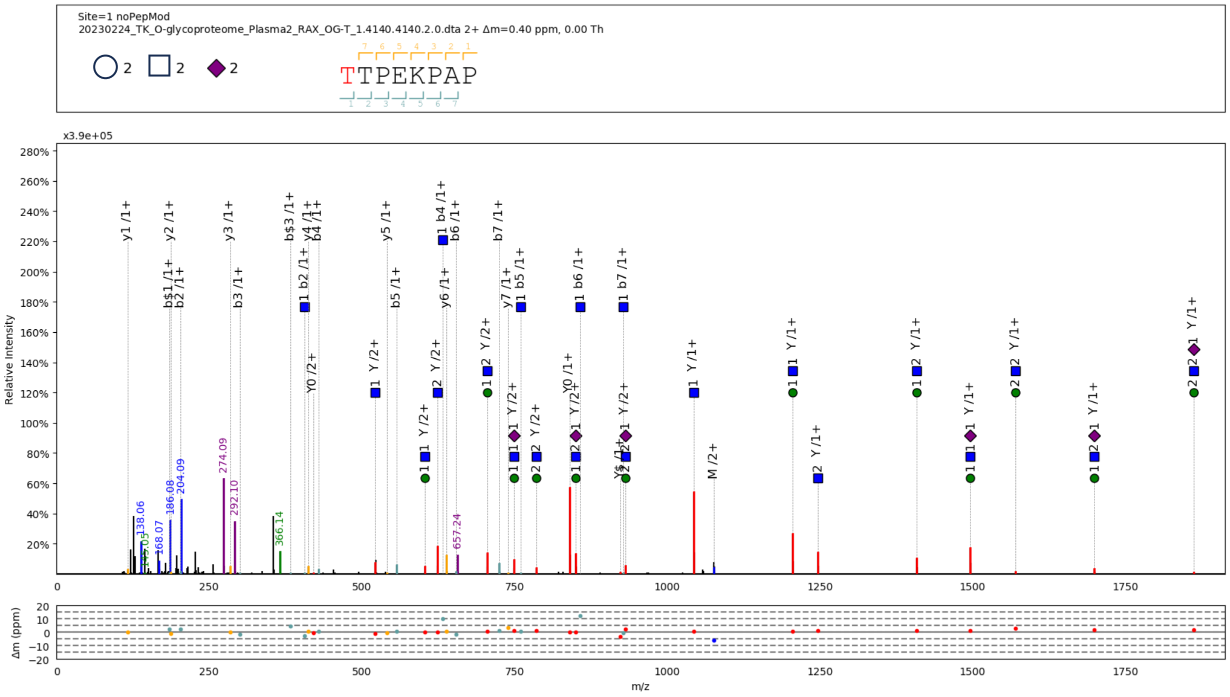

PRG4–T635, H (2) N (2) A (1)

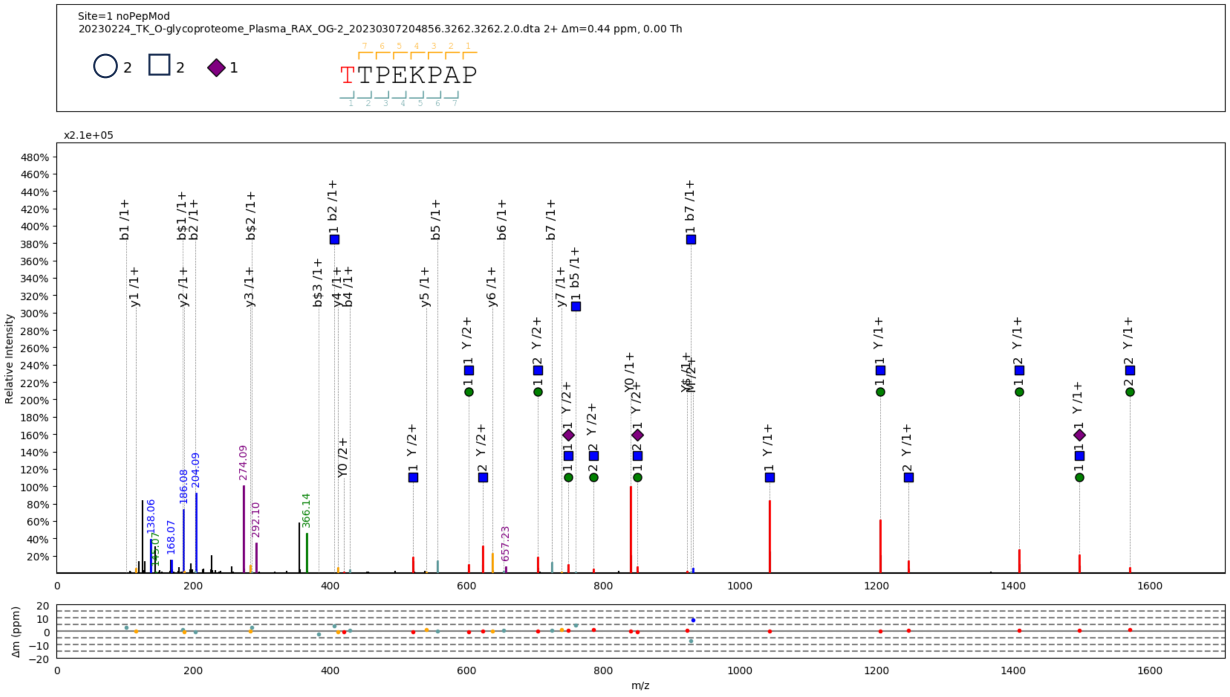

PRG4–T635, H (1) N (1) A (1)

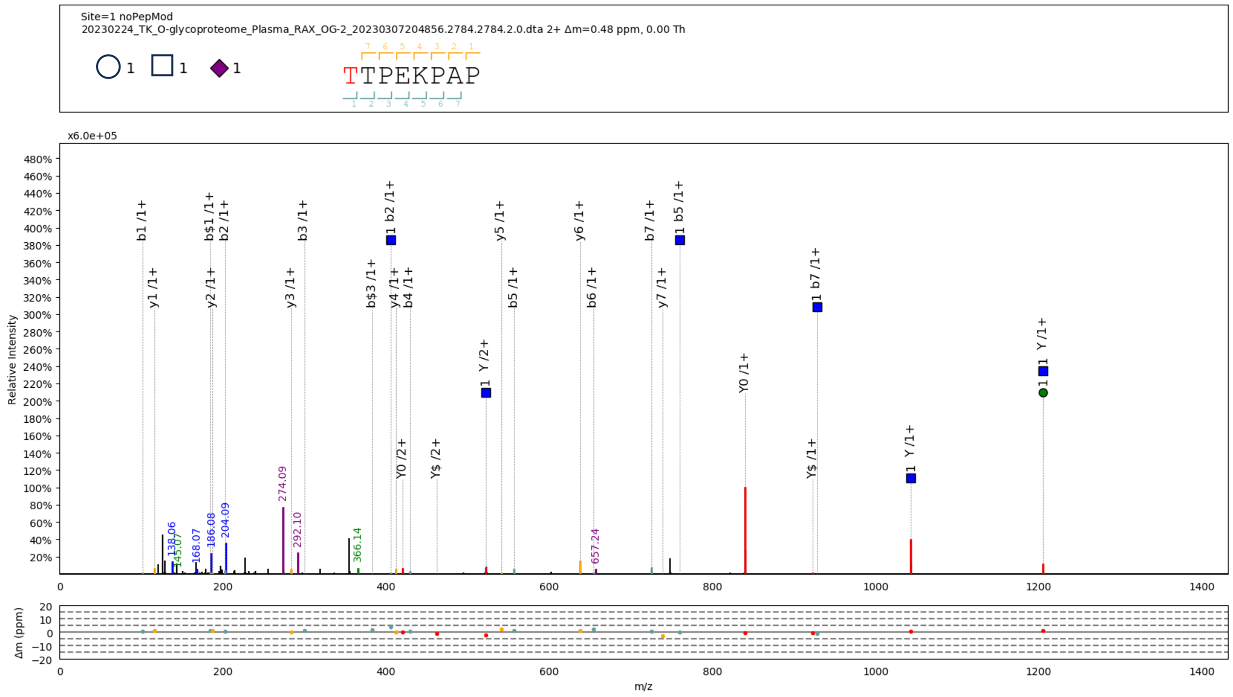

PRG4–T635, H (1) N (2) A (1)

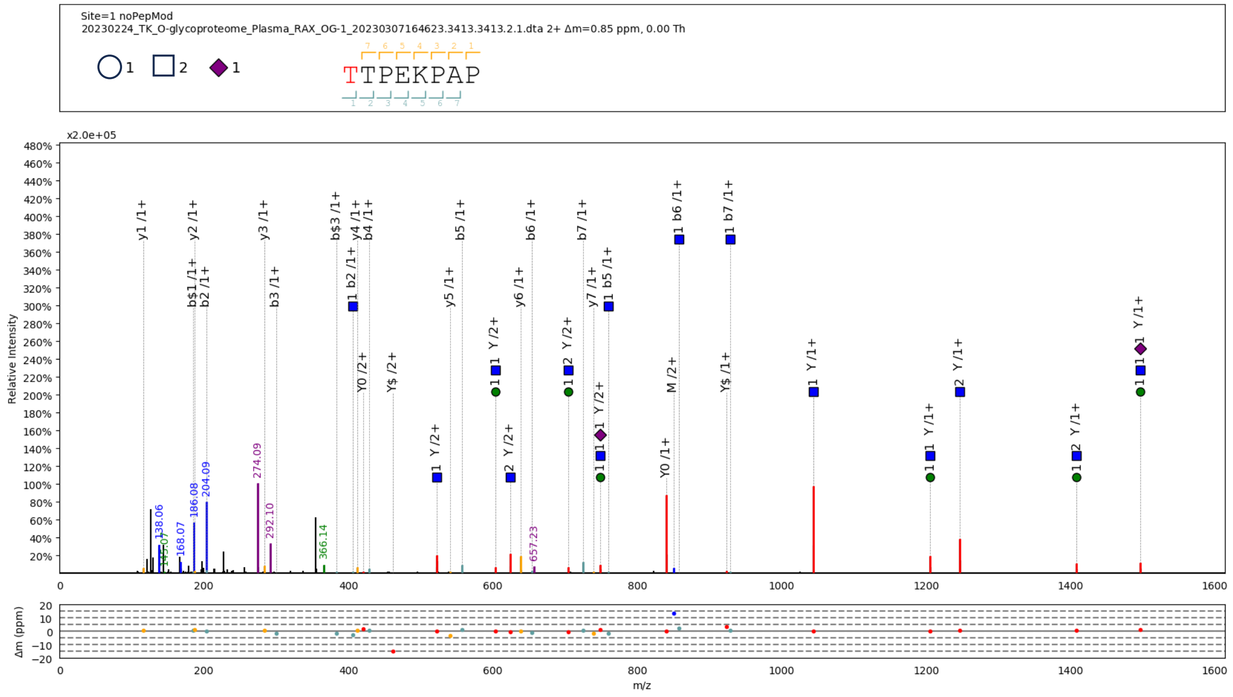

PRG4–T635, H (1) N (1) A (2)

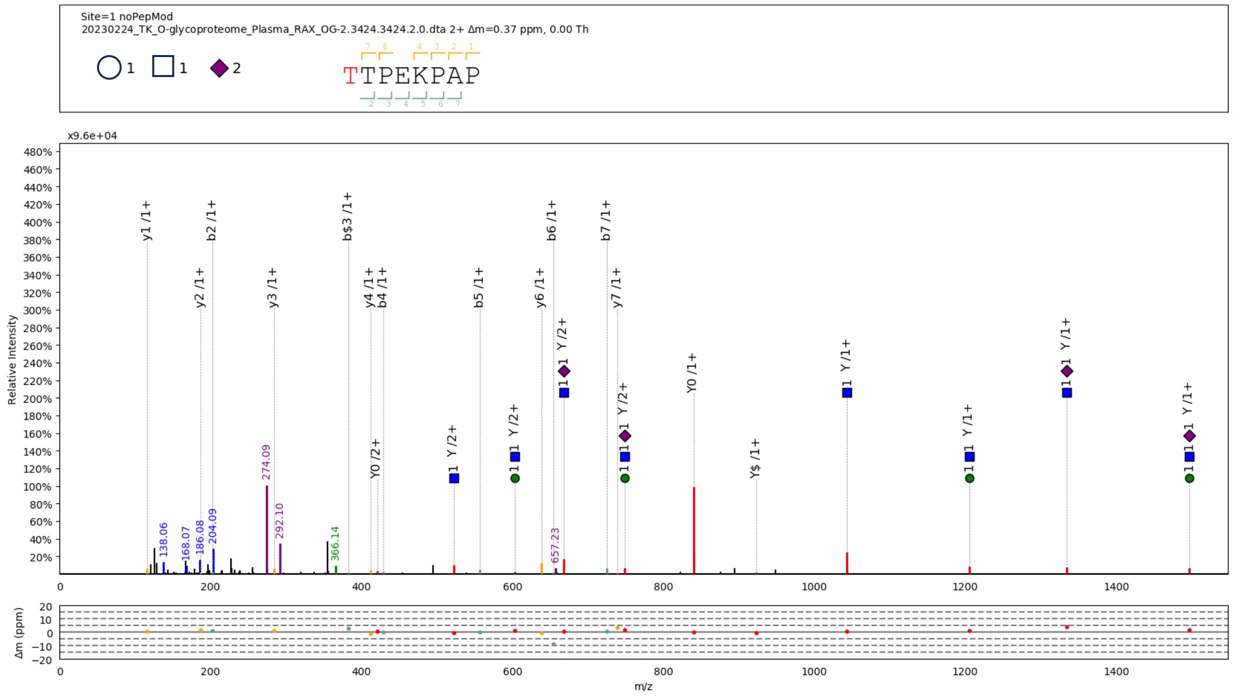

PRG4–T635, H (1) N (1)

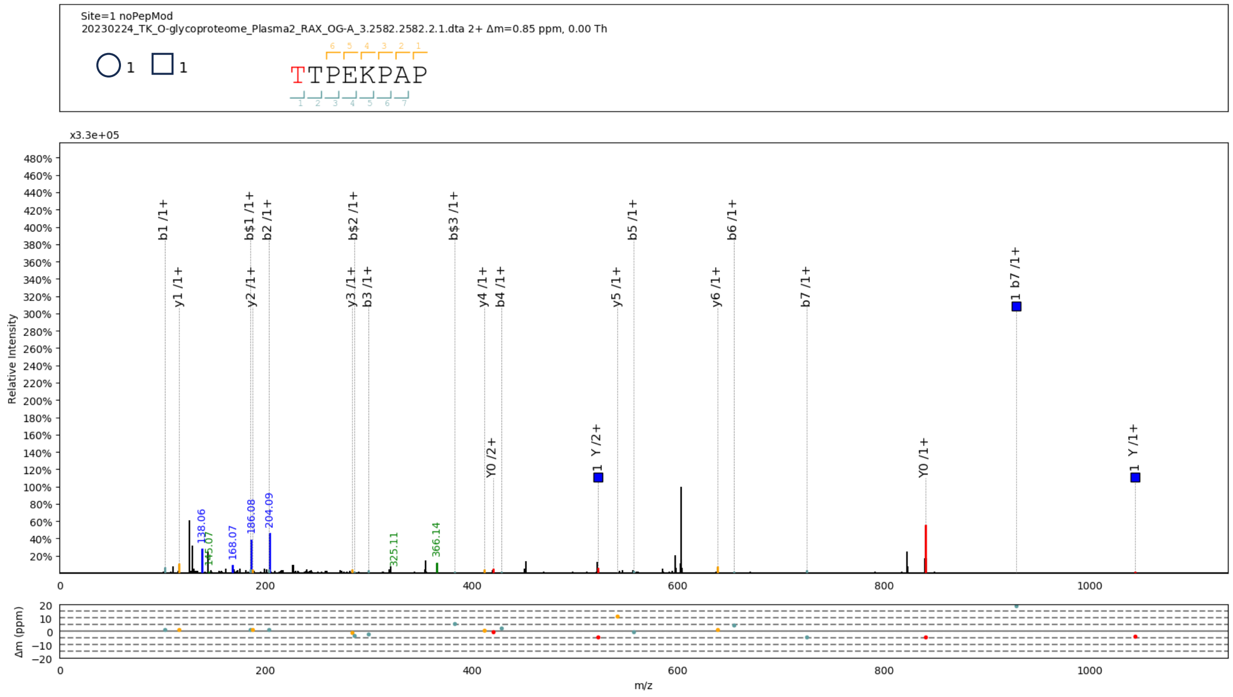

PRG4−T635, H (3) N (2) A (1) F (1)

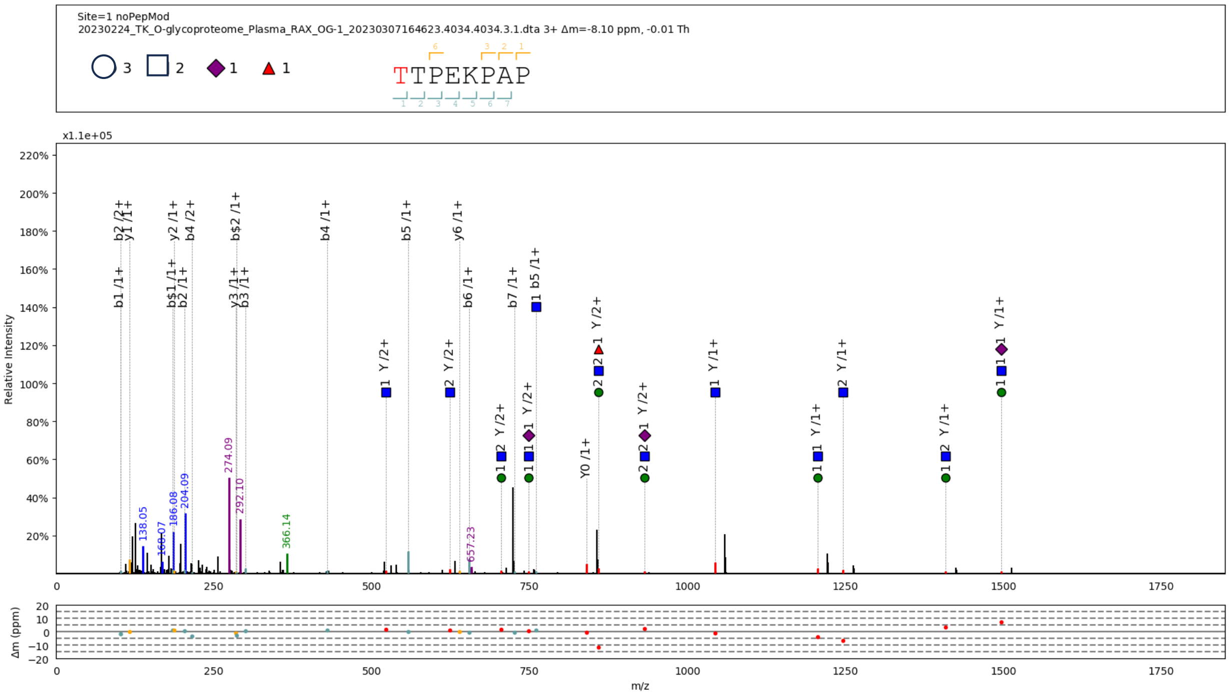

PRG4−T651, H (1) N (1) A (1)

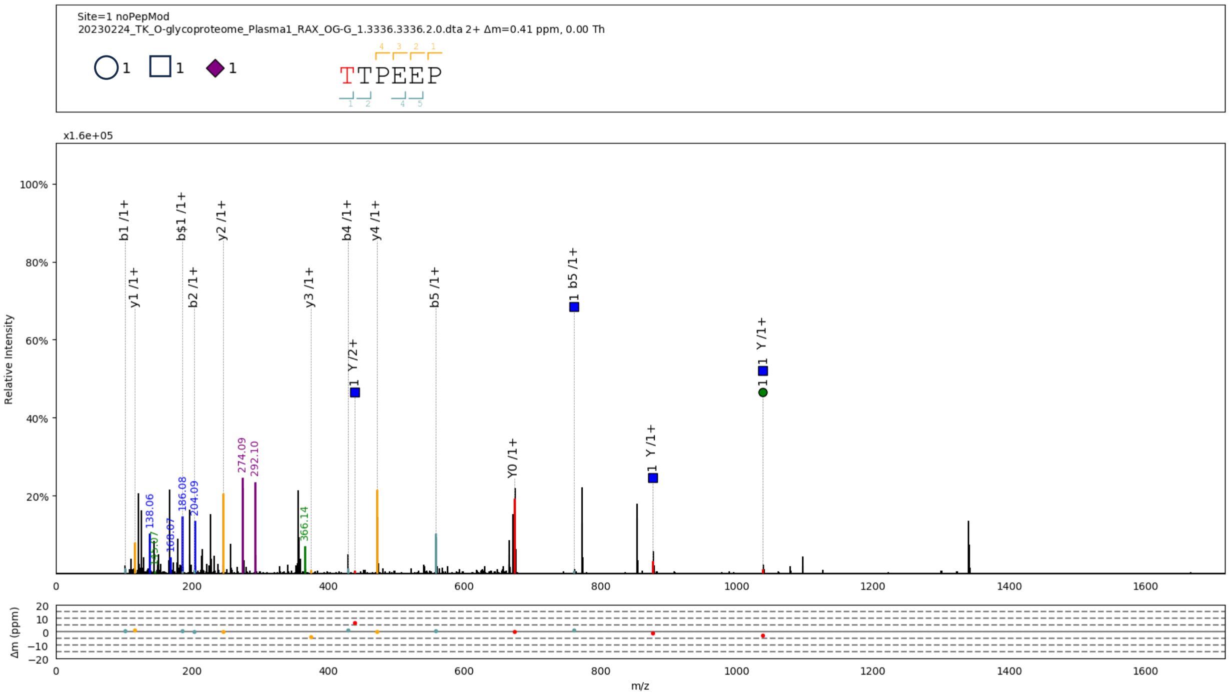

PRG4–T657, H (2) N (2) A (2)

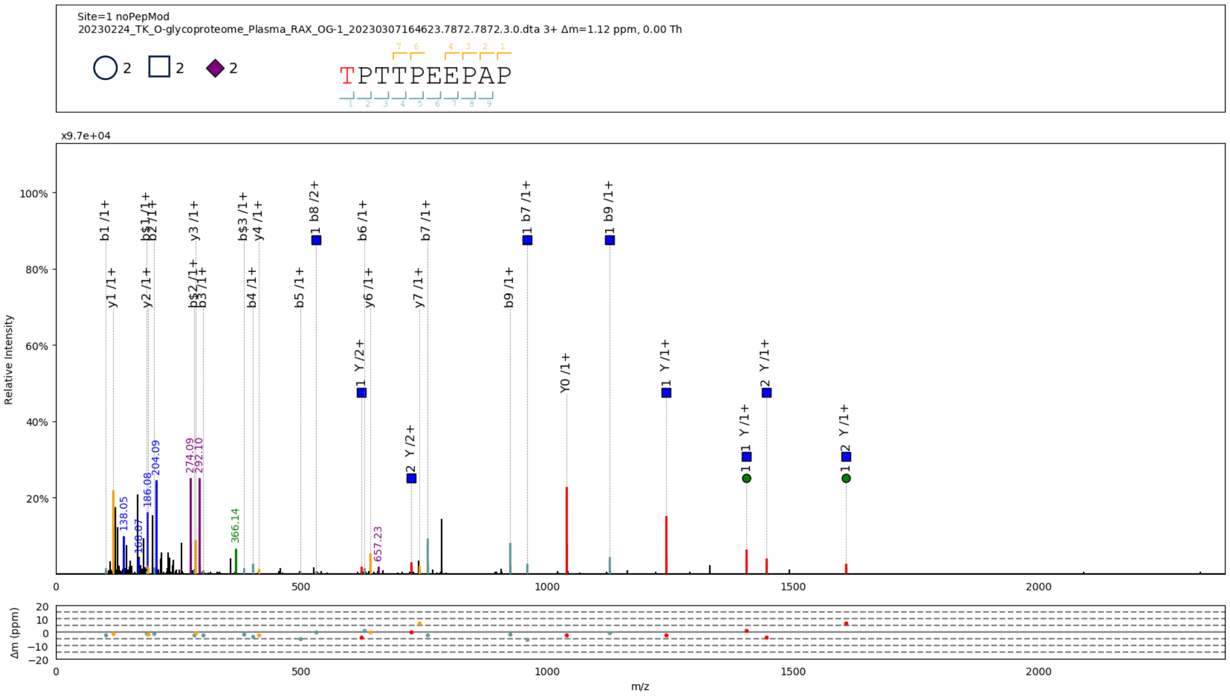

PRG4–T667, H (3) N (3) A (3)

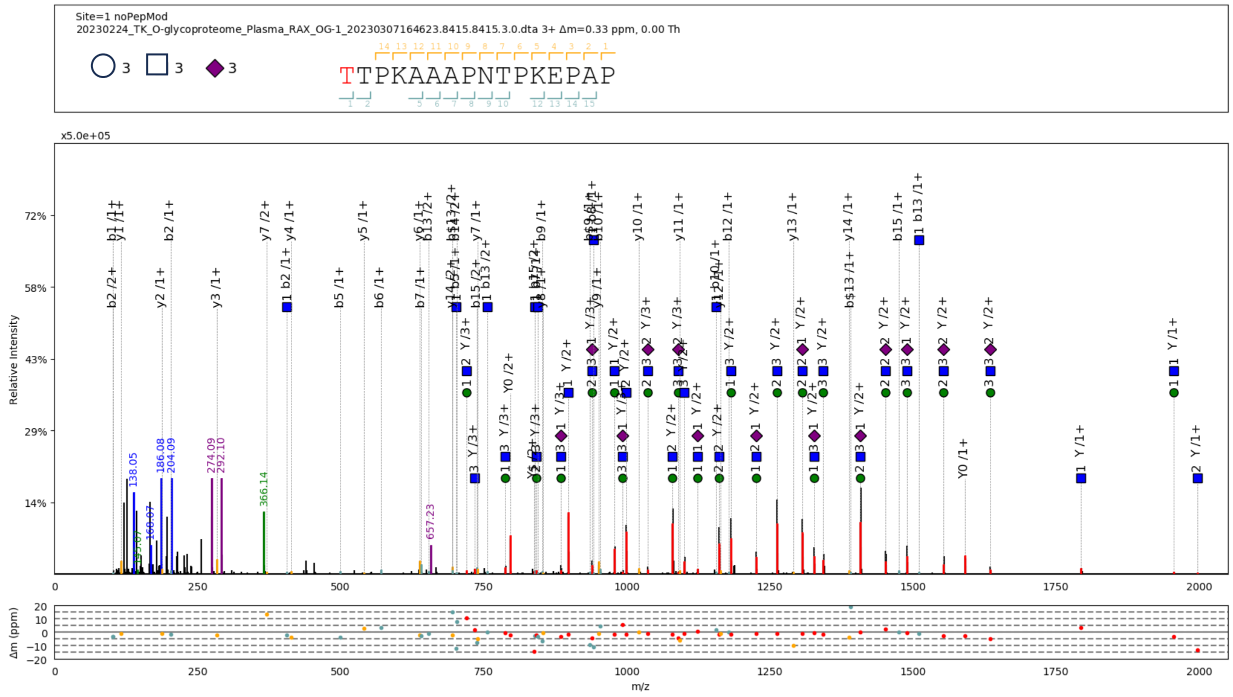

PRG4–T667, H (2) N (2) A (2)

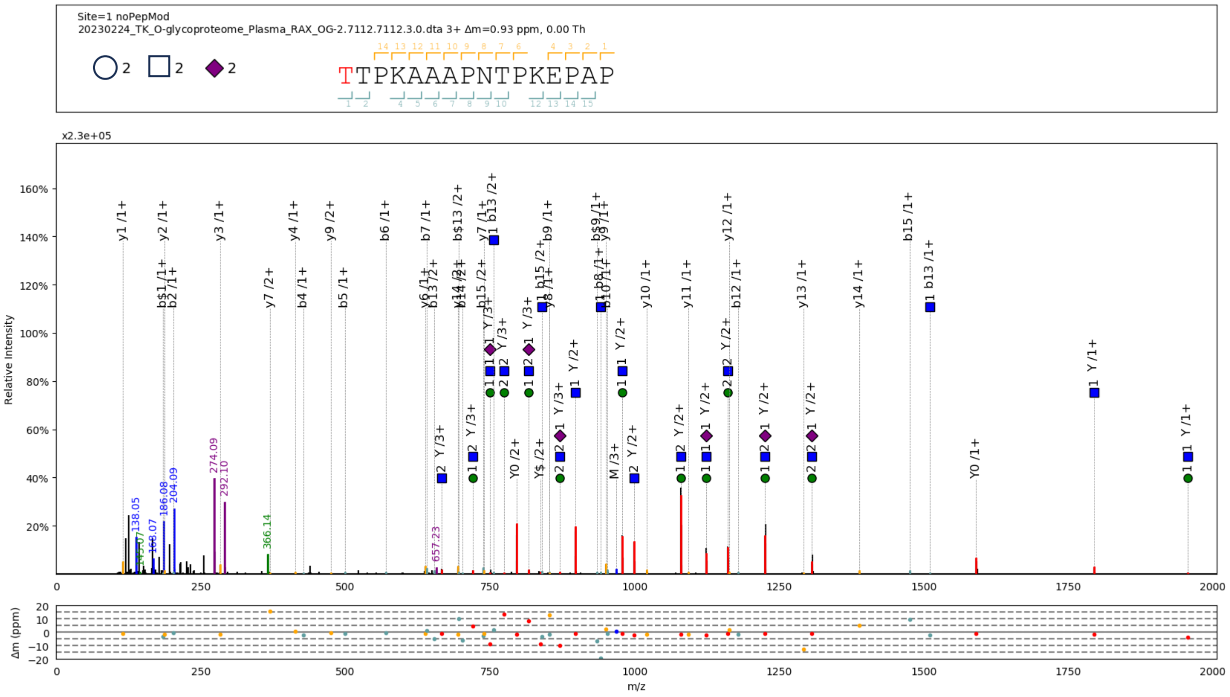

PRG4–T668, H (2) N (2) A (2)

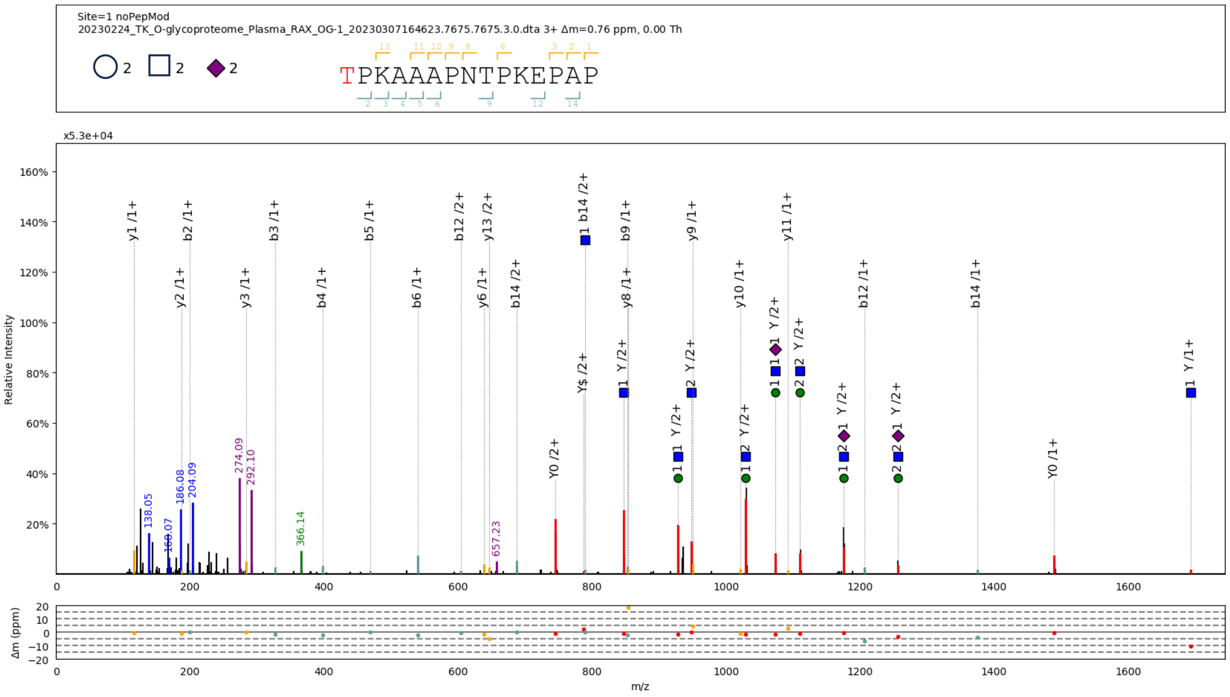

PRG4−T668, H (1) N (1) A (1)

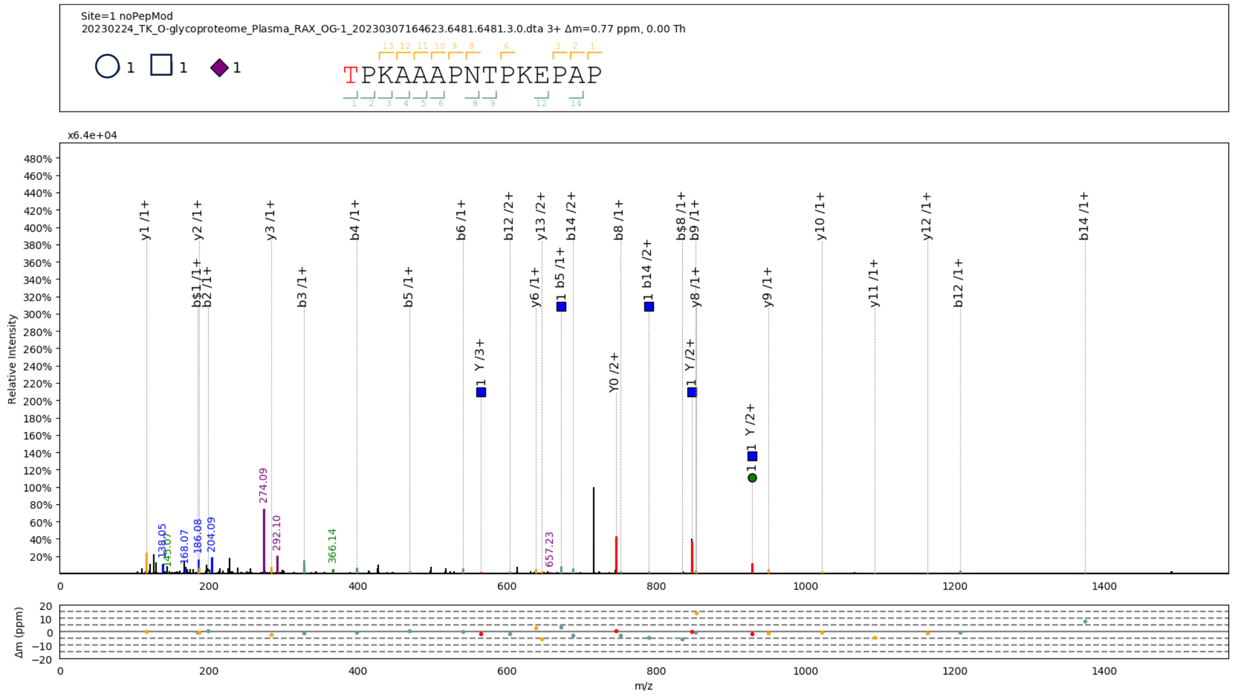

PRG4−T712, H (2) N (2) A (2)

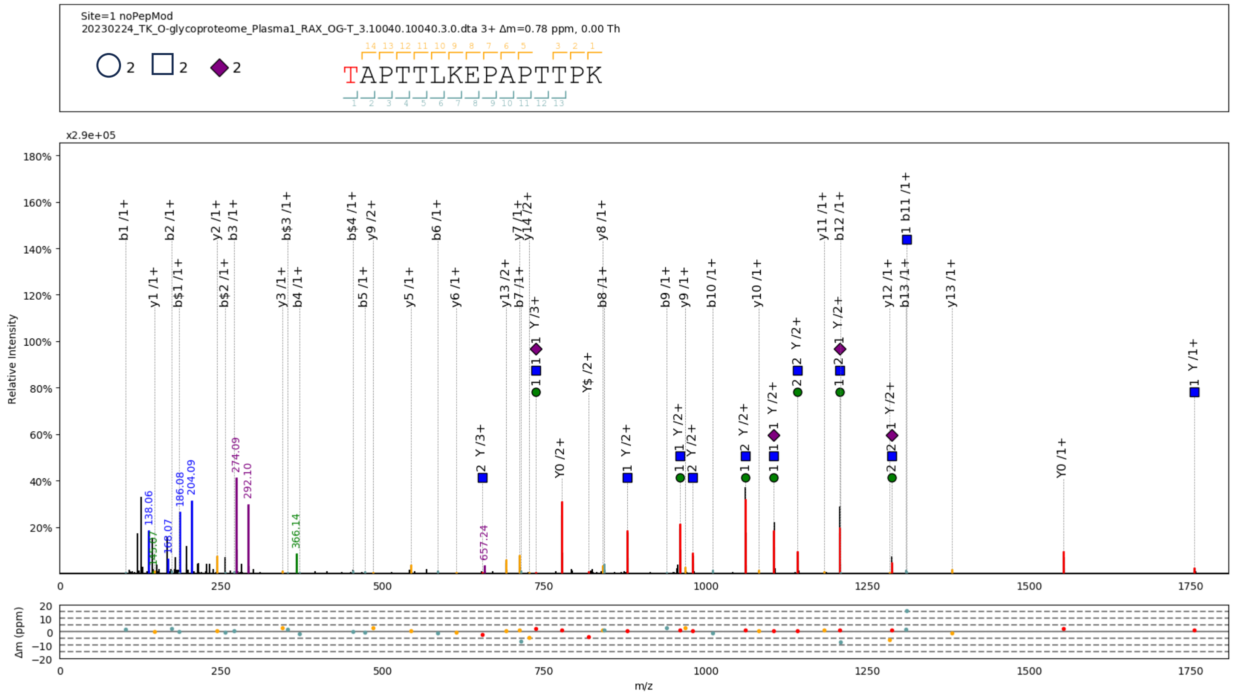

PRG4–T712, H (1) N (1) A (1)

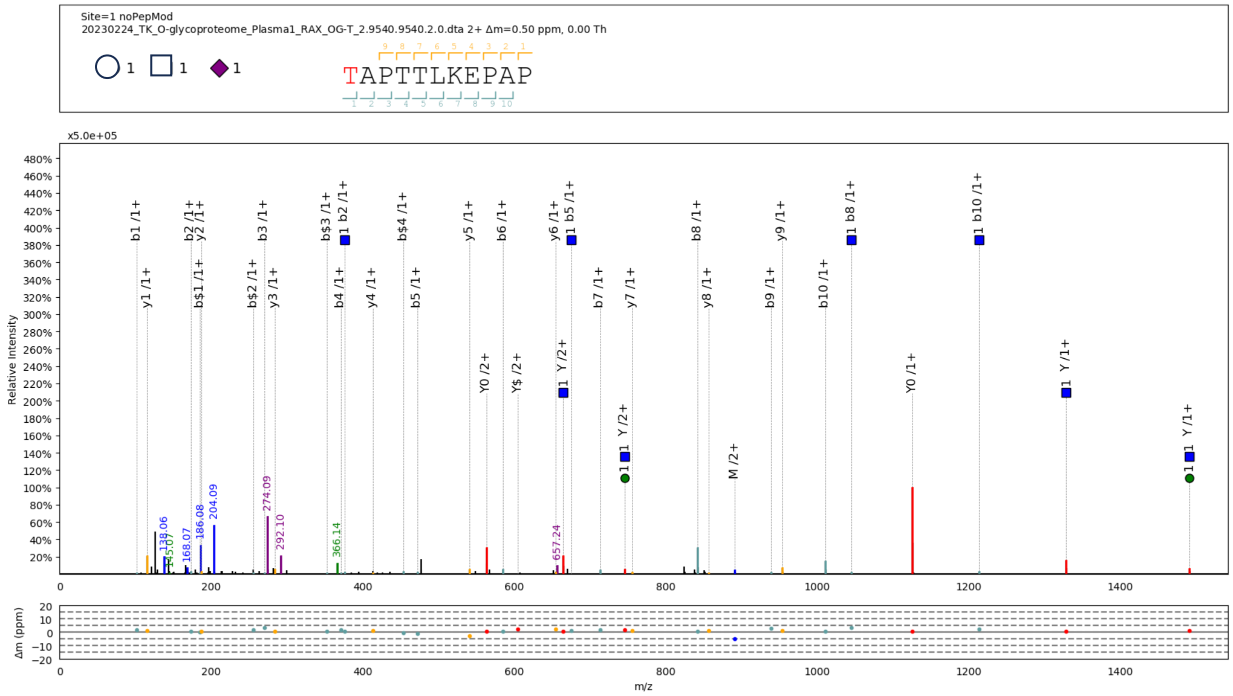

PRG4–T712, H (2) N (2) A (1)

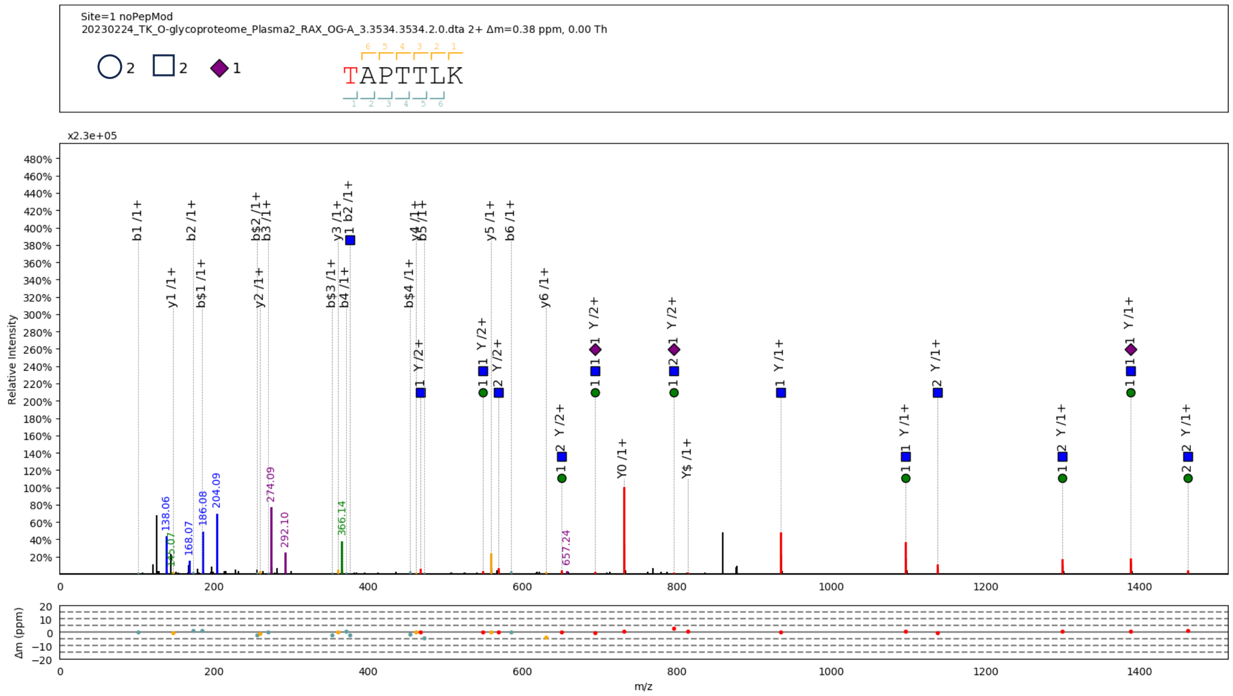

PRG4–T712, H (1) N (1)

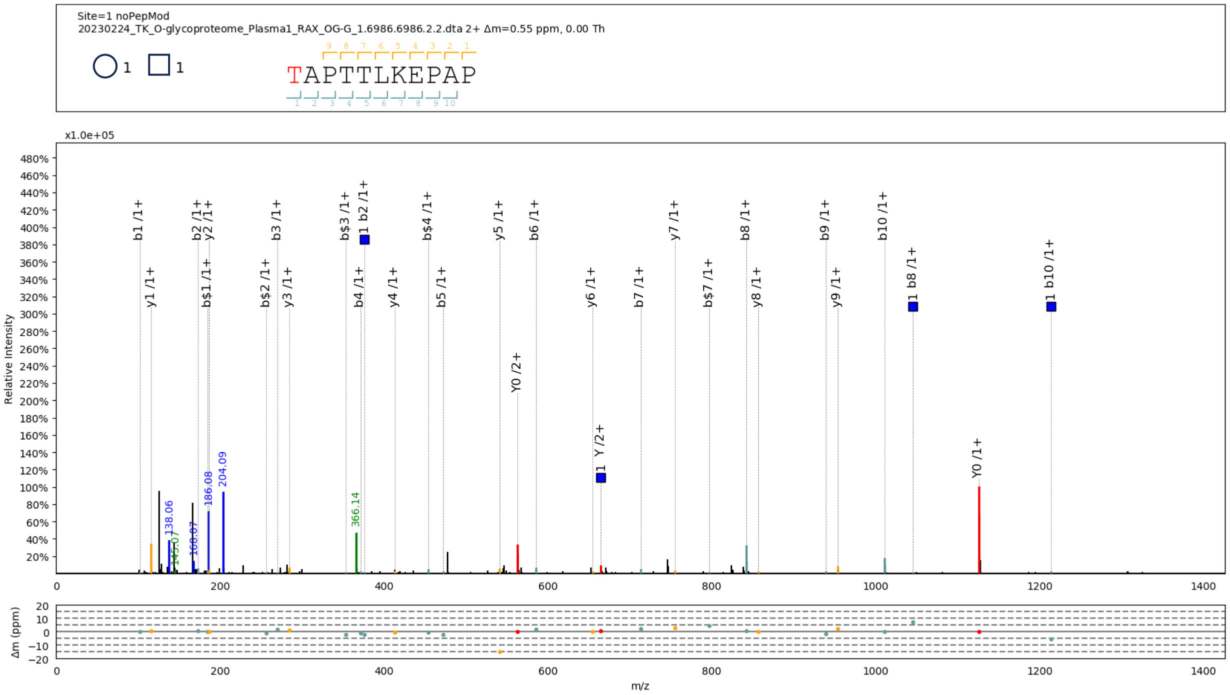

PRG4–T712, N (1)

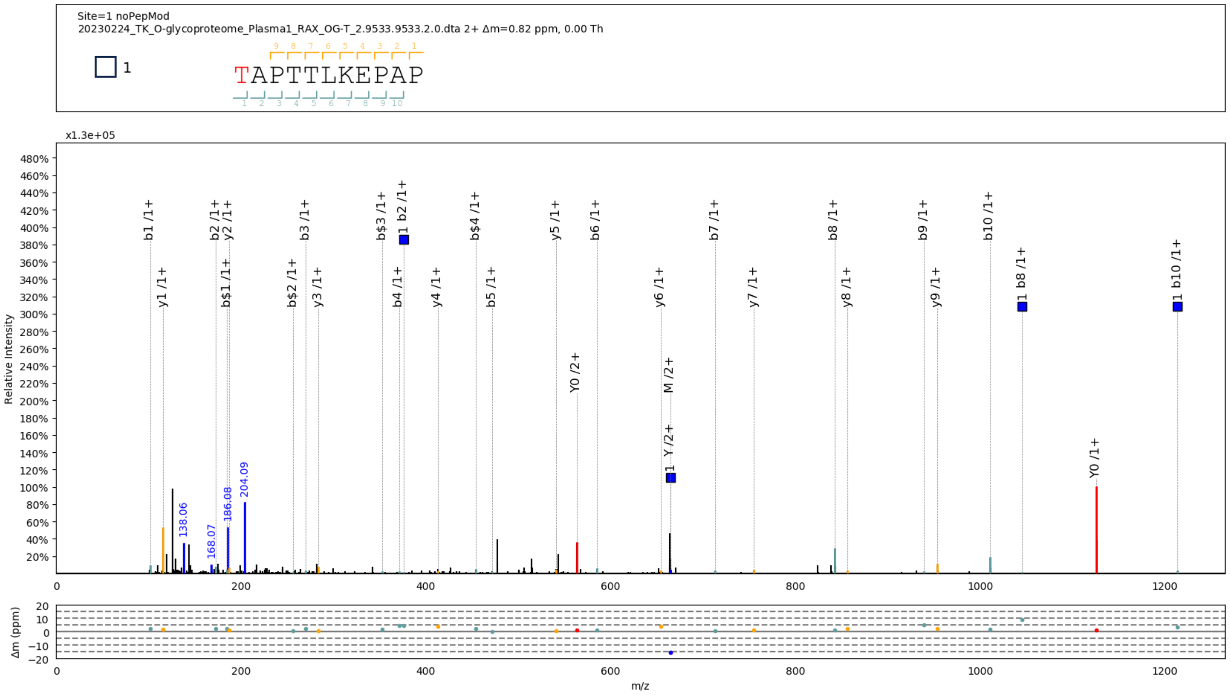

PRG4–T712, H (1) N (2) A (1)

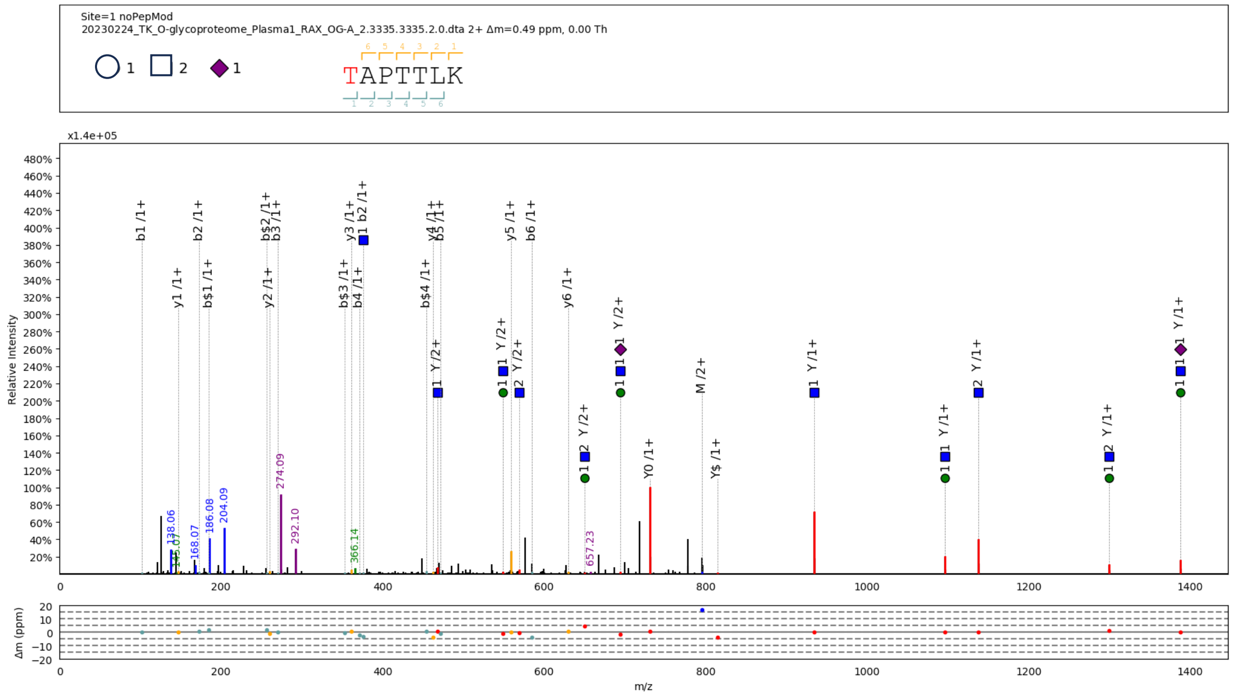

PRG4–T712, H (1) N (1) A (2)

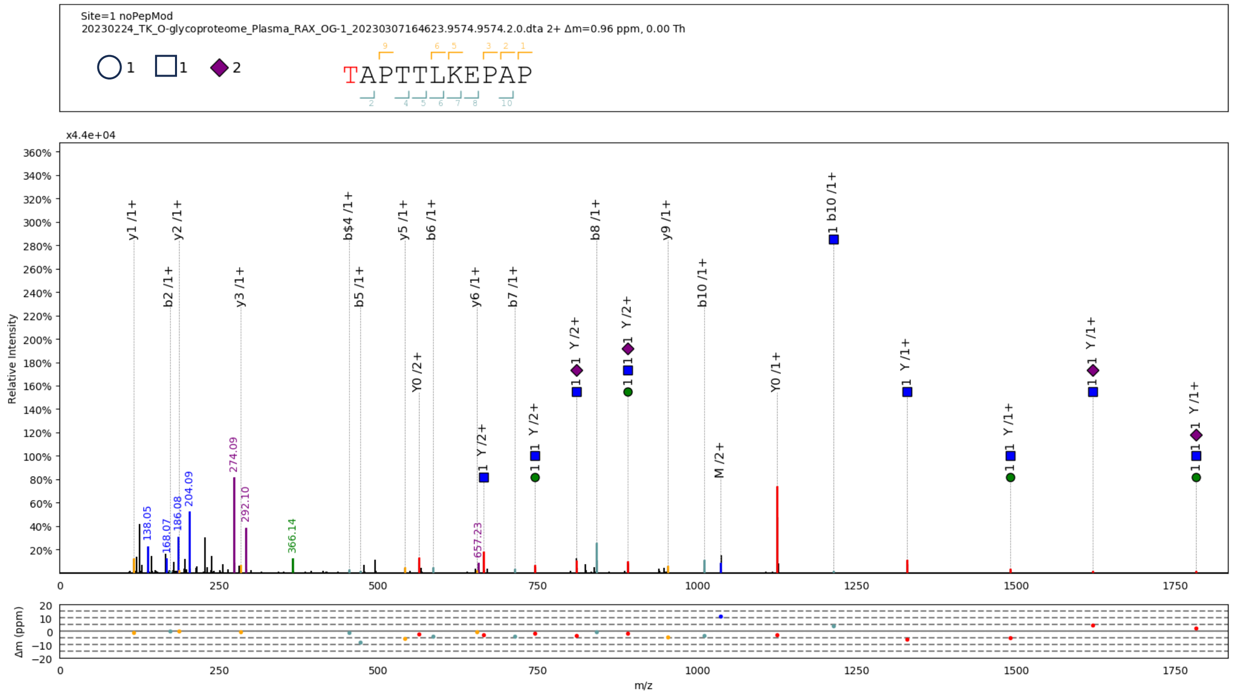

PRG4–T723-T792, H (1) N (1) A (1)

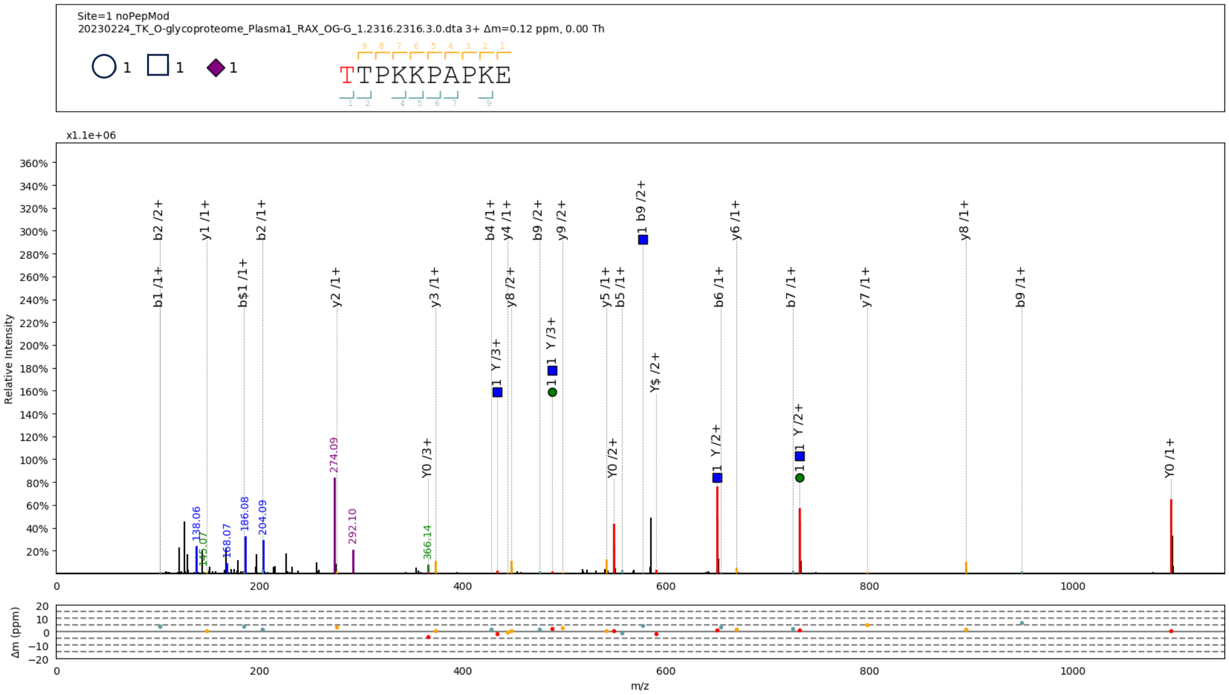

PRG4–T723-T792, H (2) N (2) A (2)

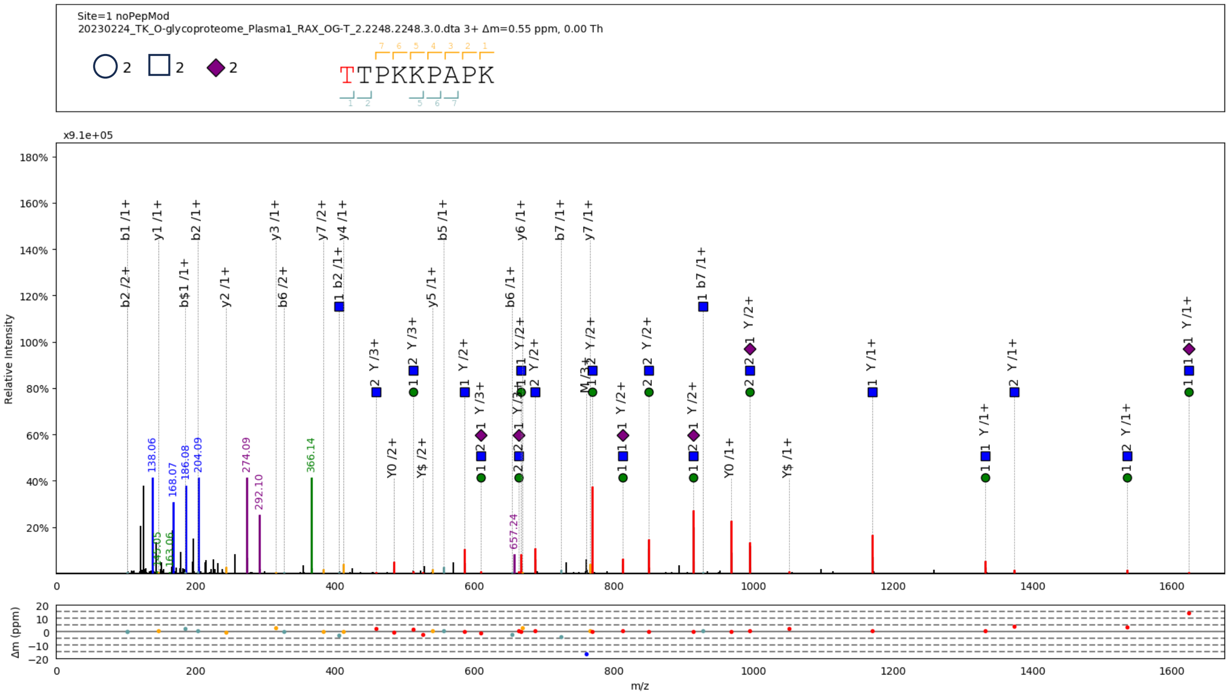

PRG4–T723-T792, H (2) N (2) A (1)

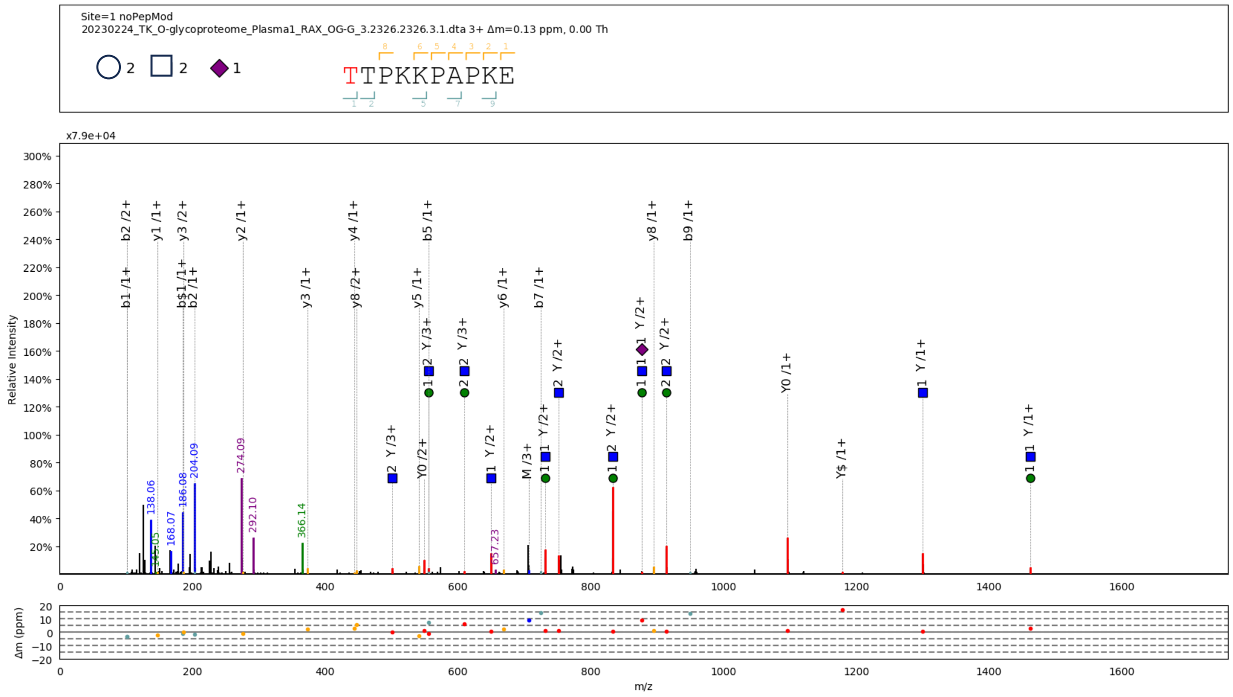

PRG4–T723-T792, H (1) N (1)

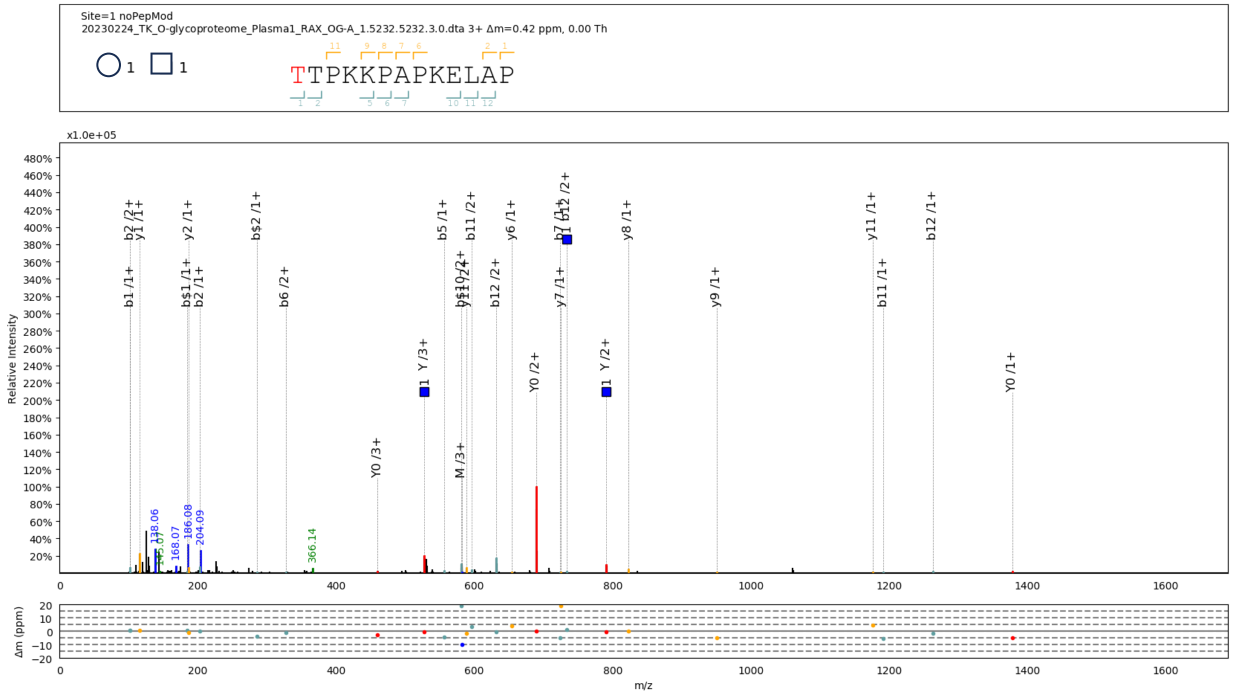

PRG4–T744-T813, H (2) N (2) A (2)

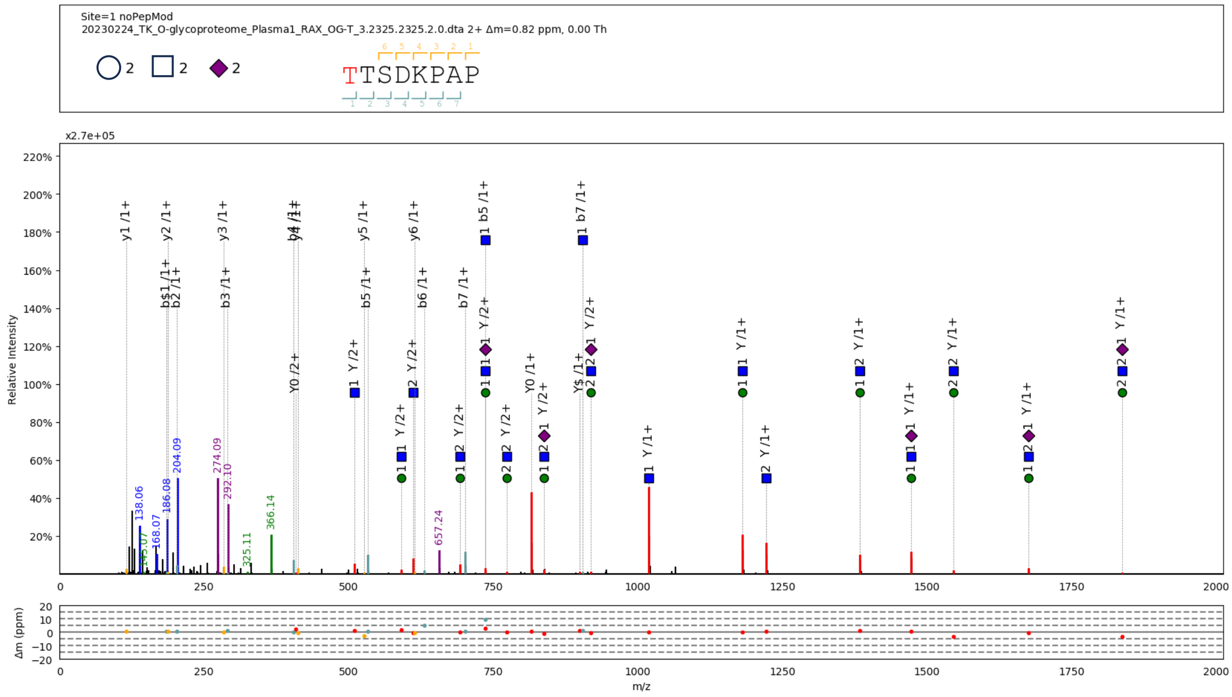

PRG4–T744-T813, H (2) N (2) A (1)

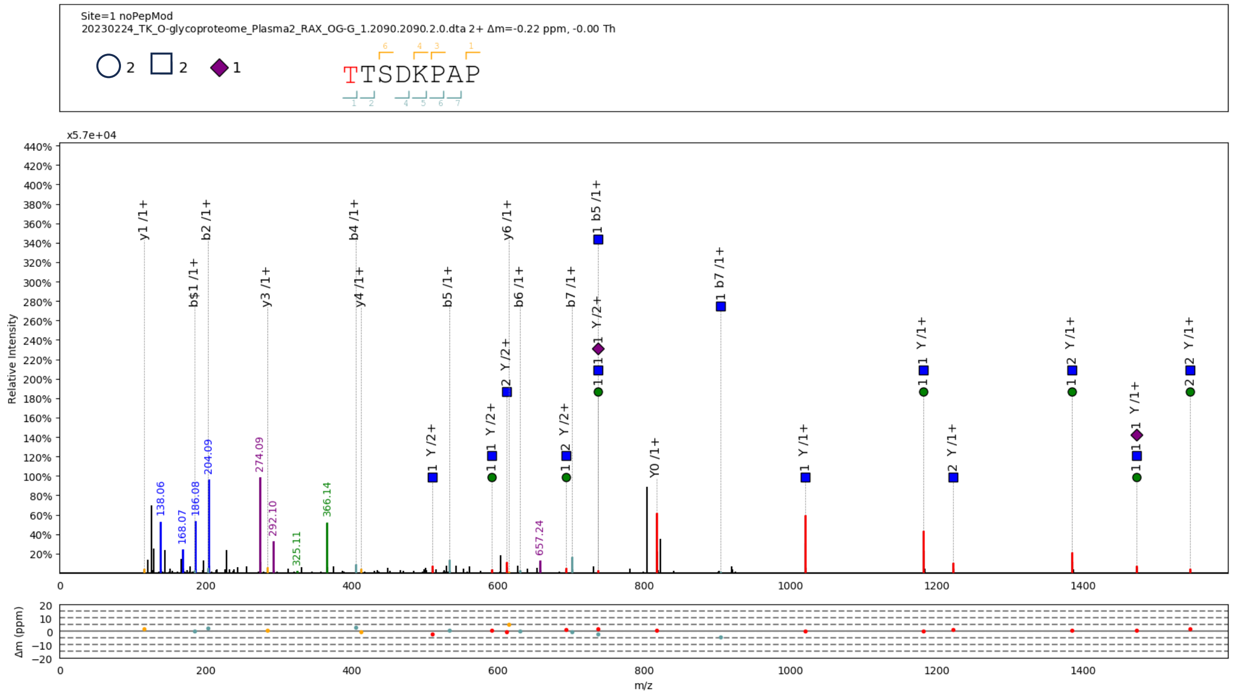

PRG4–T757-T826, H (2) N (2) A (2)

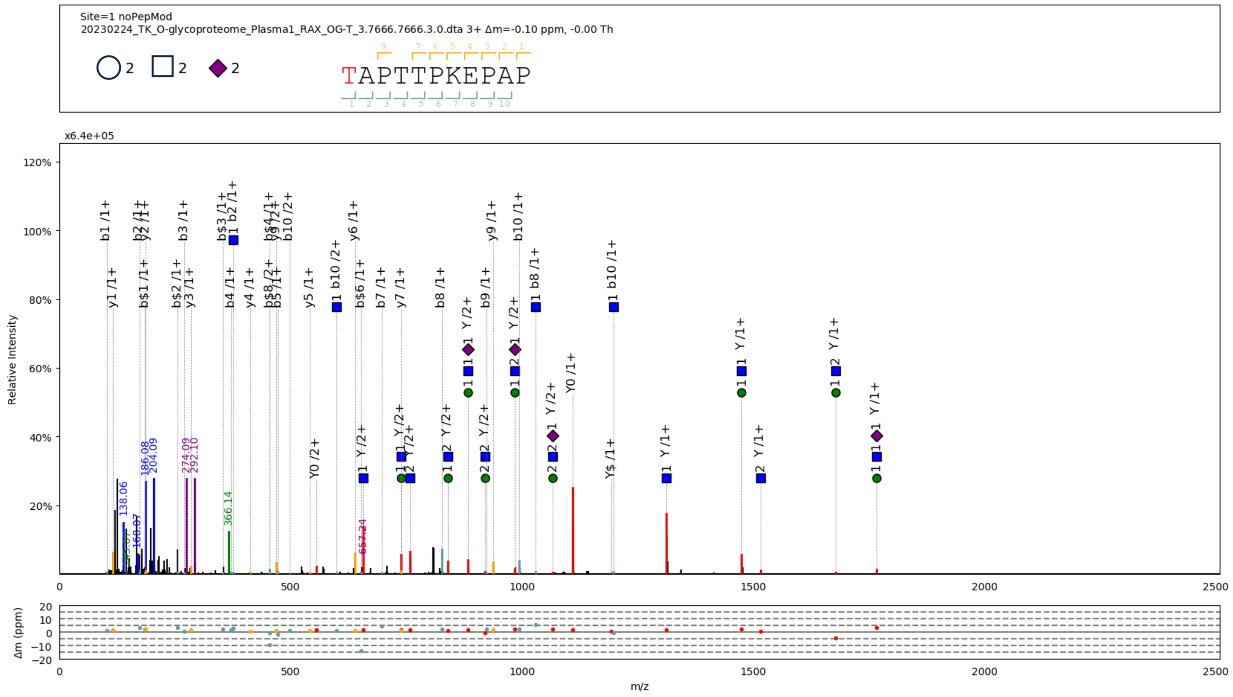

PRG4–T757-T826, H (3) N (3) A (3)

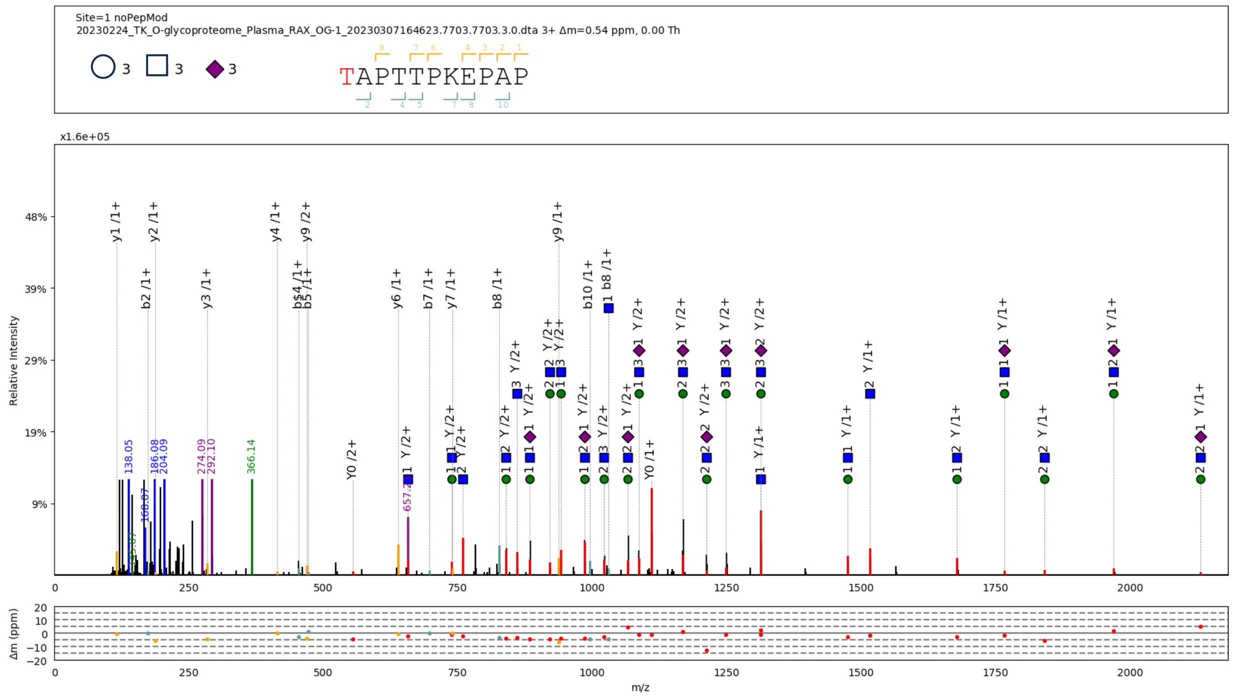

PRG4‒T757-T826, H (2) N (2) A (1)

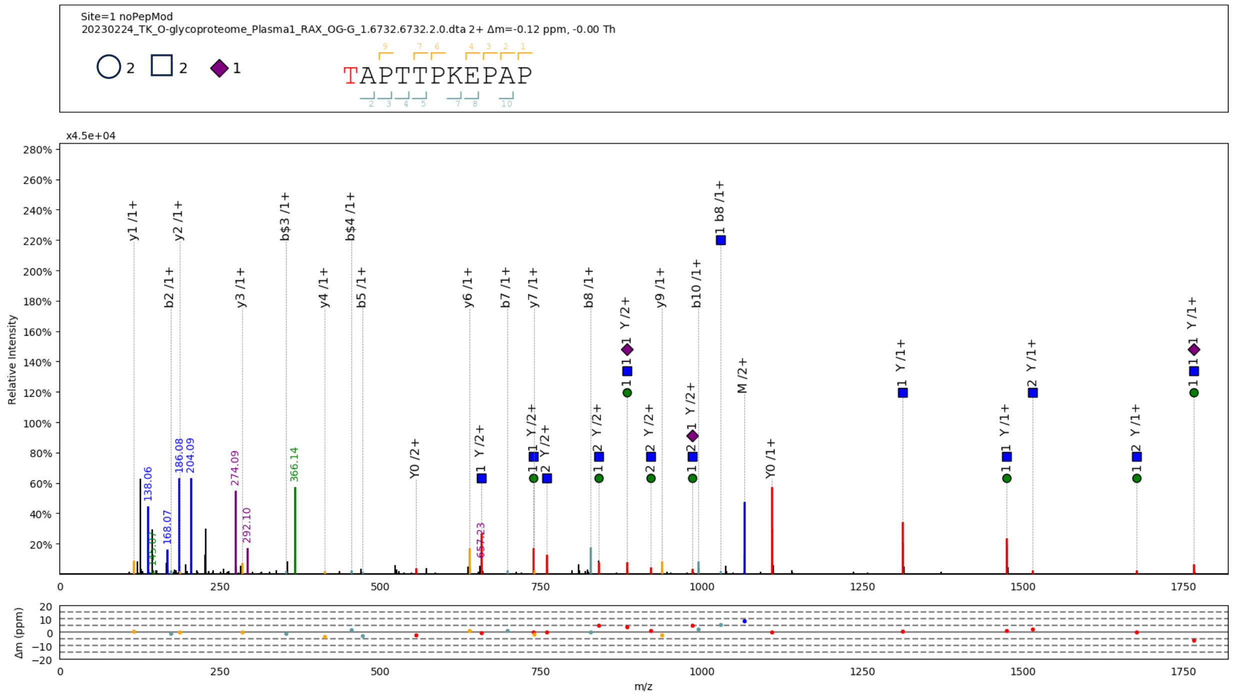

PRG4‒T757-T826, H (1) N (2) A (1)

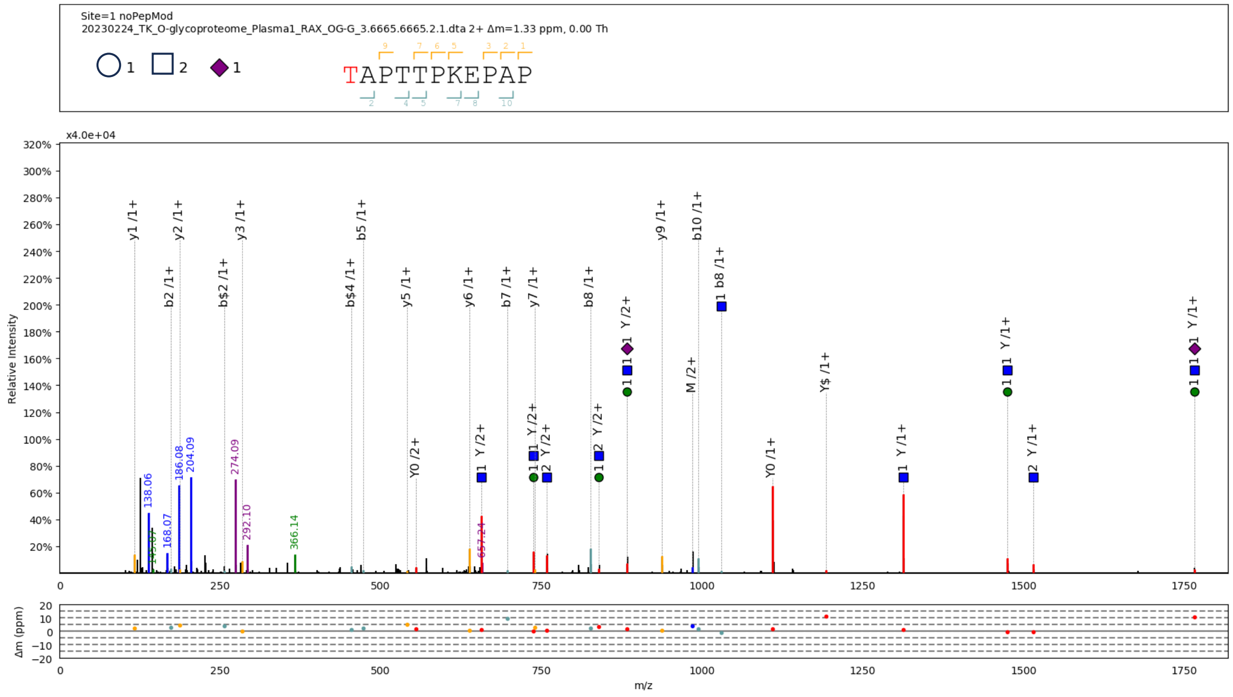

PRG4−T868, H (1) N (1)

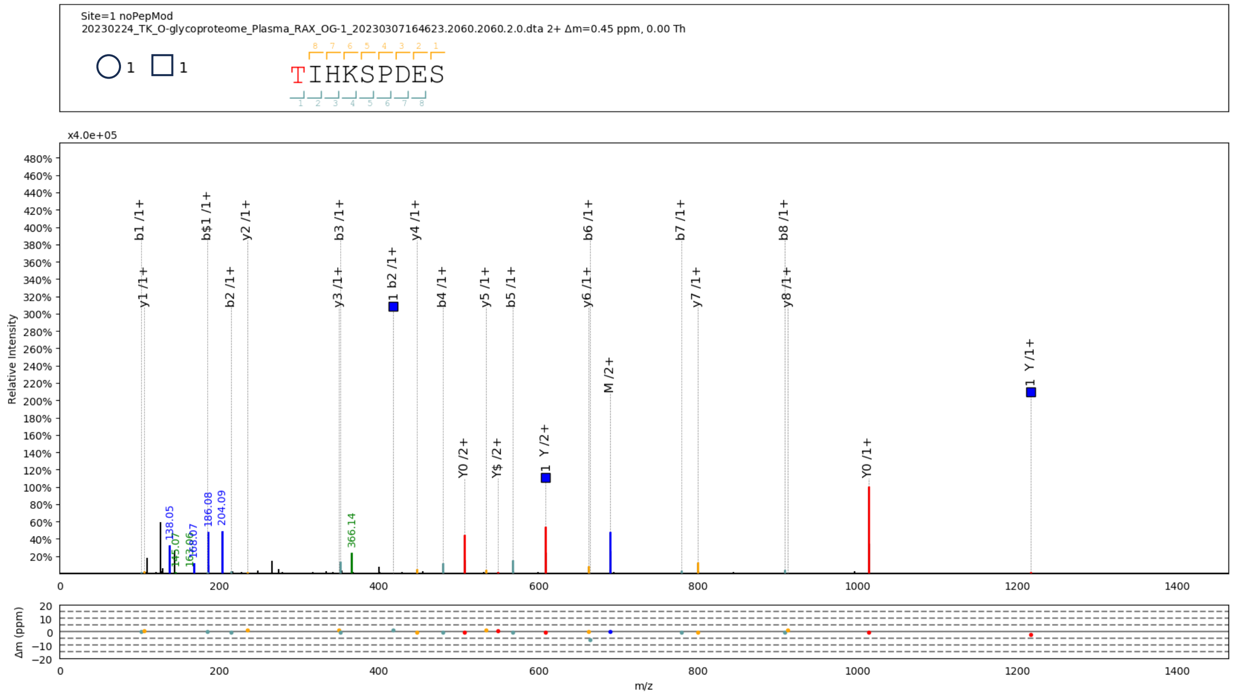

PRG4−T868, H (1) N (1) A (1)

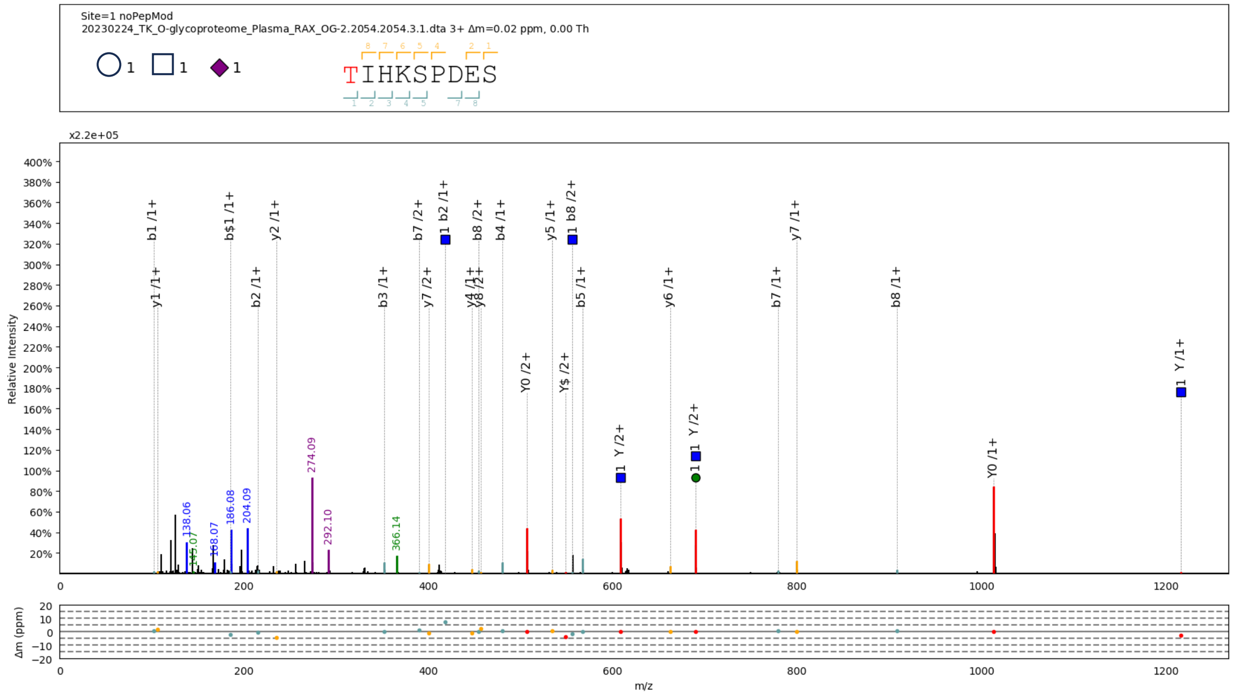

PRG4−T868, N (1)

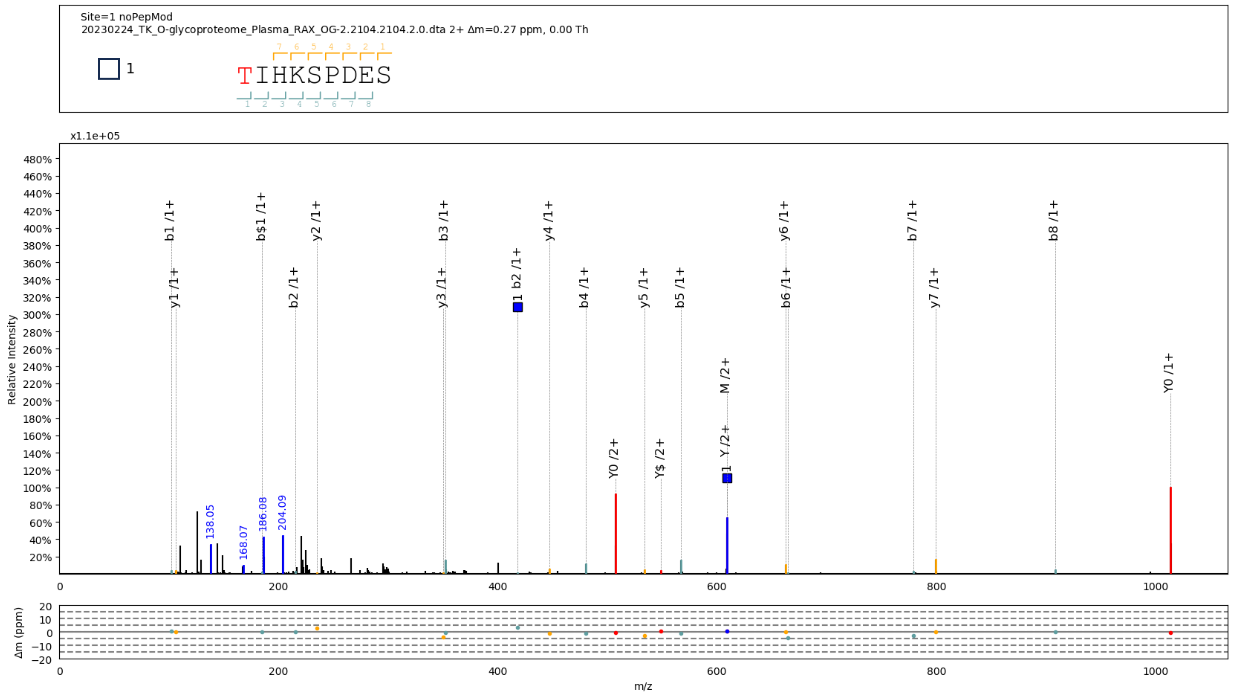

PRG4−T885, H (1) N (1) A (1)

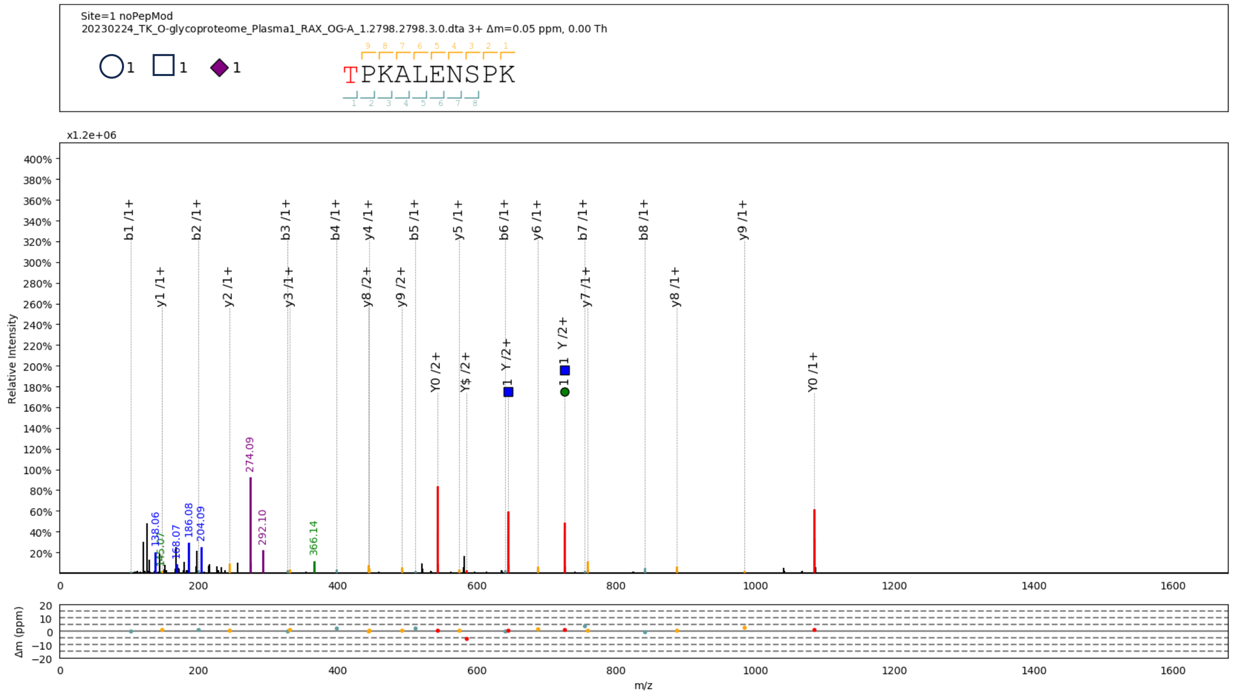

PRG4−T885, H (1) N (1) A (2)

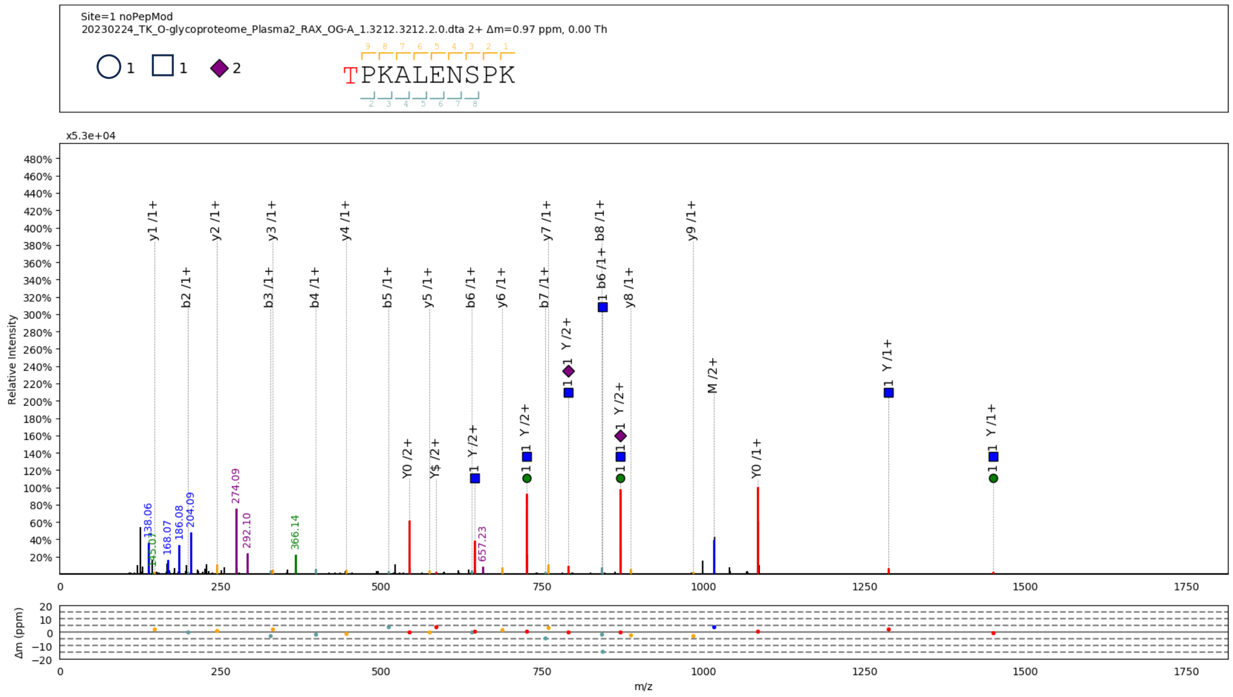

PRG4−T885, H (1) N (1)

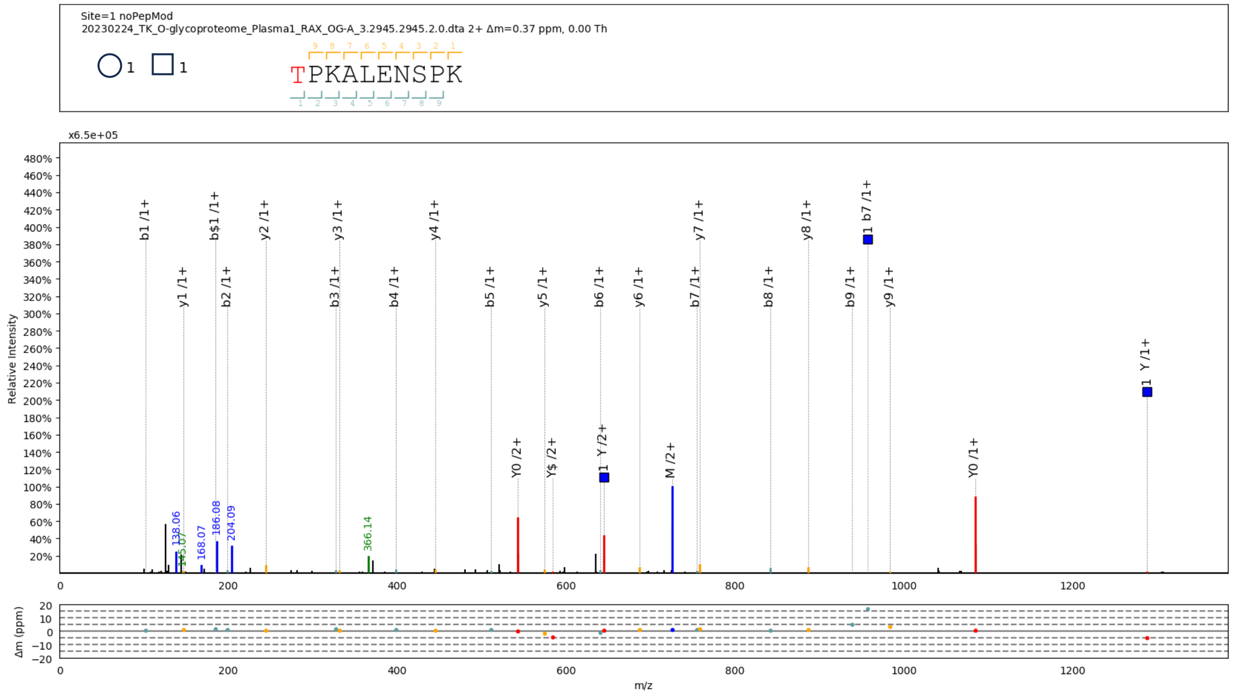

PRG4–T885, N (1)

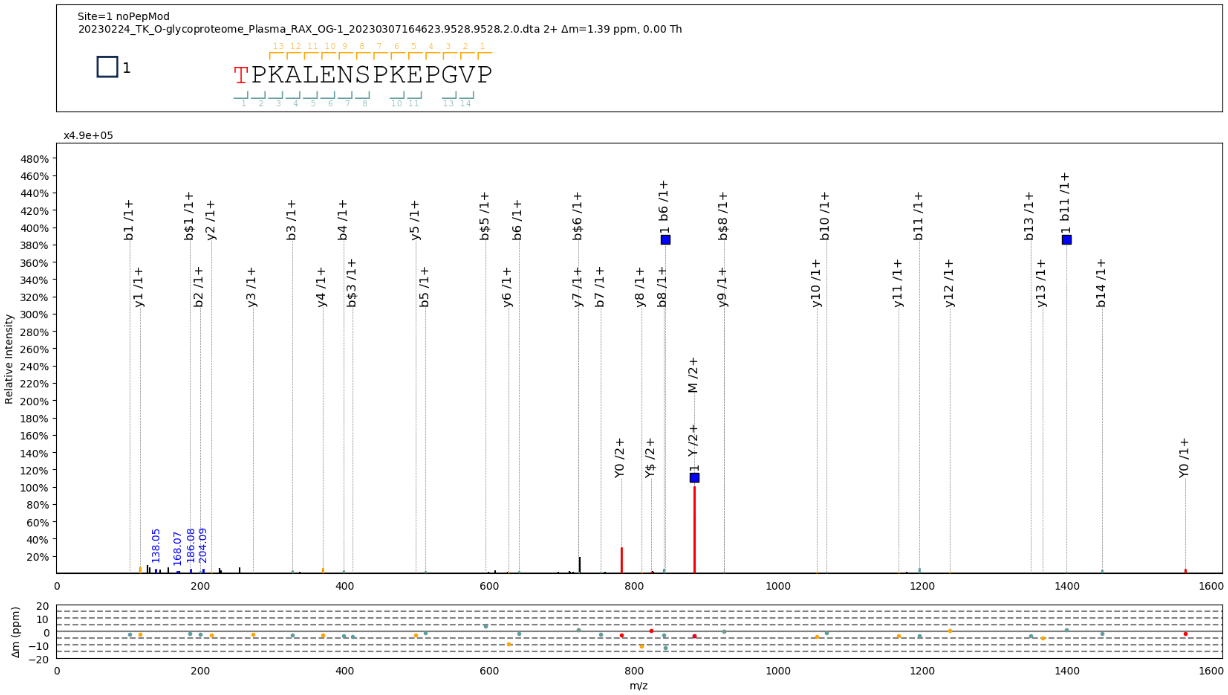

PRG4–T900, H (1) N (1) A (1)

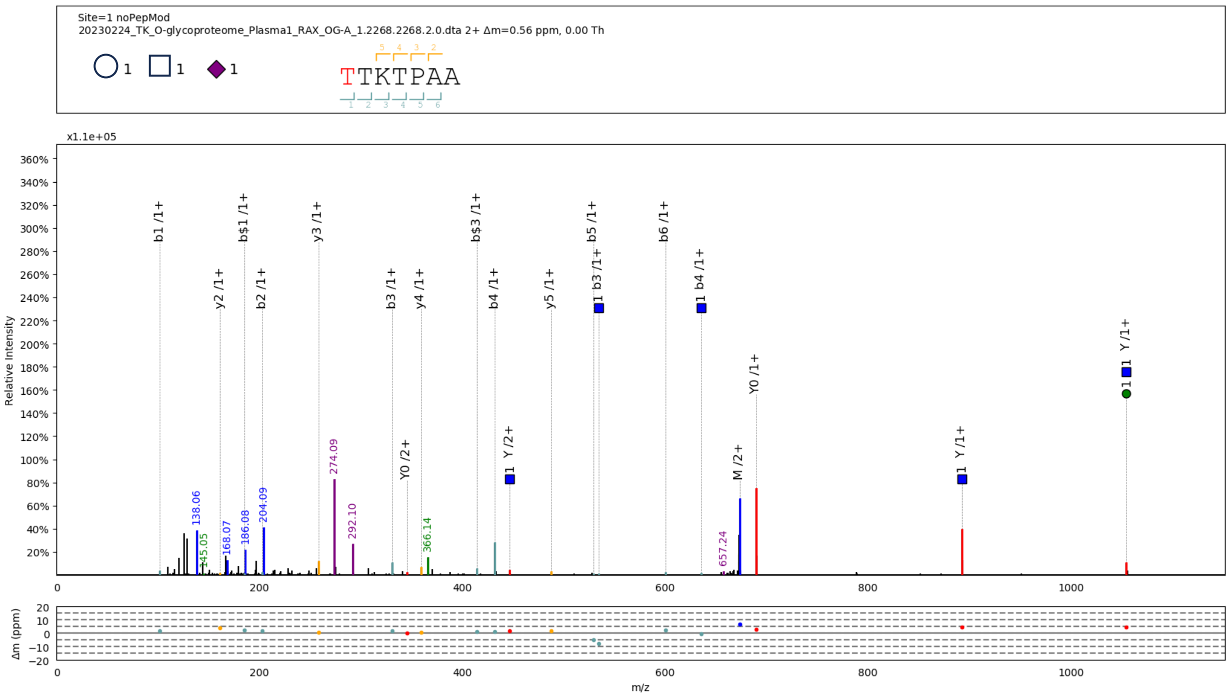

PRG4–T930, H (1) N (1) A (1)

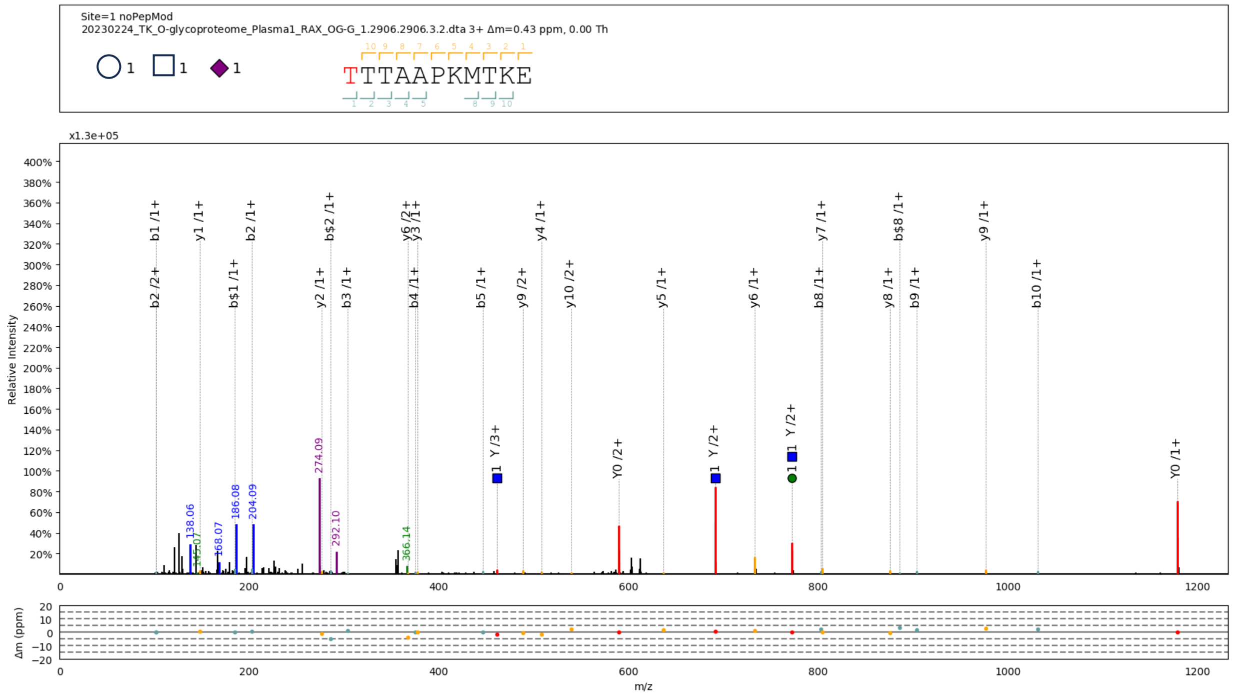

PRG4–T991, H (2) N (2) A (2)

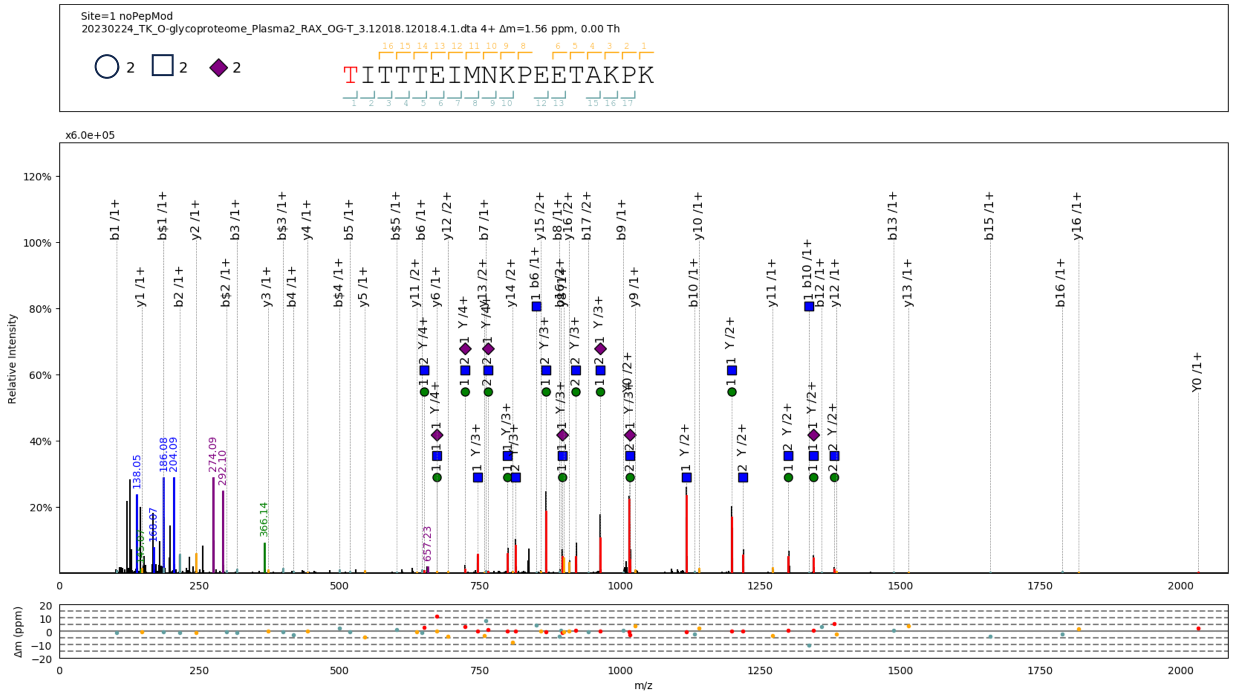

PRG4–T991, H (1) N (1) A (1)

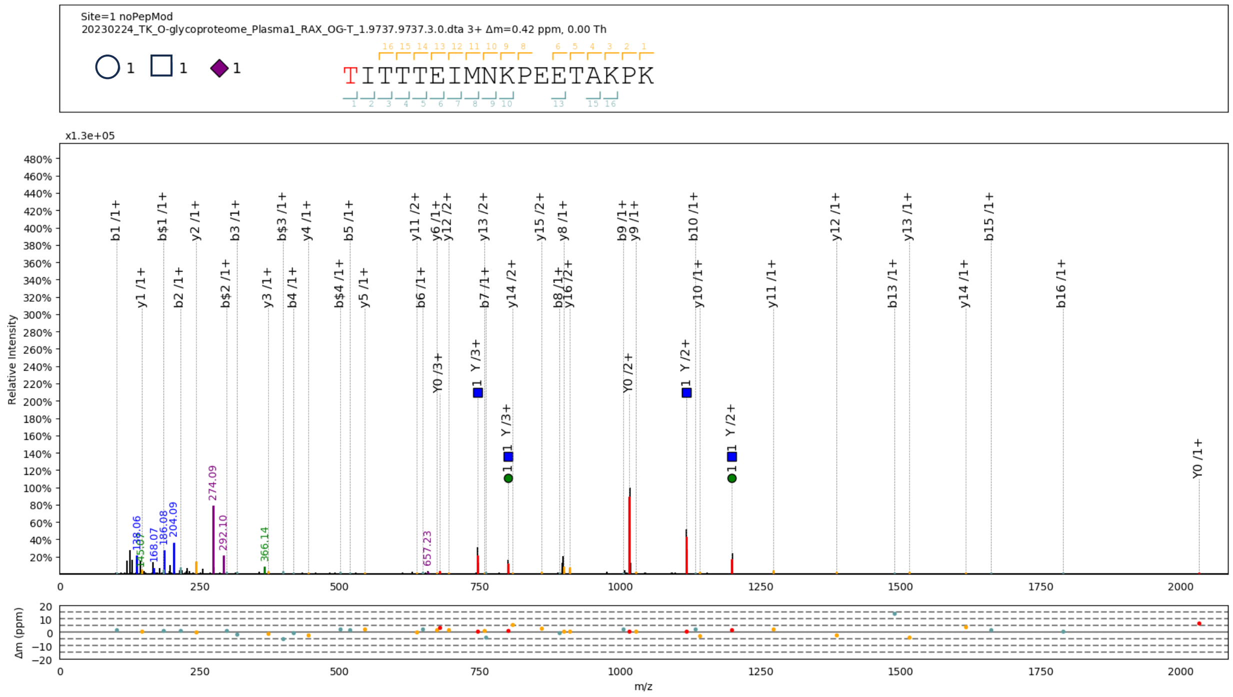

PRG4–T991, H (2) N (2) A (1)

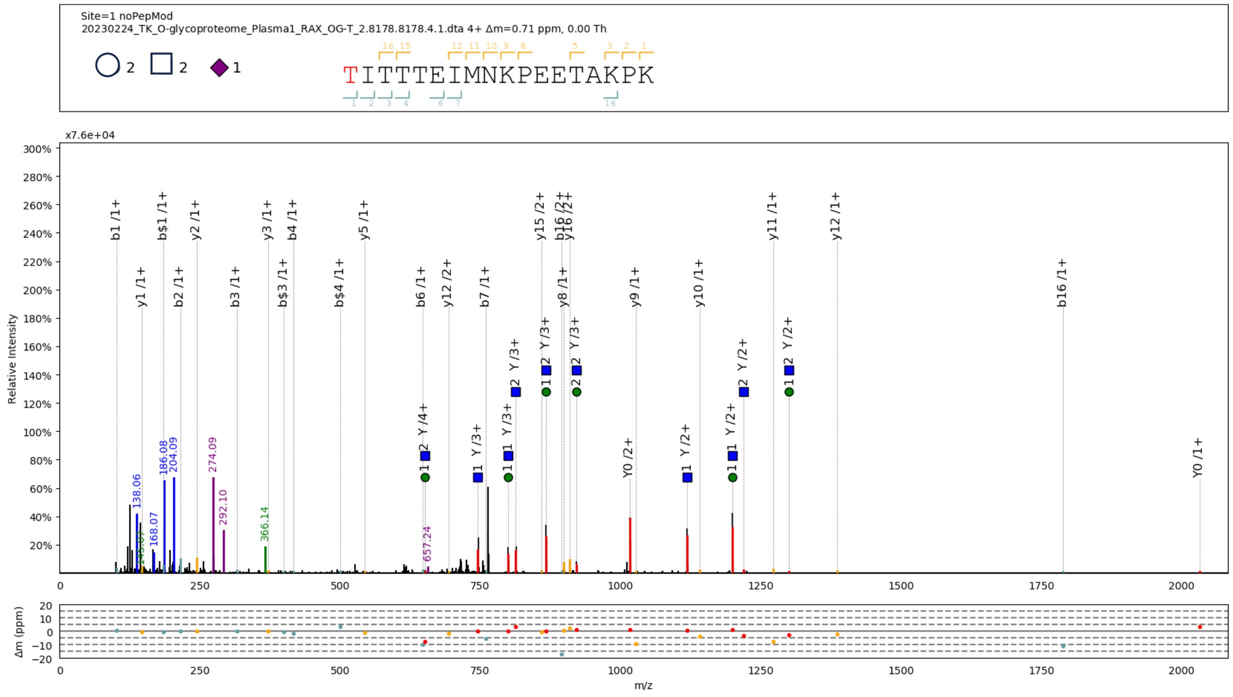

PRG4–T1025, H (1) N (1) A (1)

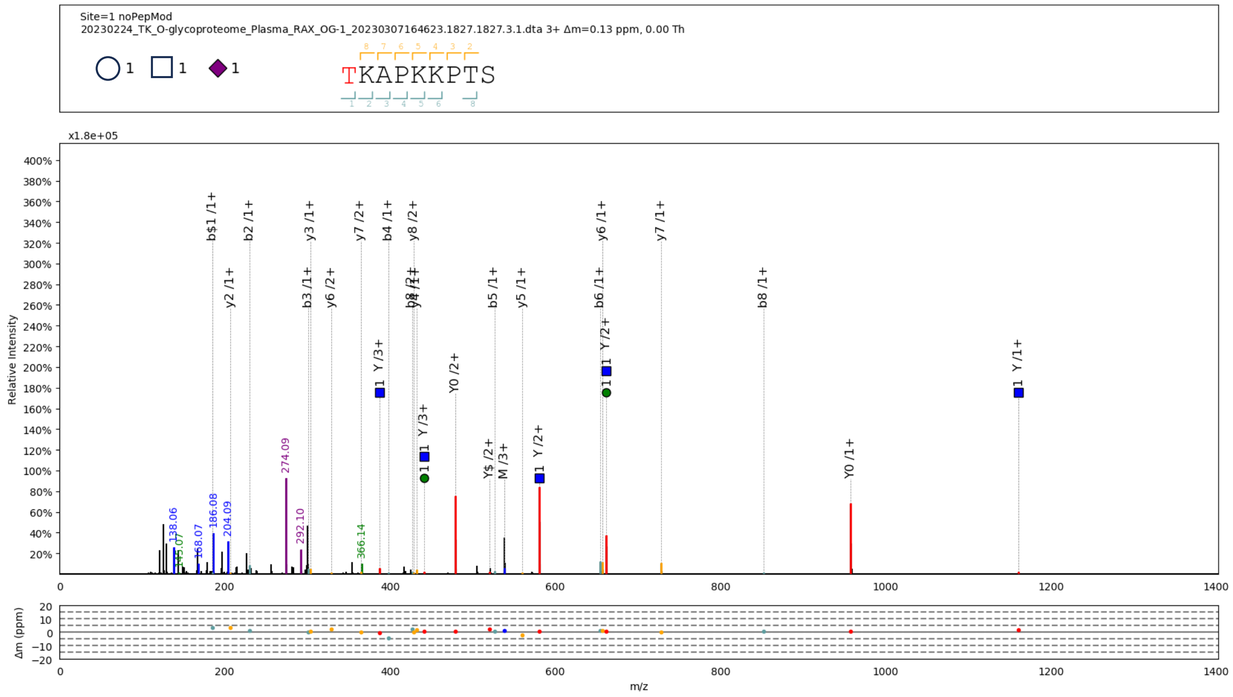

PRG4–T1082, H (1) N (1) A (1)

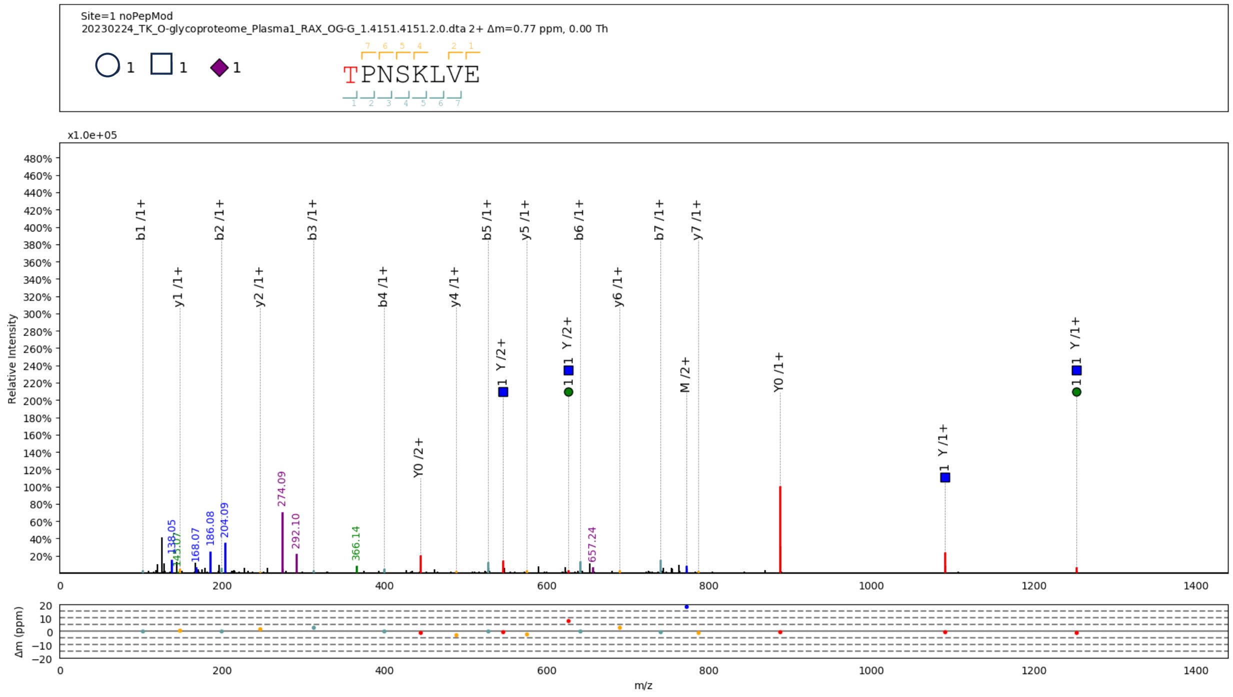

Data S2. MS/MS spectra derived from five proteins purified from human plasma (kininogen-1, fetuin-A, fibrinogen, apolipoprotein E and plasminogen), related to Figure 5.

(A) Fetuin-A (AHSG)

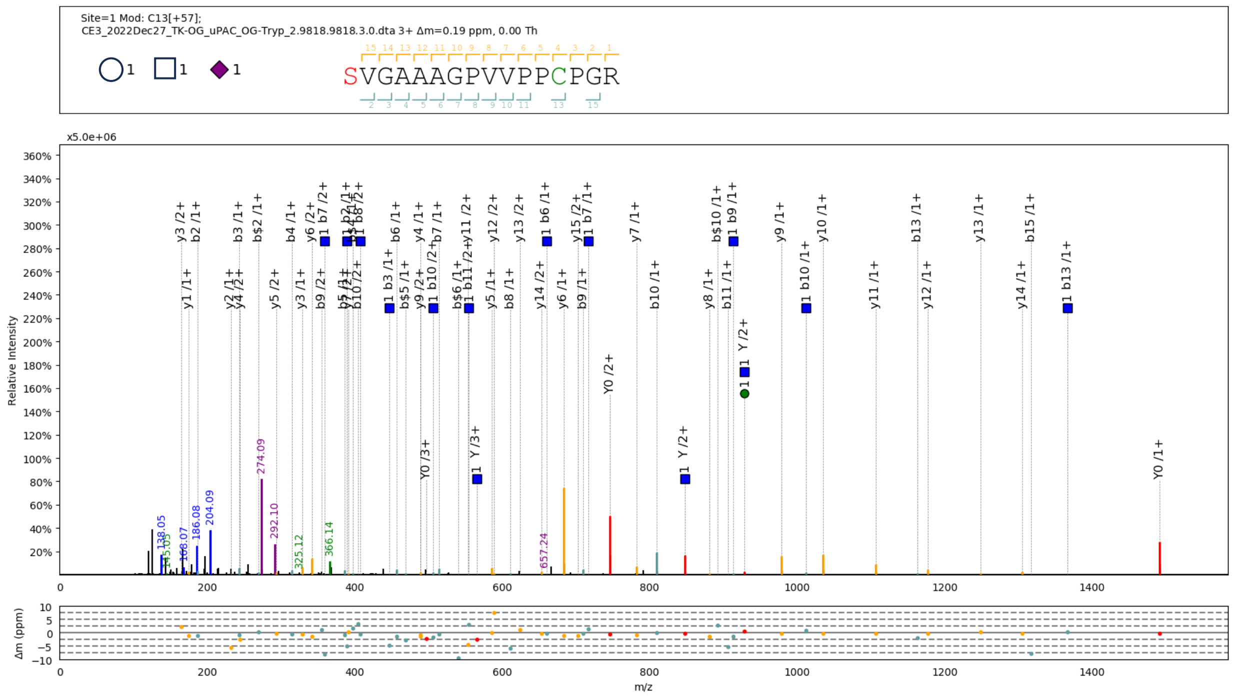

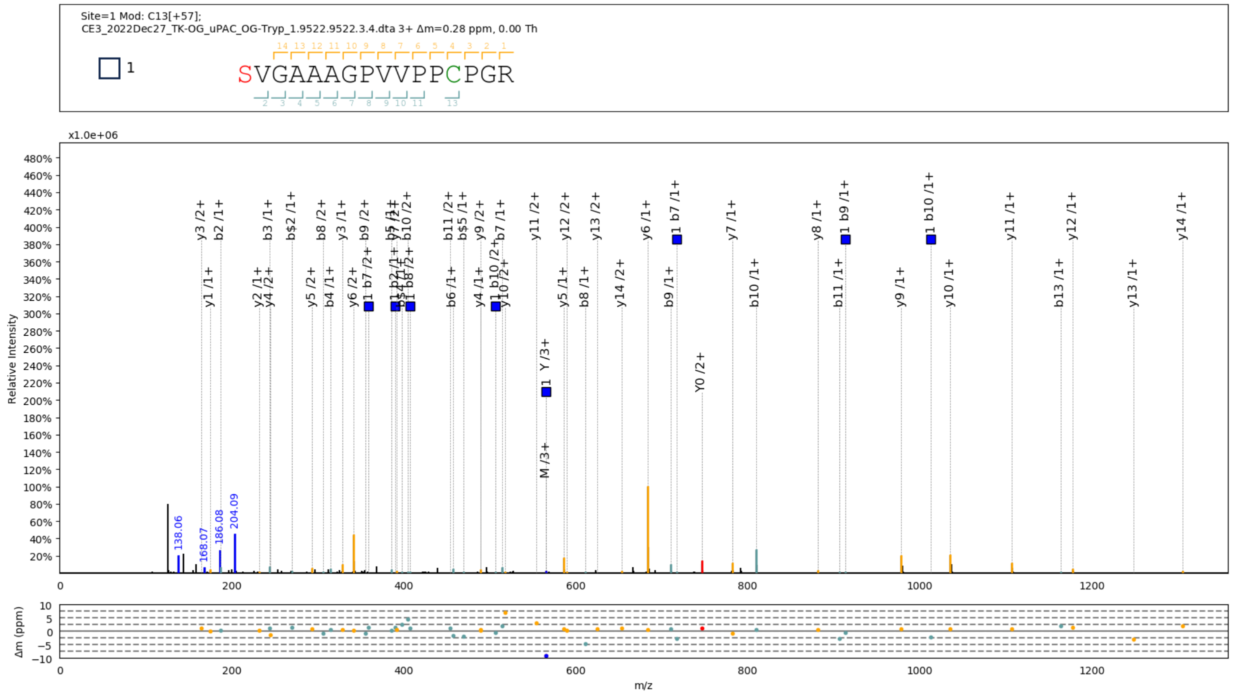

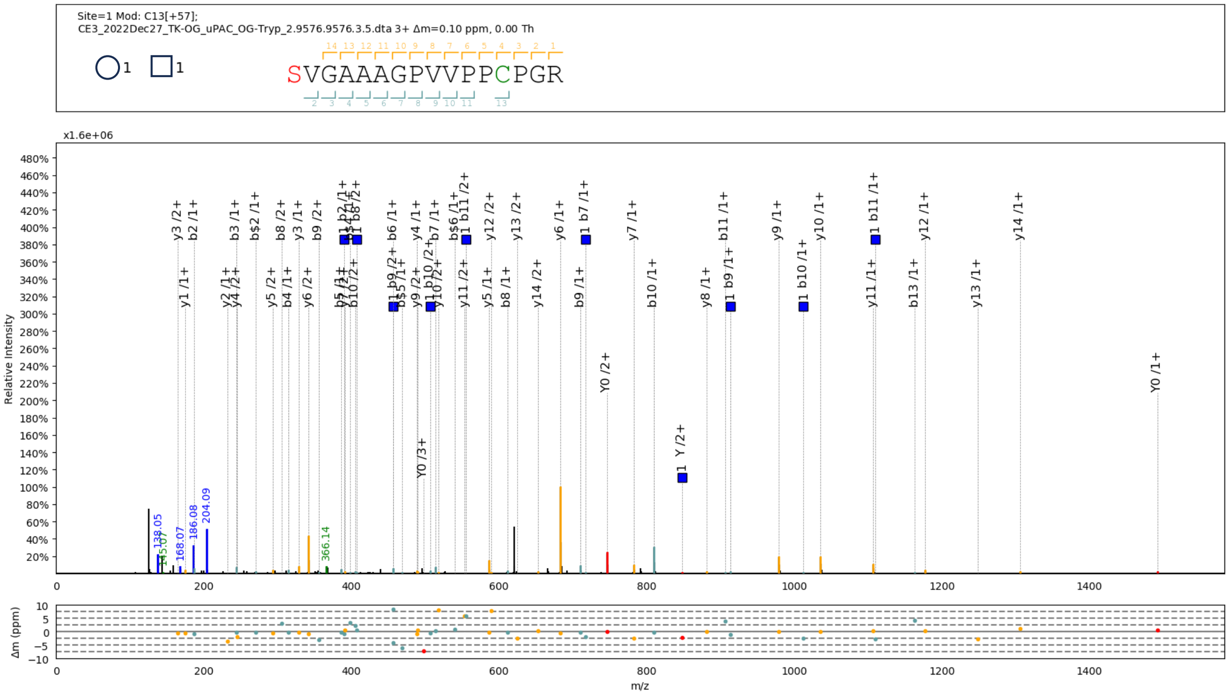
Site=1 Mod: C13[+57];
CE3_2022Dec27_TK-OG_uPAC_OG-Tryp_2.9576.9576.3.5.dta 3+ Δm=0.10 ppm, 0.00 Th
SVGAAAGPVVPPCPGR
x1.6e+06
Relative Intensity
Δm (ppm)
m/z

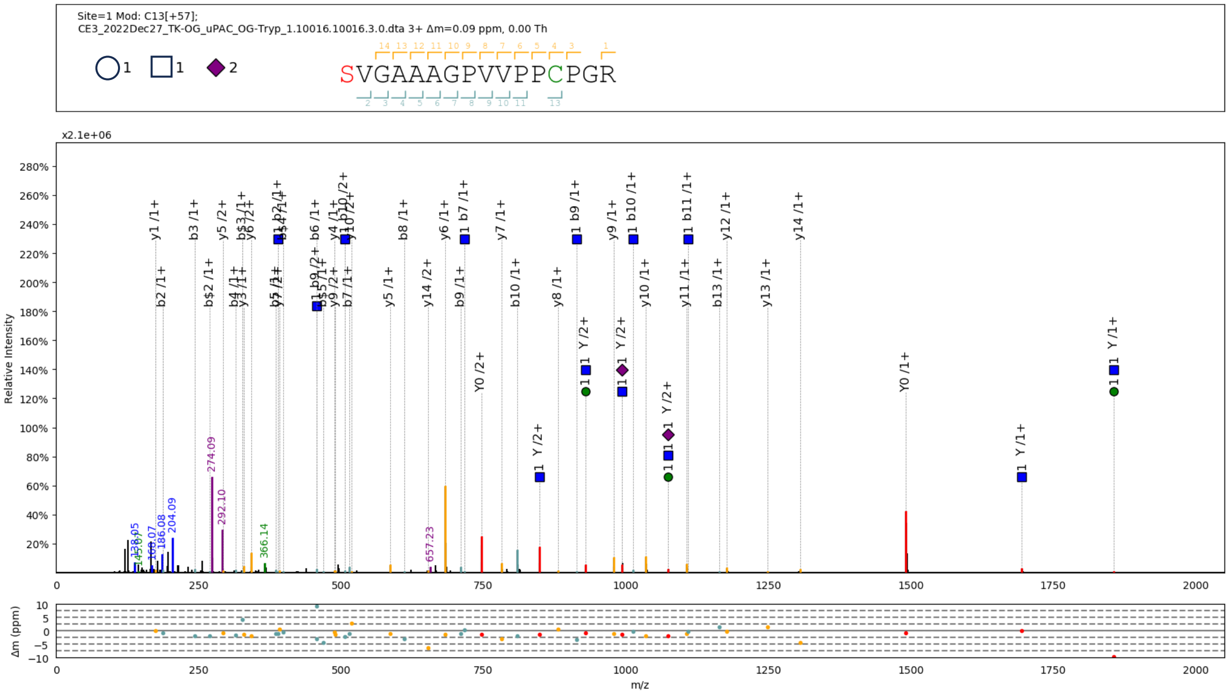
Site=1 Mod: C13[+57];
CE3_2022Dec27_TK-OG_uPAC_OG-Tryp_1.10016.10016.3.0.dta 3+ Δm=0.09 ppm, 0.00 Th
SVGAAAGPVVPPCPGR
x2.1e+06
Relative Intensity
Δm (ppm)
m/z

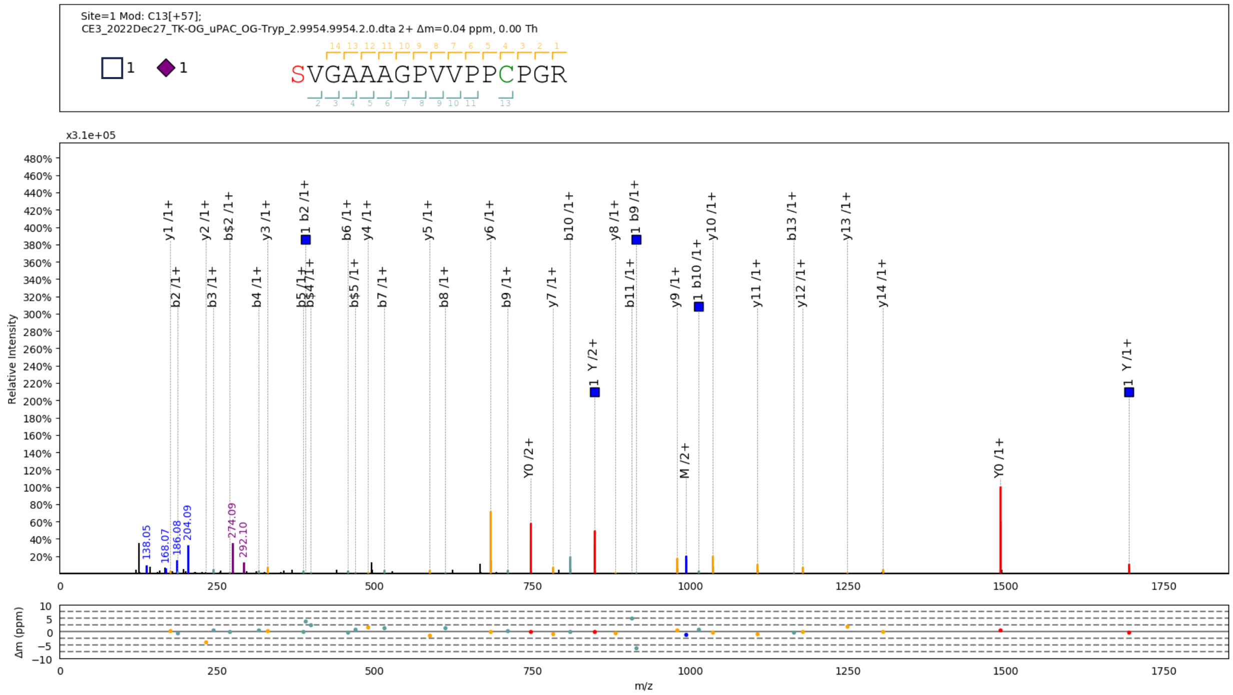

Site=1 Mod: C13[+57];
CE3_2022Dec27_TK-OG_uPAC_OG-Tryp_2.9954.9954.2.0.dta 2+ Δm=0.04 ppm, 0.00 Th
1
1
SVGAAAGPVVPPCPGR
x3.1e+05
y1 /1+
y2 /1+
b$2 /1+
y3 /1+
1 b2 /1+
b6 /1+
y4 /1+
y5 /1+
y6 /1+
b10 /1+
y8 /1+
1 b9 /1+
y10 /1+
b13 /1+
y13 /1+
b2 /1+
b3 /1+
b4 /1+
b5 /1+
b$4 /1+
b$5 /1+
b7 /1+
b8 /1+
b9 /1+
y7 /1+
b11 /1+
y9 /1+
1 b10 /1+
y11 /1+
y12 /1+
y14 /1+
1 Y /2+
1 Y /1+
Y0 /2+
M /2+
Y0 /1+
138.05
168.07
186.08
204.09
274.09
292.10
Relative Intensity
Δm (ppm)
m/z

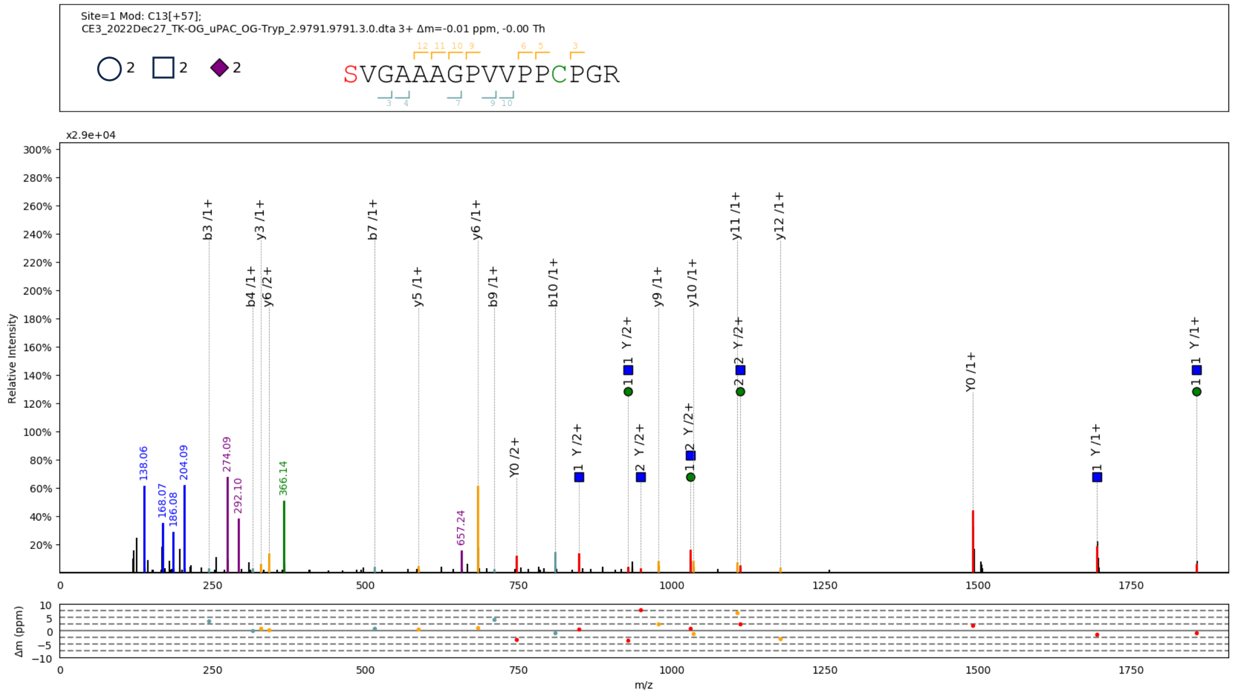

Site=1 Mod: C13[+57];
CE3_2022Dec27_TK-OG_uPAC_OG-Tryp_2.9791.9791.3.0.dta 3+ Δm=-0.01 ppm, -0.00 Th
2
2
2
SVGAAAGPVVPPCPGR
x2.9e+04
b3 /1+
y3 /1+
b7 /1+
y6 /1+
y11 /1+
y12 /1+
b4 /1+
y6 /2+
y5 /1+
b9 /1+
b10 /1+
y9 /1+
y10 /1+
1 1 Y /2+
2 2 Y /2+
1 Y /1+
Y0 /1+
1 2 Y /2+
Y0 /2+
1 Y /2+
2 Y /2+
138.06
168.07
186.08
204.09
274.09
292.10
366.14
657.24
Relative Intensity
Δm (ppm)
m/z

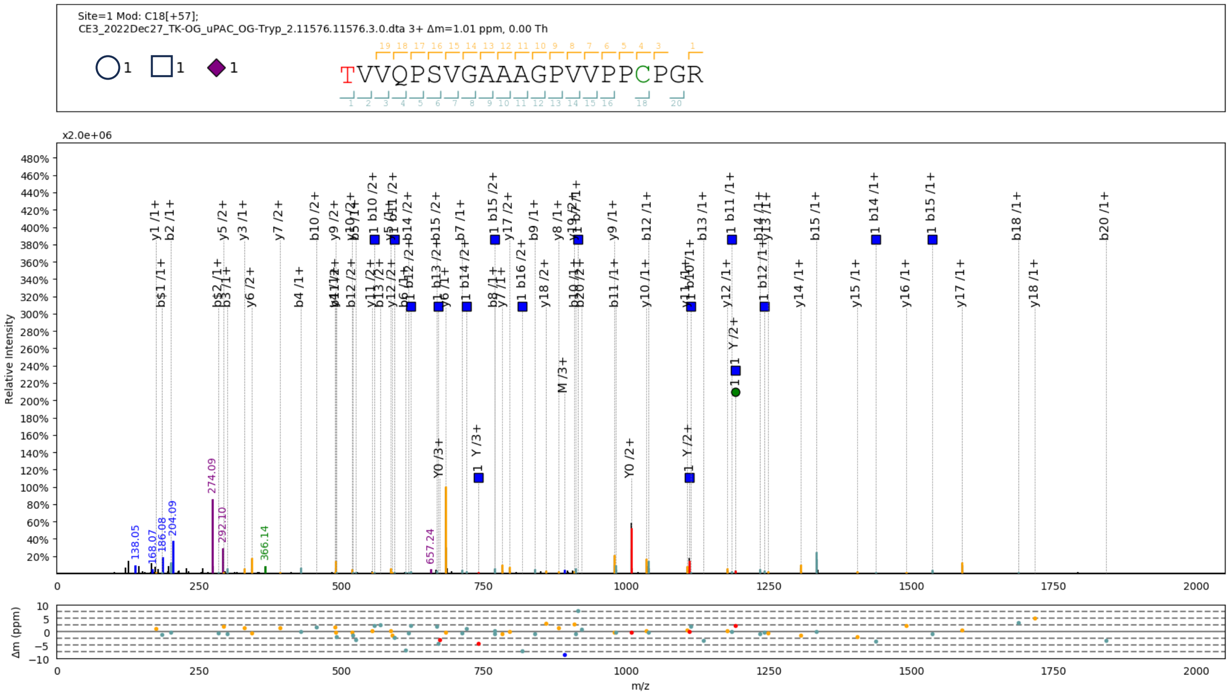
Site=1 Mod: C18[+57];
CE3_2022Dec27_TK-OG_uPAC_OG-Tryp_2.11576.11576.3.0.dta 3+ Δm=1.01 ppm, 0.00 Th
TVVQPSVGAAAGPVVPPCPGR
x2.0e+06
Relative Intensity
Δm (ppm)
m/z

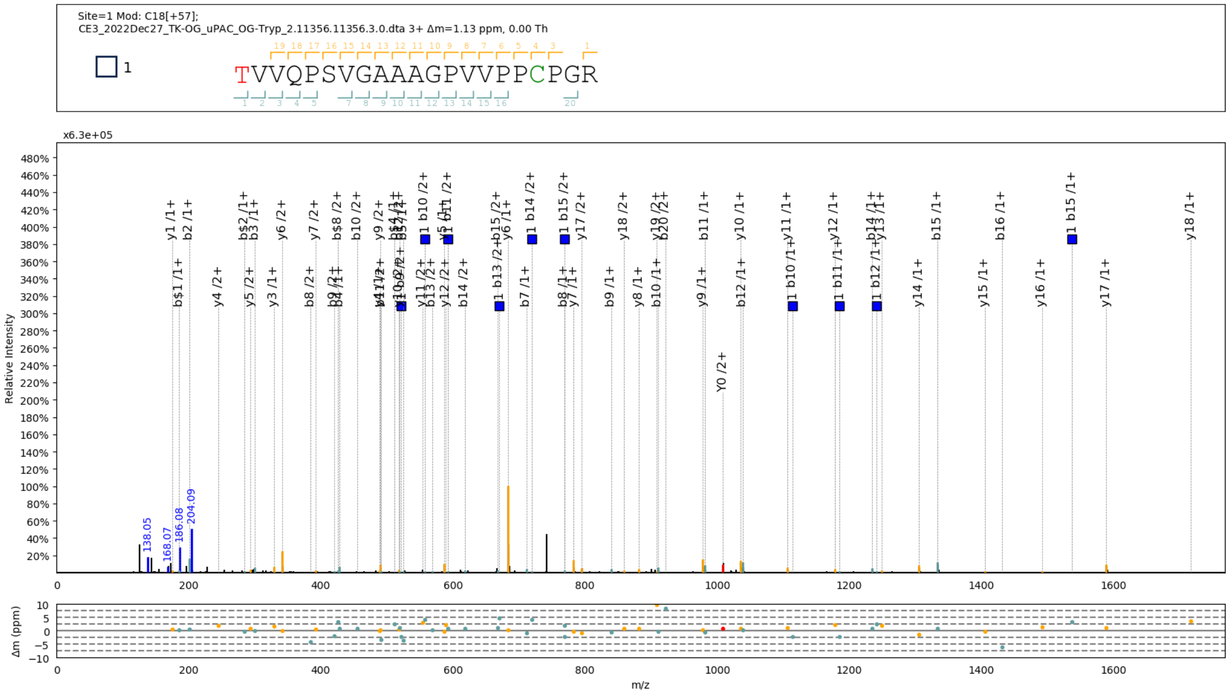
Site=1 Mod: C18[+57];
CE3_2022Dec27_TK-OG_uPAC_OG-Tryp_2.11356.11356.3.0.dta 3+ Δm=1.13 ppm, 0.00 Th
TVVQPSVGAAAGPVVPPCPGR
x6.3e+05
Relative Intensity
Δm (ppm)
m/z

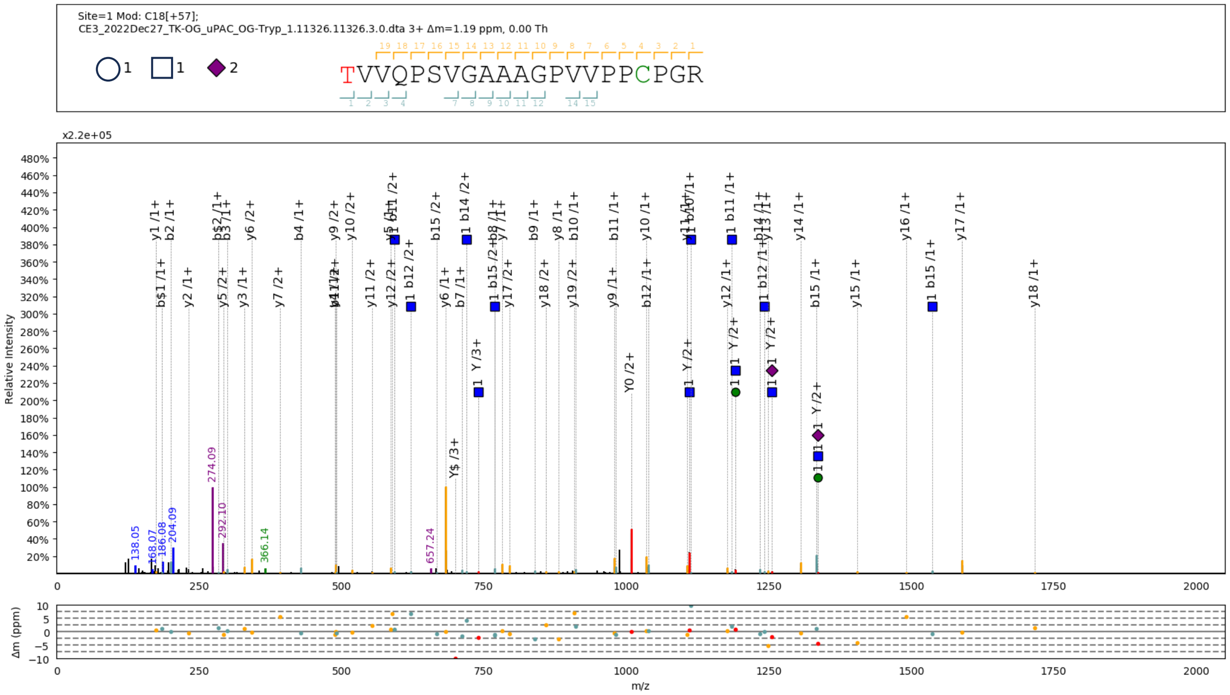
Site=1 Mod: C18[+57];
CE3_2022Dec27_TK-OG_uPAC_OG-Tryp_1.11326.11326.3.0.dta 3+ Δm=1.19 ppm, 0.00 Th
TVVQPSVGAAAGPVVPPCPGR
x2.2e+05
Relative Intensity
Δm (ppm)
m/z

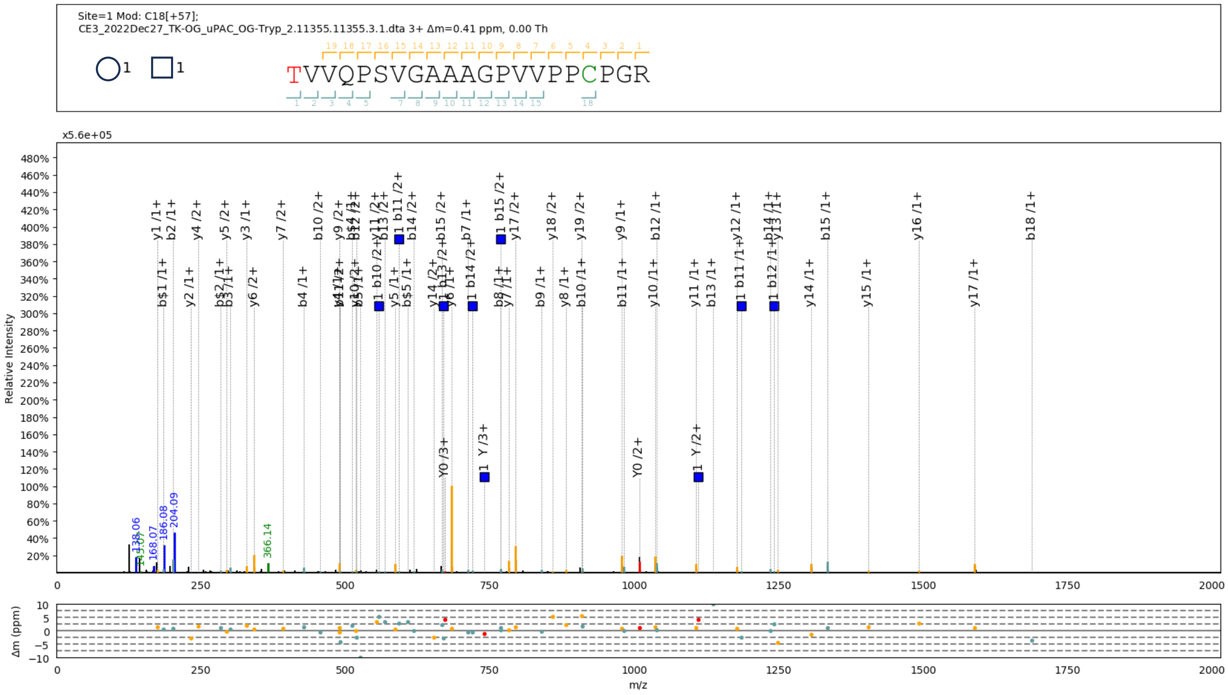
Site=1 Mod: C18[+57];
CE3_2022Dec27_TK-OG_uPAC_OG-Tryp_2.11355.11355.3.1.dta 3+ Δm=0.41 ppm, 0.00 Th
TVVQPSVGAAAGPVVPPCPGR
x5.6e+05
Relative Intensity
Δm (ppm)
m/z

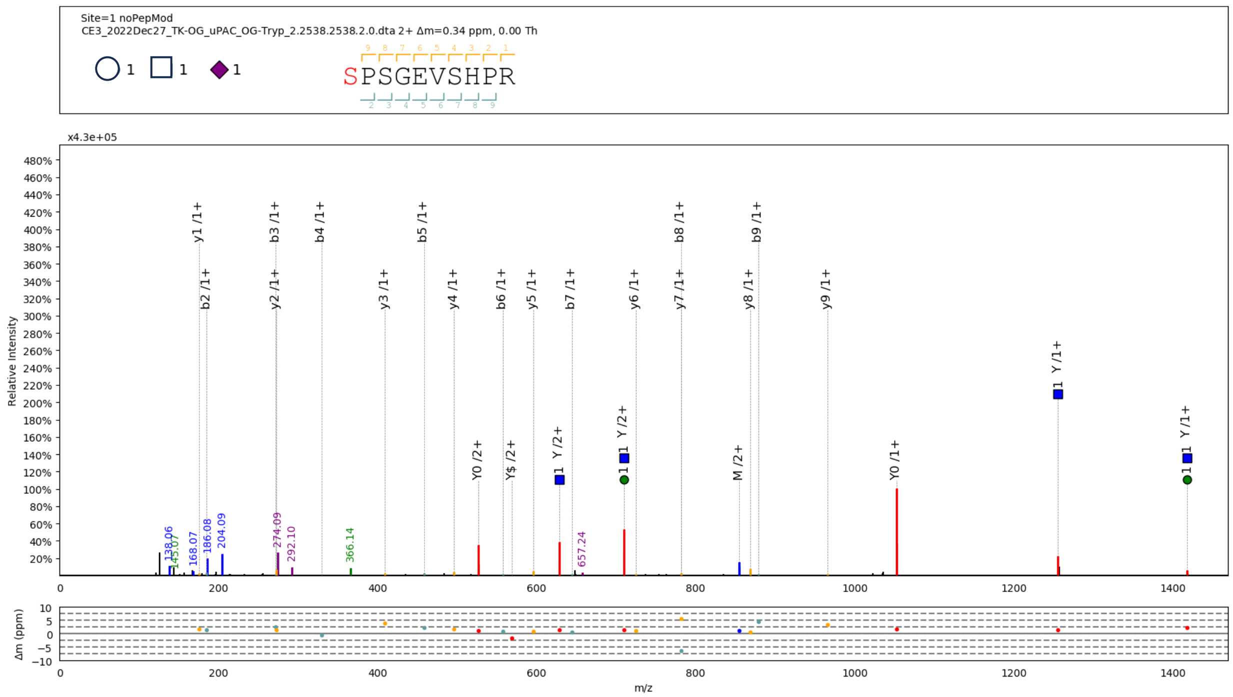
Site=1 noPepMod
CE3_2022Dec27_TK-OG_uPAC_OG-Tryp_2.2538.2538.2.0.dta 2+ Δm=0.34 ppm, 0.00 Th
SPSGEVSHPR
Relative Intensity
Δm (ppm)
m/z

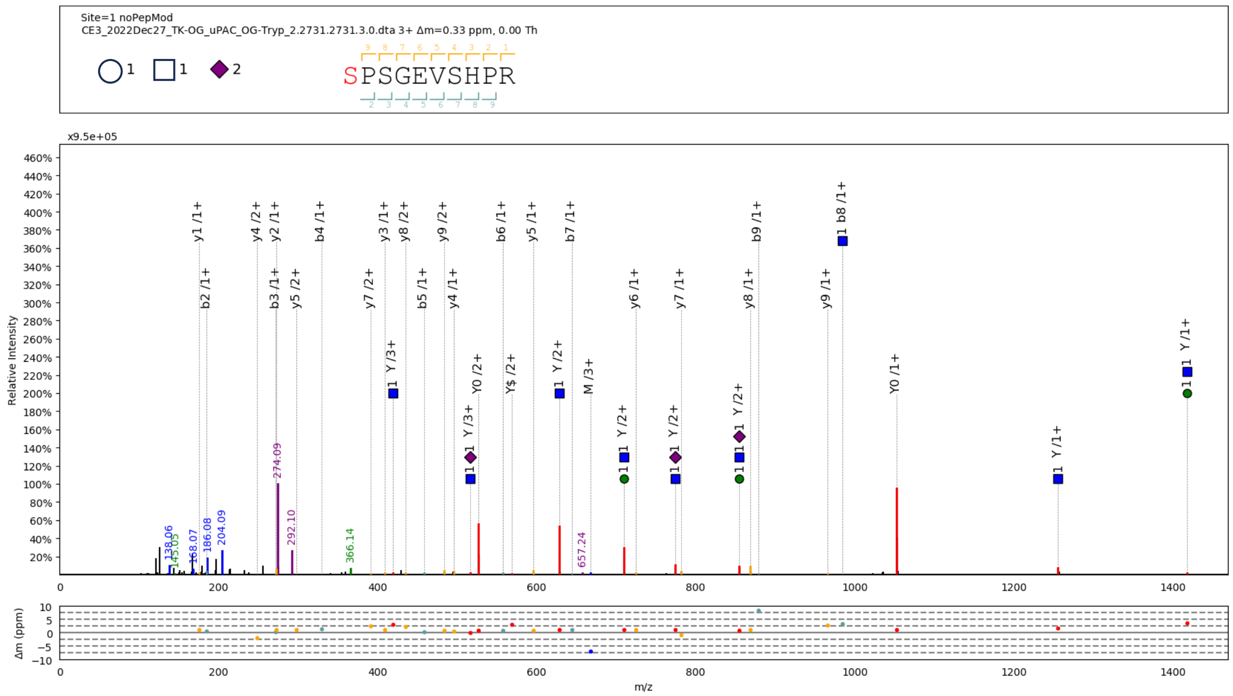
Site=1 noPepMod
CE3_2022Dec27_TK-OG_uPAC_OG-Tryp_2.2731.2731.3.0.dta 3+ Δm=0.33 ppm, 0.00 Th
SPSGEVSHPR
Relative Intensity
Δm (ppm)
m/z

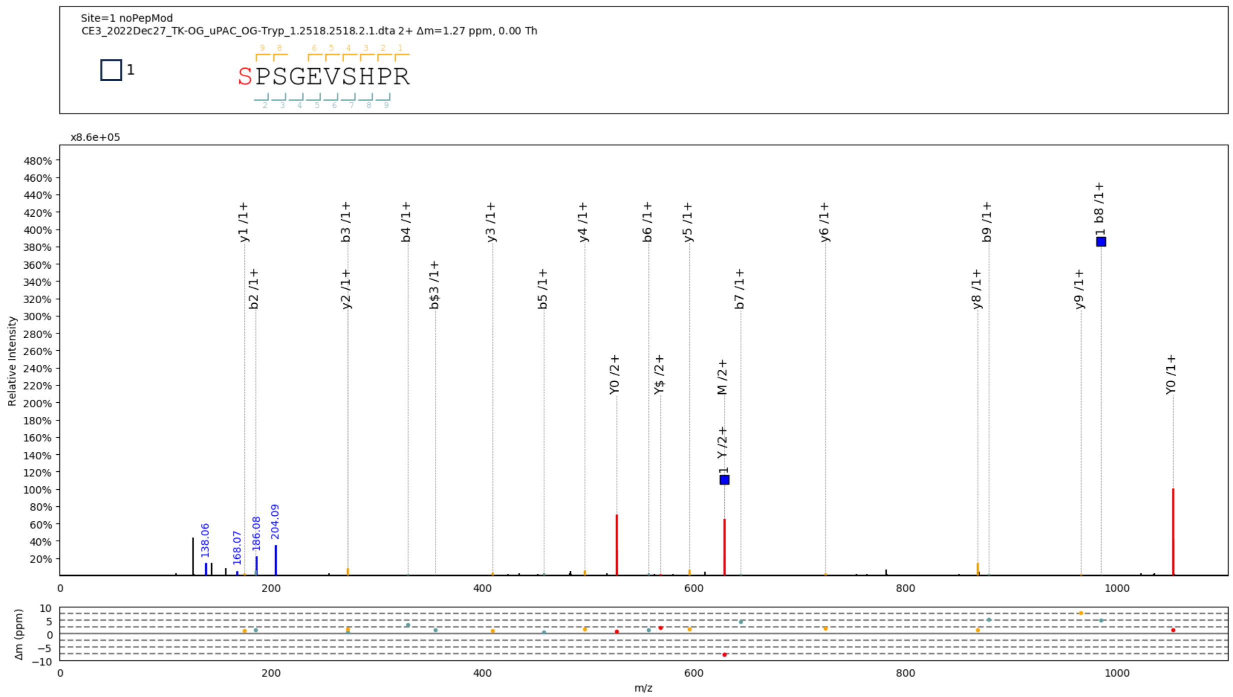
Site=1 noPepMod
CE3_2022Dec27_TK-OG_uPAC_OG-Tryp_1.2518.2518.2.1.dta 2+ Δm=1.27 ppm, 0.00 Th
1
SPSGEVSHPR
x8.6e+05
Relative Intensity
y1 /1+
b2 /1+
b3 /1+
y2 /1+
b4 /1+
b$3 /1+
y3 /1+
b5 /1+
y4 /1+
Y0 /2+
b6 /1+
Y$ /2+
y5 /1+
M /2+
1 Y /2+
b7 /1+
y6 /1+
y8 /1+
b9 /1+
y9 /1+
1 b8 /1+
Y0 /1+
138.06
168.07
186.08
204.09
Δm (ppm)
m/z

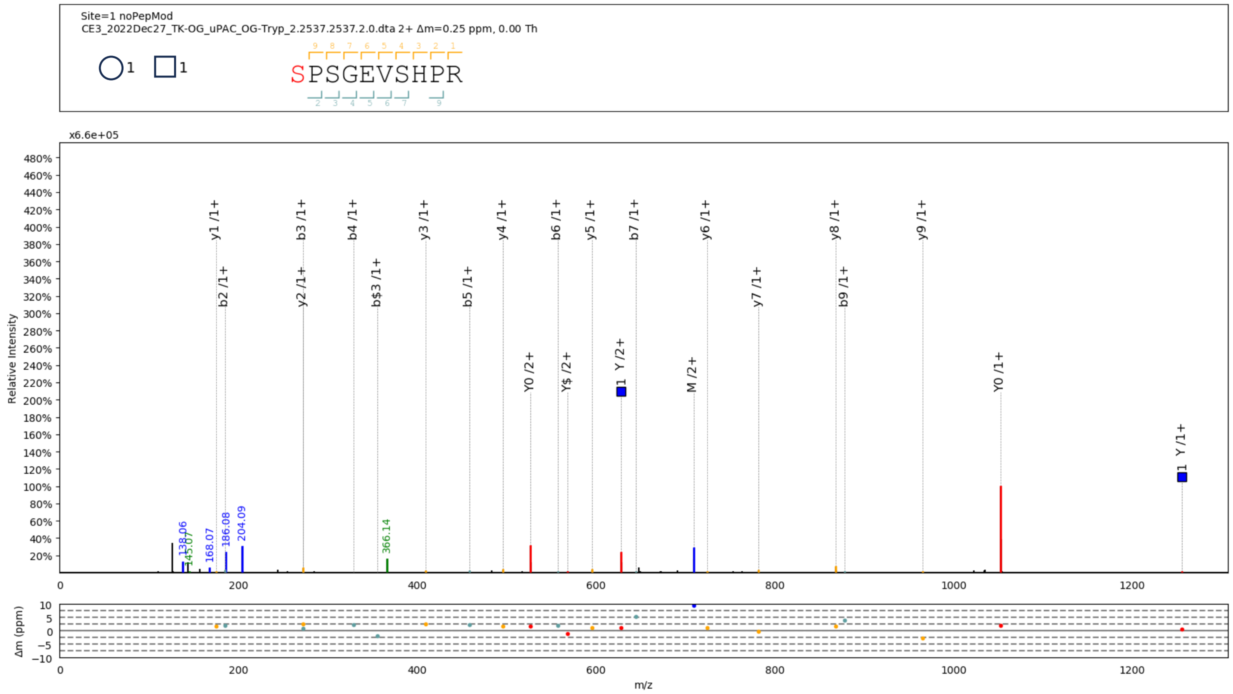
Site=1 noPepMod
CE3_2022Dec27_TK-OG_uPAC_OG-Tryp_2.2537.2537.2.0.dta 2+ Δm=0.25 ppm, 0.00 Th
1 1
SPSGEVSHPR
x6.6e+05
Relative Intensity
y1 /1+
b2 /1+
b3 /1+
y2 /1+
b4 /1+
b$3 /1+
y3 /1+
b5 /1+
y4 /1+
Y0 /2+
b6 /1+
Y$ /2+
y5 /1+
1 Y /2+
b7 /1+
M /2+
y6 /1+
y7 /1+
y8 /1+
b9 /1+
y9 /1+
Y0 /1+
1 Y /1+
138.06
168.07
186.08
204.09
366.14
Δm (ppm)
m/z

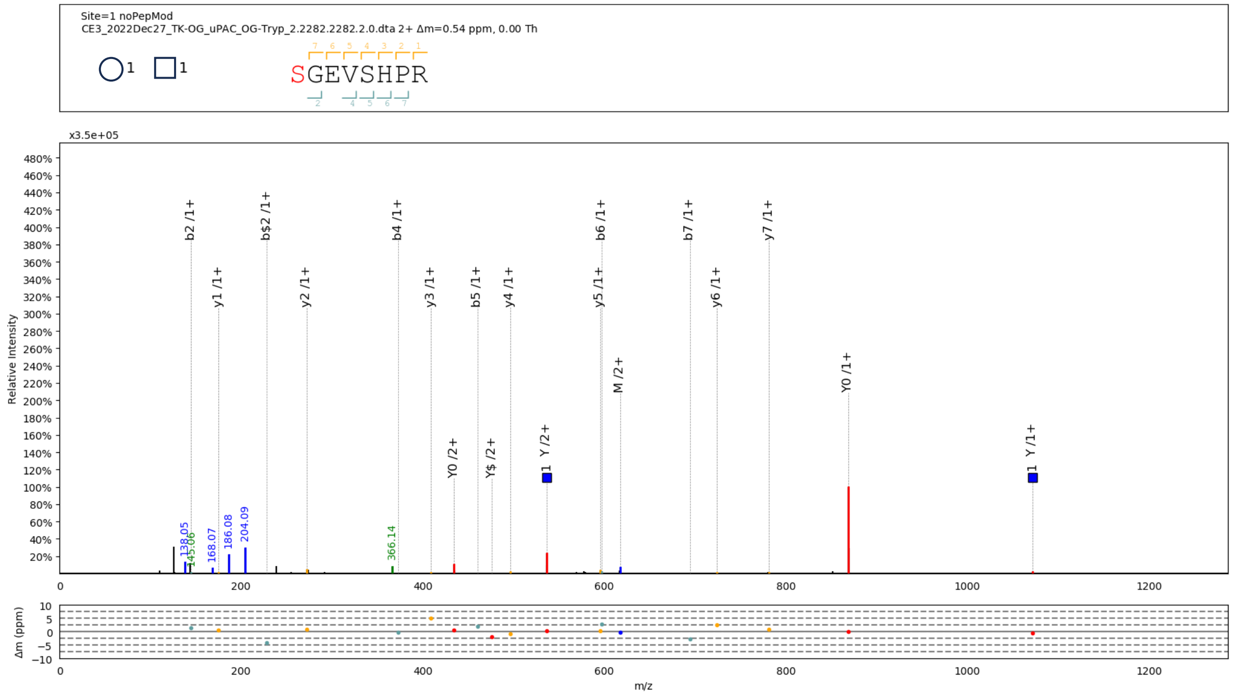

Site=1 noPepMod
CE3_2022Dec27_TK-OG_uPAC_OG-Tryp_2.2282.2282.2.0.dta 2+ Δm=0.54 ppm, 0.00 Th
SGEVSHPR
x3.5e+05
Relative Intensity
Δm (ppm)
m/z

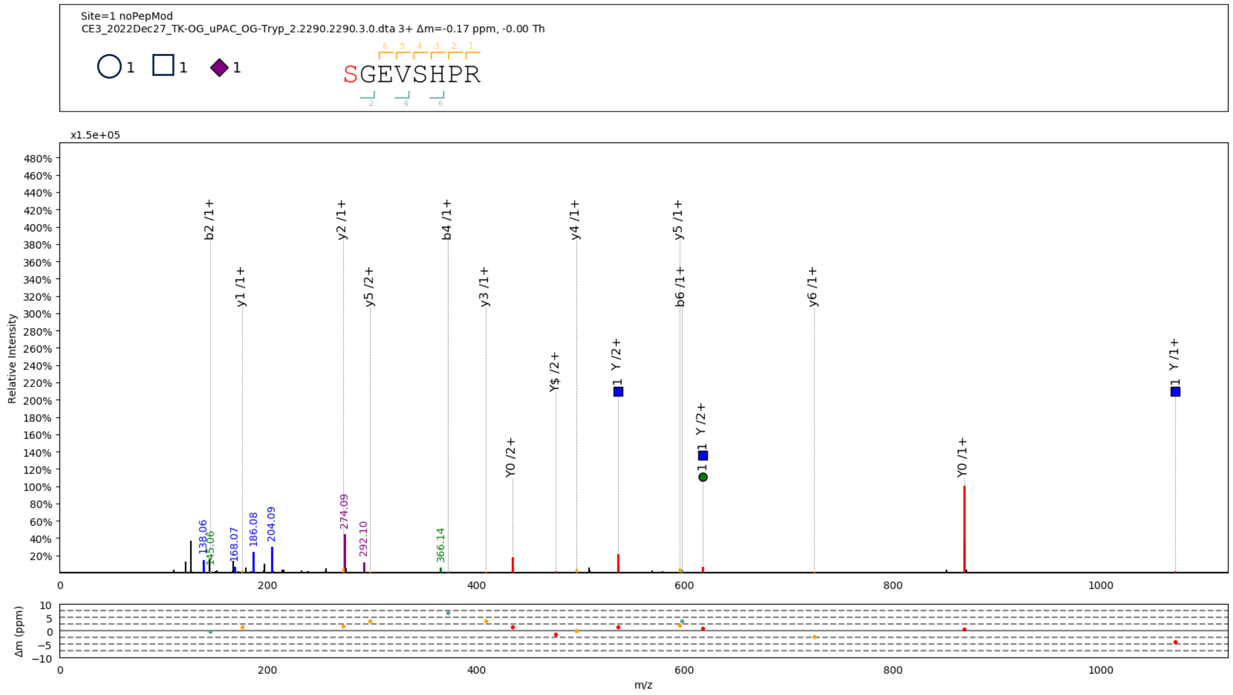

Site=1 noPepMod
CE3_2022Dec27_TK-OG_uPAC_OG-Tryp_2.2290.2290.3.0.dta 3+ Δm=-0.17 ppm, -0.00 Th
SGEVSHPR
x1.5e+05
Relative Intensity
Δm (ppm)
m/z

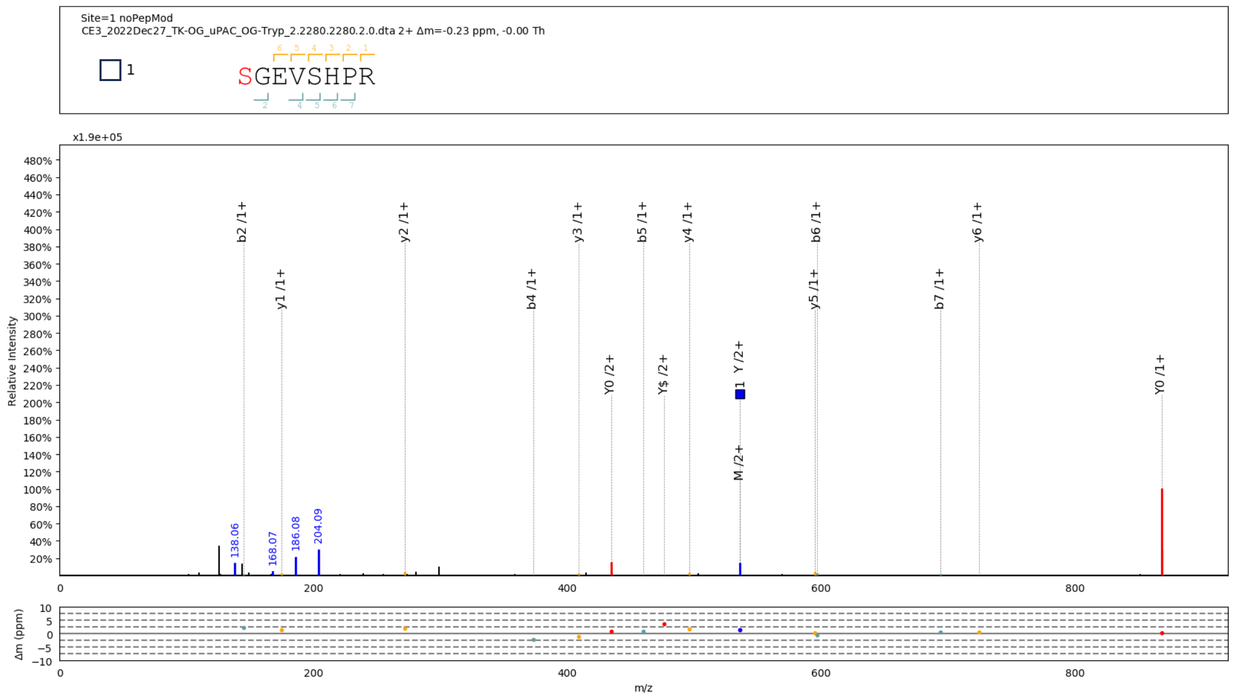
Site=1 noPepMod
CE3_2022Dec27_TK-OG_uPAC_OG-Tryp_2.2280.2280.2.0.dta 2+ Δm=-0.23 ppm, -0.00 Th
1
SGEVSHPR
x1.9e+05
Relative Intensity
Δm (ppm)
m/z

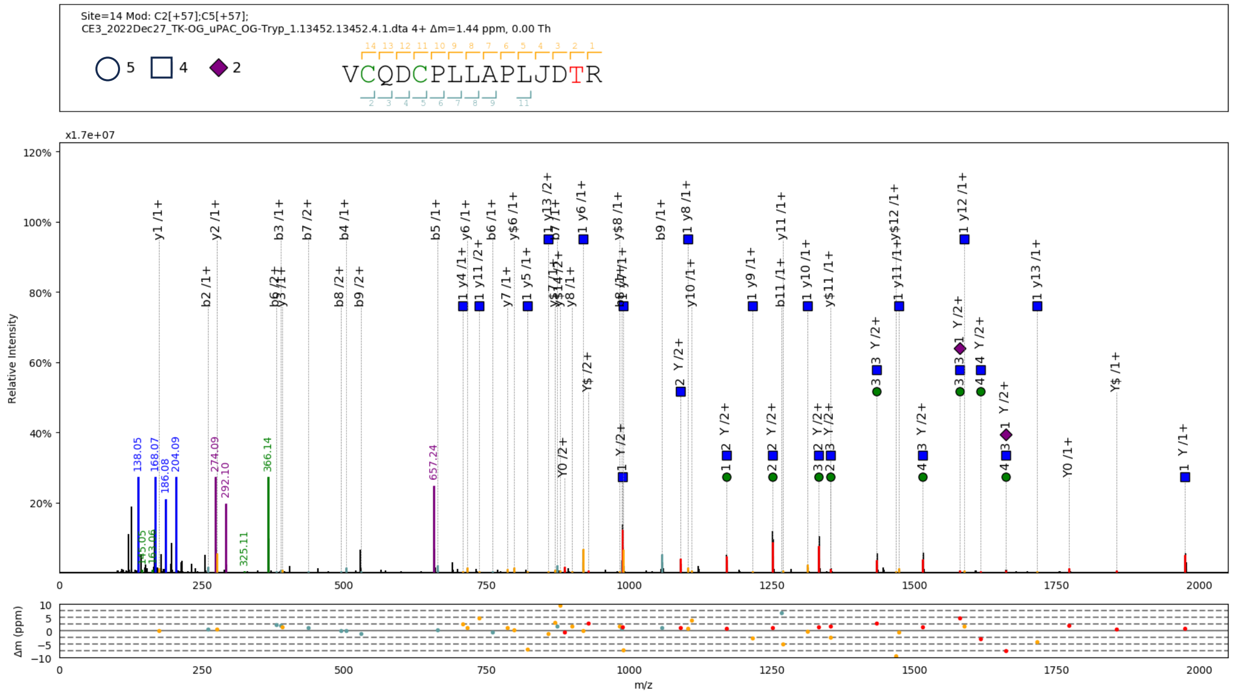
Site=14 Mod: C2[+57];C5[+57];
CE3_2022Dec27_TK-OG_uPAC_OG-Tryp_1.13452.13452.4.1.dta 4+ Δm=1.44 ppm, 0.00 Th
5
4
2
VCQDCPLLAPLJDTR
x1.7e+07
Relative Intensity
Δm (ppm)
m/z

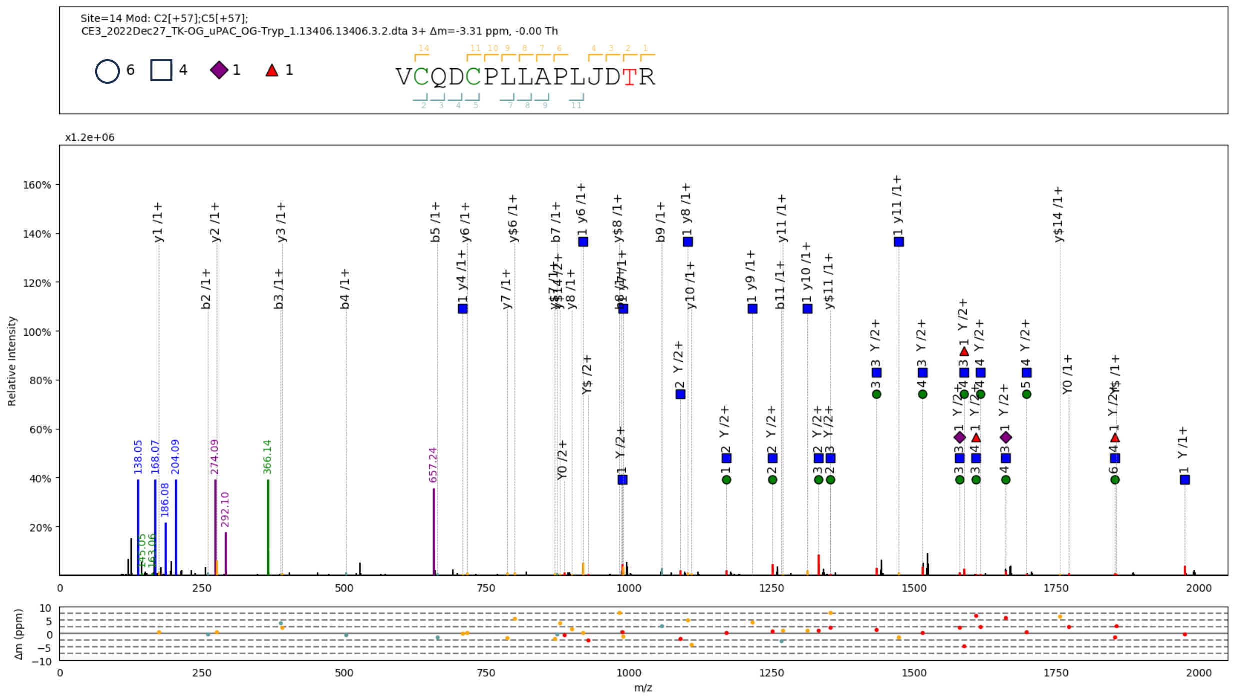
Site=14 Mod: C2[+57];C5[+57];
CE3_2022Dec27_TK-OG_uPAC_OG-Tryp_1.13406.13406.3.2.dta 3+ Δm=-3.31 ppm, -0.00 Th
VCQDCPLLAPLJDTR
x1.2e+06
Relative Intensity
m/z
Δm (ppm)

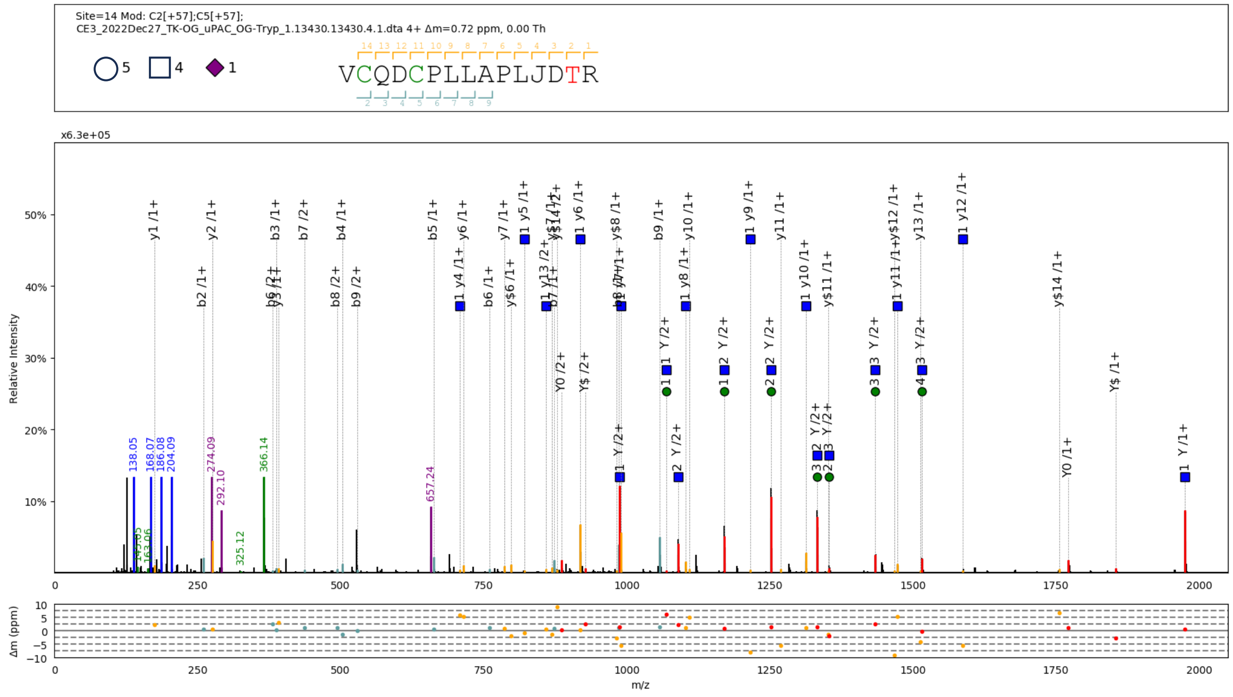
Site=14 Mod: C2[+57];C5[+57];
CE3_2022Dec27_TK-OG_uPAC_OG-Tryp_1.13430.13430.4.1.dta 4+ Δm=0.72 ppm, 0.00 Th
VCQDCPLLAPLJDTR
x6.3e+05
Relative Intensity
m/z
Δm (ppm)

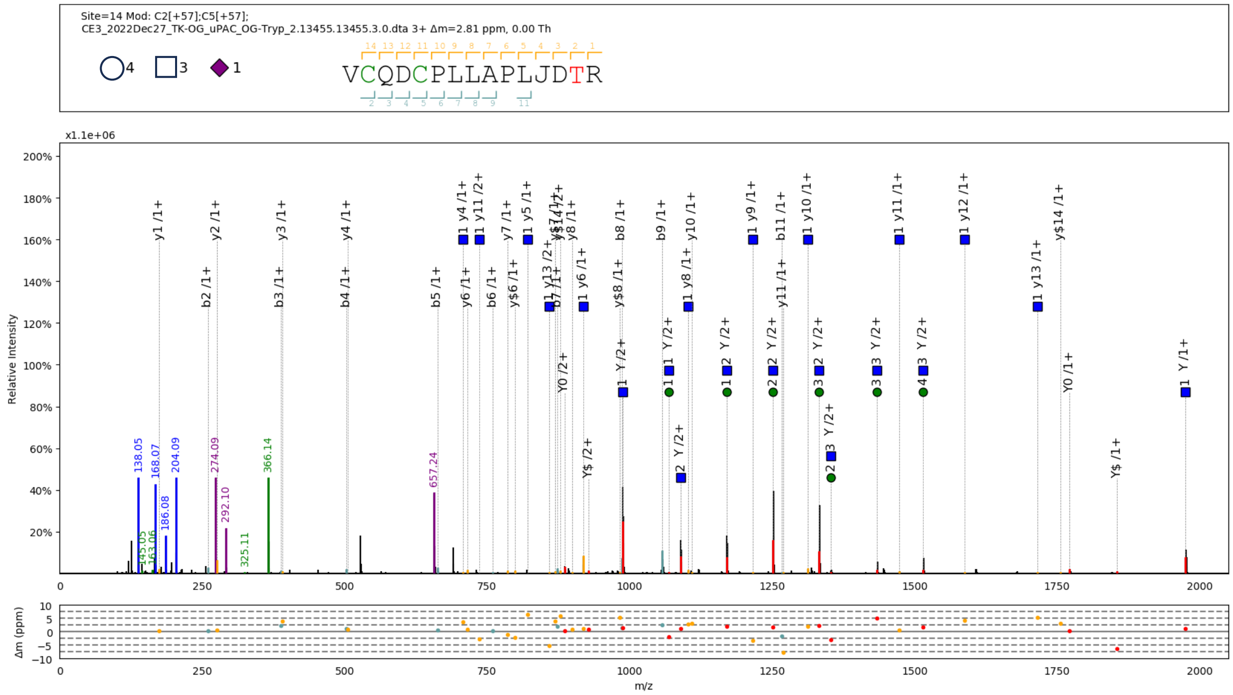
Site=14 Mod: C2[+57];C5[+57];
CE3_2022Dec27_TK-OG_uPAC_OG-Tryp_2.13455.13455.3.0.dta 3+ Δm=2.81 ppm, 0.00 Th
4
3
1
VCQDCPLLAPLJDTR
x1.1e+06
Relative Intensity
Δm (ppm)
m/z

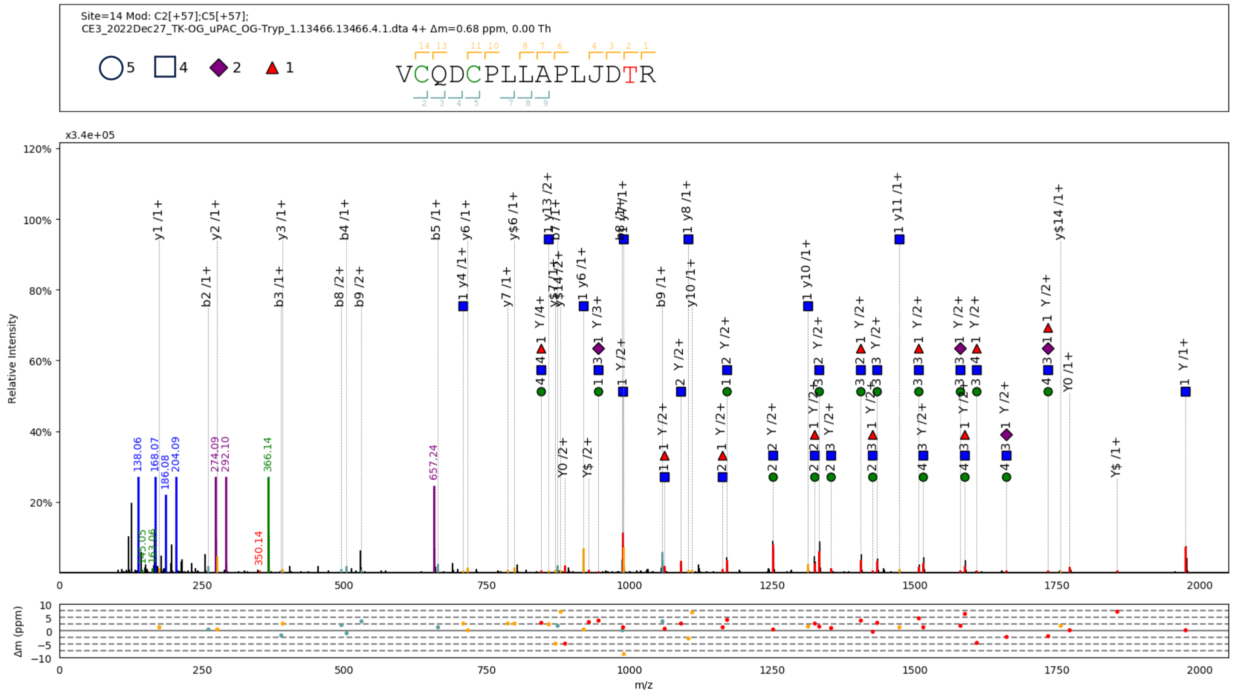
Site=14 Mod: C2[+57];C5[+57];
CE3_2022Dec27_TK-OG_uPAC_OG-Tryp_1.13466.13466.4.1.dta 4+ Δm=0.68 ppm, 0.00 Th
5
4
2
1
VCQDCPLLAPLJDTR
x3.4e+05
Relative Intensity
Δm (ppm)
m/z

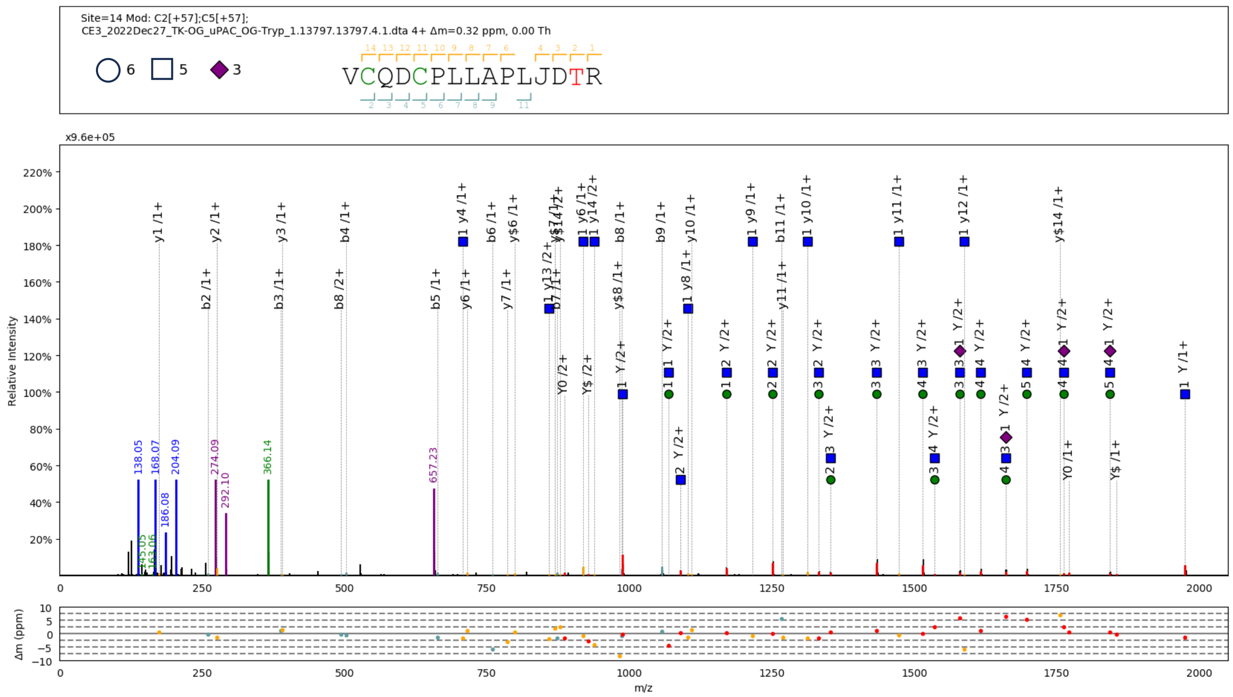
Site=14 Mod: C2[+57];C5[+57];
CE3_2022Dec27_TK-OG_uPAC_OG-Tryp_1.13797.13797.4.1.dta 4+ Δm=0.32 ppm, 0.00 Th
VCQDCPLLAPLJDTR
Relative Intensity
Δm (ppm)
m/z

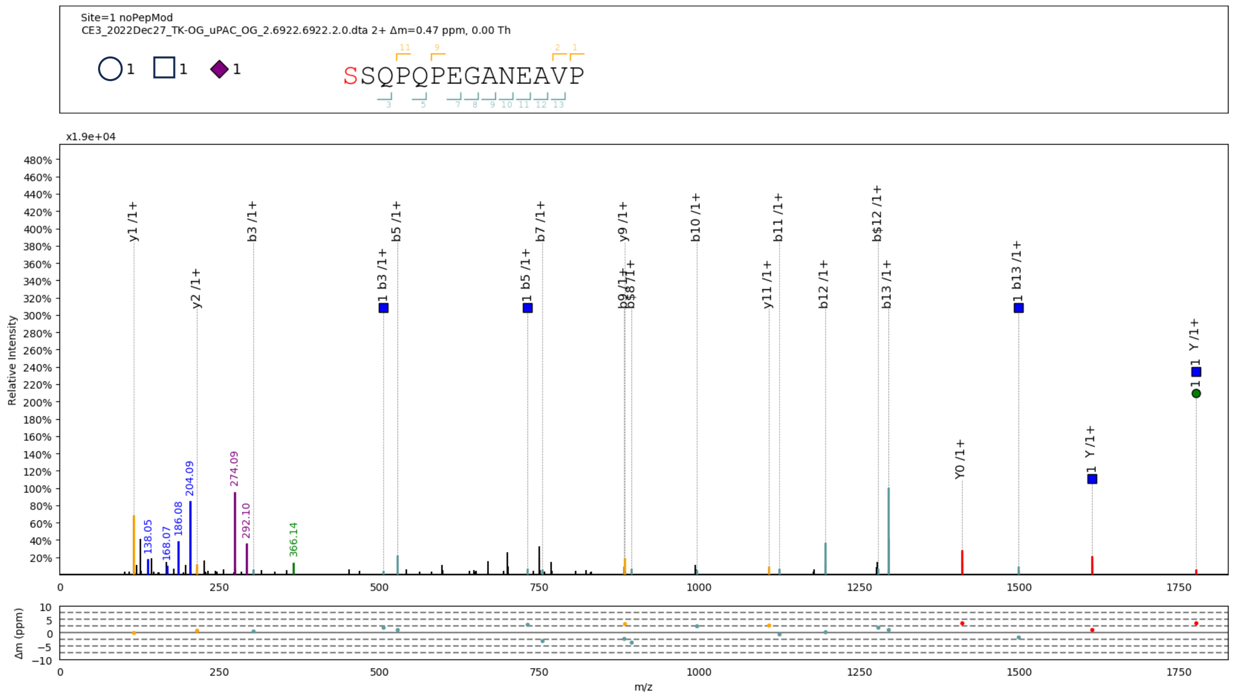
Site=1 noPepMod
CE3_2022Dec27_TK-OG_uPAC_OG_2.6922.6922.2.0.dta 2+ Δm=0.47 ppm, 0.00 Th
SSQPQPEGANEAVP
Relative Intensity
Δm (ppm)
m/z

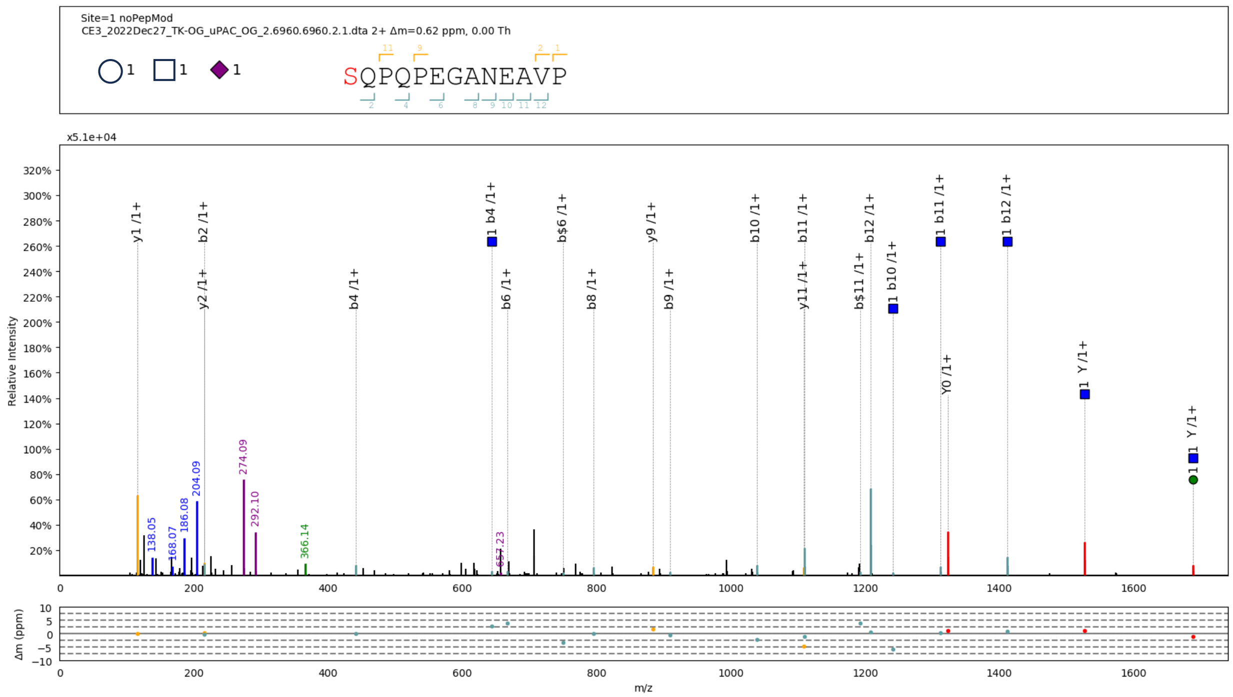
Site=1 noPepMod
CE3_2022Dec27_TK-OG_uPAC_OG_2.6960.6960.2.1.dta 2+ Δm=0.62 ppm, 0.00 Th
SQPQPEGANEAVP
x5.1e+04
Relative Intensity
y1 /1+
b2 /1+
y2 /1+
b4 /1+
b4 /1+
b6 /1+
b$6 /1+
b8 /1+
y9 /1+
b9 /1+
b10 /1+
b11 /1+
y11 /1+
b$11 /1+
b12 /1+
b10 /1+
b11 /1+
Y0 /1+
b12 /1+
Y /1+
Y /1+
138.05
168.07
186.08
204.09
274.09
292.10
366.14
657.23
Δm (ppm)
m/z

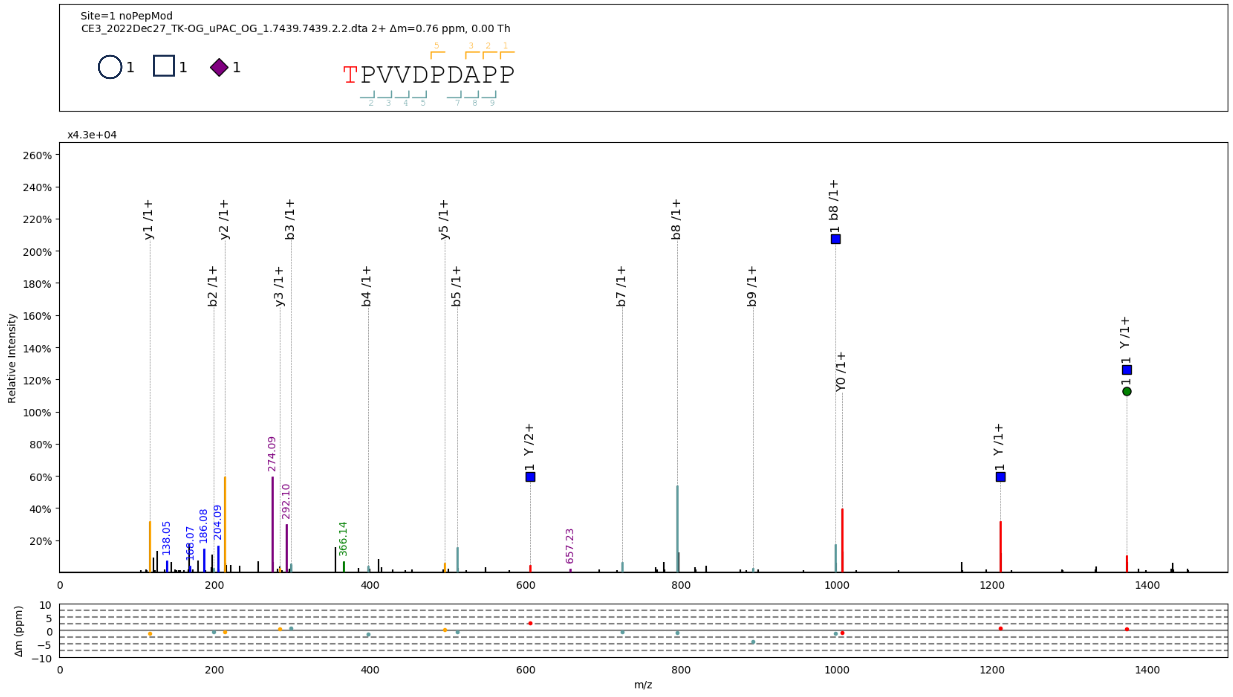
Site=1 noPepMod
CE3_2022Dec27_TK-OG_uPAC_OG_1.7439.7439.2.2.dta 2+ Δm=0.76 ppm, 0.00 Th
TPVVDPDAPP
x4.3e+04
Relative Intensity
y1 /1+
y2 /1+
b3 /1+
b2 /1+
y3 /1+
b4 /1+
y5 /1+
b5 /1+
b7 /1+
b8 /1+
b9 /1+
b8 /1+
Y0 /1+
Y /2+
Y /1+
Y /1+
138.05
168.07
186.08
204.09
274.09
292.10
366.14
657.23
Δm (ppm)
m/z

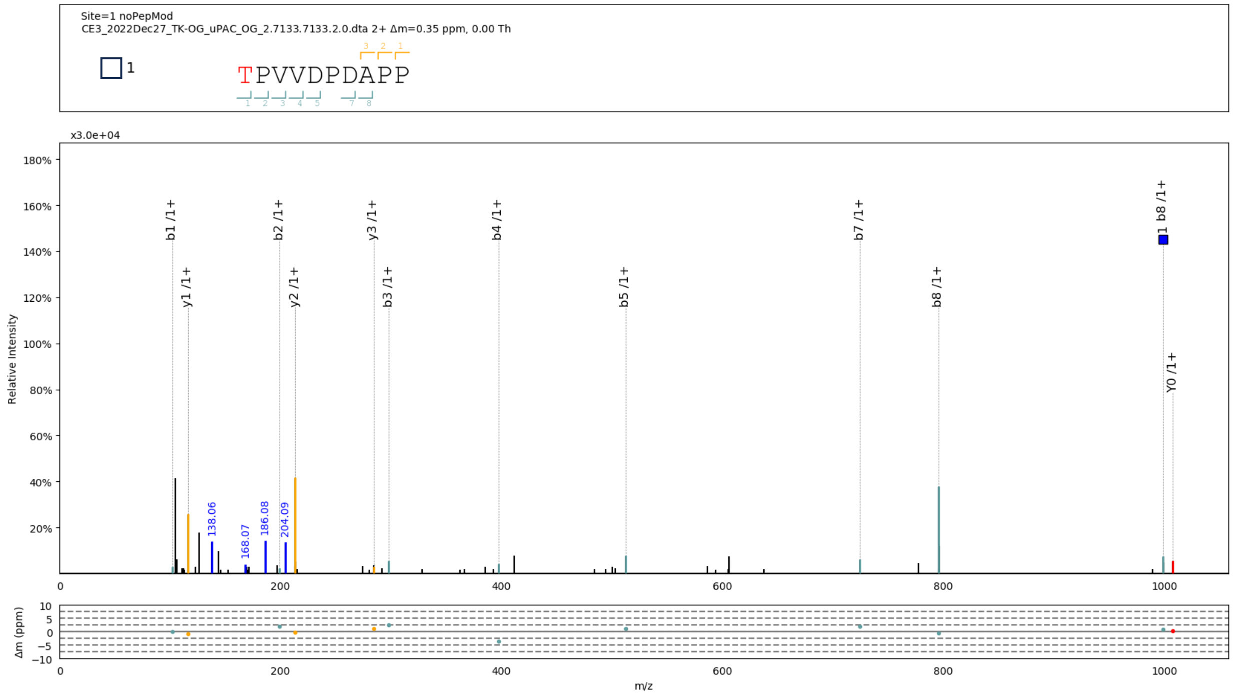
Site=1 noPepMod
CE3_2022Dec27_TK-OG_uPAC_OG_2.7133.7133.2.0.dta 2+ Δm=0.35 ppm, 0.00 Th
1
TPVVDPDAPP
x3.0e+04
Relative Intensity
b1 /1+
y1 /1+
b2 /1+
y2 /1+
y3 /1+
b3 /1+
b4 /1+
b5 /1+
b7 /1+
b8 /1+
1 b8 /1+
Y0 /1+
138.06
168.07
186.08
204.09
Δm (ppm)
m/z

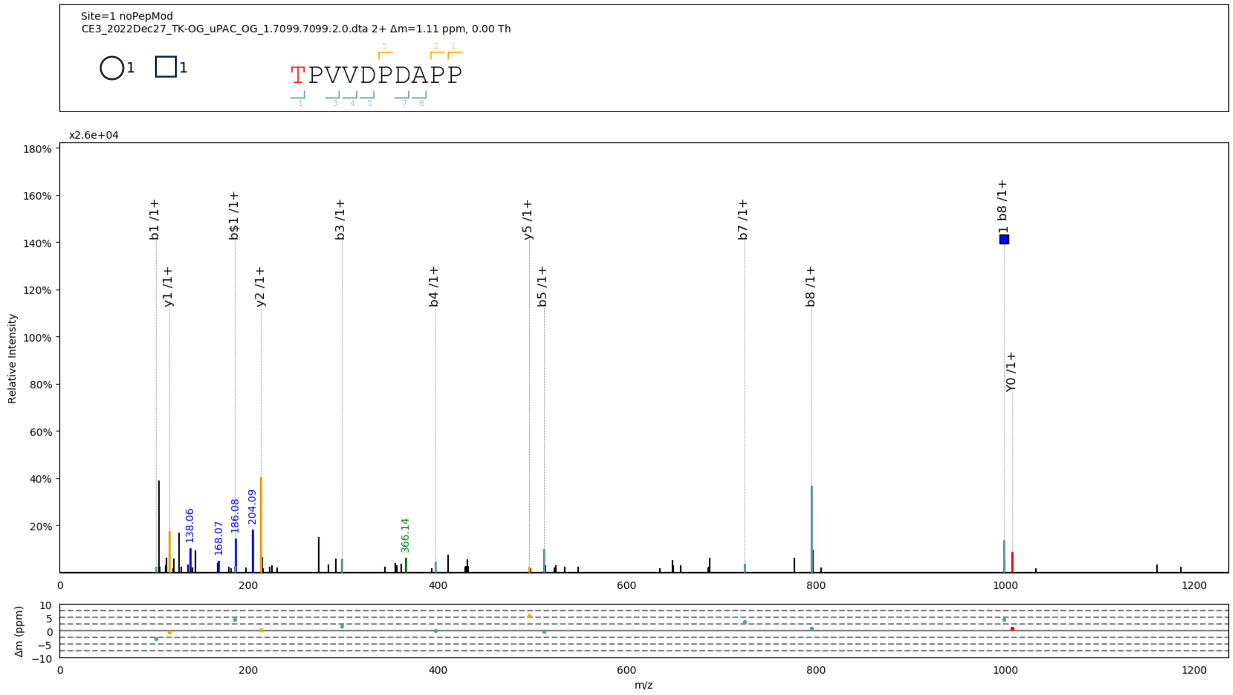
Site=1 noPepMod
CE3_2022Dec27_TK-OG_uPAC_OG_1.7099.7099.2.0.dta 2+ Δm=1.11 ppm, 0.00 Th
1
1
TPVVDPDAPP
x2.6e+04
Relative Intensity
b1 /1+
y1 /1+
b$1 /1+
y2 /1+
b3 /1+
b4 /1+
y5 /1+
b5 /1+
b7 /1+
b8 /1+
1 b8 /1+
Y0 /1+
138.06
168.07
186.08
204.09
366.14
Δm (ppm)
m/z

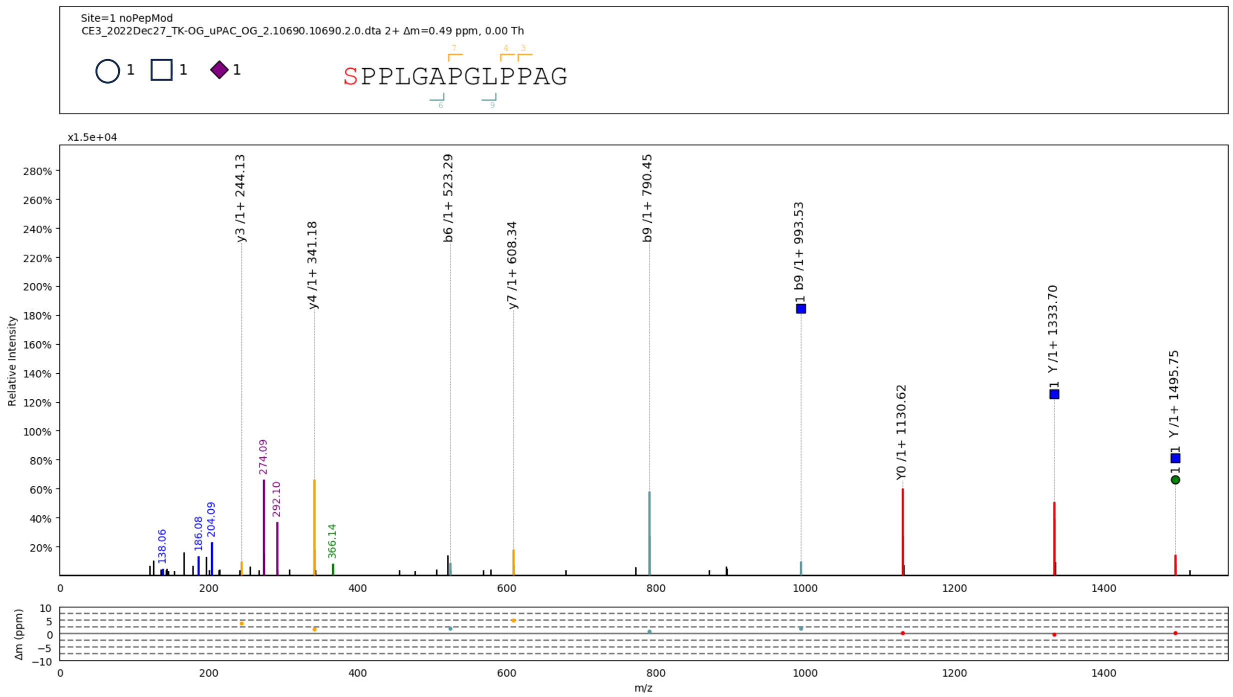

Site=1 noPepMod
CE3_2022Dec27_TK-OG_uPAC_OG_2.10690.10690.2.0.dta 2+ Δm=0.49 ppm, 0.00 Th
SPPLGAPGLPPAG
x1.5e+04
Relative Intensity
y3 /1+ 244.13
y4 /1+ 341.18
b6 /1+ 523.29
y7 /1+ 608.34
b9 /1+ 790.45
1 b9 /1+ 993.53
Y0 /1+ 1130.62
1 Y /1+ 1333.70
1 1 Y /1+ 1495.75
138.06
186.08
204.09
274.09
292.10
366.14
m/z
Δm (ppm)

(B) Kininogen-1 (KNG1)

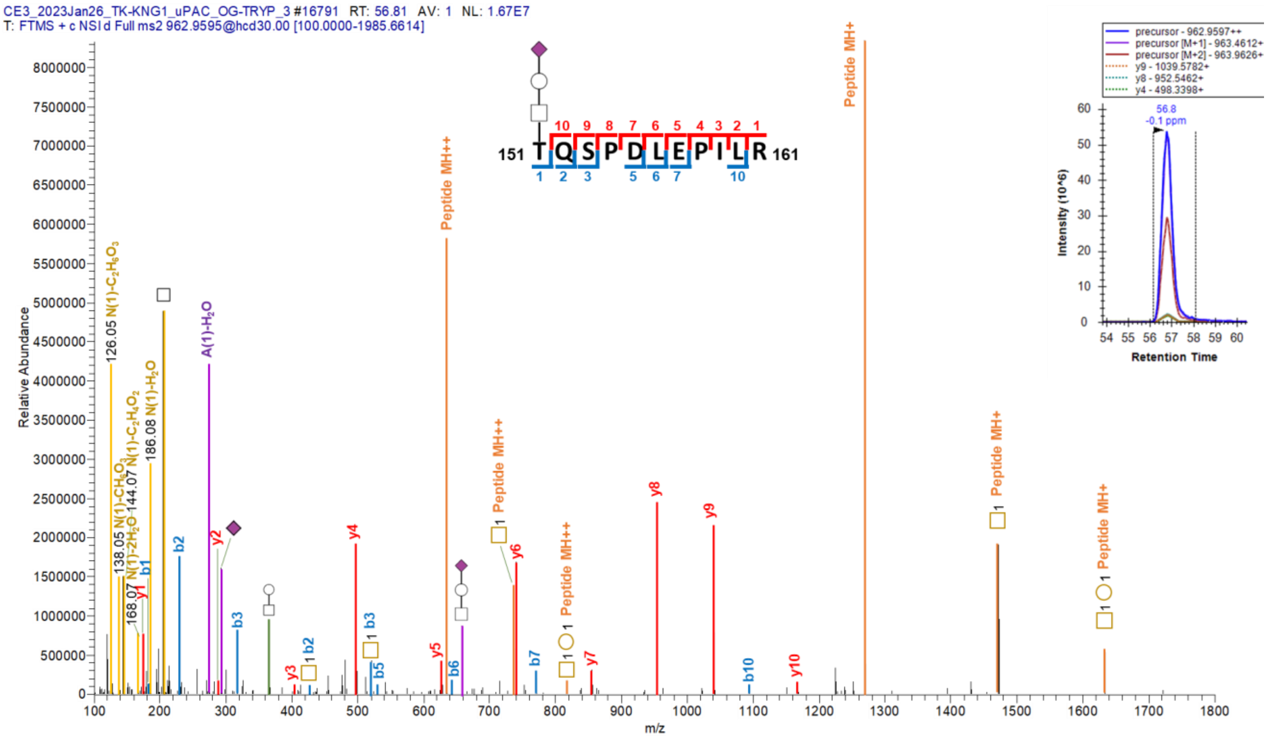

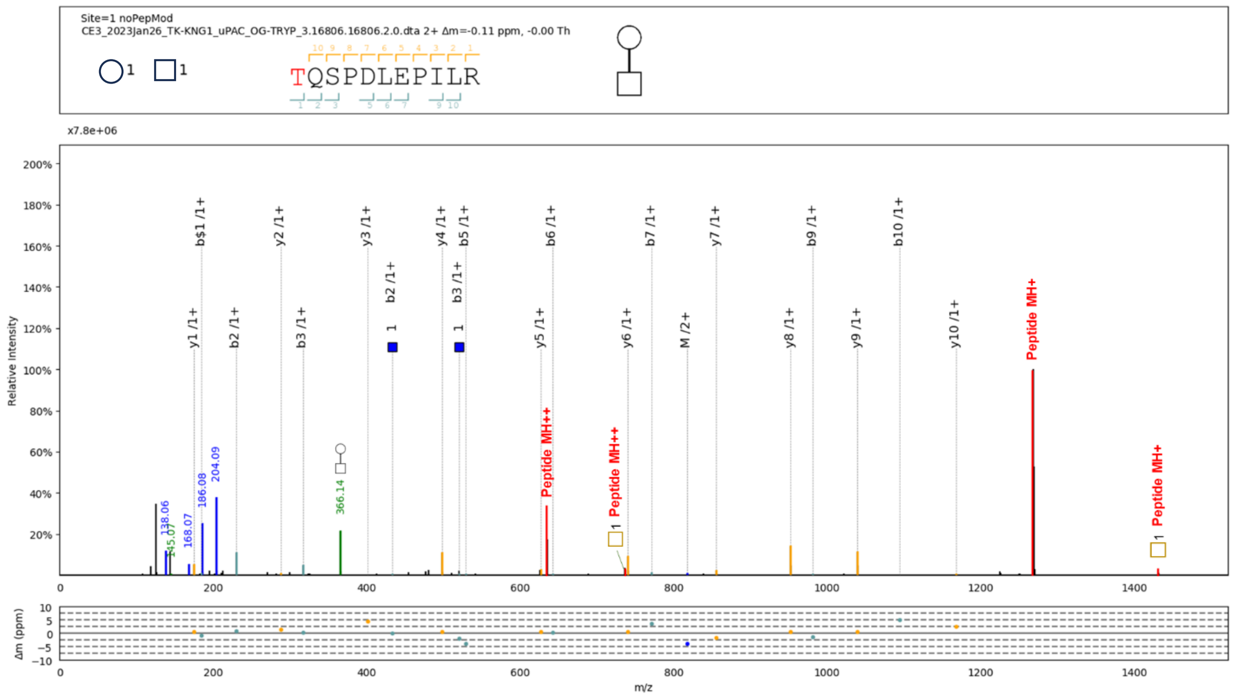

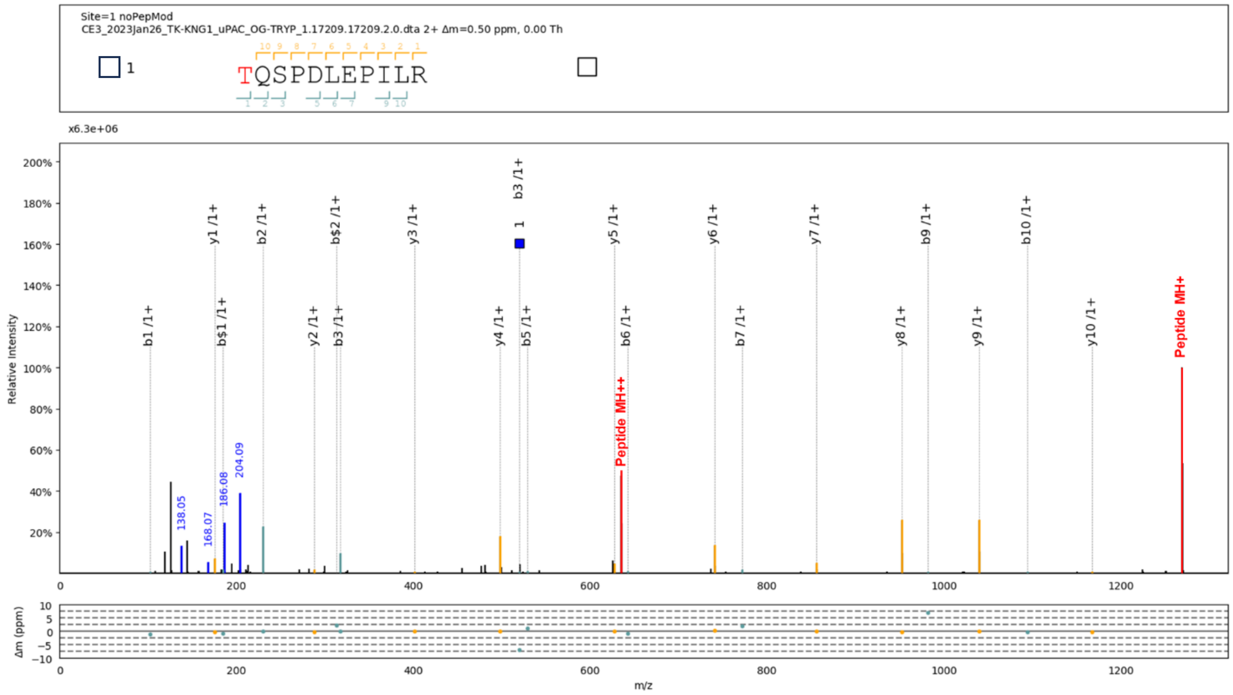
Site=1 noPepMod
CE3_2023Jan26_TK-KNG1_uPAC_OG-TRYP_1.17209.17209.2.0.dta 2+ Δm=0.50 ppm, 0.00 Th
1
TQSPDLEPILR
x6.3e+06
Relative Intensity
b1 /1+
y1 /1+
b$1 /1+
b2 /1+
y2 /1+
b$2 /1+
b3 /1+
y3 /1+
y4 /1+
b3 /1+
b5 /1+
y5 /1+
b6 /1+
y6 /1+
b7 /1+
y7 /1+
y8 /1+
b9 /1+
y9 /1+
b10 /1+
y10 /1+
Peptide MH++
Peptide MH+
138.05
168.07
186.08
204.09
Δm (ppm)
m/z

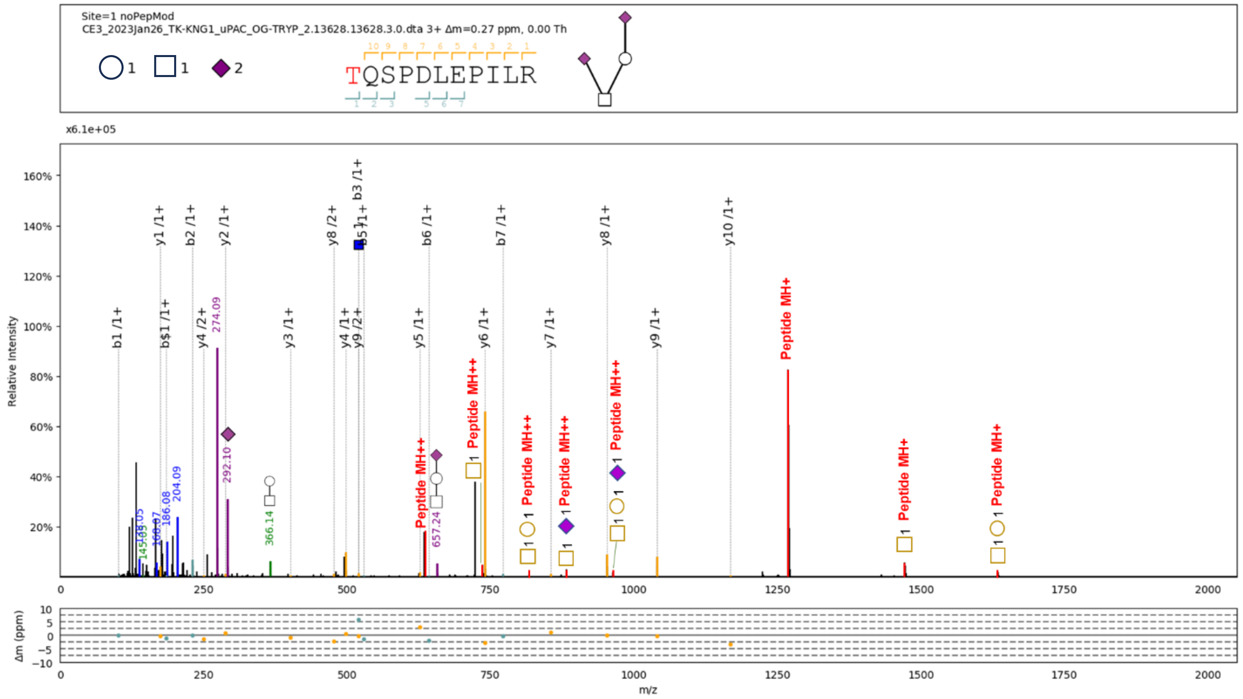
Site=1 noPepMod
CE3_2023Jan26_TK-KNG1_uPAC_OG-TRYP_2.13628.13628.3.0.dta 3+ Δm=0.27 ppm, 0.00 Th
1
1
2
TQSPDLEPILR
x6.1e+05
Relative Intensity
b1 /1+
y1 /1+
b$1 /1+
b2 /1+
y4 /2+
y2 /1+
y3 /1+
y8 /2+
y4 /1+
y9 /2+
b3 /1+
b5 /1+
y5 /1+
b6 /1+
y6 /1+
b7 /1+
y7 /1+
y8 /1+
y9 /1+
y10 /1+
Peptide MH++
Peptide MH+
274.09
292.10
366.14
657.24
186.08
204.09
Δm (ppm)
m/z

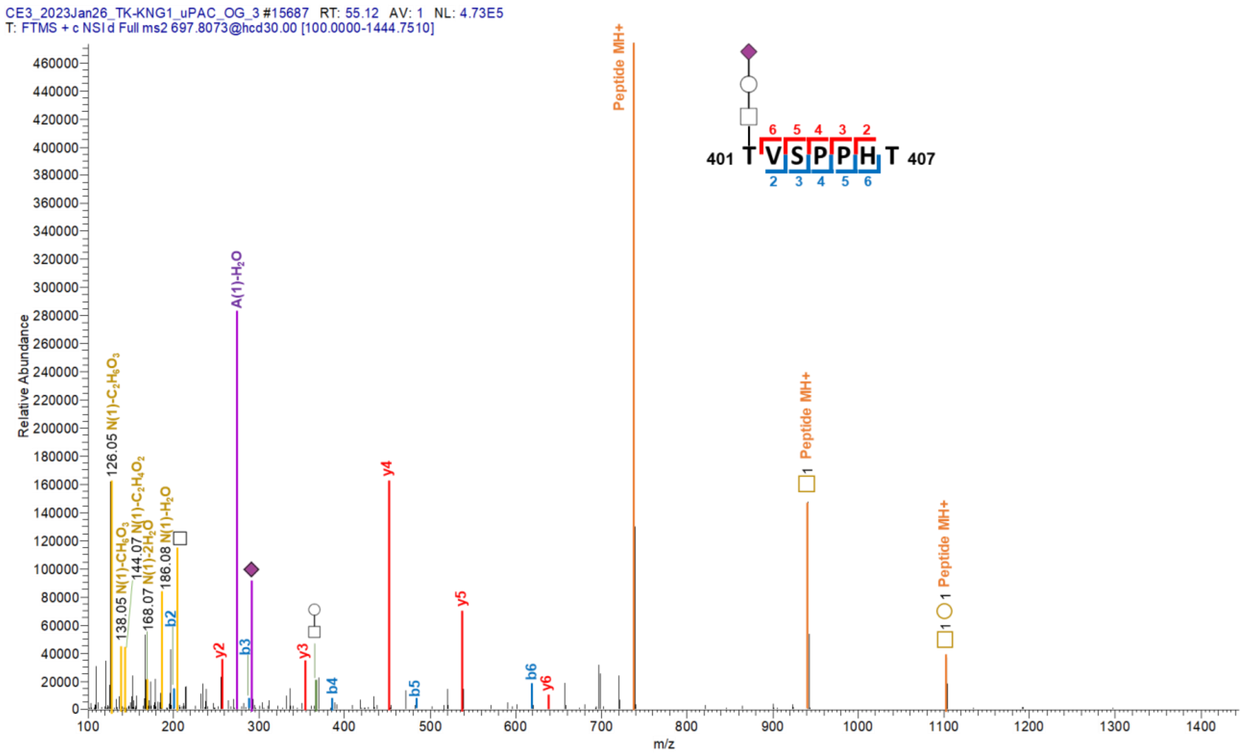
CE3_2023Jan26_TK-KNG1_uPAC_OG_3 #15687 RT: 55.12 AV: 1 NL: 4.73E5
T: FTMS + c NSI d Full ms2 697.8073@hcd30.00 [100.0000-1444.7510]
401 T V S P P H T 407
Peptide MH+
A(1)-H2O
126.05 N(1)-C2H6O3
138.05 N(1)-CH6O3
144.07 N(1)-C2H4O2
168.07 N(1)-2H2O
186.08 N(1)-H2O
y2 y3 y4 y5 y6
b2 b3 b4 b5 b6
Relative Abundance
m/z

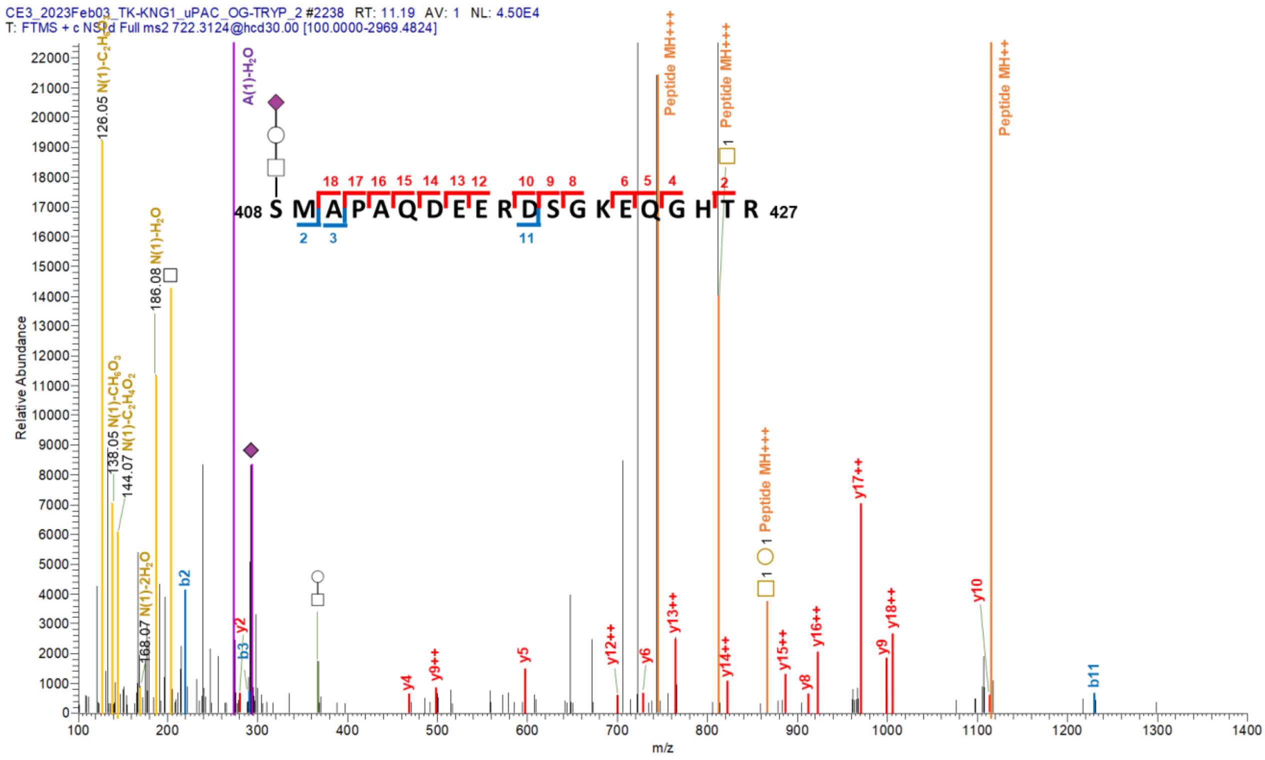
CE3_2023Feb03_TK-KNG1_uPAC_OG-TRYP_2 #2238 RT: 11.19 AV: 1 NL: 4.50E4
T: FTMS + c NSI d Full ms2 722.3124@hcd30.00 [100.0000-2969.4824]
408 S M A P A Q D E E R D S G K E Q G H T R 427
Peptide MH+++
Peptide MH++
A(1)-H2O
126.05 N(1)-C2H6O3
138.05 N(1)-CH6O3
144.07 N(1)-C2H4O2
168.07 N(1)-2H2O
186.08 N(1)-H2O
y2 y4 y5 y6 y8 y9 y10 y9++ y12++ y13++ y14++ y15++ y16++ y17++ y18++
b2 b3 b11
Relative Abundance
m/z

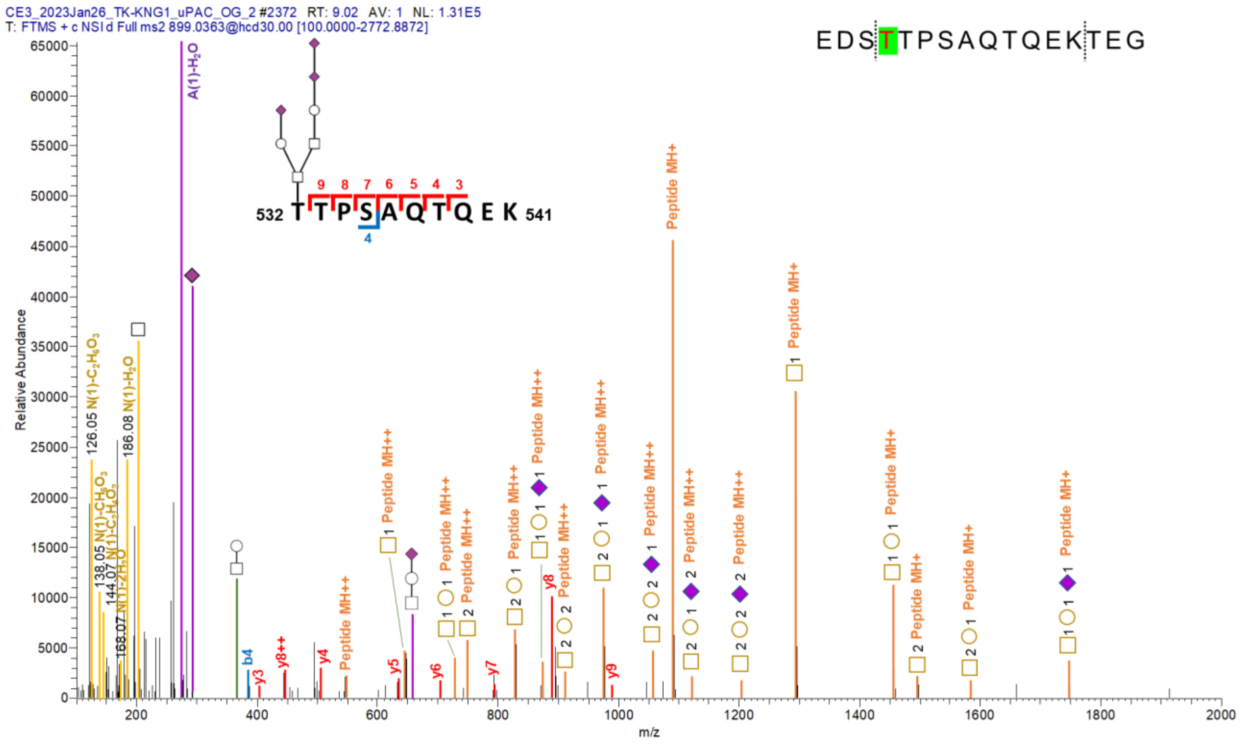
CE3_2023Jan26_TK-KNG1_uPAC_OG_2 #2372 RT: 9.02 AV: 1 NL: 1.31E5
T: FTMS + c NSI d Full ms2 899.0363@hcd30.00 [100.0000-2772.8872]
EDSTTPSAQTQEKTEG
532 T T P S A Q T Q E K 541
9 8 7 6 5 4 3
4
Relative Abundance
m/z
A(1)-H2O
Peptide MH+
Peptide MH++
126.05 N(1)-C2H6O3
138.05 N(1)-CH6O3
144.07 N(1)-C2H4O2
168.07 N(1)-2H2O
186.08 N(1)-H2O
b4
y3
y8++
y4
y5
y6
y7
y8
y9

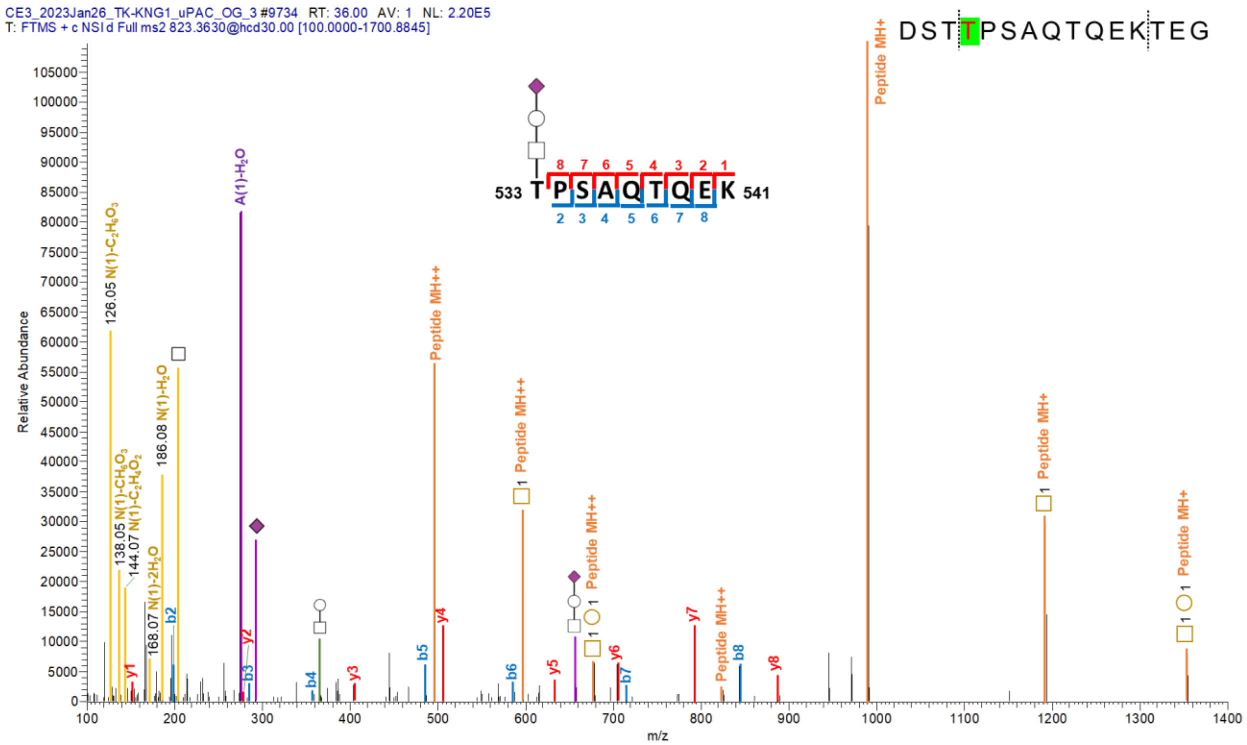
CE3_2023Jan26_TK-KNG1_uPAC_OG_3 #9734 RT: 36.00 AV: 1 NL: 2.20E5
T: FTMS + c NSI d Full ms2 823.3630@hcd30.00 [100.0000-1700.8845]
DSTTPSAQTQEKTEG
533 T P S A Q T Q E K 541
8 7 6 5 4 3 2 1
2 3 4 5 6 7 8
Relative Abundance
m/z
A(1)-H2O
Peptide MH+
Peptide MH++
126.05 N(1)-C2H6O3
138.05 N(1)-CH6O3
144.07 N(1)-C2H4O2
168.07 N(1)-2H2O
186.08 N(1)-H2O
y1
b2
y2
b3
b4
y3
b5
y4
b6
y5
y6
b7
y7
b8
y8

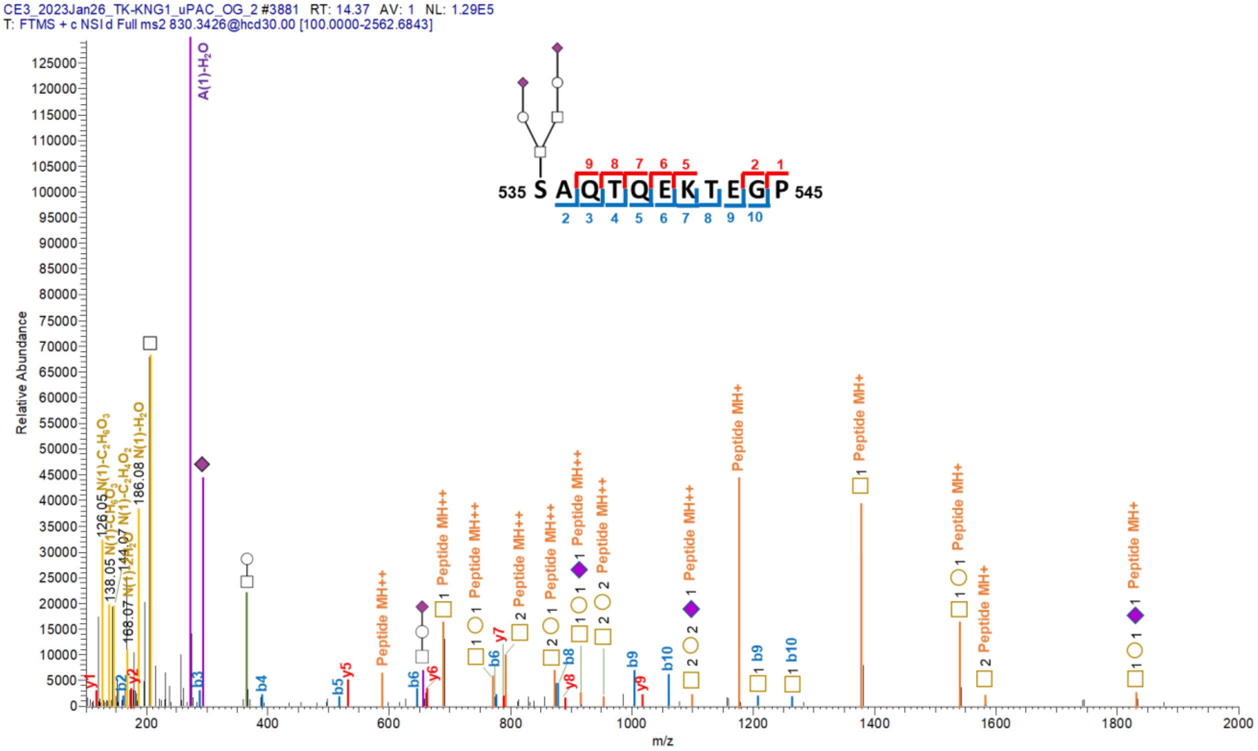

CE3_2023Jan26_TK-KNG1_uPAC_OG_2 #3881 RT: 14.37 AV: 1 NL: 1.29E5
T: FTMS + c NSI d Full ms2 830.3426@hcd30.00 [100.0000-2562.6843]
535 S A Q T Q E K T E G P 545
Relative Abundance
m/z

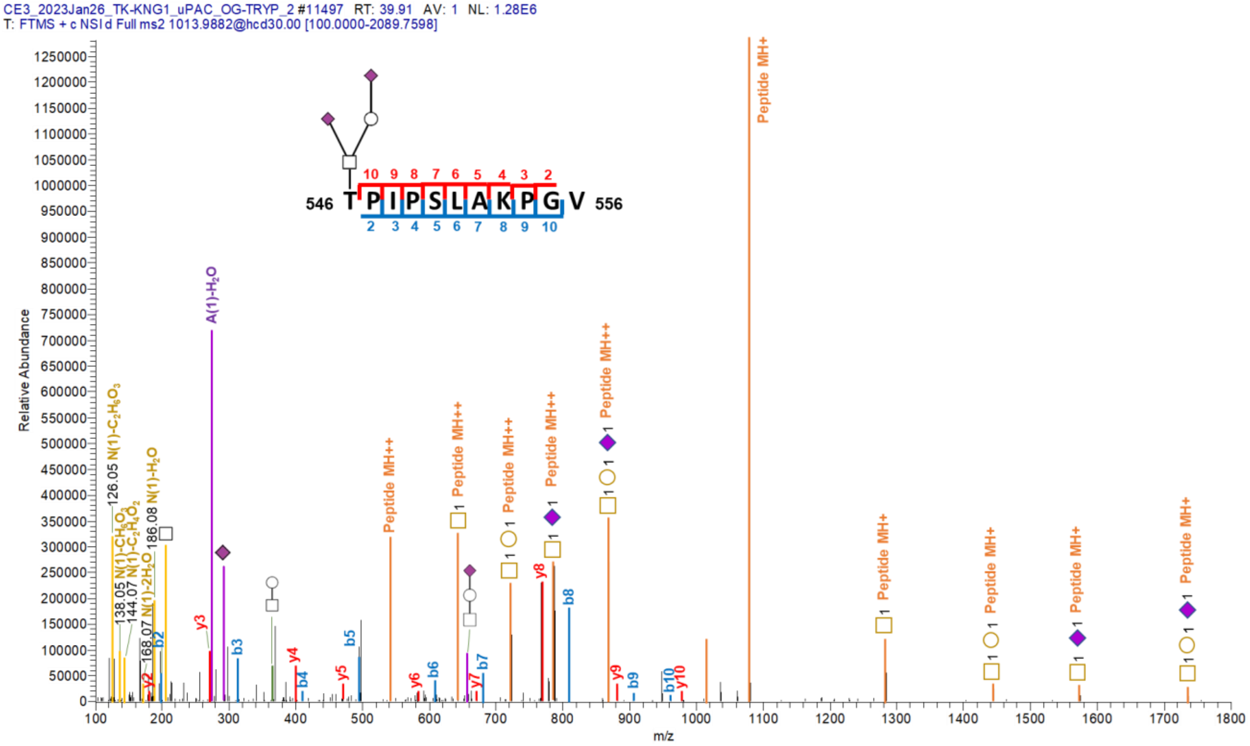

CE3_2023Jan26_TK-KNG1_uPAC_OG-TRYP_2 #11497 RT: 39.91 AV: 1 NL: 1.28E6
T: FTMS + c NSI d Full ms2 1013.9882@hcd30.00 [100.0000-2089.7598]
546 T P I P S L A K P G V 556
Relative Abundance
m/z

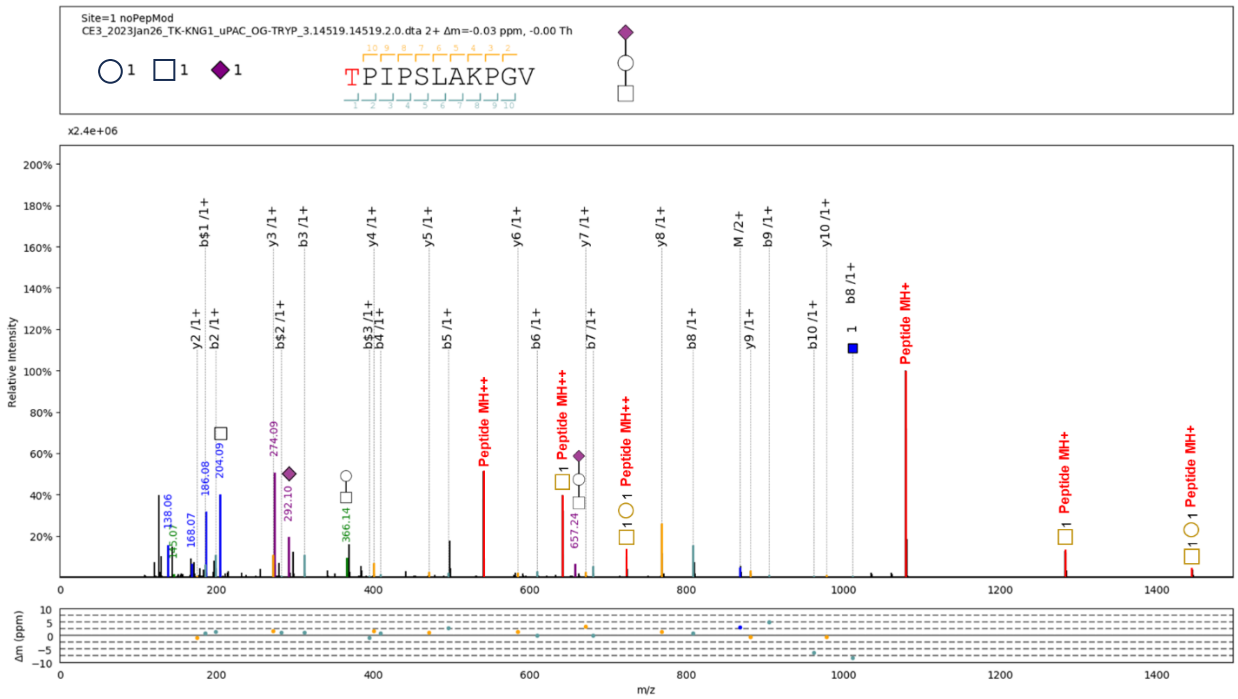
Site=1 noPepMod
CE3_2023Jan26_TK-KNG1_uPAC_OG-TRYP_3.14519.14519.2.0.dta 2+ Δm=-0.03 ppm, -0.00 Th
TPIPSLAKPGV
x2.4e+06
Relative Intensity
b$1 /1+
y3 /1+
b3 /1+
y4 /1+
y5 /1+
y6 /1+
y7 /1+
y8 /1+
M /2+
y9 /1+
y10 /1+
y2 /1+
b2 /1+
b$2 /1+
b$3 /1+
b4 /1+
b5 /1+
b6 /1+
b7 /1+
b8 /1+
b9 /1+
b10 /1+
b8 /1+
Peptide MH+
Peptide MH++
138.06
145.07
168.07
186.08
204.09
274.09
292.10
366.14
657.24
Δm (ppm)
m/z

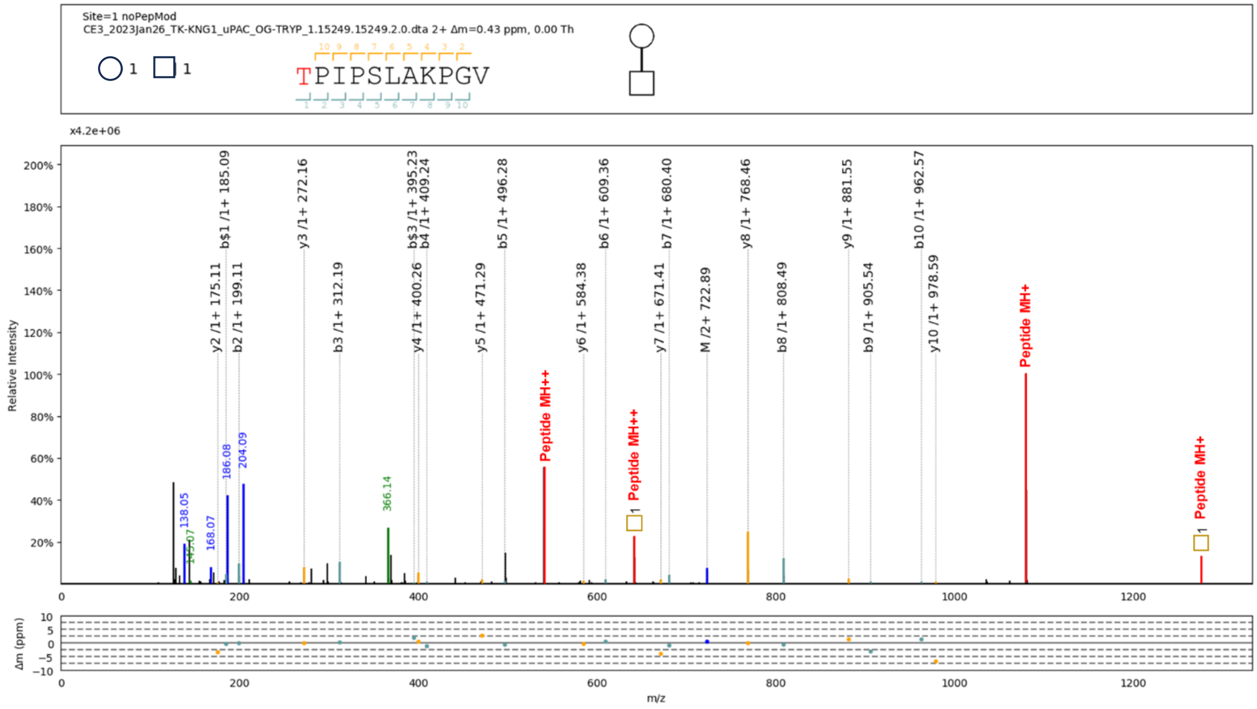
Site=1 noPepMod
CE3_2023Jan26_TK-KNG1_uPAC_OG-TRYP_1.15249.15249.2.0.dta 2+ Δm=0.43 ppm, 0.00 Th
TPIPSLAKPGV
x4.2e+06
Relative Intensity
b$1 /1+ 185.09
y3 /1+ 272.16
b$3 /1+ 395.23
b4 /1+ 409.24
b5 /1+ 496.28
b6 /1+ 609.36
b7 /1+ 680.40
y8 /1+ 768.46
y9 /1+ 881.55
b10 /1+ 962.57
y2 /1+ 175.11
b2 /1+ 199.11
b3 /1+ 312.19
y4 /1+ 400.26
y5 /1+ 471.29
y6 /1+ 584.38
y7 /1+ 671.41
M /2+ 722.89
b8 /1+ 808.49
b9 /1+ 905.54
y10 /1+ 978.59
Peptide MH+
Peptide MH++
138.05
145.07
168.07
186.08
204.09
366.14
Δm (ppm)
m/z

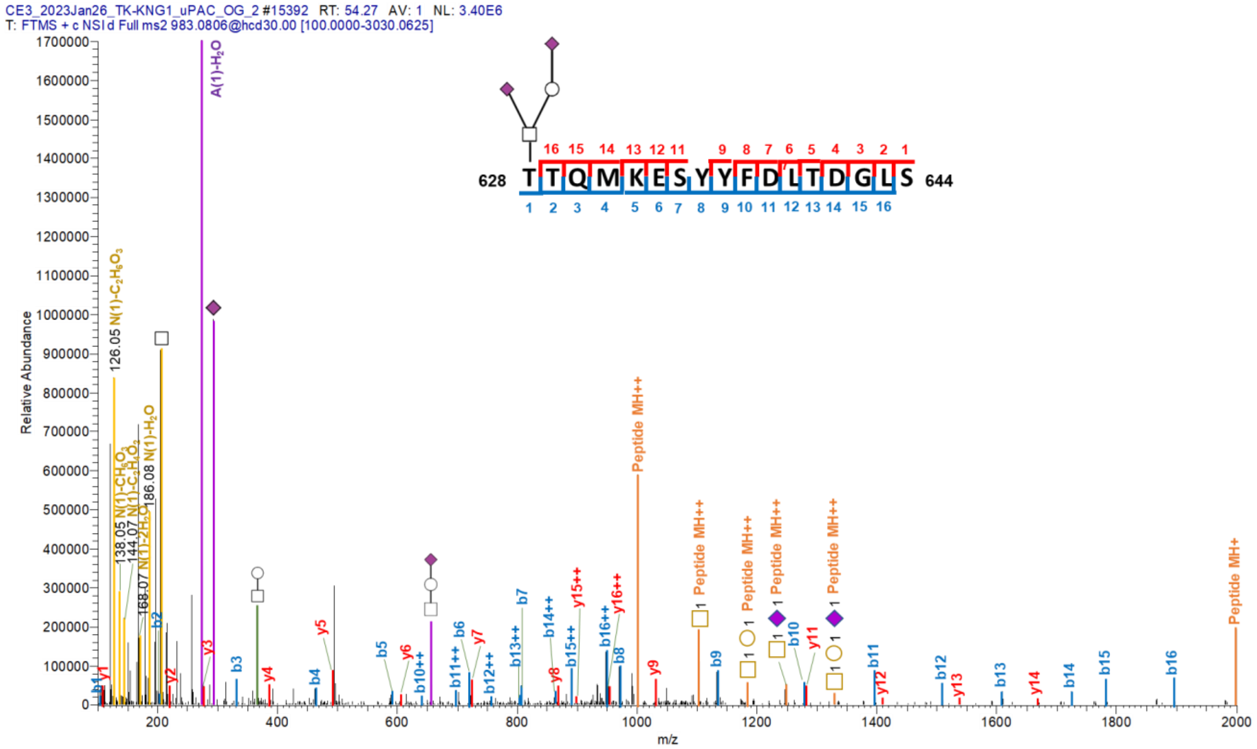
CE3_2023Jan26_TK-KNG1_uPAC_OG_2 #15392 RT: 54.27 AV: 1 NL: 3.40E6
T: FTMS + c NSI d Full ms2 983.0806@hcd30.00 [100.0000-3030.0625]
Relative Abundance
m/z
628 TTQMKESYYFDLTDGLS 644
A(1)-H2O
126.05 N(1)-C2H6O3
186.08 N(1)-H2O
Peptide MH++
Peptide MH+

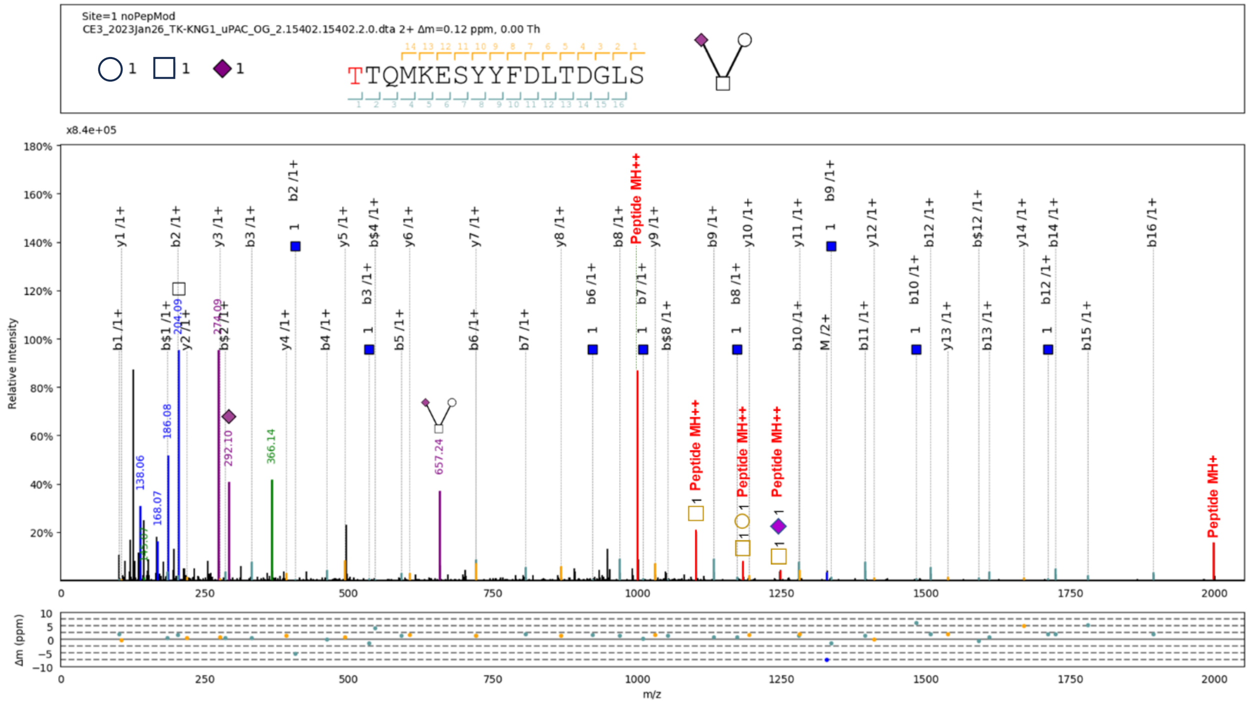
Site=1 noPepMod
CE3_2023Jan26_TK-KNG1_uPAC_OG_2.15402.15402.2.0.dta 2+ Δm=0.12 ppm, 0.00 Th
TTQMKESYYFDLTDGLS
x8.4e+05
Relative Intensity
Δm (ppm)
m/z
Peptide MH++
Peptide MH+

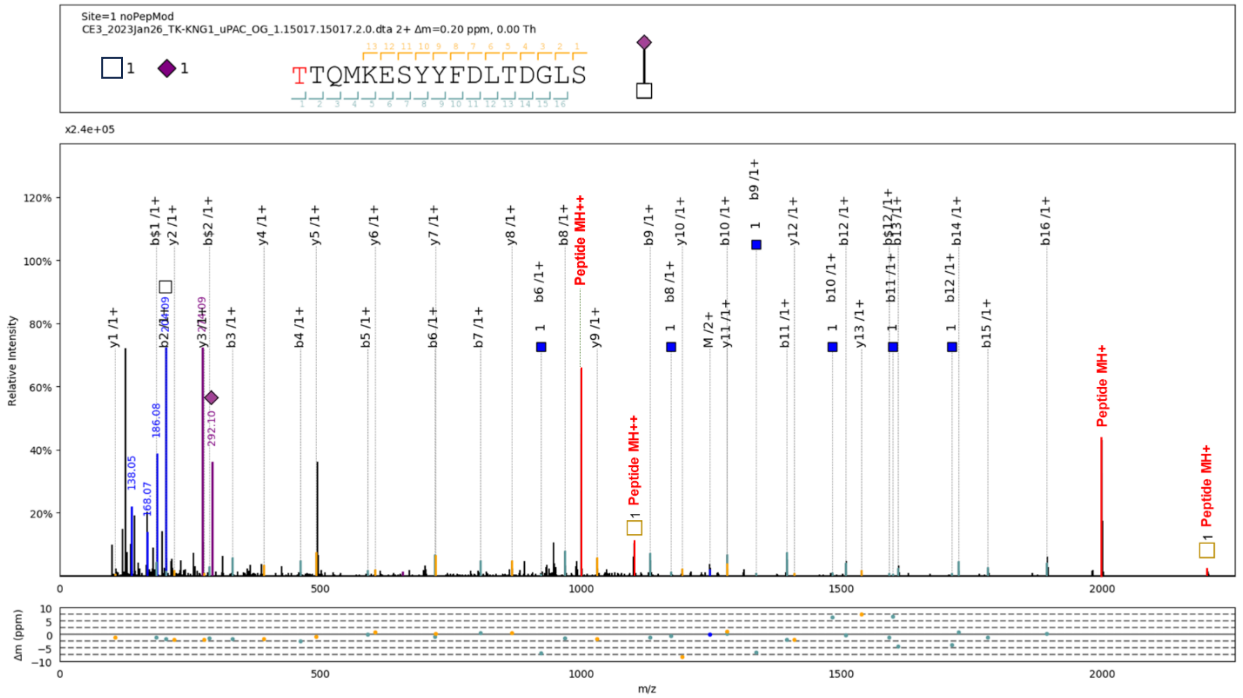

(C) Apolipoprotein E (APOE)

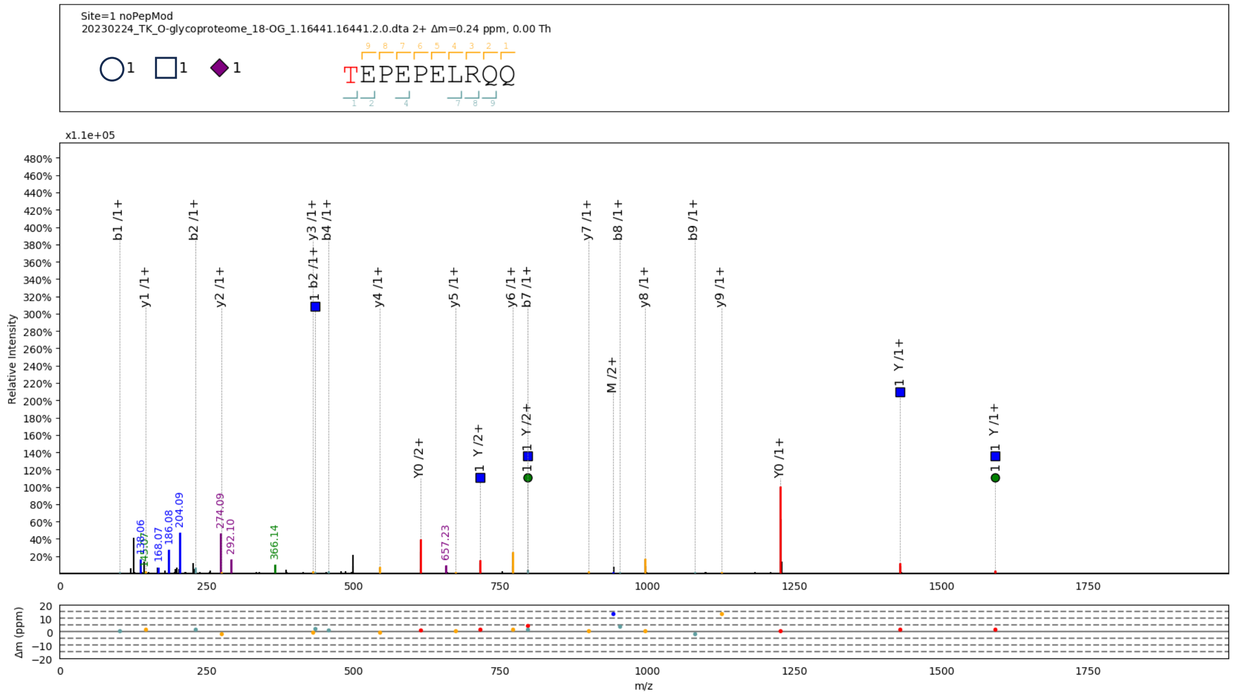

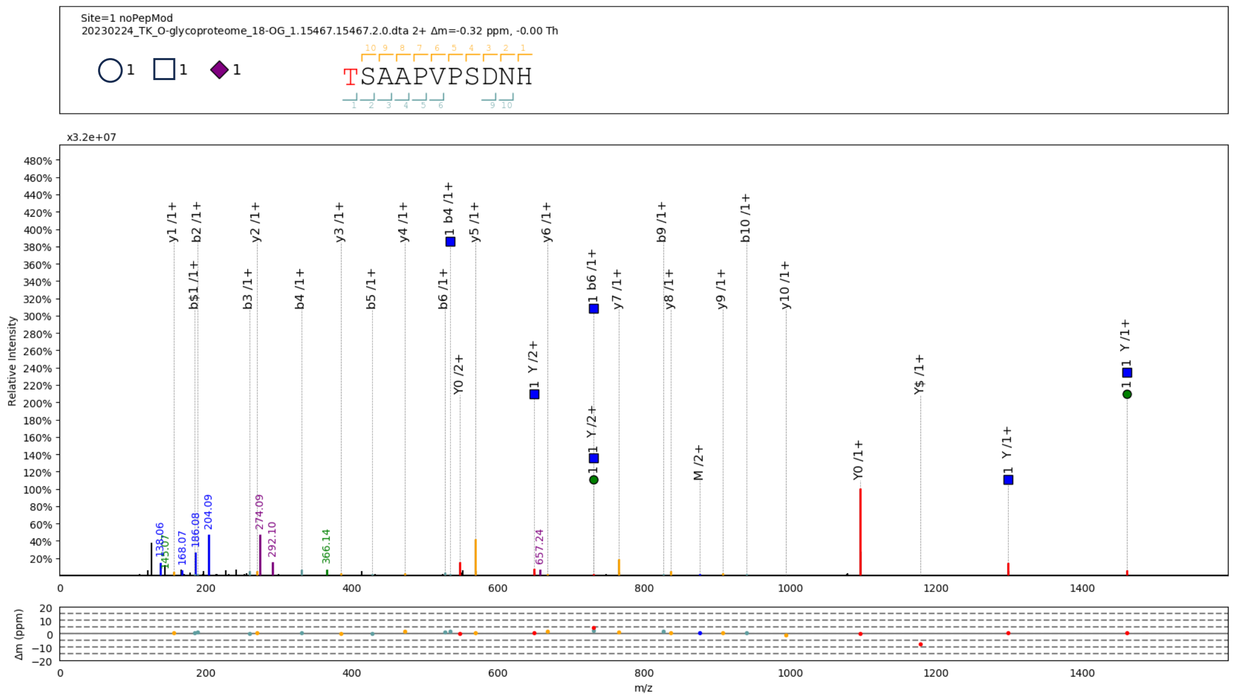

Site=1 noPepMod
20230224_TK_O-glycoproteome_18-OG_1.15467.15467.2.0.dta 2+ Δm=-0.32 ppm, -0.00 Th
TSAAPVPSDNH
x3.2e+07
Relative Intensity
Δm (ppm)
m/z
y1 /1+
b2 /1+
y2 /1+
y3 /1+
y4 /1+
b4 /1+
y5 /1+
y6 /1+
b9 /1+
b10 /1+
b$1 /1+
b3 /1+
b5 /1+
b6 /1+
b6 /1+
y7 /1+
y8 /1+
y9 /1+
y10 /1+
Y0 /2+
Y /2+
M /2+
Y0 /1+
Y$ /1+
Y /1+
138.06
168.07
186.08
204.09
274.09
292.10
366.14
657.24

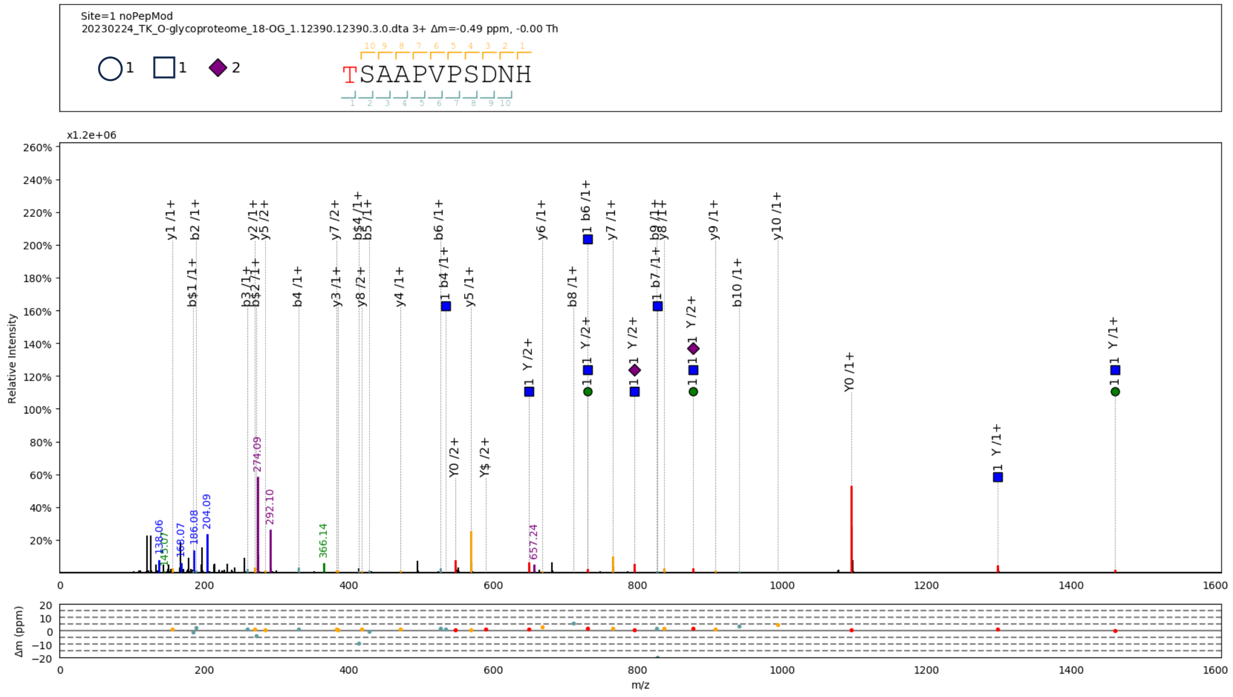

Site=1 noPepMod
20230224_TK_O-glycoproteome_18-OG_1.12390.12390.3.0.dta 3+ Δm=-0.49 ppm, -0.00 Th
TSAAPVPSDNH
x1.2e+06
Relative Intensity
Δm (ppm)
m/z
y1 /1+
b2 /1+
y2 /1+
y5 /2+
y7 /2+
b$4 /1+
b5 /1+
b6 /1+
y6 /1+
b6 /1+
y7 /1+
b9 /1+
y8 /1+
y9 /1+
y10 /1+
b$1 /1+
b3 /1+
b$2 /1+
b4 /1+
y3 /1+
y8 /2+
y4 /1+
b4 /1+
y5 /1+
b8 /1+
b7 /1+
Y /2+
b10 /1+
Y0 /1+
Y0 /2+
Y$ /2+
Y /1+
138.06
168.07
186.08
204.09
274.09
292.10
366.14
657.24

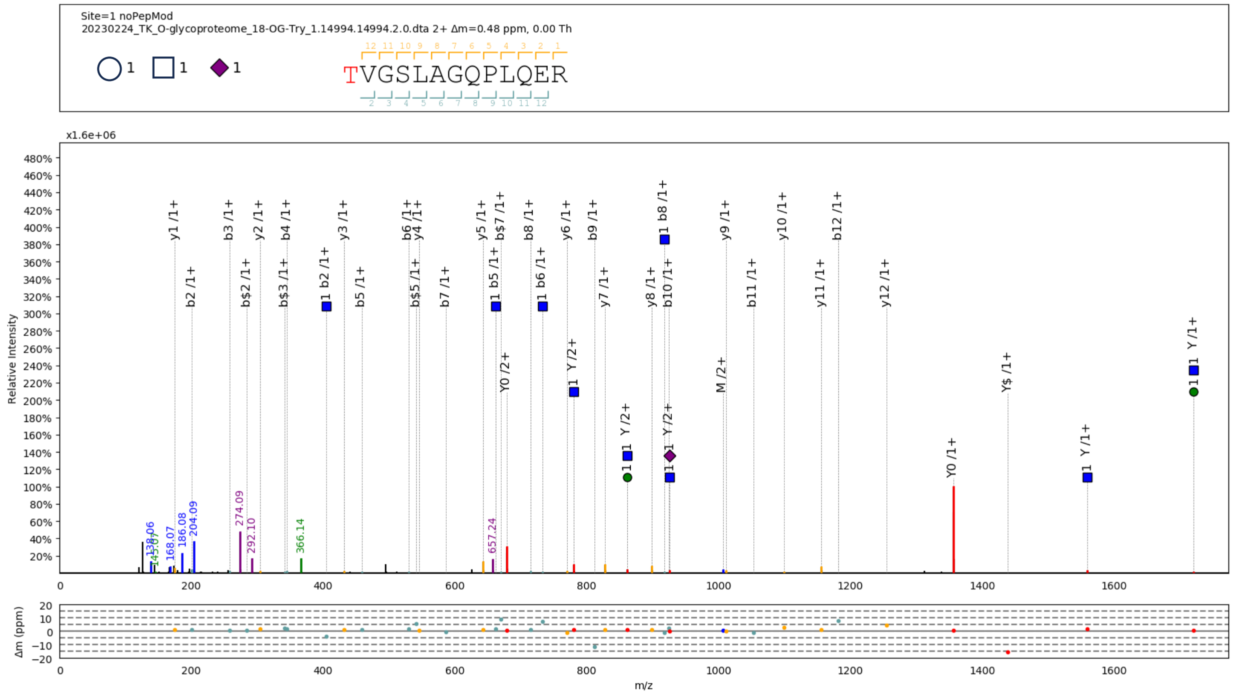
Site=1 noPepMod
20230224_TK_O-glycoproteome_18-OG-Try_1.14994.14994.2.0.dta 2+ Δm=0.48 ppm, 0.00 Th
TVGSLAGQPLQER
x1.6e+06
Relative Intensity
Δm (ppm)
m/z

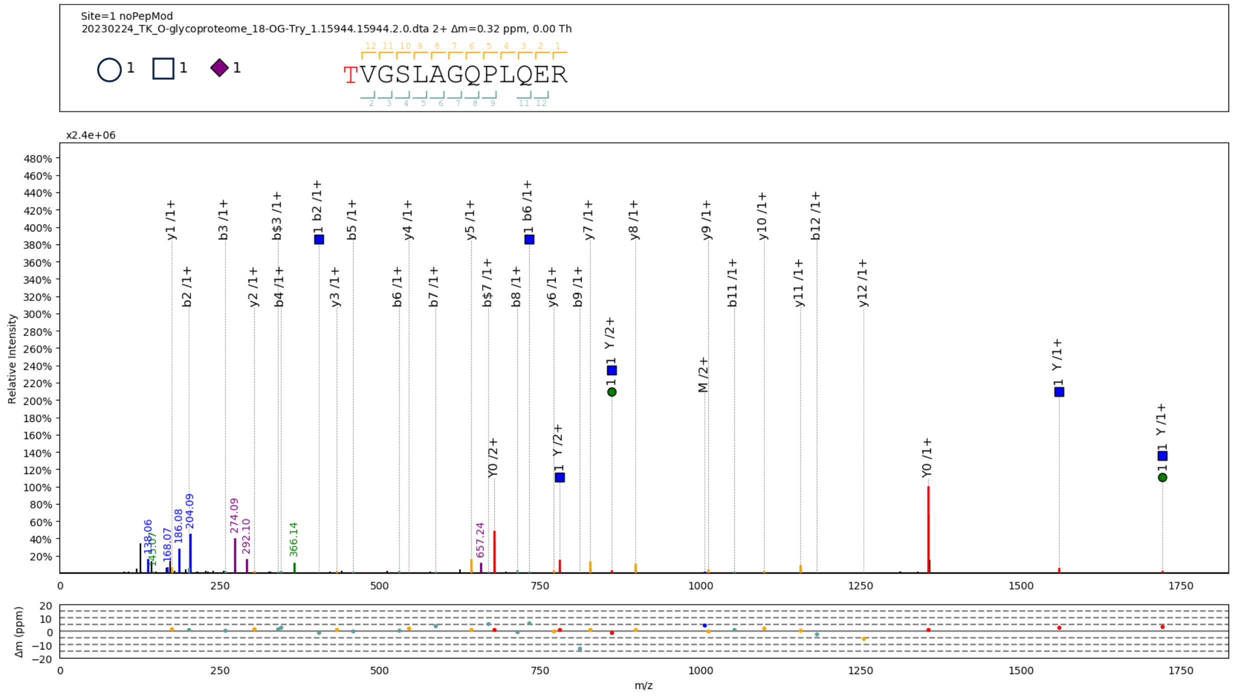
Site=1 noPepMod
20230224_TK_O-glycoproteome_18-OG-Try_1.15944.15944.2.0.dta 2+ Δm=0.32 ppm, 0.00 Th
TVGSLAGQPLQER
x2.4e+06
Relative Intensity
Δm (ppm)
m/z

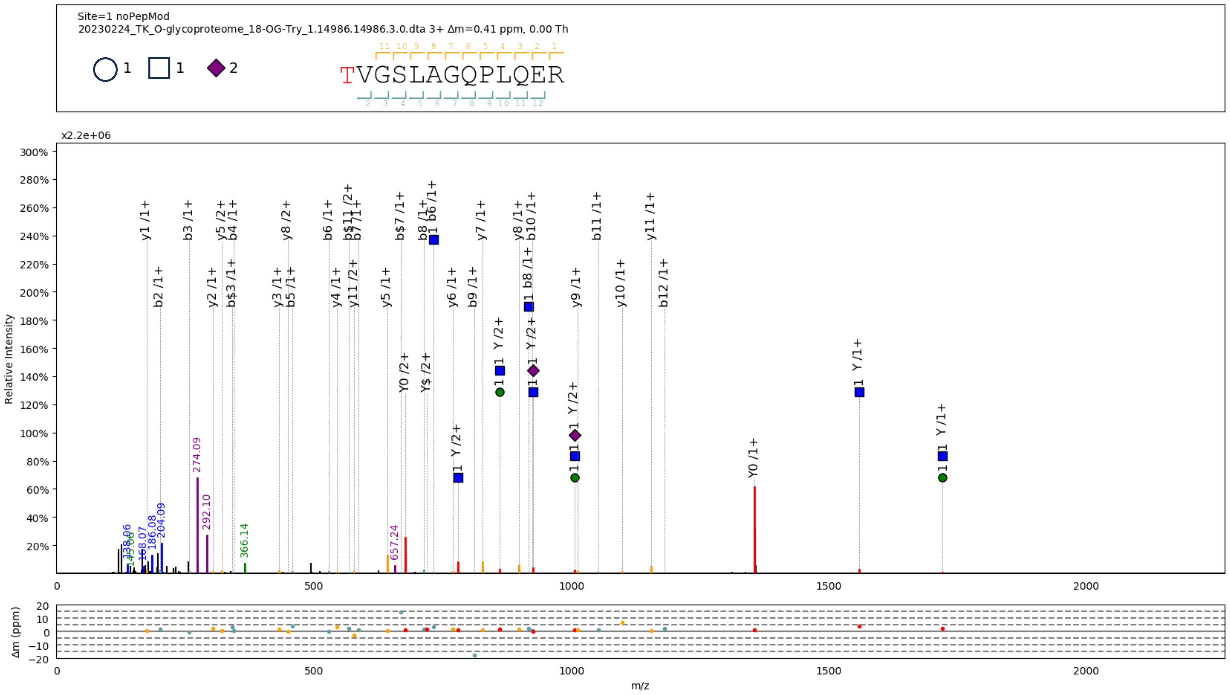
Site=1 noPepMod
20230224_TK_O-glycoproteome_18-OG-Try_1.14986.14986.3.0.dta 3+ Δm=0.41 ppm, 0.00 Th
TVGSLAGQPLQER
Relative Intensity
Δm (ppm)
m/z

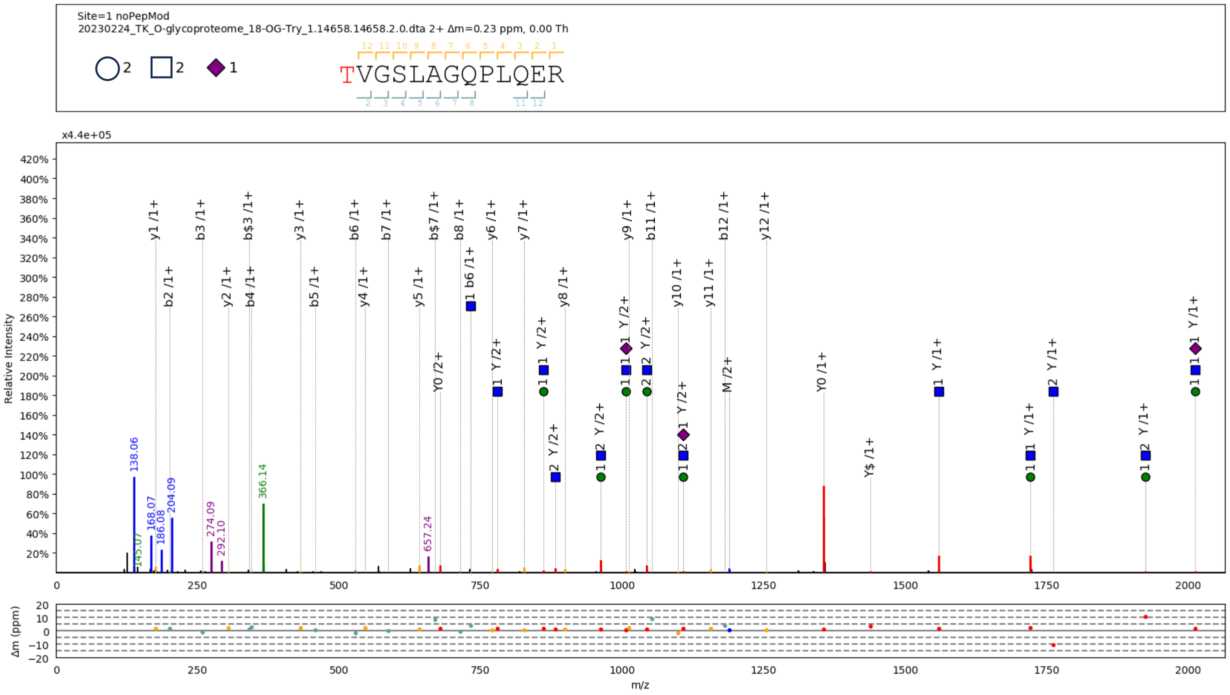
Site=1 noPepMod
20230224_TK_O-glycoproteome_18-OG-Try_1.14658.14658.2.0.dta 2+ Δm=0.23 ppm, 0.00 Th
TVGSLAGQPLQER
Relative Intensity
Δm (ppm)
m/z

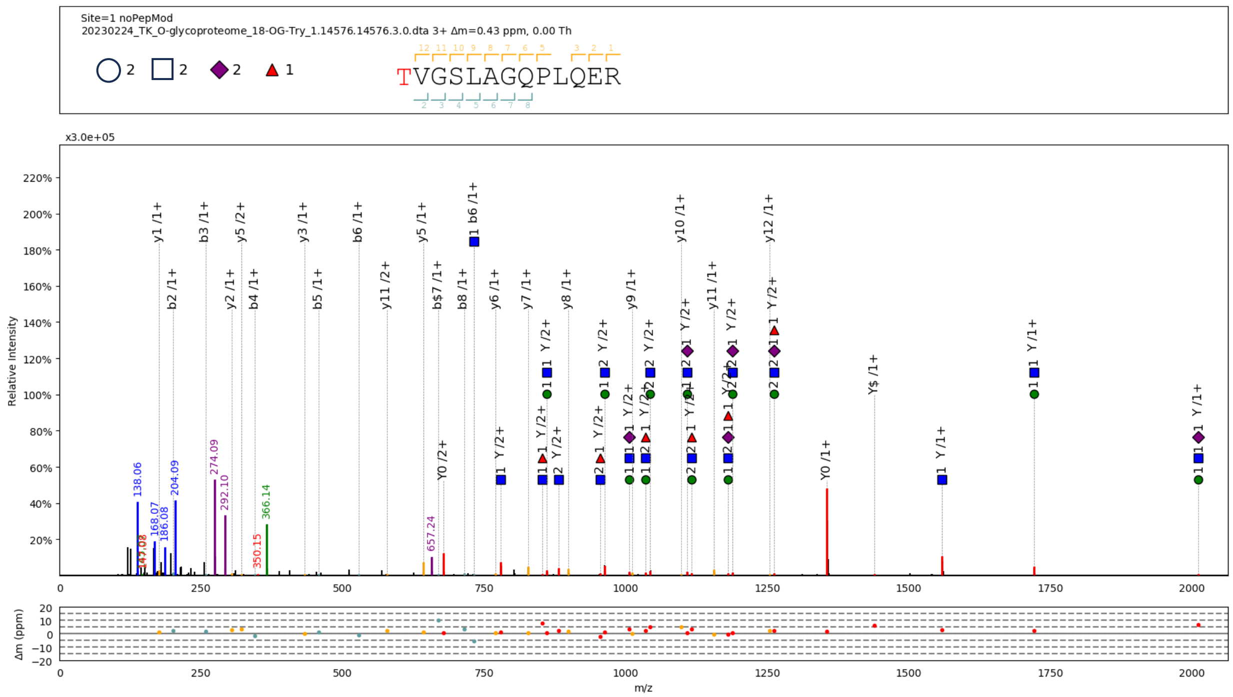

(D) Fibrinogen alpha chain (FGA)

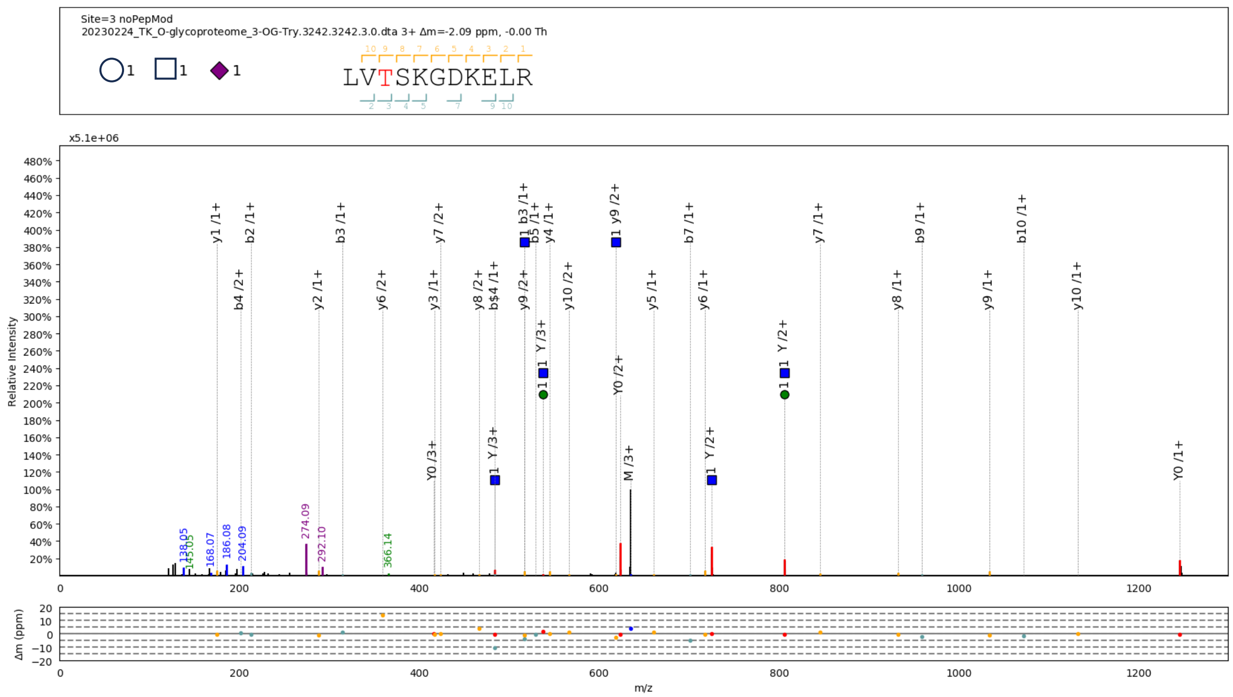

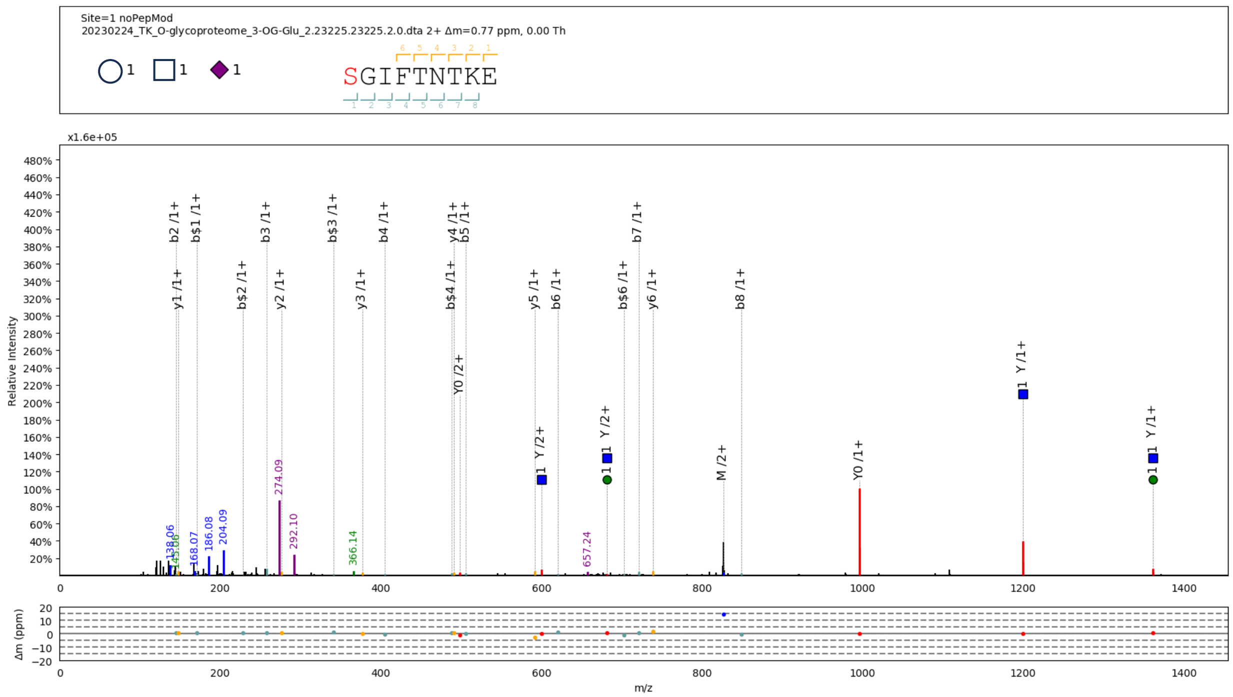
Site=1 noPepMod
20230224_TK_O-glycoproteome_3-OG-Glu_2.23225.23225.2.0.dta 2+ Δm=0.77 ppm, 0.00 Th
SGIFTNTKE
x1.6e+05
Relative Intensity
Δm (ppm)
m/z

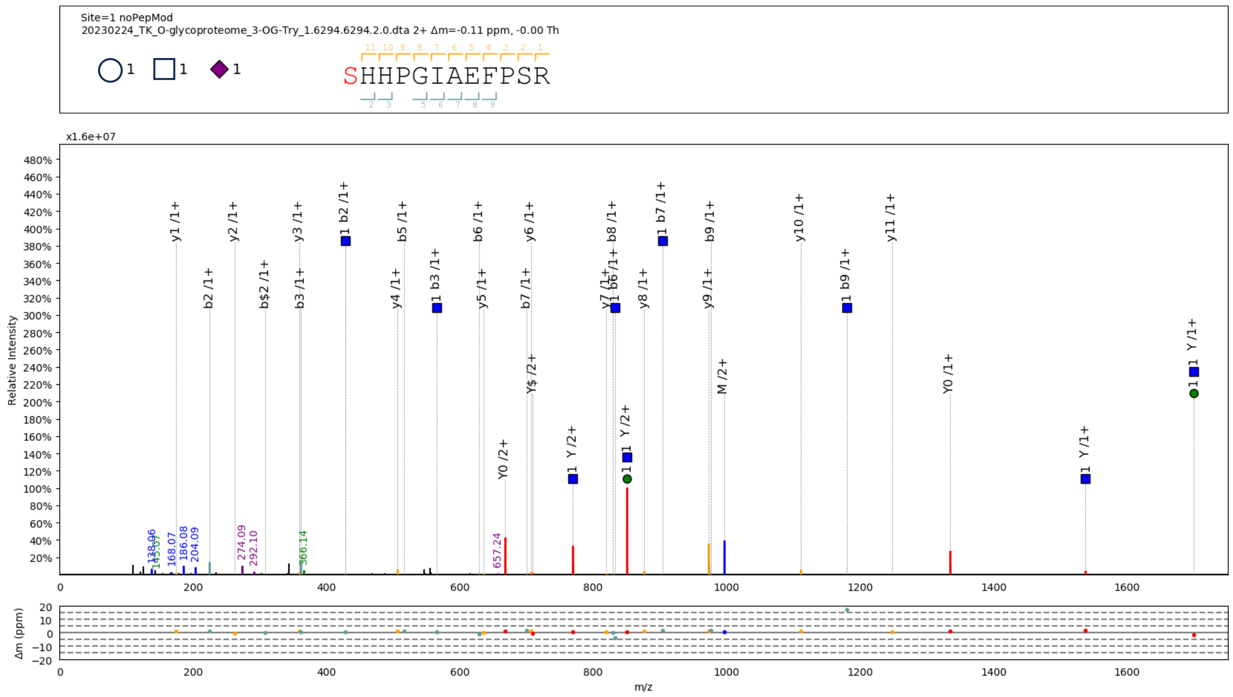
Site=1 noPepMod
20230224_TK_O-glycoproteome_3-OG-Try_1.6294.6294.2.0.dta 2+ Δm=-0.11 ppm, -0.00 Th
SHHPGIAEFPSR
x1.6e+07
Relative Intensity
Δm (ppm)
m/z

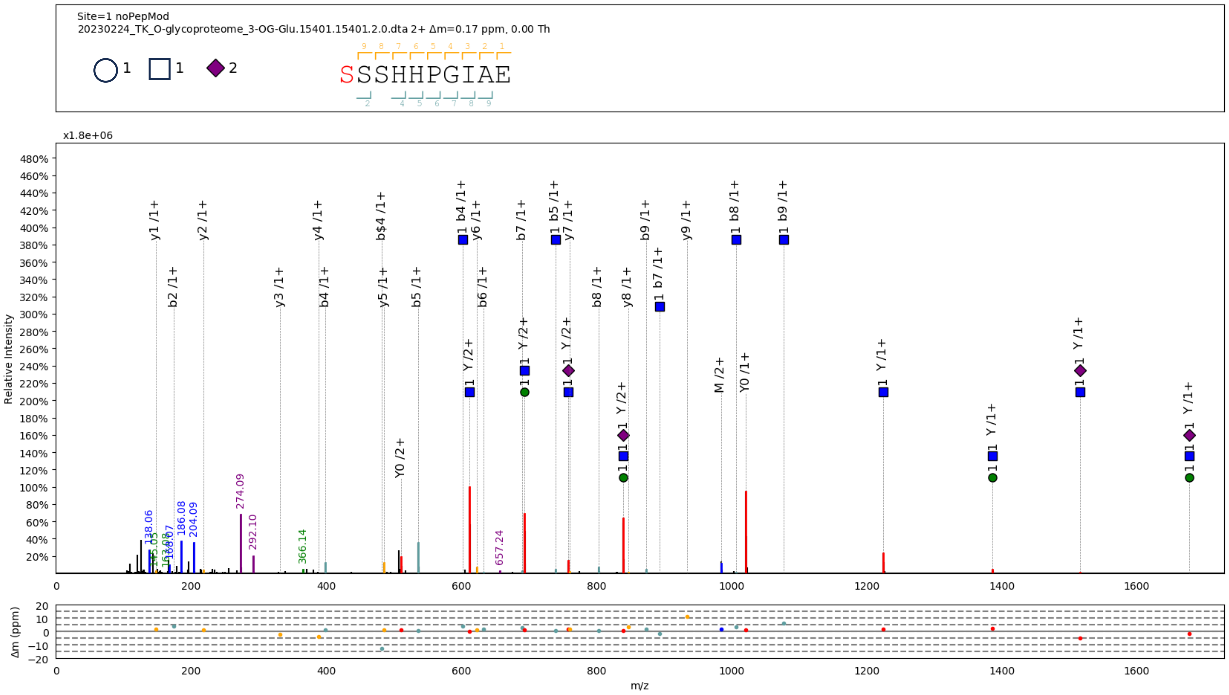
Site=1 noPepMod
20230224_TK_O-glycoproteome_3-OG-Glu.15401.15401.2.0.dta 2+ Δm=0.17 ppm, 0.00 Th
1
1
2
SSSHHPGIAE
x1.8e+06
Relative Intensity
y1 /1+
b2 /1+
y2 /1+
y3 /1+
y4 /1+
b4 /1+
b$4 /1+
y5 /1+
b5 /1+
Y0 /2+
1 b4 /1+
y6 /1+
b6 /1+
1 Y/2+
b7 /1+
1 b5 /1+
y7 /1+
b8 /1+
y8 /1+
b9 /1+
1 b7 /1+
y9 /1+
M /2+
1 b8 /1+
Y0 /1+
1 b9 /1+
1 Y/1+
138.06
186.08
204.09
274.09
292.10
366.14
657.24
Δm (ppm)
m/z

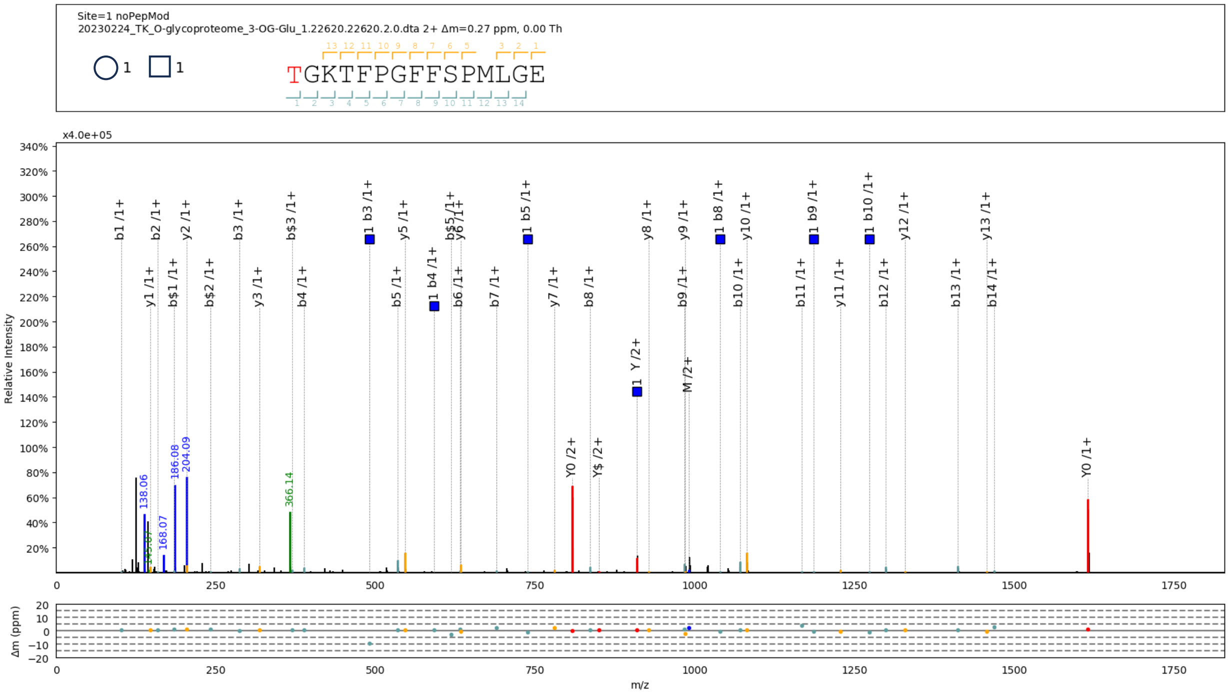
Site=1 noPepMod
20230224_TK_O-glycoproteome_3-OG-Glu_1.22620.22620.2.0.dta 2+ Δm=0.27 ppm, 0.00 Th
1
1
TGKTFPGFFSPMLGE
x4.0e+05
Relative Intensity
b1 /1+
y1 /1+
b2 /1+
b$1 /1+
y2 /1+
b$2 /1+
b3 /1+
y3 /1+
b$3 /1+
b4 /1+
1 b3 /1+
b5 /1+
y5 /1+
1 b4 /1+
b$5 /1+
y6 /1+
b6 /1+
b7 /1+
1 b5 /1+
y7 /1+
Y0 /2+
b8 /1+
Y$ /2+
1 Y/2+
y8 /1+
y9 /1+
b9 /1+
M /2+
1 b8 /1+
b10 /1+
y10 /1+
b11 /1+
1 b9 /1+
y11 /1+
1 b10 /1+
b12 /1+
y12 /1+
b13 /1+
y13 /1+
b14 /1+
Y0 /1+
138.06
168.07
186.08
204.09
366.14
Δm (ppm)
m/z

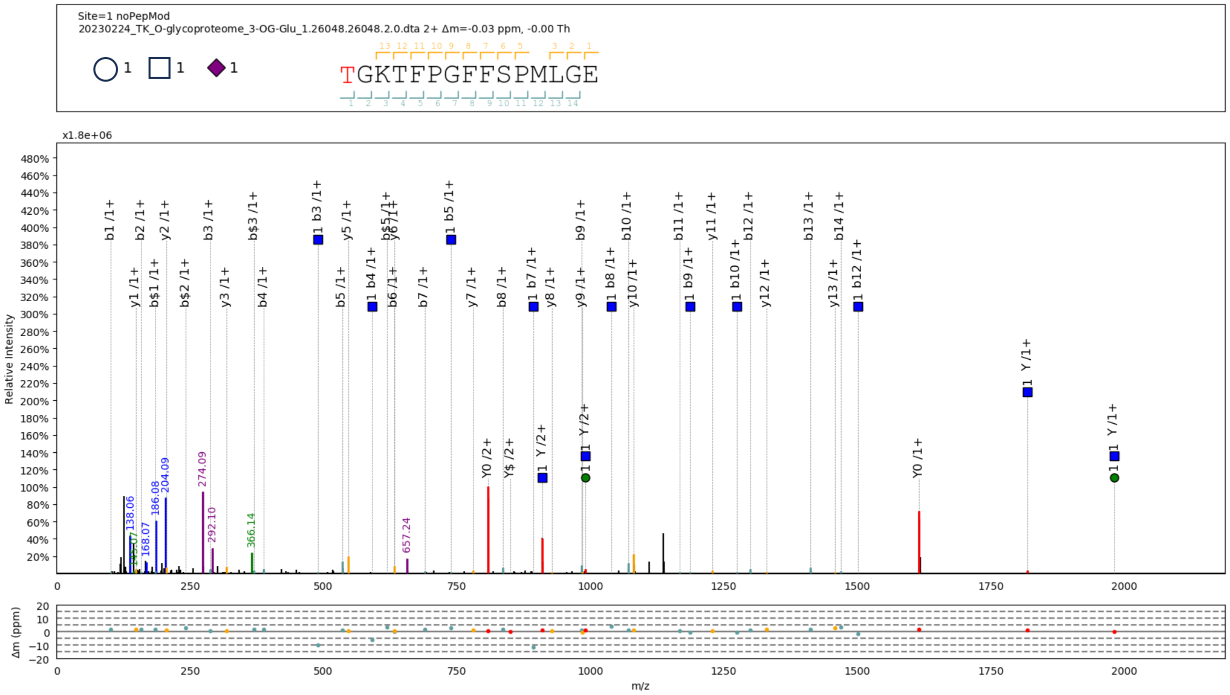
Site=1 noPepMod
20230224_TK_O-glycoproteome_3-OG-Glu_1.26048.26048.2.0.dta 2+ Δm=-0.03 ppm, -0.00 Th
TGKTFPGFFSPMLGE
x1.8e+06
Relative Intensity
Δm (ppm)
m/z

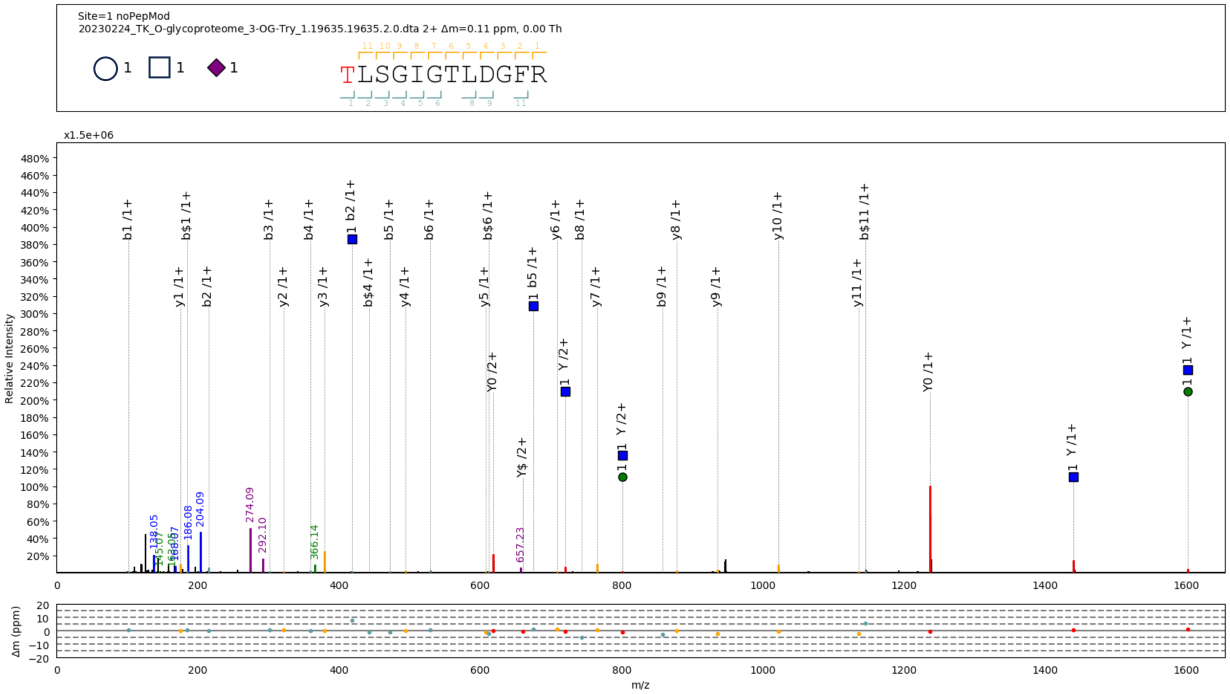
Site=1 noPepMod
20230224_TK_O-glycoproteome_3-OG-Try_1.19635.19635.2.0.dta 2+ Δm=0.11 ppm, 0.00 Th
TLSGIGTLDGFR
x1.5e+06
Relative Intensity
Δm (ppm)
m/z

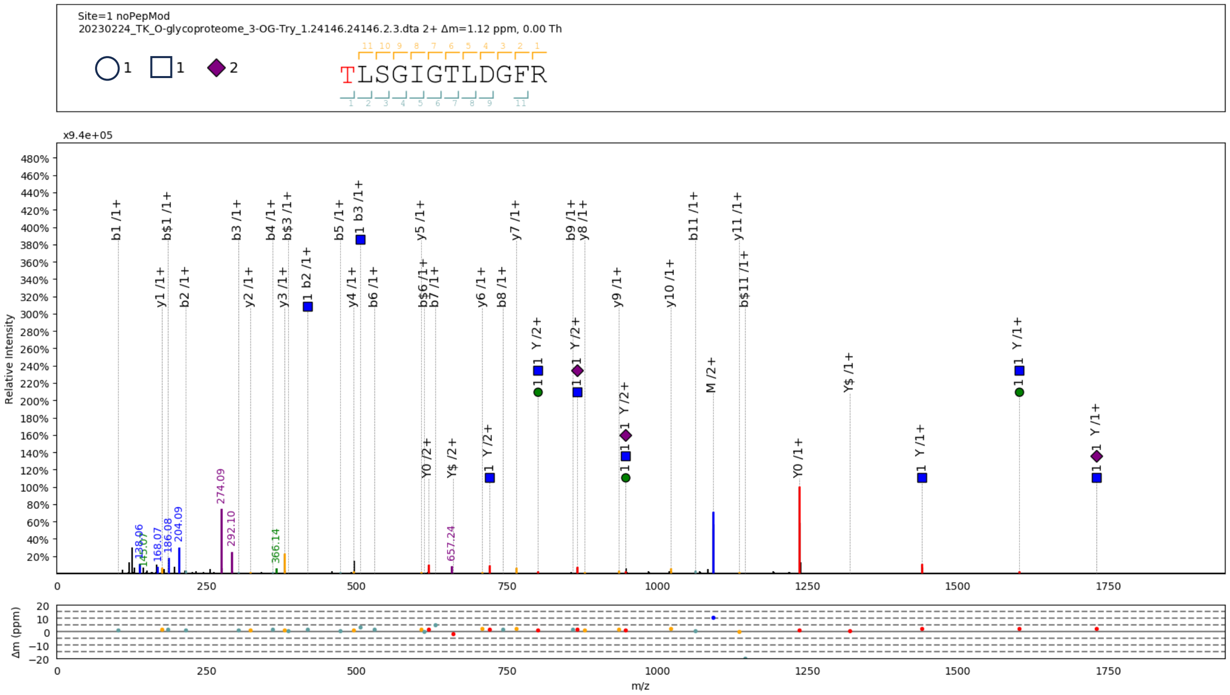
Site=1 noPepMod
20230224_TK_O-glycoproteome_3-OG-Try_1.24146.24146.2.3.dta 2+ Δm=1.12 ppm, 0.00 Th
TLSGIGTLDGFR
x9.4e+05
Relative Intensity
Δm (ppm)
m/z

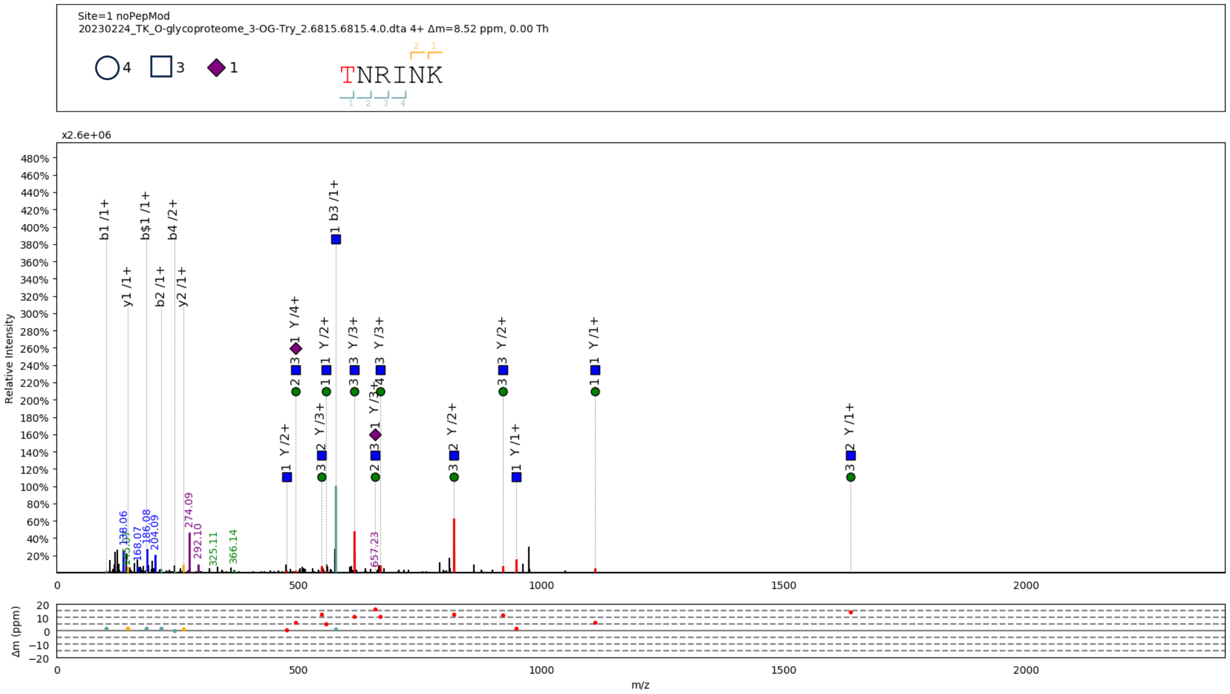
Site=1 noPepMod
20230224_TK_O-glycoproteome_3-OG-Try_2.6815.6815.4.0.dta 4+ Δm=8.52 ppm, 0.00 Th
TNRINK
x2.6e+06
Relative Intensity
Δm (ppm)
m/z

(E) Fibrinogen beta chain (FGB)

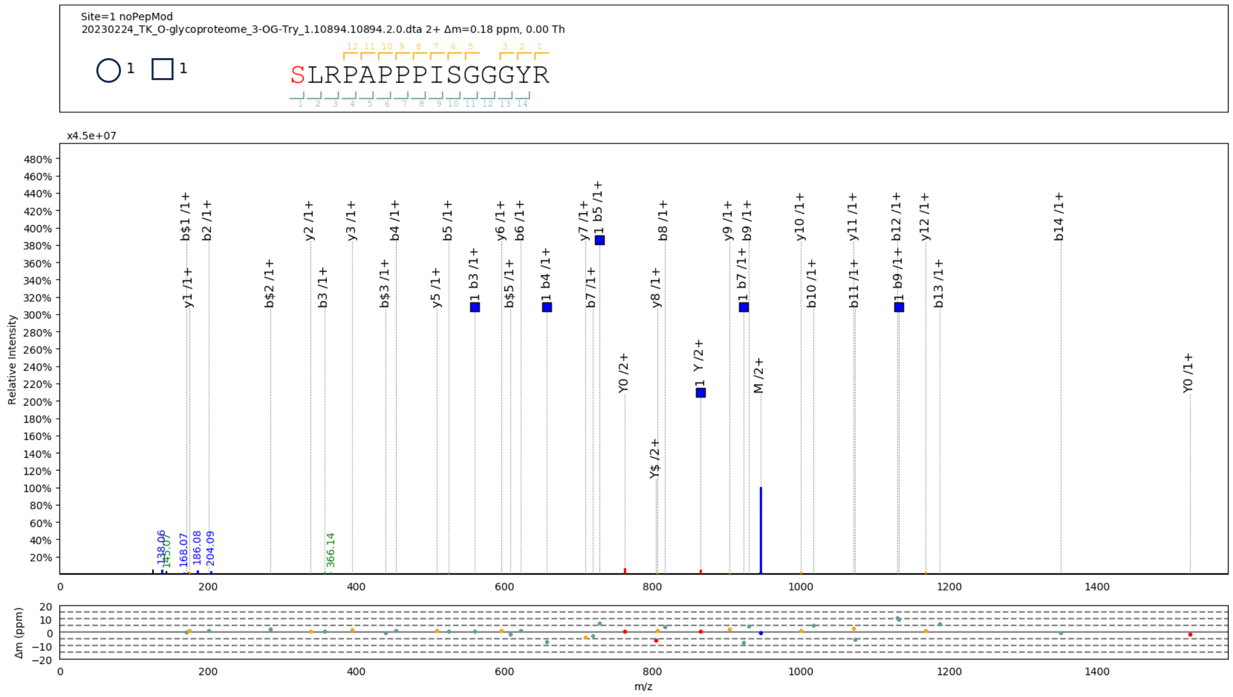

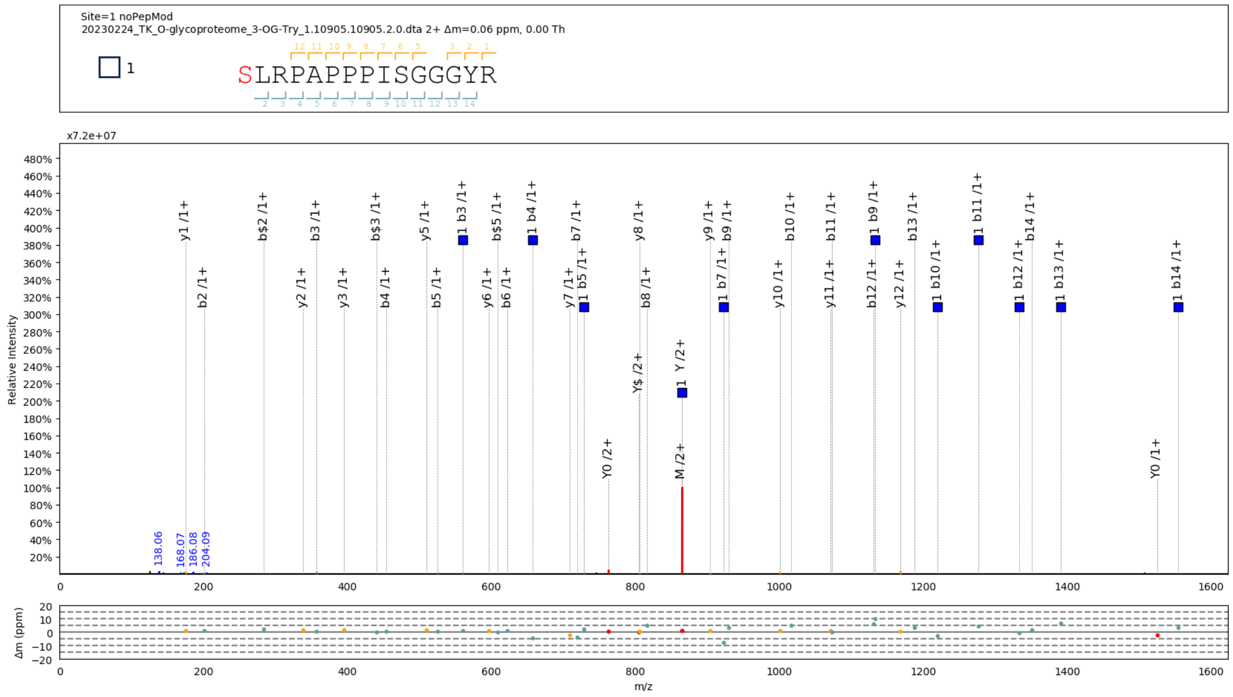

Supplement: Document S2. Article plus supplemental information [file mmc4.pdf]
